# Synthesis, Modeling, and Biological Properties of Fluoroprostacyclin Analogues: Potent Agonists for Prostanoid Receptors

**DOI:** 10.1021/acs.jmedchem.6c00630

**Published:** 2026-07-25

**Authors:** Changcheng Jing, Isabel Perez-Powell, Hannah Baars, Shahida Mallah, Justin S. Trory, Robin A. Corey, Stuart J. Mundell, Xin Xu, Jay Tromans, Samantha F. Moore, Christopher J. Arthur, Ingeborg Hers, Varinder K. Aggarwal

**Affiliations:** † School of Chemistry, 1980 University of Bristol, Cantock’s Close, Bristol BS8 1TS, U.K.; ‡ Bristol Medical School, University of Bristol, University Walk, Biomedical Sciences Building, Bristol BS8 1TD, U.K.; ◊ School of Psychology & Neuroscience, University of Bristol, University Walk, Biomedical Sciences Building, Bristol BS8 1TD, U.K.

## Abstract

Prostacyclin (PGI_2_, epoprostenol) and its more stable analogues iloprost and cicaprost are used in the treatment of pulmonary arterial hypertension (PAH) and other related diseases. Currently, PGI_2_ therapy is the most effective treatment for PAH, but is administered intravenously due to its instability under physiological conditions. We considered creating more chemically stable hybrids of PGI_2_ by merging essential features of iloprost/cicaprost with a more stable C-7 fluorinated PGI_2_, which maintained the cyclic enol ether. The synthesis employed our key bicyclic enal and furnished the required PGI_2_ analogues in just 7–8 steps, providing the most expedient route to this class of molecules. This led to the discovery of compound **9**, a picomolar-potent, IP receptor-selective, and chemically stable PGI_2_ analogue that combined the ω-side chain of cicaprost with the C-7 difluorinated enol ether of PGI_2_. This compound provides the most potent PGI_2_ analogue tested to date.

## Graphical abstract

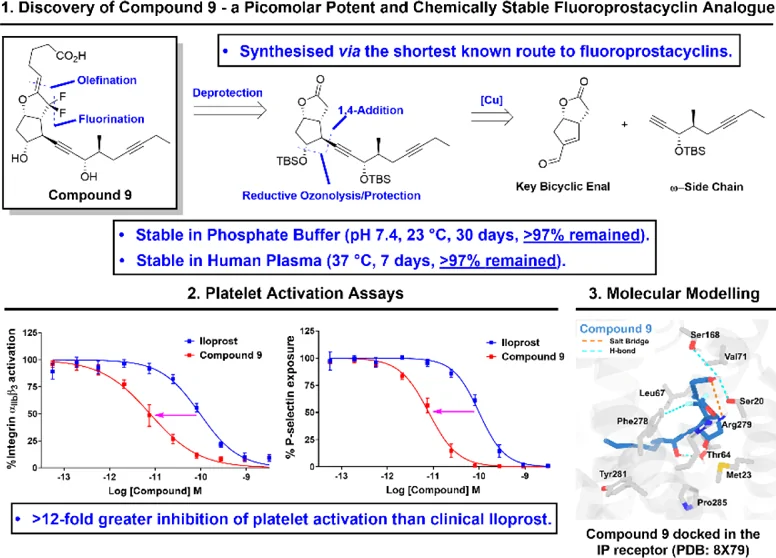

## 1 Introduction

Prostacyclin (PGI_2_, epoprostenol) and its analogues are used in the treatment of pulmonary arterial hypertension (PAH) and less commonly as antithrombotic treatment in intensive care.^1–5^ PAH is a progressive disease characterized by elevated pulmonary arterial pressure and pulmonary vascular resistance, leading to right ventricular failure and death.^6–9^ At present, PGI_2_ therapy is one of the most effective treatments for PAH, but it has to be administered intravenously due to its short chemical half-life (*t*_1/2_ = 10.5 min, pH = 7.48)^10,11^ and metabolic half-life (*t*_1/2_ = 3–6 min, human whole blood).^12–14^ The short chemical half-life is a consequence of the rapid hydrolysis of the enol ether under physiological conditions.^15^ More stable C-7 mono- ^11,16,17^ (PGI_2_–F) and difluorinated (PGI_2_–F_2_) analogues^18^ have been developed with substantially enhanced chemical half-lives (Figure 1, PGI_2_-F > 30 days, PGI_2_–F_2_ > 90 days). Both analogues show similar activity to PGI_2_; however, for PGI_2_–F, its activity is dependent on the stereochemistry of the fluorine atom.^11^ The presence of the electron-withdrawing fluorine atom destabilizes a developing oxocarbenium ion formed upon protonation of the enol ether, lowering reactivity toward hydrolysis and leading to increased chemical stability. Beyond C-7 fluorination, fluorination of the C-5 position^19^ and difluorination of the C-10 position^20^ have also been explored, and these analogues also show enhanced stability over PGI_2_. 5-Fluoroprostacyclin is stable for 24 h at pH 9.0 (24 °C),^19^ whereas 10,10-difluoroprostacyclin has a half-life of 24 h in Krebs buffer solution (pH 7.4, 37 °C);^20^ both analogues are equipotent to PGI_2_. Although fluorination of different positions of PGI_2_ is well explored, almost all fluoroprostacyclins retain the ω-side chain of PGI_2_.

**Figure 1:**
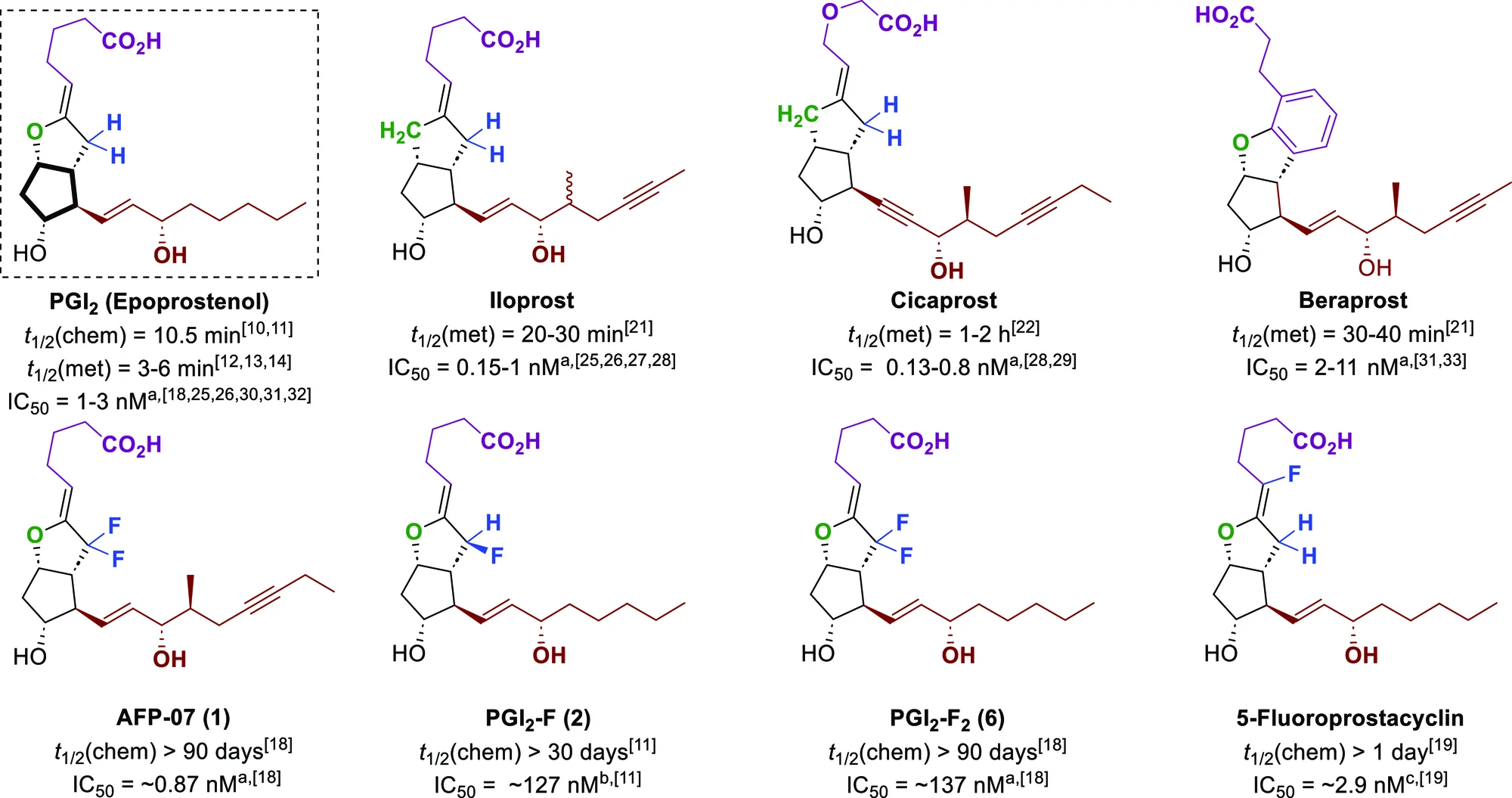
PGI_2_ drugs and analogues. The chemical structures of various PGI_2_ analogues and their reported half-life (*t*_1/2_ (chem) = chemical half-life, *t*_1/2_ (met) = metabolic plasma half-life) and biological activity [drug concentration required for 50% inhibition of *in vitro* ADP-stimulated platelet aggregation (IC_50_)]. ^a^IC_50_ for inhibition of human platelet aggregation. ^b^IC_50_ for inhibition of rabbit platelet aggregation. ^c^IC_50_ for inhibition of rat platelet aggregation.

In addition, analogues devoid of the enol ether have been developed that have substantially longer half-lives than PGI_2_,^21,22^ e.g., iloprost, cicaprost, and beraprost, and these compounds can be administered by alternative methods (inhalation^23^ or orally^24^). The improved half-lives of these analogues are attributed to replacement of the hydrolytically sensitive enol ether with a more chemically stable alkene (iloprost and cicaprost) or aromatic ring (beraprost). Iloprost^25–28^ and cicaprost^28,29^ are the most active molecules in their class (Figure 1). All PGI_2_ analogues share the same stereodefined cyclopentane ring but with different α- and ω-side chains. We hypothesized that combining the ω-side chains of beraprost, iloprost, or cicaprost with the bicyclic core of PGI_2_, together with the introduction of fluorine atoms to stabilize the enol ether, would create PGI_2_ hybrids with improved function in terms of stability, potency, and/or selectivity. Encouragingly, Matsumura conducted a limited study of PGI_2_ analogues, which showed that difluorinated analogue **1** (AFP-07), with an ω-side chain similar to iloprost, was a highly potent inhibitor of platelet aggregation.^18,34^ Notably, this study focused on 7,7-difluorinated analogues. Difluorination often leads to a widening of the bond angle and increased polarization relative to the hydrocarbon.^35^ Indeed, in a comparative study of the CH_2_–, CHF–, and CF_2_– phosphonate analogues of *syn*-glycerol-3-phosphate, O’Hagan found that the monofluorinated substrate was the optimum substrate for the dehydrogenase enzyme.^36^ We were, therefore, particularly interested in introducing a single fluorine atom at C-7, as this modification was expected to preserve sufficient enol ether stability while maintaining physicochemical properties comparable to those of the parent compound, PGI_2_, thereby providing an optimal balance between stability and structural mimicry of the natural product.^30–33^ PGI_2_–F has previously been synthesized via prostaglandin F2α (PGF_2α_) in 25 steps (16 steps from the Corey lactone), with some of the steps suffering from low stereo- or regiocontrol.^37,38^ Similarly, the shortest synthesis of PGI_2_–F_2_, to date, is by Matsumura and co-workers, and required 19 steps (10 steps from the Corey lactone).^18^

We, therefore, set out to prepare a set of PGI_2_ analogues (Figure 2) having the same α-side chain and enol ether as the parent compound, stabilized by one (**2**, **3**, **4,** and **5**) or two fluorine (**6**, **7**, **8,** and **9**) atoms, but with a range of ω-side chains found in PGI_2_ itself and related analogues, and to examine their biological activity. We decided to focus on C-7 fluorination, as these analogues boast the greatest chemical stability over C-5 or C-10 fluorination. Although compounds **2** (PGI_2_–F) and **6** (PGI_2_–F_2_) had previously been reported, we wanted to compare their biological activity with the activity of our novel fluoroprostacyclins. The analogous compounds **4** and **8** differ only in ω-side-chain geometry, containing an alkyne in place of the *E*-alkene present in **2** and **6**; as both motifs have been employed in PGI_2_ analogues, we sought to evaluate the optimal geometry at this position. Finally, compounds **3** and **7** incorporate the ω-side chain of beraprost (a stereoisomer of the iloprost ω-side chain), whereas compounds **5** and **9** share the ω-side chain of cicaprost. The *anti* diastereoisomers of the ω-side chains in compounds **3**, **5**, **7**, and **9** were selected because it was previously found to be more active than the *syn* isomer of compound **1**.^18^

**Figure 2:**
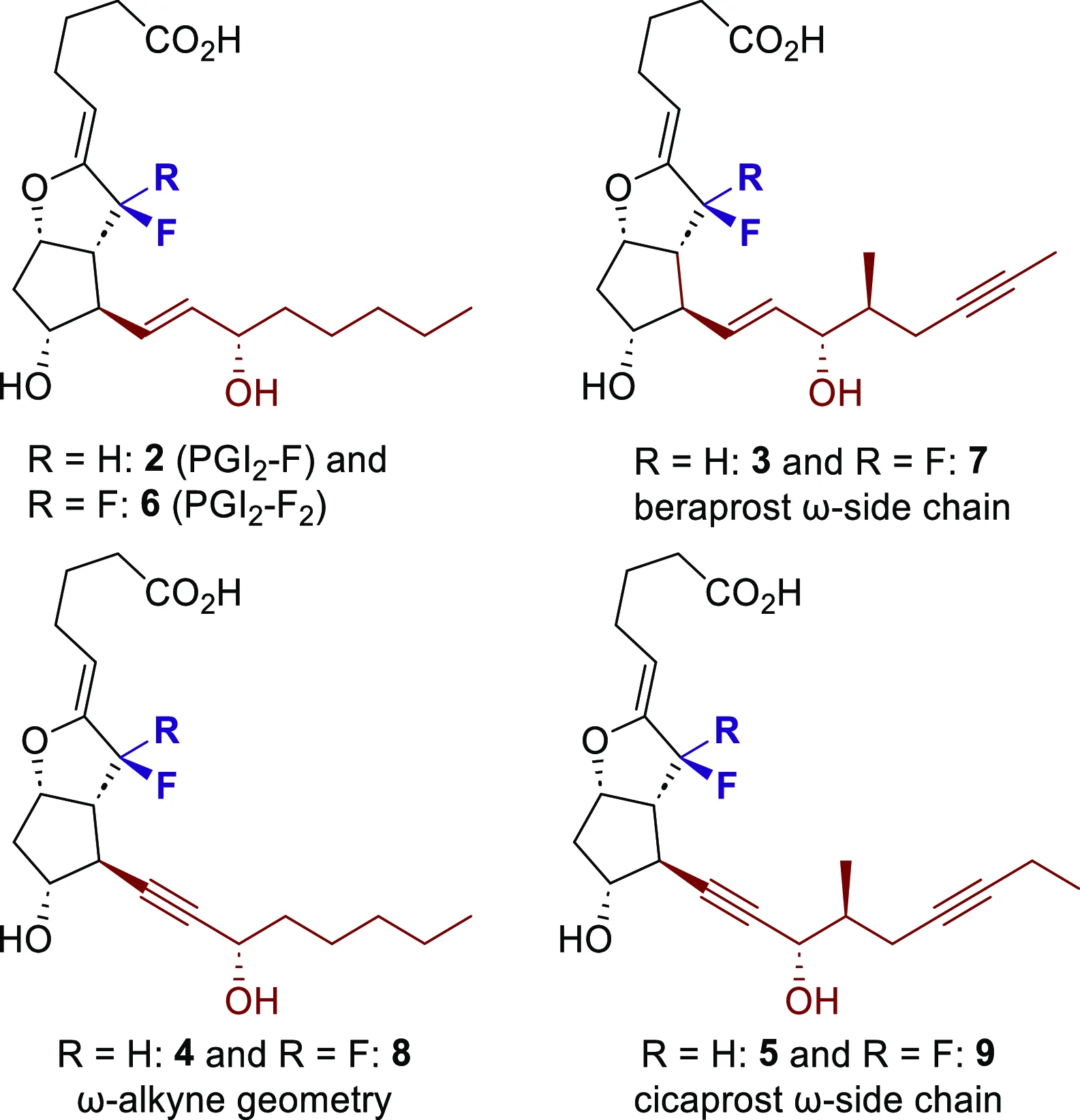
Fluorinated PGI_2_ targets.

Part of our motivation for studying the prostacyclins lay in our previously described syntheses of PGF_2α_.^39,40^ The original synthesis was achieved in just 7 steps, using a double aldol reaction of succinaldehyde to prepare the key enal intermediate **10**. The yield of this key step in the original synthesis was 14%, but this has recently been improved to provide enal **10** in 29% yield on a 50 g scale (Scheme 1).^41,42^ To demonstrate the utility of enal **10,** we synthesized the antiglaucoma drug latanoprost in 6 steps and >99% ee.^43^ We were keen to extend this chemistry further and in particular to demonstrate its application to much shorter syntheses of other important prostanoids, like the prostacyclins. Just as the Corey lactone has been used for the preparation of a number of prostanoids,^44,45^ we see our enal intermediate **10** as being perfectly set up for further transformations to access other members of the larger family of prostanoids in a dramatically shorter manner.

**Scheme 1:**
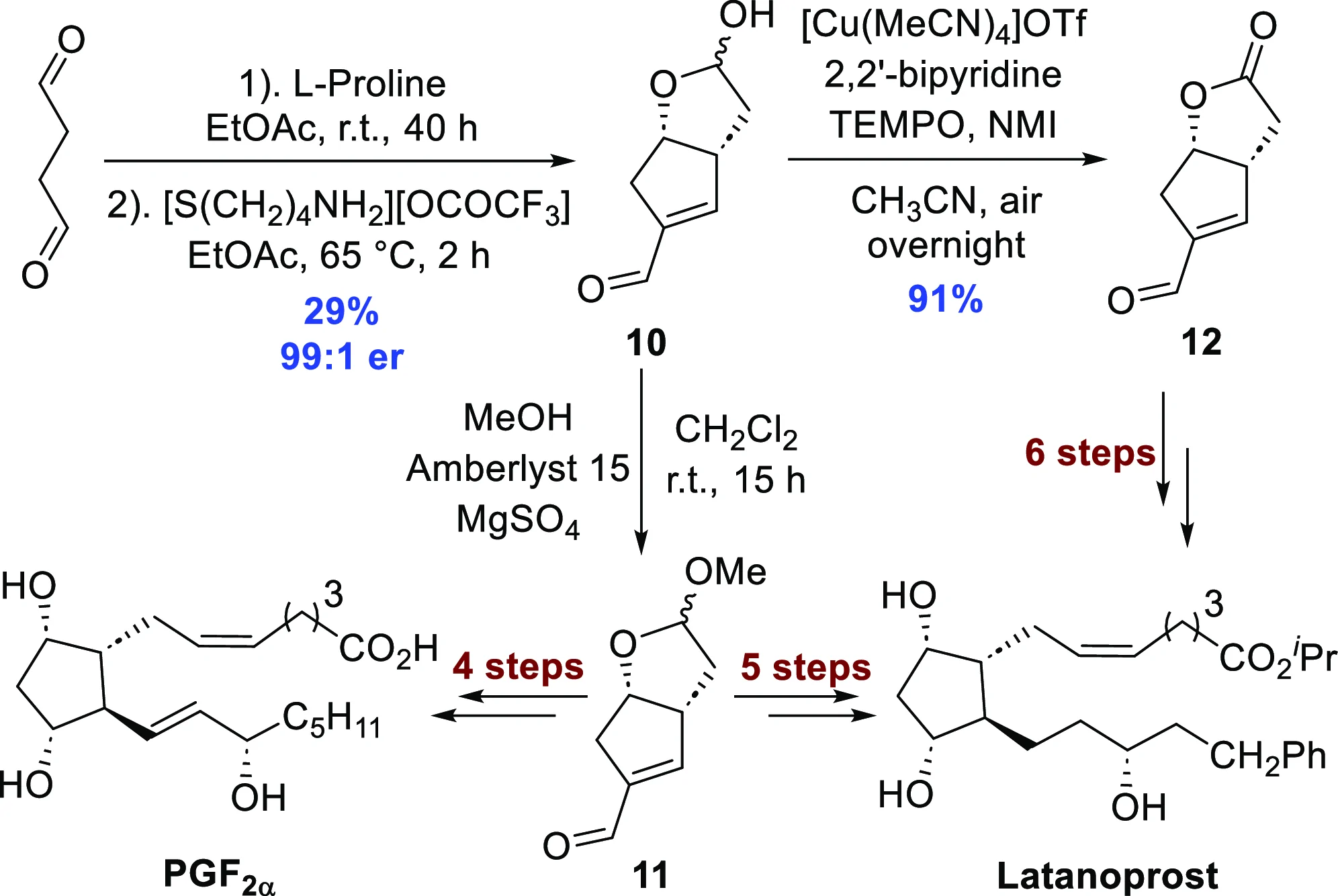
Aggarwal Route to Prostanoids *via* Key Bicyclic Enals

## 2 Results and Discussion

Our retrosynthetic analysis for the set of PGI_2_ analogues is shown in Scheme 2. We envisaged that the enol ether could be constructed by some type of alkenylation reaction of the corresponding fluorinated lactone. This could be obtained by fluorination of the lactone, which itself could be obtained by conjugate addition of the ω-side chain to the key enal-lactone reported previously.^43^

**Scheme 2:**
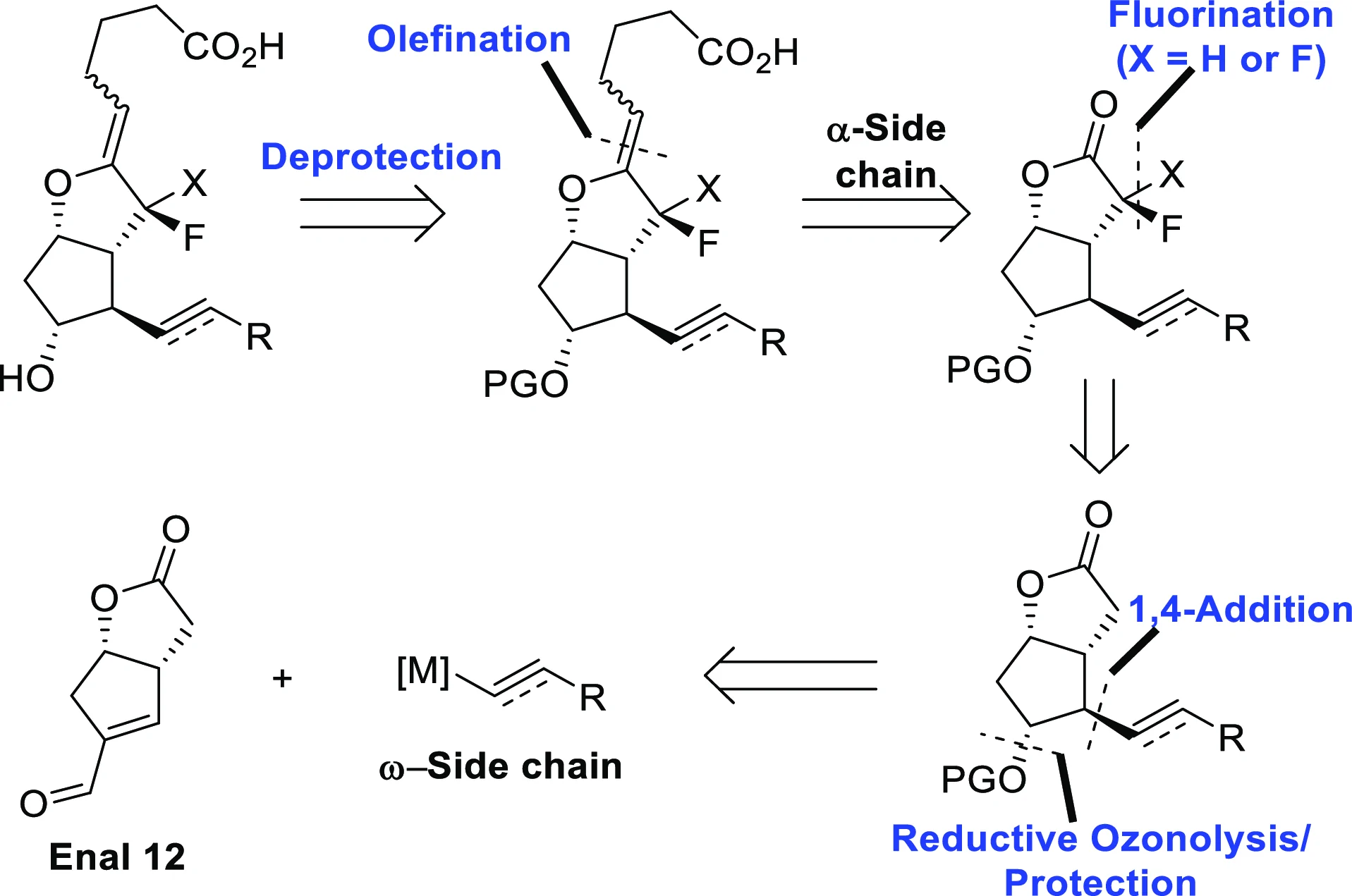
Retrosynthesis of Fluoro PGI_2_ Analogues from the Bicyclic Enal **12**

### 2.1 Synthesis of ω-Side Chains

The synthesis of the ω-side chain required for compounds **2**/**6** has been previously described,^46,47^ and the ω-side chain required for **4**/**8** is commercially available. The synthesis of the side chain required for **3**/**7** is shown in Scheme 3a. Aldehyde **13** ^48^ was subjected to a prolinol-catalyzed aldol reaction with propionaldehyde as described by Hayashi,^49^ followed by *in situ* reduction with NaBH_4_, giving diol **14** in good yield with high enantio- and diastereoselectivity. Selective transformation of the primary hydroxy group to a triflate and the secondary hydroxy group to the TBS ether was achieved in one pot by treatment with triflic anhydride followed by TBSOTf in the presence of 2,6-lutidine. Subsequent reaction of triflate **15** with propynyllithium furnished dialkyne **16**, which after selective deprotection of the alkyne moiety under basic conditions gave dialkyne **17**. Selective hydrozirconation of the terminal alkyne with the Schwartz reagent generated *in situ*, followed by iodination, provided the required vinyl iodide **18**. The synthesis of the side chain required for **5**/**9** is shown in Scheme 3b and followed essentially the same route. The reaction between triflate **15** and butynyllithium furnished dialkyne **19,** which was deprotected under basic conditions to give ω-side chain **20**.

**Scheme 3:**
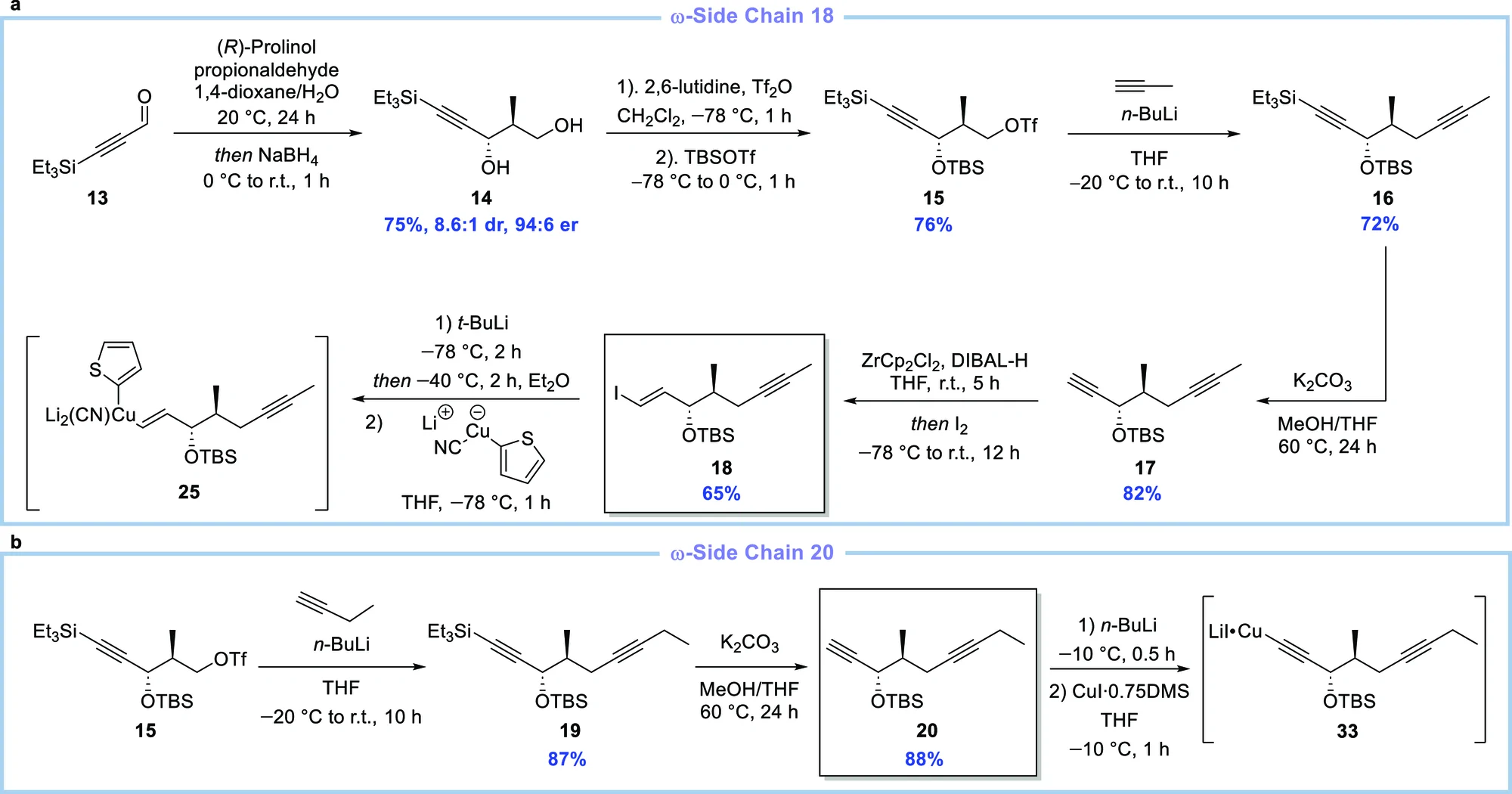
(a) Synthesis of ω-Side Chain 18; (b) Synthesis of ω-Side Chain **20**

### 2.2 Conjugate Addition of ω-Side Chains and the Routes to the Key Precursors

The enal **12** required for 1,4-addition of the ω-side chains was prepared using the improved aldol reaction we recently reported, followed by oxidation (Scheme 2).^41^ We initially focused on the synthesis of the mono- and difluorinated alkene targets **2**, **3**, **6,** and **7** (Figure 2). The ω-side chains were transformed into the corresponding cuprates **21** and **25** and added to enal **12,** and the enolate was trapped as the TMS-enol ether, which was directly submitted to ozonolysis, followed by reduction with NaBH_4_ (Scheme 4). This protocol gave alcohols **23** and **27** in good yields over 2 steps with essentially complete stereoselectivity; the cuprate and subsequent hydride were added from the *exo*-face of the bicyclic lactone. Protection of the alcohol as the TBS-ether furnished precursors **24** and **28**.

**Scheme 4:**
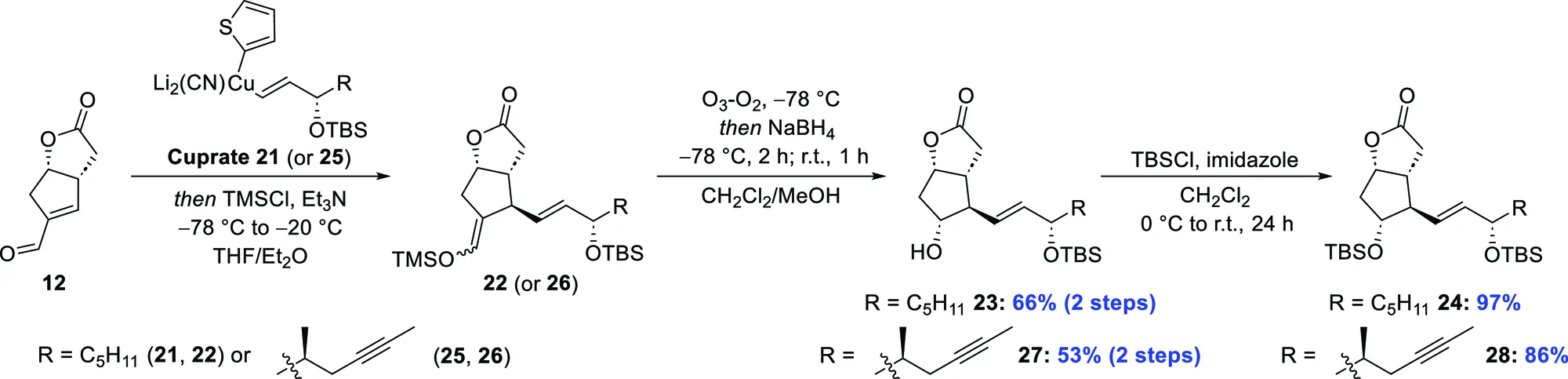
Synthesis of the Precursor for Alkene Targets **24** and **28**

For the alkyne targets, we needed to carry out a 1,4-addition of the alkyne to the enal. This is usually difficult since the alkyne of a copper acetylide often acts as a nontransferable group.^50^ However, it has been demonstrated that this transformation is possible using iodotrimethylsilane as an activator.^51^ Thus, the reaction of enal **12** with alkynyl cuprates **29** and **33** gave TMS-enol ethers **30** and **34**, respectively, which were directly subjected to the ozonolysis and reduction sequence (Scheme 5). In order to obtain high yields in the ozonolysis step, it was necessary to wash intermediates **30** and **34** with a solution of aqueous sodium thiosulfate to remove any traces of iodide or copper salts. After silyl protection, alkyne precursors **32** and **36** were obtained.

**Scheme 5:**
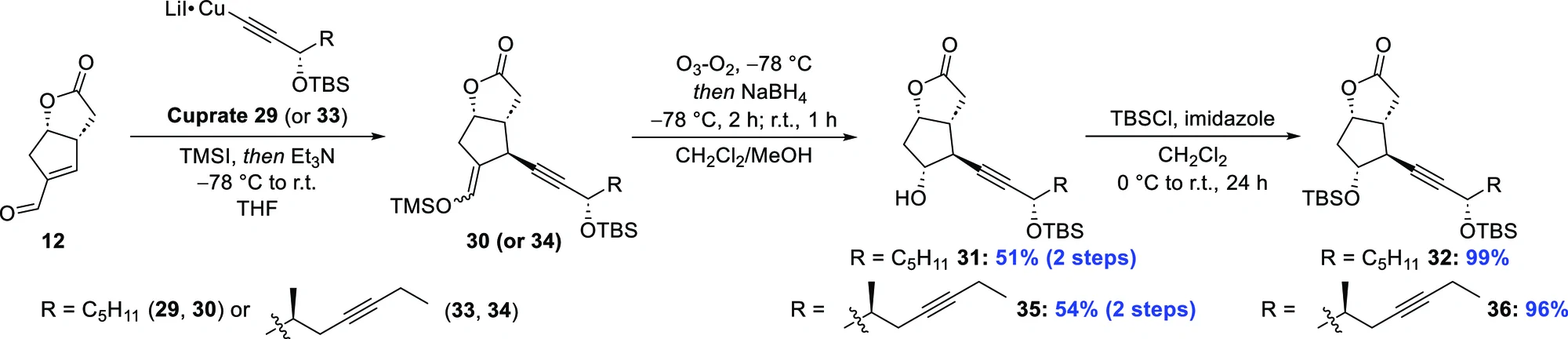
Synthesis of Precursor for Alkyne Targets **32** and **36**

### 2.3 Fluorination, Introduction of α-Side Chains, and Completion of Targets

We initially focused on the difluorinated targets and planned to apply the strategy reported by Matsumura.^34^ He showed that *gem*-difluorination of lactones could be achieved using KHMDS and NFSI in the presence of MnBr_2_. However, employing these conditions to lactone **24** gave the desired difluorinated product **37** in only 15% yield together with 9% of the monofluorinated product. We, therefore, screened other additives and found that ZrCp_2_Cl_2_ was more effective,^18^ giving the difluorinated product **37** in 53% yield (Scheme 6). These conditions were applied to the other alkene and alkyne analogues. In all cases, the difluorinated products were obtained in good yields, showing the generality of these new conditions. We then wished to convert the lactone into the enol ether. It is normally challenging to engage lactones in the Wittig reaction,^52–56^ but Matsumura showed that difluorinated lactones were suitable substrates.^34^ We, therefore, explored the Wittig reaction between (4-carboxybutyl)­triphenyl-phosphonium bromide and lactone **37** and found that the enol ether was formed in moderate yield and good *Z*-selectivity. The stereochemistry of the trisubstituted alkene was established by proton-carbon scalar coupling constants (see Supporting Information for detailed information). The Wittig reaction was applied to the other analogues, and again, the enol ethers were formed in good yield and good selectivity, showing the generality of the process. Finally, silyl deprotection with TBAF furnished PGI_2_–F_2_ (**6**) and the corresponding analogues **7**, **8,** and **9** (9–16% overall yields and 81:19–91:9 selectivities).

**Scheme 6:**
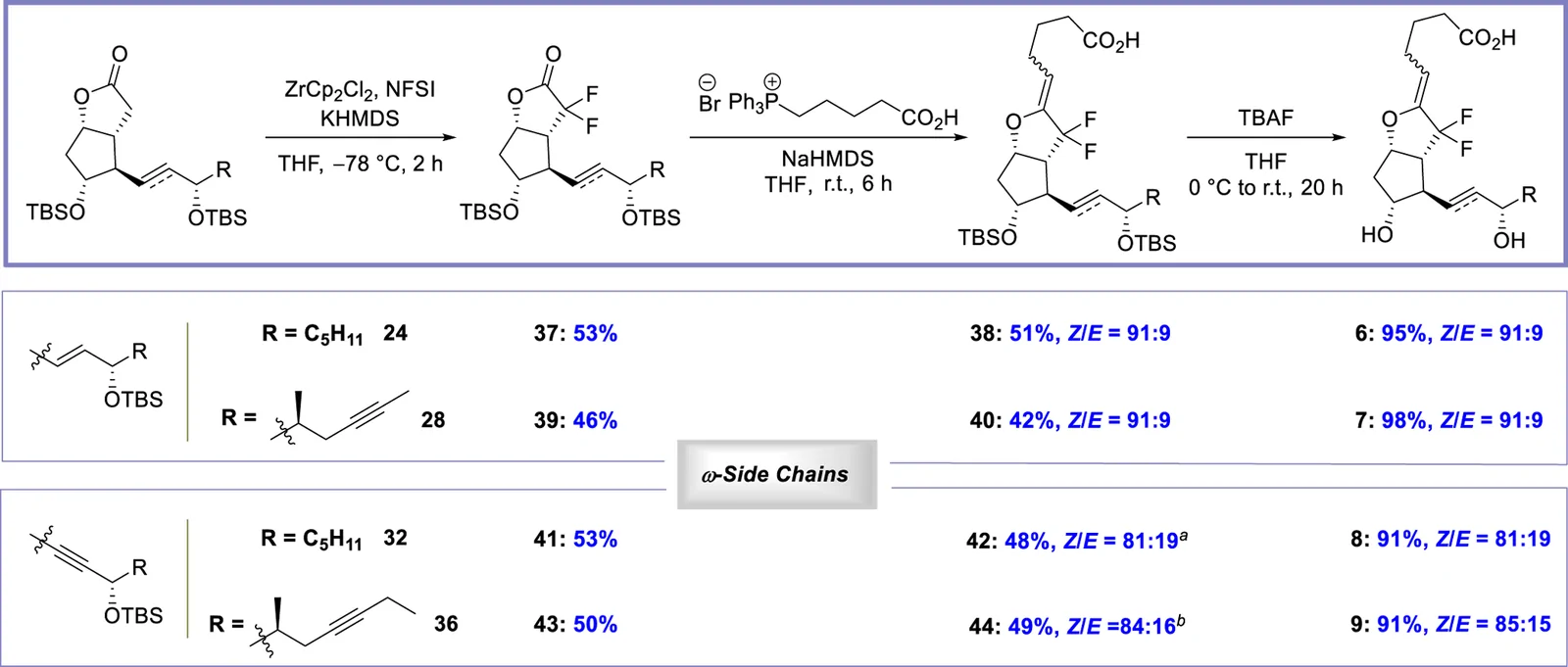
Completion of the Synthesis of Difluorinated Targets **6**, **7**, **8,** and **9** ^a^ ^,^ ^b^

a The bisilyl ether (10%) was isolated together with the monosilyl ether (38%), both of which were converted to the final target **8**.
b The reaction was conducted at rt for 3 h.

We then moved on to the monofluorinated targets. Predicting that the olefination would be more challenging compared to the difluorinated substrate, we explored conditions on model substrate **45** (see Supporting Information for details). In our first attempt, we applied the same Wittig protocol used for the difluorinated substrate **37** but without success. We also explored olefination reactions using Takeda’s Cp_2_Ti­(P­(OEt)_3_)_2_/thioacetal and Takai’s *gem*-dihaloalkane-Zn-TiCl_4_ methods but again without success.^57,58^ Analysis of the literature revealed one example of an olefination (specifically a methylenation) of an α-monofluorinated lactone using a Julia–Kocienski reaction with 2-methanesulfonylbenzothiazole, giving the olefinated product in 78% yield.^59^ To our delight, applying these conditions to our model lactone **45** using the sulfonylbenzothiazole bearing the required α-side chain yielded product **46** in 67% yield (Scheme 7a). Unfortunately, the undesired *E*-isomer was formed preferentially in a 25:75 *Z*/*E*-ratio. A variety of conditions and sulfonyl reagents were explored, but in all cases, either poor selectivity or even higher *E* selectivity was observed (see Table S3 for full details). Notably, the phenylsulfonyltetrazole reagent gave an 11:89 ratio in favor of the *E* isomer. We, therefore, returned to the Wittig reaction and explored alternative conditions, modifying the base and stoichiometry. Ultimately, we found that using LiHMDS instead of NaHMDS as base and a ratio of lactone (**45**): Wittig salt: LiHMDS of 1:1:2 at r.t. was successful, giving the enol ether product in 45% yield and a 76:24 *Z*/*E* ratio (Scheme 7b). By lowering the temperature, the ratio was further increased to 89:11 at −20 °C (see Table S4 for full details).

**Scheme 7:**
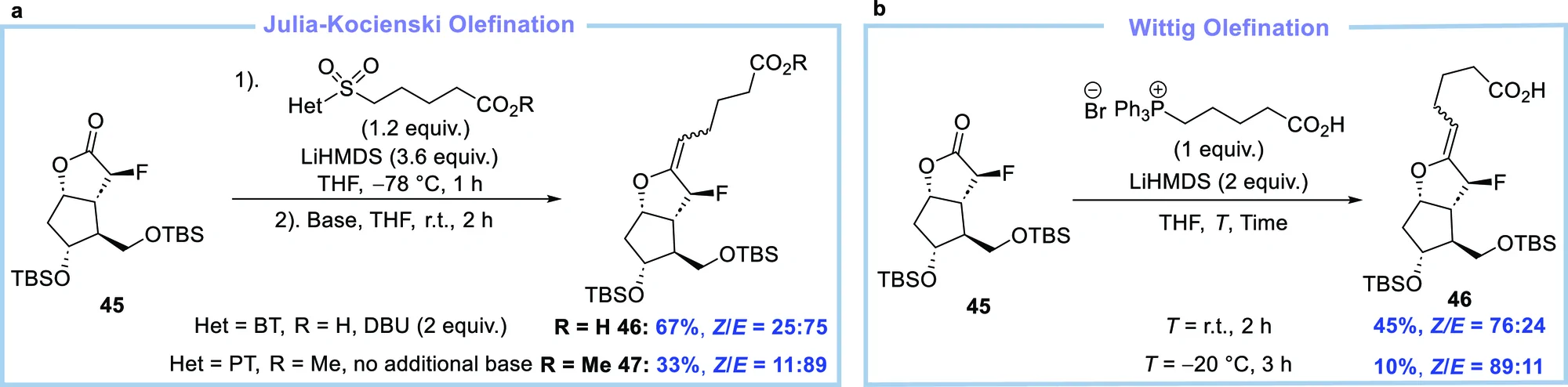
(a) Julia–Kocienski Olefination on α-Monofluorinated Model Lactone **45**; (b) Wittig Olefination on α-Monofluorinated Model Lactone **45** ^a^

a Yield and *Z*/*E* ratio determined by ^19^F NMR using α,α,α-trifluorotoluene as an internal standard. BT = benzothiazole, PT = 1-phenyl-1*H*-tetrazole.

Having established conditions for olefination on the model substrate, we turned to the actual target (Scheme 8). The monofluorinated substrate **48** was synthesized from lactone **24** with NFSI as a single diastereoisomer. The stereochemistry was established as (3*S*) by ^1^H–^19^F HOESY (see Supporting Information for detailed information). In order to obtain high yields in this step, it was necessary to use Barbier-type conditions, where the base was added to a solution of the substrate and NFSI. Brief optimization of the olefination reaction revealed that using 1.5 equiv of the Wittig salt and stirring the reaction at r.t. for 3 h gave the product **49** in 40% yield and an 83:17 *Z*/*E* ratio. Final deprotection with TBAF gave the monofluorinated alkene **2**. The sequence of monofluorination, Wittig reaction, and deprotection was applied to the remaining lactones, giving the targets **3**, **4,** and **5** in reasonable overall yields (7–18%) and selectivities (78:22–89:11).

**Scheme 8:**
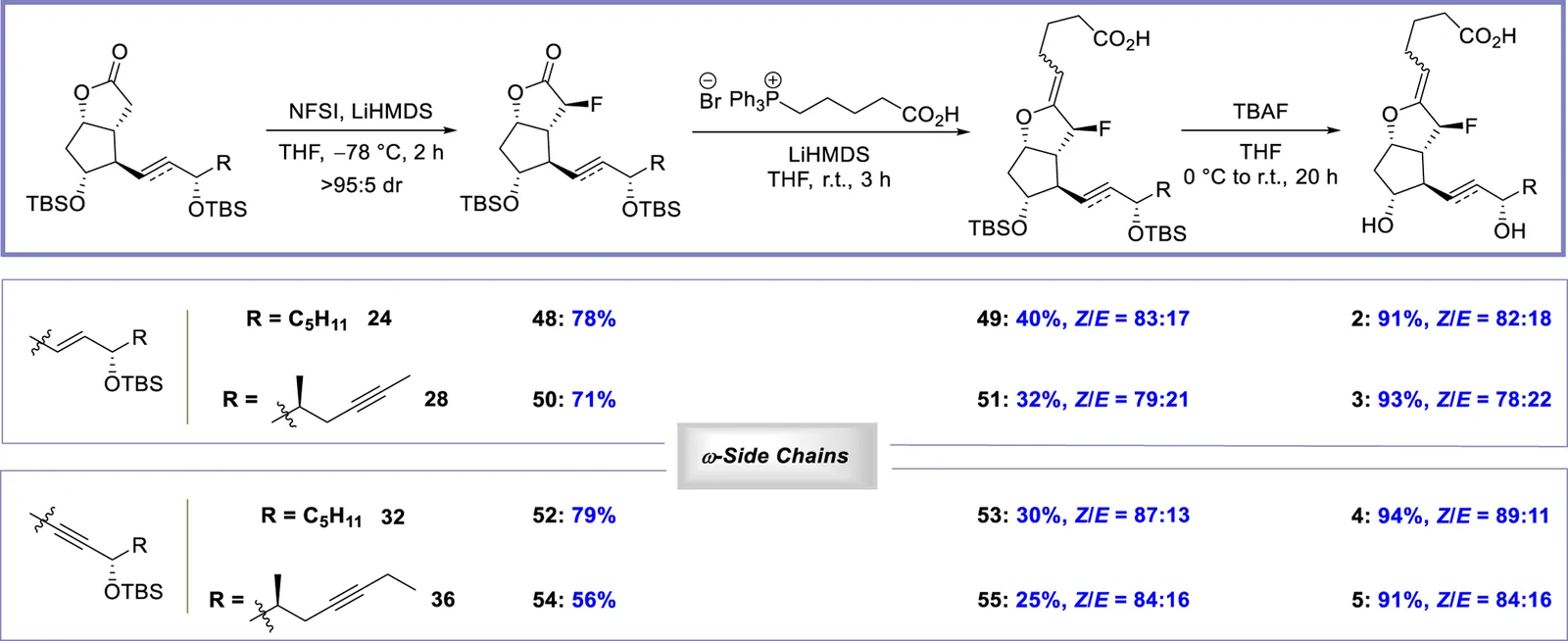
Completion of the Synthesis of Monofluorinated Targets

### 2.4 Bioactivity Results and Analysis

To evaluate the biological activity of the fluorinated PGI_2_ compounds **2**–**9**, concentration-inhibition experiments were performed on human platelets. We measured two biological outcomes: integrin α_IIb_β_3_ activation and surface expression of the α-granule marker P-selectin in response to stimulation of the thrombin receptor protease-activated receptor 1 (PAR1) (Figure 3). These outcomes were measured, as they (i) underlie platelet aggregation and thrombus formation, (ii) are effectively inhibited by IP receptor agonists, and (iii) are amenable to high throughput.^60^ The clinically used compounds, iloprost and cicaprost, were used as reference compounds.

**Figure 3:**
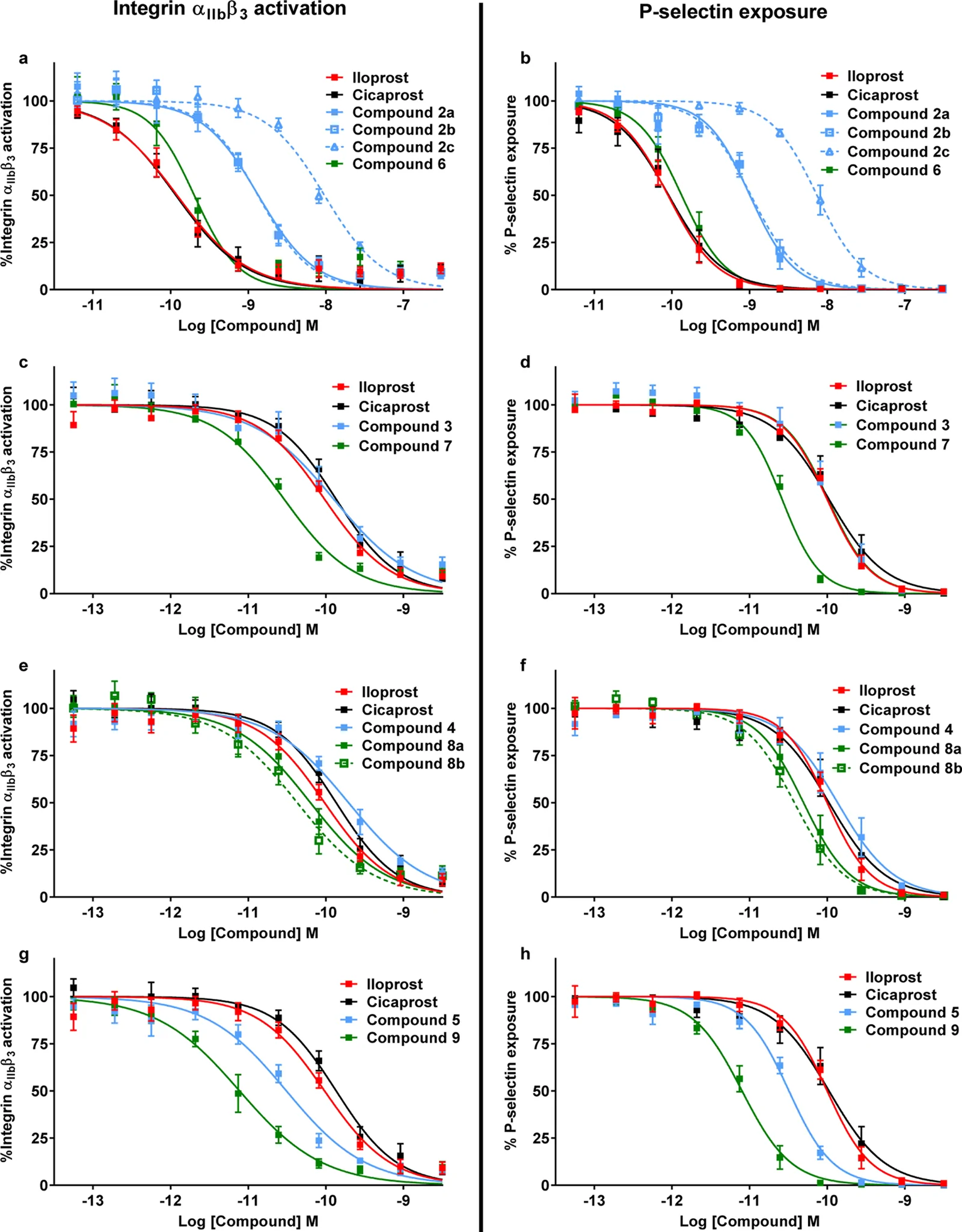
The antiplatelet effects of fluorinated PGI_2_ analogues **2–9**. Concentration–response curves demonstrating the inhibitory effect of the fluorinated PGI_2_ analogues **2–9** and control compounds iloprost and cicaprost on PAR-mediated integrin α_IIb_β_3_ activation (a,c,e,g) and P-selectin exposure (b,d,f,h). Platelets were incubated with compounds (5 min) before stimulation with the PAR-1 agonist SFLLRN (5 μM, 10 min) in the presence of fluorescently conjugated antibodies against the active form of integrin α_IIb_β_3_ (FITC-PAC1) and the platelet α-granule marker, P-selectin (PE-CD62P). Platelets were fixed, and events were captured by FACS analysis using a BD Accuri C6 Plus flow cytometer. Data are expressed as a percentage of maximal (mean ± SEM, *n* = 7).

As shown in Figure 3 and Table 1, greater antiplatelet effects were observed for the difluorinated PGI_2_ analogues over the monofluorinated analogues (e.g., Figure 3a–b, **2** vs **6**, Figure 3c–d, **3** vs **7**), with compound **7** showing enhanced biological activity over the reference compounds (curves left shifted and increased pIC_50_). Replacing the ω-alkene of the natural PGI_2_ linkage to the cyclopentane ring with an alkyne linkage also improved potency of both monofluorinated (**4**) and difluorinated (**8**) PGI_2_ (Figure 3e–f). The greatest enhancement in biological activity was, however, found using the PGI_2_ analogues that had the same ω-side chain as cicaprost, with both the monofluorinated (**5**) and difluorinated (**9**) analogues (Figure 3g–h), showing superior biological activity over the reference compounds (curves substantially left shifted and increased pIC_50_) and the reported activity of previous fluoroprostacyclins (compound **1**, 5-fluoro- and 10,10-difluoroprostacyclin).

**Table 1:** Comparison of the Concentration of Reference Compounds and Fluorinated PGI_2_ Analogues 2–9 That Inhibit Platelet Function by 50% (IC_50_)

		integrin α_IIb_β_3_ activation			P-selectin expression		
compound	*Z*/*E* ratio	pIC_50_ ^a^± SEM, *n* = 7	IC_50_ ^b^ (nM)	IC_50_ ^c^ (nM)	pIC_50_ ^a^± SEM, *n* = 7	IC_50_ ^b^ (nM)	IC_50_ ^c^ (nM)
**Iloprost**	-	10.03 ± 0.06	0.093	-	10.00 ± 0.07	0.099	-
**Cicaprost**	-	9.85 ± 0.05	0.141	-	10.02 ± 0.20	0.094	-
**2a**	82:18	8.89 ± 0.08	1.291	1.059	9.05 ± 0.13	0.889	0.729
**2b**	71:29	8.88 ± 0.06	1.313	0.932	8.99 ± 0.07	1.028	0.730
**2c**	14:86	8.04 ± 0.04	9.193	1.287	8.18 ± 0.09	6.550	0.916
**3**	78:22	10.0 ± 0.16	0.100	0.078	10.0 ± 0.13	0.099	0.077
**4**	89:11	9.73 ± 0.08	0.187	0.166	9.89 ± 0.18	0.127	0.113
**5**	84:16	10.59 ± 0.09	0.025	0.021	10.50 ± 0.05	0.031	0.026
**6**	91:9	9.72 ± 0.07	0.190	0.173	9.88 ± 0.09	0.131	0.119
**7**	91:9	10.55 ± 0.08	0.028	0.025	10.59 ± 0.06	0.025	0.023
**8a**	81:19	10.28 ± 0.13	0.052	0.042	10.32 ± 0.11	0.047	0.038
**8b**	96:4	10.39 ± 0.13	0.040	0.038	10.43 ± 0.12	0.037	0.035
**9**	85:15	11.04 ± 0.04	0.009	0.007	10.95 ± 0.11	0.011	0.009
*Z* **-9**	100:0	11.03 ± 0.18	0.009	-	11.06 ± 0.18	0.008	-
*E* **-9**	0:100	9.29 ± 0.12	0.514	-	9.31 ± 0.12	0.487	-

a The inhibitory effect of the reference compounds and PGI_2_ analogues **2–9** was assessed as described in Figure 3. Mean pIC_50_ values were calculated by averaging pIC_50_ values (*n* = 7 ± SEM) obtained from individual concentration-inhibition curves fitted with a four-parameter logistic equation (GraphPad Prism 7).

b IC_50_ values [nM] were calculated by taking the antilog of the mean of the logIC_50_ values and are the concentration of compound required for 50% inhibition of PAR-mediated integrin α_IIb_β_3_ activation and P-selectin exposure on human platelets.

c The IC_50_ values were recalculated by refitting concentration-inhibition curves based on the assumption that the *Z* isomer is the active form of the prostanoid analogue and the *E* isomer is inactive (no binding affinity or efficacy for platelet prostanoid receptors). See Figures S1 and S2 in Supporting Information for detailed information.

Previous studies by Djurić on the *Z* and *E* isomers of 7-F prostacyclins had shown that the *Z* isomer was significantly more potent than the *E* isomer (approximately 200-fold).^17^ We tested different ratios of *Z* and *E* isomers for compounds **2** and **8,** as indicated in Table 1, and found that those enriched in the *Z* isomer had substantially higher potency (compare pIC_50_ for **2a**, **2b,** and **2c** and **8a** and **8b**), mirroring earlier observations. Of note, a ∼6-fold reduction in the concentration of the *Z* isomer of **2** with a corresponding ∼6-fold increase in the *E* isomer led to a ∼7-fold reduction in potency, indicating that the majority of the biological effect lay with the *Z* isomer. Assuming that the *Z* isomer is the active form and the *E* isomer has limited affinity/efficacy for platelet prostanoid receptors, we refitted the concentration inhibition curves (see Figure S1 for full details) and recalculated the pIC_50_ values (see Table S1 for full details). Interestingly, this resulted in the concentration–inhibition curves and IC_50_ values for the different *Z*/*E* mixtures of compound **2** to converge, confirming that the *Z* isomer is indeed largely responsible for the biological activity.

The PGI_2_ analogues were initially tested as *E*/*Z* mixtures, as we were unable to separate the *E*/*Z* isomers of any of the compounds despite considerable effort using normal and reverse phase high performance liquid chromatography (HPLC) (>100 different columns and conditions). However, to confirm the above findings, further effort was made to find a derivative or intermediate of the most active difluorinated analogue **9** that would enable separation of the *E*/*Z* isomers. Eventually, we found that the *E*/*Z* isomers of the methyl ester of **44** could be separated by normal phase HPLC using a Chiralcel OD-3 column (achiral columns gave no separation, so we had to resort to a chiral column) with hexane/IPA (99:1) as eluent. The isomers were then hydrolyzed and deprotected to give pure *E* **-9** and *Z* **-9,** which were tested separately. As shown in Table 1 and Figure S2 in Supporting Information, *Z* **-9** was >50-fold more active than the *E* isomer, mirroring studies on the monofluorinated prostacyclins by Djurić,^17^ and providing further validation for our approach.

### 2.5 Prostanoid Receptor Specificity

To assess prostanoid receptor selectivity, the four most potent compounds (**5**, **7**, **8b,** and **9**; Table 1) were screened alongside the reference PGI_2_ analogues iloprost and compound **1** (AFP-07) using the PRESTO-Tango β-arrestin recruitment assay in cells transiently transfected with prostanoid receptors. This platform enables parallel profiling across multiple G-protein coupled receptors using a uniform transcriptional readout. Among the tested compounds, the difluorinated compounds with the ω-side chain of iloprost (**7**) and cicaprost (**9**) were the most potent IP receptor agonists (Figure 4), demonstrating a left-shifted concentration–response curve in comparison to both the monofluorinated form (**5**) and compound **1**; compound **9** was ∼333-fold more potent than compound **1**. No IP signal was observed for iloprost, likely due to its chemical instability over the 24 h assay incubation period.

**Figure 4:**
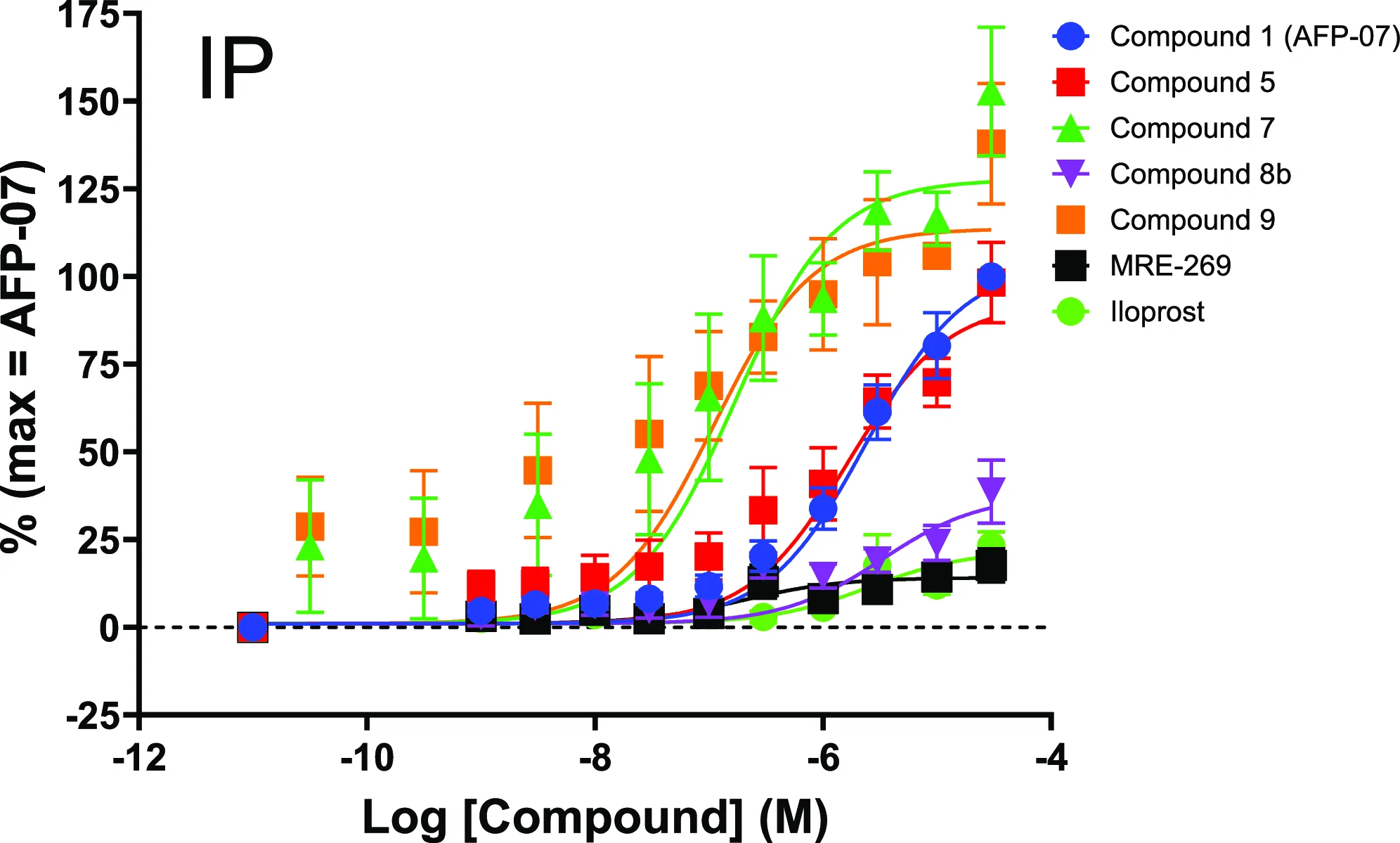
The novel PGI_2_ analogues ligand **7** and ligand **9** both demonstrate high potency for the IP receptor. This was demonstrated *via* concentration-dependent ligand-induced β-arrestin2 recruitment through the IP receptor, which was transiently expressed in the HTLA cell line assessed via the PRESTO-Tango assay utilizing compound **1** (AFP-07) as a control to normalize data. Error bars represent ± SEM (*n* = 4).

The compounds were subsequently evaluated against other prostanoid receptors (DP1, DP2, EP1–4, FP, and TP; Figure S4). At DP1, compounds **7** and **9** showed no detectable activity, while compounds **5**, **8b,** and **1** induced only weak responses compared to the DP1 agonist BW245C (Figure S4c). At DP2, the selective agonist PGD2 produced robust β-arrestin recruitment, whereas none of the PGI_2_ analogues elicited measurable activity (Figure S4d). Within the EP receptor family, compound **1** was the most potent EP1 agonist, followed by compound **7**, while compound **9** was inactive (Figure S4e). For EP2, only the highest concentration of compound **9** (30 μM) induced detectable receptor activation. As EP3 couples to Gαi and can counteract PGI_2_ signaling and contribute to side effects of PGI_2_ analogues, potential off-target activation was carefully examined. Using sulprostone as a reference agonist, none of the PGI_2_ analogues activated EP3 at concentrations required for IP receptor stimulation (Figure S4g). Partial EP3 activation was observed only at the highest concentration of compounds **7** and **8b**, whereas compounds **9**, **5,** and **1** showed no significant effect. In contrast, compound **9** displayed substantial activity at EP4 receptors, with the difluorinated compounds **7** and **8b** showing comparably lower potency and compound **5** exhibiting weaker responses. This suggests that the additional fluorine in PGI_2_ with the ω-side chain of cicaprost (**9**) provides a significantly enhanced EP4 selectivity compared to the monofluorinated form (**5**). The 10,000-fold lower potency of the difluorinated compound **1** at the EP4 receptor compared to compound **9** indicates that the cicaprost ω-side chain of compound **9** also makes a significant contribution to the enhanced EP4 receptor potency. As both IP and EP4 receptors couple to Gαs, this dual activity profile suggests the potential for additive signaling effects. Dual targeting of the prostacyclin IP receptor and the EP4 receptor may provide complementary therapeutic benefits in both platelets and pulmonary arteries. As both the IP and EP4 receptors are predominantly Gαs-coupled, their activation increases cAMP, leading to inhibition of platelet aggregation and relaxation of pulmonary vascular smooth muscle. Therefore, a PGI_2_ analogue with additional EP4 activity may enhance antithrombotic and pulmonary vasodilatory effects compared with selective IP receptor activation alone. No measurable activity was detected for any of the compounds at FP or TP receptors (Figure S4a,b). Overall selectivity profiling (Table S2) identified compound **9** as the most potent and selective PGI_2_ analogue, combining strong IP receptor activation with significant EP4 activity and minimal off-target effects.

### 2.6 Stability Studies

The chemical stability of the most potent prostacyclin analogue **9** was tested in pH 7.4 phosphate buffer by ^19^F NMR using an internal standard (Scheme 9). After 30 days at 23 °C, >97% of **9** remained. We also examined its stability in human plasma by LC–MS/MS using indomethacin as an internal standard.^61,62^ After 7 days at 37 °C, >97% of compound **9** remained. Once again, this showed that the prostacyclin analogue **9** was completely stable under these conditions. Compared to the chemical half-life of PGI_2_ (∼10 min), this demonstrates the remarkable stabilizing effect of the difluoro group.

**Scheme 9:**
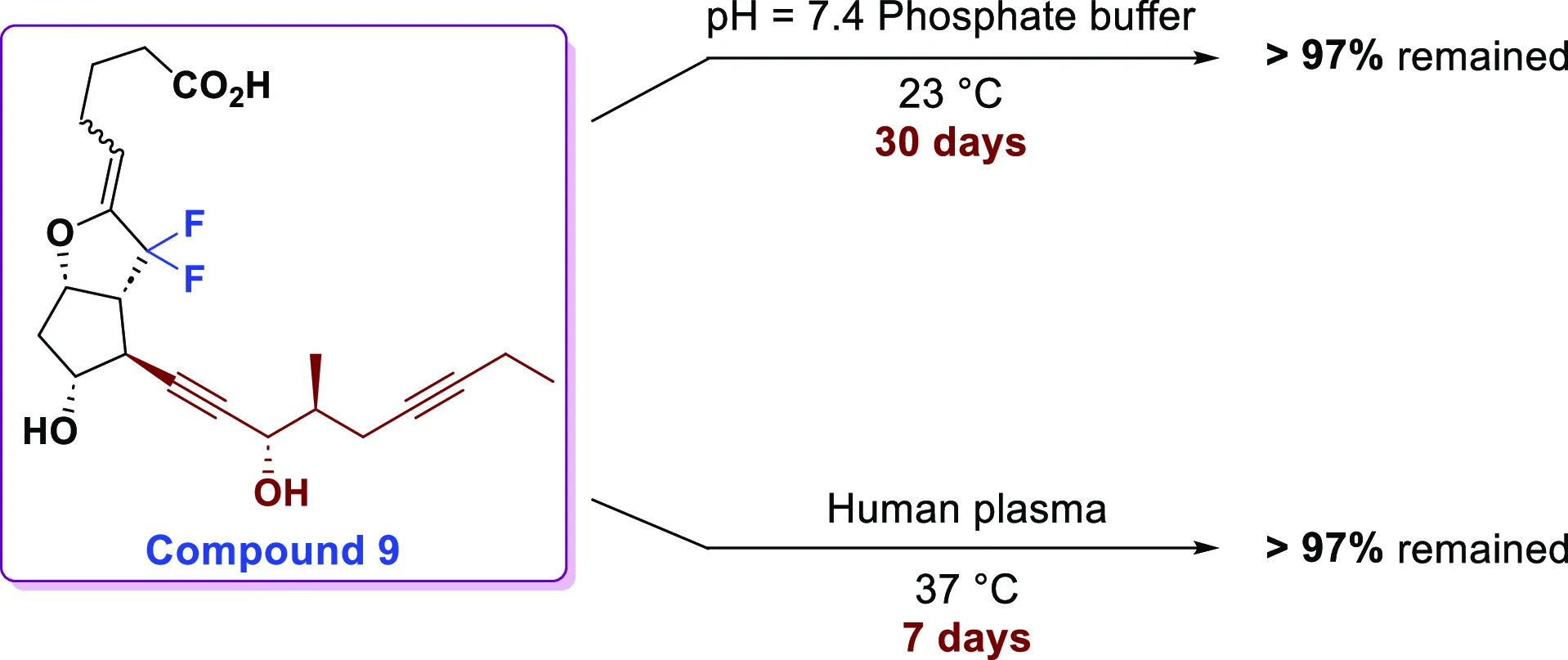
Chemical and Human Plasma Stability for Compound **9**

To investigate the metabolic stability of compound **9**, Sprague–Dawley rats were dosed by intravenous administration (0.05 mg/kg dosage; see Supporting Information for details). Using LC–MS/MS analysis, the exposure levels of compound **9** in rat plasma were analyzed over 24 h, and the metabolic half-life was determined to be 24 min. Therefore, our lead compound is more metabolically stable than PGI_2_ (3–6 min)^12^ and comparably stable to iloprost (20–30 min).^21^ Although compound **9** is not as metabolically stable as cicaprost (1–2 h),^22^ it is 15-fold more potent.

### 2.7 Molecular Modeling

To shed light on the enhanced potency of prostacyclin analogues **5**, **7**, and **9**, molecular modeling studies were conducted using the IP receptor crystal structure cocrystallized with MRE-269 (PDB: 8X79).^63^ PGI_2_ and analogues **5**, **7**, and **9** were docked using the GNINA 1.0 deep learning augmented docking method.^64^ Subsequent molecular dynamics (MD) simulations were performed, and binding free energies were estimated using the resulting trajectories (see Supporting Information for details). Docking and MD simulations revealed that all four compounds adopted stable binding poses, with the core interactions of PGI_2_ conserved across analogues **5**, **7**, and **9**. A key salt bridge between Arg279 and the prostacyclin carboxylate was maintained in all cases, along with consistent contacts involving residues Ser168, Val71, Ser20, Met23, and Tyr281 (Figure 5a).

**Figure 5:**
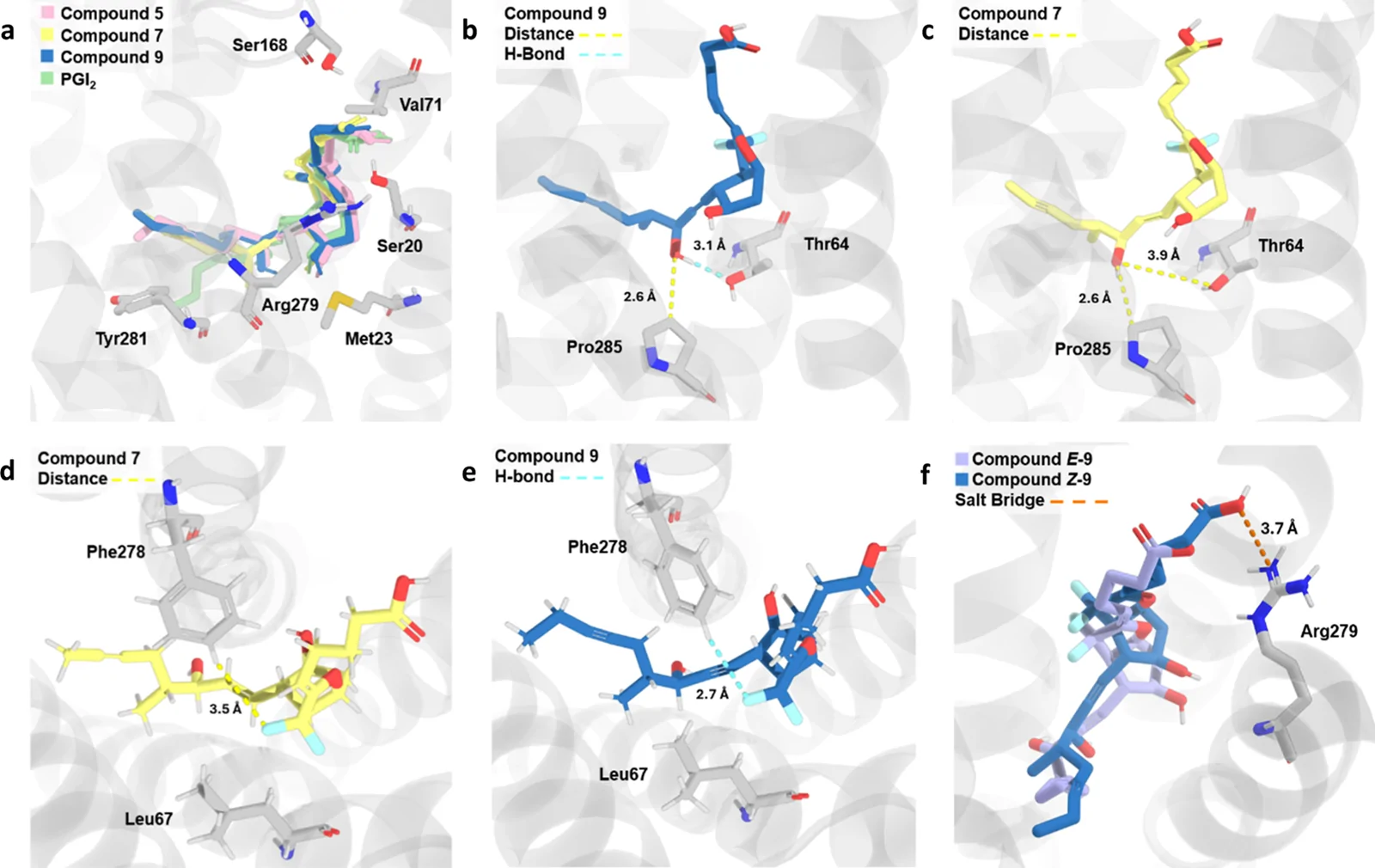
Molecular modeling studies of PGI_2_ and analogues **5**, **7**, and **9**. For the molecular docking, the structures of MRE-269 and treprostinil bound to IP receptors were used (PDBs 8X79 and 8X7A, respectively). (a) Overlaid view of compounds **5** (pink), **7** (yellow), **9** (blue), and PGI_2_ (green) docked in the IP receptor (gray); (b) view of compound **9** with average distances from the propargyl alcohol to amino acids Thr64 (H bond, cyan dashed line) and Pro285 (yellow dashed line); (c) view of compound **7** with average distances from the allylic alcohol to amino acids Thr64 and Pro285 (yellow dashed lines); (d) view of compound **7** and neighboring lipophilic residues Leu67 and Phe278 with average distance to Phe278 (yellow dashed line); (e) view of compound **9** and neighboring lipophilic residues Leu67 and Phe278 with average distance to Phe278 (H bond, cyan dashed line); (f) overlaid view of compounds *E* **-9** and *Z* **-9**, only the *Z*-isomer forms a stable salt bridge (orange dashed line) to the guanidinium side chain of Arg279.

Furthermore, all poses were consistent with reported mutagenesis studies and binding data.^65–67^ Analogues **7** and **9** differ only at the ω-side chain (alkyne, **9**, vs *E-*alkene, **7**), yet analogue **9** is markedly more potent. Modeling reveals that the propargylic alcohol forms a hydrogen bond (∼3.1 Å, Figure 5b) to the Thr64 side chain hydroxyl, whereas the allylic alcohol does not (∼3.9 Å, Figure 5c). Furthermore, from MD simulations, alkyne **9** forms a tighter complex with the protein compared to alkene **7,** which forms a looser complex with much greater fluctuation in distances over time (see Supporting Information Figure S9), reflecting its superior binding. As an illustration, both analogues **7** and **9** are within van der Waals distance of Pro285. While compound **9** maintains a near-constant distance from Pro285 throughout the simulation, compound **7** shows greater variability (see Figure S10). This structural instability has a direct impact on the energetic contributions. Our energy decomposition analysis using MM/PBSA reveals that Pro285 contributes −1.8 kcal/mol more favorably to the binding of compound **9** compared to compound **7**, a substantial improvement to the overall affinity. Our energy decomposition analysis suggests that these interactions collectively contribute −3.5 kcal/mol more favorable binding energy for analogue **9** relative to analogue **7**, which is consistent with the observed SAR.

Fluorine substituents at the 7-position were originally introduced to prevent rapid hydrolysis of the highly labile enol ether present in PGI_2_ (*t*_1/2_: PGI_2_ = 31 s, **7** & **9** > 1 year). However, our modeling shows that the fluorine atoms are also engaged in favorable van der Waals interactions with hydrophobic residues of the protein. In particular, the lipophilic residue Leu67 resides close to the fluorine atoms of analogues **5**, **7**, and **9**. Compared to PGI_2_, this interaction contributes −3.3 kcal/mol more binding free energy for monofluorinated **5**, and −4.6 kcal/mol for difluorinated analogues **7** and **9** (Figure 5d vs 5e; note, the distances between the β-carbon of leucine and the ligand are similar but the binding energy for the fluorine-containing substrates is greater). Furthermore, the binding pose of analogue **9** uniquely positions one fluorine atom to form a weak hydrogen bond with the side chain of residue Phe278 (2.7 Å, ∼180° angle),^68–70^ contributing an additional −2.4 kcal/mol of binding energy. This interaction is not observed for analogue **7** due to differences in the modeled pose. Hence, compared to other analogues, analogue **9** benefits from enhanced interactions of both fluorine atoms to the protein as well as hydrogen bonding of the propargylic alcohol to Thr64, accounting for its superior binding affinity and the observed SAR. Finally, we investigated the stark difference in affinity for the *Z*- and *E*-isomers of **9** (Figure 5f). Our MD simulation shows the carboxylate of *E* **-9** is poorly orientated and cannot form a stable salt bridge with Arg279, whereas the *Z*-isomer maintains this critical interaction throughout the simulation. This difference could account for the >50-fold increase in affinity observed for *Z* **-9** over *E* **-9**.

## 3 Conclusion

We have developed short (7–8 steps) syntheses of fluorinated prostacyclin analogues, utilizing our enal intermediate, which is available in just 2 steps from 2,5-dimethoxyTHF. Other key features include difluorination of a lactone and Wittig olefination of both mono- and difluorinated lactones with good levels of *Z* selectivity. The brevity of the synthesis facilitated analogues being easily made and tested for their biological activity. In particular, the 1,4-addition of alkynes to the enal intermediate enabled the ready access to the most potent analogue.

Key findings from the biological data (Figure 6) indicate that (i) in all cases the ω-alkyne linkages to the cyclopentane ring showed higher activity than the alkene linkages (compare **5** and **3**, **9** and **7**, **4** and **2**, and **8** and **6**). Interestingly, cicaprost, which also has a similar ω-alkyne linkage, shows higher activity than the related ω-alkene linkage present in iloprost (albeit with different α-side chains); (ii) Although we had expected the monofluoro derivatives to be better mimics of prostacyclin than the difluoro analogues, the biological activity of the difluoro compounds was 4–10× more potent. The *anti* diastereoisomer of the ω-side chain was known to be the optimal stereochemistry; likewise, the *Z*-vinyl ether was >50-fold more active than the *E*-isomer. Four of the eight compounds tested (**5**, **7**, **8**, and **9**) had superior activity to iloprost, demonstrating that marrying the ω-side chains of iloprost and cicaprost with the stable fluorinated enol ether of prostacyclin was an effective strategy for creating hybrids with improved biological activity. In fact, compound **9** was an order of magnitude more potent than iloprost and cicaprost and thus provides the highest potency reported to date. From modeling studies, it was found that many of the functional groups present in **9** (CO_2_H, OH) as well as the fluorine atoms are involved in binding to the PGI_2_ receptor, providing a tentative model for the high potency of compound **9**. In addition, compound **9** also showed exceptional stability in pH 7.4 buffer and blood plasma and metabolic stability comparable to iloprost. Selectivity profiling of different compounds to the various prostanoid receptors identified compound **9** as the most potent and selective PGI_2_ analogue, combining strong IP receptor activation with significant EP4 activity and minimal off-target effects.

**Figure 6:**
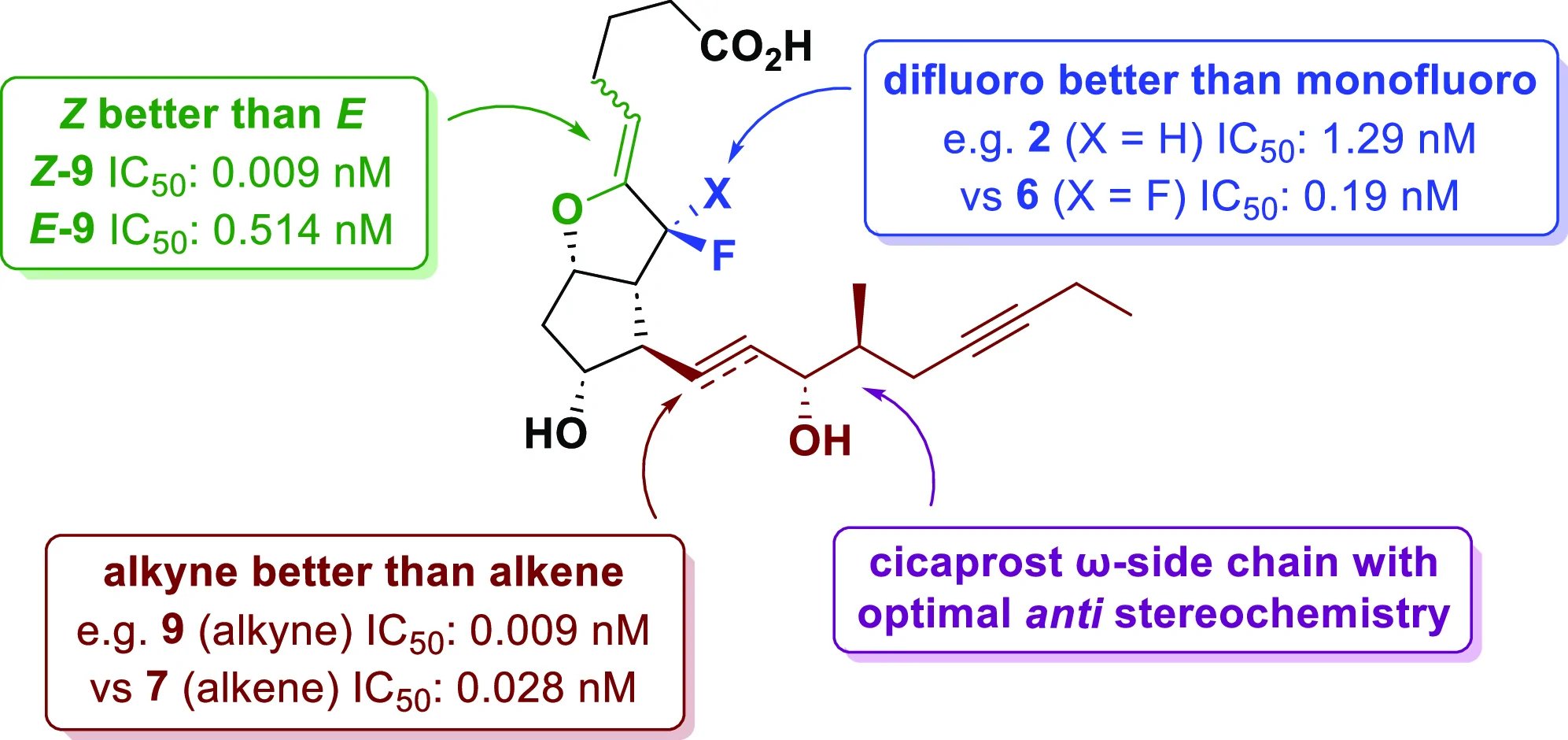
Identification of key functionalities that contribute to the improved SAR of compound **9**.

## 4 Experimental Section

### 4.1 General Information

#### 4.1.1 Solvents and Reagents

Generally, reactions were carried out using reagent-grade solvents and without air exclusion. Reactions requiring anhydrous solvents and an inert atmosphere were carried out under a nitrogen atmosphere using standard manifold techniques. All solvents were commercially supplied or obtained from a purification column composed of activated alumina.^71^ All chemicals were purchased from Acros, Aldrich, Alfa Aesar, Fluka, Lancaster, or Merck and used as received unless otherwise stated. Propanal was distilled prior to use. 2,6-Lutidine was treated with anhydrous AlCl_3_ and distilled under reduced pressure prior to use. Triethylamine and trimethylsilyl chloride were distilled over CaH_2_ under reduced pressure prior to use. *n*-BuLi and *tert*-BuLi were purchased from Acros and were titrated against *N*-benzylbenzamide using the procedure of Chong et al.^72^ LiHMDS solution was prepared freshly from distilled HMDS and *n*-BuLi. O_3_–*O*_2_(*g*) was generated by Ozone Generator C-Lasky from AirTree Ozone Technology Co., Ltd. *Chromatography*. Flash column chromatography (FCC) was carried out using Sigma-Aldrich silica gel (60 Å, 230–400 mesh, 40–63 μm) or using Biotage Isolera One with Biotage SNAP/SNAP Ultra Cartridges. All reactions were followed by thin-layer chromatography (TLC) when practical, using Merck Kieselgel 60 F254 fluorescent-treated silica gel, which was visualized under UV light or by staining with aqueous basic potassium permanganate or an ethanolic and acidic solution of *p*-anisaldehyde. *Purity of compounds.* All compounds subjected to *in vitro* and *ex vivo* assays are >95% pure, determined by HPLC performed on a reverse phase HPLC Agilent 1260 Infinity II HPLC system, using a Waters Spherisorb S5 ODS2 column (5 μm particle size, 4.6 mm internal diameter × 250 mm length) with detection carried out at 214 nm. H2O/MeCN (with 0.1% formic acid) mixtures were used as eluents for all analyses. Analysis was performed at room temperature using the following method: 1 mL/min flow rate, 20 min run, MeCN/H2O (0.1% formic acid) gradient [time]: 30:70 [0 min], 30:70 [1 min], 95:5 [16 min], 95:5 [17 min], 30:70 [18 min]. *Spectroscopy*. ^1^H, ^13^C NMR, and ^19^F NMR spectra were recorded on Jeol ECS 400, Bruker Nano 400, Varian VNMRS500, and Bruker Avance III HD 500 Cryo spectrometers. Chemical shifts (δ values) are referenced relative to the residual protonated solvent and are reported in parts per million (ppm), and coupling constants (*J* values) are given in Hertz (Hz). The ^1^H NMR spectra are reported as follows: chemical shift, multiplicity (s = singlet, br s = broad singlet, d = doublet, t = triplet, q = quartet, quin = quintet, sex = sextet, h = heptet, m = multiplet, dd = doublet of doublets, etc.), coupling constants, and assignment. NMR assignments are made according to spin systems, using two-dimensional NMR spectroscopy (COSY, HSQC, and HMBC) to assist the assignment. High resolution mass spectra (HRMS) were recorded on a VG Analytical Autospec spectrometer by electron ionization (EI) or chemical ionization (CI) or on a Bruker micrOTOF instrument by electrospray ionization (ESI). Infrared (IR) spectra were recorded on a PerkinElmer Spectrum One FT-IR spectrometer as a thin film. Only the selected absorption maxima (ν_max_) are reported in wavenumbers (cm^–1^). Melting points were recorded in degrees Celsius (°C), using a Kofler hot-stage microscope apparatus and are reported uncorrected. Optical rotation ([α]_D_^T^) was measured on a Bellingham and Stanley Ltd. ADP220 polarimeter and is quoted in (° mL) (g dm)^−1^. Chiral supercritical fluid chromatography was performed using a Daicel Chiralpak IA column (4.6 × 250 mm × 5 μm) using a Waters TharSFC system and monitored by a diode array detector (DAD). Chiral HPLC was performed on an HP Agilent 1100 with a Daicel Chiralpak OD-H column (4.6 × 250 mm × 5 μm) fitted with the respective guard (4 × 10 mm) and monitored by DAD. *Naming of Compounds*. Compound names are generated by ChemBioDraw 11.0 software (PerkinElmer), following IUPAC nomenclature. Detailed procedures for the synthesis of all intermediates, including ω-side chains, can be found in the Supporting Information.

### 4.2 Synthesis of Key Bicyclic Enal **12**

Lactol **10** was prepared from freshly distilled succinaldehyde according to the literature, and analytical data were consistent with data reported in the literature.^73^

#### 4.2.1 (3a*R*,6a*S*)-2-Oxo-3,3a,6,6a-tetrahydro-2*H*-cyclopenta­[*b*]­furan-5-carbaldehyde (**12**)

To a solution of lactol **10** (13.0 g, 84.3 mmol, 1.0 equiv) in acetonitrile (420 mL) was added tetrakisacetonitrile copper­(I) triflate (1.59 g, 4.22 mmol, 5 mol %), 2,2′-bipyridine (658 mg, 4.22 mmol, 5 mol %), TEMPO (658 mg, 4.22 mmol, 5 mol %), and *N*-methylimidazole (671 μL, 8.44 mmol, 10 mol %). The reaction mixture was stirred at r.t. overnight and then filtered through a silica plug and washed with EtOAc. The filtrate was concentrated, and the crude mixture was purified by FCC on silica gel (Hexanes/EtOAc = 50:50 to 30:70) to give the lactone **12** as a white solid (11.67 g, 76.71 mmol, 91%, 99:1 er). Analytical data were consistent with data reported in the literature.^74^

### 4.3 Synthesis of ω-Side Chain **18**

#### 4.3.1 (2*S*,3*S*)-2-Methyl-5-(triethylsilyl)­pent-4-yne-1,3-diol (**14**)

(*R*)-2-[Bis­(3,5-bis-trifluoromethyl-phenyl)­hydroxymethyl]­pyrrolidine (2.14 g, 4.07 mmol, 10 mol %), H_2_O (2.20 mL, 122 mmol, 3.0 equiv), and propionaldehyde (7.17 mL, 5.92 g, 102 mmol, 2.5 equiv) were sequentially added to a stirring solution of 3-(triethylsilyl)­propiolaldehyde (6.86 g, 40.7 mmol, 1.0 equiv) in 1,4-dioxane (41 mL) at r.t. The reaction mixture was stirred at r.t. for 24 h, and then NaBH_4_ (4.16 g, 110 mmol, 2.7 equiv) was added to the above reaction mixture at 0 °C. After stirring at r.t. for another 1 h, the reaction mixture was quenched by addition of buffer (pH = 7.0) and extracted with EtOAc (3 × 80 mL). The organic phases were combined, washed with brine, dried over MgSO_4_, filtered, and concentrated in *vacuo*. The crude products were purified by FCC on silica gel (Hexanes/EtOAc = 90:10 to 75:25) to give a mixture of two isomers **14** as a pale yellow oil (6.94 g, 30.4 mmol, 75%, *anti*/*syn* = 8.6:1). The mixture of two isomers was further purified by FCC on silica gel (Hexanes/EtOAc = 95:5 to 70:30) to give pure major isomer as a colorless oil (6.20 g, 27.2 mmol). ^**1**^ **H NMR** (400 MHz, CDCl_3_): δ_H_ = 4.37 (d, *J* = 7.0 Hz, 1H), 3.78 (dd, *J* = 11.0, 3.9 Hz, 1H), 3.62 (dd, *J* = 11.0, 7.1 Hz, 1H), 2.92 (overlapping, br s + br s, 2H), 2.01–1.91 (m, 1H), 1.01–0.95 (m, 12H), 0.59 (q, *J* = 7.9 Hz, 6H). ^**13**^ **C NMR** (100 MHz, CDCl_3_): δ_C_ = 106.8, 88.0, 67.3, 66.4, 41.5, 13.1, 7.5, 4.4. **HRMS** (ESI^+^) calcd for C_12_H_24_NaO_2_Si [M + Na^+^] 251.1438; found, 251.1446.

#### 4.3.2 (2*S*,3*S*)-3-(*tert*-Butyldimethylsilyloxy)-2-methyl-5-(triethylsilyl)­pent-4-ynyl Trifluoromethanesulfonate (**15**)

A solution of Tf_2_O (8.00 mL, 13.5 g, 47.7 mmol, 1.1 equiv) in dry CH_2_Cl_2_ (100 mL) and a solution of 2,6-lutidine (25.3 mL, 23.3 g, 217 mmol, 5.0 equiv) in dry CH_2_Cl_2_ (100 mL) were added to a solution of **14** (9.92 g, 43.4 mmol, 1.0 equiv) in dry CH_2_Cl_2_ (220 mL) separately within 20 min at −78 °C. The resulting reaction mixture was stirred at −78 °C for another 40 min. Then, TBSOTf (19.9 mL, 22.9 g, 86.8 mmol, 2.0 equiv) was added to the above reaction mixture dropwise over 10 min at −78 °C. After stirring at 0 °C for another 1 h, the reaction was quenched by addition of saturated NH_4_Cl and extracted with Et_2_O (2 × 400 mL). The organic phases were combined, washed with brine, dried over MgSO_4_, filtered, and concentrated in *vacuo*. The crude product was purified by FCC on silica gel (Hexanes/EtOAc = 100:0 to 96:4) to give pure **15** as a pale pink oil (15.7 g, 33.0 mmol, 76%). ^**1**^ **H NMR** (400 MHz, CDCl_3_): δ_H_ = 4.68 (dd, *J* = 9.6, 5.0 Hz, 1H), 4.53 (dd, *J* = 9.6, 6.4 Hz, 1H), 4.35 (d, *J* = 6.1 Hz, 1H), 2.25–2.15 (m, 1H), 1.13 (d, *J* = 6.9 Hz, 3H), 0.99 (t, *J* = 7.9 Hz, 9H), 0.90 (s, 9H), 0.60 (q, *J* = 7.9 Hz, 6H), 0.16 (s, 3H), 0.12 (s, 3H). ^**13**^ **C NMR** (100 MHz, CDCl_3_): δ_C_ = 118.8 (d, ^1^ *J*_CF_ = 319.6 Hz), 105.6, 88.8, 78.8, 64.4, 40.7, 25.8, 18.3, 12.7, 7.5, 4.4, −4.4, −5.2. **HRMS** (ESI^+^) calcd for C_19_H_37_F_3_NaO_4_SSi_2_ [M + Na^+^] 497.1795; found, 497.1796.

#### 4.3.3 *tert*-Butyldimethyl­((3*S*,4*S*)-4-methyl-1-(triethylsilyl)­octa-1,6-diyn-3-yloxy)­silane (**16**)

Propyne (11.6 mL, 6.14 g, 153 mmol, 5.0 equiv) was condensed in a flame-dried Schlenk flask at −78 °C and diluted with dry THF (185 mL). To this stirring solution was added dropwise *n*-BuLi (1.6 M in hexanes, 99.6 mL, 159 mmol, 5.2 equiv), and the reaction mixture was stirred at −78 °C for 1.5 h. The reaction mixture was warmed to −20 °C, and a solution of triflate **15** (14.6 g, 30.7 mmol, 1.0 equiv) in THF (247 mL) was added dropwise. Then, the reaction mixture was warmed to r.t. and stirred at r.t. for 12 h. The reaction mixture was carefully quenched by addition of water (100 mL) and extracted with Et_2_O (2 × 300 mL). The organic phases were combined, washed with brine, dried over MgSO_4_, filtered, and concentrated in *vacuo*. The crude product was purified by FCC on silica gel (hexane) to give pure **16** as a colorless oil (8.04 g, 22.1 mmol, 72%). ^**1**^ **H NMR** (400 MHz, CDCl_3_): δ_H_ = 4.28 (d, *J* = 6.7 Hz, 1H), 2.31–2.24 (m, 1H), 2.21–2.13 (m, 1H), 1.87–1.81 (m, 1H), 1.78 (t, *J* = 2.6 Hz, 3H), 1.06 (d, *J* = 6.8 Hz, 3H), 0.98 (t, *J* = 7.9 Hz, 9H), 0.90 (s, 9H), 0.58 (q, *J* = 7.9 Hz, 6H), 0.14 (s, 3H), 0.11 (s, 3H). ^**13**^ **C NMR** (100 MHz, CDCl_3_): δ_C_ = 107.6, 87.1, 77.4, 76.8, 66.9, 40.2, 25.9, 22.0, 18.4, 15.3, 7.6, 4.5, 3.6, −4.4, −5.0. **HRMS** (ESI^+^) calcd for C_21_H_40_NaOSi_2_ [M + Na^+^] 387.2510; found, 387.2508.

#### 4.3.4 *tert*-Butyldimethyl­((3*S*,4*S*)-4-methylocta-1,6-diyn-3-yloxy)­silane (**17**)

To a solution of **16** (4.15 g, 11.4 mmol, 1.0 equiv) in wet THF/MeOH (228 mL, 1:1 *v*/*v*) was added K_2_CO_3_ (15.7 g, 114 mmol, 10.0 equiv) at r.t. After stirring at 60 °C for 24 h, the reaction solvents were removed under reduced pressure. The reaction mixture was added to water (50 mL) and extracted with EtOAc (3 × 50 mL). The organic phases were combined, washed with brine, dried over MgSO_4_, filtered, and concentrated in *vacuo*. The crude product was purified by FCC on silica gel (Hexanes/EtOAc = 100:0 to 99:1) to give pure **17** as a colorless oil (2.35 g, 9.38 mmol, 82%). ^**1**^ **H NMR** (400 MHz, CDCl_3_): δ_H_ = 4.32 (dd, *J* = 6.4, 2.1 Hz, 1H), 2.37 (d, *J* = 2.1 Hz, 1H), 2.30–2.22 (m, 1H), 2.21–2.14 (m, 1H), 1.89–1.83 (m, 1H), 1.77 (t, *J* = 2.6 Hz, 3H), 1.06 (d, *J* = 6.8 Hz, 3H), 0.90 (s, 9H), 0.14 (s, 3H), 0.11 (s, 3H). ^**13**^ **C NMR** (100 MHz, CDCl_3_): δ_C_ = 84.2, 77.3, 77.1, 73.3, 66.2, 40.1, 25.9, 22.0, 18.3, 15.1, 3.6, −4.5, −5.1. **HRMS** (ESI^+^) calcd for C_15_H_27_OSi [M + H^+^] 251.1826; found, 251.1820.

#### 4.3.5 *tert*-Butyl­((3*S*,4*S*,*E*)-1-iodo-4-methyloct-1-en-6-yn-3-yloxy)­dimethylsilane (**18**)

The product is light-sensitive. Therefore, the reaction flask was always covered in aluminum foil and was handled in the fume hood when the light was switched off. DIBAL-H (1.0 M in hexanes, 2.20 mL, 2.16 mmol, 1.2 equiv) was added dropwise to a stirring suspension of ZrCp_2_Cl_2_ (684 mg, 2.34 mmol, 1.3 equiv) in dry THF (3.6 mL) at 0 °C, and the resulting suspension was stirred for 1 h at 0 °C before a solution of alkyne **17** (451 mg, 1.80 mmol, 1.0 equiv) in dry THF (1.3 mL) was added dropwise. The mixture was stirred for 5 h at r.t., during which time it became a yellow solution before it was cooled to −78 °C. Then a solution of iodine (548 mg, 2.16 mmol, 1.2 equiv) in THF (1.3 mL) was added, and the mixture was stirred overnight (12 h) while warming from −78 °C to r.t. The reaction was quenched by addition of H_2_O and stirred for 15 min before it was poured over saturated Na_2_S_2_O_3_ and NaHCO_3_ solution (2:1, 20 mL) and extracted with EtOAc (3 × 20 mL). The organic phases were combined, washed with brine, dried over MgSO_4_, filtered, and concentrated in *vacuo*. The crude product was purified by FCC on silica gel (Hexanes) to give pure **18** as a colorless oil (440 mg, 1.16 mmol, 65%). ^**1**^ **H NMR** (400 MHz, C_6_D_6_): δ_H_ = 6.41 (dd, *J* = 14.5, 7.1 Hz, 1H), 6.02 (dd, *J* = 14.5, 0.8 Hz, 1H), 3.85 (t, *J* = 6.7 Hz, 1H), 2.17–2.09 (m, 2H), 1.64–1.59 (m, 1H), 1.57 (t, *J* = 2.6 Hz, 3H), 0.91 (s, 9H), 0.86 (d, *J* = 6.9 Hz, 3H), 0.02 (s, 3H), −0.01 (s, 3H). ^**13**^ **C NMR** (100 MHz, C_6_D_6_): δ_C_ = 147.7, 78.5, 77.6, 77.3, 77.1, 39.3, 26.2, 22.3, 18.4, 15.3, 3.4, −4.2, −4.9. **HRMS** (ESI^+^) calcd for C_15_H_27_INaOSi [M + Na^+^] 401.0768; found, 401.0777.

### 4.4 Synthesis of ω-Side Chain **20**

#### 4.4.1 *tert*-Butyldimethyl­((3*S*,4*S*)-4-methyl-1-(triethylsilyl)­nona-1,6-diyn-3-yloxy)­silane (**19**)

But-1-yne (9.53 mL, 6.44 g, 119 mmol, 5.0 equiv) was condensed in a flame-dried Schlenk flask at −78 °C and diluted with dry THF (144 mL). To this stirring solution was added dropwise *n*-BuLi (1.6 M in hexanes, 77.4 mL, 124 mmol, 5.2 equiv), and the reaction mixture was stirred at −78 °C for 1.5 h. The reaction mixture was warmed to −20 °C, and a solution of triflate **15** (11.3 g, 23.8 mmol, 1.0 equiv) in THF (192 mL) was added dropwise. Then the reaction mixture was warmed to r.t. and stirred at r.t. for 10 h. The reaction mixture was carefully quenched by addition of water (80 mL) and extracted with Et_2_O (2 × 340 mL). The organic phases were combined, washed with brine, dried over MgSO_4_, filtered, and concentrated in *vacuo*. The crude product was purified by FCC on silica gel (Hexanes/EtOAc = 100:0 to 98:2) to give pure **19** as a colorless oil (7.85 g, 22.7 mmol, 87%). ^**1**^ **H NMR** (400 MHz, CDCl_3_): δ_H_ = 4.29 (d, *J* = 6.8 Hz, 1H), 2.32–2.21 (m, 2H), 2.19–2.12 (m, 2H), 1.89–1.79 (m, 1H), 1.11 (t, *J* = 7.5 Hz, 3H), 1.06 (d, *J* = 6.8 Hz, 3H), 0.99 (t, *J* = 7.9 Hz, 9H), 0.90 (s, 9H), 0.59 (q, *J* = 7.9 Hz, 6H), 0.14 (s, 3H), 0.11 (s, 3H). ^**13**^ **C NMR** (100 MHz, CDCl_3_): δ_C_ = 107.6, 87.0, 83.1, 77.5, 66.9, 40.2, 25.9, 22.0, 18.4, 15.2, 14.5, 12.6, 7.6, 4.5, −4.4, −5.0. **HRMS** (ESI^+^) calcd for C_22_H_42_NaOSi_2_ [M + Na^+^] 401.2666; found, 401.2667.

#### 4.4.2 *tert*-Butyldimethyl­((3*S*,4*S*)-4-methylnona-1,6-diyn-3-yloxy)­silane (**20**)

To a solution of **19** (1.29 g, 3.40 mmol, 1.0 equiv) in wet THF/MeOH (68 mL, 1:1 *v*/*v*) was added K_2_CO_3_ (4.70 g, 34.0 mmol, 10.0 equiv) at r.t. After stirring at r.t. for 24 h, the reaction solvents were removed under reduced pressure. The reaction mixture was added to water (15 mL) and extracted with EtOAc (3 × 15 mL). The organic phases were combined, washed with brine, dried over MgSO_4_, filtered, and concentrated in *vacuo*. The crude product was purified by FCC on silica gel (Hexanes/EtOAc = 100:0 to 98:2) to give pure **20** as a colorless oil (0.79 g, 2.98 mmol, 88%). ^**1**^ **H NMR** (400 MHz, CDCl_3_): δ_H_ = 4.34 (dd, *J* = 6.4, 2.1 Hz, 1H), 2.37 (d, *J* = 2.1 Hz, 1H), 2.31–2.21 (m, 2H), 2.20–2.12 (m, 2H), 1.91–1.81 (m, 1H), 1.11 (t, *J* = 7.5 Hz, 3H), 1.06 (d, *J* = 6.8 Hz, 3H), 0.90 (s, 9H), 0.14 (s, 3H), 0.11 (s, 3H). ^**13**^ **C NMR** (100 MHz, CDCl_3_): δ_C_ = 84.2, 83.2, 77.4, 73.2, 66.1, 40.1, 25.9, 22.0, 18.3, 15.0, 14.5, 12.6, −4.5, −5.1. **HRMS** (ESI^+^) calcd for C_16_H_28_NaOSi [M + Na^+^] 287.1802; found, 287.1800.

### 4.5 Synthesis of Alkene Precursor **24**

#### 4.5.1 (3a*R*,4*R*,5*R*,6a*S*)-4-((*S,E*)-3-((*tert*-Butyldimethylsilyl)­oxy)­oct-1-enyl)-5-hydroxyhexahydro-2*H*-cyclopenta­[*b*]­furan-2-one (**23**).

##### 4.5.1.1 *In Situ* Preparation of Cuprate **21**

To a solution of (*S,E*)-((1-bromooct-1-en-3-yl)­oxy) (*tert*-butyl)­dimethylsilane (1.93 g, 6.00 mmol, 1.2 equiv) in Et_2_O (28.8 mL) stirring at −78 °C, *t*-BuLi (1.70 M in pentane, 7.10 mL, 12.0 mmol, 2.4 equiv) was added dropwise and the resulting pale yellow solution was stirred at −78 °C for 2 h prior to warming to −40 °C to stir for a further 2 h before cooling back to −78 °C.

A separate solution of thiophene (480 μL, 506 mg, 6.00 mmol, 1.2 equiv) in THF (28.8 mL) was prepared and cooled to −40 °C, and *n*-BuLi (1.60 M in hexanes, 3.80 mL, 6.00 mmol, 1.2 equiv) was added dropwise. The resulting colorless/very pale yellow, clear solution was stirred at −40 °C for 45 min before cooling to −78 °C, whereupon CuCN (538 mg, 6.00 mmol, 1.2 equiv) was added in a single portion, and the solution was warmed to r.t. The formed thienyl cuprate solution was then added to the above vinyl lithium solution at −78 °C, and THF (28.8 mL) was added (used for washing the flask). The bright yellow reaction mixture was warmed to −20 °C to facilitate the formation of cuprate **21**. The reaction mixture was stirred at −20 °C for 1 h before cooling back to −78 °C.

##### 4.5.1.2 Subsequent Synthesis of Compound **23**

A solution of **12** (761 mg, 5.00 mmol, 1.0 equiv) in THF (28.8 mL) was prepared and added to the solution of cuprate **21** at −78 °C to give a bright orange reaction mixture. The reaction mixture was stirred at −78 °C for 1 h before the sequential addition of TMSCl (3.20 mL, 2.72 g, 25.0 mmol, 5.0 equiv) and NEt_3_ (4.20 mL, 3.04 g, 30.0 mmol, 6.0 equiv). The reaction mixture was warmed to −20 °C to stir for 20 min before quenching by addition of saturated aqueous NH_4_Cl solution (60 mL) and warming to r.t. The aqueous phase was extracted with EtOAc (3 × 60 mL), and the organic phases were then combined, washed further with saturated aqueous NH_4_Cl solution (60 mL), washed with brine, dried over MgSO_4_, filtered, and concentrated in vacuo to give crude **22** as a brown oil, which was used directly in the next step.

In a round-bottom flask, a solution of crude **22** in CH_2_Cl_2_/MeOH (40 mL, 3:1 v/v) was cooled to −78 °C, and a stream of O_3_–*O*_2_(*g*) was bubbled through the reaction solution. The reaction progress was carefully monitored by TLC (CH_2_Cl_2_) until a small amount of **22** remained (∼3.0 min). The reaction mixture was then purged of *O*_3_(*g*) by bubbling *N*_2_(*g*) through the solution for 20 min, followed by portion-wise addition of freshly ground NaBH_4_ (568 mg, 15.0 mmol, 3.0 equiv). The reaction was stirred at −78 °C for 2 h before the dry ice/acetone bath was removed and the reaction was allowed to warm to r.t., at which point it was stirred for a further 1 h. The reaction mixture was then poured over saturated brine solution (50 mL) and extracted with EtOAc (3 × 50 mL). The organic phases were combined, dried over MgSO_4_, filtered, and concentrated in vacuo. The crude product was purified by FCC on silica gel (CH_2_Cl_2_/EtOAc = 95:5 to 90:10) to give pure **23** as a colorless oil (1.27 g, 3.32 mmol, 66%). ^**1**^ **H NMR** (500 MHz, CDCl_3_): δ_H_ = 5.59 (ddd, *J* = 15.4, 5.8, 0.8 Hz, 1H), 5.41 (ddd, *J* = 15.4, 8.1, 1.0 Hz, 1H), 4.92 (td, *J* = 7.0, 2.8 Hz, 1H), 4.09–4.05 (m, 1H), 4.00 (q, *J* = 6.7 Hz, 1H), 2.74 (dd, *J* = 18.1, 9.8 Hz, 1H), 2.63–2.57 (m, 1H), 2.49–2.46 (m, 1H), 2.45–2.44 (m, 1H), 2.36–2.32 (m, 1H), 1.98 (ddd, *J* = 14.9, 6.9, 2.8 Hz, 1H), 1.76 (br s, 1H), 1.49–1.40 (m, 2H), 1.34–1.24 (m, 6H), 0.89–0.86 (m, 12H), 0.04 (s, 3H), 0.01 (s, 3H). ^**13**^ **C NMR** (125 MHz, CDCl_3_): δ_C_ = 175.9, 136.5, 126.7, 81.8, 76.1, 72.0, 55.3, 41.8, 39.1, 37.4, 33.5, 30.9, 25.0, 24.2, 21.8, 17.4, 13.2, −5.3, −5.6. **HRMS** (ESI^+^) calcd for C_21_H_38_NaO_4_Si [M + Na^+^] 405.2432; found, 405.2433.

#### 4.5.2 (3a*R*,4*R*,5*R*,6a*S*)-4-((*S*,*E*)-3-(*tert*-Butyldimethylsilyloxy)­oct-1-enyl)-5-hydroxyhexahydro-2*H*-cyclopenta­[*b*]­furan-2-one (**24**)

To a solution of **23** (2.40 g, 6.27 mmol, 1.0 equiv) in CH_2_Cl_2_ (20.9 mL) were added imidazole (982 mg, 14.4 mmol, 2.3 equiv) and TBSCl (1.42 g, 9.41 mmol, 1.5 equiv) at 0 °C. The reaction mixture was stirred at r.t. for 24 h before the mixture was poured over H_2_O (30 mL), and the aqueous phase was extracted with CH_2_Cl_2_ (3 × 30 mL). The organic phases were combined, washed with brine, dried over MgSO_4_, filtered, and concentrated *in vacuo*. The crude product was purified by FCC on silica gel (Hexanes/EtOAc = 95:5 to 90:10) to give pure **24** as a white, amorphous solid (3.02 g, 6.08 mmol, 97%). ^**1**^ **H NMR** (500 MHz, CDCl_3_): δ_H_ = 5.49 (dd, *J* = 15.4, 5.7 Hz, 1H), 5.36 (dd, *J* = 15.4, 7.6 Hz, 1H), 4.94 (td, *J* = 7.0, 2.0 Hz, 1H), 4.03 (q, *J* = 5.9 Hz, 1H), 3.97 (q, *J* = 5.2 Hz, 1H), 2.74 (dd, *J* = 18.0, 10.4 Hz, 1H), 2.63 (dtd, *J* = 10.2 Hz, 6.8 Hz, 2.6 Hz, 1H), 2.48 (dd, *J* = 18.0, 2.4 Hz, 1H), 2.43 (q, *J* = 6.2 Hz, 1H), 2.26–2.21 (m, 1H), 1.97 (dd, *J* = 14.5, 3.2 Hz, 1H), 1.48–1.36 (m, 2H), 1.29–1.25 (m, 6H), 0.88–0.86 (m, 21H), 0.04 (s, 3H), 0.04 (s, 3H), 0.03 (s, 3H), −0.01 (s, 3H). ^**13**^ **C NMR** (125 MHz, CDCl_3_): δ_C_ = 177.2, 136.0, 128.5, 83.5, 78.2, 73.0, 56.8, 42.4, 40.8, 38.5, 35.2, 31.9, 26.0, 25.9, 25.2, 22.7, 18.4, 18.1, 14.2, −4.1, −4.6, −4.6, −4.8. **HRMS** (ESI^+^) calcd for C_27_H_52_NaO_4_Si_2_ [M + Na^+^] 519.3296; found, 519.3292.

### 4.6 Synthesis of Alkene Precursor **28**

#### 4.6.1 (3a*R*,4*R*,5*R*,6a*S*)-4-((3*S*,4*S*,*E*)-3-(*tert*-Butyldimethylsilyloxy)-4-methyloct-1-en-6-ynyl)-5-hydroxyhexahydro-2*H*-cyclopenta­[*b*]­furan-2-one (27).

##### 4.6.1.1 *In Situ* Preparation of Cuprate **25**

To a solution of **18** (953 mg, 2.52 mmol, 1.2 equiv) in Et_2_O (10.1 mL) stirring at −78 °C, *t*-BuLi (1.70 M in pentane, 3.00 mL, 5.04 mmol, 2.4 equiv) was added dropwise, and the resulting pale yellow solution was stirred at −78 °C for 2 h prior to warming to −40 °C to stir for a further 2 h before cooling back to −78 °C.

A separate solution of thiophene (202 μL, 212 mg, 2.52 mmol, 1.2 equiv) in THF (10.1 mL) was prepared and cooled to −40 °C, and *n*-BuLi (1.60 M in hexanes, 1.60 mL, 2.52 mmol, 1.2 equiv) was added dropwise. The resulting dark yellow, clear solution was stirred at −40 °C for 45 min before cooling to −78 °C, whereupon CuCN (227 mg, 2.52 mmol, 1.2 equiv) was added in a single portion, and the solution was then warmed to r.t. The formed thienyl cuprate solution was then added to the above vinyl lithium solution at −78 °C, and THF (10.1 mL) was added (used for washing the flask). The bright yellow reaction mixture was warmed to −20 °C to facilitate the formation of cuprate **25**. The reaction mixture was stirred at −20 °C for 1 h before cooling back to −78 °C.

##### 4.6.1.2 Subsequent Synthesis of Compound **27**

A solution of **12** (319 mg, 2.10 mmol, 1.0 equiv) in THF (10.1 mL) was prepared and added to the solution of cuprate **25** at −78 °C to give a bright orange reaction mixture. The reaction mixture was stirred at −78 °C for 1 h before the sequential addition of TMSCl (1.30 mL, 1.14 g, 10.5 mmol, 5.0 equiv) and NEt_3_ (1.80 mL, 1.27 g, 12.6 mmol, 6.0 equiv). The reaction mixture was warmed to −20 °C to stir for 20 min before quenching by addition of saturated aqueous NH_4_Cl solution (20 mL) and warming to r.t. The aqueous phase was extracted with EtOAc (3 × 20 mL), and the organic phases were then combined, washed further with saturated aqueous NH_4_Cl solution (20 mL), washed with brine, dried over MgSO_4_, filtered, and concentrated *in vacuo* to give crude **26** as a brown oil, which was used directly in the next step.

In a round-bottom flask, a solution of crude **26** in CH_2_Cl_2_/MeOH (16.8 mL, 3:1 *v*/*v*) was cooled to −78 °C, and a stream of O_3_–*O*_2_(*g*) was bubbled through the reaction solution. The reaction progress was carefully monitored by TLC (CH_2_Cl_2_) until a small amount of **26** remained (∼2.0 min). The reaction mixture was then purged of *O*_3_(*g*) by bubbling *N*_2_(*g*) through the solution for 20 min, followed by portion-wise addition of freshly ground NaBH_4_ (238 mg, 6.30 mmol, 3.0 equiv). The reaction was stirred at −78 °C for 2 h before the dry ice/acetone bath was removed, and the reaction was allowed to warm to r.t., at which point it was stirred for a further 1 h. The reaction mixture was then poured over saturated brine solution (15 mL) and extracted with EtOAc (3 × 15 mL). The organic phases were combined, dried over MgSO_4_, filtered, and concentrated *in vacuo*. The crude product was purified by FCC on silica gel (CH_2_Cl_2_/EtOAc = 95:5 to 90:10) to give pure **27** as a colorless oil (436 mg, 1.11 mmol, 53%). ^**1**^ **H NMR** (400 MHz, CDCl_3_): δ_H_ = 5.55 (dd, *J* = 15.6, 6.3 Hz, 1H), 5.42 (dd, *J* = 15.6, 7.9 Hz, 1H), 4.92 (td, *J* = 7.0, 2.7 Hz, 1H), 4.04–3.97 (m, 2H), 2.75 (dd, *J* = 18.0, 9.8 Hz, 1H), 2.60 (ddd, *J* = 9.7, 8.4, 1.9 Hz, 1H), 2.50–2.43 (m, 2H), 2.39–2.34 (m, 1H), 2.20–2.13 (m, 1H), 2.12–2.04 (m, 1H), 1.99 (ddd, *J* = 14.8, 6.8, 2.7 Hz, 1H), 1.78–1.77 (m, 4H), 1.72–1.63 (m, 1H), 0.92–0.87 (m, 12H), 0.05 (s, 3H), −0.01 (s, 3H). ^**13**^ **C NMR** (100 MHz, CDCl_3_): δ_C_ = 176.9, 135.3, 129.6, 82.8, 77.9, 77.5, 76.8, 75.8, 56.4, 42.8, 40.2, 39.5, 34.5, 26.0, 21.9, 18.3, 15.7, 3.6, −3.9, −4.7. **HRMS** (ESI^+^) calcd for C_22_H_36_NaO_4_Si [M + Na^+^] 415.2275; found, 415.2281.

#### 4.6.2 (3a*R*,4*R*,5*R*,6a*S*)-5-(*tert*-Butyldimethylsilyloxy)-4-((3*S*,4*S*,*E*)-3-(*tert*-butyldimethylsilyloxy)-4-methyloct-1-en-6-ynyl)­hexahydro-2*H*-cyclopenta­[*b*]­furan-2-one (**28**)

To a solution of **27** (500 mg, 1.28 mmol, 1.0 equiv) in CH_2_Cl_2_ (4.3 mL) were added imidazole (200 mg, 2.93 mmol, 2.3 equiv) and TBSCl (288 mg, 1.91 mmol, 1.5 equiv) at 0 °C. The reaction mixture was stirred at r.t. for 24 h before the mixture was poured over H_2_O (6 mL), and the aqueous phase was extracted with CH_2_Cl_2_ (3 × 6 mL). The organic phases were combined, washed with brine, dried over MgSO_4_, filtered, and concentrated *in vacuo*. The crude product was purified by FCC on silica gel (Hexanes/EtOAc = 95:5 to 90:10) to give pure **28** as a white, amorphous solid (557 mg, 1.10 mmol, 86%). ^**1**^ **H NMR** (400 MHz, CDCl_3_): δ_H_ = 5.45 (dd, *J* = 15.5, 5.7 Hz, 1H), 5.38 (dd, *J* = 15.6, 6.7 Hz, 1H), 4.96 (td, *J* = 7.0, 1.9 Hz, 1H), 4.02–3.94 (m, 2H), 2.76 (dd, *J* = 17.7, 10.5 Hz, 1H), 2.69–2.62 (m, 1H), 2.53–2.46 (m, 2H), 2.25–2.18 (m, 1H), 2.17–2.12 (m, 1H), 2.11–2.03 (m, 1H), 1.99 (dd, *J* = 14.7, 2.8 Hz, 1H), 1.78 (t, *J* = 2.5 Hz, 3H), 1.70–1.61 (m, 1H), 0.89–0.87 (m, 21H), 0.05 (s, 6H), 0.04 (s, 3H), −0.02 (s, 3H). ^**13**^ **C NMR** (100 MHz, CDCl_3_): δ_C_ = 177.2, 133.5, 130.5, 83.7, 78.3, 77.8, 76.7, 76.0, 57.1, 42.5, 40.8, 39.7, 35.3, 26.0, 25.9, 22.1, 18.3, 18.1, 15.6, 3.6, −3.9, −4.6, −4.7, −4.8. **HRMS** (ESI^+^) calcd for C_28_H_50_NaO_4_Si_2_ [M + Na^+^] 529.3140; found, 529.3132.

### 4.7 Synthesis of Alkyne Precursor **32**

#### 4.7.1 (3a*R*,4*S*,5*R*,6a*S*)-4-((*S*)-3-(*tert*-Butyldimethylsilyloxy)­oct-1-ynyl)-5-hydroxyhexahydro-2*H*-cyclopenta­[*b*]­furan-2-one (**31**)

To a solution of (*R*)-*tert*-butyldimethyl­(oct-1-yn-3-yloxy)­silane (2.53 g, 10.5 mmol, 1.5 equiv) in anhydrous THF (27.5 mL) stirring at −10 °C, *n*-BuLi (1.6 M in hexanes, 6.60 mL, 10.5 mmol, 1.5 equiv) was added dropwise and the resulting pale yellow solution was stirred at −10 °C for 30 min, whereupon CuI·0.75DMS (2.74 g, 11.6 mmol, 1.65 equiv) was added in one portion, and the mixture was stirred for an additional 1 h. The solution was cooled to −78 °C, and TMSI (1.50 mL, 2.10 g, 10.5 mmol, 1.5 equiv) was added. After 5 min, enal **12** (1.07 g, 7.00 mmol, 1.0 equiv) in anhydrous THF (10.5 mL) was added dropwise to the solution of cuprate **29**. After the addition was completed, the mixture was stirred for an additional 1 h at −78 °C before Et_3_N (4.40 mL, 3.19 g, 31.5 mmol, 4.5 equiv) was added. The reaction mixture was warmed to room temperature to stir for 1 h before diluting by addition of Et_2_O (30 mL). The reaction mixture was quenched by addition of saturated aqueous NaHCO_3_ solution (30 mL). The aqueous phase was extracted with Et_2_O (3 × 30 mL), and the combined organic phases were washed further with brine, dried over MgSO_4_, filtered, and concentrated in *vacuo* to give crude **30** as a brown oil, which was used directly in the next step.

In a round-bottom flask, a solution of crude **30** in CH_2_Cl_2_/MeOH (56.0 mL, 3:1 *v*/*v*) was cooled to −78 °C, and a stream of O_3_–*O*_2_(*g*) was bubbled through the reaction solution. The reaction progress was carefully monitored by TLC (CH_2_Cl_2_) until only a small amount of **30** remained (∼5.0 min). The reaction mixture was then purged of *O*_3_(*g*) by bubbling *N*_2_(*g*) through the solution for 30 min, followed by portion-wise addition of freshly ground NaBH_4_ (794 mg, 21.0 mmol, 3.0 equiv). The reaction was stirred at −78 °C for 2 h before the dry ice/acetone bath was removed, and the reaction was allowed to warm to r.t., at which point it was stirred for a further 1 h. The reaction mixture was then poured over saturated brine solution (28 mL) and extracted with EtOAc (3 × 28 mL). The combined organic phases were washed with brine, dried over MgSO_4_, filtered, and concentrated *in vacuo*. The crude product was purified by FCC on silica gel (CH_2_Cl_2_/EtOAc = 95:5 to 90:10) to give pure **31** as a pale yellow oil (1.36 g, 3.57 mmol, 51%). ^**1**^ **H NMR** (400 MHz, CDCl_3_): δ_H_ = 5.06 (t, *J* = 6.6 Hz, 1H), 4.31–4.24 (m, 2H), 3.03–2.97 (m, 2H), 2.83–2.75 (m, 2H), 2.56 (dd, *J* = 18.6, 3.3 Hz, 1H), 2.27 (ddd, *J* = 15.1, 6.5, 4.5 Hz, 1H), 2.13 (d, *J* = 15.2 Hz, 1H), 1.61–1.49 (m, 2H), 1.39–1.20 (m, 6H), 0.86–0.83 (m, 12H), 0.05 (s, 3H), 0.04 (s, 3H). ^**13**^ **C NMR** (101 MHz, CDCl_3_): δ_C_ = 177.9, 85.7, 84.7, 83.2, 78.0, 63.1, 45.9, 44.7, 40.1, 38.7, 35.9, 31.4, 25.8, 25.0, 22.6, 18.3, 14.0, −4.5, −4.9. **HRMS** (ESI^+^) calcd for C_21_H_36_NaO_4_Si [M + Na^+^] 403.2275; found, 403.2266.

#### 4.7.2 (3a*R*,4*S*,5*R*,6a*S*)-5-(*tert*-Butyldimethylsilyloxy)-4-((*S*)-3-(*tert*-Butyldimethylsilyloxy)­oct-1-ynyl)­hexahydro-2*H*-cyclopenta­[*b*]­furan-2-one (**32**)

To a solution of **31** (1.26 g, 3.31 mmol, 1.0 equiv) in CH_2_Cl_2_ (11.0 mL) were added imidazole (1.50 g, 9.93 mmol, 3.0 equiv) and TBSCl (1.08 g, 15.9 mmol, 4.8 equiv) at 0 °C. The reaction mixture was stirred at r.t. for 24 h before the mixture was poured over H_2_O (22 mL), and the aqueous phase was extracted with CH_2_Cl_2_ (3 × 22 mL). The organic phases were combined, washed with brine, dried over MgSO_4_, filtered, and concentrated *in vacuo*. The crude product was purified by FCC on silica gel (Hexanes/EtOAc = 95:5 to 90:10) to give pure **32** as a white, amorphous solid (1.76 g, 3.27 mmol, 99%). ^**1**^ **H NMR** (400 MHz, CDCl_3_): δ_H_ = 5.03 (t, *J* = 6.8 Hz, 1H), 4.26 (dd, *J* = 6.4, 1.8 Hz, 1H), 4.24–4.22 (m, 1H), 2.99 (ddt, *J* = 11.1, 7.2, 3.5 Hz, 1H), 2.77 (dd, *J* = 18.3, 11.3 Hz, 1H), 2.72–2.69 (m, 1H), 2.50 (dd, *J* = 18.4, 3.7 Hz, 1H), 2.27–2.20 (m, 1H), 2.03 (d, *J* = 14.8 Hz, 1H), 1.59–1.52 (m, 2H), 1.36–1.21 (m, 6H), 0.87–0.83 (m, 12H), 0.83 (s, 9H), 0.05 (s, 3H), 0.04 (s, 6H), 0.04 (s, 3H). ^**13**^ **C NMR** (101 MHz, CDCl_3_): δ_C_ = 176.7, 85.7, 84.2, 83.3, 78.8, 63.0, 46.6, 44.7, 40.8, 38.7, 35.8, 31.4, 25.8, 25.7, 25.0, 22.6, 18.3, 18.0, 14.0, −4.5, −4.9, −4.9, −5.0. **HRMS** (ESI^+^) calcd for C_27_H_50_NaO_4_Si_2_ [M + Na^+^] 517.3140; found, 517.3138.

### 4.8 Synthesis of Alkyne Precursor **36**

#### 4.8.1 (3a*R*,4*S*,5*R*,6a*S*)-4-((3*S*,4*S*)-3-(*tert*-Butyldimethylsilyloxy)-4-methylnona-1,6-diynyl)-5-hydroxyhexahydro-2*H*-cyclopenta­[*b*]­furan-2-one (**35**)

To a solution of alkyne **20** (1.19 g, 4.50 mmol, 1.5 equiv) in anhydrous THF (11.7 mL) stirring at −10 °C, *n*-BuLi (1.6 M in hexanes, 2.81 mL, 4.50 mmol, 1.5 equiv) was added dropwise, and the resulting pale yellow solution was stirred at −10 °C for 30 min, whereupon CuI·0.75DMS (1.17 g, 4.95 mmol, 1.65 equiv) was added in one portion, and the mixture was stirred for an additional 1 h. The solution was cooled to −78 °C, and TMSI (0.85 mL, 1.20 g, 6.00 mmol, 2.0 equiv) was added. After 5 min, enal **12** (456 mg, 3.00 mmol, 1.0 equiv) in anhydrous THF (4.5 mL) was added dropwise to the solution of cuprate **33**. After the addition was completed, the mixture was stirred for an additional 1 h at −78 °C before Et_3_N (1.88 mL, 1.37 g, 13.5 mmol, 4.5 equiv) was added. The reaction mixture was warmed to room temperature to stir for 1 h before diluting by addition of Et_2_O (10 mL). The reaction mixture was quenched by addition of saturated aqueous NaHCO_3_ solution (10 mL). The aqueous phase was extracted with Et_2_O (3 × 10 mL), and the combined organic phases were washed further with brine, dried over MgSO_4_, filtered, and concentrated in *vacuo* to give crude **34** as a brown oil, which was used directly in the next step.

In a round-bottom flask, a solution of crude **34** in CH_2_Cl_2_/MeOH (24.0 mL, 3:1 *v*/*v*) was cooled to −78 °C, and a stream of O_3_–*O*_2_(*g*) was bubbled through the reaction solution. The reaction progress was carefully monitored by TLC (CH_2_Cl_2_) until only a small amount of **34** remained (∼2.0 min). The reaction mixture was then purged of *O*_3_(*g*) by bubbling *N*_2_(*g*) through the solution for 20 min, followed by the portionwise addition of freshly ground NaBH_4_ (341 mg, 9.00 mmol, 3.0 equiv). The reaction was stirred at −78 °C for 2 h before the dry ice/acetone bath was removed, and the reaction was allowed to warm to r.t., at which point it was stirred for a further 1 h. The reaction mixture was then poured over saturated brine solution (12 mL) and extracted with EtOAc (3 × 12 mL). The combined organic phases were washed with brine, dried over MgSO_4_, filtered, and concentrated *in vacuo*. The crude product was purified by FCC on silica gel (CH_2_Cl_2_/EtOAc = 95:5 to 90:10) to give pure **35** as a colorless oil (656 mg, 1.62 mmol, 54%). ^**1**^ **H NMR** (400 MHz, CDCl_3_): δ_H_ = 5.08 (dd, *J* = 10.0, 3.8 Hz, 1H), 4.40–4.28 (m, 2H), 3.02 (ddd, *J* = 14.5, 7.3, 3.6 Hz, 1H), 2.84 (dd, *J* = 16.6, 9.2 Hz, 1H), 2.81–2.77 (m, 1H), 2.60 (dd, *J* = 18.4, 3.1 Hz, 1H), 2.38 (ddd, *J* = 15.2, 6.6, 4.9 Hz, 1H), 2.20–2.12 (m, 4H), 2.03 (dd, *J* = 8.5, 2.4 Hz, 1H), 1.80 (dt, *J* = 12.7, 6.5 Hz, 1H), 1.11 (t, *J* = 7.4 Hz, 3H), 1.01 (d, *J* = 6.8 Hz, 3H), 0.89 (s, 9H), 0.10 (s, 3H), 0.09 (s, 3H). ^**13**^ **C NMR** (101 MHz, CDCl_3_): δ_C_ = 177.0, 84.4, 84.1, 84.0, 83.4, 78.3, 77.3, 66.3, 46.0, 44.8, 40.5, 40.2, 35.7, 25.9, 22.1, 18.3, 15.3, 14.5, 12.6, −4.4, −5.0. **HRMS** (ESI^+^) calcd for C_23_H_36_NaO_4_Si [M + Na^+^] 427.2275; found, 427.2274.

#### 4.8.2 (3a*R*,4*S*,5*R*,6a*S*)-5-(*tert*-Butyldimethylsilyloxy)-4-((3S,4S)-3-(*tert*-butyldimethylsilyloxy)-4-methylnona-1,6-diynyl)­hexahydro-2*H*-cyclopenta­[*b*]­furan-2-one (36)

To a solution of **35** (1.26 g, 3.31 mmol, 1.0 equiv) in CH_2_Cl_2_ (11.0 mL) were added imidazole (1.50 g, 9.93 mmol, 3.0 equiv) and TBSCl (1.08 g, 15.9 mmol, 4.8 equiv) at 0 °C. The reaction mixture was stirred at r.t. for 24 h before the mixture was poured over H_2_O (22.0 mL), and the aqueous phase was extracted with CH_2_Cl_2_ (3 × 20 mL). The organic phases were combined, washed with brine, dried over MgSO_4_, filtered, and concentrated *in vacuo*. The crude product was purified by FCC on silica gel (Hexanes/EtOAc = 95:5 to 90:10) to give pure **36** as a white, amorphous solid (1.76 g, 3.27 mmol, 96%). ^**1**^ **H NMR** (400 MHz, CDCl_3_): δ_H_ = 5.07 (t, *J* = 6.9 Hz, 1H), 4.38–4.25 (m, 2H), 3.06–2.99 (m, 1H), 2.80 (dd, *J* = 18.4, 11.3 Hz, 1H), 2.77–2.75 (m, 1H), 2.54 (dd, *J* = 18.4, 3.8 Hz, 1H), 2.29–2.24 (m, 1H), 2.20–2.06 (m, 5H), 1.79 (dt, *J* = 12.7, 6.5 Hz, 1H), 1.10 (t, *J* = 7.4 Hz, 3H), 1.00 (d, *J* = 6.8 Hz, 3H), 0.89 (s, 9H), 0.86 (s, 9H), 0.09 (s, 3H), 0.08 (s, 3H), 0.07 (s, 3H), 0.07 (s, 3H). ^**13**^ **C NMR** (101 MHz, CDCl_3_): δ_C_ = 176.8, 84.6, 84.4, 84.2, 83.3, 78.9, 77.3, 66.3, 46.7, 44.8, 40.9, 40.2, 36.0, 25.9, 25.8, 22.0, 18.3, 18.1, 15.3, 14.5, 12.5, −4.4, −4.8, −5.0, −5.0. **HRMS** (ESI^+^) calcd for C_29_H_50_NaO_4_Si_2_ [M + Na^+^] 541.3140; found, 541.3132.

### 4.9 Synthesis of Difluorinated Compounds **6**, **7**, **8,** and **9**

#### 4.9.1 (3a*R*,4*R*,5*R*,*6*a*S*)-5-(*tert*-Butyldimethylsilyloxy)-4-((*S*,*E*)-3-(*tert*-butyldimethylsilyloxy)­oct-1-enyl)-3,3-difluorohexahydro-2*H*-cyclopenta­[*b*]­furan-2-one (37)

A solution of NFSI (2.14 g, 6.80 mmol, 4.0 equiv) and ZrCp_2_Cl_2_ (1.99 g, 6.80 mmol, 4.0 equiv) in THF (21.3 mL) was stirred at r.t. for 1 h and then cooled to −78 °C. A solution of **24** (845 mg, 1.70 mmol, 1.0 equiv) in THF (11.3 mL) was added, followed by the dropwise addition of KHMDS (1.0 M in THF, 8.50 mL, 8.50 mmol, 5.0 equiv) over 1 h; the reaction mixture was then stirred at −78 °C for a further hour. The reaction mixture was warmed to r.t. and quenched by addition of saturated NaHCO_3_ solution (30 mL), and the aqueous phase was subsequently extracted with Et_2_O (3 × 30 mL). The organic phases were combined, washed with brine, dried over MgSO_4_, filtered, and concentrated *in vacuo*. The crude product was purified by FCC on silica gel (Hexanes/Et_2_O = 100:0 to 95:5) to give pure **37** as a pale yellow solid (482 mg, 0.90 mmol, 53%). ^**1**^ **H NMR** (400 MHz, CDCl_3_): δ_H_ = 5.55 (ddd, *J* = 15.4, 5.8, 0.9 Hz, 1H), 5.37 (dd, *J* = 15.5, 7.7 Hz, 1H), 5.16–5.13 (m, 1H), 4.08–4.03 (m, 2H), 3.01 (dd, *J* = 8.1, 3.1 Hz, 1H), 2.95 (ddd, *J* = 25.6, 6.9, 3.4 Hz, 1H), 2.14–2.05 (m, 2H), 1.50–1.37 (m, 2H), 1.32–1.21 (m, 6H), 0.90–0.85 (m, 21H), 0.05 (s, 3H), 0.04 (s, 3H), 0.04 (s, 3H), 0.00 (s, 3H). ^**13**^ **C NMR** (100 MHz, CDCl_3_): δ_C_ = 165.5 (dd, ^2^ *J*_CF_ = 35.3 Hz, ^2^ *J*_CF_ = 32.7 Hz), 136.7, 127.1, 116.2 (dd, ^1^ *J*_CF_ = 265.0 Hz, ^1^ *J*_CF_ = 243.9 Hz), 81.8 (dd, ^3^ *J*_CF_ = 3.6 Hz, ^3^ *J*_CF_ = 2.1 Hz), 78.5, 72.9, 51.4 (dd, ^3^ *J*_CF_ = 5.3 Hz, ^3^ *J*_CF_ = 2.4 Hz), 50.0 (dd, ^2^ *J*_CF_ = 23.3 Hz, ^2^ *J*_CF_ = 19.0 Hz), 40.1 (d, ^4^ *J*_CF_ = 0.6 Hz), 38.3, 31.9, 26.0, 25.8, 25.0, 22.7, 18.4, 18.2, 14.1, −4.2, −4.6, −4.9, −5.0. **HRMS** (ESI^+^) calcd for C_27_H_50_F_2_NaO_4_Si_2_ [M + Na^+^] 555.3108; found, 555.3090.

#### 4.9.2 5-((3a*R*,4*R*,5*R*,6a*S*)-5-(*tert*-Butyldimethylsilyloxy)-4-((*S*,*E*)-3-(*tert*-butyldimethylsilyloxy)­oct-1-enyl)-3,3-difluorohexahydro-2*H*-cyclopenta­[*b*]­furan-2-ylidene)­pentanoic Acid (**38**)

NaHMDS (1.0 M in THF, 1.50 mL, 1.50 mmol, 10.0 equiv) was added to a stirring suspension of (4-carboxybutyl)­triphenylphosphonium bromide (332 mg, 0.75 mmol, 5.0 equiv) in THF (1.0 mL). The resulting orange suspension was heated to 70 °C for 1 h before cooling to r.t. **37** (80.0 mg, 0.15 mmol, 1.0 equiv) was added as a solution in THF (0.15 mL) to the orange suspension, and the reaction was stirred at r.t. for 6 h. The reaction mixture was quenched by addition of water (15 mL) and extracted with EtOAc (3 × 15 mL). The combined organic phases were washed with water (15 mL), treated with aqueous HCl (1.0 M, 15 mL), washed with brine, dried over MgSO_4_, filtered, and concentrated *in vacuo*. The crude product was purified by FCC on silica gel (Hexanes/EtOAc = 95:5 to 90:10; then Hexanes/EtOAc/AcOH = 90:9:1) to give **38** as a colorless oil (47.2 mg, 76.5 μmol, 51%, 91:9 *Z*/*E*). ^**1**^ **H NMR** (400 MHz, CDCl_3_) (*Z*)-**38**: δ_H_ = 5.60 (dd, *J* = 15.5, 5.3 Hz, 1H), 5.48 (dd, *J* = 15.4, 6.1 Hz, 1H), 4.81 (td, *J* = 7.4, 5.0 Hz, 1H), 4.75 (td, *J* = 6.3, 3.4 Hz, 1H), 4.07 (q, *J* = 5.8 Hz, 1H), 3.88 (q, *J* = 7.5 Hz, 1H), 2.63–2.54 (m, 2H), 2.44–2.36 (m, 3H), 2.17–2.12 (m, 2H), 1.82 (ddd, *J* = 12.1, 8.2, 3.5 Hz, 1H), 1.79–1.72 (m, 2H), 1.50–1.37 (m, 2H), 1.35–1.26 (m, 6H), 0.96–0.75 (m, 21H), 0.04 (s, 3H), 0.03 (s, 3H), 0.03 (s, 3H), 0.02 (s, 3H). ^**13**^ **C NMR** (100 MHz, CDCl_3_) (*Z*)-**38**: δ_C_ = 178.9, 149.6 (dd, ^2^ *J*_CF_ = 30.4 Hz, ^2^ *J*_CF_ = 25.5 Hz), 136.3, 127.8, 124.2 (dd, ^1^ *J*_CF_ = 255.1 Hz, ^1^ *J*_CF_ = 240.1 Hz), 99.9 (d, ^3^ *J*_CF_ = 4.5 Hz), 81.5 (d, ^3^ *J*_CF_ = 5.4 Hz), 78.1 (d, ^4^ *J*_CF_ = 2.6 Hz), 73.0, 51.7 (dd, ^2^ *J*_CF_ = 25.7 Hz, ^2^ *J*_CF_ = 19.5 Hz), 49.9 (d, ^3^ *J*_CF_ = 4.2 Hz), 41.9, 38.6, 33.4, 31.8, 26.1, 25.9, 25.2, 24.3 (d, ^4^ *J*_CF_ = 3.0 Hz), 24.1 (d, ^5^ *J*_CF_ = 1.9 Hz), 22.8, 18.4, 18.2, 14.2, −4.2, −4.4, −4.5, −4.7. **HRMS** (ESI^+^) calcd for C_32_H_58_F_2_NaO_5_Si_2_ [M + Na^+^] 639.3683; found, 639.3690.

#### 4.9.3 5-((3a*R*,4*R*,5*R*,6a*S*)-3,3-Difluoro-5-hydroxy-4-((*S*,*E*)-3-hydroxyoct-1-enyl)­hexahydro-2*H*-cyclopenta­[*b*]­furan-2-ylidene)­pentanoic Acid (6)

To a stirring solution of **38** (19.0 mg, 30.8 μmol, 1.0 equiv) in THF (0.31 mL) at 0 °C, TBAF (1.0 M in THF, 92.0 μL, 92.4 μmol, 3.0 equiv) was added dropwise. The reaction mixture was warmed to r.t. and stirred for a further 20 h. The reaction was quenched by addition of H_2_O (1 mL), and the aqueous phase was extracted with Et_2_O (3 × 3 mL). The combined organic phases were washed with brine, dried over MgSO_4_, filtered, and concentrated *in vacuo*. The crude product was purified by FCC on silica gel (CH_2_Cl_2_/EtOH/AcOH = 97:2:1 to 91:8:1) to give **6** as a colorless oil (11.4 mg, 30.8 μmol, 95%, 91:9 *Z*/*E*). ^**1**^ **H NMR** (500 MHz, CDCl_3_) (*Z*)-**6**: δ_H_ = 5.61 (dd, *J* = 15.2, 7.6 Hz, 1H), 5.49 (dd, *J* = 15.3, 8.3 Hz, 1H), 4.84 (overlapping, br.s + br.s), 4.82 (ddd, *J* = 8.8, 6.5, 5.1 Hz, 1H), 4.74 (td, *J* = 6.5, 3.0 Hz, 1H), 4.06 (q, *J* = 7.0 Hz, 1H), 3.86 (q, *J* = 8.4 Hz, 1H), 2.62–2.55 (m, 1H), 2.53–2.45 (m, 2H), 2.38–2.33 (m, 2H), 2.30–2.22 (m, 1H), 2.13–2.07 (m, 1H), 1.87–1.79 (m, 2H), 1.74–1.67 (m, 1H), 1.59–1.54 (m, 1H), 1.50–1.42 (m, 1H), 1.31–1.28 (m, 6H), 0.89 (t, *J* = 6.8 Hz, 3H). ^**13**^ **C NMR** (125 MHz, CDCl_3_) (*Z*)-**6**: δ_C_ = 178.1, 149.2 (dd, ^2^ *J*_CF_ = 30.5 Hz, ^2^ *J*_CF_ = 25.6 Hz), 136.6, 130.7, 124.1 (dd, ^1^ *J*_CF_ = 254.6 Hz, ^1^ *J*_CF_ = 241.1 Hz), 100.5 (d, ^3^ *J*_CF_ = 4.6 Hz), 81.1 (d, ^3^ *J*_CF_ = 6.2 Hz), 77.3 (d, ^4^ *J*_CF_ = 1.8 Hz), 73.5, 52.0 (dd, ^2^ *J*_CF_ = 25.6 Hz, ^2^ *J*_CF_ = 19.3 Hz), 50.7 (d, ^3^ *J*_CF_ = 4.4 Hz), 40.4, 37.0, 33.1, 31.8, 25.1, 23.9 (d, ^4^ *J*_CF_ = 2.6 Hz), 23.6 (d, ^5^ *J*_CF_ = 1.3 Hz), 22.7, 14.1. **HRMS** (ESI^+^) calcd for C_20_H_30_F_2_NaO_5_ [M + Na^+^] 411.1954; found, 411.1972.

#### 4.9.4 (3a*R*,4*R*,5*R*,6a*S*)-5-(*tert*-Butyldimethylsilyloxy)-4-((*3*S,4*S*,*E*)-3-(tert-butyldimethylsilyloxy)-4-methyloct-1-en-6-ynyl)-3,3-difluorohexahydro-2*H*-cyclopenta­[*b*]­furan-2-one (39)

A solution of NFSI (883 mg, 2.80 mmol, 4.0 equiv) and ZrCp_2_Cl_2_ (818 mg, 2.80 mmol, 4.0 equiv) in THF (8.8 mL) was stirred at r.t. for 1 h and then cooled to −78 °C. A solution of **28** (355 mg, 0.70 mmol, 1.0 equiv) in THF (4.7 mL) was added, followed by the dropwise addition of KHMDS (1.0 M in THF, 3.50 mL, 3.50 mmol, 5.0 equiv) over 1 h; the reaction mixture was then stirred at −78 °C for a further hour. The reaction mixture was warmed to r.t. and quenched by addition of saturated NaHCO_3_ solution (15 mL), and the aqueous phase was subsequently extracted with Et_2_O (3 × 15 mL). The organic phases were combined, washed with brine, dried over MgSO_4_, filtered, and concentrated *in vacuo*. The crude product was purified by FCC on silica gel (Hexanes/Et_2_O = 100:0 to 90:10) to give pure **39** as a white foam (175 mg, 0.32 mmol, 46%). ^**1**^ **H NMR** (400 MHz, CDCl_3_): δ_H_ = 5.51 (ddd, *J* = 15.5, 6.6, 0.8 Hz, 1H), 5.37 (dd, *J* = 15.5, 7.7 Hz, 1H), 5.15 (t, *J* = 6.0 Hz, 1H), 4.09–4.06 (m, 1H), 3.98–3.95 (m, 1H), 3.04–3.03 (m, 1H), 2.95 (ddd, *J* = 25.8, 6.9, 3.1 Hz, 1H), 2.19–2.10 (m, 3H), 2.09–2.04 (m, 1H), 1.77 (t, *J* = 2.5 Hz, 3H), 1.71–1.64 (m, 1H), 0.89–0.85 (m, 21H), 0.04 (s, 3H), 0.04 (s, 6H), −0.02 (s, 3H). ^**13**^ **C NMR** (100 MHz, CDCl_3_): δ_C_ = 165.4 (dd, ^2^ *J*_CF_ = 35.3 Hz, ^2^ *J*_CF_ = 32.7 Hz), 134.5, 129.0, 116.2 (dd, ^1^ *J*_CF_ = 265.0 Hz, ^1^ *J*_CF_ = 243.9 Hz), 81.8 (dd, ^3^ *J*_CF_ = 3.4 Hz, ^3^ *J*_CF_ = 2.1 Hz), 78.4, 77.6, 76.8, 75.9, 51.6 (dd, ^3^ *J*_CF_ = 5.3 Hz, ^3^ *J*_CF_ = 2.4 Hz), 50.0 (dd, ^2^ *J*_CF_ = 23.4 Hz, ^2^ *J*_CF_ = 19.0 Hz), 40.1, 39.5, 26.0, 25.8, 22.0, 18.3, 18.2, 15.5, 3.6, −4.0, −4.7, −5.0, −5.0. **HRMS** (ESI^+^) calcd for C_28_H_48_F_2_NaO_4_Si_2_ [M + Na^+^] 565.2951; found, 565.2933.

#### 4.9.5 5-((3a*R*,4*R*,5*R*,6a*S*)-5-(*tert*-Butyldimethylsilyloxy)-4-((3*S*,4*S*,*E*)-3-(*tert*-butyldimethylsilyloxy)-4-methyloct-1-en-6-ynyl)-3,3-difluorohexahydro-2*H*-cyclopenta­[*b*]­furan-2-ylidene)­pentanoic Acid (40)

NaHMDS (1.0 M in THF, 0.50 mL, 0.50 mmol, 10.0 equiv) was added to a stirring suspension of (4-carboxybutyl)­triphenylphosphonium bromide (111 mg, 0.25 mmol, 5.0 equiv) in THF (0.34 mL). The resulting orange suspension was heated to 70 °C for 1 h before cooling to r.t. **39** (27.1 mg, 51.0 μmol 1.0 equiv) was added as a solution in THF (50 μL) to the orange suspension, and the reaction was stirred at r.t. for 6 h. The reaction mixture was quenched by addition of water (5 mL) and extracted with EtOAc (3 × 5 mL). The combined organic phases were washed with water (10 mL), treated with aqueous HCl (1.0 M, 10 mL), washed with brine, dried over MgSO_4_, filtered, and concentrated *in vacuo*. The crude product was purified by FCC on silica gel (Hexanes/EtOAc = 95:5 to 90:10; then Hexanes/EtOAc/AcOH = 90:9:1) to give **40** as a colorless oil (13.2 mg, 21.1 μmol, 42%, 91:9 *Z*/*E*). ^**1**^ **H NMR** (400 MHz, CDCl_3_) (*Z*)-**40**: δ_H_ = 5.54–5.52 (m, 2H), 4.81 (td, *J* = 7.5, 4.8 Hz, 1H), 4.76 (td, *J* = 6.6, 3.3 Hz, 1H), 4.02–3.99 (m, 1H), 3.90 (q, *J* = 7.4 Hz, 1H), 2.67–2.63 (m, 1H), 2.62–2.55 (m, 1H), 2.43–2.35 (m, 3H), 2.21–2.13 (m, 3H), 2.11–2.03 (m, 1H), 1.86–1.81 (m, 1H), 1.78 (t, *J* = 2.5 Hz, 3H), 1.77–1.73 (m, 2H), 1.70–1.65 (m, 1H), 0.92–0.88 (m, 12H), 0.87 (s, 9H), 0.05 (s, 3H), 0.04 (s, 3H), 0.04 (s, 3H), 0.01 (s, 3H). ^**13**^ **C NMR** (100 MHz, CDCl_3_) (*Z*)-**40**: δ_C_ = 179.2, 149.6 (dd, ^2^ *J*_CF_ = 30.3 Hz, ^2^ *J*_CF_ = 25.6 Hz), 133.8, 129.7, 124.2 (dd, ^1^ *J*_CF_ = 254.9 Hz, ^1^ *J*_CF_ = 240.4 Hz), 99.9 (d, ^3^ *J*_CF_ = 4.5 Hz), 81.6 (d, ^3^ *J*_CF_ = 5.4 Hz), 78.1, 78.1, 76.6, 75.9, 51.6 (dd, ^2^ *J*_CF_ = 25.7 Hz, ^2^ *J*_CF_ = 19.5 Hz), 49.9 (d, ^3^ *J*_CF_ = 4.1 Hz), 41.9, 39.9, 33.5, 26.0, 25.9, 24.3 (d, ^4^ *J*_CF_ = 2.9 Hz), 24.1 (d, ^5^ *J*_CF_ = 1.2 Hz), 22.2, 18.3, 18.1, 15.5, 3.6, −4.0, −4.4, −4.6, −4.8. **HRMS** (ESI^+^) calcd for C_33_H_56_F_2_NaO_5_Si_2_ [M + Na^+^] 649.3527; found, 649.3539.

#### 4.9.6 5-((3a*R*,4*R*,5*R*,6a*S*)-3,3-Difluoro-5-hydroxy-4-((3*S*,4*S*,*E*)-3-hydroxy-4-methyloct-1-en-6-ynyl)­hexahydro-2*H*-cyclopenta­[*b*]­furan-2-ylidene)­pentanoic Acid (7)

To a stirring solution of **40** (23.7 mg, 37.8 μmol, 1.0 equiv) in THF (0.38 mL) at 0 °C, TBAF (1.0 M in THF, 113 μL, 113 μmol, 3.0 equiv) was added dropwise. The reaction mixture was warmed to r.t. and stirred for a further 20 h. The reaction was quenched by addition of H_2_O (1 mL), and the aqueous phase was extracted with Et_2_O (3 × 3 mL). The combined organic phases were washed with brine, dried over MgSO_4_, filtered, and concentrated *in vacuo*. The crude product was purified by FCC on silica gel (CH_2_Cl_2_/EtOAc/AcOH = 97:2:1 to 91:8:1) to give **7** as a colorless oil (14.8 mg, 37.1 μmol, 98%, 91:9 *Z*/*E*). ^**1**^ **H NMR** (500 MHz, CDCl_3_) (*Z*)-**7**: δ_H_ = 5.62 (dd, *J* = 15.3, 7.8 Hz, 1H), 5.52 (dd, *J* = 15.2, 8.1 Hz, 1H), 4.92–4.56 (overlapping, br.s + br.s, 2H), 4.84–4.80 (m, 1H), 4.74 (td, *J* = 6.3, 2.4 Hz, 1H), 3.97 (t, *J* = 7.6 Hz, 1H), 3.89 (q, *J* = 8.1 Hz, 1H), 2.64–2.47 (m, 3H), 2.37–2.35 (t, *J* = 6.3 Hz, 2H), 2.28–2.21 (m, 3H), 2.13–2.07 (m, 1H), 1.87–1.81 (m, 2H), 1.79 (t, *J* = 2.5 Hz, 3H), 1.76–1.66 (m, 2H), 0.93 (d, *J* = 6.8 Hz, 3H). ^**13**^ **C NMR** (125 MHz, CDCl_3_) (*Z*)-**7**: δ_C_ = 178.3, 149.1 (dd, ^2^ *J*_CF_ = 30.4 Hz, ^2^ *J*_CF_ = 25.2 Hz), 134.7, 131.8, 124.1 (dd, ^1^ *J*_CF_ = 254.5 Hz, ^1^ *J*_CF_ = 241.0 Hz), 100.6 (d, ^3^ *J*_CF_ = 4.4 Hz), 81.1 (d, ^3^ *J*_CF_ = 6.2 Hz), 77.4, 77.3, 77.3, 76.5, 52.0 (dd, ^2^ *J*_CF_ = 25.6 Hz, ^2^ *J*_CF_ = 19.0 Hz), 50.8 (d, ^3^ *J*_CF_ = 4.3 Hz), 40.4, 38.3, 33.3, 24.0 (d, ^4^ *J*_CF_ = 1.6 Hz), 23.7 (d, ^5^ *J*_CF_ = 0.6 Hz), 22.4, 15.7, 3.7. **HRMS** (ESI^+^) calcd for C_21_H_28_F_2_NaO_5_ [M + Na^+^] 421.1797; found, 421.1777.

#### 4.9.7 (3a*R*,4*S*,5*R*,6a*S*)-5-(*tert*-Butyldimethylsilyloxy)-4-((*S*)-3-(*tert-*butyldimethylsilyloxy)­oct-1-ynyl)-3,3-difluorohexahydro-2*H*-cyclopenta­[*b*]­furan-2-one (41)

A solution of NFSI (1.89 g, 6.00 mmol, 4.0 equiv) and ZrCp_2_Cl_2_ (1.75 g, 6.00 mmol, 4.0 equiv) in THF (19.0 mL) was stirred at r.t. for 1 h and then cooled to −78 °C. A solution of **32** (742 mg, 1.50 mmol, 1.0 equiv) in THF (10.0 mL) was added, followed by the dropwise addition of KHMDS (1.0 M in THF, 7.50 mL, 7.50 mmol, 5.0 equiv) over 1 h; the reaction mixture was then stirred at −78 °C for a further hour. The reaction mixture was warmed to r.t. and quenched by addition of saturated NaHCO_3_ solution (20 mL), and the aqueous phase was subsequently extracted with Et_2_O (3 × 20 mL). The organic phases were combined, washed with brine, dried over MgSO_4_, filtered, and concentrated *in vacuo*. The crude product was purified by FCC on silica gel (Hexanes/Et_2_O = 100:0 to 95:5) to give pure **41** as a yellow oil (422 mg, 0.80 mmol, 53%). ^**1**^ **H NMR** (400 MHz, CDCl_3_): δ_H_ = 5.21 (t, *J* = 6.2 Hz, 1H), 4.30–4.29 (m, 1H), 4.29–4.27 (m, 1H), 3.29–3.27 (m, 1H), 3.23–3.21 (m, 1H), 2.25 (ddd, *J* = 14.9, 5.7, 3.6 Hz, 1H), 2.17 (d, *J* = 14.9 Hz, 1H), 1.65–1.53 (m, 2H), 1.39–1.25 (m, 6H), 0.92–0.87 (m, 12H), 0.84 (s, 9H), 0.09 (s, 3H), 0.08 (s, 3H), 0.06 (s, 3H), 0.05 (s, 3H). ^**13**^ **C NMR** (100 MHz, CDCl_3_): δ_C_ = 165.1 (dd, ^2^ *J*_CF_ = 34.5 Hz, ^2^ *J*_CF_ = 33.2 Hz), 115.2 (dd, ^1^ *J*_CF_ = 265.8 Hz, ^1^ *J*_CF_ = 244.3 Hz), 86.5, 81.9 (dd, ^3^ *J*_CF_ = 2.7 Hz, ^3^ *J*_CF_ = 2.6 Hz), 81.6, 78.7, 63.1, 51.7 (dd, ^2^ *J*_CF_ = 24.9 Hz, ^2^ *J*_CF_ = 18.5 Hz), 41.3 (dd, ^3^ *J*_CF_ = 5.8, ^3^ *J*_CF_ = 4.1 Hz), 40.4, 38.7, 31.5, 25.9, 25.7, 25.1, 22.7, 18.4, 18.2, 14.1, −4.5, −4.8, −5.1, −5.1. **HRMS** (ESI^+^) calcd for C_27_H_48_F_2_NaO_4_Si_2_ [M + Na^+^] 553.2951; found, 553.2945.

#### 4.9.8 5-((3a*R*,4*S*,5*R*,6a*S*)-5-(*tert*-Butyldimethylsilyloxy)-4-((*S*)-3-(*tert*-butyldimethylsilyloxy)­oct-1-ynyl)-3,3-difluorohexahydro-2*H*-cyclopenta­[*b*]­furan-2-ylidene)­pentanoic Acid (42)

LiHMDS (1.0 M in THF, 0.96 mL, 0.96 mmol, 3.2 equiv) was added to a stirring suspension of (4-carboxybutyl)­triphenylphosphonium bromide (200 mg, 0.45 mmol, 1.5 equiv) in THF (1.5 mL). The resulting orange suspension was stirred at r.t. for 1 h. **41** (159 mg, 0.30 mmol, 1.0 equiv) was added as a solution in THF (1.5 mL) to the orange suspension, and the reaction was stirred at −40 °C for 6 h. The reaction mixture was quenched by addition of water (30 mL) and extracted with EtOAc (3 × 30 mL). The combined organic phases were washed with water (20 mL), treated with aqueous HCl (1.0 M, 30 mL), washed with brine, dried over MgSO_4_, filtered, and concentrated *in vacuo*. The crude product was purified by FCC on silica gel (Hexanes/EtOAc = 95:5; then Hexanes/EtOAc/AcOH = 90:9:1) to give **42** as a colorless oil (29.0 mg, 47.2 μmol, 16%, 96:4 *Z*/*E*). ^**1**^ **H NMR** (400 MHz, CDCl_3_) (*Z*)-**42**: δ_H_ = 4.96–4.73 (m, 2H), 4.35–4.31 (m, 1H), 4.17 (q, *J* = 6.3 Hz, 1H), 2.97–2.82 (m, 2H), 2.40–2.32 (m, 3H), 2.18–2.10 (m, 2H), 1.90–1.84 (m, 1H), 1.78–1.71 (m, 2H), 1.67–1.57 (m, 2H), 1.30–1.26 (m, 6H), 0.91–0.88 (m, 12H), 0.87 (s, 9H), 0.11 (s, 3H), 0.09 (s, 3H), 0.08 (s, 3H), 0.07 (s, 3H). ^**13**^ **C NMR** (100 MHz, CDCl_3_) (*Z*)-**42**: δ_C_ = 179.2, 149.5 (dd, ^2^ *J*_CF_ = 30.1 Hz, ^2^ *J*_CF_ = 25.8 Hz), 123.6 (dd, ^1^ *J*_CF_ = 255.3 Hz, ^1^ *J*_CF_ = 240.7 Hz), 100.1 (d, ^3^ *J*_CF_ = 4.4 Hz), 84.7, 83.1, 82.1 (dd, ^3^ *J*_CF_ = 5.1 Hz, ^3^ *J*_CF_ = 1.0 Hz), 78.8 (d, ^4^ *J*_CF_ = 1.9 Hz), 63.2, 53.9 (dd, ^2^ *J*_CF_ = 27.2 Hz, ^2^ *J*_CF_ = 19.5 Hz), 41.8, 39.8 (dd, ^3^ *J*_CF_ = 4.5 Hz, ^3^ *J*_CF_ = 2.1 Hz), 38.9, 33.5, 31.6, 26.0, 25.8, 25.1, 24.3 (d, ^4^ *J*_CF_ = 2.8 Hz), 24.1 (d, ^5^ *J*_CF_ = 1.2 Hz), 22.7, 18.4, 18.2, 14.2, −4.4, −4.6, −4.8, −4.9. **HRMS** (ESI^+^) calcd for C_32_H_56_F_2_NaO_5_Si_2_ [M + Na^+^] 637.3527; found, 637.3529.

#### 4.9.9 5-((3a*R*,4*S*,5*R*,6a*S*)-3,3-Difluoro-5-hydroxy-4-((*S*)-3-hydroxyoct-1-ynyl)­hexahydro-2*H*-cyclopenta­[*b*]­furan-2-ylidene)­pentanoic Acid (8)

To a stirring solution of **42** (16.6 mg, 27.0 μmol, 1.0 eq., 96:4 *Z*/*E*) in THF (0.27 mL) at 0 °C, TBAF (1.0 M in THF, 81.0 μL, 81.0 μmol, 3.0 equiv) was added dropwise. The reaction mixture was warmed to r.t. and stirred for a further 20 h. The reaction was quenched by addition of H2O (1 mL), and the aqueous phase was extracted with Et_2_O (3 × 2 mL). The combined organic phases were washed with brine, dried over MgSO_4_, filtered, and concentrated *in vacuo*. The crude product was purified by FCC on silica gel (CH_2_Cl_2_/EtOH/AcOH = 97:2:1 to 91:8:1) to give **8b** as a colorless oil (10.0 mg, 25.9 μmol, 96%, 96:4 *Z*/*E*). ^**1**^ **H NMR** (500 MHz, CDCl_3_) (*Z*)-**8**: δ_H_ = 4.88–4.83 (m, 2H), 4.35 (t, *J* = 6.5 Hz, 1H), 4.25–4.21 (m, 1H), 4.05 (overlapping, br.s + br.s, 2H), 2.94–2.87 (m, 2H), 2.49–2.44 (m, 1H), 2.38–2.32 (m, 2H), 2.23–2.19 (m, 1H), 2.14–2.06 (m, 1H), 1.97 (dd, *J* = 14.2, 6.1 Hz, 1H), 1.85–1.78 (m, 1H), 1.72–1.64 (m, 3H), 1.45–1.39 (m, 2H), 1.34–1.25 (m, 4H), 0.90 (t, *J* = 6.9 Hz, 3H). ^**13**^ **C NMR** (125 MHz, CDCl_3_) (*Z*)-**8**: δ_C_ = 178.6, 149.1 (dd, ^2^ *J*_CF_ = 30.2 Hz, ^2^ *J*_CF_ = 25.8 Hz), 123.5 (dd, ^1^ *J*_CF_ = 254.9 Hz, ^1^ *J*_CF_ = 241.5 Hz), 101.0 (d, ^3^ *J*_CF_ = 4.5 Hz), 84.2, 83.8, 82.3 (d, ^3^ *J*_CF_ = 5.6 Hz), 78.7, 62.7, 54.2 (dd, ^2^ *J*_CF_ = 26.9 Hz, ^2^ *J*_CF_ = 19.7 Hz), 40.4, 39.7 (d, ^3^ *J*_CF_ = 4.2 Hz), 38.0, 31.5, 29.9, 25.0, 24.1, 23.8, 22.7, 14.1. **HRMS** (ESI^+^) calcd for C_20_H_28_F_2_NaO_5_ [M + Na^+^] 409.1797; found, 409.1806.

#### 4.9.10 (3a*R*,4*S*,5*R*,6a*S*)-5-(*tert*-Butyldimethylsilyloxy)-4-((3*S*,4*S*)-3-(*tert*-butyldimethylsilyloxy)-4-methylnona-1,6-diynyl)-3,3-difluorohexahydro-2*H*-cyclopenta­[*b*]­furan-2-one (43)

A solution of NFSI (1.00 g, 3.20 mmol, 4.0 equiv) and ZrCp_2_Cl_2_ (935 mg, 3.20 mmol, 4.0 equiv) in THF (10.0 mL) was stirred at r.t. for 1 h and then cooled to −78 °C. A solution of **36** (415 mg, 0.80 mmol, 1.0 equiv) in THF (5.3 mL) was added, followed by the dropwise addition of KHMDS (1.0 M in THF, 4.00 mL, 4.00 mmol, 5.0 equiv) over 1 h; the reaction mixture was then stirred at −78 °C for a further hour. The reaction mixture was warmed to r.t. and quenched by addition of saturated NaHCO_3_ solution (10 mL), and the aqueous phase was subsequently extracted with Et_2_O (3 × 10 mL). The organic phases were combined, washed with brine, dried over MgSO_4_, filtered, and concentrated *in vacuo*. The crude product was purified by FCC on silica gel (Hexanes/Et_2_O = 100:0 to 95:5) to give pure **43** as a yellow oil (223 mg, 0.40 mmol, 50%). ^**1**^ **H NMR** (400 MHz, CDCl_3_): δ_H_ = 5.23 (t, *J* = 6.0 Hz, 1H), 4.41–4.27 (m, 2H), 3.30–3.22 (m, 2H), 2.29–2.23 (m, 1H), 2.21–2.13 (m, 5H), 1.81 (dt, *J* = 13.0, 6.4 Hz, 1H), 1.12 (t, *J* = 7.4 Hz, 3H), 1.01 (d, *J* = 6.8 Hz, 3H), 0.90 (s, 9H), 0.85 (s, 9H), 0.10 (s, 3H), 0.09 (s, 3H), 0.07 (s, 3H), 0.06 (s, 3H). ^**13**^ **C NMR** (100 MHz, CDCl_3_): δ_C_ = 165.1 (dd, ^2^ *J*_CF_ = 34.8 Hz, ^2^ *J*_CF_ = 32.8 Hz), 115.2 (dd, ^1^ *J*_CF_ = 265.9 Hz, ^1^ *J*_CF_ = 244.4 Hz), 84.9, 83.5, 82.8, 81.9 (d, ^3^ *J*_CF_ = 2.9 Hz, ^3^ *J*_CF_ = 2.5 Hz), 78.7, 77.1, 66.3, 51.7 (dd, ^2^ *J*_CF_ = 25.0 Hz, ^2^ *J*_CF_ = 18.5 Hz), 41.4 (dd, ^3^ *J*_CF_ = 5.9 Hz, ^3^ *J*_CF_ = 4.1 Hz), 40.5, 40.1, 25.9, 25.7, 22.1, 18.3, 18.2, 15.3, 14.5, 12.6, −4.5, −5.0, −5.1, −5.1. **HRMS** (ESI^+^) calcd for C_29_H_48_F_2_NaO_4_Si_2_ [M + Na^+^] 577.2951; found, 577.2960.

#### 4.9.11 5-((3a*R*,4*S*,5*R*,6a*S*)-5-(*tert*-Butyldimethylsilyloxy)-4-((3*S*,4*S*)-3-(*tert*-butyldimethylsilyloxy)-4-methylnona-1,6-diynyl)-3,3-difluorohexahydro-2*H*-cyclopenta­[*b*]­furan-2-ylidene)­pentanoic Acid (44)

NaHMDS (1.0 M in THF, 1.26 mL, 1.26 mmol, 10.0 equiv) was added to a stirring suspension of (4-carboxybutyl)­triphenylphosphonium bromide (279 mg, 0.63 mmol, 5.0 equiv) in THF (0.89 mL). The resulting orange suspension was heated to 70 °C for 1 h before cooling to r.t. **43** (70.0 mg, 126 μmol, 1.0 equiv) was added as a solution in THF (0.13 mL) to the orange suspension, and the reaction was stirred at r.t. for 3 h. The reaction mixture was quenched by addition of water (25.2 mL) and extracted with EtOAc (3 × 12.5 mL). The combined organic phases were washed with water (12.5 mL), treated with aqueous HCl (0.1 M, 12.5 mL), washed with brine, dried over MgSO_4_, filtered, and concentrated *in vacuo*. The crude product was purified by FCC on silica gel (Hexanes/EtOAc = 95:5 to 90:10; then Hexanes/EtOAc/AcOH = 90:9:1 to 80:19:1) to give **44** as a colorless oil (39.5 mg, 61.8 μmol, 49%, 84:16 *Z*/*E*). ^**1**^ **H NMR** (500 MHz, CDCl_3_) (*Z*)-**44**: δ_H_ = 4.83–4.75 (m, 2H), 4.30 (dd, *J* = 6.6, 1.1 Hz, 1H), 4.17 (q, *J* = 6.2 Hz, 1H), 2.94–2.82 (m, 2H), 2.39–2.34 (m, 3H), 2.25–2.13 (m, 5H), 1.90–1.86 (m, 1H), 1.84–1.79 (m, 1H), 1.76–1.71 (m, 2H), 1.63–1.58 (m, 1H), 1.12 (t, *J* = 7.4 Hz, 3H), 1.03 (d, *J* = 6.8 Hz, 3H), 0.90 (s, 9H), 0.87 (s, 9H), 0.12 (s, 3H), 0.10 (s, 3H), 0.08 (s, 3H), 0.07 (s, 3H). ^**13**^ **C NMR** (125 MHz, CDCl_3_) (*Z*)-**44**: δ_C_ = 178.9, 149.5 (dd, ^2^ *J*_CF_ = 29.9 Hz, ^2^ *J*_CF_ = 25.9 Hz), 123.6 (dd, ^1^ *J*_CF_ = 255.2 Hz, ^1^ *J*_CF_ = 240.7 Hz), 100.2 (d, ^3^ *J*_CF_ = 4.4 Hz), 84.3, 83.2, 83.1, 82.2 (d, ^3^ *J*_CF_ = 4.3 Hz), 78.8 (d, ^4^ *J*_CF_ = 1.8 Hz), 77.5, 66.4, 54.0 (dd, ^2^ *J*_CF_ = 27.2 Hz, ^2^ *J*_CF_ = 19.4 Hz), 41.8, 40.2, 39.9 (dd, ^3^ *J*_CF_ = 4.4 Hz, ^3^ *J*_CF_ = 2.1 Hz), 29.9, 25.9, 25.8, 24.3 (d, ^5^ *J*_CF_ = 2.8 Hz), 24.1 (d, ^4^ *J*_CF_ = 0.7 Hz), 22.0, 18.3, 18.1, 15.3, 14.5, 12.6, −4.4, −4.6, −4.7, −5.0. **HRMS** (ESI^+^) calcd for C_34_H_56_F_2_NaO_5_Si_2_ [M + Na^+^] 661.3527; found, 661.3542.

#### 4.9.12 5-((3a*R*,4*S*,5*R*,6a*S*)-3,3-Difluoro-5-hydroxy-4-((3*S*,4*S*)-3-hydroxy-4-methylnona-1,6-diynyl)­hexahydro-2*H*-cyclopenta­[*b*]­furan-2-ylidene)­pentanoic Acid (9)

To a stirring solution of **44** (16.0 mg, 25.0 μmol, 1.0 equiv) in THF (0.25 mL) at 0 °C, TBAF (1.0 M in THF, 75.0 μL, 75.0 μmol, 3.0 equiv) was added dropwise. The reaction mixture was warmed to r.t. and stirred for a further 20 h. The reaction was quenched by addition of H_2_O (1.2 mL), and the aqueous phase was extracted with Et_2_O (3 × 2.5 mL). The combined organic phases were washed with brine, dried over MgSO_4_, filtered, and concentrated *in vacuo*. The crude product was purified by FCC on silica gel (CH_2_Cl_2_/EtOAc/AcOH = 60:39:1 to 40:59:1) to give **9** as a colorless oil (9.40 mg, 22.7 μmol, 91%, 85:15 *Z*/*E*). ^**1**^ **H NMR** (500 MHz, CDCl_3_) (*Z*)-**9**: δ_H_ = 4.88–4.83 (m, 2H), 4.65 (overlapping, br.s + br.s, 2H), 4.36 (d, *J* = 6.2 Hz, 1H), 4.27–4.21 (m, 1H), 2.93–2.89 (m, 2H), 2.50–2.44 (m, 1H), 2.38–2.32 (m, 2H), 2.26–2.21 (m, 2H), 2.19–2.07 (m, 4H), 2.01–1.95 (m, 1H), 1.92–1.87 (m, 1H), 1.85–1.78 (m, 1H), 1.74–1.66 (m, 1H), 1.12 (t, *J* = 7.5 Hz, 3H), 1.07 (d, *J* = 6.8 Hz, 3H). ^**13**^ **C NMR** (125 MHz, CDCl_3_) (*Z*)-**9**: δ_C_ = 178.9, 149.0 (dd, ^2^ *J*_CF_ = 30.0 Hz, ^2^ *J*_CF_ = 25.5 Hz), 123.5 (dd, ^1^ *J*_CF_ = 254.9 Hz, ^1^ *J*_CF_ = 241.4 Hz), 101.1 (d, ^3^ *J*_CF_ = 4.3 Hz), 85.0, 83.8, 82.4, 82.2 (d, ^3^ *J*_CF_ = 5.3 Hz), 78.6, 77.1, 66.2, 54.1 (dd, ^2^ *J*_CF_ = 27.2 Hz, ^2^ *J*_CF_ = 19.4 Hz), 40.4, 39.7 (d, ^3^ *J*_CF_ = 4.0 Hz), 39.3, 33.4, 24.1, 23.7, 22.3, 15.3, 14.4, 12.6. **HRMS** (ESI^+^) calcd for C_22_H_28_F_2_NaO_5_ [M + Na^+^] 433.1797; found, 433.1780.

### 4.10 Synthesis of Monofluorinated Targets **2**, **3**, **4**, and **5**

#### 4.10.1 (3*S*,3a*R*,4*R*,5*R*,6a*S*)-5-(*tert*-Butyldimethylsilyloxy)-4-((*S*,*E*)-3-(*tert*-butyldimethylsilyloxy)­oct-1-enyl)-3-fluorohexahydro-2*H*-cyclopenta­[*b*]­furan-2-one (48)

To a solution of **24** (745 mg, 1.50 mmol, 1.0 equiv) and NFSI (591 mg, 1.88 mmol, 1.25 equiv) in THF (7.5 mL) at −78 °C, LiHMDS (1.0 M in THF, 1.73 mL, 1.73 mmol, 1.15 equiv) was added dropwise. The reaction mixture was stirred at −78 °C for 2 h before it was warmed to r.t., quenched by addition of saturated NaHCO_3_ solution (15 mL), and the aqueous phase was extracted with Et_2_O (3 × 15 mL). The organic phases were combined, washed with brine, dried over MgSO_4_, filtered, and concentrated *in vacuo*. The crude product was purified by FCC on silica gel (Hexanes/Et_2_O = 100:0 to 95:5) to give pure **48** as a colorless oil (602 mg, 1.17 mmol, 78%, > 95:5 dr). ^**1**^ **H NMR** (400 MHz, CDCl_3_): δ_H_ = 5.51 (ddd, *J* = 15.5, 5.8, 1.0 Hz, 1H), 5.36 (ddd, *J* = 15.5, 7.3, 1.2 Hz, 1H), 5.18–5.14 (m, 1H), 5.08 (dd, *J* = 51.9, 2.4 Hz, 1H), 4.07–4.06 (m, 1H), 4.05–4.02 (m, 1H), 2.93 (ddt, *J* = 32.6, 7.4, 2.5 Hz, 1H), 2.81 (d, *J* = 7.0 Hz, 1H), 2.10–1.99 (m, 2H), 1.49–1.36 (m, 2H), 1.32–1.21 (m, 6H), 0.90–0.85 (m, 21H), 0.06 (s, 3H), 0.05 (s, 3H), 0.03 (s, 3H), −0.00 (s, 3H). ^**13**^ **C NMR** (100 MHz, CDCl_3_): δ_C_ = 172.0 (d, ^2^ *J*_CF_ = 21.2 Hz) 136.2, 127.4, 92.1 (d, ^1^ *J*_CF_ = 183.4 Hz), 83.2 (d, ^3^ *J*_CF_ = 2.7 Hz), 78.6, 73.0, 55.0 (d, ^3^ *J*_CF_ = 4.9 Hz), 50.2 (d, ^2^ *J*_CF_ = 20.2 Hz), 39.7 (d, ^4^ *J*_CF_ = 1.5 Hz), 38.4, 31.9, 26.0, 25.8, 25.1, 22.7, 18.4, 18.2, 14.2, −4.2, −4.5, −4.8, −4.9. **HRMS** (ESI^+^) calcd for C_27_H_51_FNaO_4_Si_2_ [M + Na^+^] 537.3202; found, 537.3197.

#### 4.10.2 5-((3*S*,3a*R*,4*R*,5*R*,6a*S*)-5-(*tert*-Butyldimethylsilyloxy)-4-((*S*,*E*)-3-(*tert-*butyldimethylsilyloxy)­oct-1-enyl)-3-fluorohexahydro-2*H*-cyclopenta­[*b*]­furan-2-ylidene)­pentanoic Acid (49)

LiHMDS (1.0 M in THF, 0.26 mL, 0.26 mmol, 3.2 equiv) was added to a stirring suspension of (4-carboxybutyl)­triphenylphosphonium bromide (53.2 mg, 0.12 mmol, 1.5 equiv) in THF (0.4 mL). The resulting orange suspension was stirred at r.t. for 1 h. **48** (41.2 mg, 80.0 μmol, 1.0 equiv) was added as a solution in THF (0.40 mL) to the orange suspension, and the reaction was stirred at r.t. for 3 h. The reaction mixture was quenched by addition of water (8 mL) and extracted with EtOAc (3 × 8 mL). The combined organic phases were washed with water (8 mL), treated with aqueous HCl (0.1 M, 8 mL), washed with brine, dried over MgSO_4_, filtered, and concentrated *in vacuo*. The crude product was purified by FCC on silica gel (Hexanes/EtOAc = 95:5; then Hexanes/EtOAc/AcOH = 95:4:1) to give **49** as a colorless oil (19.2 mg, 32.1 μmol, 40%, 83:17 *Z*/*E*). ^**1**^ **H NMR** (500 MHz, CDCl_3_) (*Z*)-**49**: δ_H_ = 5.58 (dd, *J* = 15.4, 5.2 Hz, 1H), 5.48 (ddd, *J* = 15.4, 7.7, 0.9 Hz, 1H), 4.91 (d, *J* = 56.0 Hz, 1H), 4.80 (td, *J* = 6.7, 2.9 Hz, 1H), 4.67 (q, *J* = 7.3 Hz, 1H), 4.10–4.06 (m, 1H), 3.86 (q, *J* = 8.0 Hz, 1H), 2.47–2.36 (m, 4H), 2.20–2.14 (m, 2H), 2.09–2.04 (m, 1H), 1.77–1.71 (m, 3H), 1.48–1.40 (m, 2H), 1.35–1.25 (m, 6H), 0.90–0.85 (m, 21H), 0.04 (s, 3H), 0.03 (s, 3H), 0.02 (s, 3H), 0.02 (s, 3H). ^**13**^ **C NMR** (125 MHz, CDCl_3_) (*Z*)-**49**: δ_C_ = 179.4, 153.9 (d, ^2^ *J*_CF_ = 14.7 Hz), 136.7, 128.1, 103.8 (d, ^3^ *J*_CF_ = 10.6 Hz), 95.0 (d, ^1^ *J*_CF_ = 176.0 Hz), 82.9, 78.0 (d, ^4^ *J*_CF_ = 2.7 Hz), 72.8, 51.9 (d, ^2^ *J*_CF_ = 21.4 Hz), 51.0 (d, ^3^ *J*_CF_ = 6.5 Hz), 41.6, 38.6, 33.6, 32.0, 26.1, 25.9, 25.2, 24.9 (d, ^5^ *J*_CF_ = 3.8 Hz), 24.5 (d, ^4^ *J*_CF_ = 5.2 Hz), 22.8, 18.2, 18.2, 14.2, −4.1, −4.4, −4.5, −4.6. **HRMS** (ESI^+^) calcd for C_32_H_59_FNaO_5_Si_2_ [M + Na^+^] 621.3777; found, 621.3779.

#### 4.10.3 5-((3*S*,3a*R*,4*R*,5*R*,6a*S*)-3-Fluoro-5-hydroxy-4-((*S*,*E*)-3-hydroxyoct-1-enyl)­hexahydro-2*H*-cyclopenta­[*b*]­furan-2-ylidene)­pentanoic Acid (2)

To a stirring solution of **49** (12.0 mg, 20.0 μmol, 1.0 eq., 83:17 *Z*/*E*) in THF (0.2 mL) at 0 °C, TBAF (1.0 M in THF, 60.0 μL, 60.0 μmol, 3.0 equiv) was added dropwise. The reaction mixture was warmed to r.t. and stirred for a further 20 h. The reaction was quenched by addition of H_2_O (1 mL), and the aqueous phase was extracted with Et_2_O (3 × 3 mL). The combined organic phases were washed with brine, dried over MgSO_4_, filtered, and concentrated *in vacuo*. The crude product was purified by FCC on silica gel (CH_2_Cl_2_/EtOAc/AcOH = 60:39:1 to 40:59:1) to give **2a** as a colorless oil (6.80 mg, 18.4 μmol, 91%, 82:18 *Z*/*E*). ^**1**^ **H NMR** (500 MHz, CD_2_Cl_2_+CD_3_OD) (*Z*)-**2**: δ_H_ = 5.59–5.53 (m, 1H), 5.53–5.47 (m, 1H), 4.89 (d, *J* = 55.5 Hz, 1H), 4.79 (t, *J* = 6.0 Hz, 1H), 4.72 (dt, *J* = 8.2, 4.1 Hz, 1H), 4.01 (q, *J* = 6.5 Hz, 1H), 3.86–3.80 (m, 1H), 2.56–2.41 (m, 2H), 2.30–2.23 (m, 2H), 2.19–2.01 (m, 2H), 1.95–1.89 (m, 1H), 1.76–1.64 (m, 3H), 1.55–1.50 (m, 1H), 1.47–1.41 (m, 1H), 1.29–1.24 (m, 6H), 0.89 (d, *J* = 6.7 Hz, 3H). ^**13**^ **C NMR** (125 MHz, CD_2_Cl_2_) (*Z*)-**2**: δ_C_ = 178.2, 154.0 (d, ^2^ *J*_CF_ = 13.8 Hz), 137.4, 130.9, 104.9 (d, ^3^ *J*_CF_ = 9.8 Hz), 94.9 (d, ^1^ *J*_CF_ = 175.9 Hz), 83.3, 77.5, 73.3, 52.6 (d, ^2^ *J*_CF_ = 21.6 Hz), 51.8 (d, ^3^ *J*_CF_ = 3.9 Hz), 40.6, 37.4, 32.1, 30.1, 25.6, 24.7 (d, ^4^ *J*_CF_ = 3.2 Hz), 24.5, 23.0, 14.2. **HRMS** (ESI^+^) calcd for C_20_H_31_FNaO_5_ [M + Na^+^] 393.2048; found, 393.2051.

#### 4.10.4 (3*S*,3a*R*,4*R*,5*R*,6a*S*)-5-(*tert*-Butyldimethylsilyloxy)-4-((3*S*,4*S*,*E*)-3-(*tert*-butyldimethylsilyloxy)-4-methyloct-1-en-6-ynyl)-3-fluorohexahydro-2*H*-cyclopenta­[*b*]­furan-2-one (50)

To a solution of **28** (208 mg, 0.42 mmol, 1.0 eq.) and NFSI (165 mg, 0.52 mmol, 1.25 equiv) in THF (2.1 mL) at −78 °C, LiHMDS (1.0 M in THF, 0.48 mL, 0.48 mmol, 1.15 equiv) was added dropwise. The reaction mixture was stirred at −78 °C for 2 h before it was warmed to r.t. and quenched by addition of saturated NaHCO_3_ solution (4 mL), and the aqueous phase was extracted with Et_2_O (3 × 4 mL). The organic phases were combined, washed with brine, dried over MgSO_4_, filtered, and concentrated *in vacuo*. The crude product was purified by FCC on silica gel (Hexanes/Et_2_O = 100:0 to 90:10) to give pure **50** as a white foam (157 mg, 0.30 mmol, 71%, > 95:5 dr). ^**1**^ **H NMR** (400 MHz, CDCl_3_): δ_H_ = 5.47 (dd, *J* = 15.8, 6.4 Hz, 1H), 5.37 (dd, *J* = 15.6, 7.1 Hz, 1H), 5.19–5.16 (m, 1H), 5.09 (dd, *J* = 52.0, 2.5 Hz, 1H), 4.08 (s, 1H), 3.96 (t, *J* = 6.3 Hz, 1H), 2.94 (ddt, *J* = 32.8, 7.5, 2.4 Hz, 1H), 2.85 (d, *J* = 7.0 Hz, 1H), 2.18–2.08 (m, 2H), 2.07–2.00 (m, 2H), 1.77 (t, *J* = 2.5 Hz, 3H), 1.67 (dt, *J* = 12.8, 6.6 Hz, 1H), 0.89–0.87 (m, 12H), 0.85 (s, 9H), 0.06 (s, 3H), 0.05 (s, 3H), 0.04 (s, 3H), −0.02 (s, 3H). ^**13**^ **C NMR** (100 MHz, CDCl_3_): δ_C_ = 172.0 (d, ^2^ *J*_CF_ = 21.3 Hz), 134.1, 129.4, 92.1 (d, ^1^ *J*_CF_ = 183.5 Hz), 83.2 (d, ^3^ *J*_CF_ = 1.5 Hz), 78.6, 77.6, 76.8, 75.9, 55.3 (d, ^3^ *J*_CF_ = 4.9 Hz), 50.2 (d, ^2^ *J*_CF_ = 20.3 Hz), 39.8, 39.5, 26.0, 25.8, 22.1, 18.3, 18.2, 15.6, 3.6, −4.0, −4.7, −4.8, −5.0. **HRMS** (ESI^+^) calcd for C_28_H_49_FNaO_4_Si_2_ [M + Na^+^] 547.3046; found, 547.3049.

#### 4.10.5 5-((3*S*,3a*R*,4*R*,5*R*,6a*S*)-5-(*tert*-Butyldimethylsilyloxy)-4-((3*S*,4*S*,*E*)-3-(*tert*-butyldimethylsilyloxy)-4-methyloct-1-en-6-ynyl)-3-fluorohexahydro-2*H*-cyclopenta­[*b*]­furan-2-ylidene)­pentanoic Acid (51)

LiHMDS (1.0 M in THF, 0.26 mL, 0.26 mmol, 3.2 equiv) was added to a stirring suspension of (4-carboxybutyl)­triphenylphosphonium bromide (53.2 mg, 0.12 mmol, 1.5 equiv) in THF (0.4 mL). The resulting orange suspension was stirred at r.t. for 1 h. **50** (42.0 mg, 80.0 μmol, 1.0 equiv) was added as a solution in THF (0.4 mL) to the orange suspension, and the reaction was stirred at r.t. for 3 h. The reaction mixture was quenched by addition of water (8 mL) and extracted with EtOAc (3 × 8 mL). The combined organic phases were washed with water (8 mL), treated with aqueous HCl (0.1 M, 8 mL), washed with brine, dried over MgSO_4_, filtered, and concentrated *in vacuo*. The crude product was purified by FCC on silica gel (Hexanes/EtOAc = 95:5; then Hexanes/EtOAc/AcOH = 95:4:1) to give **51** as a colorless oil (15.6 mg, 25.6 μmol, 32%, 79:21 *Z*/*E*). ^**1**^ **H NMR** (500 MHz, CD_2_Cl_2_) (*Z*)-**51**: δ_H_ = 5.55–5.54 (m, 2H), 4.95 (d, *J* = 56.1 Hz, 1H), 4.81 (td, *J* = 6.7, 2.8 Hz, 1H), 4.69 (q, *J* = 7.3 Hz, 1H), 4.06–4.04 (m, 1H), 3.92 (q, *J* = 7.8 Hz, 1H), 2.49–2.40 (m, 2H), 2.39–2.36 (m, 2H), 2.22–2.19 (m, 1H), 2.18–2.15 (m, 2H), 2.13–2.04 (m, 3H), 1.76 (t, *J* = 2.5 Hz, 3H), 1.74–1.69 (m, 3H), 0.91–0.90 (m, 12H), 0.87 (s, 9H), 0.07 (s, 3H), 0.04 (s, 3H), 0.04 (s, 3H), 0.03 (s, 3H). ^**13**^ **C NMR** (125 MHz, CD_2_Cl_2_) (*Z*)-**51**: δ_C_ = 178.6, 154.2 (d, ^2^ *J*_CF_ = 14.7 Hz), 134.2, 130.5, 104.0 (d, ^3^ *J*_CF_ = 10.5 Hz), 95.4 (d, ^1^ *J*_CF_ = 175.6 Hz), 83.3, 78.2 (d, ^3^ *J*_CF_ = 2.6 Hz), 78.1, 76.8, 76.1, 52.2 (d, ^2^ *J*_CF_ = 21.5 Hz), 51.5 (d, ^3^ *J*_CF_ = 6.5 Hz), 42.0, 40.2, 33.6, 26.1, 26.0, 25.0 (d, ^5^ *J*_CF_ = 3.8 Hz), 24.8 (d, ^4^ *J*_CF_ = 5.2 Hz), 22.4, 18.5, 18.3, 15.6, 3.5, −3.9, −4.4, −4.6, −4.8. **HRMS** (ESI^+^) calcd for C_33_H_57_FNaO_5_Si_2_ [M + Na^+^] 631.3621; found, 631.3616.

#### 4.10.6 5-((3*S*,3a*R*,4*R*,5*R*,6a*S*)-3-Fluoro-5-hydroxy-4-((3*S*,4*S*,*E*)-3-hydroxy-4-methyloct-1-en-6-ynyl)­hexahydro-2*H*-cyclopenta­[*b*]­furan-2-ylidene)­pentanoic Acid (3)

To a stirring solution of **51** (12.0 mg, 19.7 μmol, 1.0 equiv) in THF (0.2 mL) at 0 °C, TBAF (1.0 M in THF, 59 μL, 59.1 μmol, 3.0 equiv) was added dropwise. The reaction mixture was warmed to r.t. and stirred for a further 20 h. The reaction was quenched by addition of H2O (1 mL), and the aqueous phase was extracted with Et_2_O (3 × 3 mL). The combined organic phases were washed with brine, dried over MgSO_4_, filtered, and concentrated *in vacuo*. The crude product was purified by FCC on silica gel (CH_2_Cl_2_/EtOAc/AcOH = 60:39:1 to 40:59:1) to give **3** as a colorless oil (7.00 mg, 18.4 μmol, 93%, 78:22 *Z*/*E*). ^**1**^ **H NMR** (500 MHz, CD_2_Cl_2_+CD_3_OD) (*Z*)-**3**: δ_H_ = 5.58–5.54 (m, 2H), 4.89 (d, *J* = 55.6 Hz, 1H), 4.79 (td, *J* = 6.4, 1.2 Hz, 1H), 4.75–4.70 (m, 1H), 3.91–3.88 (m, 1H), 3.87–3.80 (m, 1H), 2.57–2.42 (m, 2H), 2.27–2.20 (m, 4H), 2.16–2.11 (m, 2H), 2.08–2.04 (m, 1H), 2.00–1.92 (m, 1H), 1.76 (t, *J* = 2.5 Hz, 3H), 1.71–1.64 (m, 3H), 0.93 (d, *J* = 6.8 Hz, 3H). ^**13**^ **C NMR** (125 MHz, CD_2_Cl_2_) (*Z*)-**3**: δ_C_ = 178.9, 153.9 (d, ^2^ *J*_CF_ = 14.4 Hz), 135.3, 132.3, 105.1 (d, ^3^ *J*_CF_ = 9.9 Hz), 94.9 (d, ^1^ *J*_CF_ = 175.9 Hz), 83.3, 77.6, 77.4, 77.3, 76.5, 52.6 (d, ^2^ *J*_CF_ = 21.7 Hz), 51.9 (d, ^3^ *J*_CF_ = 4.3 Hz), 40.6, 38.5, 32.3, 30.1, 24.8, 22.5, 15.9, 3.6. **HRMS** (ESI^−^) calcd for C_21_H_28_FO_5_ [M-H^–^] 379.1926; found, 379.1909.

#### 4.10.7 (3*S*,3a*R*,4*S*,5*R*,6a*S*)-5-(*tert*-Butyldimethylsilyloxy)-4-((*S*)-3-(*tert*-butyldimethylsilyloxy)­oct-1-ynyl)-3-fluorohexahydro-2*H*-cyclopenta­[*b*]­furan-2-one (52)

To a solution of **32** (650 mg, 1.31 mmol, 1.0 equiv) and NFSI (517 mg, 1.64 mmol, 1.25 equiv) in THF (6.5 mL) at −78 °C, LiHMDS (1.0 M in THF, 1.51 mL, 1.51 mmol, 1.15 equiv) was added dropwise. The reaction mixture was stirred at −78 °C for 2 h before it was warmed to r.t. and quenched by addition of saturated NaHCO_3_ solution (13 mL), and the aqueous phase was extracted with Et_2_O (3 × 13 mL). The organic phases were combined, washed with brine, dried over MgSO_4_, filtered, and concentrated *in vacuo*. The crude product was purified by FCC on silica gel (Hexanes/Et_2_O = 100:0 to 95:5) to give pure **52** as a yellow oil (531 mg, 1.03 mmol, 79%, > 95:5 dr). ^**1**^ **H NMR** (400 MHz, CDCl_3_): δ_H_ = 5.22 (t, *J* = 7.2 Hz, 1H), 5.05 (dd, *J* = 51.7, 2.7 Hz, 1H), 4.28 (dd, *J* = 6.1, 1.8 Hz, 1H), 4.27 (dd, *J* = 6.8, 1.8 Hz, 1H), 3.20 (dd, *J* = 32.2, 7.6 Hz, 1H), 3.04 (s, 1H), 2.25 (ddd, *J* = 15.1, 6.8, 3.7 Hz, 1H), 2.07 (d, *J* = 15.1 Hz, 1H), 1.66–1.54 (m, 2H), 1.41–1.23 (m, 6H), 0.89–0.88 (m, 12H), 0.84 (s, 9H), 0.08 (s, 3H), 0.07 (s, 6H), 0.05 (s, 3H). ^**13**^ **C NMR** (100 MHz, CDCl_3_): δ_C_ = 171.7 (d, ^2^ *J*_CF_ = 21.4 Hz), 91.4 (d, ^1^ *J*_CF_ = 184.4 Hz), 86.7, 83.1 (d, ^3^ *J*_CF_ = 3.0 Hz), 81.9, 78.6, 63.1, 52.0 (d, ^2^ *J*_CF_ = 21.2 Hz), 44.6 (d, ^3^ *J*_CF_ = 5.4 Hz), 40.1 (d, ^4^ *J*_CF_ = 1.2 Hz), 38.7, 31.5, 25.9, 25.7, 25.0, 22.7, 18.4, 18.1, 14.1, −4.5, −4.8, −4.9, −5.1. **HRMS** (ESI^+^) calcd for C_27_H_49_FNaO_4_Si_2_ [M + Na^+^] 535.3046; found, 535.3030.

#### 4.10.8 5-((3*S*,3a*R*,4*S*,5*R*,6a*S*)-5-(*tert*-Butyldimethylsilyloxy)-4-((*S*)-3-(*tert*-butyldimethylsilyloxy)­oct-1-ynyl)-3-fluorohexahydro-2*H*-cyclopenta­[*b*]­furan-2-ylidene)­pentanoic Acid (53)

LiHMDS (1.0 M in THF, 0.64 mL, 0.64 mmol, 3.2 equiv) was added to a stirring suspension of (4-carboxybutyl)­triphenylphosphonium bromide (133 mg, 0.30 mmol, 1.5 equiv) in THF (1.0 mL). The resulting orange suspension was stirred at r.t. for 1 h. **52** (103 mg, 0.20 mmol, 1.0 equiv) was added as a solution in THF (1.0 mL) to the orange suspension, and the reaction was stirred at r.t. for 3 h. The reaction mixture was quenched by addition of water (20 mL) and extracted with EtOAc (3 × 20 mL). The combined organic phases were washed with water (20 mL), treated with aqueous HCl (0.1 M, 20 mL), washed with brine, dried over MgSO_4_, filtered, and concentrated *in vacuo*. The crude product was purified by FCC on silica gel (Hexanes/EtOAc = 95:5; then Hexanes/EtOAc/AcOH = 95:4:1) to give **53** as a colorless oil (35.8 mg, 60.0 μmol, 30%, 87:13 *Z*/*E*). ^**1**^ **H NMR** (500 MHz, CDCl_3_) (*Z*)-**53**: δ_H_ = 5.11 (d, *J* = 55.8 Hz, 1H), 4.84 (td, *J* = 6.6, 2.3 Hz, 1H), 4.68 (q, *J* = 7.3 Hz, 1H), 4.33 (td, *J* = 6.5, 1.7 Hz, 1H), 4.11 (q, *J* = 7.1 Hz, 1H), 2.73 (ddd, *J* = 20.8, 9.6, 6.2 Hz, 1H), 2.41–2.35 (m, 4H), 2.17–2.14 (m, 2H), 1.76–1.70 (m, 3H), 1.65–1.59 (m, 2H), 1.37–1.25 (m, 6H), 0.91–0.90 (m, 12H), 0.87 (s, 9H), 0.11 (s, 3H), 0.10 (s, 3H), 0.08 (s, 3H), 0.07 (s, 3H). ^**13**^ **C NMR** (125 MHz, CDCl_3_) (*Z*)-**53**: δ_C_ = 179.0, 153.6 (d, ^2^ *J*_CF_ = 15.0 Hz), 103.8 (d, ^3^ *J*_CF_ = 10.2 Hz), 95.0 (d, ^1^ *J*_CF_ = 177.0 Hz), 85.0, 83.1, 83.0, 78.3 (d, ^4^ *J*_CF_ = 2.1 Hz), 63.2, 53.9 (d, ^2^ *J*_CF_ = 22.5 Hz), 41.4, 40.7 (d, ^3^ *J*_CF_ = 6.7 Hz), 38.9, 33.5, 31.6, 26.0, 25.9, 25.2, 24.8 (d, ^5^ *J*_CF_ = 3.7 Hz), 24.5 (d, ^4^ *J*_CF_ = 5.1 Hz), 22.7, 18.4, 18.2, 14.2, −4.3, −4.5, −4.7, −4.8. **HRMS** (ESI^+^) calcd for C_32_H_57_FNaO_5_Si_2_ [M + Na^+^] 619.3621; found, 619.3613.

#### 4.10.9 5-((3*S*,3a*R*,4*S*,5*R*,6a*S*)-3-fluoro-5-hydroxy-4-((*S*)-3-hydroxyoct-1-ynyl)­hexahydro-2*H*-cyclopenta­[*b*]­furan-2-ylidene)­pentanoic Acid (4)

To a stirring solution of **53** (16.8 mg, 28.0 μmol, 1.0 equiv) in THF (0.28 mL) at 0 °C, TBAF (1.0 M in THF, 84.0 μL, 84.0 μmol, 3.0 equiv) was added dropwise. The reaction mixture was warmed to r.t. and stirred for a further 20 h. The reaction was quenched by addition of H2O (1.5 mL), and the aqueous phase was extracted with Et_2_O (3 × 3 mL). The combined organic phases were washed with brine, dried over MgSO_4_, filtered, and concentrated *in vacuo*. The crude product was purified by FCC on silica gel (CH_2_Cl_2_/EtOAc/AcOH = 60:39:1 to 40:59:1) to give **4** as a colorless oil (9.70 mg, 26.3 μmol, 94%, 89:11 *Z*/*E*). ^**1**^ **H NMR** (500 MHz, CDCl_3_) (*Z*)-**4**: δ_H_ = 5.13 (d, *J* = 55.1 Hz, 1H), 4.89 (td, *J* = 6.1, 2.0 Hz, 1H), 4.76–4.71 (m, 1H), 4.37 (td, *J* = 6.6, 1.8 Hz, 1H), 4.17 (q, *J* = 7.1 Hz, 1H), 2.76 (ddd, *J* = 19.1, 9.7, 5.7 Hz, 1H), 2.50–2.44 (m, 1H), 2.40–2.35 (m, 3H), 2.27–2.20 (m, 1H), 2.15–2.09 (m, 1H), 1.88 (ddd, *J* = 14.9, 6.6, 1.9 Hz, 1H), 1.80 (td, *J* = 14.0, 7.0 Hz, 1H), 1.70–1.62 (m, 3H), 1.42 (m, 2H), 1.33–1.28 (m, 4H), 0.90 (t, *J* = 7.0 Hz, 3H). ^**13**^ **C NMR** (125 MHz, CDCl_3_) (*Z*)-**4**: δ_C_ = 178.6, 153.4 (d, ^2^ *J*_CF_ = 14.8 Hz), 104.8 (d, ^3^ *J*_CF_ = 10.5 Hz), 94.6 (d, ^1^ *J*_CF_ = 177.2 Hz), 84.5, 83.8, 83.6, 78.4 (d, ^4^ *J*_CF_ = 1.8 Hz), 62.7, 54.2 (d, ^2^ *J*_CF_ = 22.9 Hz), 40.6 (d, ^3^ *J*_CF_ = 7.1 Hz), 40.1, 38.0, 33.2, 31.6, 25.1, 24.5 (d, ^5^ *J*_CF_ = 3.7 Hz), 24.2 (d, ^4^ *J*_CF_ = 5.3 Hz), 22.7, 14.1. **HRMS** (ESI^+^) calcd for C_20_H_29_FNaO_5_ [M + Na^+^] 391.1891; found, 391.1883.

#### 4.10.10 (3*S*,3a*R*,4*S*,5*R*,6a*S*)-5-(*tert*-Butyldimethylsilyloxy)-4-((3*S*,4*S*)-3-(*tert*-butyldimethylsilyloxy)-4-methylnona-1,6-diynyl)-3-fluorohexahydro-2*H*-cyclopenta­[*b*]­furan-2-one (54)

To a solution of **36** (415 mg, 0.80 mmol, 1.0 equiv) and NFSI (315 mg, 1.00 mmol, 1.25 equiv) in THF (4.0 mL) at −78 °C, LiHMDS (1.0 M in THF, 0.92 mL, 0.92 mmol, 1.15 equiv) was added dropwise. The reaction mixture was stirred at −78 °C for 2 h before it was warmed to r.t. and quenched by addition of saturated NaHCO_3_ solution (8 mL), and the aqueous phase was extracted with Et_2_O (3 × 16 mL). The organic phases were combined, washed with brine, dried over MgSO_4_, filtered, and concentrated *in vacuo*. The crude product was purified by FCC on silica gel (Hexanes/Et_2_O = 100:0 to 95:5) to give pure **54** as a colorless oil (240 mg, 0.45 mmol, 56%, > 95:5 dr). ^**1**^ **H NMR** (400 MHz, CDCl_3_): δ_H_ = 5.24 (t, *J* = 7.2 Hz, 1H), 5.07 (dd, *J* = 51.7, 2.7 Hz, 1H), 4.45–4.27 (m, 2H), 3.21 (dd, *J* = 31.5, 7.6 Hz, 1H), 3.07 (s, 1H), 2.29–2.23 (m, 1H), 2.22–2.12 (m, 4H), 2.09 (d, *J* = 15.2 Hz, 1H), 1.85–1.75 (m, 1H), 1.11 (t, *J* = 7.4 Hz, 3H), 1.00 (d, *J* = 6.8 Hz, 3H), 0.90 (s, 9H), 0.85 (s, 9H), 0.10 (s, 3H), 0.09 (s, 3H), 0.08 (s, 3H), 0.07 (s, 3H). ^**13**^ **C NMR** (100 MHz, CDCl_3_): δ_C_ = 171.7 (d, ^2^ *J*_CF_ = 21.4 Hz), 91.4 (d, ^1^ *J*_CF_ = 184.4 Hz), 85.1, 83.4, 83.1 (d, ^3^ *J*_CF_ = 3.1 Hz), 83.2, 78.6, 77.1, 66.3, 52.1 (d, ^2^ *J*_CF_ = 21.2 Hz), 44.7 (d, ^3^ *J*_CF_ = 5.4 Hz), 40.1 (d, ^4^ *J*_CF_ = 1.2 Hz), 40.1, 25.9, 25.8, 22.1, 18.3, 18.1, 15.3, 14.5, 12.6, −4.5, −4.9, −5.0, −5.0. **HRMS** (ESI^+^) calcd for C_29_H_49_FNaO_4_Si_2_ [M + Na^+^] 559.3046; found, 559.3047.

#### 4.10.11 5-((3*S*,3a*R*,4*S*,5*R*,6a*S*)-5-(*tert*-Butyldimethylsilyloxy)-4-((3*S*,4*S*)-3-(*tert*-butyldimethylsilyloxy)-4-methylnona-1,6-diynyl)-3-fluorohexahydro-2*H*-cyclopenta­[*b*]­furan-2-ylidene)­pentanoic Acid (55)

LiHMDS (1.0 M in THF, 0.32 mL, 0.32 mmol, 3.2 equiv) was added to a stirring suspension of (4-carboxybutyl)­triphenylphosphonium bromide (66.5 mg, 0.15 mmol, 1.5 equiv) in THF (0.5 mL). The resulting orange suspension was stirred at r.t. for 1 h. **54** (53.7 mg, 0.10 mmol, 1.0 equiv) was added as a solution in THF (0.5 mL) to the orange suspension, and the reaction was stirred at r.t. for 3 h. The reaction mixture was quenched by addition of water (10 mL) and extracted with EtOAc (3 × 10 mL). The combined organic phases were washed with water (10 mL), treated with aqueous HCl (0.1 M, 10 mL), washed with brine, dried over MgSO_4_, filtered, and concentrated *in vacuo*. The crude product was purified by FCC on silica gel (Hexanes/EtOAc = 95:5; then Hexanes/EtOAc/AcOH = 95:4:1) to give **55** as a colorless oil (15.8 mg, 25.4 μmol, 25%, 84:16 *Z*/*E*). ^**1**^ **H NMR** (500 MHz, CDCl_3_) (*Z*)-**55**: δ_H_ = 5.12 (d, *J* = 55.9 Hz, 1H), 4.85 (td, *J* = 6.6, 2.3 Hz, 1H), 4.68 (q, *J* = 7.3 Hz, 1H), 4.32 (dd, *J* = 6.5, 1.6 Hz, 1H), 4.12 (q, *J* = 7.0 Hz, 1H), 2.73 (ddd, *J* = 20.9, 9.5, 6.2 Hz, 1H), 2.41–2.34 (m, 4H), 2.21–2.13 (m, 5H), 1.85–1.75 (m, 3H), 1.75–1.71 (m, 2H), 1.13 (t, *J* = 7.4 Hz, 3H), 1.03 (d, *J* = 6.8 Hz, 3H), 0.90 (s, 9H), 0.87 (s, 9H), 0.13 (s, 3H), 0.10 (s, 3H), 0.08 (s, 3H), 0.07 (s, 3H). ^**13**^ **C NMR** (125 MHz, CDCl_3_) (*Z*)-**55**: δ_C_ = 178.3, 153.6 (d, ^2^ *J*_CF_ = 15.0 Hz), 103.8 (d, ^3^ *J*_CF_ = 10.2 Hz), 95.0 (d, ^1^ *J*_CF_ = 177.0 Hz), 84.2, 83.3, 83.2, 83.2, 78.3 (d, ^4^ *J*_CF_ = 2.0 Hz), 77.4, 66.4, 54.0 (d, ^2^ *J*_CF_ = 22.6 Hz), 41.5, 40.8 (d, ^4^ *J*_CF_ = 6.7 Hz), 40.2, 33.4, 26.0, 25.8, 24.8 (d, ^5^ *J*_CF_ = 3.7 Hz), 24.5 (d, ^4^ *J*_CF_ = 5.1 Hz), 22.1, 18.4, 18.2, 15.3, 14.5, 12.6, −4.3, −4.6, −4.6, −5.0. **HRMS** (ESI^+^) calcd for C_34_H_57_FNaO_5_Si_2_ [M + Na^+^] 643.3621; found, 643.3617.

#### 4.10.12 5-((3*S*,3a*R*,4*S*,5*R*,6a*S*)-3-Fluoro-5-hydroxy-4-((3*S*,4*S*)-3-hydroxy-4-methylnona-1,6-diynyl)­hexahydro-2*H*-cyclopenta­[*b*]­furan-2-ylidene)­pentanoic Acid (5)

To a stirring solution of **55** (8.00 mg, 12.9 μmol, 1.0 equiv) in THF (0.13 mL) at 0 °C, TBAF (1.0 M in THF, 39.0 μL, 38.7 μmol, 3.0 equiv) was added dropwise. The reaction mixture was warmed to r.t. and stirred for a further 20 h. The reaction was quenched by addition of H2O (1.3 mL), and the aqueous phase was extracted with Et_2_O (3 × 2.6 mL). The combined organic phases were washed with brine, dried over MgSO_4_, filtered, and concentrated *in vacuo*. The crude product was purified by FCC on silica gel (CH_2_Cl_2_/EtOAc/AcOH = 60:39:1 to 40:59:1) to give **5** as a colorless oil (4.60 mg, 11.7 μmol, 91%, 84:16 *Z*/*E*). ^**1**^ **H NMR** (500 MHz, CDCl_3_) (*Z*)-**5**: δ_H_ = 5.13 (d, *J* = 55.1 Hz, 1H), 4.90 (td, *J* = 6.1, 1.6 Hz, 1H), 4.74 (q, *J* = 7.6 Hz, 1H), 4.39 (dd, *J* = 6.5, 1.7 Hz, 1H), 4.22–4.17 (m, 1H), 2.82–2.75 (m, 1H), 2.49–2.42 (m, 2H), 2.38–2.35 (m, 2H), 2.28–2.25 (m, 1H), 2.19–2.09 (m, 4H), 1.93–1.87 (m, 2H), 1.80 (dd, *J* = 13.9, 6.9 Hz, 1H), 1.74–1.66 (m, 1H), 1.13 (t, *J* = 7.5 Hz, 3H), 1.08 (d, *J* = 6.8 Hz, 3H). ^**13**^ **C NMR** (125 MHz, CDCl_3_) (*Z*)-**5**: δ_C_ = 178.2, 153.4 (d, ^2^ *J*_CF_ = 14.8 Hz), 104.8 (d, ^3^ *J*_CF_ = 10.4 Hz), 94.7 (d, ^1^ *J*_CF_ = 177.3 Hz), 85.0, 83.9, 83.8, 82.7, 78.5 (d, ^4^ *J*_CF_ = 1.5 Hz), 77.0, 66.3, 54.4 (d, ^2^ *J*_CF_ = 22.9 Hz), 40.7 (d, ^4^ *J*_CF_ = 7.1 Hz), 40.2, 39.2, 33.1, 24.5 (d, ^5^ *J*_CF_ = 3.7 Hz), 24.2 (d, ^4^ *J*_CF_ = 5.3 Hz), 22.3, 15.4, 14.4, 12.6. **HRMS** (ESI^+^) calcd for C_22_H_29_FNaO_5_ [M + Na^+^] 415.1891; found, 415.1892.

### 4.11 Isolation and Preparation of Human Platelets

Blood was donated by healthy volunteers. Whole blood was then acidified with prewarmed (30 °C) acid citrate dextrose (1:7 *v*/*v*, 85 mM sodium citrate, 71 mM citric acid and 111 mM d-glucose), decanted into 12 × 75 (mm) polystyrene tubes, and centrifuged (180 *x*g, 17 min, RT). The platelet-rich plasma was collected and pooled in a 50 mL Falcon tube and supplemented with 10 μM indomethacin and 0.02 U mL^–1^ apyrase before centrifugation (520 xg, 10 min, RT). Supernatant plasma was aspirated, and the resulting platelet pellet was resuspended in modified-HEPES-Tyrode’s (10 mM HEPES free-acid pH 7.2, 145 mM NaCl, 3 mM KCl, 0.5 mM Na_2_HPO_4_, 1 mM MgSO_4_·7H_2_O, 0.1% glucose, 10 μM indomethacin, and 0.02 U mL-1 apyrase). Platelet concentration was measured (Beckman Coulter Z1 particle counter) and adjusted to 4 × 10^8^ platelets mL^–1^. Platelets were rested for 30 min at 30 °C before use.

### 4.12 Preparation of Reference and Novel Prostacyclin Analogues

Stock solutions of between 10 and 15 mM of the novel prostacyclin analogues were prepared in dimethyl sulfoxide (DMSO). Beraprost sodium stock solutions of 10 mM were prepared in DMSO. Iloprost and cicaprost were commercially formulated as solutions in methyl acetate. All compounds were subdiluted in DMSO to 500x the indicated concentrations.

### 4.13 Dual Color Flow Cytometry Analyses of Platelet Activation in the Presence of Prostacyclin Analogues

Platelet activation was monitored by measuring the activation of integrin α_IIb_β_3_ and expression of P-selectin (CD62P) on the platelet surface using FITC-PAC-1 and PE-CD62P (AK-4), respectively. Washed platelets were diluted to 2 × 10^7^ mL^–1^ in modified-HEPES-Tyrode’s before use. Platelets were dispensed into a 96-well plate for preincubation (5 min, RT) with prostacyclin analogues at the indicated concentrations. Platelets were then transferred to a 96-well plate for stimulation (10 min, RT) with the selective PAR1 agonist, SFLLRN-NH2 (5 μM) in the presence of FITC-PAC-1 and PE-CD62P (AK-4). Samples were fixed with 1% paraformaldehyde (30 min, RT) before analysis with a BD Accuri C6 Plus flow cytometer. The platelet population was gated based on forward and side-scatter profiles, and 10,000 platelet events per sample were captured. Median fluorescence values of the gated platelet populations were obtained using the BD Accuri C6 Plus software.

### 4.14 Data Analysis

Median fluorescence values were expressed as a percentage of an SFLLRN (5 μM) maximal activation control. Percentage fluorescence values (*y*-axis) were plotted against the logarithm (log) of the indicated compound concentration (*x*-axis) using GraphPad Prism 7.0. Curves were fitted to the data using a four-parameter logistic equation to obtain logIC_50_ values using GraphPad Prism 7.0. The bottom of the curve was constrained to 0 and the top to 100. pIC_50_ values indicate the -logIC_50_, where the IC_50_ represents the compound concentration required for 50% inhibition. Mean pIC_50_ values ± SEM were calculated by averaging the pIC_50_ values obtained from individual concentration-inhibition curves.

Estimated potencies (Table S1) of the novel prostacyclin analogues as a pure *Z* isomer were obtained by multiplying the molar compound concentration by the ratio of the *Z* isomer (e.g., for Compound **2a**, x 0.82) and replotting the percentage fluorescence values (*y*-axis) against the recalculated logarithmic concentration of the indicated concentration (*x*-axis) using GraphPad Prism 7.0. This estimation assumes that the *E* isomer has no affinity or efficacy for the IP receptor.

### 4.15 PRESTO-Tango Assay

#### 4.15.1 Plasmid Isolation

Tango-ized constructs to express prostanoid receptors PTGDR (ID #: 66482), GPR44 (ID #: 66359), PTGER1 (ID #: 66483), PTGER2 (ID #: 66484), PTGER3 (ID #: 66485), PTGER4 (ID #: 66486), PTGFR (ID #: 66487), TBXA2R (ID #: 66517), and PTGIR (ID #: 66488) in HTLA cell line (HEK293 derived with stable integration of β-arrestin2-TEV fusion and tTA-dependent luciferase reporter) were purchased from addgene (https://www.addgene.org/). Stab cultures were grown in colonies on agar plates as instructed by the company. Plasmids were isolated using a Quick-start protocol High-speed plasmid Maxi kits (ref#12662 QIAGEN). Following isolation, concentration was measured, and plasmid samples were sent to SourceBioscience (https://www.sourcebioscience.com/) for sequencing. Constructs were assembled to confirm their sequence.

#### 4.15.2 Cell Culture

HTLA cells were grown in Dulbecco’s Modified Eagle’s Medium [High glucose (+) 4500 mg/L glucose, (−) l-glutamine, sodium pyruvate] supplemented with 10% fetal bovine serum 2 mM l-glutamine, 50 U/ml penicillin, 50 μg/mL streptomycin, 2 μg/mL Puromycin, and 100 μg/mL hygromycin B. Cells were maintained in an incubator at ∼37 °C, 95% air/5% CO_2_.

#### 4.15.3 Transient Transfection

On day one, HTLA cells were split and plated at 8.8 × 10^6^ cells in a 100 mm cell culture dish overnight; the next day, cells were transiently transfected by using Polyethylenimine. Plasmid: SERUM FREE MEDIA: PEI (1:100:3) were combined and incubated at room temperature for 15–20 min and added in a dropwise manner to the dish.

#### 4.15.4 Surface Expression of Prostanoid Receptors

Transfected cells were checked for surface expression of receptors via flow cytometry. Media was aspirated, and cells were detached from 100 mm dishes with 5 mM ethylenediaminetetraacetic acid (EDTA) dissolved in PBS (150 mM NaCl, 2.6 mM KCl, 10 mM Na_2_HPO_4_, and 1.9 mM KH_2_PO_4_, pH 7.4). The detached cells (1000 g for 3 min) were centrifuged, and the supernatant was discarded. The pellet was resuspended in PBS, mixed, and recentrifuged, and 100 μL of it was incubated with an anti-FLAG–FITC conjugate for 30 min at 37 °C. The mixture was diluted with 200 μL of PBS, cells were fixed with 1% PFA, and surface expression was monitored through a BD Accuri C6 Plus cytometer.

#### 4.15.5 PRESTO-Tango Assay

Transiently transfected HTLA cells on day 3 from 100 mm dishes at 3.3 × 10^4^ cells per well in 100 μl of medium were transferred to a polystyrene white 96-well flat-bottom TC-treated cell culture microplate with a lid coated with poly- *d* -lysine (PDL). On day 4, ligands (3.5X) were prepared in assay buffer [Hank’s buffered saline solution (HBSS) (1X HBSS supplemented with 20 mM HEPES)] and incubated overnight within a cell-containing 96-well plate. Finally, on day 5, media were removed, and cells were incubated for 20 min with a Bright-Glo luciferase assay system diluted 10-fold into assay buffer. Luminescence was measured using a Tecan Infinite M200PRO. Data were transferred into Excel and normalized to the maximum response of the reference compound for each receptor, and curves were fitted into GraphPad Prism utilizing a nonlinear regression, log­(agonist) vs response, and slope = 1. The area under the curve for IP receptors was compared by one-way ANOVA, followed by Dunnett’s multiple comparisons test using GraphPad Prism version 7.00.

## Supplementary Material





## Notes

Experimental procedures, characterization, and spectral data for all compounds (PDF)
Molecular formula strings table of the compounds (CSV)

## Abbreviations

BT: benzothiazole
chem: chemical
DMS: dimethyl sulfide
DP: D-type prostanoid receptor
dr: diastereomeric ratio
EP: E-type prostanoid receptor
FACS: Fluorescence-Activated Cell Sorting
FP: F-type prostanoid receptor
HMDS: hexamethyldisilazane
HOESY: Hetero-nuclear Overhauser Enhancement Spectroscopy
IP: I-type prostanoid receptor
met: metabolic
NFSI: *N*-fluorobenzenesulfonimide
NMI: *N*-methylimidazole
PAH: pulmonary arterial hypertension
PAP: pulmonary arterial pressure
PAR: protease-activated receptor
PGF_2α_: Prostaglandin F2α
PRESTO: Predicted Receptor Expression and Signaling Technology platform
PT: 1-phenyl-1*H*-tetrazole
PVR: pulmonary vascular resistance
SEM: standard error of the mean
TEMPO: 2,2,6,6-tetramethylpiperidine 1-oxyl
Tf: triflate
TP: thromboxane prostanoid receptor
