# Supplementary material for: Synthesis, Modeling, and Biological Properties of Fluoroprostacyclin Analogues: Potent Agonists for Prostanoid Receptors
Source: J Med Chem. 2026 Jul 25;69(15):18204–25. doi: 10.1021/acs.jmedchem.6c00630 (PMC13492276; doi:10.1021/acs.jmedchem.6c00630)
Supplement: Supplementary file 1 [file jm6c00630_si_001.pdf]

Supporting Information

Synthesis, Modelling and Biological Properties of Fluoroprostacyclin Analogues: Potent Agonists for Prostanoid Receptors

Changcheng Jing,† Isabel Perez-Powell,† Hannah Baars,† Shahida Mallah,‡ , Justin S. Trory,‡ Robin A. Corey,◊ Stuart J. Mundell,‡ Xin Xu,◊ Jay Tromans,† Samantha F. Moore,‡ Christopher J. Arthur,† Ingeborg Hers,*‡ and Varinder K. Aggarwal*†

†School of Chemistry, University of Bristol, Cantock's Close, Bristol BS8 1TS, United Kingdom

‡Bristol Medical School, University of Bristol, University Walk, Biomedical Sciences Building, Bristol BS8 1TD, United Kingdom

◊School of Psychology & Neuroscience, University of Bristol, University Walk, Biomedical Sciences Building, Bristol BS8 1TD, United Kingdom

Corresponding Author Emails:

Varinder K. Aggarwal - V.Aggarwal@bristol.ac.uk

Ingeborg Hers – I.Hers@bristol.ac.uk

Table of Contents

Page

General Information……………………………………………………………....................S2

Supplementary Information for Biological Properties Studies……..................S4

Stability Studies for Compound 9…........…........….........…......……..................S14

Modelling Studies…........…........…........…...S23

Experimental Procedures…………………...……………….............................S28

Assignment of the Olefin Geometry……………………………………….................S100

Determination of Stereochemistry of Monofluorinated Lactone......................S102

Notes and References……………………………………………………................S103

NMR Spectra for the Products…………………………………...............................S106

HPLC Traces for Compounds 5, 7, 8, and 9……………………………………S196

General Information

Solvents and Reagents. Generally, reactions were carried out using reagent grade solvents and without air excluded. Reactions requiring anhydrous solvents and an inert atmosphere were carried out under a nitrogen atmosphere using standard manifold techniques. All solvents were commercially supplied or obtained from a purification column composed of activated alumina.[1] All chemicals were purchased from Acros, Aldrich, Alfa Aesar, Fluka, Lancaster or Merck and used as received unless otherwise stated. Propanal was distilled prior to use. 2,6-Lutidine was treated with anhydrous $AlCl_3$ and distilled under reduced pressure prior to use. Triethylamine and trimethylsilyl chloride were distilled over CaH_2 under reduced pressure prior to use. *n*-BuLi and *tert*-BuLi were purchased from Acros and were titrated against N-benzylbenzamide using the procedure of Chong *et al.*[2] LiHMDS solution was prepared freshly from distilled HMDS and *n*-BuLi. O_3-O_2(g) was generated by Ozone Generator C-Lasky from AirTree Ozone Technology Co., Ltd.

Chromatography. Flash column chromatography (FCC) was carried out using Sigma-Aldrich silica gel (60 Å, 230 – 400 mesh, 40 – 63 μm) or using Biotage Isolera™ One with Biotage® SNAP/SNAP Ultra Cartridges. All reactions were followed by thin-layer chromatography (TLC) when practical, using Merck Kieselgel 60 F254 fluorescent treated silica gel, which was visualized under UV light or by staining with aqueous basic potassium permanganate or an ethanolic and acidic solution of *p*-anisaldehyde. Compound purity was determined by High Performance Liquid Chromatography (HPLC) performed on a reverse phase HPLC Agilent 1260 Infinity II HPLC system, using a Waters Spherisorb S5 ODS2 column (5 μm diameter, 4.6 × 250 mm length) with detection carried out at 214 nm. H2O:MeCN (with 0.1% formic acid) mixtures were used as eluents for all analysis. Analysis was performed at room temperature using the following method: 1 mL/min flow rate, 20 min run, MeCN:H2O (0.1% Formic Acid) gradient [time]: 30:70 [0 min], 30:70 [1 min], 95:5 [16 min], 95:5 [17 min], 30:7 [18 min].

Spectroscopy. ^{1}H, ^{13}C NMR and ^{19}F NMR spectra were recorded on Jeol ECS 400, Bruker Nano 400, Varian VNMRS500 and Bruker Avance III HD 500 Cryo spectrometers. Chemical shift (*δ* value) are referenced relative to the residual protiated solvent and are reported in parts per million (ppm), and coupling constants (*J* value) are given in Hertz (Hz). The ^{1}H NMR spectra are reported as follows: chemical shift, multiplicity (s = singlet, br.s = broad singlet, d = doublet, t = triplet, q = quartet, quin = quintet, sex = sextet, h = heptet, m = multiplet, dd = doublet of doublets, etc.), coupling constants and assignment. NMR assignments are made

according to spin systems, using two-dimensional NMR spectroscopy (COSY, HSQC and HMBC etc.) to assist the assignment. High resolution mass spectra (HRMS) were recorded on a VG Analytical Autospec spectrometer by Electron Ionisation (EI) or Chemical Ionisation (CI) or on a on a Bruker micrOTOF instrument by Electrospray Ionisation (ESI). Infra-red (IR) spectra were recorded on a Perkin Elmer Spectrum One FT-IR spectrometer as a thin film. Only selected absorption maxima (ν_{max}) are reported in wavenumbers (cm^{-1}). Melting points were recorded in degrees Celsius (°C), using a Kofler hot-stage microscope apparatus and are reported uncorrected. Optical rotation ($[\alpha]_D^T$) was measured on a Bellingham and Stanley Ltd. ADP220 polarimeter and is quoted in (° mL)(g dm)$^{-1}$. Chiral supercritical fluid chromatography (SFC) was performed using a Daicel Chiralpak® IA column (4.6 × 250 mm × 5µm) using a Waters TharSFC system and monitored by Diode Array Detector (DAD). Chiral HPLC was performed on a HP agilent 1100 with a Daicel Chiralpak® OD-H column (4.6 × 250 mm × 5 µm) fitted with the respective guard (4 × 10 mm) and monitored by Diode Array Detector (DAD).

Naming of Compounds. Compound names are generated by ChemBioDraw 11.0 software (PerkinElmer), following IUPAC nomenclature.

Purity of Compounds. All compounds subjected to *in vitro* and *ex vivo* assays are >95% pure, determined by HPLC analysis.

Supplementary Information for Biological Properties Studies

Materials

TRAP-6 amide trifluoroacetate salt (H-Ser-Phe-Leu-Leu-Arg-Asn(SFLLRN)-NH_2) (#H-2936, CAS № 141923-40-2) a synthetic selective protease-activated receptor 1 (PAR1) agonist was from Bachem (Bubendrof, Switzerland). Iloprost (#18215, CAS № 78919-13-8) was from Cayman CHEMICAL (Cambridge Bioscience, Cambridge, UK). Cicaprost (#SC-358799, CAS № 94079-80-8) was purchased from Santa Cruz Biotechnology (Insight Biotechnology, Middlesex, UK). Beraprost sodium (#4180, CAS № 88475-69-8) was from Bio-Techne Ltd (Abingdon, UK). Fluorescein isothiocyanate (FITC) mouse anti-human PAC-1 (#340507) and phycoerythrin (PE) mouse anti-human CD62P Clone AK-4 (#555524) were from BD Biosciences (Wokingham, UK). All other reagents were from Sigma-Aldrich (Poole, UK) unless otherwise stated.

Methods

Isolation and preparation of human platelets

Blood was donated by healthy volunteers after an approval from local research ethics committee at the University of Bristol. Volunteers gave full informed consent in accordance with the declaration of Helsinki and confirmed that they had not taken any medication two weeks prior to donation. Venous blood was drawn from the antecubital region using the BD Vacutainer® Safety-Lok™ Blood Collection Set (21G x ¾" x 12, #367286) into a syringe containing 4 % trisodium citrate in (1:10 *v*/*v*). Blood was then acidified with pre-warmed (30 °C) acid citrate dextrose (1:7 *v*/*v*, 85 mM sodium citrate, 71 mM citric acid and 111 mM d-glucose), decanted into 12 x 75 (mm) polystyrene tubes and centrifuged (180 *x*g, 17 min, RT) using a swing out rotor in a Heraeus® Megafuge® 11 (ThermoFisher Scientific). The upper layer of the spun blood (platelet-rich plasma, PRP) was collected and pooled in a 50 mL falcon tube and supplemented with 10 µM indomethacin and 0.02 U mL^{-1} apyrase (Grade VII) before centrifugation (520 xg, 10 min, RT) using a swing out rotor in a Heraeus® Megafuge® 11 (ThermoFisher Scientific). Supernatant plasma was aspirated, and the resulting platelet pellet resuspended in modified-HEPES-Tyrode's (10 mM HEPES free-acid pH 7.2, 145 mM NaCl, 3 mM KCl, 0.5 mM Na_2HPO_4, 1 mM $MgSO_4.7H_2O$, 0.1% glucose, 10 µM indomethacin and 0.02 U mL-1 apyrase). Platelet density was measured (Beckman Coulter Z1 particle counter) and adjusted to $4x10^8$ platelets mL^{-1}. Platelets were rested for 30 min at 30 °C before use.

Preparation of reference and novel prostacyclin analogues

Stock solutions of between 10 – 15 mM of the novel prostacyclin analogues were prepared in dimethyl sulfoxide (DMSO). Beraprost sodium stock solutions of 10 mM were prepared in DMSO. Iloprost and cicaprost were commercially formulated as solutions in methyl acetate. All compounds were sub-diluted in DMSO to 500x the indicated concentrations.

Dual colour flow cytometry analyses of platelet activation in the presence of prostacyclin analogues.

Platelet activation was monitored by measuring the activation of the fibrinogen receptor integrin $\alpha_{IIb}\beta_3$ and the presence of the α-granule marker; P-selectin (CD62P) on the platelet surface using FITC-PAC-1 and PE-CD62P (AK-4) respectively. Washed platelets were diluted to 2 x 10^7 mL^{-1} in modified-HEPES-Tyrode's before use. Platelets were dispensed into a 96-well plate for pre-incubation (5 min, RT) with prostacyclin analogues at the indicated concentrations. Platelets were then transferred to a 96-well plate for stimulation (10 min, RT) with the selective protease-activated receptor 1 (PAR1) agonist; SFLLRN-NH2 (5 μM) in the presence of FITC-PAC-1 and PE-CD62P (AK-4). Samples were fixed with 1% of paraformaldehyde (30 min, RT) before analysis with a BD Accuri™ C6 Plus flow cytometer (BD Biosciences, Oxford, UK). The platelet population was gated based on forward and side-scatter profiles and 10,000 platelet events per sample captured. Median fluorescence values (FL-1: FITC, FL-2: PE) of the gated platelet populations were obtained using the BD Accuri™ C6 Plus software.

Data analysis

Median fluorescence values (FL-1: FITC, FL-2: PE) were expressed as a percentage of the median fluorescence values (FL-1: FITC, FL-2: PE) obtained in the platelet sample treated with SFLLRN (5 μM) in the absence of prostacyclin analogue (maximal activation). Percentage fluorescence values (y-axis) were plotted against the logarithmic (log) concentration of the indicated concentration (x-axis) using GraphPad Prism 7.0. Curves were fitted to the data using a four-parameter logistic equation (Equation 1) to obtain $logIC_{50}$ values using GraphPad Prism 7.0. The bottom of the curve was constrained to 0 and the top to 100. pIC_{50} values indicate the $-logIC_{50}$, where the IC_{50} represents the compound concentration required for 50% inhibition. Mean pIC_{50} values ± s.e.m were calculated by averaging the pIC_{50} values obtained from individual concentration-inhibition curves.

Equation 1: Y=Bottom + (Top-Bottom)/(1+10^((LogIC_{50}-X)*HillSlope))

Estimated potencies (Table S1) of the novel prostacyclin analogues as a pure *Z* isomer were obtained by multiplying the molar compound concentration by the ratio of the *Z* isomer (e.g. for Compound **2a**, x 0.82) and replotting the percentage fluorescence values (y-axis) against the recalculated logarithmic concentration of the indicated concentration (x-axis) using GraphPad Prism 7.0. This estimation assumes that the *E* isomer has no affinity or efficacy for the IP receptor.

PRESTO-Tango assay;

Plasmid isolation

Tango-ized constructs to express prostanoid receptors PTDGR (ID #: 66482), GPR44 (ID #: 66359),PTGER1 (ID #: 66483), PTGER2 (ID #: 66484), PTGER3 (ID #: 66485), PTGER4 (ID #: 66486), PTGFR(ID #: 66487), TBAX2R(ID #: 66517), and PTGIR (ID #: 66488) in HTLA cell LINE (HEK293 derived with stable integration of β-arrestin2-TEV fusion and tTa-dependent luciferase reporter) were purchased from addgene (https://www.addgene.org/). Stab cultures were grown in colonies in agar plates as instructed by the company. Plasmids were isolated by using a Quick-start protocol High-speed plasmid Maxi kits (Ref#12662 QIAGEN). Following isolation, concentration was measured, and plasmid samples were sent to SourceBioscience (https://www.sourcebioscience.com/) for sequencing. Constructs were assembled to confirm their sequence.

Cell culture

HTLA cells (HEK293 derived with stable integration of β-arrestin2-TEV fusion and tTa-dependent luciferase reporter) were grown in Dulbecco's Modified Eagle's Medium (DMEM) (High glucose (+) 4500 mg/L glucose, (-) L-glutamine, sodium pyruvate, 0.1 µm sterile filtered,D5671, SIGMA-ALDRICH) supplemented with 10% foetal bovine serum (FBS) (REF#10270-106, Gibco) 2mM L-glutamate (REF# 2503-081, Gibco), 50 U/ml penicillin and 50 µg/ml streptomycin (Cat#1670049, ThermoFisher scientific), 2µg/ml Puromycin, 100µg/ml hygromycin B (REF# 10687010, Invitrogen). Cells were maintained in incubator at ~37 °C, 95% O_2 / 5% CO_2.

Transient transfection

On day one HTLA cells were split and, plated 8.8 x10^6 cells in 100mm cell culture dish overnight, next day cells were transiently transfected by using Polyethylenimine (PEI) (Cat# 23966. Polysciences, USA). Briefly, plasmid: SERUM FREE MEDIA: PEI (1:100:3) were combined and incubated at room temperature for 15-20 min and added in drop-wise manner to dish.

Surface expression of Prostanoid receptors

Transfected cells were also checked for surface expression of receptors by using flowcytometry. Media was aspirated, cells were detached from 100mm dishes with 5mM ethylenediaminetetraacetic acid (EDTA) dissolved in PBS (150 mM NaCl, 2.6 mM KCl, 10 mM Na_2PO_4, and 1.9 mM KH_2PO_4, pH 7.4). Centrifuged (1000 g for 3 minutes) detached cells and discarded supernant. Resuspended pellet in PBS, mixed and recentrifuged. Pellet was resuspended in 1x PBS and 100 µl was incubated with anti-flag FITC conjugate (F-4049, M2 Sigma) for 30 minutes at 37°C. Diluted mixture with 200 µl of PBS, fixed cells with 1 % PFA and surface expression was monitored through BD Accuri C6 Plus cytometer.

PRESTO-Tango assay

PRESTO-tango assay is described in (Kroeze *et al.*, 2015) for screening of ligands and it is G-protein independent assay. Transiently transfected HTLA cells on day 3 from 100 mm dishes 3.3 x 10^4 cells per well in 100 ul of medium were transferred to polystyrene white 96 well flat bottom TC-treated cell culture microplate with lid (REF#353377, Corning Falcon) coated with poly-D-lysine (PDL) (LOT# SLBW8246, Sigma-Aldrich). On day 4 ligands (3.5X) were prepared in assay buffer (Hank's buffered saline solution (HBSS) (Cat# BE10-527F, LONZA) (1X HBSS supplemented with 20 mM HEPES (H0887, PH 7.0-7.6, sterile, filtered Sigma)) and incubated overnight within cell containing 96 well plate. Finally, on day 5 media was removed and cells were incubated 20 minutes with Bright-Glo™ luciferase assay system (Cat# E2610, Promega corporation) diluted 10-fold into assay buffer. Luminescence was measured through Tecan Infinite M200PRO. Data was acquired into excel and normalised to the maximum response of reference compound for each receptor and curves were fitted into GraphPad Prism by using a non-linear regression, log(agonist) *vs*. response and slope=1. Area under curve for IP receptors was compared by One-Way ANOVA followed by Dunnett's multiple comparisons test using GraphPad prism version 7.00.

Table S1. Detailed Information of the Estimated Potencies of Fluorinated Prostacyclin Analogues 2-9.

Compound		Integrin $\alpha_{IIb}\beta_3$ activation		P-selectin exposure	
	Z/*E* Ratio	pIC_{50}[a] ± SEM, N = 7	IC_{50}[b] (pM)	pIC_{50}[a] ± SEM, N = 7	IC_{50}[b] (pM)
**2a**	82:18	8.98 ± 0.08	1059	9.14 ± 0.13	729
**2b**	71:29	9.03 ± 0.06	932	9.14 ± 0.07	730
**2c**	14:86	8.89 ± 0.04	1287	9.04 ± 0.09	916
**3**	78:22	10.11 ± 0.16	78	10.11 ± 0.13	77
**4**	89:11	9.78 ± 0.08	166	9.94 ± 0.18	113
**5**	84:16	10.66 ± 0.09	21	10.57 ± 0.04	26
**6**	91:9	9.76 ± 0.07	173	9.92 ± 0.09	119
**7**	91:9	10.59 ± 0.08	25	10.63 ± 0.06	23
**8a**	81:19	10.37 ± 0.13	42	10.41 ± 0.11	38
**8b**	96:4	10.41 ± 0.13	38	10.45 ± 0.12	35
**9**	85:15	11.04 ± 0.04	7	10.95 ± 0.11	9

[a] The inhibitory effect of the reference compounds and prostacyclin analogues 2-9 were assessed as described. Mean pIC_{50} values were calculated by averaging pIC_{50} values (n = 7 ± SEM) obtained from individual concentration-inhibition curves fitted with a four-parameter logistic equation (GraphPad Prism 7.0). [b] The IC_{50} values were recalculated by re-fitting concentration-inhibition curves based on the assumption that the *Z* isomer is the active form of the prostanoid analogue and the *E* isomer is inactive (no binding affinity or efficacy for platelet prostanoid receptors).

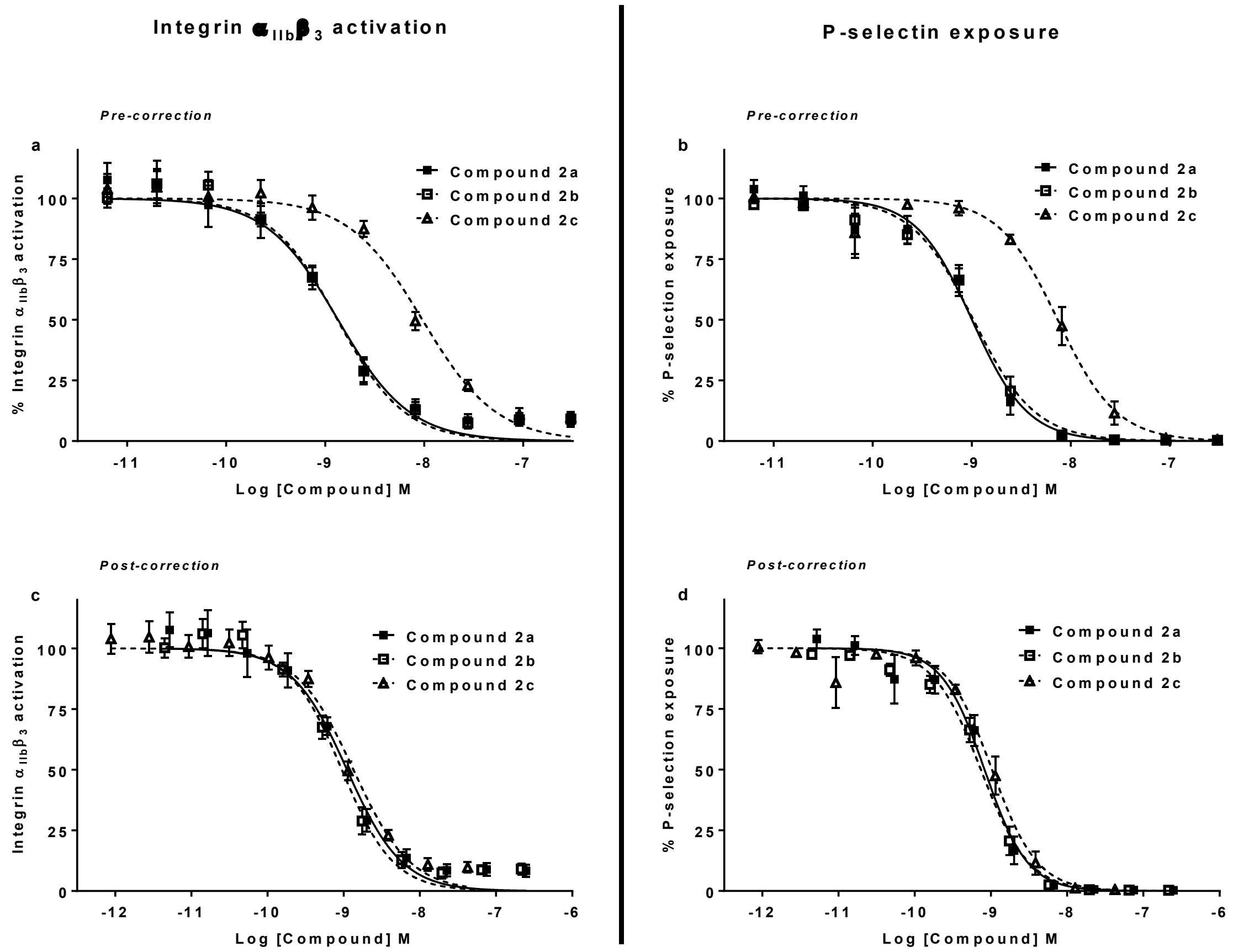

Figure S1. Concentration-Inhibition Curves for the Different Isomer Mixes of Compound 2. Concentration-inhibition curves demonstrating the inhibitory effect of prostacyclin analogue; Compound 2 as isomer mixes with *Z/E* ratios of 82:18 (**2a**), 71:29 (**2b**) and 14:86 (**2c**) on PAR-mediated integrin $\alpha_{IIb}\beta_3$ activation (a) and P-selectin exposure (b). To estimate the potency of Compound **2** as a pure *Z* isomer, molar concentrations were multiplied by the ratio of the *Z* isomer (e.g. for Compound **2a**, x 0.82) and the percentage fluorescence values (y-axis) re-plotted against the corrected concentration (x-axis) This estimation assumes that the *E* isomer has no affinity or efficacy for the IP receptor. Concentration-inhibition curves post-correction for PAR-mediated integrin $\alpha_{IIb}\beta_3$ activation (c) and P-selectin exposure (d) demonstrate that the curves for the isomer mixes converge, indicating that most of the biological effect of Compound **2** is with the *Z* isomer. Data is expressed as a percentage of maximal (mean ± SEM, n = 7).

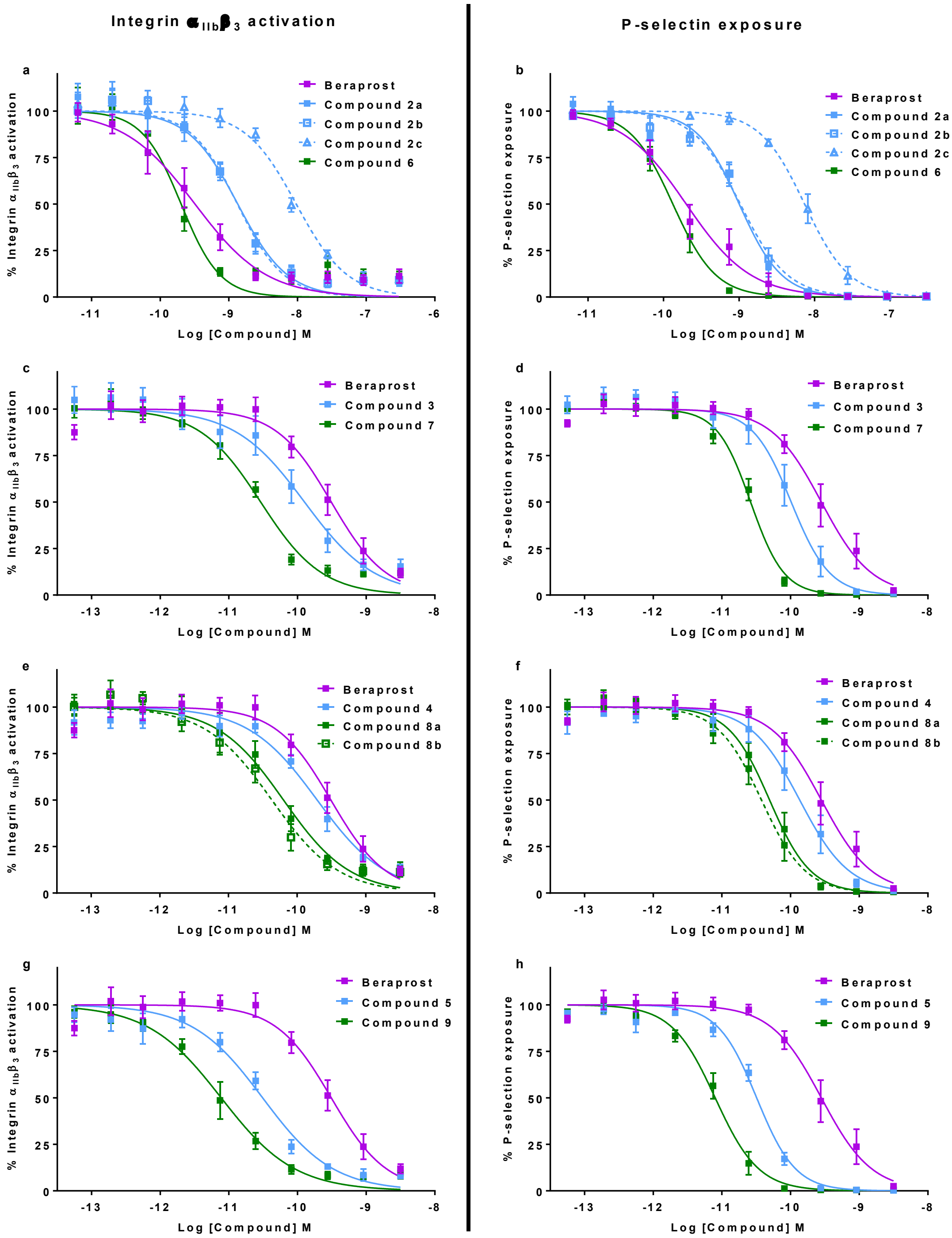

Figure S2. The Antiplatelet Effects of Fluorinated Prostacyclin Analogues 2-9. Concentration-inhibition curves demonstrating the inhibitory effect of the fluorinated prostacyclin analogues **2-9** and control compound; beraprost on PAR-mediated integrin $\alpha_{IIb}\beta_3$ activation (a, c, e, g) and P-selectin exposure (b, d, f, h). Platelets were incubated with compounds (5 min) before stimulation with the PAR-1 agonist SFLLRN (5 µM, 10 min) in the presence of fluorescently conjugated antibodies against the active form of integrin αIIbβ3 (FITC-PAC1) and the platelet α-granule marker; P-selectin (PE-CD62P). Platelets were fixed, and events captured by FACS analysis using a BD Accuri™ C6 Plus flow cytometer. Data is expressed as a percentage of maximal (mean ± SEM, n = 7).

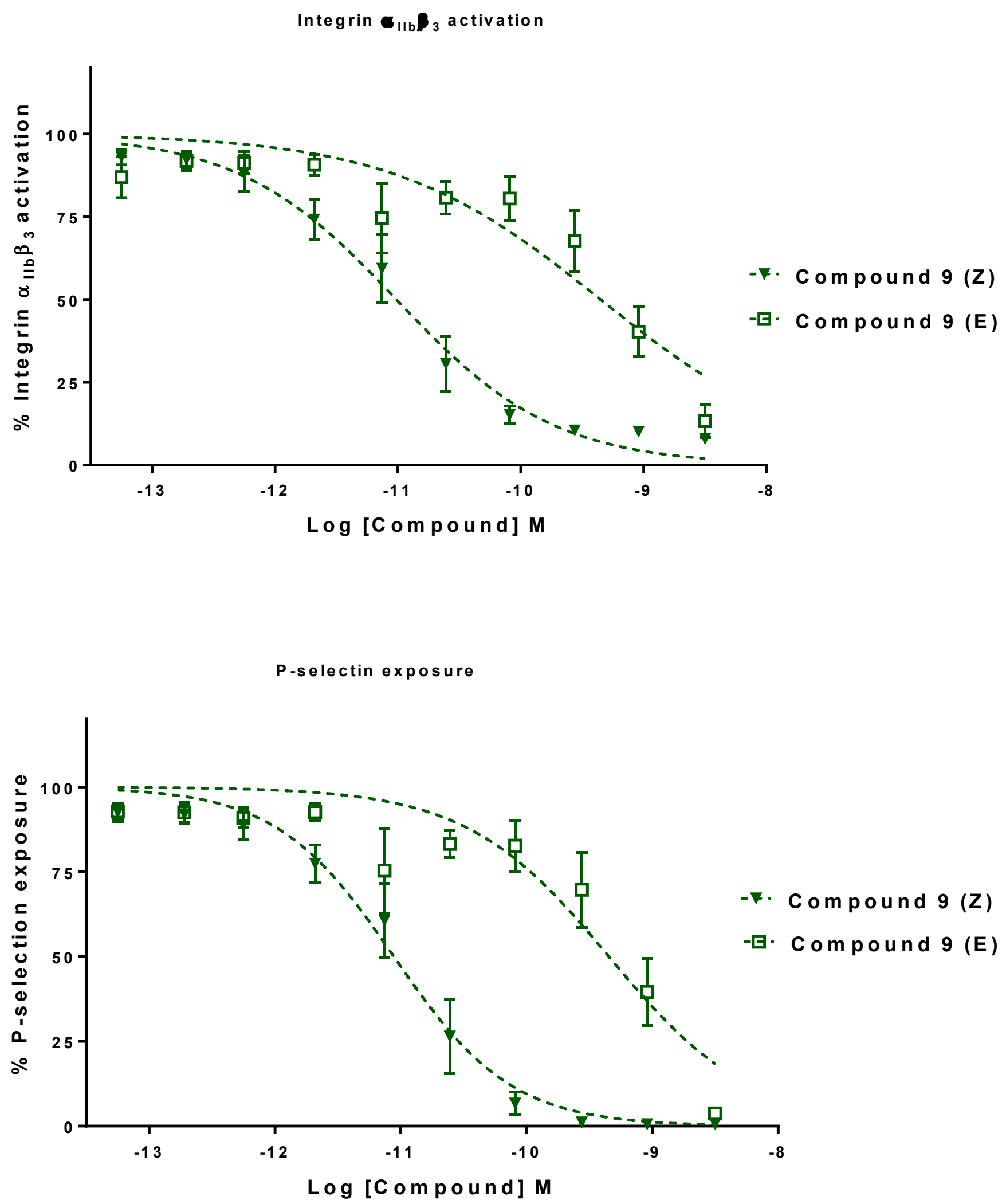

Figure S3. The Antiplatelet Effect of Compound 9 (*Z*) & (*E*) Isomer. The antiplatelet effect of *Z* and *E* isomers of Compound **9** was measured in a concentration-dependent manner. Platelets were incubated for 5 min with increasing concentrations of compounds before stimulation with 5 μM of the PAR1 agonist SFLLRN for 10 min in the presence of fluorescently conjugated antibodies against the active form of integrin $\alpha_{IIb}\beta_3$ (FITC-PAC1) and the platelet α-granule marker P-selectin (PE-CD62P). Platelets were fixed and events captured by FACS analysis using a BD Accuri™ C6 Plus flow cytometer. Data is expressed as a percentage of maximal (mean ± SEM, n = 7).

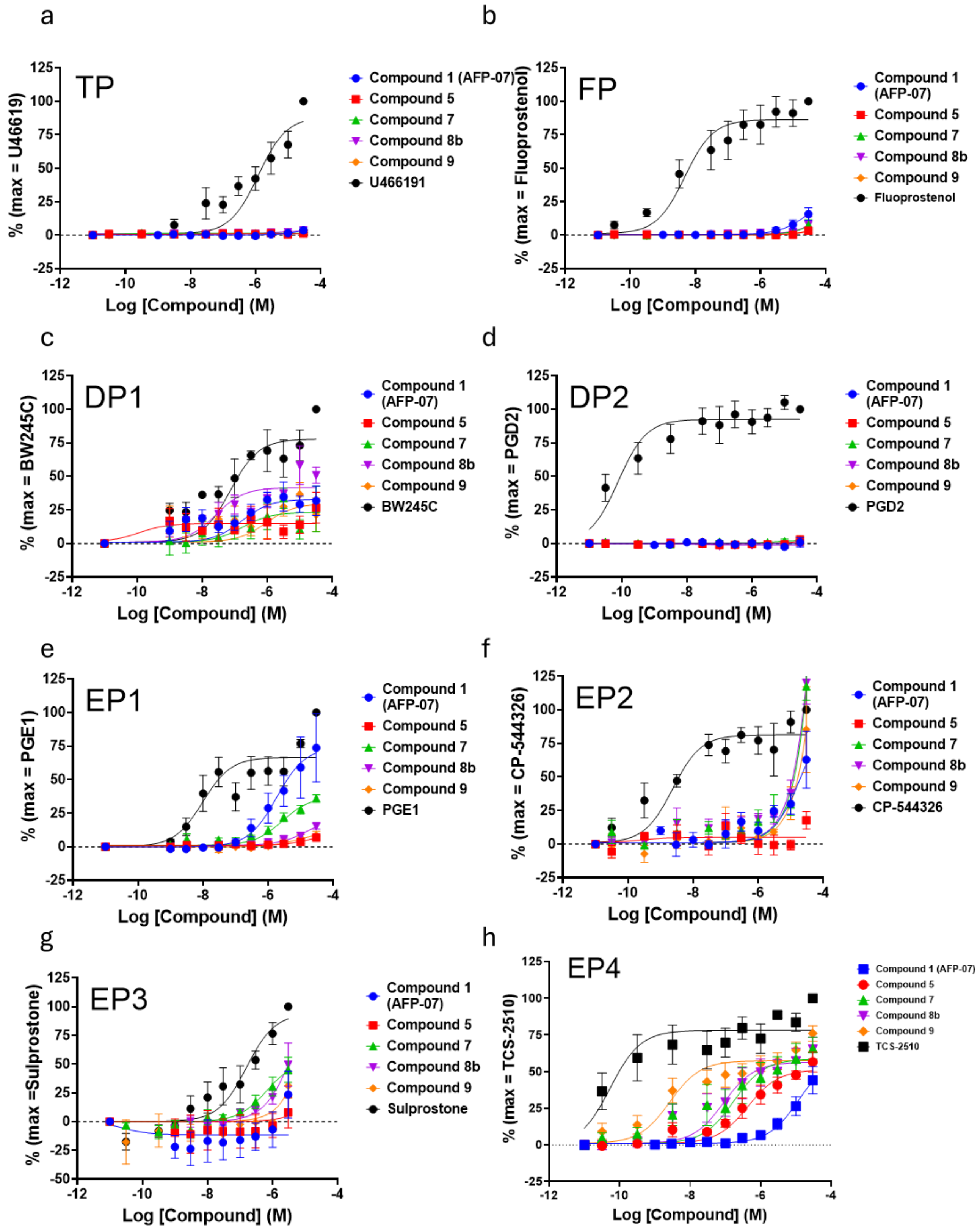

Supplementary Figure S4 – Assessment of prostacyclin analogue ligands selectivity for specific receptors. A series of novel prostacyclin analogues were tested against a variety of prostanoid receptors (a-g) that were transiently expressed in the HTLA cell line using a PRESTO-Tango assay. Data expressed as percent of maximum β-arrestin2 mobilisation induced by a receptor specific reference compound, Error bars represent ± SEM (n=4).

Table S2 – EC_{50} values of novel prostacyclin analogues against the IP receptor as determined by PRESTO-Tango.

Compound	PRESTO-Tango assay		
	Z/E Ratio	EC_{50}[a] (μM)	EC_{50}[b] (μM)
**AFP-07**	-	5.7 ± 0.2	2.0
**Ligand 5**	84:16	6.6 ± 0.6	0.2
**Ligand 7**	91:9	8.0 ± 1.0	0.01
**Ligand 8-2**	96:4	6.4 ± 0.7	0.4
**Ligand 9**	85:15	8.3 ± 1.0	0.006

[a]pEC_{50} the negative logarithm of the EC_{50} calculated for novel prostacyclin analogues in cell line stably expressing ß-arrestin and transiently transfected with IP receptors. [b]EC_{50} the concentration of novel analogues which produces half-maximal response in PRESTO-Tango assay.

Stability Studies for Compound 9

Chemical stability test in pH 7.4 phosphate buffer

Compound **9** (3.76 μmol) was dissolved in 0.1 mL DMSO containing 2.2 μmol of 2,2,2-trifluoroethanol as an internal standard. Then, it was dissolved in 0.4 mL of pH 7.4 phosphate buffer. The solution was stored at 23 °C, and the ratio of compound **9** to 2,2,2-trifluoroethanol was measured by ^{19}F quantitative NMR over a period of 30 days. As shown below, it has been confirmed that the compound **9** exhibited excellent stability in pH 7.4 phosphate buffer.

Compound 9	7 days	10 days	20 days	30 days
**Remaining Ratio**	>99	99	>98	>97

Stability test in human plasma

Sample Preparation

Human plasma (2 mL) containing compound **9** (100 μg) was vortexed for 30 s and incubated at 37 °C. At the appropriate time points, an aliquot of plasma (100 μL) was removed and transferred to a fresh Eppendorf tube. Precipitating reagent (Acetonitrile:MeOH, *v*:*v* 1:1, 300 μL) containing the internal standard (1.11 μg/mL Indomethacin) was added to each tube. The samples were vortexed for 30 s and centrifuged for 10 min (approximately 13,000 rotations per min (RPM)). The supernatant was transferred to a glass vial and stored at 4 °C until analysis.

Liquid Chromatography Mass Spectrometry

Samples were analysed on Thermo Scientific Orbitrap Elite Mass Spectrometer using an Ascentis Express C18 column (50 x 2.1 mm, 2.7μm solid-core particles, Sigma-Aldrich). Compounds were eluted with a linear gradient between 35% acetonitrile (aq, 0.1% formic acid) and 70% acetonitrile (aq, 0.1% formic acid) over 11 min. Samples were ionised using atmospheric pressure chemical ionisation (APCI) using a source voltage of 5kV and a source current of 4 μA. Full scan positive mode spectra (200 to 500 Da) were recorded at 60,000 resolution and selected ion monitoring (SIM) scans at 358 Da (internal standard) and 411 Da (compound **9**) were recorded at 15,000 resolution (Fig. **S4**). Extracted ion chromatogram (EIC) and SIM chromatographic peak areas for the internal standards were determined with the ISIS peak detection algorithm within Thermo Xcalibur. Each sample was analysed in duplicate. Calibration curves (Fig. **S5** and **S6**) were plotted and used to determine the concentration in the test samples at each time point (Fig. **S7**).

:

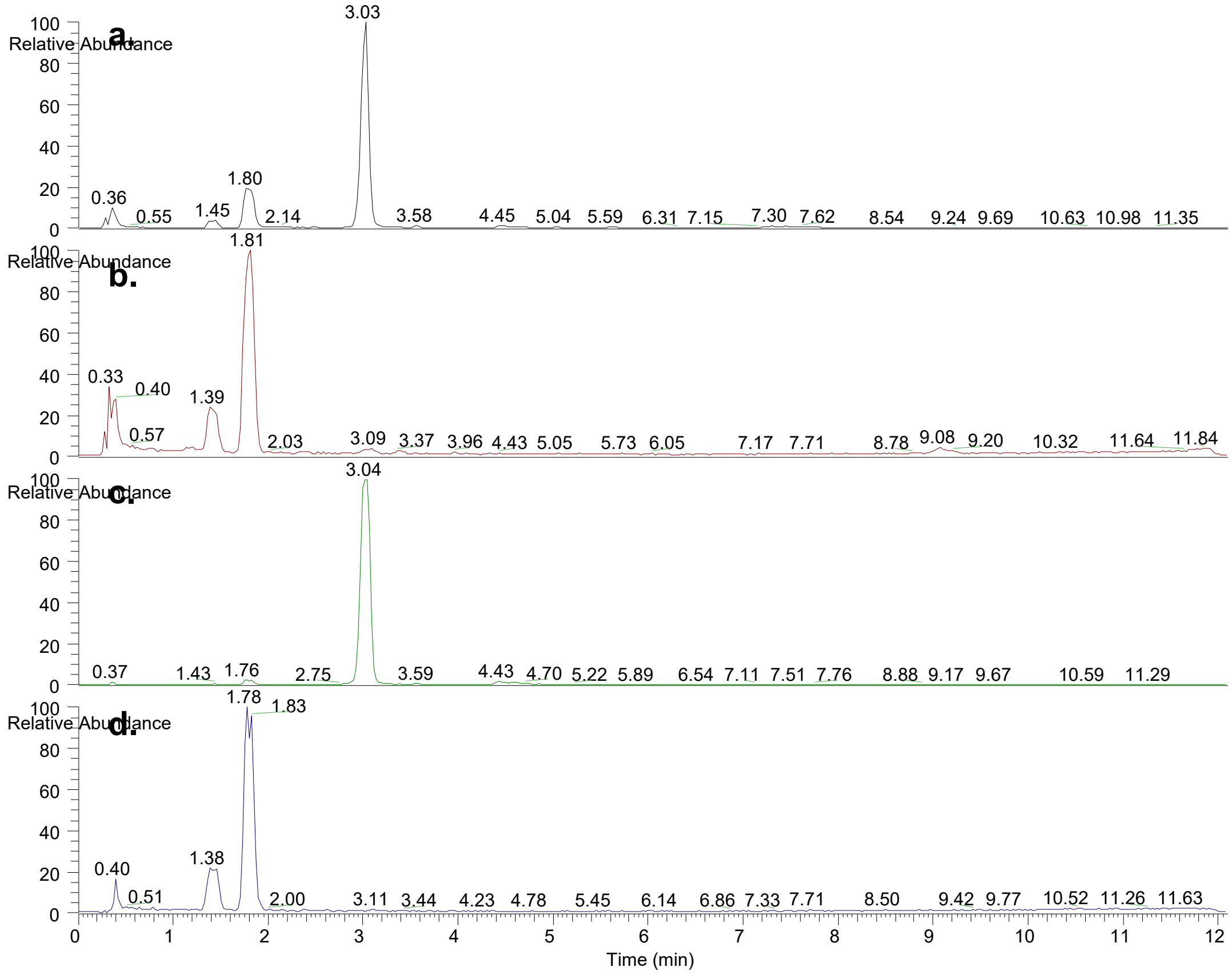

Figure S5. Representative EIC and SIM Chromatograms. Indomethacin internal standard and compound **9** elutes at 3 min and 1.8 min respectively under the conditions used. a. SIM of 358 Da (Internal Standard). b. SIM chromatogram of 411 Da (Compound **9**). c. Extracted ion chromatogram of 358 Da (Internal Standard). d. Extracted ion chromatogram of 411 Da (Compound **9**). The peak at 1.8minutes observed in the 358 Da SIM corresponds to a fragment ion of Compound **9** generated during the ionisation progress.

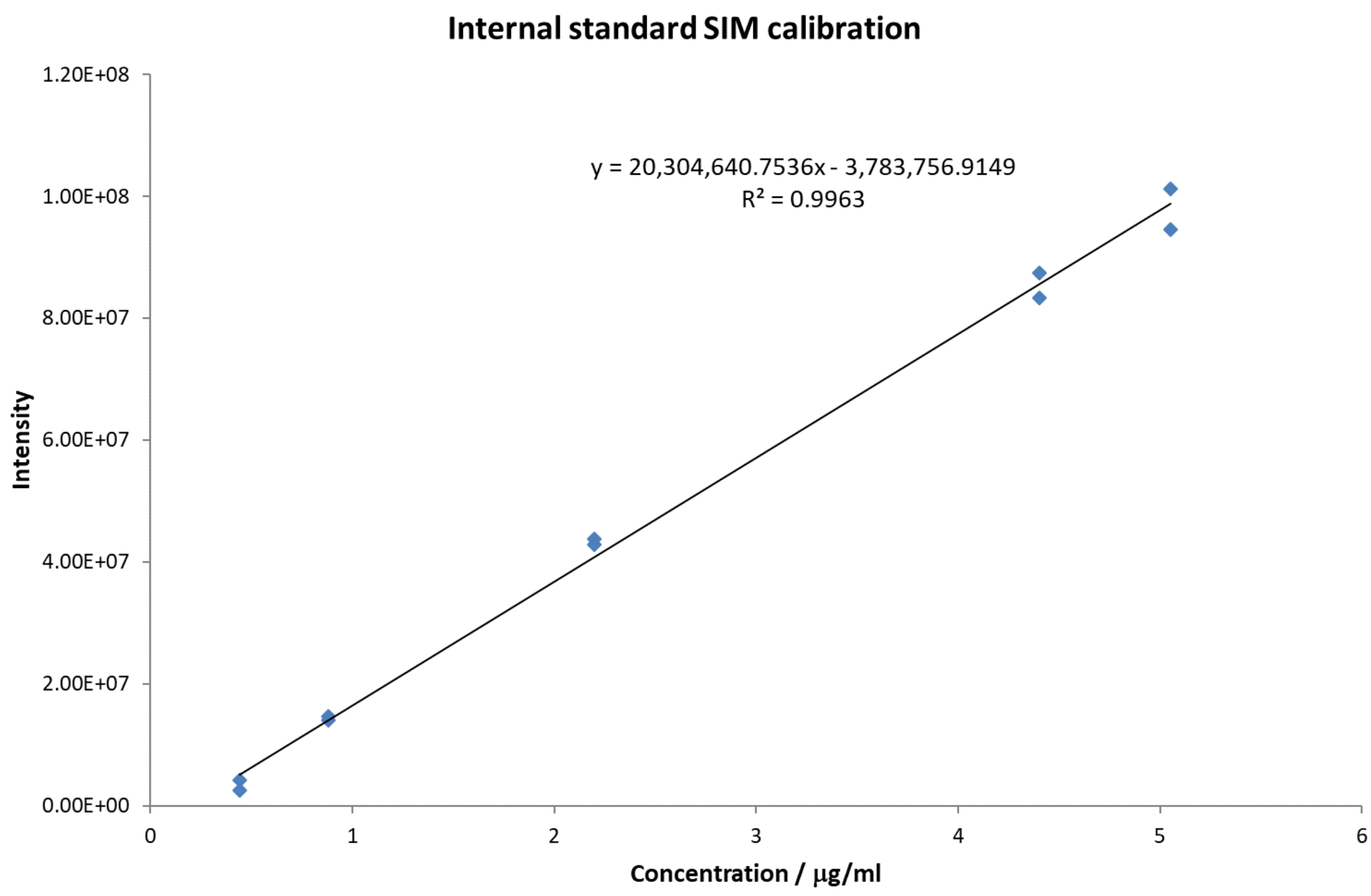

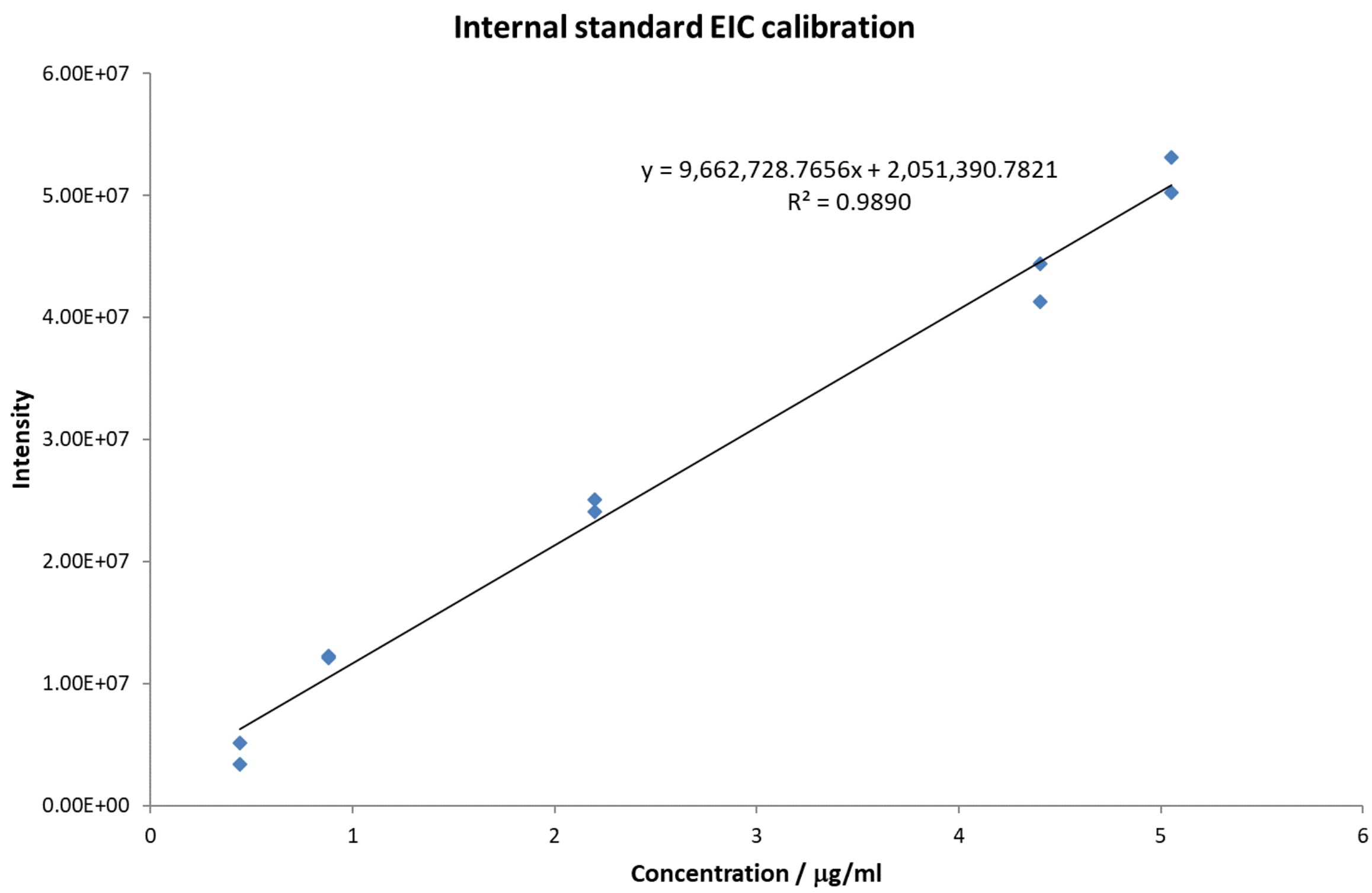

Figure S6. **Calibration Curve of Internal Standard from Selected Ion Monitoring (SIM) and Extracted Ion Chromatogram (EIC) Observed Peak Area**.

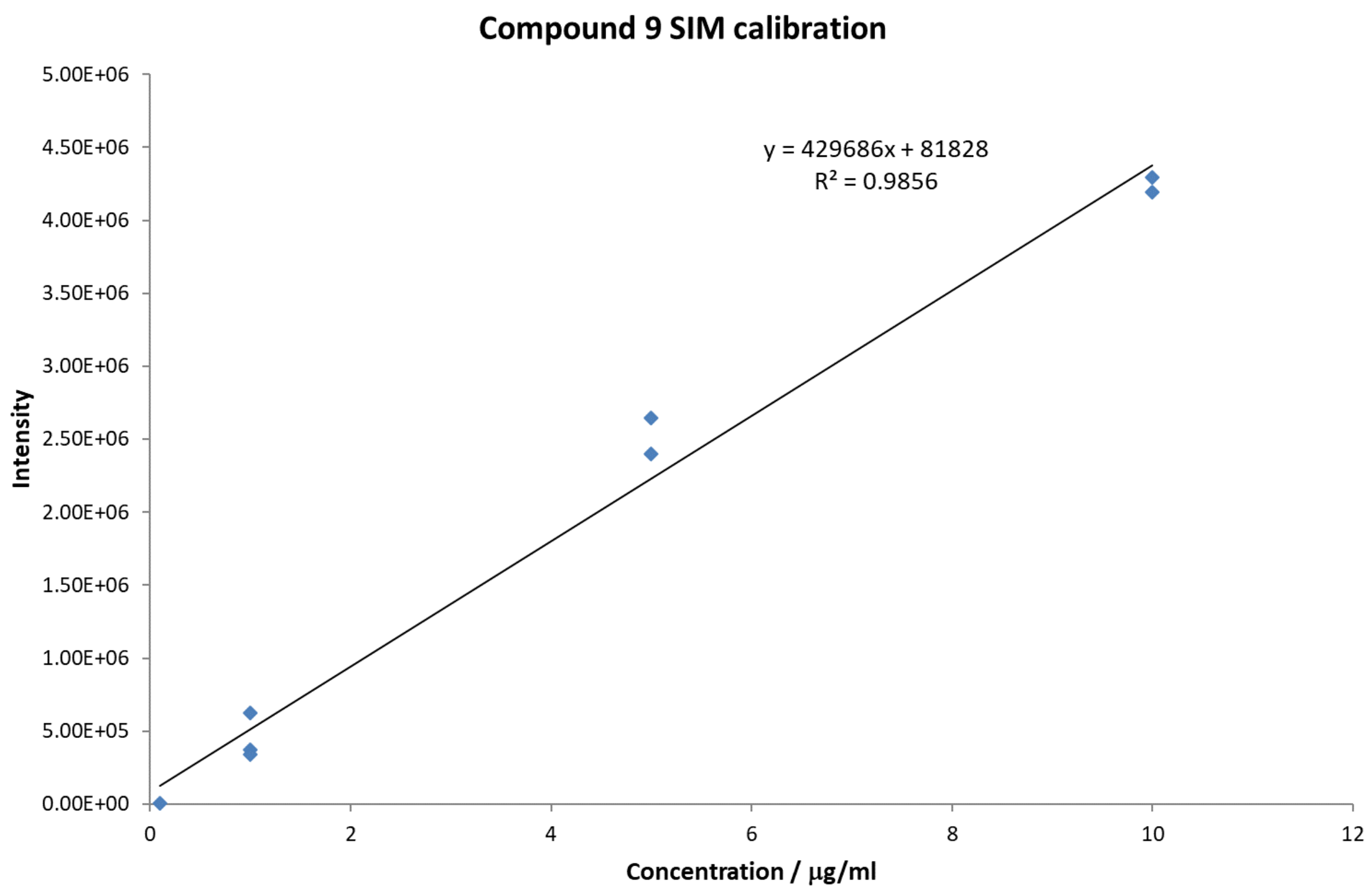

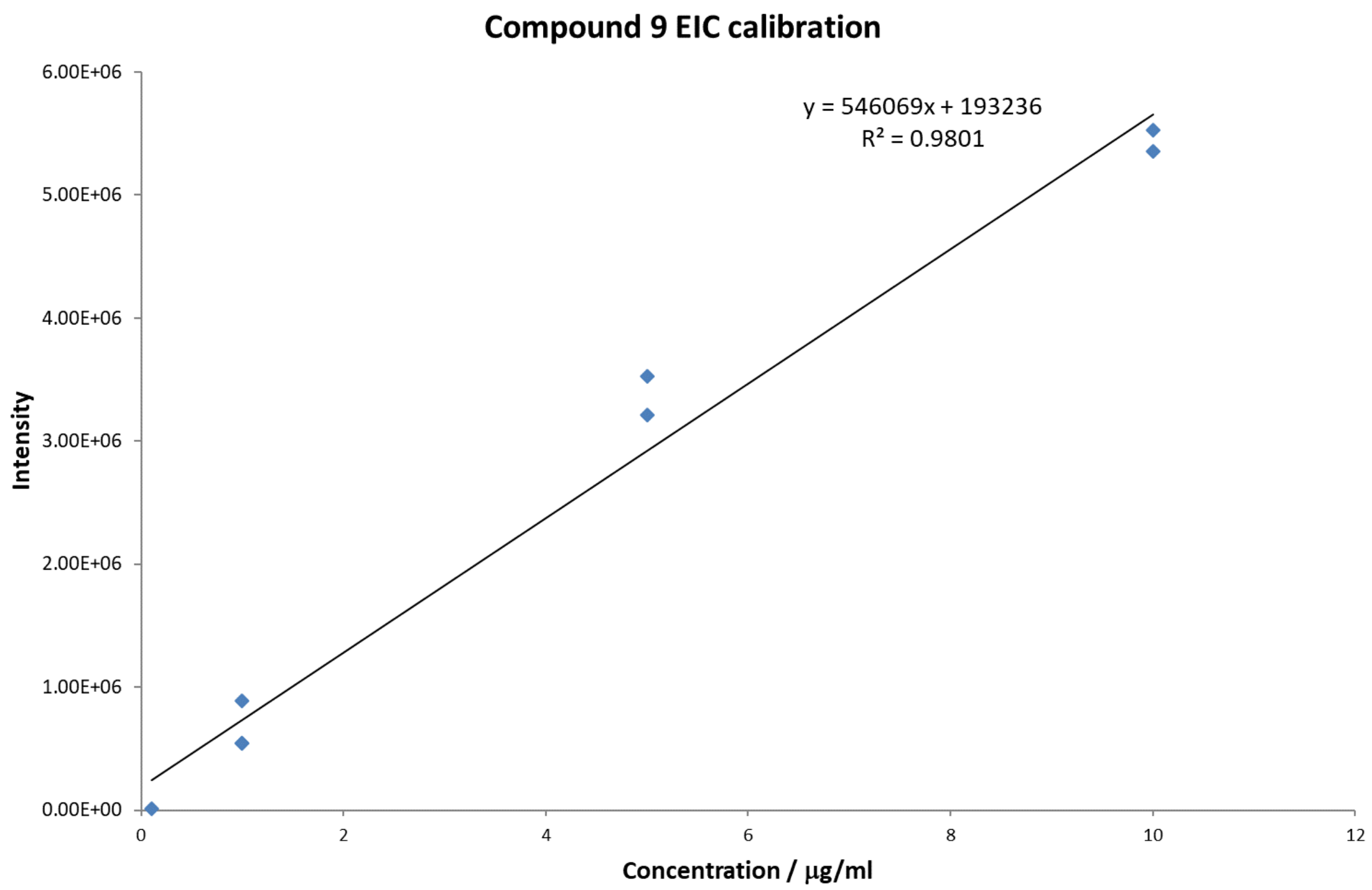

Figure S7. Calibration Curve of Compound 9 from Selected Ion Monitoring (SIM) and Extracted Ion Chromatogram (EIC) Observed Peak Area.

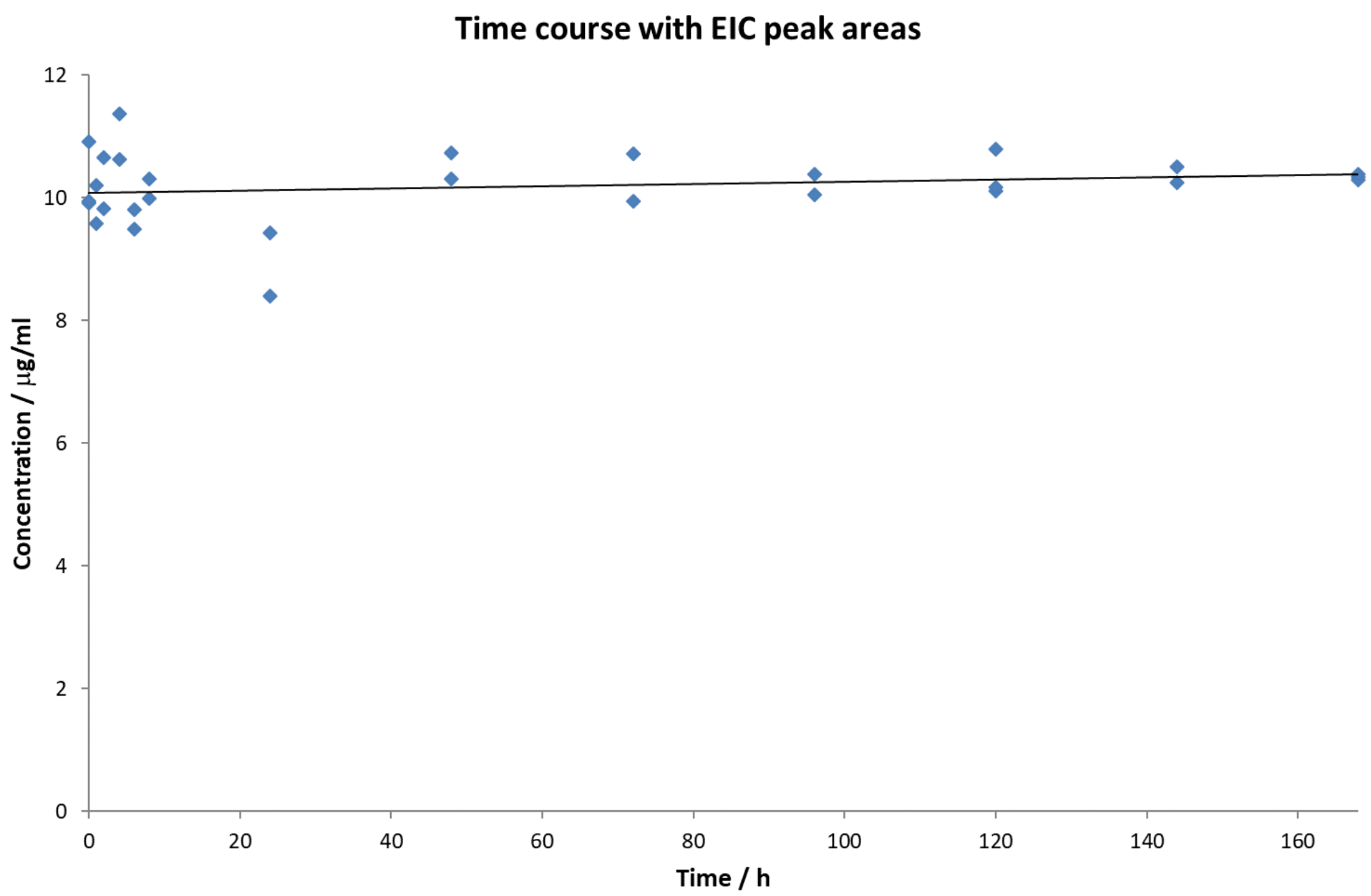

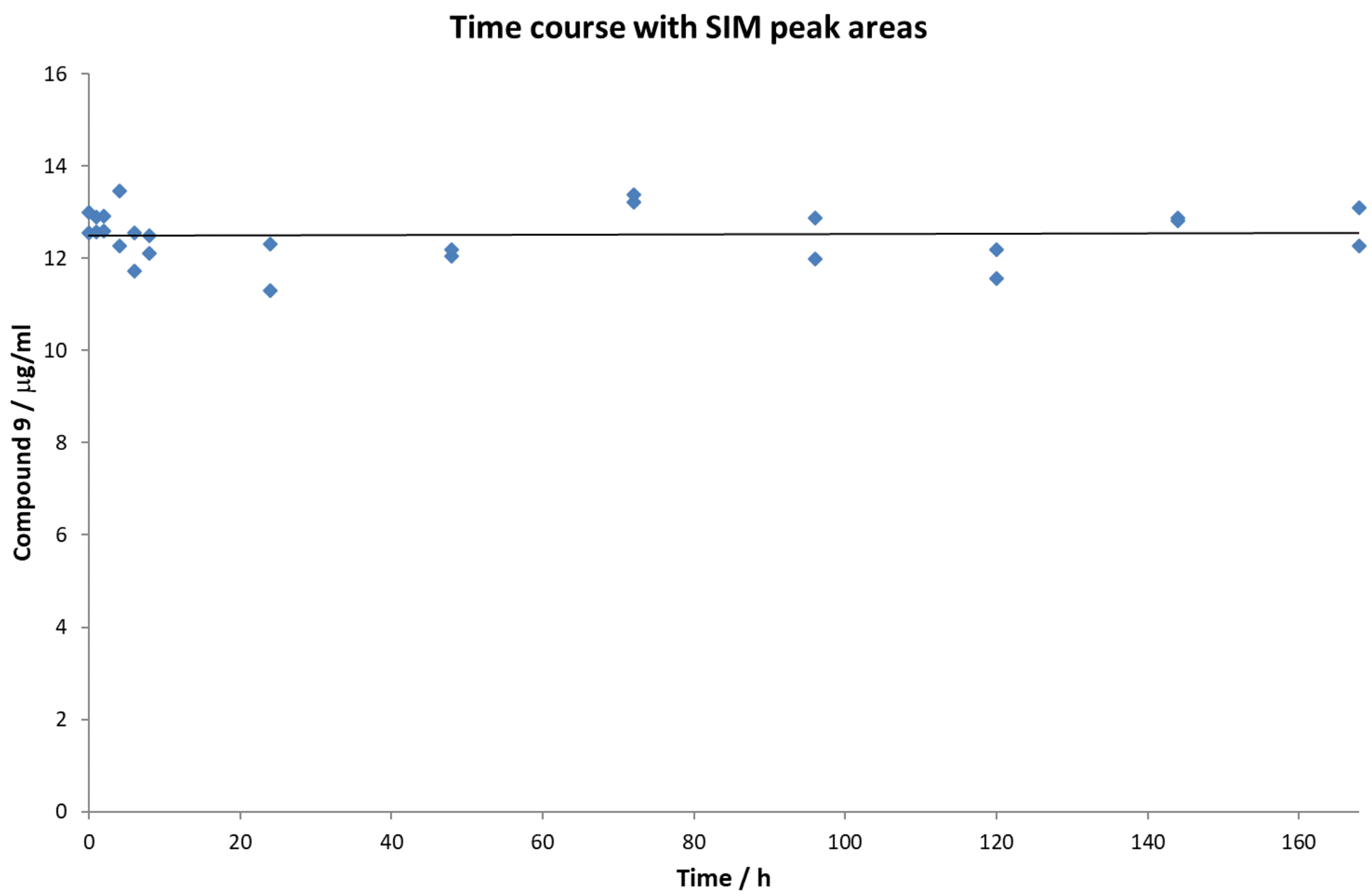

Figure S8. Time course of Compound 9 in Human Plasma over 168 h (7 Days) as Monitored by Selected Ion Monitoring (EIC) and Extracted Ion Chromatogram (SIM). Time points are as follows: 0 h, 1h, 2 h, 4 h, 6 h, 8 h, 24 h, 48 h, 72 h, 96 h, 120 h, 144 h, 168 h.

Both the selected ion monitoring (SIM) and extracted ion chromatogram (EIC) graphs show that compound 9 is completely stable in human plasma at 37 °C. for 7 days.

Metabolic Stability of Compound 9

An in-life pharmacokinetic study (PK) study was performed in male Sprague-Dawley (SD) rats following intravenous (IV) administration of compound **9** at 0.05 mg/kg (50 µg/kg). The plasma samples were collected at 0.05, 0.167, 0.5, 1, 2, 4, 8, and 24 hour(s) after IV injection.

This PK study was outsourced to Pharmacology Discovery Services.

Test Substance and Dosing Regimen

Ligand 9, pre-formulated by the University of Bristol at 10 mM (4.1 mg/mL) in dimethyl sulfoxide (DMSO), was diluted in 0.9% NaCl (physiological saline) at 0.01 mg/mL (10 µg/mL) for IV administration at a dosing volume of 5 mL/kg.

Animals

Male Sprague-Dawley (SD) rats weighing 180 - 250 g were provided by BioLasco Taiwan (under Charles River Laboratories Licensee). Space allocation for two to three animals was 45 x 23 x 21 cm3. All animals were maintained in a controlled temperature (20 - 24oC) and humidity (30% - 70%) environment with 12 hours light/dark cycles during the study period. Free access to standard lab diet [MFG (Oriental Yeast Co., Ltd., Japan)] and autoclaved tap water were granted. All aspects of this work including housing, experimentation, and animal disposal were performed in general accordance with the "Guide for the Care and Use of Laboratory Animals: Eighth Edition" (National Academies Press, Washington, D.C., 2011) in our AAALAC-accredited laboratory animal facility. In addition, the animal care and use protocol was reviewed and approved by the IACUC at Pharmacology Discovery Services Taiwan, Ltd.

Chemicals

0.9% NaCl (Sing-Tong, Taiwan), Acetonitrile (ACN; Fisher Scientific U.K. Ltd., England), DMSO (Honeywell Research Chemicals, USA), Formic acid (FA; Merck, Germany), Losartan (Sigma, USA), and Sodium heparin [Bodene (Pty) Ltd Trading as Intramed, South Africa].

Equipment

0-1000 g Electronic scale (Tanita Corporation, Japan), Animal cage (Allentown, USA), Agilent Poroshell 120 EC-C18 column (2.7 µm, 3.0 x 50 mm; Agilent Technologies, Inc., USA), BD® lithium heparin tube (BD®, USA), Centrifuge 5810R (Eppendrof, Germany), Disposal syringe (1 mL, Terumo Corporation, Japan), Gilson Pipettes (# P200 Neo-P10N Micro Pipette; Gilson,

France), Microcentrifuge tubes 1.5 mL click-cap (Treff AG, Switzerland), NEPHELOstar® microplate reader (BMG LabTech, Germany), Pipetman (# P200 Gilson, France), Pipette Tips (Costar, USA), Pipette Tips (Costar, USA), LC-MS/MS Triple Quad™ 5500+ (SCIEX, USA), Polypropylene 96-well round U-bottom deepwell plates and silicone microplate lids (StorPlate-96 U and StorMat-96; PerkinElmer Inc., USA), RAININ Pipettes (E4 Multi E12-50XLS+, E12-300XLS, Refurbished Rainin E4™ XLS™ electronic 12 channel pipette, 30-300 µL, E4 Pipette Multi E12-1200XLS+, and EA6-300XLS; RAININ, USA), and Stop watch (Casio, China).

Animal Preparation and Surgery

Few days prior to the compound administrations, animals were initially anesthetized with pentobarbital [50 mg/kg, IP] injection for jugular catheter implantation. A single dose of meloxicam [1 mg/kg, subcutaneously (SC)] was given as the analgesics to relieve the post-operative pain.

Plasma Sample Collection from Rats

Blood aliquots (300-400 µL) were collected from jugular vein catheterized rats into tubes coated with lithium heparin at the specified time intervals. The tubes were mixed gently and kept on ice and then centrifuged at 2,500 ×g for 15 minutes at 4°C, within 1 hour after collections.

Pharmacokinetics

The exposure levels (ng/mL) of Ligand 9 in plasma were reported. The plot of plasma concentrations versus (vs.) time (mean ± SD) for Ligand 9 was constructed. The fundamental PK parameters of Ligand 9 after IV dosing were obtained from NCA of the plasma data using WinNonlin (best-fit mode).

Results

Body weight for each animal recorded before dosing

Dose	N	Body Weight (g)
**0.05 mg/kg 5 mL/kg IV**	1	217
	2	209
	3	217
	Mean	214.3
	SEM	2.7

SEM – standard error of the mean

Pharmacokinetic Study and Bioanalysis in Rat

	Sample concentration (ng/mL)				
**Time (h)**	**Rat #1**	**Rat #2**	**Rat #3**	**Mean**	**SD**
**0.05**	31.43	25.98	19.49	25.63	5.98
0.167	9.41	7.66	5.10	7.39	2.17
0.5	1.11	0.08	0.08	0.42	0.59
1	0.14	0.10	0.09	0.11	0.03
2	0.08	BLOQ	0.08	0.08	0.00
4	BLOQ	0.08	BLOQ	0.08	N/A
8	BLOQ	BLOQ	BLOQ	BLOQ	N/A
24	BLOQ	BLOQ	BLOQ	BLOQ	N/A

BLOQ – below 75% of lower limit of quantification; SD – Standard Deviation

Metabolic Half-life of compound **9**

Animal	$t_{1/2}$ (h)
Rat #1	0.24
Rat #2	0.69
Rat #3	0.28
Mean	0.40
SD	0.25

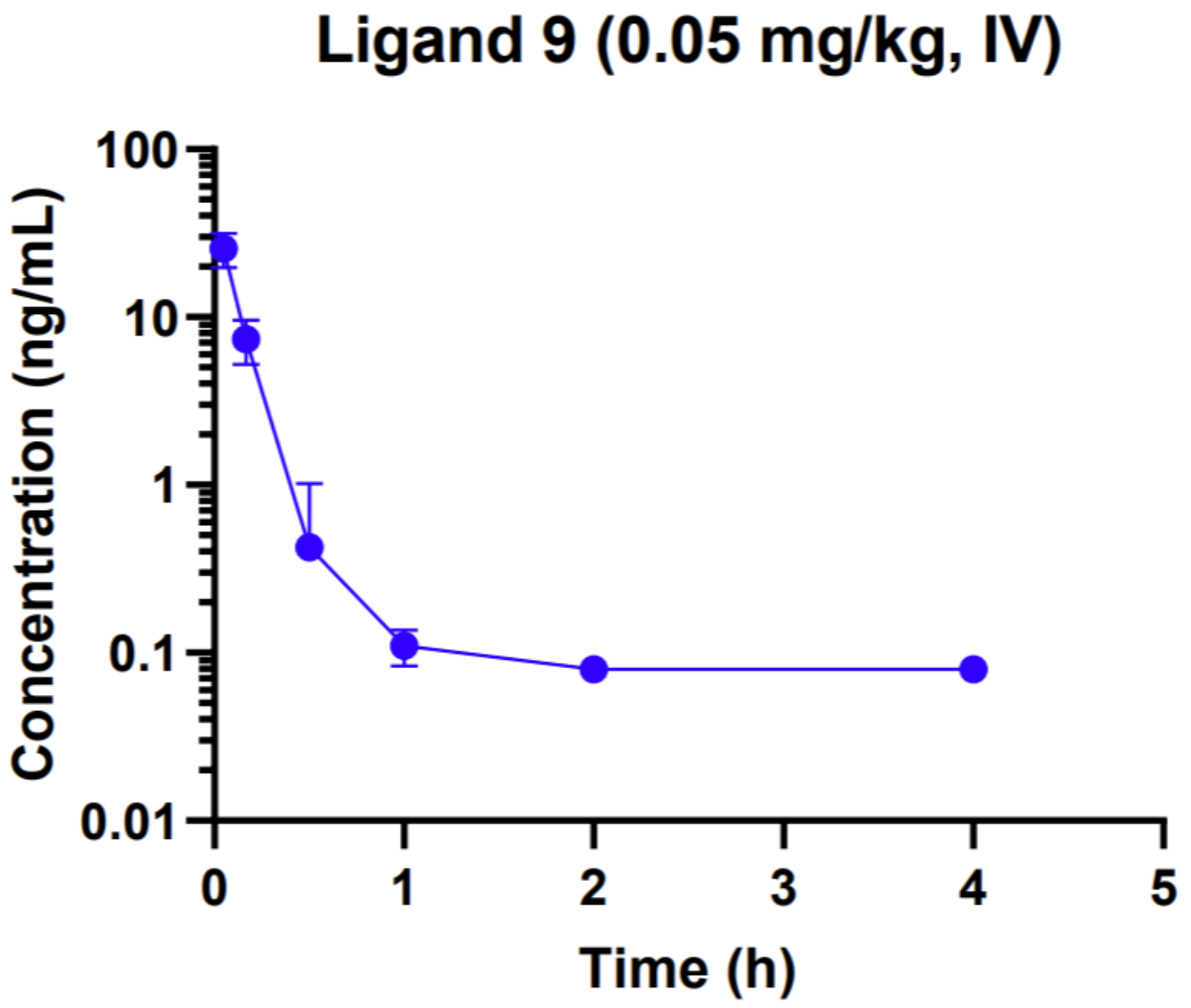

Figure S9 Exposure levels (ng/mL) of compound **9** in rat plasma samples after administration (0.05 mg/kg, IV) analysed through LC-MS/MS.

Modelling Studies

For the molecular docking, the structure of MRE-269 and treprostinil bound IP receptors were used (PDBs 8X79 and 8X7A respectively).[18] Chain E of each was selected, corresponding to the IP receptor. Non-protein atoms were removed. Each of the selected compounds was drawn in 2D using ChemDraw and converted to 3D and minimised using Chem3D (Revvity Signals Software, Inc) before being saved as a .sdf. These were then used for running docking calculations, using the deep learning augmented GNINA 1.0 protocol.[19] A docking box was built centred on the ligand from the experimental PDB using a 4 Å buffer on all sides. Poses were ranked according to GNINA's convolutional neural network (CNN). For all compounds, better scores and more consistent poses were observed for the MRE-269-bound receptor than the treprostinil-bound, so these data were used for downstream analysis. For all compounds, we selected the pose that had a combination of the best CNN score, highest predicted affinity (in kcal/mol) and most similarity to the other poses. All selected poses had CNN scores above 0.8 and affinities above -9.0 kcal/mol, suggesting very high confidence. Docked poses were analysed using the Protein-Ligand Interaction Profiler and PyMOL (Schrödinger LLC).[20]

Optimal poses for each compound were then used to seed atomistic molecular dynamics (MD) simulations. The receptor-compound complexes were each built into a lipid bilayer consisting of POPC and cholesterol in a ratio of 8:2 and solublised with TIP3 waters and neutralised with sodium and chloride ions at a concentration of 0.15M via CHARMM-GUI Membrane Builder.[21] Proteins were described using the CHARMM36m forcefield and the compounds were each parameterised using the CHARMM-GUI ligand reader.[22-23] Protein side chains were all set to default protonation states.

Using GROMACS 2024, the systems were minimised and equilibrated according to standard CHARMM-GUI protocol.[24] MD simulations were then conducted across each system for 50 ns. Simulations used a 2 fs time step in the NPT ensemble with the velocity-rescale thermostat and c-rescale pressure coupling. The results of the MD simulations were analysed using built-in GROMACS tools. MM/PBSA calculations were run for each system using gmx_mmpbsa using the entire trajectory using the dual trajectory method. To account for the use of CHARMM36m, PBRadii was set to 7.[25] Temperature was set to 303.15. Other settings were as follows:

/

```
&pb
memopt=2, emem=4.0, indi=1.0,
poretype=1,
radiopt=0, istrng=0.150, fillratio=1.25, inp=2,
solvopt=1, ipb=1, bcopt=10, nfocus=1, linit=1000,
eneopt=1, cutfd=7.0, cutnb=99.0,
maxarcdot=15000,
npbverb=1,


/
&decomp
idecomp=2, dec_verbose=3,
print_res="within 6"
/
```

Supplementary Molecular Modelling Figures

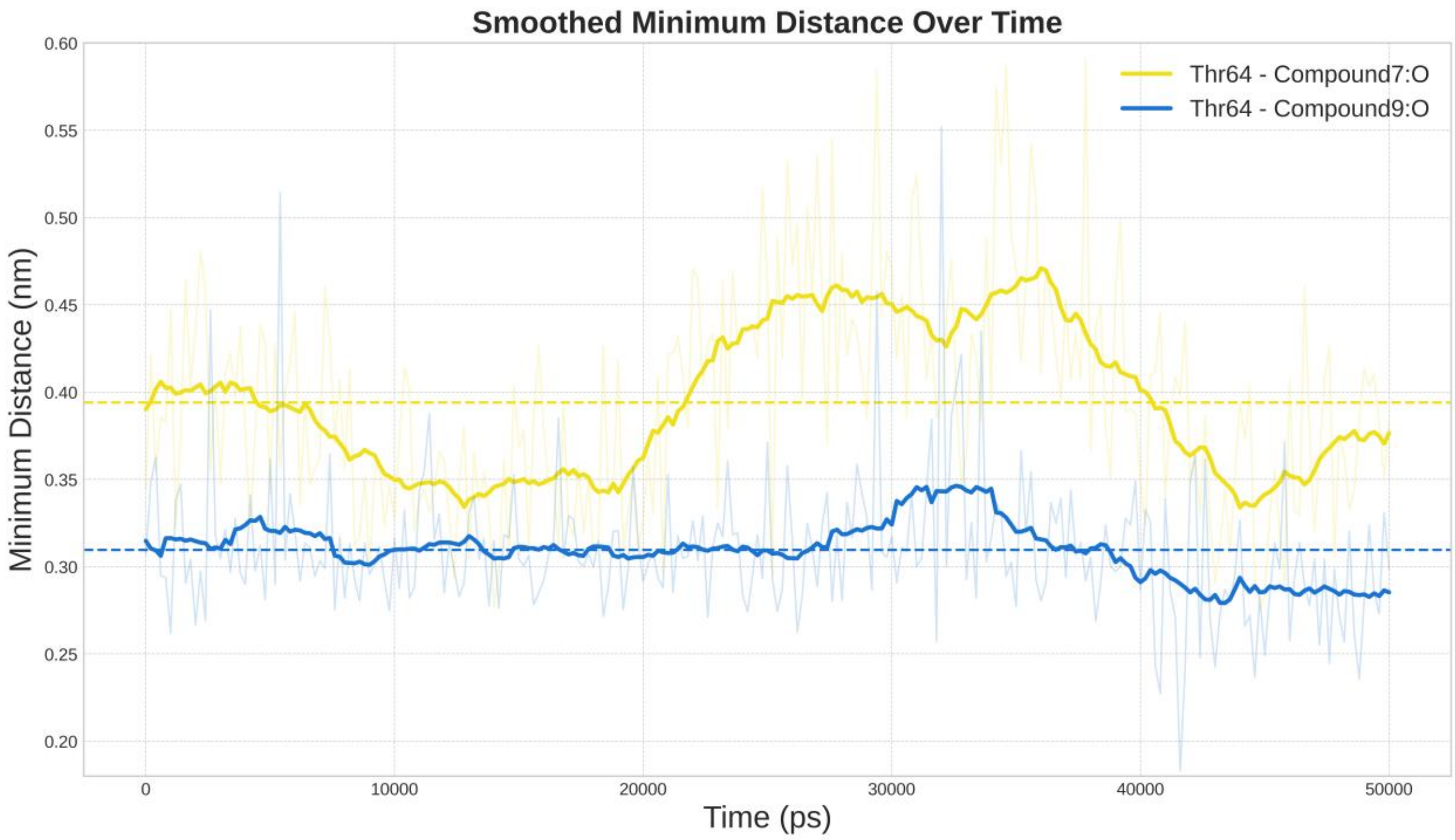

Figure S10. Plot of the average minimum distance between the hydroxyl O atom of compounds 7 and 9, and the hydroxyl of amino acid Thr64. See accompanying figures 4b and 4c in the main text.

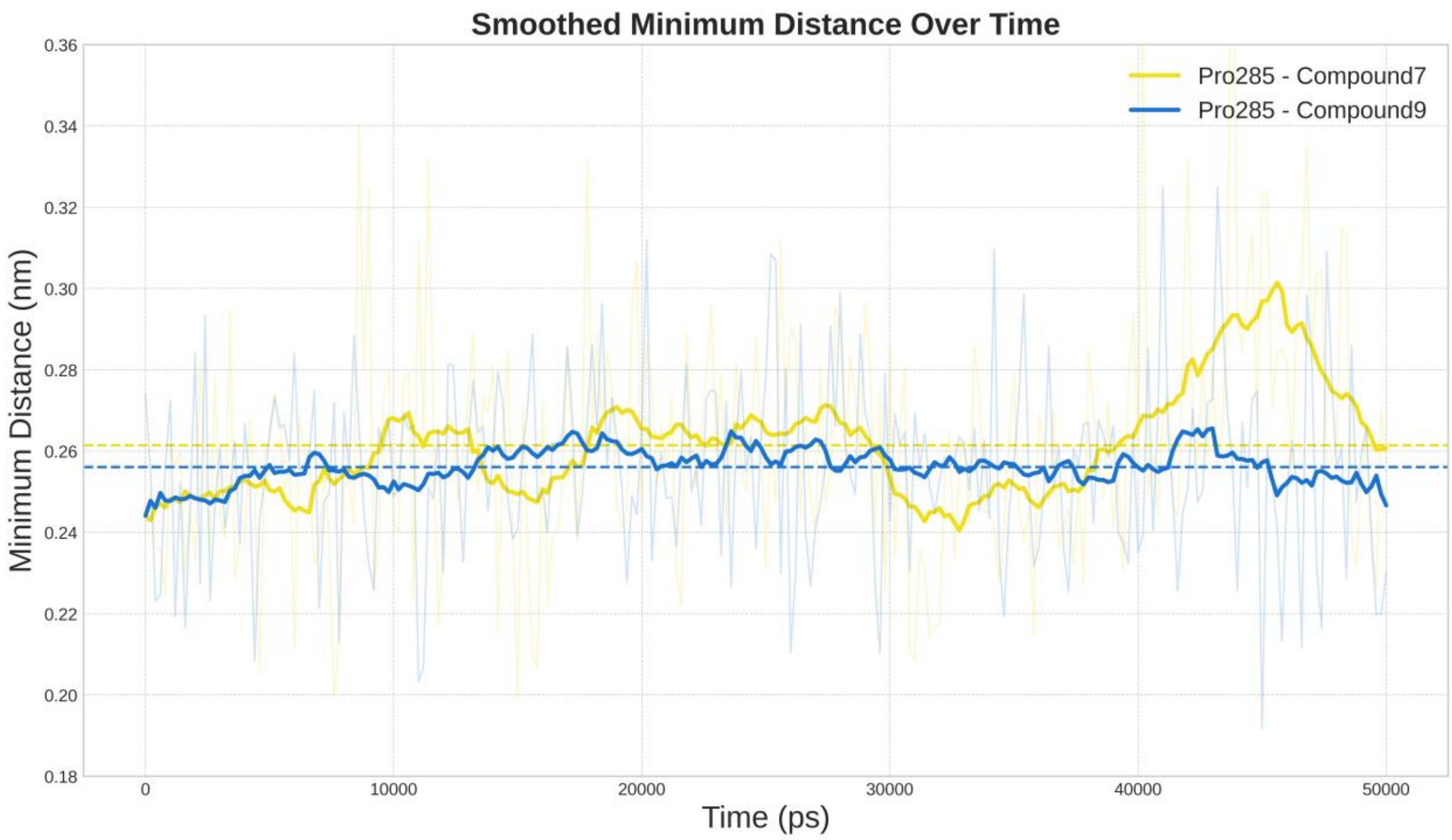

Figure S11. Plot of the average minimum distance between the hydroxyl O atom of compounds 7 and 9, and the nearest atom of the amino acid Pro285. See accompanying figures 4b and 4c in the main text.

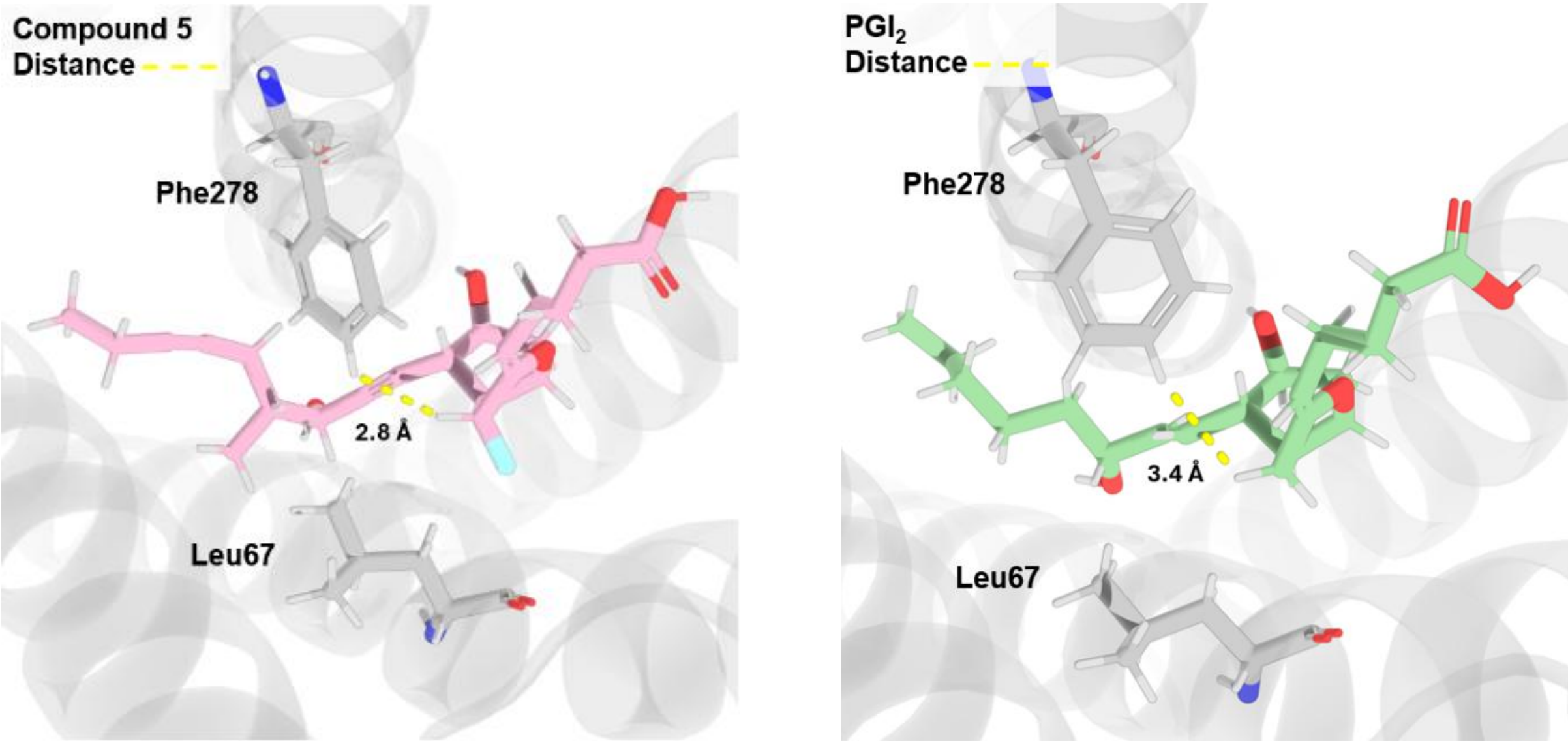

Figure S12. View of compound 5 (pink) and PGI_2 (green) docked in IP receptor (grey) with the average distance from C—H to Phe278 side chain shown (yellow dashed line). Phe278 forms hydrophobic interactions with the compounds. See Figure 4D-E in the main text for compounds **7** and **9**.

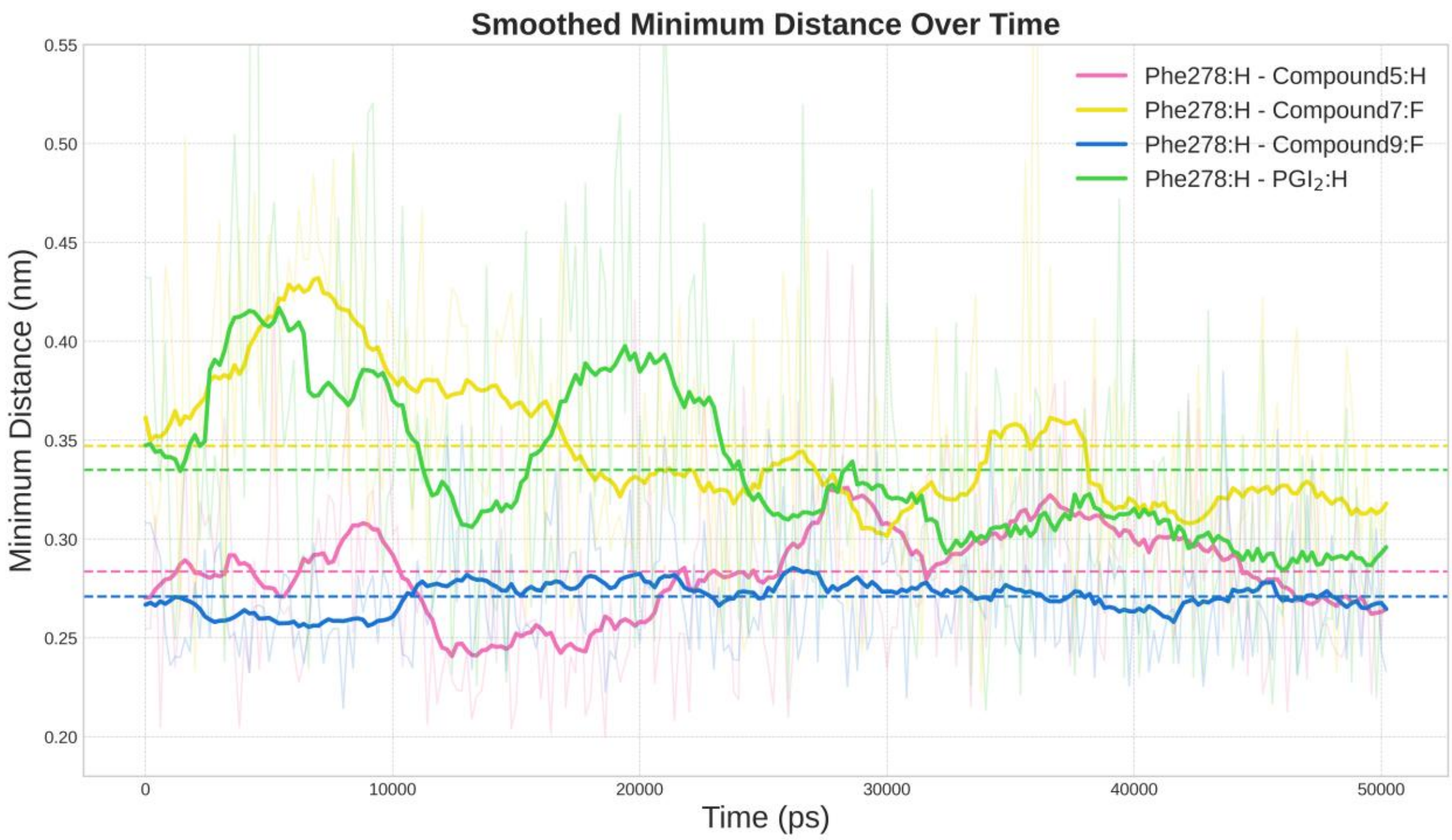

Figure S13. Plot of the average minimum distance between the H/F atom of compounds 5, 7, 9, and PGI_2, and the amino acid Phe278. See accompanying figures 4d and 4e in the main text, and structural view above.

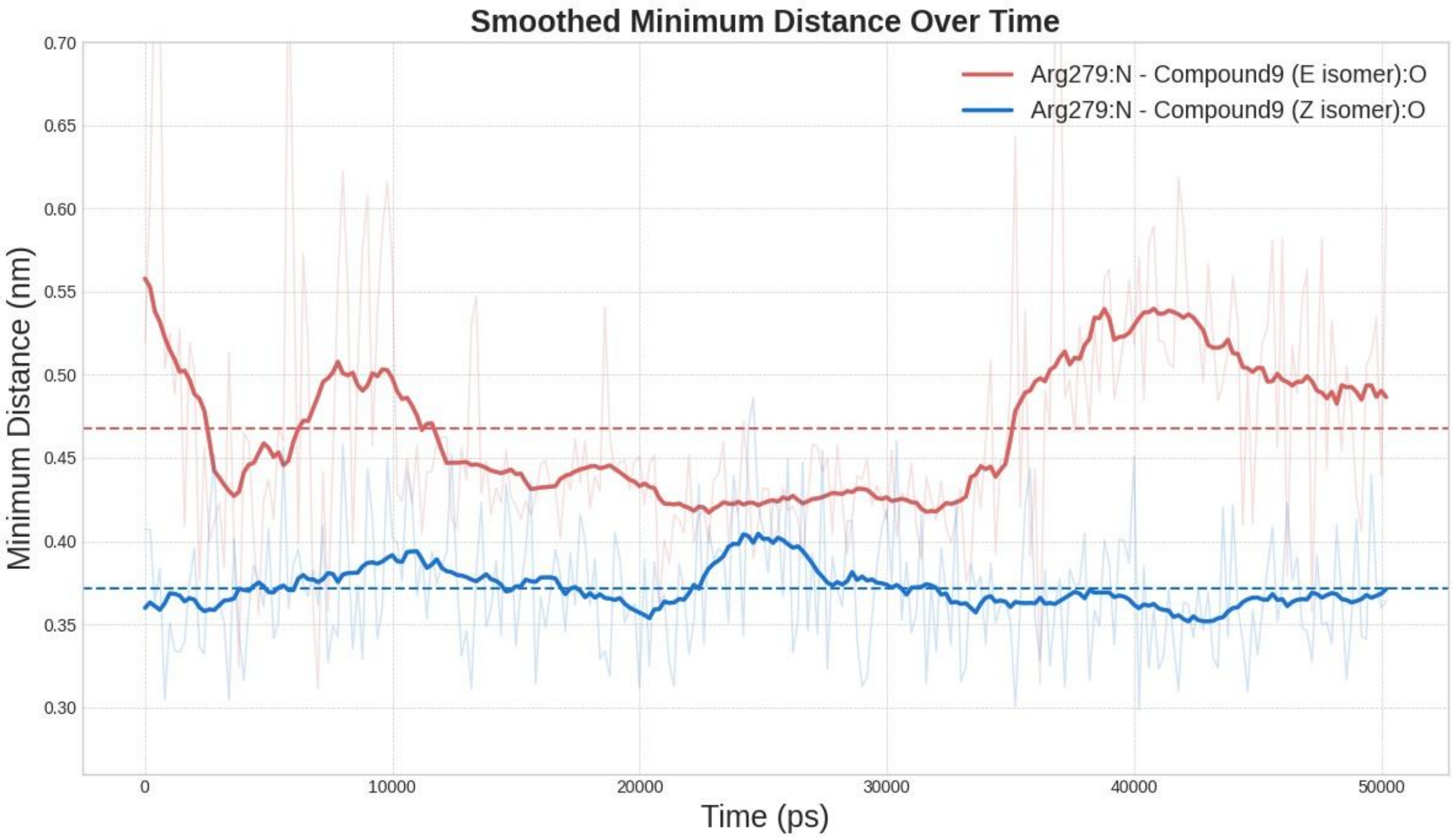

Figure S14. Plot of the average minimum distance between the carboxylic acid hydroxyl of isomers *E*-9 and *Z*-9 and the guanidinium of amino acid Arg279. See accompanying figure 4f in the main text.

Experimental Procedures

Synthesis of the key bicyclic enals

Synthesis of lactol 10 from 2,5-dimethoxytetrahydrofuran (S1)

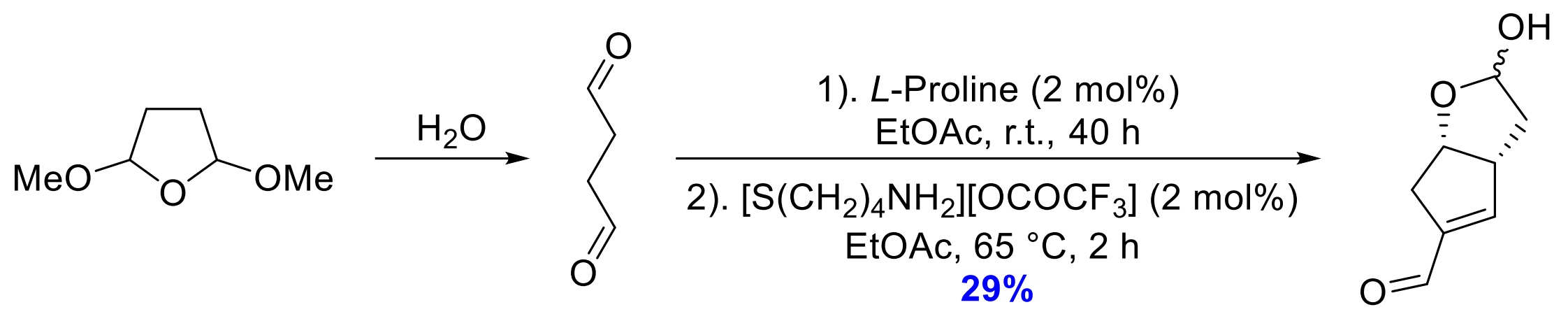

Lactol **10** was prepared from freshly distilled succinaldehyde **S2** according to the literature and analytical data was consistent with data reported in the literature.[3]

(3a*R*,6a*S*)-2-Oxo-3,3a,6,6a-tetrahydro-2*H*-cyclopenta[*b*]furan-5-carbaldehyde (12)

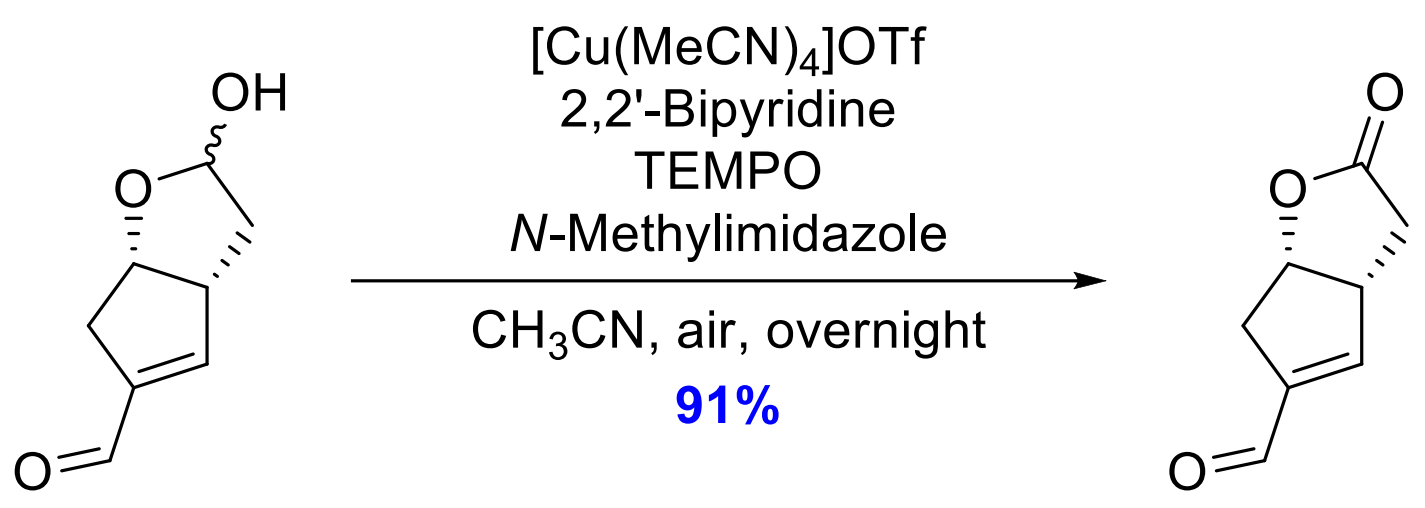

To a solution of lactol **10** (13.0 g, 84.3 mmol, 1.0 eq.) in acetonitrile (420 mL) were added tetrakisacetonitrile copper (I) triflate (1.59 g, 4.22 mmol, 5 mol%), 2,2'-bipyridine (658 mg, 4.22 mmol, 5 mol%), TEMPO (658 mg, 4.22 mmol, 5 mo %) and *N*-methylimidazole (671 µL, 8.44 mmol, 10 mol%). The reaction mixture was stirred at r.t. overnight and then filtered through a silica plug, washed with EtOAc. The filtrate was concentrated, and the crude mixture was purified by flash column chromatography on silica gel (Hexanes/EtOAc = 50:50 to 30:70) to give the lactone **12** as a white solid (11.7 g, 76.7 mmol, 91%, 99:1 er).

Analytical data was consistent with data reported in the literature.[4]

The enantiomeric excess was determined by SFC analysis:
Chrial SFC Conditions: Chiralpak® IA column; 125 bar, 40 °C, flow rate 4 mL/min, CO_2/*i*-PrOH = 95:5; retention time: t_{minor} = 7.3 min, t_{major} = 8.7 min; er = 99:1.

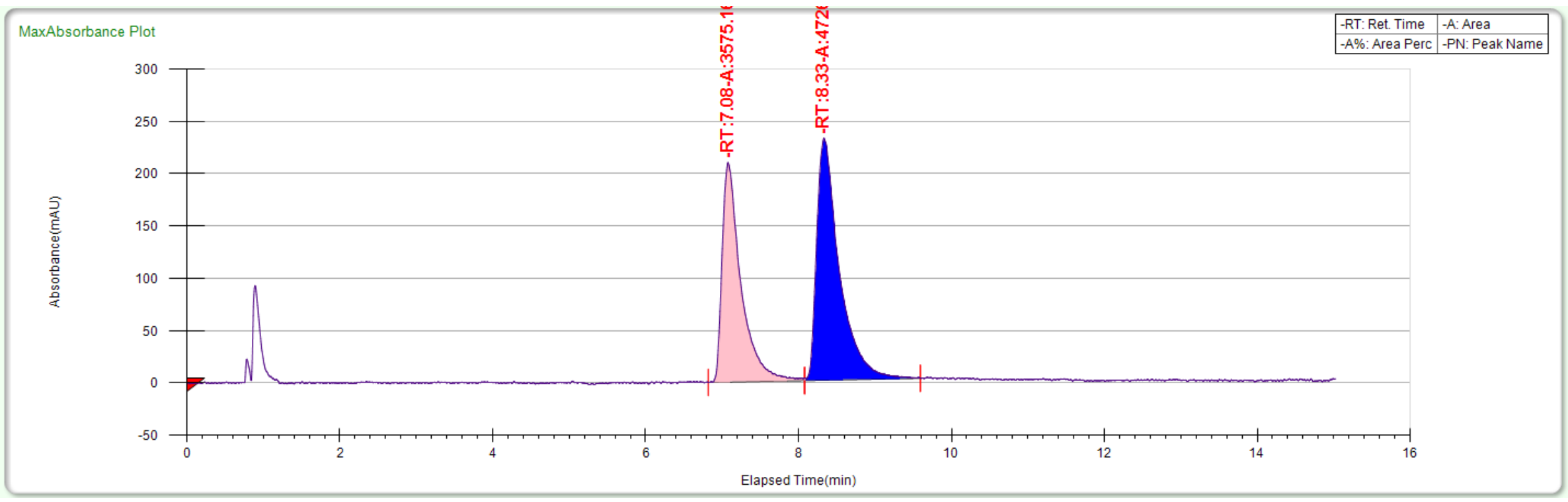

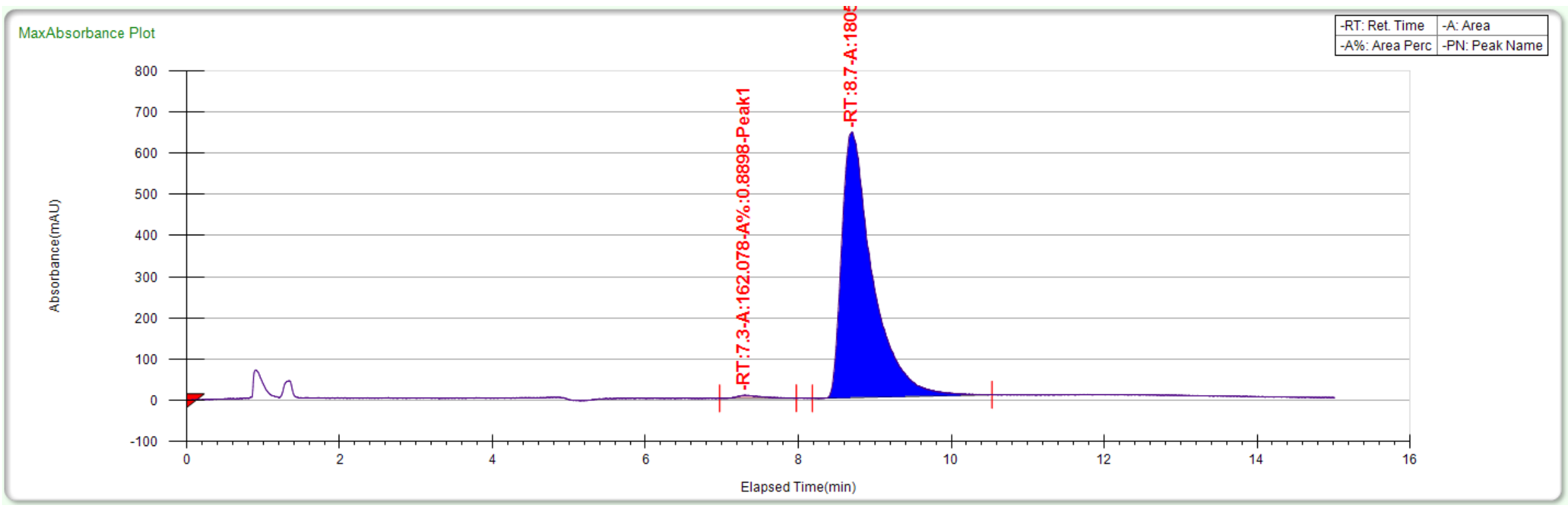

Synthesis of side chains

Synthesis of (*S*,*E*)-((1-bromooct-1-en-3-yl)oxy)(*tert*-butyl)dimethylsilane (S6) from S3

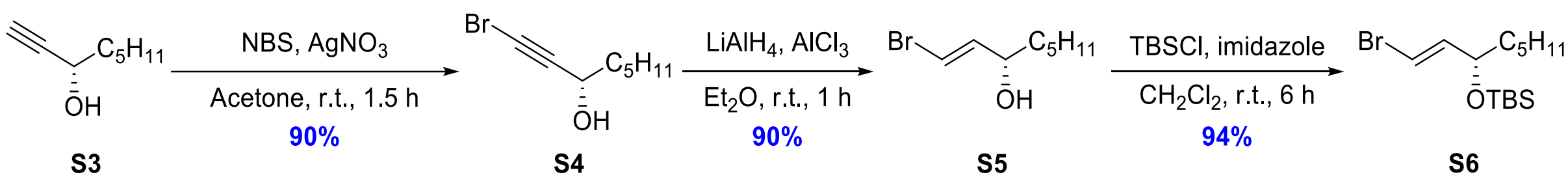

(*S*)-1-Bromooct-1-yn-3-ol (S4)

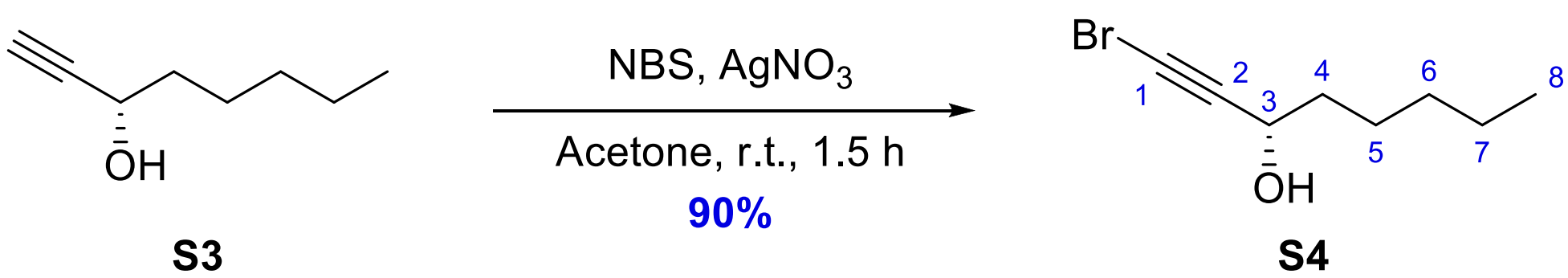

To a stirring solution of **S3** (3.79 g, 30.0 mmol, 1.0 eq.) in acetone (150 mL) were added NBS (6.41 g, 36.0 mmol, 1.2 eq.) and $AgNO_3$ (510 mg, 3.00 mmol, 10 mol%) sequentially. The reaction mixture was stirred at r.t. for 1.5 h before it was poured over H_2O (160 mL) and extracted with EtOAc (3 × 160 mL). The organic phases were combined, washed with brine, dried over $MgSO_4$, filtered, and the solvent was removed under reduced pressure. The crude product was purified by flash column chromatography on silica gel (Hexanes/EtOAc = 95:5 to 90:10) to give alcohol **S4** as a clear, colourless oil (5.54 g, 27.0 mmol, 90%).

$\boldsymbol{R_f}$ = 0.46 (Hexanes/EtOAc = 90:10)

IR (thin film) ν_{max} /cm^{-1}: 3357, 2958, 2933, 2860, 1462, 1390, 1250, 1224, 1055, 895, 757.
^{1}H NMR (400 MHz, $CDCl_3$) δ_H = 4.39 (t, J = 6.6 Hz, 1H, ***H***C3), 1.73 – 1.67 (m, 3H, ***H*****2**C4+O***H***), 1.49 – 1.39 (m, 2H, ***H*****2**C5), 1.38 – 1.27 (m, 4H, ***H*****2**C6+***H*****2**C7), 0.90 (t, J = 7.0 Hz, 3H, ***H*****3**C8).
^{13}C NMR (100 MHz, $CDCl_3$) δ_C = 81.4 (**C2**), 63.4 (**C3**), 45.1 (**C1**), 37.8 (**C4**), 31.5 (**C6**), 24.8 (**C5**), 22.7 (**C7**), 14.1 (**C8**).
$[\alpha]_D^{25}$ = +3.0 (c 1.00, $CHCl_3$).
Analytical data is consistent with data reported in the literature.[5]

(*S,E*)-1-Bromooct-1-en-3-ol (S5)

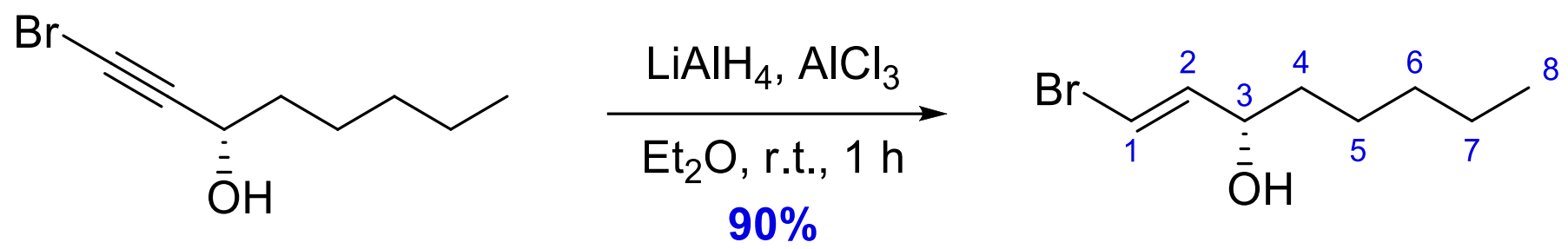

To a solution of $LiAlH_4$ (1.52 g, 40.0 mmol, 2.0 eq.) in dry Et_2O (75 mL), $AlCl_3$ (4.00 g, 30.0 mmol, 1.5 eq.) was carefully added portion-wise. A solution of **S4** (4.10 g, 20.0 mmol, 1.0 eq.) in dry Et_2O (14.5 mL) was added dropwise to the above mixture, and the resulting reaction mixture was stirred at r.t. for 1 h. Following cooling to 0 °C, H_2O (1.52 mL) was carefully added dropwise followed by aqueous NaOH solution (0.5 M, 1.52 mL) and then a further portion of H_2O (4.55 mL). The mixture was stirred at r.t. for 30 min, $MgSO_4$(*s*) (20.5 g) was added and the mixture was stirred for a further 15 min prior to sinter filtration, washing with Et_2O (3 × 80 mL). The filtrate was concentrated *in vacuo* to afford pure **S5** as a clear, colourless oil (3.71 g, 17.9 mmol, 90%).

***R*f** = 0.46 (Hexanes/EtOAc = 90:10)
IR (thin film) ν_{max} /cm^{-1}: 3358, 2956, 2929, 2858, 1620, 1465, 1379, 1266, 1028, 937, 791, 724.
^{1}H NMR (400 MHz, $CDCl_3$) δ_H = 6.32 (dd, J = 13.6, 0.9 Hz, 1H, ***H***C1), 6.23 (dd, J = 13.6, 6.5 Hz, 1H, ***H***C2), 4.11 (qd, J = 6.9, 0.8 Hz, 1H, ***H***C3), 1.72 (br.s, 1H, O***H***), 1.54 – 1.48 (m, 2H, ***H*****2**C4), 1.35 – 1.27 (m, 6H, ***H*****2**C5+***H*****2**C6+***H*****2**C7), 0.89 (t, J = 6.9 Hz, 3H, ***H*****3**C8).
^{13}C NMR (100 MHz, $CDCl_3$) δ_C = 140.6 (**C2**), 107.1 (**C1**), 72.8 (**C3**), 37.0 (**C4**), 31.8 (**C6**), 25.0 (**C5**), 22.7 (**C7**), 14.1 (**C8**).
$[\alpha]_D^{25}$ = +4.0 (c 1.00, $CHCl_3$) [lit.[5] **$[\alpha]_D^{23}$** = +5.0 (c 2.00, $CHCl_3$)]
Analytical data is consistent with data reported in the literature.[5,6]

(*S*,*E*)-((1-Bromooct-1-en-3-yl)oxy)(*tert*-butyl)dimethylsilane (S6)

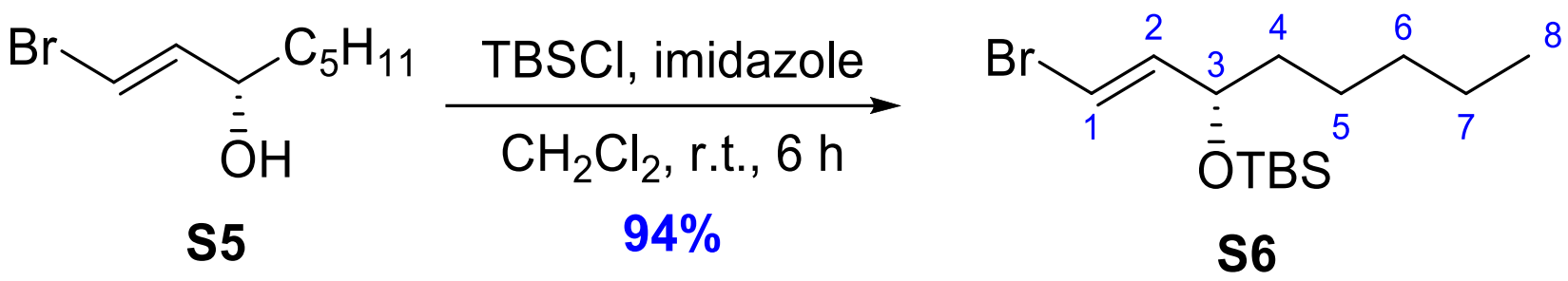

Imidazole (2.44 g, 35.8 mmol, 2.0 eq.) and TBSCl (3.78 g, 25.1 mmol, 1.4 eq.) were sequentially added to a stirring solution of **S5** (3.70 g, 17.9 mmol, 1.0 eq.) in CH_2Cl_2 (40 mL) at 0 °C. The reaction mixture was stirred at r.t. for 6 h before it was poured over aqueous HCl (0.5 M, 80 mL) and extracted with Et_2O (3 × 80 mL). The organic phases were combined, dried over $MgSO_4$, filtered and concentrated *in vacuo*. The crude product was purified by flash column chromatography on silica gel (Hexanes) to give pure **S6** as a clear, colourless oil (5.41 g, 16.8 mmol, 94%).

$\boldsymbol{R_f}$ = 0.52 (Hexanes)
IR (thin film) $\boldsymbol{\nu_{max}}$/cm^{-1}: 2955, 2929, 2857, 1624, 1470, 1360, 1255, 1089, 936, 835, 715, 706.
^{1}H NMR (400 MHz, $CDCl_3$) δ_H = 6.22 – 6.14 (m, 2H, ***H***C1= ***H***C2), 4.09 (tdd, *J* = 6.2, 4.0, 0.9 Hz, 1H, ***H***C3), 1.52–1.41 (m, 2H, ***H*₂**C4), 1.35 – 1.23 (m, 6H, $\boldsymbol{H_2}C_5$+$\boldsymbol{H_2}C_6$+$\boldsymbol{H_2}C_7$), 0.90 – 0.87 (m, 12H, SiC(C$\boldsymbol{H_3}$)$_3$+$\boldsymbol{H_3}C_8$), 0.05 (s, 3H, SiC$\boldsymbol{H_3}$), 0.04 (s, 3H, SiC$\boldsymbol{H_3}$).
^{13}C NMR (100 MHz, $CDCl_3$) δ_C = 141.2 (**C2**), 105.6 (**C1**), 73.3 (**C3**), 37.9 (**C4**), 31.9 (**C6**), 26.0 (SiC(*C*H$_3$)$_3$), 24.7 (**C5**), 22.5 (**C7**), 18.3 (Si*C*(CH$_3$)$_3$), 14.2 (**C8**), -4.4 (Si*C*H$_3$), -4.7 (Si*C*H$_3$).
$[\alpha]_D^{24}$ = −14.0 (*c* 1.00, $CHCl_3$) [lit.[7] $[\alpha]_D^{22}$ = –19.9 (*c*. 1.00, $CHCl_3$)]
Analytical data is consistent with data reported in the literature.[7]

Synthesis of *tert*-butyl((3*S*,4*S*,*E*)-1-iodo-4-methyloct-1-en-6-yn-3-yloxy)dimethylsilanefrom (18) from 13

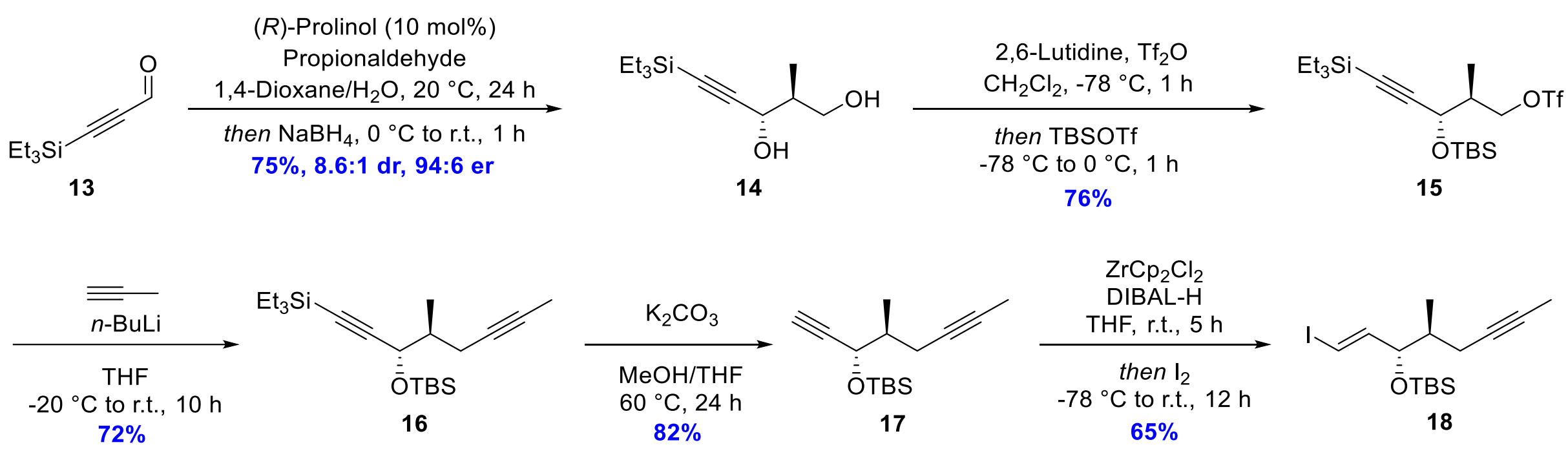

3-(Triethylsilyl)propiolaldehyde (**13**)[8] and (*R*)-2-[Bis(3,5-bis-trifluoromethyl-phenyl)hydroxymethyl]pyrrolidine[9] were prepared according to literature procedures.

(2*S*,3*S*)-2-Methyl-5-(triethylsilyl)pent-4-yne-1,3-diol (14)

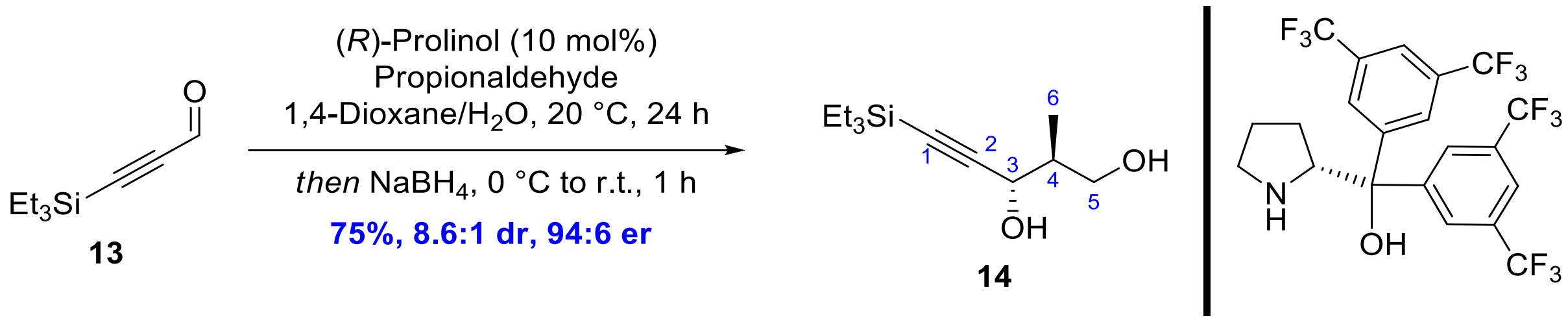

(*R*)-2-[Bis(3,5-bis-trifluoromethyl-phenyl)hydroxymethyl]pyrrolidine (2.14 g, 4.07 mmol, 10 mol%), H_2O (2.20 mL, 122 mmol, 3.0 eq.) and propionaldehyde (7.17 mL, 5.92 g, 102 mmol. 2.5 eq.) were sequentially added to a stirring solution of 3-(triethylsilyl)propiolaldehyde (6.86 g, 40.7 mmol, 1.0 eq.) in 1,4-dioxane (41 mL) at r.t.. The reaction mixture was stirred at r.t. for 24 h, then $NaBH_4$ (4.16 g, 110 mmol. 2.7 eq.) was added to the above reaction mixture at 0 °C. After stirring at r.t. for another 1 h, the reaction mixture was quenched by addition of buffer (pH = 7.0) and extracted with EtOAc (3 × 80 mL). The organic phases were combined, washed with brine, dried over $MgSO_4$, filtered and concentrated in *vacuo*. The crude products were purified by flash column chromatography on silica gel (Hexanes/EtOAc = 90:10 to 75:25) to give a mixture of two isomers **14** as a pale yellow oil (6.94 g, 30.4 mmol, 75%, *anti:syn* = 8.6:1). The mixture of two isomers was purified furtherly by flash column chromatography on silica gel (Hexanes/EtOAc = 95:5 to 70:30) to give pure major isomer as a colourless oil (6.20 g, 27.2 mmol).

$\boldsymbol{R_f}$ = 0.23 (Hexanes/EtOAc = 75:25)
IR (thin film) $\boldsymbol{\nu_{max}}$/cm^{-1}: 3339, 2955, 2911, 2875, 2170, 1457, 1415, 1235, 1081, 1004, 978, 721.
^{1}H NMR (400 MHz, $CDCl_3$) δ_H = 4.37 (d, J = 7.0 Hz, 1H, $\boldsymbol{H}C_3$), 3.78 (dd, J = 11.0, 3.9 Hz, 1H, $\boldsymbol{H_A}C_5$), 3.62 (dd, J = 11.0, 7.1 Hz, 1H, $\boldsymbol{H_B}C_5$), 2.92 (overlapping, br.s+br.s, 2H, O***H***+O***H***), 2.01 – 1.91 (m, 1H, $\boldsymbol{H}C_4$), 1.01 – 0.95 (m, 12H, $Si(CH_2C\boldsymbol{H_3})_3$+$\boldsymbol{H_3}C_6$), 0.59 (q, J = 7.9 Hz, 6H, $Si(C\boldsymbol{H_2}CH_3)_3$).
^{13}C NMR (100 MHz, $CDCl_3$) δ_C = 106.8 (**C_2**), 88.0 (**C_1**), 67.3 (**C_3**), 66.4 (**C_5**), 41.5 (**C_4**), 13.1 (**C_6**), 7.5 ($Si(CH_2\boldsymbol{C}H_3)_3$), 4.4 ($Si(\boldsymbol{C}H_2CH_3)_3$).
HRMS (ESI$^+$) calculated for $C_{12}H_{24}NaO_2Si$ [M+Na$^+$] = 251.1438, found 251.1446.
$[\alpha]_D^{24}$ = –1.7 (*c* 1.00, $CHCl_3$)

The er was determined following benzoylation of pure major isomer **14** to the corresponding alcohol **S7** and subsequent analysis by chiral HPLC.

Benzyl (2*S*,3*S*)-3-hydroxy-2-methyl-5-(triethylsilyl)pent-4-ynyl carbonate (S7)

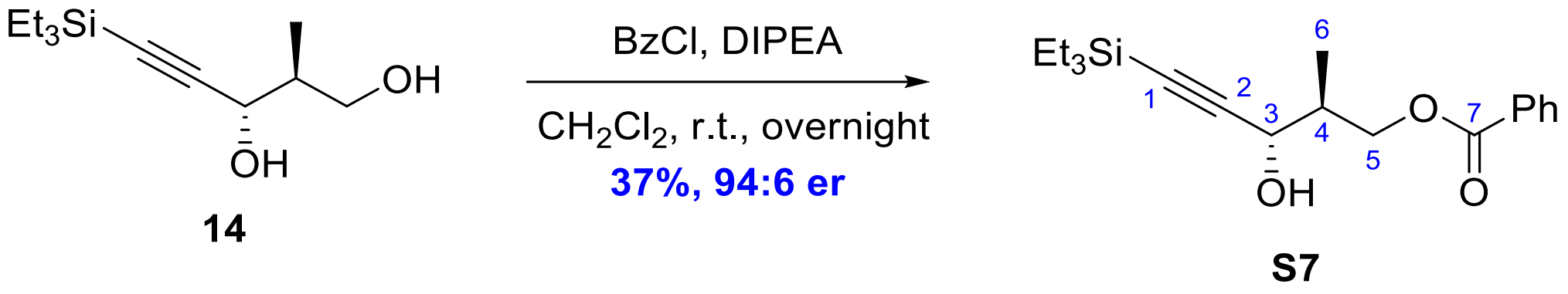

To a solution of alcohol **14** (333 mg, 1.46 mmol, 1.0 eq.) in dry CH_2Cl_2 (3.0 mL) was added DIPEA (0.29 mL, 226 mg, 1.75 mmol, 1.2 eq.) at 0 °C, and BzCl (0.19 mL, 226 mg, 1.61 mmol, 1.1 eq.) was added dropwise to the above mixture at the same temperature. The mixture was stirred at r.t. overnight before MeOH (1.5 mL) was added. After stirred at r.t. for another 15 min, the solvent was removed and the crude mixture was purified by flash column chromatography on silica gel (Hexanes/EtOAc = 90:10 to 80:20) to give pure **S7** as a clear, colourless oil (179 mg, 0.54 mmol, 37%).

$\boldsymbol{R_f}$ = 0.51 (Hexanes/EtOAc = 80:20)

IR (thin film) $\boldsymbol{\nu_{max}}$/cm^{-1}: 3456, 2955, 2910, 2875, 1721, 1703, 1602, 1452, 1388, 1270, 1070, 1025, 708.

^{1}H NMR (400 MHz, $CDCl_3$) δ_H = 8.05 – 8.02 (m, 2H, ***H***-Ar), 7.58 – 7.54 (m, 1H, ***H***-Ar), 7.47 – 7.42 (m, 2H, ***H***-Ar), 4.50 (d, *J* = 6.4 Hz, 1H, ***H***C_3), 4.43 (dd, *J* = 11.1, 5.4 Hz, 1H, $\boldsymbol{H_A}C_5$), 4.33 (dd, *J* = 11.1, 6.2 Hz, 1H, $\boldsymbol{H_B}C_5$), 2.32 – 2.22 (m, 2H, ***H***C_4+O***H***), 1.16 (d, *J* = 6.9 Hz, 3H, $\boldsymbol{H_3}C_6$), 0.98 (t, *J* = 7.9 Hz, 9H, Si($CH_2C\boldsymbol{H_3}$)$_3$), 0.59 (q, *J* = 7.9 Hz, 6H, Si($C\boldsymbol{H_2}CH_3$)$_3$).

^{13}C NMR (100 MHz, $CDCl_3$) δ_C = 166.8 (**C$_7$**), 133.2 (**C$_{Ar}$**H), 130.3 (**C$_{Ar}$**C), 129.7 (**C$_{Ar}$**H), 128.5 (**C$_{Ar}$**H), 105.7 (**C$_2$**), 88.7 (**C$_1$**), 66.4 (**C$_3$**), 64.7 (**C$_5$**), 39.5 (**C$_4$**), 12.7(**C$_6$**), 7.6 (Si(CH_2***C***H_3)$_3$), 4.4 (Si(***C***H_2CH_3)$_3$).

HRMS (ESI$^+$) calculated for $C_{19}H_{27}O_2Si$ [$M+H^+-H_2O$] = 315.1775, found 315.1789.

$[\alpha]_D^{24}$ = +33.0 (*c* 1.00, $CHCl_3$)

Chrial HPLC Conditions: Chiralpak® OD-H column; flow rate 1.0 mL/min, 230 nm, Hexanes/*i*-PrOH = 98:2; retention time: t_{minor} = 8.7 min, t_{major} = 10.5 min; er = 94:6.

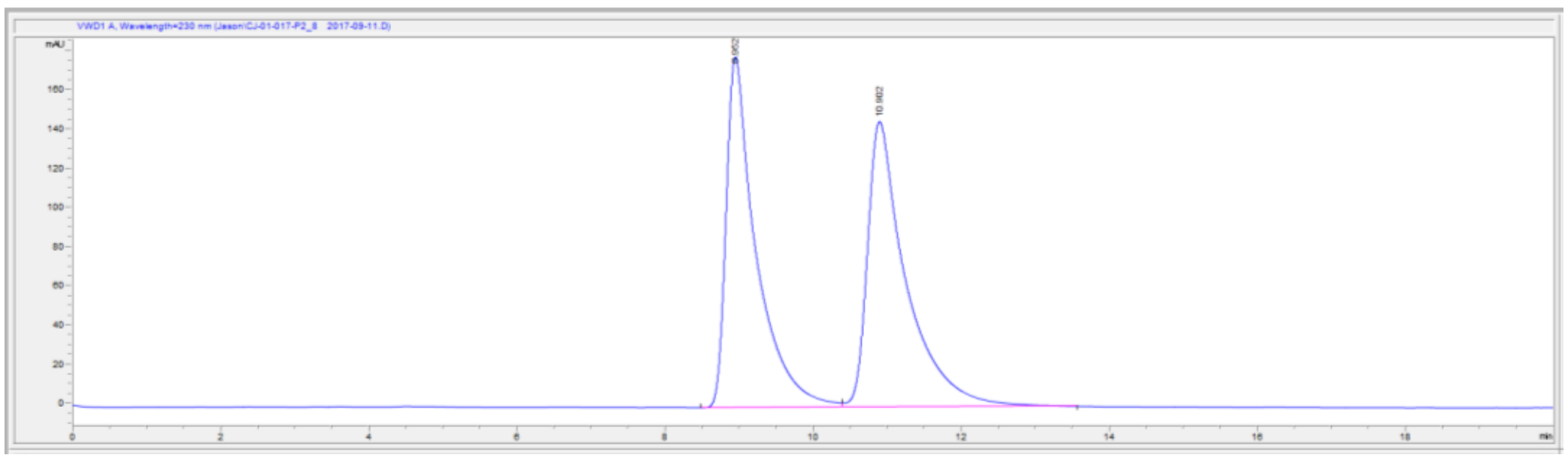

#	Time	Type	Area	Height	Width	Area%	Symmetry
1	8.952	BV	5030.1	179	0.3871	48.830	0.412
2	10.902	VB	5271.1	145.6	0.4947	51.170	0.432

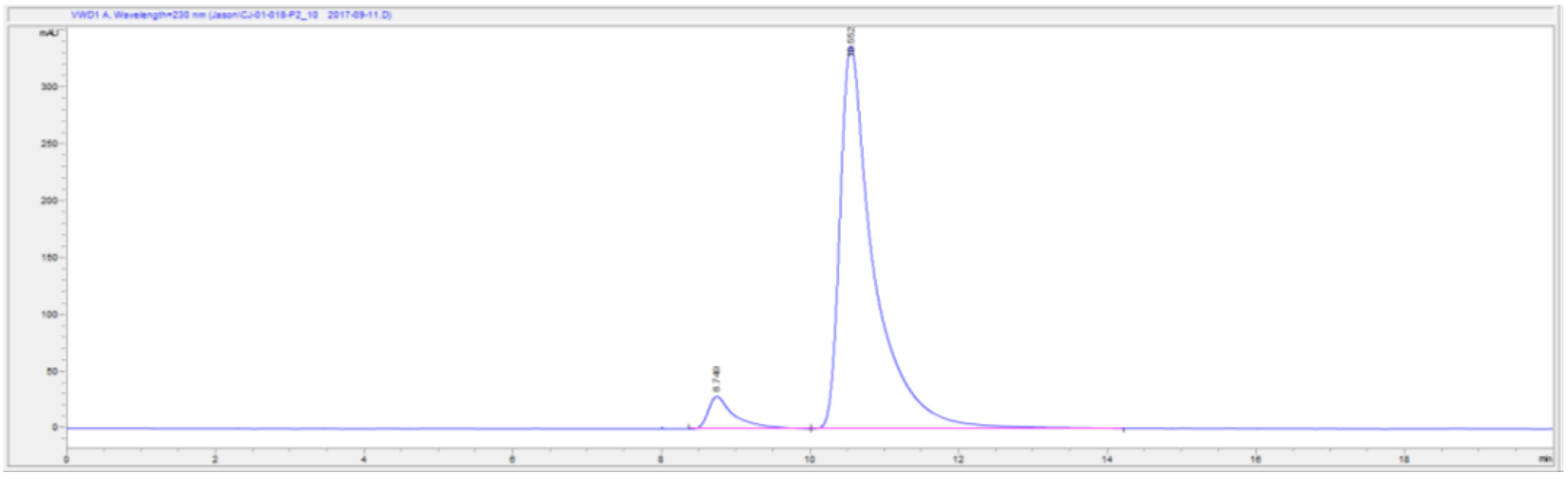

#	Time	Type	Area	Height	Width	Area%	Symmetry
1	8.749	BB	688.2	28.4	0.3398	6.057	0.461
2	10.552	BB	10672.5	336	0.4521	93.943	0.427

(2*S*,3*S*)-3-(*tert*-Butyldimethylsilyloxy)-2-methyl-5-(triethylsilyl)pent-4-ynyl trifluoromethanesulfonate (15)

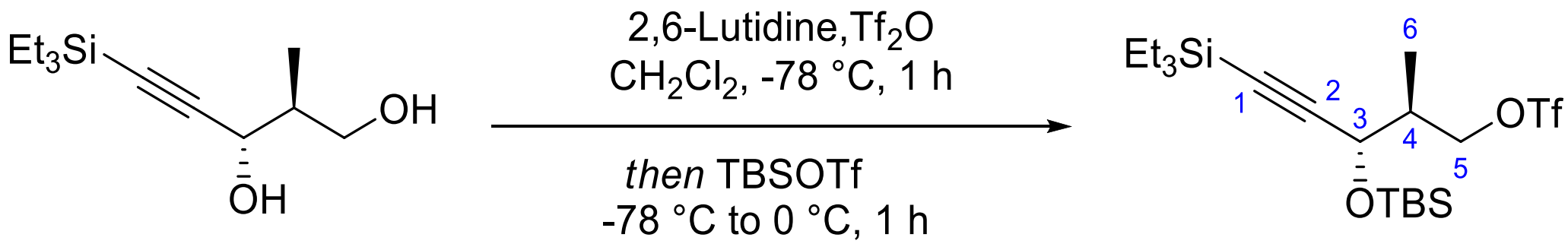

14 76% 15

A solution of Tf_2O (8.00 mL, 13.5 g, 47.7 mmol, 1.1 eq.) in dry CH_2Cl_2 (100 mL) and a solution of 2,6-lutidine (25.3 mL, 23.3 g, 217 mmol, 5.0 eq.) in dry CH_2Cl_2 (100 mL) were added to a solution of **14** (9.92 g, 43.4 mmol, 1.0 eq.) in dry CH_2Cl_2 (220 mL) separately within 20 min at -78 °C. The resulting reaction mixture was stirred at -78 °C for another 40 min. Then TBSOTf (20.0 mL, 22.9 g, 86.8 mmol, 2.0 eq.) was added to the above reaction mixture dropwise over 10 min at -78 °C. After stirring at 0 °C for another 1 h, the reaction was quenched by addition of saturated NH_4Cl and extracted with Et_2O (2 × 400 mL). The organic phases were combined, washed with brine, dried over $MgSO_4$, filtered and concentrated in *vacuo*. The crude product was purified by flash column chromatography on silica gel (Hexanes/EtOAc = 100:0 to 96:4) to give pure **15** as a pale pink oil (15.7 g, 33.0 mmol, 76%).

$\boldsymbol{R_f}$ = 0.52 (Hexanes/EtOAc = 96:4)

IR (thin film) $\boldsymbol{\nu_{max}}$/cm^{-1}: 2956, 2930, 2876, 2857, 2169, 1462, 1416, 1378, 1361, 1212, 1148, 1080, 1018, 981, 938, 836, 777, 726.

^{1}H NMR (400 MHz, $CDCl_3$) δ_H = 4.68 (dd, J = 9.6, 5.0 Hz, 1H, $\boldsymbol{H_A}C_5$), 4.53 (dd, J = 9.6, 6.4 Hz, 1H, $\boldsymbol{H_B}C_5$), 4.35 (d, J = 6.1 Hz, 1H, $\boldsymbol{H}C_3$), 2.25 – 2.15 (m, 1H, $\boldsymbol{H}C_4$), 1.13 (d, J = 6.9 Hz,

3H, H_3C_6), 0.99 (t, J = 7.9 Hz, 9H, Si(CH_2CH_3)$_3$), 0.90 (s, 9H, SiC(CH_3)$_3$), 0.60 (q, J = 7.9 Hz, 6H, Si(CH_2CH_3)$_3$), 0.16 (s, 3H, SiCH_3), 0.12 (s, 3H, SiCH_3).

^{13}C NMR (100 MHz, $CDCl_3$) δ_C = 118.8 (d, $^1J_{CF}$ = 319.6 Hz, CF_3), 105.6 (**C_2**), 88.8 (**C_1**), 78.8 (**C_5**), 64.4 (**C_3**), 40.7 (**C_4**), 25.8 (SiC(CH_3)$_3$), 18.3 (Si*C*(CH_3)$_3$), 12.7 (**C_6**), 7.5 (Si(CH_2CH_3)$_3$), 4.4 (Si(CH_2CH_3)$_3$), -4.4 (SiCH_3), -5.2 (SiCH_3).

HRMS (ESI^+) calculated for $C_{19}H_{37}F_3NaO_4SSi_2$ [$M+Na^+$] = 497.1795, found 497.1796.

$[\alpha]_D^{23}$ = –27.0 (*c* 1.00, $CHCl_3$)

***tert*-Butyldimethyl((3*S*,4*S*)-4-methyl-1-(triethylsilyl)octa-1,6-diyn-3-yloxy)silane (16)**

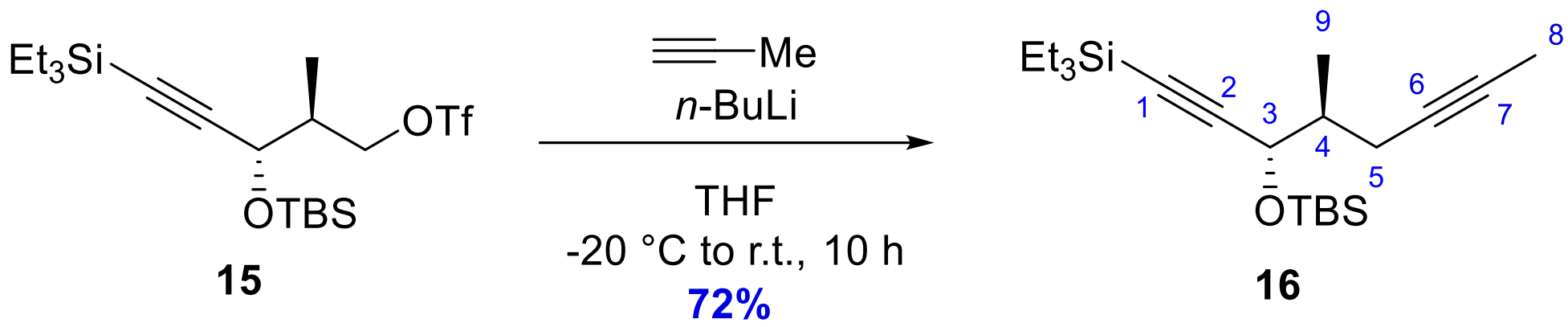

Propyne (11.6 mL, 6.14 g, 153 mmol, 5.0 eq.) was condensed in flame dried Schlenk flask at -78 °C and diluted with dry THF (185 mL). To this stirring solution was added dropwise *n*-BuLi (1.6 M in hexanes, 99.6 mL, 159 mmol, 5.2 eq.) and the reaction mixture was stirred at -78 °C for 1.5 h. The reaction mixture was warmed to -20 °C, and a solution of triflate **15** (14.6 g, 30.7 mmol, 1.0 eq.) in THF (247 mL) was added dropwise. Then the reaction mixture was warmed to r.t. and stirred at r.t. for 12 h. The reaction mixture was carefully quenched by addition of water (100 mL) and extracted with Et_2O (2 × 300 mL). The organic phases were combined, washed with brine, dried over $MgSO_4$, filtered and concentrated in *vacuo*. The crude product was purified by flash column chromatography on silica gel (Hexanes) to give pure **16** as colourless oil (8.04 g, 22.1 mmol, 72%).

R_f = 0.46 (Hexanes)

IR (thin film) ν_{max}/cm^{-1}: 2956, 2930, 2876, 2857, 2169, 1462, 1416, 1250, 1212, 1148, 1080, 1018, 981, 938, 836, 726.

^{1}H NMR (400 MHz, $CDCl_3$) δ_H = 4.28 (d, J = 6.7 Hz, 1H, HC_3), 2.31 – 2.24 (m, 1H, H_AC_5), 2.21 – 2.13 (m, 1H, H_BC_5), 1.87 – 1.81 (m, 1H, HC_4), 1.78 (t, J = 2.6 Hz, 3H, H_3C_8), 1.06 (d, J = 6.8 Hz, 3H, H_3C_9), 0.98 (t, J = 7.9 Hz, 9H, Si(CH_2CH_3)$_3$), 0.90 (s, 9H, SiC(CH_3)$_3$), 0.58 (q, J = 7.9 Hz, 6H, Si(CH_2CH_3)$_3$), 0.14 (s, 3H, SiCH_3), 0.11 (s, 3H, SiCH_3).

^{13}C NMR (100 MHz, $CDCl_3$) δ_C = 107.6 (**C_2**), 87.1 (**C_1**), 77.4 (**C_6**), 76.8 (**C_7**), 66.9 (**C_3**), 40.2 (**C_4**), 25.9 (SiC(CH_3)$_3$), 22.0 (**C_5**), 18.4 (Si*C*(CH_3)$_3$), 15.3 (**C_9**), 7.6 (Si(CH_2CH_3)$_3$), 4.5 (Si(CH_2CH_3)$_3$), 3.6 (**C_8**), -4.4 (SiCH_3), -5.0 (SiCH_3).

HRMS (ESI^+) calculated for $C_{21}H_{40}NaOSi_2$ [$M+Na^+$] = 387.2510, found 387.2508.

$[\alpha]_D^{18}$ = –24.0 (*c* 1.00, $CHCl_3$)

***tert*-Butyldimethyl((3*S*,4*S*)-4-methylocta-1,6-diyn-3-yloxy)silane (17)**

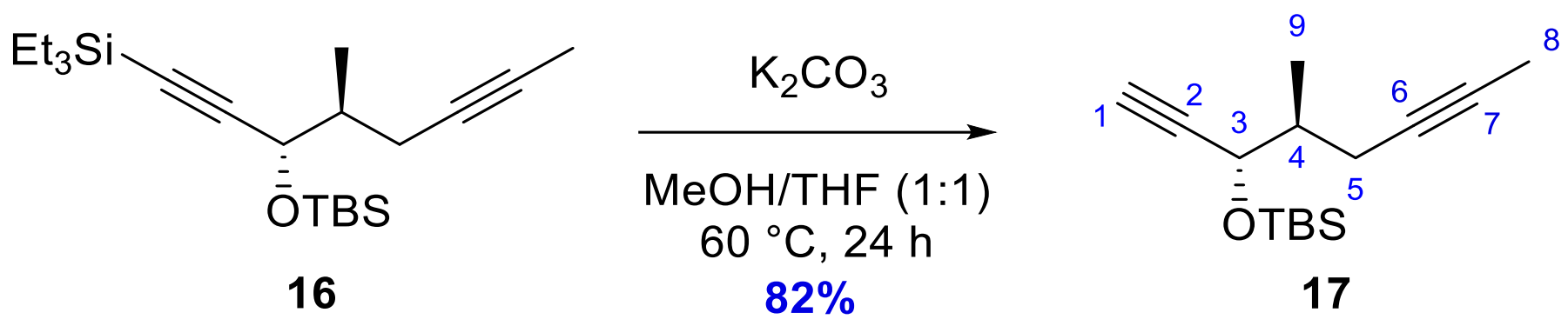

To a solution of **16** (4.15 g, 11.4 mmol, 1.0 eq.) in wet THF/MeOH (228 mL, 1:1 *v*/*v*) was added K_2CO_3 (15.7 g, 114 mmol, 10.0 eq.) at r.t.. After stirring at 60 °C for 24 h, the reaction solvents were removed under reduced pressure. The reaction mixture was added water (50 mL) and extracted with EtOAc (3 × 50 mL). The organic phases were combined, washed with brine, dried over $MgSO_4$, filtered and concentrated in *vacuo*. The crude product was purified by flash column chromatography on silica gel (Hexanes/EtOAc = 100:0 to 99:1) to give pure **17** as a colourless oil (2.35 g, 9.38 mmol, 82%).

R_f = 0.43 (Hexanes)

IR (thin film) ν_{max}/cm^{-1}: 3310, 2957, 2929, 2857, 1472, 1462, 1361, 1252, 1081, 1028, 1006, 836, 777.

^{1}H NMR (400 MHz, $CDCl_3$) δ_H = 4.32 (dd, *J* = 6.4, 2.1 Hz, 1H, ***H***C_3), 2.37 (d, *J* = 2.1 Hz, 1H, ***H***C_1), 2.30 – 2.22 (m, 1H, $H_A C_5$), 2.21 – 2.14 (m, 1H, $H_B C_5$), 1.89 – 1.83 (m, 1H, ***H***C_4), 1.77 (t, *J* = 2.6 Hz, 3H, H_3C_8), 1.06 (d, *J* = 6.8 Hz, 3H, H_3C_9), 0.90 (s, 9H, $Si(CH_2CH_3)_3$), 0.14 (s, 3H, $SiCH_3$), 0.11 (s, 3H, $SiCH_3$).

^{13}C NMR (100 MHz, $CDCl_3$) δ_C = 84.2 (**C_2**), 77.3 (**C_1**), 77.1 (**C_6**), 73.3 (**C_7**), 66.2 (**C_3**), 40.1 (**C_4**), 25.9 ($SiC(CH_3)_3$), 22.0 (**C_5**), 18.3 ($SiC(CH_3)_3$), 15.1 (**C_9**), 3.6 (**C_8**), -4.5 ($SiCH_3$), -5.1 ($SiCH_3$).

HRMS (ESI^+) calculated for $C_{15}H_{27}OSi$ [$M+H^+$] = 251.1826, found 251.1820.

$[\alpha]_D^{18}$ = –24.0 (*c* 1.00, $CHCl_3$)

***tert*-Butyl((3*S*,4*S*,*E*)-1-iodo-4-methyloct-1-en-6-yn-3-yloxy)dimethylsilane (18)**

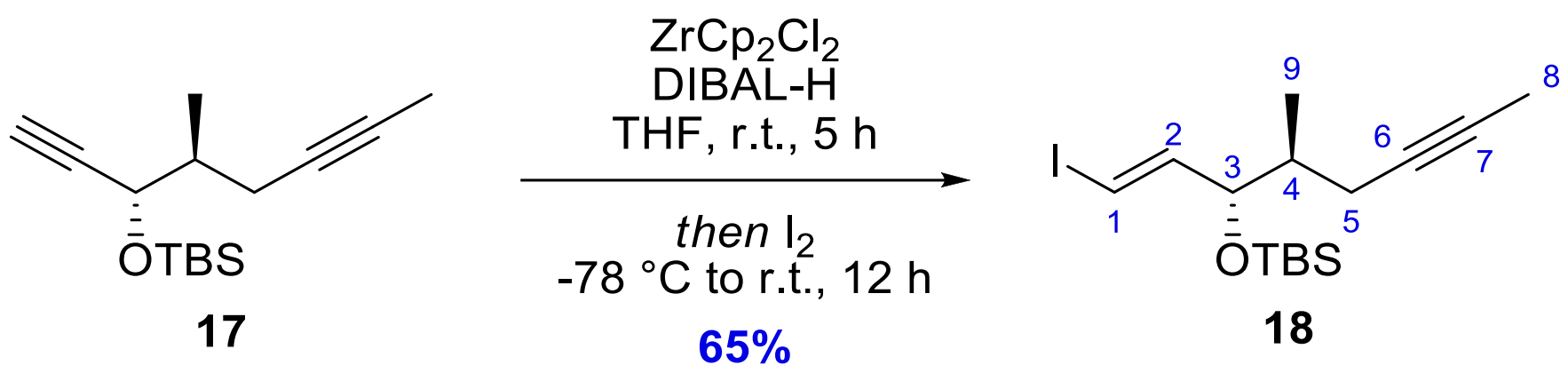

The product is light-sensitive. Therefore, the reaction flask was always covered in aluminium foil and was handled in the fume hood when the light was switched off. DIBAL-H (1.0 M in hexanes, 2.16 mL, 2.16 mmol, 1.2 eq.) was added dropwise to a stirring suspension of $ZrCp_2Cl_2$ (684 mg, 2.34 mmol, 1.3 eq.) in dry THF (3.6 mL) at 0 °C and the resulting suspension was stirred for 1 h at 0 °C before a solution of alkyne **17** (451 mg, 1.80 mmol, 1.0 eq.) in dry THF (1.3 mL) was added dropwise. The mixture stirred for 5 h at r.t. in which time it became a yellow solution before it was cooled to -78 °C. Then a solution of iodine (548 mg, 2.16 mmol, 1.2 eq.) in THF (1.3 mL) was added and the mixture was stirred overnight (12 h) while warming from -78 °C to r.t. The reaction was quenched by addition of H_2O, and stirred for 15 min before it was poured over saturated $Na_2S_2O_3$ and $NaHCO_3$ solution (2:1, 20 mL) and extracted with EtOAc (3 × 20 mL). The organic phases were combined, washed with brine, dried over $MgSO_4$, filtered and concentrated in *vacuo*. The crude product was purified by flash column chromatography on silica gel (Hexanes) to give pure **18** as a colourless oil (440 mg, 1.16 mmol, 65%).

$\boldsymbol{R_f}$ = 0.33 (Hexanes)

IR (thin film) $\boldsymbol{\nu_{max}}$/cm^{-1}: 2955, 2928, 2856, 1607, 1471, 1462, 1361, 1253, 1166, 1064, 1029, 951, 835, 775.

^{1}H NMR (400 MHz, C_6D_6) δ_H = 6.41 (dd, J = 14.5, 7.1 Hz, 1H, ***H***C_1), 6.02 (dd, J = 14.5, 0.8 Hz, 1H, ***H***C_2), 3.85 (t, J = 6.7 Hz, 1H, ***H***C_3), 2.17 – 2.09 (m, 2H, $\boldsymbol{H_A}C_5$+$\boldsymbol{H_B}C_5$), 1.64 – 1.59 (m, 1H, ***H***C_4), 1.57 (t, J = 2.6 Hz, 3H, $\boldsymbol{H_3}C_8$), 0.91 (s, 9H, Si($CH_2C\boldsymbol{H_3}$)$_3$), 0.86 (d, J = 6.9 Hz, 3H, $\boldsymbol{H_3C_9}$), 0.02 (s, 3H, Si$C\boldsymbol{H_3}$), -0.01 (s, 3H, Si$C\boldsymbol{H_3}$).

^{13}C NMR (100 MHz, C_6D_6) δ_C = 147.7 (**C$_1$**), 78.5 (**C$_2$**), 77.6 (**C$_6$**), 77.3 (**C$_7$**), 77.1 (**C$_3$**), 39.3 (**C$_4$**), 26.2 (SiC(***C***H_3)$_3$), 22.3 (**C$_5$**), 18.4 (Si***C***(CH_3)$_3$), 15.3 (**C$_9$**), 3.4 (**C$_8$**), -4.2 (Si***C***H_3), -4.9 (Si***C***H_3).

HRMS (ESI$^+$) calculated for $C_{15}H_{27}INaOSi$ [M+Na$^+$] = 401.0768, found 401.0777.

$[\alpha]_D^{18}$ = +6.0 (*c* 0.82, $CHCl_3$)

Synthesis of (*R*)-*tert*-butyldimethyl(oct-1-yn-3-yloxy)silane (S8) from S3

(*R*)-*tert*-Butyldimethyl(oct-1-yn-3-yloxy)silane (S8)

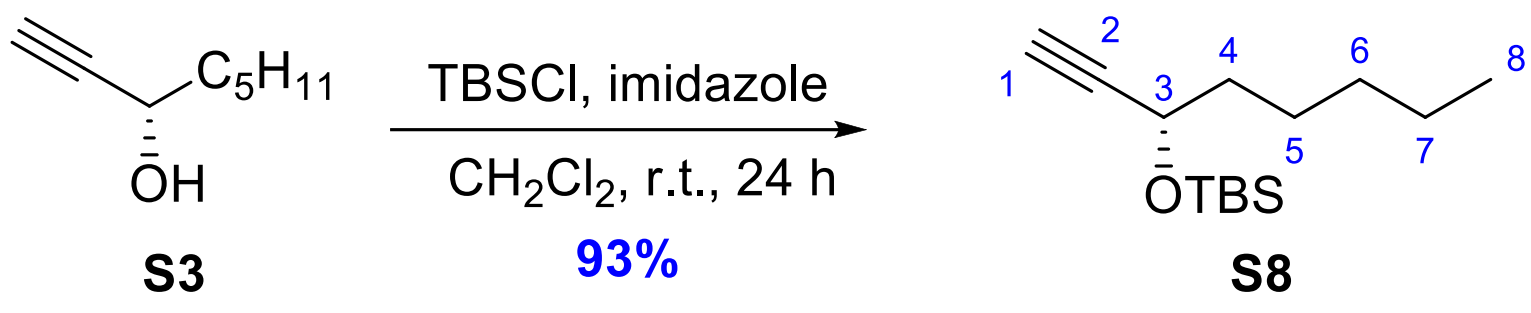

Imidazole (3.68 g, 54.0 mmol, 1.8 eq.) and TBSCl (5.43 g, 36.0 mmol, 1.2 eq.) were sequentially added to a stirring solution of **S3** (3.79 g, 30.0 mmol, 1.0 eq.) in CH_2Cl_2 (60 mL) at 0 °C. The reaction mixture was stirred at r.t. for 24 h before it was poured over aqueous HCl (1.0 M, 140 mL) and extracted with pentane (3 × 140 mL). The organic phases were combined, washed with brine, dried over $MgSO_4$, filtered and concentrated *in vacuo*. The crude product was purified by flash column chromatography on silica gel (Hexanes) to give pure **S8** as a clear, colourless oil (6.71 g, 27.9 mmol, 93%).

***R*f** = 0.31 (Hexanes)
IR (thin film) ν_{max} /cm^{-1}: 3313, 2955, 2929, 2858, 1472, 1464, 1251, 1088, 835, 776.
^{1}H NMR (400 MHz, $CDCl_3$) δ_H = 4.33 (td, *J* = 6.5, 2.1 Hz, 1H, ***H***C3), 2.37 (d, *J* = 2.1 Hz, 1H, ***H***C1), 1.70 – 1.64 (m, 2H, ***H*2**C4), 1.46 – 1.38 (m, 2H, ***H*2**C5), 1.36 – 1.26 (m, 4H, ***H*2**C6+***H*2**C7), 0.92 – 0.87 (m, 12H, SiC(C***H*3**)3+***H*3**C8), 0.13 (s, 3H, SiC***H*3**), 0.11 (s, 3H, SiC***H*3**).
^{13}C NMR (101 MHz, $CDCl_3$) δ_C = 86.0 (**C2**), 72.0 (**C1**), 63.0 (**C3**), 38.7 (**C4**), 31.6 (**C6**), 25.9 (SiC(***C***H3)3), 24.9 (**C5**), 22.7 (**C7**), 18.4 (Si***C***(CH3)3), 14.1 (**C8**), -4.4 (Si***C***H3), -4.9 (Si***C***H3).
***m*/*z* (CI)** 241 (M+H$^+$, 62%), 225 (88%), 133 ($^+H_2OSi(CH_3)_2C(CH_3)_3$, 93%), 109 (M+H$^+$ – $HOSi(CH_3)_2C(CH_3)_3$, 100%).
[*α*]D^{24} = −30.3 (*c* 1.00, $CHCl_3$)

Synthesis of *tert*-butyldimethyl(((3*S*,4*S*)-4-methylocta-1,6-diyn-3-yl)oxy)silane (20) from 15

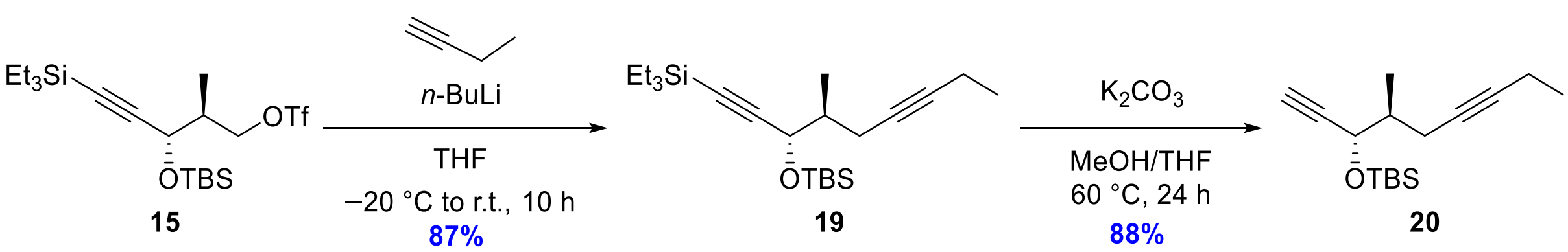

***tert*-Butyldimethyl((3*S*,4*S*)-4-methyl-1-(triethylsilyl)nona-1,6-diyn-3-yloxy)silane (19)**

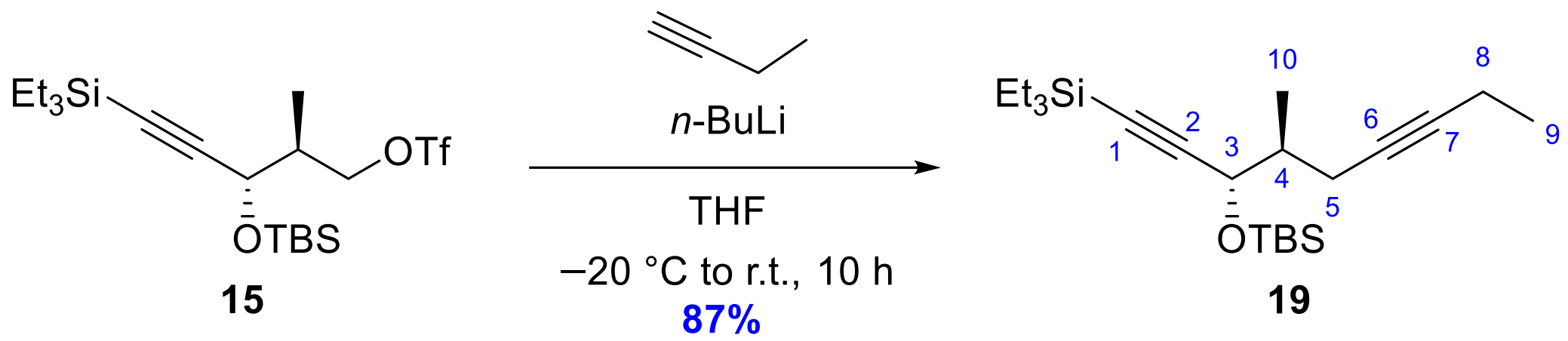

But-1-yne (9.53 mL, 6.44 g, 119 mmol, 5.0 eq.) was condensed in flame dried Schlenk flask at -78 °C and diluted with dry THF (144 mL). To this stirring solution was added dropwise *n*-BuLi (1.6 M in hexanes, 77.4 mL, 124 mmol, 5.2 eq.) and the reaction mixture was stirred at -78 °C for 1.5 h. The reaction mixture was warmed to -20 °C, and a solution of triflate **15** (11.3 g, 23.8 mmol, 1.0 eq.) in THF (192 mL) was added dropwise. Then the reaction mixture was warmed to r.t. and stirred at r.t. for 10 h. The reaction mixture was carefully quenched by addition of water (80 mL) and extracted with Et_2O (2 × 340 mL). The organic phases were combined, washed with brine, dried over $MgSO_4$, filtered and concentrated in *vacuo*. The crude product was purified by flash column chromatography on silica gel (Hexanes/EtOAc = 100:0 to 98:2) to give pure **19** as a colourless oil (7.85 g, 22.7 mmol, 87%).

$\boldsymbol{R_f}$ = 0.40 (Hexanes/EtOAc = 99:1)

IR (thin film) ν_{max}/cm^{-1}: 2955, 2930, 2876, 2857, 2169, 1461, 1414, 1251, 1078, 1018, 982, 938, 835, 724.

^{1}H NMR (400 MHz, $CDCl_3$) δ_H = 4.29 (d, *J* = 6.8 Hz, 1H, ***H***C_3), 2.32 – 2.21 (m, 2H, $\boldsymbol{H_A}C_5$+$\boldsymbol{H_B}C_5$), 2.19 – 2.12 (m, 2H, $\boldsymbol{H_2}C_8$), 1.89 – 1.79 (m, 1H, ***H***C_4), 1.11 (t, *J* = 7.5 Hz, 3H, $\boldsymbol{H_3}C_9$), 1.06 (d, *J* = 6.8 Hz, 3H, $\boldsymbol{H_3}C_{10}$), 0.99 (t, *J* = 7.9 Hz, 9H, $Si(CH_2C\boldsymbol{H_3})_3$), 0.90 (s, 9H, $SiC(C\boldsymbol{H_3})_3$), 0.59 (q, *J* = 7.9 Hz, 6H, $Si(C\boldsymbol{H_2}CH_3)_3$), 0.14 (s, 3H, $SiC\boldsymbol{H_3}$), 0.11 (s, 3H, $SiC\boldsymbol{H_3}$).

^{13}C NMR (100 MHz, $CDCl_3$) δ_C = 107.6 (**C_2**), 87.0 (**C_1**), 83.1 (**C_7**), 77.5 (**C_6**), 66.9 (**C_3**), 40.2 (**C_4**), 25.9 ($SiC(\boldsymbol{C}H_3)_3$), 22.0 (**C_5**), 18.4 ($Si\boldsymbol{C}(CH_3)_3$), 15.2 (**C_{10}**), 14.5 (**C_9**), 12.6 (**C_8**), 7.6 ($Si(CH_2\boldsymbol{C}H_3)_3$), 4.5 ($Si(\boldsymbol{C}H_2CH_3)_3$), -4.4 ($Si\boldsymbol{C}H_3$), -5.0 ($Si\boldsymbol{C}H_3$).

HRMS (ESI^+) calculated for $C_{22}H_{42}NaOSi_2$ [$M+Na^+$] = 401.2666, found 401.2667.

$[\alpha]_D^{24}$= –21.0 (*c* 1.00, $CHCl_3$)

***tert*-Butyldimethyl((3*S*,4*S*)-4-methylnona-1,6-diyn-3-yloxy)silane (20)**

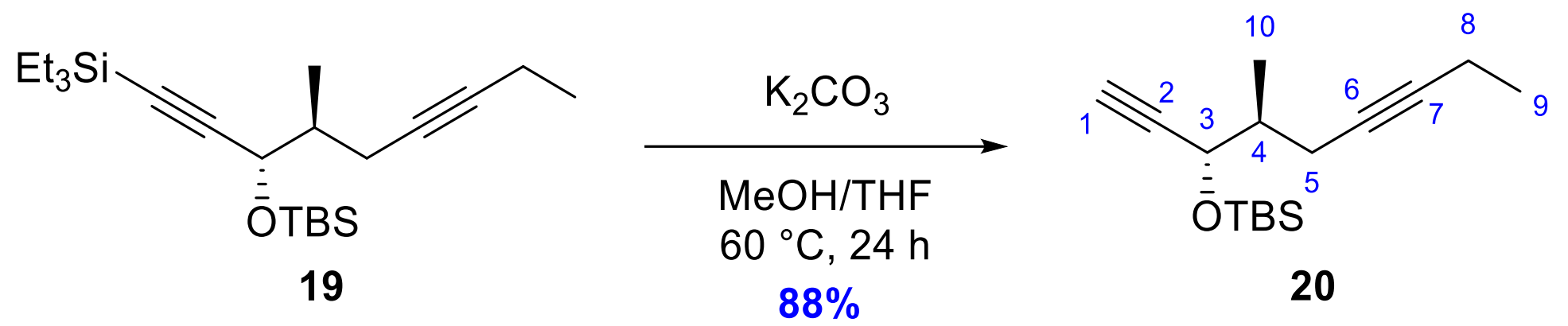

To a solution of **19** (1.29 g, 3.40 mmol, 1.0 eq.) in wet THF/MeOH (68 mL, 1:1 *v*/*v*) was added K_2CO_3 (4.70 g, 34.0 mmol, 10.0 eq.) at r.t.. After stirring at r.t. for 24 h, the reaction solvents were removed under reduced pressure. The reaction mixture was added water (15 mL) and extracted with EtOAc (3 × 15 mL). The organic phases were combined, washed with brine, dried over $MgSO_4$, filtered and concentrated in *vacuo*. The crude product was purified by flash column chromatography on silica gel (Hexanes/EtOAc = 100:0 to 98:2) to give pure **20** as a colourless oil (0.79 g, 2.98 mmol, 88%).

$\boldsymbol{R_f}$ = 0.38 (Hexanes/EtOAc = 99:1)

IR (thin film) $\boldsymbol{\nu_{max}}$/cm^{-1}: 3311, 2957, 2929, 2857, 1715, 1462, 1251, 1080, 1028, 1005, 901, 835, 776.

^{1}H NMR (400 MHz, $CDCl_3$) δ_H = 4.34 (dd, *J* = 6.4, 2.1 Hz, 1H, ***H***C_3), 2.37 (d, *J* = 2.1 Hz, 1H, ***H***C_1), 2.31 – 2.21 (m, 2H, $\boldsymbol{H_A}C_5$+$\boldsymbol{H_B}C_5$), 2.20 – 2.12 (m, 2H, $\boldsymbol{H_2}C_8$), 1.91 – 1.81 (m, 1H, ***H***C_4), 1.11 (t, *J* = 7.5 Hz, 3H, $\boldsymbol{H_3}C_9$), 1.06 (d, *J* = 6.8 Hz, 3H, $\boldsymbol{H_3}C_{10}$), 0.90 (s, 9H, SiC(C$\boldsymbol{H_3}$)$_3$), 0.14 (s, 3H, SiC$\boldsymbol{H_3}$), 0.11 (s, 3H, SiC$\boldsymbol{H_3}$).

^{13}C NMR (100 MHz, $CDCl_3$) δ_C = 84.2 (**C$_2$**), 83.2 (**C$_7$**), 77.4 (**C$_6$**), 73.2 (**C$_1$**), 66.1 (**C$_3$**), 40.1 (**C$_4$**), 25.9 (SiC(***C***H$_3$)$_3$), 22.0 (**C$_5$**), 18.3 (Si***C***(CH$_3$)$_3$), 15.0 (**C$_{10}$**), 14.5 (**C$_9$**), 12.6 (**C$_8$**), -4.5 (Si***C***H$_3$), -5.1 (Si***C***H$_3$).

HRMS (ESI$^+$) calculated for $C_{16}H_{28}NaOSi$ [M+Na$^+$] = 287.1802, found 287.1800.

[*α*]$_D$^{23} = –19.0 (*c* 1.00, $CHCl_3$)

Synthesis of α-monofluorinated model lactone 45

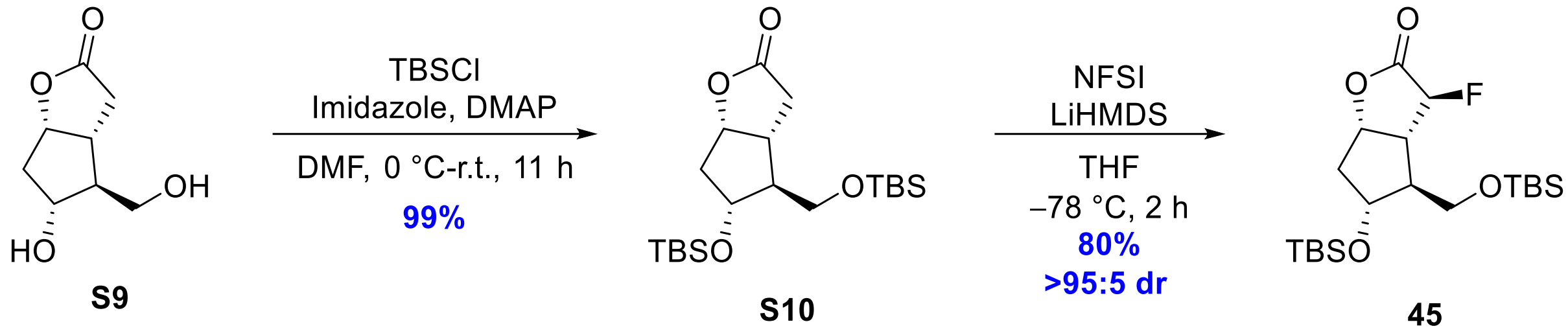

(3a*R*,4*S*,5*R*,6a*S*)-5-(*tert*-Butyldimethylsilyloxy)-4-((*tert*-butyldimethylsilyloxy)methyl)hexahydro-2*H*-cyclopenta[*b*]furan-2-one (S10)

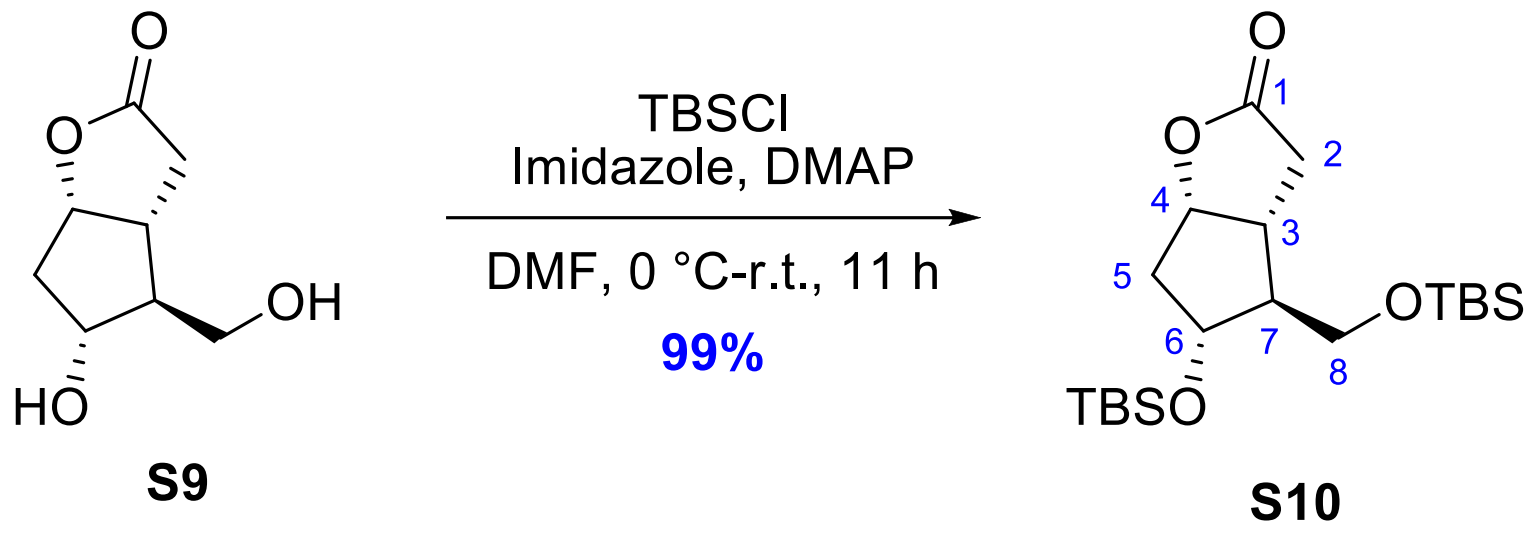

To a solution of (3a*R*,4*S*,5*R*,6a*S*)-5-hydroxy-4-(hydroxymethyl)hexahydro-2*H*-cyclopenta[*b*]furan-2-one (**S9**) (1.89 g, 11.0 mmol, 1.0 eq.) in anhydrous DMF (18.0 mL), imidazole (4.49 g, 66.0 mmol, 6.0 eq.), TBSCl (3.98 g, 26.4 mmol, 2.4 eq.) and DMAP (269 mg, 2.20 mmol, 0.2 eq.) were sequentially added at 0 °C. The reaction mixture was warmed to r.t. and stirred for 11 h. Saturated brine solution (90 mL) was added and the resulting mixture was stirred for 5 minutes. The solution was diluted with EtOAc (90 mL) and the aqueous phase was extracted with EtOAc (3 × 90 mL). The organic phases were combined, washed with brine (2 × 60 mL), dried over $MgSO_4$, filtered and concentrated in *vacuo*. The crude product was purified by flash column chromatography on silica gel (Hexanes/EtOAc = 90:10 to 80:20) to give pure **S10** as a white, amorphous solid (4.36 g, 10.9 mmol, 99%).

Mp 100–104 ˚C

R_f = 0.19 (Hexanes/EtOAc = 90:10)

IR (thin film) ν_{max}/cm^{-1}: 2952, 2928, 2885, 2855, 1752, 1471, 1251, 1089, 1033, 1005, 951, 858, 775.

^{1}H NMR (400 MHz, $CDCl_3$) δ_H = 4.92 (td, *J* = 7.1, 2.3 Hz, 1H, ***H***C4), 4.12 (dd, *J* = 10.3, 4.8 Hz, 1H, ***H***C6), 3.55 (dd, *J* = 10.3, 5.3 Hz, 1H, ***H$_A$***C8), 3.48 (dd, *J* = 10.3, 5.6 Hz, 1H, ***H$_B$***C8), 2.77 (dd, *J* = 17.8, 10.5 Hz, 1H, ***H$_A$***C2), 2.69 – 2.63 (m, 1H, ***H***C3), 2.53 (dd, *J* = 17.8, 2.8 Hz, 1H, ***H$_B$***C2), 2.23 (ddd, *J* = 14.8, 6.9, 5.8 Hz, 1H, ***H$_A$***C5), 2.00 – 1.95 (m, 2H, ***H$_B$***C5+ ***H***C7), 0.88 (s, 9H, SiC(C***H$_3$***)$_3$), 0.87 (s, 9H, SiC(C***H$_3$***)$_3$), 0.05 (s, 6H, SiC***H$_3$***+SiC***H$_3$***), 0.04 (s, 3H, SiC***H$_3$***), 0.04 (s, 3H, SiC***H$_3$***).

^{13}C NMR (100 MHz, $CDCl_3$) δ_C = 177.4 (**C$_1$**), 84.3 (**C$_4$**), 74.8 (**C$_6$**), 62.7 (**C$_8$**), 57.1 (**C$_7$**), 41.2 (**C$_5$**), 39.3 (**C$_3$**), 35.8 (**C$_2$**), 26.0 (SiC(*C*H$_3$)$_3$), 25.9 (SiC(*C*H$_3$)$_3$), 18.4 (Si*C*(CH$_3$)$_3$), 18.0 (Si*C*(CH$_3$)$_3$), -4.6 (Si*C*H$_3$), -4.9 (Si*C*H$_3$), -5.3 (Si*C*H$_3$), -5.4 (Si*C*H$_3$).
HRMS (ESI$^+$) calculated for $C_{20}H_{40}NaO_4Si_2$ [M+Na$^+$] = 423.2357, found 423.2349.
$[\alpha]_D^{25}$ = –30.0 (*c* 1.00, $CHCl_3$)

(3*S*,3a*R*,4*S*,5*R*,6a*S*)-5-(*tert*-Butyldimethylsilyloxy)-4-((*tert*-butyldimethylsilyloxy)methyl)-3-fluorohexahydro-2*H*-cyclopenta[*b*]furan-2-one (45)

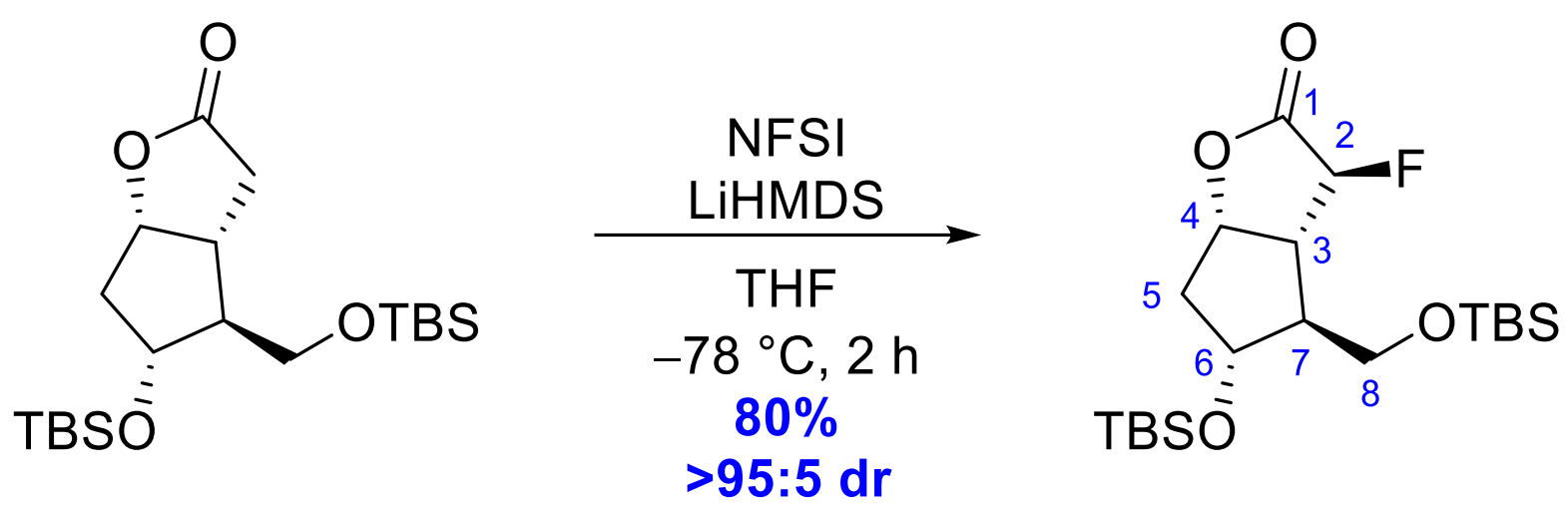

S10 **45**

To a mixture of **S10** (300 mg, 0.75 mmol, 1.0 eq.) and NFSI (272 mg, 0.86 mmol, 1.15 eq.) in THF (3.75 mL) at –78 °C, LiHMDS (1.0 M in THF, 0.86 mL, 0.86 mmol, 1.15 eq.) was added dropwise. The reaction mixture was stirred at –78 °C for 2 h before it was warmed to r.t., quenched by addition of saturated $NaHCO_3$ solution (5 mL) and the aqueous phase was extracted with Et_2O (3 × 10 mL). The organic phases were combined, washed with brine, dried over $MgSO_4$, filtered and concentrated in *vacuo*. The crude product was purified by flash column chromatography on silica gel (Hexanes/EtOAc = 95:5 to 90:10) to give pure **45** as a white, amorphous solid (251 mg, 0.60 mmol, 80%, > 95:5 dr).

Mp 38–41 °C
***R*$_f$** = 0.30 (Hexanes/Et_2O = 90:10)
IR (thin film) ν_{max}/cm^{-1}: 2953, 2930, 2895, 2857, 1789, 1471, 1255, 1205, 1093, 1074, 1003, 1008, 835, 785.
^{1}H NMR (400 MHz, $CDCl_3$) δ_H = 5.11 (t, *J* = 6.8 Hz, 1H, ***H***C$_4$), 5.09 (dd, *J* = 51.8, 2.5 Hz, 1H, ***H***C$_2$), 4.23 – 4.20 (m, *J* = 2.1 Hz, 1H, ***H***C$_6$), 3.56 (dd, *J* = 10.3, 5.2 Hz, 1H, ***H$_A$***C$_8$), 3.47 (dd, *J* = 10.3, 6.7 Hz, 1H, ***H$_B$***C$_8$), 2.84 (ddt, *J* = 32.1, 7.4, 2.7 Hz, 1H, ***H***C$_3$), 2.35 – 2.27 (m, 1H, ***H***C$_7$), 2.14 (ddd, *J* = 15.1, 6.9, 4.6 Hz, 1H, ***H$_A$***C$_5$), 2.01 (dd, *J* = 15.1, 1.3 Hz, 1H, ***H$_B$***C$_8$), 0.89 (s, 9H, SiC(C***H$_3$***)$_3$), 0.86 (s, 9H, SiC(C***H$_3$***)$_3$), 0.05 (s, 6H, SiC***H$_3$***+ SiC***H$_3$***), 0.05 – 0.03 (s, 6H, SiC***H$_3$***+ SiC***H$_3$***).
^{19}F NMR (377 MHz, $CDCl_3$) δ_F = −177.95 (dd, $^2J_{HF}$ = 51.8, $^3J_{HF}$ = 32.1 Hz).

^{13}**C NMR** (100 MHz, $CDCl_3$) δ_C = 172.1 (d, $^2J_{CF}$ = 21.1 Hz, **C$_1$**), 92.4 (d, $^1J_{CF}$ = 183.6 Hz, **C$_2$**), 83.9 (d, $^3J_{CF}$ = 2.5 Hz, **C$_4$**), 76.0 (d, $^4J_{CF}$ = 0.5 Hz, **C$_6$**), 63.3 (**C$_8$**), 55.5 (d, $^3J_{CF}$ = 4.8 Hz, **C$_7$**), 47.5 (d, $^2J_{CF}$ = 20.1 Hz, **C$_3$**), 40.6 (d, $^4J_{CF}$ = 1.3 Hz, **C$_5$**), 26.0 (SiC(*C*H$_3$)$_3$), 25.8 (SiC(*C*H$_3$)$_3$), 18.3 (Si*C*(CH$_3$)$_3$), 18.1 (Si*C*(CH$_3$)$_3$), -4.7 (Si*C*H$_3$), -4.9 (Si*C*H$_3$), -5.4 (Si*C*H$_3$), -5.5 (Si*C*H$_3$).
HRMS (ESI$^+$) calculated for $C_{20}H_{39}FNaO_4Si_2$ [M+Na$^+$] = 441.2263, found 441.2278.
[α]$_D^{25}$ = –41.7 (*c* 1.00, $CHCl_3$)

Synthesis of sulfonyl reagents

Methyl 5-(1-phenyl-1*H*-tetrazol-5-ylsulfonyl)pentanoate (**S11**) was prepared according to literature procedures.[10]

Synthesis of 5-(benzo[*d*]thiazol-2-ylsulfonyl)pentanoic acid (S14)

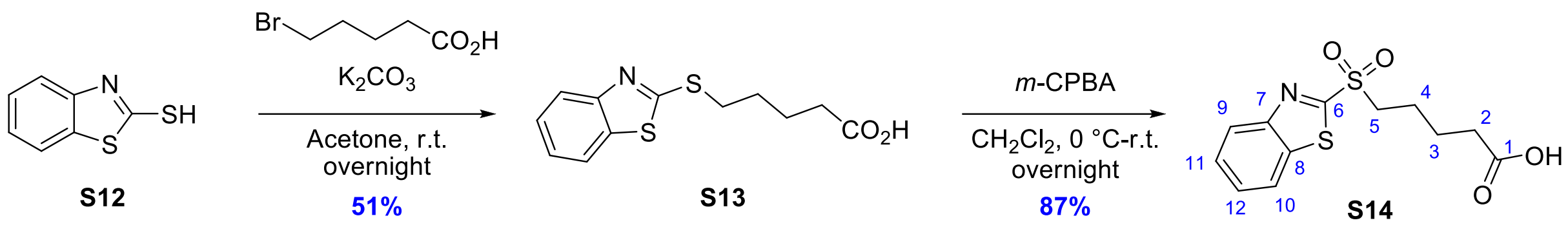

Anhydrous K_2CO_3 (4.15 g, 30.0 mmol, 2.5 eq.) was added to a solution of 5-bromovaleric acid (2.00 g, 12.0 mmol, 1.0 eq.) and mercaptobenzothiazole (2.16 g, 12.0 mmol, 1.0 eq.) in acetone (20.0 mL), and the reaction mixture was stirred at r.t. overnight. HCl (0.5 M in H_2O, 40 mL) was added and the aqueous phase was extracted with CH_2Cl_2 (3 × 40 mL). The organic phases were combined, washed with brine, dried over $MgSO_4$, filtered and concentrated in *vacuo*. The crude product was purified by flash column chromatography on silica gel (pentane/EtOAc/AcOH = 48:50:2) to give pure 5-(benzo[*d*]thiazol-2-ylthio)pentanoic acid (**S13**) as a white powder (1.62 g, 6.06 mmol, 51%).

***R*$_f$** = 0.39 (Pentane/EtOAc = 1:1)

To a solution of **S13** (1.60 g, 5.98 mmol, 1.0 eq.) in CH_2Cl_2 (17.0 mL) at 0 °C, *m*-CPBA (3.10 g, 17.9 mmol, 3.00 eq.) was added. The reaction mixture was warmed to r.t. and stirred at r.t. overnight. The mixture was then poured over HCl (0.5 M in H_2O, 40 mL) and the aqueous phase was extracted with CH_2Cl_2 (3 × 40 mL). The organic phases were combined, washed with brine, dried over $MgSO_4$, filtered and concentrated in *vacuo*. The crude product was purified by flash column chromatography on silica gel (pentane/EtOAc/AcOH = 48:50:2) to give pure **S14** as an off-white solid (1.56 g, 5.21 mmol, 87%).

Mp 120–122 ˚C

$\boldsymbol{R}_f$ = 0.25 (Pentane/EtOAc = 1:1)

IR (thin film) $\boldsymbol{\nu}_{max}$/cm^{-1}: 2967, 1705, 1467, 1315, 1147.

^{1}H NMR (400 MHz, $CDCl_3$) δ_H = 8.23 – 8.21 (m, 1H, ***H***C_9), 8.04 – 8.01 (m, 1H, ***H***C_{10}), 7.67–7.58 (m, 2H, ***H***C_{11}+***H***C_{12}), 3.57 – 3.53 (m, 2H, $\boldsymbol{H_2}C_5$), 2.40 (t, J = 7.2 Hz, 2H, $\boldsymbol{H_2}C_2$), 2.01 – 1.93 (m, 2H, $\boldsymbol{H_2}C_4$), 1.84 – 1.77 (m, 2H, $\boldsymbol{H_2}C_3$).

^{13}C NMR (100 MHz, $CDCl_3$) δ_C = 178.6 (**C_1**), 165.8 (**C_6**), 152.8 (**C_7**), 136.9 (**C_8**), 128.2 (**C_{11}**), 127.9 (**C_{12}**), 125.6 (**C_9**), 122.5 (**C_{10}**), 54.4 (**C_5**), 33.2 (**C_2**), 23.3 (**C_3**), 21.9 (**C_4**).

HRMS (ESI$^+$) calculated for $C_{12}H_{13}NNaO_4S_2$ [M+Na$^+$] = 322.0178, found 322.0180.

General procedure for Julia-Kocienski olefination on α-monofluorinated model lactone

Procedure 1: To a solution of **45** (0.12 mmol, 1.0 eq.) and sulfonyl reagent (0.14 mmol, 1.2 eq.) in THF (0.48 mL) at −78 °C, LiHMDS (1 M in THF, 0.43 mmol, 3.6 eq.) was added dropwise and the resulting bright yellow solution was stirred at –78 °C for 2 h. The reaction mixture was then warmed to r.t. and stirred for a further 2 h. The reaction was quenched by addition of HCl solution (0.5 M in H_2O, 5 mL) and the aqueous phase was extracted with EtOAc (3 × 5 mL). The organic phases were combined, washed with brine, dried over $MgSO_4$, filtered and concentrated in *vacuo*.

Procedure 2: To a solution of **45** (0.12 mmol, 1.0 eq.) and sulfonyl reagent (0.14 mmol, 1.20 eq.) in THF (0.48 mL) at –78 °C, LiHMDS (1.0 M in THF) was added dropwise. The resulting bright yellow solution was stirred at –78 °C for 1 h, quenched by addition of AcOH (1.32 mmol, 11.0 eq.) and warmed to r.t. The reaction mixture was poured over H_2O (1.5 mL) and the aqueous phase was extracted with EtOAc (3 × 3 mL). The organic phases were combined, washed with brine, dried over $MgSO_4$, filtered and concentrated in *vacuo* to give the crude material which was redissolved in corresponding solvent (0.24 ml) prior to dropwise addition of DBU (0.48 mmol, 2.0 eq.). The bright orange reaction mixture was stirred at r.t. for 1 h before H_2O (1.50 mL) was added and the aqueous phase was extracted with EtOAc (3 × 5 mL). The organic phases were combined, washed with brine, dried over $MgSO_4$, filtered and concentrated in *vacuo*.

Table S3. Julia-Kocienski Olefination on α-Monofluorinated Model Lactone.[a,b]

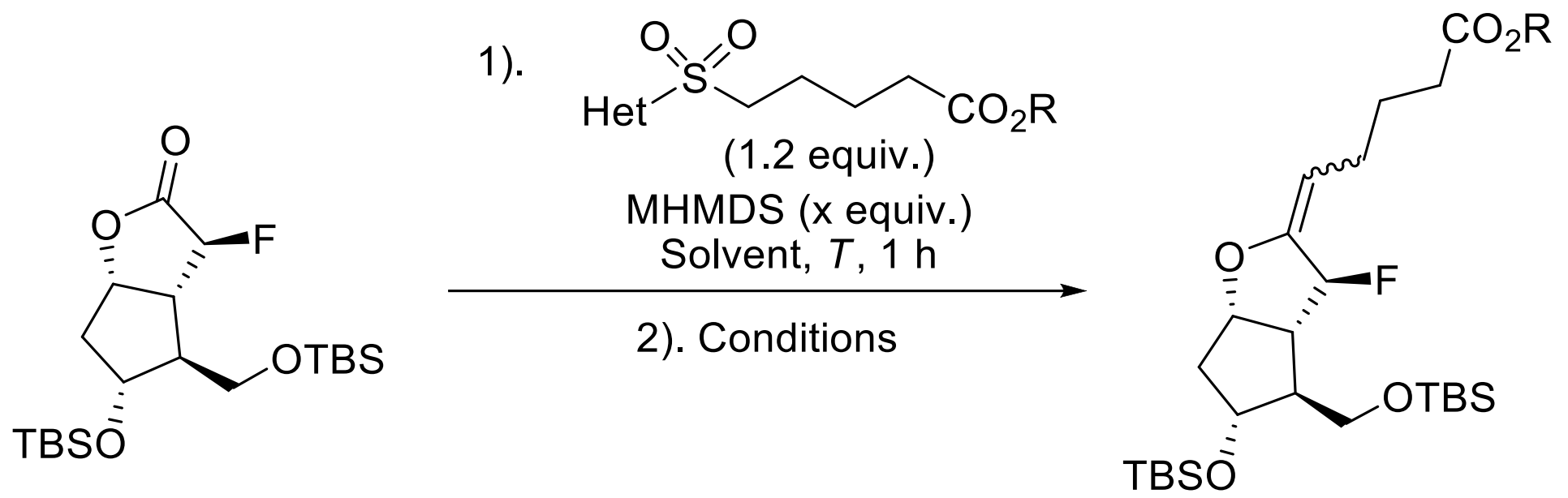

45 **46 or 47**

Entry	Het/R	M (x equiv.)	Solvent	*T* [°C]	Conditions	Yield [%]	Z/E
1	BT/H	Li (3.6)	THF	–78	DBU, THF, r.t., 1 h	67	25:75
2	BT/H	K (3.6)	THF	–78	DBU, THF, r.t., 1 h	< 5	-
3	BT/H	Na (3.6)	THF	–78	DBU, THF, r.t., 1 h	< 5	-
4	BT/H	Li (3.6)	THF	–78	r.t., 2 h	73	33:67
5	PT/H	Li (3.6)	THF	–78	r.t., 2 h	52	33:67
6	PT/H	Li (3.6)	CH_2Cl_2	0	r.t., 2 h	7	57:43
7	PT/Me	Li (3.6)	THF	–78	r.t., 2 h	33	11:89
8	PT/Me	Li (3.6)	CH_2Cl_2	–78	r.t., 2 h	< 5	-
9	PT/Me	Li (3.6)	toluene	–78	r.t., 2 h	8	75:25
10	PT/Me	Li (1.2)	toluene	–78	r.t., 2 h	25	50:50

[a] The reactions were carried out on a 0.12 mmol scale. [b] Yield and *Z*/*E* ratio determined by ^{19}F NMR using α,α,α-trifluorotoluene as an internal standard. BT = Benzothiazole, PT = 1-Phenyl-1*H*-tetrazole.

General procedure for Wittig olefination on α-monofluorinated model lactone.

LiHMDS (1.0 M in THF, 0.20 mmol, 2.0 eq.) was added to a stirring suspension of (4-carboxybutyl)triphenylphosphonium bromide (0.10 mmol, 1.0 eq.) in THF (0.50 ml) at r.t. The resulting orange solution was stirred at r.t. for 1 h before a solution of **45** (0.10 mmol, 1.0 eq.) in THF (0.50 ml) was added to the above orange solution and the reaction mixture was stirred at given temperature. The reaction mixture was quenched by water, and the aqueous phase was extracted with EtOAc (3 × 5 mL). The combined organic phases were treated with aqueous HCl (0.1 M), washed with bine, dried over $MgSO_4$, filtered and concentrated in *vacuo*. The crude products were purified by flash column chromatography on silica gel (Hexanes/EtOAc/AcOH = 98:2:0 to 95:4:1) to give *Z*/*E* isomers of **46** as a colourless oil.

Table S4. Wittig Olefination on α-Monofluorinated Model Lactone.[a,b]

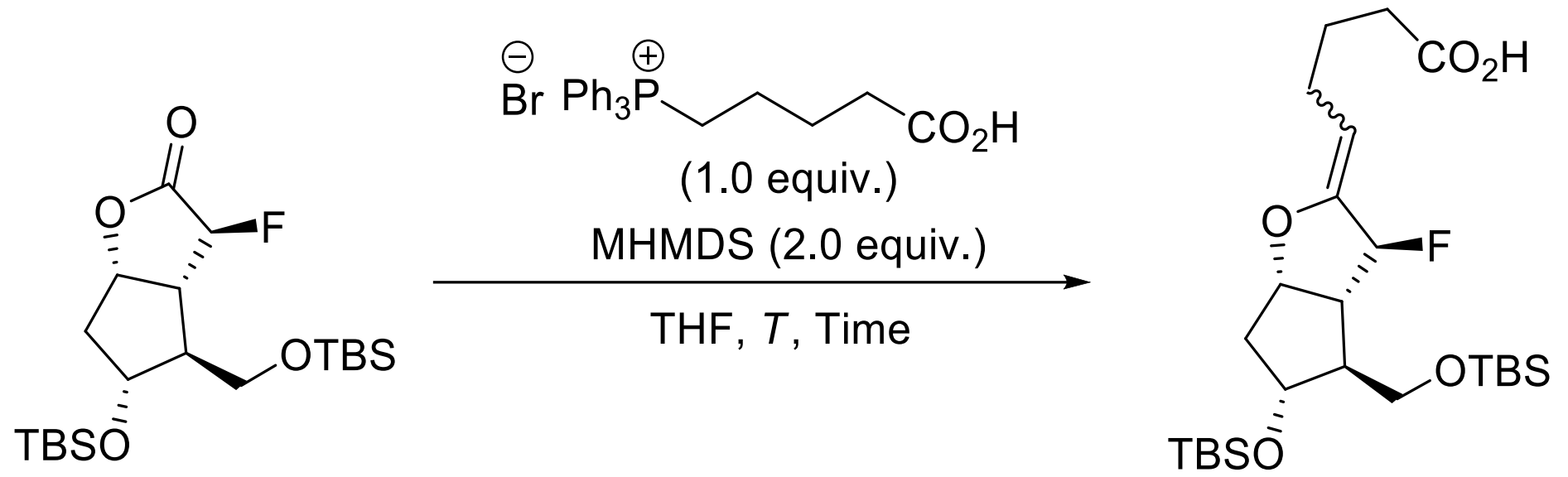

45 **46**

Entry	M (2.0 equiv.)	*T* [°C]	Time	Yield [%]	Z/E
1	Na	r.t.	2 h	< 5	-
2	K	r.t.	2 h	< 5	-
3	Li	0 °C	2.5 h	16	81:19
4	Li	–20 °C	3 h	10	89:11
5	Li	–78 °C	3 h	< 5	-
6	Li	r.t.	2 h	45	76:24

[a] The reactions were carried out on a 0.1 mmol scale. [b] Yield and *Z*/*E* ratio determined by ^{19}F NMR using α,α,α-trifluorotoluene as an internal standard.

5-((3*S*,3a*R*,4S,5*R*,6a*S*)-5-(*tert*-Butyldimethylsilyloxy)-4-((*tert*-butyldimethylsilyloxy)methyl)-3-fluorohexahydro-2*H*-cyclopenta[*b*]furan-2-ylidene)pentanoic acid (46)

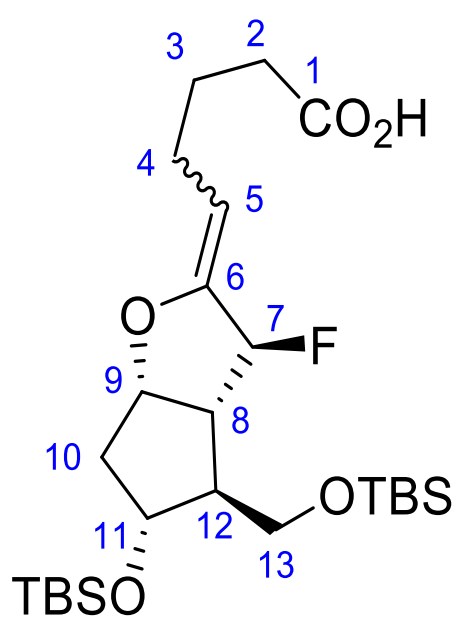

46

R_f = 0.14 (Hexanes/EtOAc = 90:10)

IR (thin film) ν_{max}/cm^{-1}: 2953, 2929, 2894, 2857, 1709, 1471, 1252, 1095, 1004, 880, 832, 774.

^{1}H NMR (400 MHz, CDCl$_3$) (*Z*)-**46**: δ_H = 5.09 (d, *J* = 56.0 Hz, 1H, ***H***C$_7$), 4.82 (td, *J* = 6.5, 2.8 Hz, 1H, ***H***C$_5$), 4.66 (q, *J* = 7.3 Hz, 1H, ***H***C$_9$), 3.98 (q, *J* = 7.6 Hz, 1H, ***H***C$_{11}$), 3.69 – 3.61 (m, 2H, ***H$_2$***C$_{13}$), 2.64 – 2.54 (m, 1H, ***H***C$_8$), 2.39 – 2.32 (m, 3H, ***H$_2$***C$_2$+***H$_A$***C$_{10}$), 2.20 – 2.13 (m, 2H,

H$_2$C$_4$), 1.77 – 1.70 (m, 3H, ***H***$_2$C$_3$+***H***$_B$C$_{10}$), 1.67 – 1.60 (m, 1H, ***H***C$_{12}$), 0.90 (s, 9H, SiC(C***H***$_3$)$_3$), 0.86 (s, 9H, SiC(C***H***$_3$)$_3$), 0.06 (s, 6H, SiC***H***$_3$+SiC***H***$_3$), 0.03 (s, 6H, SiC***H***$_3$+SiC***H***$_3$); (*E*)-**46**: δ_H = 5.43 (d, *J* = 55.3 Hz, 1H, ***H***C$_7$), 4.96 (q, *J* = 7.3 Hz, 1H, ***H***C$_9$), 4.76 (td, *J* = 6.5, 2.7 Hz, ***H***C$_5$), 3.98 (q, *J* = 7.6 Hz, 1H, ***H***C$_{11}$), 3.69 – 3.61 (m, 2H, ***H***$_2$C$_{13}$), 2.64 – 2.54 (m, 1H, ***H***C$_8$), 2.39 – 2.32 (m, 3H, ***H***$_2$C$_2$+***H***$_A$C$_{10}$), 2.20 – 2.13 (m, 2H, ***H***$_2$C$_4$), 1.77 – 1.70 (m, 3H, ***H***$_2$C$_3$+***H***$_B$C$_{10}$), 1.67 – 1.60 (m, 1H, ***H***C$_{12}$), 0.91 (s, 9H, SiC(C***H***$_3$)$_3$), 0.86 (s, 9H, SiC(C***H***$_3$)$_3$), 0.07 (s, 6H, SiC***H***$_3$+SiC***H***$_3$), 0.03 (s, 6H, SiC***H***$_3$+SiC***H***$_3$).

^{19}F NMR (377 MHz, CDCl$_3$) (*Z*)-**46**: δ_F = – 163.12 (ddd, $^2J_{HF}$ = 56.0 Hz, $^3J_{HF}$ = 22.0 Hz, $^4J_{HF}$ = 11.5 Hz); (*E*)-**46**: δ_F = –157.69 (dddd, $^2J_{HF}$ = 56.0 Hz, $^3J_{HF}$ = 26.5 Hz, $^4J_{HF}$ = 13.0 Hz, $^4J_{HF}$ = 6.0 Hz).

^{13}C NMR (100 MHz, CDCl$_3$) (*Z*)-**46**: δ_C = 179.4 (**C$_1$**), 154.1 (d, $^2J_{CF}$ = 14.6 Hz, **C$_6$**), 103.4 (d, $^3J_{CF}$ = 10.6 Hz, **C$_5$**), 95.8 (d, $^1J_{CF}$ = 175.4 Hz, **C$_7$**), 83.8 (**C$_9$**), 73.6 (d, $^4J_{CF}$ = 2.8 Hz, **C$_{11}$**), 61.7 (**C$_{13}$**), 50.7 (d, $^3J_{CF}$ = 6.4 Hz, **C$_{12}$**), 49.2 (d, $^2J_{CF}$ = 22.0 Hz, **C$_8$**), 41.6 (**C$_{10}$**), 33.6 (**C$_2$**), 26.0 (SiC(***C***H$_3$)$_3$), 25.9 (SiC(***C***H$_3$)$_3$), 24.9 (d, $^4J_{CF}$ = 3.8 Hz, **C$_4$**), 24.6 (d, $^5J_{CF}$ = 3.8 Hz, **C$_3$**), 18.4 (Si***C***(CH$_3$)$_3$), 18.1 (Si***C***(CH$_3$)$_3$), -4.4 (Si***C***H$_3$), -4.8 (Si***C***H$_3$), -5.3 (Si***C***H$_3$), -5.4 (Si***C***H$_3$); (*E*)-**46**: 179.3 (**C$_1$**), 154.8 (d, $^2J_{CF}$ = 14.4 Hz, **C$_6$**), 103.4 (d, $^3J_{CF}$ = 9.9 Hz, **C$_5$**), 92.1 (d, $^1J_{CF}$ = 165.5 Hz, **C$_7$**), 82.8 (**C$_9$**), 73.6 (d, $^4J_{CF}$ = 2.8 Hz, **C$_{11}$**), 61.8 (**C$_{13}$**), 50.6 (d, $^3J_{CF}$ = 6.5 Hz, **C$_{12}$**), 49.2 (d, $^2J_{CF}$ = 22.4 Hz, **C$_8$**), 41.4 (**C$_{10}$**), 33.2 (**C$_2$**), 26.6 (d, $^4J_{CF}$ = 3.6 Hz, **C$_4$**), 26.0 (SiC(***C***H$_3$)$_3$), 25.9 (SiC(***C***H$_3$)$_3$), 25.5 (d, $^5J_{CF}$ = 3.2 Hz, **C$_3$**), 18.4 (Si***C***(CH$_3$)$_3$), 18.1 (Si***C***(CH$_3$)$_3$), -4.4 (Si***C***H$_3$), -4.8 (Si***C***H$_3$), -5.3 (Si***C***H$_3$), -5.4 (Si***C***H$_3$).

HRMS (ESI$^+$) calculated for $C_{25}H_{47}FNaO_5Si_2$ [M+Na$^+$] = 525.2838, found 525.2808.

Synthesis of alkene targets 2, 3, 6 and 7

Synthesis of precursors 24 and 28 for alkene targets

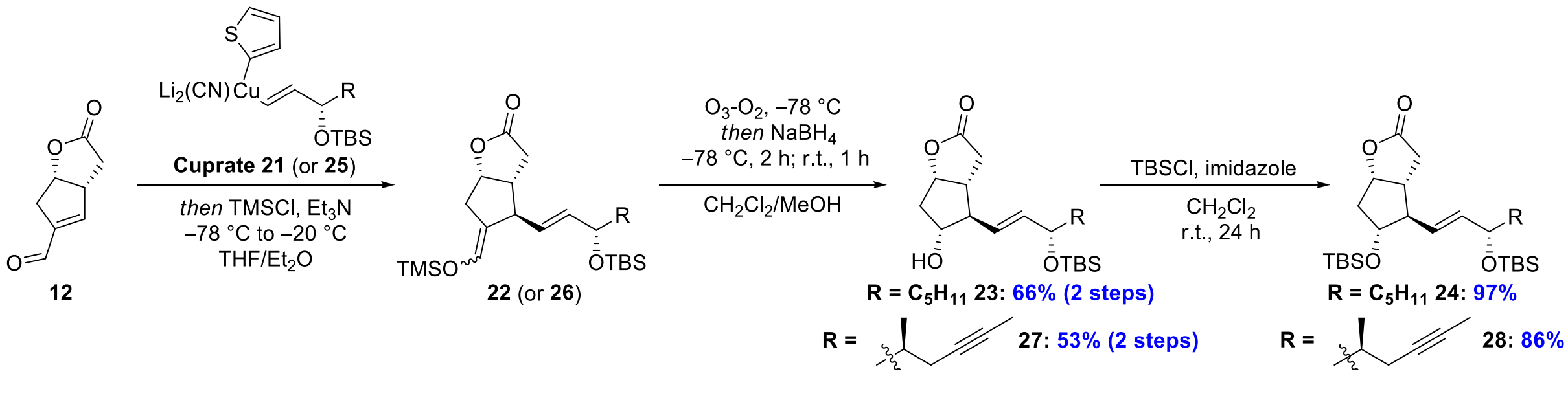

(3a*R*,4*R*,5*R*,6a*S*)-4-((*S*,*E*)-3-((*tert*-Butyldimethylsilyl)oxy)oct-1-enyl)-5-hydroxyhexahydro-2*H*-cyclopenta[*b*]furan-2-one (23)

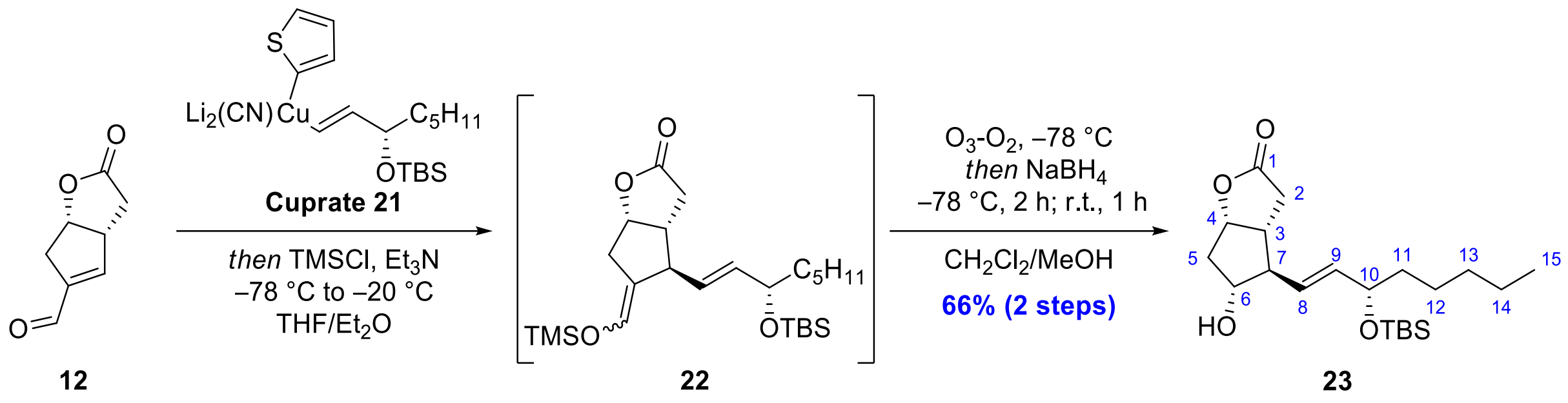

To a solution of **S6** (1.93 g, 6.00 mmol, 1.2 eq.) in Et_2O (28.8 mL) stirring at –78 °C, *t*-BuLi (1.70 M in pentane, 7.10 mL, 12.0 mmol, 2.4 eq.) was added dropwise and the resulting pale yellow solution was stirred at –78 °C for 2 h prior to warming to –40 °C to stir for a further 2 h before cooling back to –78 °C.

A separate solution of thiophene (480 µL, 506 mg, 6.00 mmol, 1.2 eq.) in THF (28.8 mL) was prepared and cooled to –40 °C and *n*-BuLi (1.60 M in hexanes, 3.80 mL, 6.00 mmol, 1.2 eq.) was added dropwise. The resulting colourless/very pale yellow, clear solution was stirred at –40 °C for 45 min before cooling to –78 °C whereupon CuCN (538 mg, 6.00 mmol, 1.2 eq.) was added in a single portion and the solution was warmed to r.t. The formed thienyl cuprate solution was then added to the above vinyl lithium solution at –78 °C and THF (28.8 mL) was added (used for washing the flask). The bright yellow reaction mixture was warmed to –20 °C to facilitate the formation of cuprate **21**. The reaction mixture was stirred at –20 °C for 1 h before cooling back to –78 °C.

A solution of **12** (761 mg, 5.00 mmol, 1.0 eq.) in THF (28.8 mL) was prepared and added to the solution of cuprate **21** at –78 °C to give a bright orange reaction mixture. The reaction mixture was stirred at –78 °C for 1 h before the sequential addition of TMSCl (3.20 mL, 2.72 g, 25.0 mmol, 5.0 eq.) and NEt_3 (4.20 mL, 3.04 g, 30.0 mmol, 6.0 eq.). The reaction mixture was warmed to –20 °C to stir for 20 min before quenching by addition of saturated aqueous NH_4Cl solution (60 mL) and warming to r.t.. The aqueous phase was extracted with EtOAc (3 × 60 mL), the organic phases were then combined, washed further with saturated aqueous NH_4Cl solution (60 mL), washed with brine, dried over $MgSO_4$, filtered and concentrated *in vacuo* to give crude **22** as a brown oil, which was used directly in the next step.

In a round-bottom flask, a solution of crude **22** in CH_2Cl_2/MeOH (40 mL, 3:1 *v/v*) was cooled to –78 °C and a stream of O_3-O_2(g) was bubbled through the reaction solution. The reaction progress was carefully monitored by TLC (CH_2Cl_2) until a small amount of **22** remained (~3.0 min). The reaction mixture was then purged of O_3(g) by bubbling N_2(g) through the solution for 20 min followed by the portionwise addition of freshly ground $NaBH_4$ (568 mg, 15.0 mmol,

3.0 eq.). The reaction was stirred at –78 °C for 2 h before the dry ice/acetone bath was removed and the reaction was allowed to warm to r.t., at which point it was stirred for a further 1 h. The reaction mixture was then poured over saturated brine solution (50 mL) and extracted with EtOAc (3 × 50 mL). The organic phases were combined, dried over $MgSO_4$, filtered and concentrated *in vacuo*. The crude product was purified by flash column chromatography on silica gel (CH_2Cl_2/EtOAc = 95:5 to 90:10) to give pure **23** as a colourless oil (1.27 g, 3.32 mmol, 66%).

$\boldsymbol{R_f}$ = 0.20 (CH_2Cl_2/EtOAc = 90:10)

IR (thin film) $\boldsymbol{\nu_{max}}$/cm^{-1}: 3470, 2955, 2929, 2856, 1761, 1463, 1364, 1250, 1171, 1078, 969, 834, 774.

^{1}**H NMR** (500 MHz, $CDCl_3$) δ_H = 5.59 (ddd, J = 15.4, 5.8, 0.8 Hz, 1H, $\boldsymbol{H}C_9$), 5.41 (ddd, J = 15.4, 8.1, 1.0 Hz, 1H, $\boldsymbol{H}C_8$), 4.92 (td, J = 7.0, 2.8 Hz, 1H, $\boldsymbol{H}C_4$), 4.09 – 4.05 (m, 1H, $\boldsymbol{H}C_{10}$), 4.00 (q, J = 6.7 Hz, 1H, $\boldsymbol{H}C_6$), 2.74 (dd, J = 18.1, 9.8 Hz, 1H, $\boldsymbol{H_A}C_2$), 2.63 – 2.57 (m, 1H, $\boldsymbol{H}C_3$), 2.49 – 2.46 (m, 1H, $\boldsymbol{H_B}C_2$), 2.45 – 2.44 (m, 1H, $\boldsymbol{H_A}C_5$), 2.36 – 2.32 (m, 1H, $\boldsymbol{H}C_7$), 1.98 (ddd, J = 14.9, 6.9, 2.8 Hz, 1H, $\boldsymbol{H_B}C_5$), 1.76 (br. s, 1H, O$\boldsymbol{H}$), 1.49 – 1.40 (m, 2H, $\boldsymbol{H_2}C_{11}$), 1.34 – 1.24 (m, 6H, $\boldsymbol{H_2}C_{12}$+$\boldsymbol{H_2}C_{13}$+$\boldsymbol{H_2}C_{14}$), 0.89 – 0.86 (m, 12H, SiC(C$\boldsymbol{H_3}$)$_3$+$\boldsymbol{H_3}C_{15}$), 0.04 (s, 3H, SiC$\boldsymbol{H_3}$), 0.01 (s, 3H, SiC$\boldsymbol{H_3}$).

^{13}**C NMR** (125 MHz, $CDCl_3$) δ_C = 175.9 (**C$_1$**), 136.5 (**C$_9$**), 126.7 (**C$_8$**), 81.8 (**C$_4$**), 76.1 (**C$_6$**), 72.0 (**C$_{10}$**), 55.3 (**C$_7$**), 41.8 (**C$_3$**), 39.1 (**C$_5$**), 37.4 (**C$_{11}$**), 33.5 (**C$_2$**), 30.9 (**C$_{12}$**), 25.0 (SiC(***C***H$_3$)$_3$), 24.2 (**C$_{13}$**), 21.8 (**C$_{14}$**), 17.4 (Si***C***(CH$_3$)$_3$), 13.2 (**C$_{15}$**), -5.3 (Si***C***H$_3$), -5.6 (Si***C***H$_3$).

HRMS (ESI$^+$) calculated for $C_{21}H_{38}NaO_4Si$ [M+Na$^+$] 405.2432, found 405.2433.

$[\alpha]_D^{18}$ = –107.3 (c 1.00, $CHCl_3$)

(3a*R*,4*R*,5*R*,6a*S*)-4-((*S*,*E*)-3-(*tert*-Butyldimethylsilyloxy)oct-1-enyl)-5-hydroxyhexahydro-2*H*-cyclopenta[*b*]furan-2-one (24)

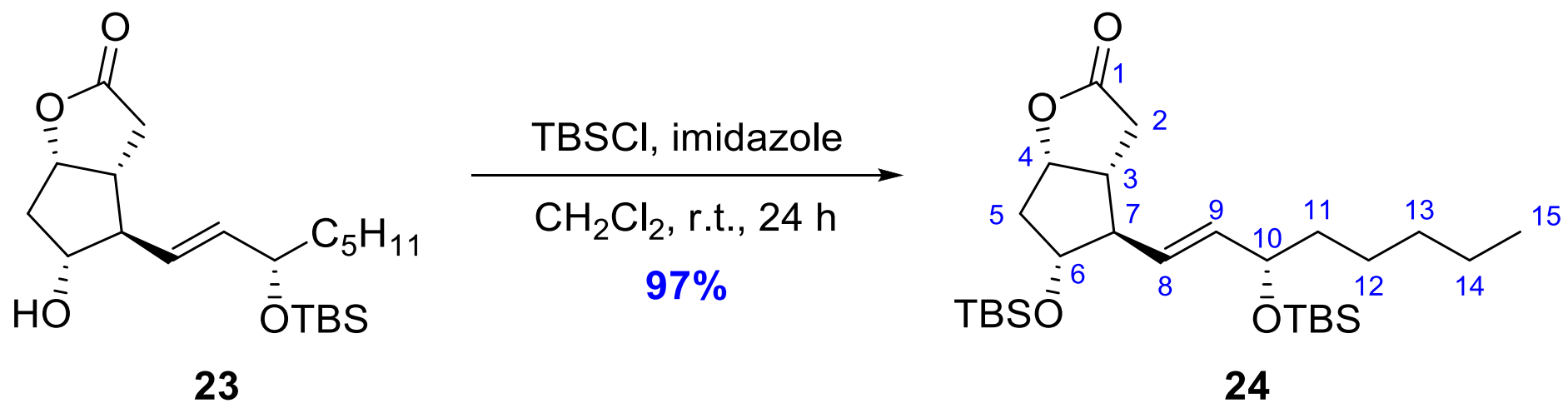

To a solution of **23** (2.40 g, 6.27 mmol, 1.0 eq.) in CH_2Cl_2 (20.9 ml) was added imidazole (982 mg, 14.4 mmol, 2.3 eq.) and TBSCl (1.42 g, 9.41 mmol, 1.5 eq.) at 0 °C. The reaction mixture was stirred at r.t. for 24 h before the mixture was poured over H_2O (30 mL) and the aqueous phase was extracted with CH_2Cl_2 (3 × 30 mL). The organic phases were combined, washed

with brine, dried over $MgSO_4$, filtered and concentrated *in vacuo*. The crude product was purified by flash column chromatography on silica gel (Hexanes/EtOAc = 95:5 to 90:10) to give pure **24** as a white, amorphous solid (3.02 g, 6.08 mmol, 97%).

Mp 68–74 °C

***R*f** = 0.20 (Hexanes/EtOAc = 90:10)

IR (thin film) ν_{max}/cm^{-1}: 2954, 2929, 2895, 2857, 1752, 1463, 1249, 1463, 1249, 1116, 973, 834, 773.

^{1}H NMR (500 MHz, $CDCl_3$) δ_H = 5.49 (dd, *J* = 15.4, 5.7 Hz, 1H, ***H***C9), 5.36 (dd, *J* = 15.4, 7.6 Hz, 1H, ***H***C8), 4.94 (td, *J* = 7.0, 2.0 Hz, 1H, ***H***C4), 4.03 (q, *J* = 5.9 Hz, 1H, ***H***C10), 3.97 (q, *J* = 5.2 Hz, 1H, ***H***C6), 2.74 (dd, *J* = 18.0, 10.4 Hz, 1H, ***H**A*C2), 2.63 (dtd, *J* = 10.2 Hz, 6.8 Hz, 2.6 Hz, 1H, ***H***C3), 2.48 (dd, *J* = 18.0, 2.4 Hz, 1H, ***H**B*C2), 2.43 (q, *J* = 6.2 Hz, 1H, ***H***C7), 2.26 – 2.21 (m, 1H, ***H**A*C5), 1.97 (dd, *J* = 14.5, 3.2 Hz, 1H, ***H**B*C5), 1.48 – 1.36 (m, 2H, ***H*2**C11), 1.29 – 1.25 (m, 6H, ***H*2**C12+***H*2**C13+***H*2**C14), 0.88 – 0.86 (m, 21H, SiC(C***H*3**)3+SiC(C***H*3**)3+***H*3**C15), 0.04 (s, 3H, SiC***H*3**), 0.04 (s, 3H, SiC***H*3**), 0.03 (s, 3H, SiC***H*3**), -0.01 (s, 3H, SiC***H*3**).

^{13}C NMR (125 MHz, $CDCl_3$) δ_C = 177.2 (**C1**), 136.0 (**C9**), 128.5 (**C8**), 83.5 (**C4**), 78.2 (**C6**), 73.0 (**C10**), 56.8 (**C7**), 42.4 (**C3**), 40.8 (**C5**), 38.5 (**C11**), 35.2 (**C2**), 31.9 (**C12**), 26.0 (SiC(***C***H3)3), 25.9 (SiC(***C***H3)3), 25.2 (**C13**), 22.7 (**C14**), 18.4 (Si***C***(CH3)3), 18.1 (Si***C***(CH3)3), 14.2 (**C15**), -4.1 (Si***C***H3), -4.6 (Si***C***H3), -4.6 (Si***C***H3), -4.8 (Si***C***H3).

HRMS (ESI^{+}) calculated for $C_{27}H_{52}NaO_4Si_2$ [M+Na^{+}] 519.3296, found 519.3292.

$[\alpha]_D^{20}$ = –23.9 (*c* 1.00, $CHCl_3$)

(3a*R*,4*R*,5*R*,6a*S*)-4-((3*S*,4*S*,*E*)-3-(*tert*-Butyldimethylsilyloxy)-4-methyloct-1-en-6-ynyl)-5-hydroxyhexahydro-2*H*-cyclopenta[*b*]furan-2-one (27)

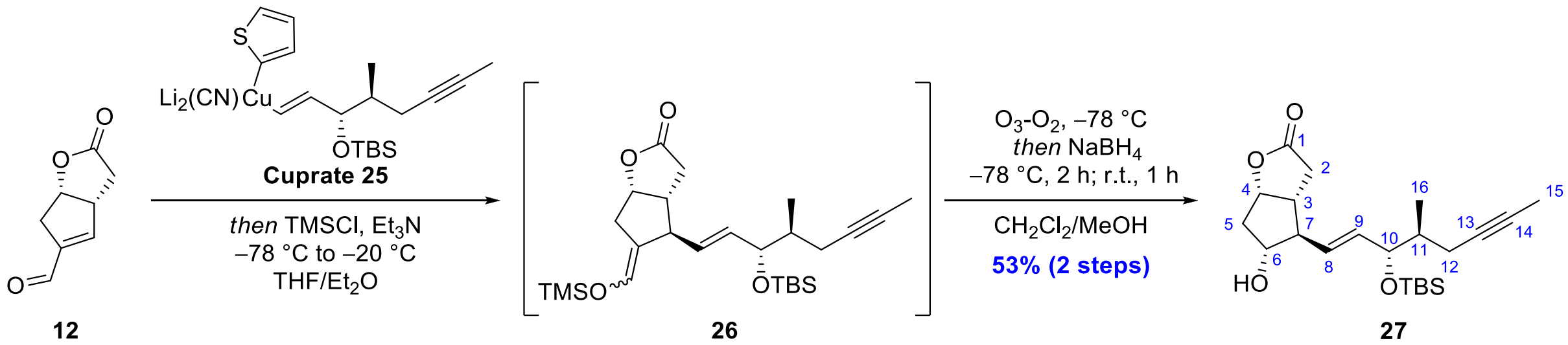

To a solution of **18** (953 g, 2.52 mmol, 1.2 eq.) in Et_2O (10.1 mL) stirring at –78 °C, *t*-BuLi (1.70 M in pentane, 3.00 mL, 5.04 mmol, 2.4 eq.) was added dropwise and the resulting pale yellow solution was stirred at –78 °C for 2 h prior to warming to –40 °C to stir for a further 2 h before cooling back to –78 °C.

A separate solution of thiophene (202 µL, 212 mg, 2.52 mmol, 1.2 eq.) in THF (10.1 mL) was prepared and cooled to –40 °C and *n*-BuLi (1.60 M in hexanes, 1.60 mL, 2.52 mmol, 1.2 eq.) was added dropwise. The resulting dark yellow, clear solution was stirred at –40 °C for 45 min before cooling to –78 °C whereupon CuCN (227 mg, 2.52 mmol, 1.2 eq.) was added in a single portion and the solution was then warmed to r.t. The formed thienyl cuprate solution was then added to the above vinyl lithium solution at –78 °C and THF (10.1 mL) was added (used for washing the flask). The bright yellow reaction mixture was warmed to –20 °C to facilitate the formation of cuprate **25**. The reaction mixture was stirred at –20 °C for 1 h before cooling back to –78 °C.

A solution of **12** (319 mg, 2.10 mmol, 1.0 eq.) in THF (10.1 mL) was prepared and added to the solution of cuprate **25** at –78 °C to give a bright orange reaction mixture. The reaction mixture was stirred at –78 °C for 1 h before the sequential addition of TMSCl (1.30 mL, 1.14 g, 10.5 mmol, 5.0 eq.) and NEt_3 (1.80 mL, 1.27 g, 12.6 mmol, 6.0 eq.). The reaction mixture was warmed to –20 °C to stir for 20 min before quenching by addition of saturated aqueous NH_4Cl solution (20 mL) and warming to r.t.. The aqueous phase was extracted with EtOAc (3 × 20 mL), the organic phases were then combined, washed further with saturated aqueous NH_4Cl solution (20 mL), washed with brine, dried over $MgSO_4$, filtered and concentrated *in vacuo* to give crude **26** as a brown oil, which was used directly in the next step.

In a round-bottom flask, a solution of crude **26** in CH_2Cl_2/MeOH (16.8 mL, 3:1 *v/v*) was cooled to –78 °C and a stream of O_3-O_2(g) was bubbled through the reaction solution. The reaction progress was carefully monitored by TLC (CH_2Cl_2) until a small amount of **26** remained (~2.0 min). The reaction mixture was then purged of O_3(g) by bubbling N_2(g) through the solution for 20 min followed by the portion-wise addition of freshly ground $NaBH_4$ (238 mg, 6.30 mmol, 3.0 eq.). The reaction was stirred at –78 °C for 2 h before the dry ice/acetone bath was removed and the reaction was allowed to warm to r.t., at which point it was stirred for a further 1 h. The reaction mixture was then poured over saturated brine solution (15 mL) and extracted with EtOAc (3 × 15 mL). The organic phases were combined, dried over $MgSO_4$, filtered and concentrated *in vacuo*. The crude product was purified by flash column chromatography on silica gel (CH_2Cl_2/EtOAc = 95:5 to 90:10) to give pure **27** as a colourless oil (436 mg, 1.11 mmol, 53%).

$\boldsymbol{R_f}$ = 0.28 (CH_2Cl_2/EtOAc = 90:10)

IR (thin film) $\boldsymbol{\nu_{max}}$/cm^{-1}: 3451, 2955, 2928, 2894, 2856, 1768, 1471, 1360, 1250, 1173, 1079, 1031, 973, 835, 775.

^{1}**H NMR** (400 MHz, $CDCl_3$) δ_H = 5.55 (dd, *J* = 15.6, 6.3 Hz, 1H, ***H***C_9), 5.42 (dd, *J* = 15.6, 7.9 Hz, 1H, ***H***C_8), 4.92 (td, *J* = 7.0, 2.7 Hz, 1H, ***H***C_4), 4.04 – 3.97 (m, 2H, ***H***C_6+***H***C_{10}), 2.75 (dd, *J* = 18.0, 9.8 Hz, 1H, $\boldsymbol{H_A}C_2$), 2.60 (ddd, *J* = 9.7, 8.4, 1.9 Hz, 1H, ***H***C_3), 2.50 – 2.43 (m, 2H, $\boldsymbol{H_B}C_2$+***H***C_7), 2.39 – 2.34 (m, 1H, $\boldsymbol{H_A}C_5$), 2.20 – 2.13 (m, 1H, $\boldsymbol{H_A}C_{12}$), 2.12 – 2.04 (m, 1H, $\boldsymbol{H_B}C_{12}$), 1.99 (ddd, *J* = 14.8, 6.8, 2.7 Hz, 1H, $\boldsymbol{H_B}C_5$), 1.78 – 1.77 (m, 4H, $\boldsymbol{H_3}C_{15}$+O***H***), 1.72 – 1.63 (m, 1H, ***H***C_{11}), 0.92 – 0.87 (m, 12H, SiC(C$\boldsymbol{H_3}$)$_3$+$\boldsymbol{H_3}C_{16}$), 0.05 (s, 3H, SiC$\boldsymbol{H_3}$), -0.01 (s, 3H, SiC$\boldsymbol{H_3}$).

^{13}**C NMR** (100 MHz, $CDCl_3$) δ_C = 176.9 (**C$_1$**), 135.3 (**C$_9$**), 129.6 (**C$_8$**), 82.8 (**C$_4$**), 77.9 (**C$_{14}$**), 77.5 (**C$_6$**), 76.8 (**C$_{13}$**), 75.8 (**C$_{10}$**), 56.4 (**C$_7$**), 42.8 (**C$_3$**), 40.2 (**C$_5$**), 39.5 (**C$_{11}$**), 34.5 (**C$_2$**), 26.0 (SiC(***C***H_3)$_3$), 21.9 (**C$_{12}$**), 18.3 (Si***C***(CH_3)$_3$), 15.7 (**C$_{16}$**), 3.6 (**C$_{15}$**), -3.9 (Si***C***H_3), -4.7 (Si***C***H_3).

HRMS (ESI^+) calculated for $C_{22}H_{36}NaO_4Si$ [$M+Na^+$] 415.2275, found 415.2281.

[*α*]$_D^{25}$= +6.2 (*c* 0.27, $CHCl_3$)

(3a*R*,4*R*,5*R*,6a*S*)-5-(*tert*-Butyldimethylsilyloxy)-4-((3*S*,4*S*,*E*)-3-(*tert*-butyldimethylsilyloxy)-4-methyloct-1-en-6-ynyl)hexahydro-2*H*-cyclopenta[*b*]furan-2-one (28)

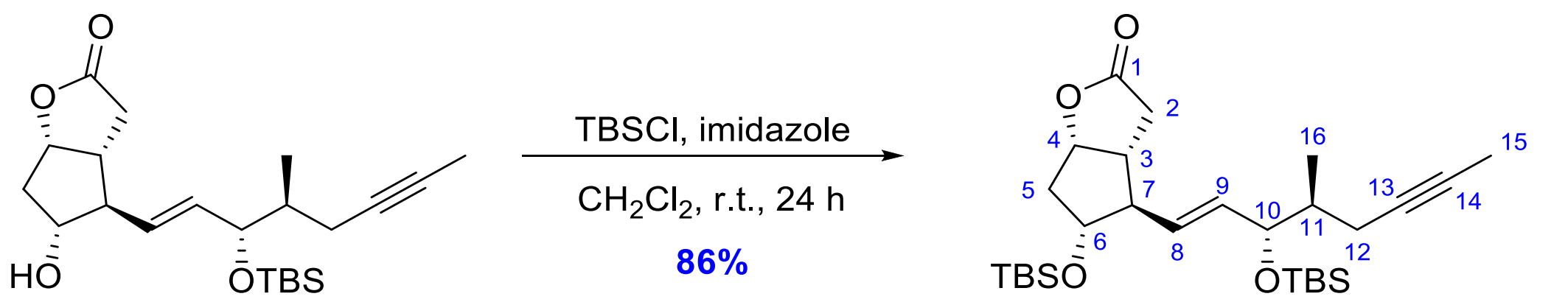

27 **28**

To a solution of **27** (500 mg, 1.28 mmol, 1.0 eq.) in CH_2Cl_2 (4.3 ml) was added imidazole (200 mg, 2.93 mmol, 2.3 eq.) and TBSCl (288 mg, 1.91 mmol, 1.5 eq.) at 0 °C. The reaction mixture was stirred at r.t. for 24 h before the mixture was poured over H_2O (6 mL) and the aqueous phase was extracted with CH_2Cl_2 (3 × 6 mL). The organic phases were combined, washed with brine, dried over $MgSO_4$, filtered and concentrated *in vacuo*. The crude product was purified by flash column chromatography on silica gel (Hexanes/EtOAc = 95:5 to 90:10) to give pure **28** as a white, amorphous solid (557 mg, 1.10 mmol, 86%).

Mp 62–66 °C

***R*$_f$** = 0.22 (Hexanes /EtOAc = 90:10)

IR (thin film) ν_{max}/cm^{-1}: 2953, 2929, 2894, 2856, 1750, 1471, 1361, 1249, 1164, 1112, 1030, 969, 913, 834, 774.

^{1}H NMR (400 MHz, $CDCl_3$) $\delta_H = 5.45$ (dd, $J = 15.5, 5.7$ Hz, 1H, $\boldsymbol{H}C_9$), 5.38 (dd, $J = 15.6, 6.7$ Hz, 1H, $\boldsymbol{H}C_8$), 4.96 (td, $J = 7.0, 1.9$ Hz, 1H, $\boldsymbol{H}C_4$), 4.02 – 3.94 (m, 2H, $\boldsymbol{H}C_6+\boldsymbol{H}C_{10}$), 2.76 (dd, $J = 17.7, 10.5$ Hz, 1H, $\boldsymbol{H_A}C_2$), 2.69 – 2.62 (m, 1H, $\boldsymbol{H}C_3$), 2.53 – 2.46 (m, 2H, $\boldsymbol{H_B}C_2+\boldsymbol{H}C_7$), 2.25 – 2.18 (m, 1H, $\boldsymbol{H_A}C_5$), 2.17 – 2.12 (m, 1H, $\boldsymbol{H_A}C_{12}$), 2.11 – 2.03 (m, 1H, $\boldsymbol{H_B}C_{12}$), 1.99 (dd, $J = 14.7, 2.8$ Hz, 1H, $\boldsymbol{H_B}C_5$), 1.78 (t, $J = 2.5$ Hz, 3H, $\boldsymbol{H_3}C_{15}$), 1.70 – 1.61 (m, 1H, $\boldsymbol{H}C_{11}$), 0.89 – 0.87 (m, 21H, $SiC(C\boldsymbol{H_3})_3$+$SiC(C\boldsymbol{H_3})_3$+$\boldsymbol{H_3}C_{16}$), 0.05 (s, 6H, $SiC\boldsymbol{H_3}$+ $SiC\boldsymbol{H_3}$), 0.04 (s, 3H, $SiC\boldsymbol{H_3}$), -0.02 (s, 3H, $SiC\boldsymbol{H_3}$).

^{13}C NMR (100 MHz, $CDCl_3$) δ_C = 177.2 (**C1**), 133.5 (**C9**), 130.5 (**C8**), 83.7 (**C4**), 78.3 (**C14**), 77.8 (**C6**), 76.7 (**C13**), 76.0 (**C10**), 57.1(**C7**), 42.5 (**C3**), 40.8 (**C5**), 39.7 (**C11**), 35.3 (**C2**), 26.0 ($SiC(\boldsymbol{C}H_3)_3$), 25.9 ($SiC(\boldsymbol{C}H_3)_3$), 22.1 (**C12**), 18.3 ($Si\boldsymbol{C}(CH_3)_3$), 18.1 ($Si\boldsymbol{C}(CH_3)_3$), 15.6 (**C16**), 3.6 (**C15**), -3.9 ($Si\boldsymbol{C}H_3$), -4.6 ($Si\boldsymbol{C}H_3$), -4.7 ($Si\boldsymbol{C}H_3$), -4.8 ($Si\boldsymbol{C}H_3$).

HRMS (ESI^+) calculated for $C_{28}H_{50}NaO_4Si_2$ $[M+Na^+]$ 529.3140, found 529.3132.

$[\alpha]_D^{20}$ = –16.0 (*c* 1.00, $CHCl_3$)

Synthesis of difluorinated targets 6 and 7.

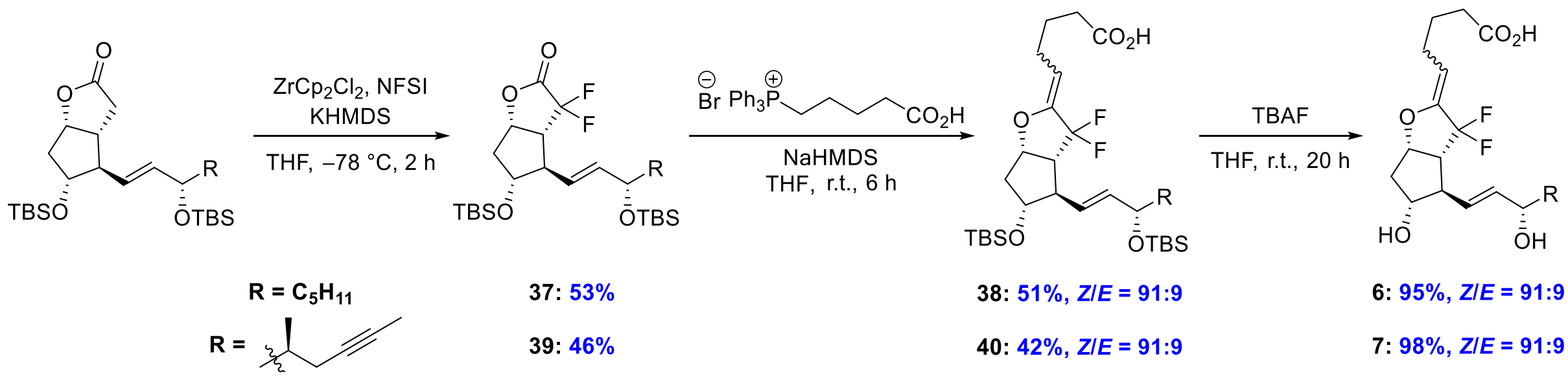

(3a*R*,4*R*,5*R*,6a*S*)-5-(*tert*-Butyldimethylsilyloxy)-4-((*S*,*E*)-3-(*tert*-butyldimethylsilyloxy)oct-1-enyl)-3,3-difluorohexahydro-2*H*-cyclopenta[*b*]furan-2-one (37)

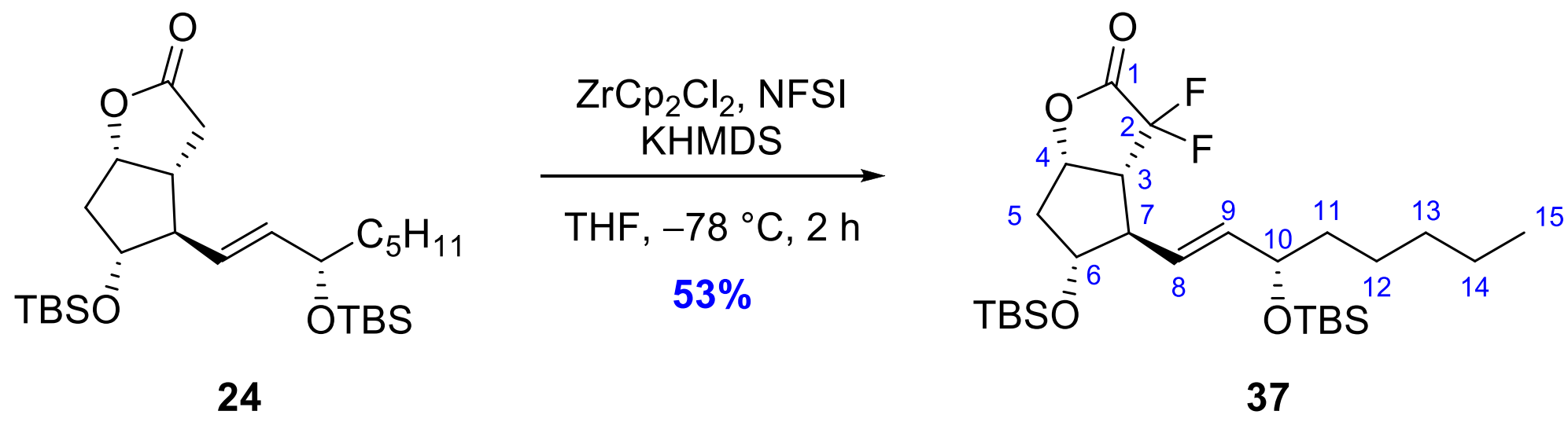

A solution of NFSI (2.14 g, 6.80 mmol, 4.0 eq.) and $ZrCp_2Cl_2$ (1.99 g, 6.80 mmol, 4.0 eq.) in THF (21.3 mL) was stirred at r.t. for 1 h and then cooled to –78 °C. A solution of **24** (845 mg, 1.70 mmol, 1.0 eq.) in THF (11.3 mL) was added followed by the drop-wise addition of KHMDS (1.0 M in THF, 8.50 mL, 8.50 mmol, 5.0 eq.) over 1 h, the reaction mixture was then

stirred at –78 °C for a further hour. The reaction mixture was warmed to r.t., quenched by addition of saturated $NaHCO_3$ solution (30 mL) and the aqueous phase was subsequently extracted with Et_2O (3 × 30 mL). The organic phases were combined, washed with brine, dried over $MgSO_4$, filtered and concentrated *in vacuo*. The crude product was purified by flash column chromatography on silica gel (Hexanes/Et_2O = 100:0 to 95:5) to give pure **37** as a pale yellow solid (482 mg, 0.90 mmol, 53%).

Mp 35–38 °C

***R*f** = 0.23 (Hexanes/Et_2O = 95:5)

IR (thin film) $\boldsymbol{\nu}_{\mathbf{max}}$/cm^{-1}: 2955, 2929, 2857, 1809, 1471, 1249, 1361, 1250, 1161, 1028, 968, 896, 835, 775.

^{1}H NMR (400 MHz, $CDCl_3$) δ_H = 5.55 (ddd, *J* = 15.4, 5.8, 0.9 Hz, 1H, ***H***C9), 5.37 (dd, *J* = 15.5, 7.7 Hz, 1H, ***H***C8), 5.16 – 5.13 (m, 1H, ***H***C4), 4.08 – 4.03 (m, 2H, ***H***C6+***H***C10), 3.01 (dd, *J* = 8.1, 3.1 Hz, 1H, ***H***C7), 2.95 (ddd, *J* = 25.6, 6.9, 3.4 Hz, 1H, ***H***C3), 2.14 – 2.05 (m, 2H, ***H$_A$***C5+***H$_B$***C5), 1.50 – 1.37 (m, 2H, ***H$_2$***C11), 1.32 – 1.21 (m, 6H, ***H$_2$***C12+***H$_2$***C13+***H$_2$***C14), 0.90 – 0.85 (m, 21H, SiC(C***H$_3$***)$_3$+SiC(C***H$_3$***)$_3$+***H$_3$***C15), 0.05 (s, 3H, SiC***H$_3$***), 0.04 (s, 3H, SiC***H$_3$***), 0.04 (s, 3H, SiC***H$_3$***), 0.00 (s, 3H, SiC***H$_3$***).

^{19}F NMR (377 MHz, $CDCl_3$) δ_F = -91.91 (dd, $^2J_{FF}$ = 280.0 Hz, $^3J_{HF}$ = 25.6 Hz), -113.81 (d, $^2J_{FF}$ = 280.0 Hz).

^{13}C NMR (100 MHz, $CDCl_3$) δ_C = 165.5 (dd, $^2J_{CF}$ = 35.3 Hz, $^2J_{CF}$ = 32.7 Hz, **C1**), 136.7 (**C9**), 127.1 (**C8**), 116.2 (dd, $^1J_{CF}$ = 265.0 Hz, $^1J_{CF}$ = 243.9 Hz, **C2**), 81.8 (dd, $^3J_{CF}$ = 3.6 Hz, $^3J_{CF}$ = 2.1 Hz, **C4**), 78.5 (**C6**), 72.9 (**C10**), 51.4 (dd, $^3J_{CF}$ = 5.3 Hz, $^3J_{CF}$ = 2.4 Hz, **C7**), 50.0 (dd, $^2J_{CF}$ = 23.3 Hz, $^2J_{CF}$ = 19.0 Hz, **C3**), 40.1 (d, $^4J_{CF}$ = 0.6 Hz, **C5**), 38.3 (**C11**), 31.9 (**C12**), 26.0 (SiC(***C***H$_3$)$_3$), 25.8 (SiC(***C***H$_3$)$_3$), 25.0 (**C13**), 22.7 (**C14**), 18.4 (Si***C***(CH$_3$)$_3$), 18.2 (Si***C***(CH$_3$)$_3$), 14.1 (**C15**), -4.2 (Si***C***H$_3$), -4.6 (Si***C***H$_3$), -4.9 (Si***C***H$_3$), -5.0 (Si***C***H$_3$).

HRMS (ESI$^+$) calculated for $C_{27}H_{50}F_2NaO_4Si_2$ [M+Na$^+$] 555.3108, found 555.3090.

[*α*]$_D^{21}$ = –25.0 (*c* 1.00, $CHCl_3$)

5-((3a*R*,4*R*,5*R*,6a*S*)-5-(*tert*-Butyldimethylsilyloxy)-4-((*S*,*E*)-3-(*tert*-butyldimethylsilyloxy)oct-1-enyl)-3,3-difluorohexahydro-2*H*-cyclopenta[*b*]furan-2-ylidene)pentanoic acid (38)

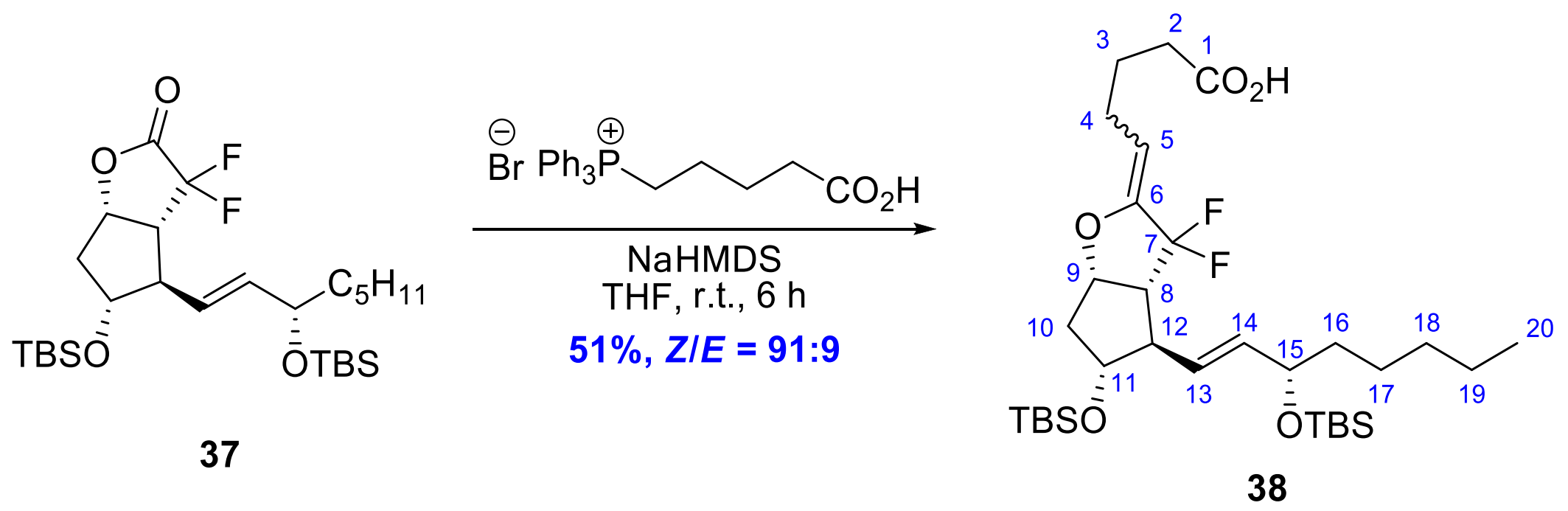

NaHMDS (1.0 M in THF, 1.50 mL, 1.50 mmol, 10.0 eq.) was added to a stirring suspension of (4-carboxybutyl)triphenylphosphonium bromide (332 mg, 0.75 mmol, 5.0 eq.) in THF (1.0 mL). The resulting orange suspension was heated to 70 °C for 1 h before cooling to r.t.. **37** (80.0 mg, 0.15 mmol, 1.0 eq.) was added as a solution in THF (0.15 mL) to the orange suspension and the reaction was stirred at r.t. for 6 h. The reaction mixture was quenched by addition of water (15 mL) and extracted with EtOAc (3 × 15 mL). The combined organic phases were washed with water (15 mL), treated with aqueous HCl (1.0 M, 15 mL), washed with brine, dried over $MgSO_4$, filtered and concentrated *in vacuo*. The crude product was purified by flash column chromatography on silica gel (Hexanes/EtOAc = 95:5 to 90:10; then Hexanes/EtOAc/AcOH = 90:9:1) to give **38** as a colourless oil (47.2 mg, 76.5 μmol, 51%, 91:9 *Z*/*E*).

$\boldsymbol{R_f}$ = 0.24 (Hexanes/EtOAc/AcOH = 80:19:1)

IR (thin film) ν_{max}/cm^{-1}: 2954, 2930, 2857, 1709, 1472, 1327, 1295, 1250, 1123, 1084, 1045, 967, 835, 775.

^{1}H NMR (400 MHz, $CDCl_3$) (*Z*)-**38**: δ_H = 5.60 (dd, *J* = 15.5, 5.3 Hz, 1H, HC_{14}), 5.48 (dd, *J* = 15.4, 6.1 Hz, 1H, HC_{13}), 4.81 (td, *J* = 7.4, 5.0 Hz, 1H, HC_5), 4.75 (td, *J* = 6.3, 3.4 Hz, 1H, HC_9), 4.07 (q, *J* = 5.8 Hz, 1H, HC_{11}), 3.88 (q, *J* = 7.5 Hz, 1H, HC_{15}), 2.63 – 2.54 (m, 2H, HC_8+HC_{12}), 2.44 – 2.36 (m, 3H, H_AC_{10}+H_2C_2), 2.17 – 2.12 (m, 2H, H_2C_4), 1.82 (ddd, *J* = 12.1, 8.2, 3.5 Hz, 1H, H_BC_{10}), 1.79 – 1.72 (m, 2H, H_2C_3), 1.50 – 1.37 (m, 2H, H_2C_{16}), 1.35 – 1.26 (m, 6H, H_2C_{17}+H_2C_{18}+H_2C_{19}), 0.96 – 0.75 (m, 21H, $SiC(CH_3)_3$+$SiC(CH_3)_3$+H_3C_{20}), 0.04 (s, 3H, $SiCH_3$), 0.03 (s, 3H, $SiCH_3$), 0.03 (s, 3H, $SiCH_3$), 0.02 (s, 3H, $SiCH_3$).

^{19}F NMR (377 MHz, $CDCl_3$) (*Z*)-**38**: δ_F = -82.74 (ddd, $^2J_{FF}$ = 251.4 Hz, $^3J_{HF}$ = 16.7 Hz, $^4J_{HF}$ = 4.6 Hz), -114.99 (d, $^2J_{FF}$ = 251.3 Hz); (*E*)-**38**: δ_F = -84.30 (dd, $^2J_{FF}$ = 259.5 Hz, $^3J_{HF}$ = 19.7 Hz), -108.19 (d, $^2J_{FF}$ = 259.9 Hz).

^{13}C NMR (100 MHz, $CDCl_3$) (*Z*)-**38**: δ_C = 178.9 (**C$_1$**), 149.6 (dd, $^2J_{CF}$ = 30.4 Hz, $^2J_{CF}$ = 25.5 Hz, **C$_6$**), 136.3 (**C$_{14}$**), 127.8 (**C$_{13}$**), 124.2 (dd, $^1J_{CF}$ = 255.1 Hz, $^1J_{CF}$ = 240.1 Hz, **C$_7$**), 99.9 (d,

$^3J_{CF}$ = 4.5 Hz, 1H, **C5**), 81.5 (d, $^3J_{CF}$ = 5.4 Hz, **C9**), 78.1 (d, $^4J_{CF}$ = 2.6 Hz, **C11**), 73.0 (**C15**), 51.7 (dd, $^2J_{CF}$ = 25.7 Hz, $^2J_{CF}$ =19.5 Hz, **C8**), 49.9 (d, $^3J_{CF}$ = 4.2 Hz, **C12**), 41.9 (**C10**), 38.6 (**C16**), 33.4 (**C2**), 31.8 (**C17**), 26.1 (SiC(*C*H3)3), 25.9 (SiC(*C*H3)3), 25.2 (**C18**), 24.3 (d, $^4J_{CF}$ = 3.0 Hz, **C4**), 24.1 (d, $^5J_{CF}$ = 1.9 Hz, **C3**), 22.8 (**C19**), 18.4 (Si*C*(CH3)3), 18.2 (Si*C*(CH3)3), 14.2 (**C20**), -4.2 (Si*C*H3), -4.4 (Si*C*H3), -4.5 (Si*C*H3), -4.7 (Si*C*H3).

HRMS (ESI$^+$) calculated for $C_{32}H_{58}F_2NaO_5Si_2$ [M+Na$^+$] 639.3683, found 639.3690.

5-((3a*R*,4*R*,5*R*,6a*S*)-3,3-Difluoro-5-hydroxy-4-((*S*,*E*)-3-hydroxyoct-1-enyl)hexahydro-2*H*-cyclopenta[*b*]furan-2-ylidene)pentanoic acid (6)

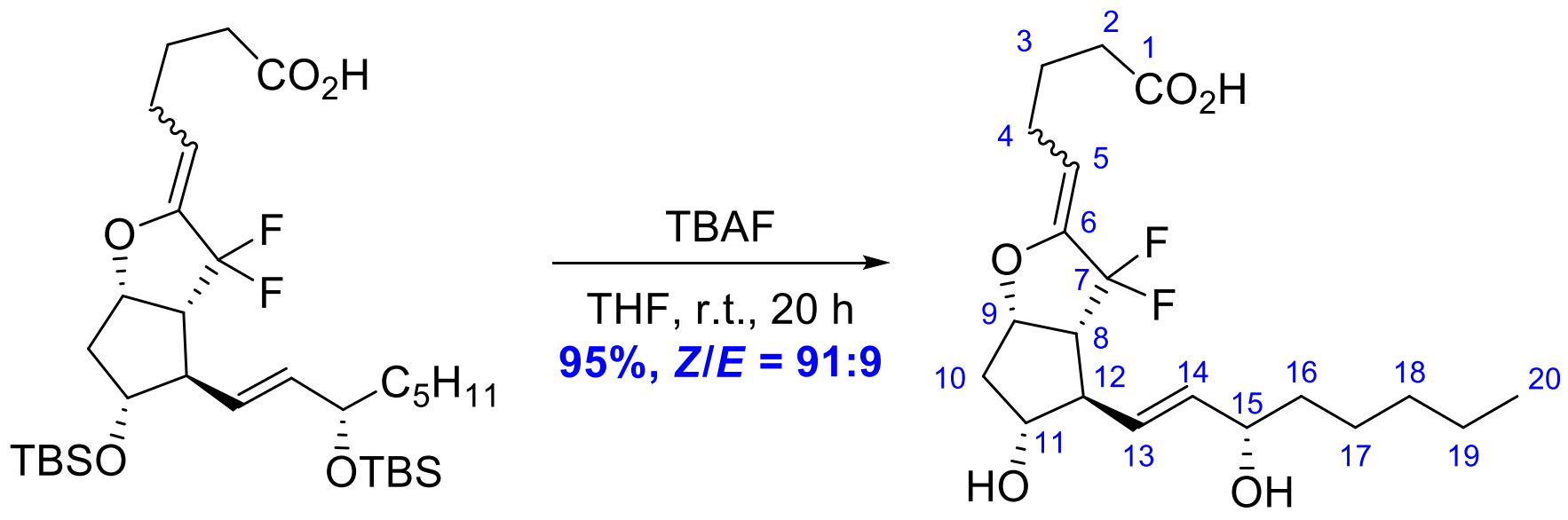

38 **6**

To a stirring solution of **38** (19.0 mg, 30.8 μmol, 1.0 eq.) in THF (0.31 mL) at 0 °C, TBAF (1.0 M in THF, 92.4 μL, 92.4 mmol, 3.0 eq.) was added dropwise. The reaction mixture was warmed to r.t. and stirred for a further 20 h. The reaction was quenched by addition of H_2O (1 mL) and the aqueous phase was extracted with Et_2O (3 × 3 mL). The combined organic phases were washed with brine, dried over $MgSO_4$, filtered and concentrated *in vacuo*. The crude product was purified by flash column chromatography on silica gel (CH_2Cl_2/EtOH/AcOH = 97:2:1 to 91:8:1) to give **6** as a colourless oil (11.4 mg, 30.8 μmol, 95%, 91:9 *Z*/*E*).

***R*f** = 0.24 (CH_2Cl_2/EtOH/AcOH = 95:4:1)

IR (thin film) ν_{max}/cm^{-1}: 3319, 2935, 2864, 1708, 1559, 1413, 1327, 1295, 1242, 1123, 1038, 967, 835, 775.

^{1}H NMR (500 MHz, $CDCl_3$) (*Z*)-**6**: δ_H = 5.61 (dd, *J* = 15.2, 7.6 Hz, 1H, ***H***C14), 5.49 (dd, *J* = 15.3, 8.3 Hz, 1H, ***H***C13), 4.84 (overlapping, br.s+br.s, 2H, O***H***+O***H***), 4.82 (ddd, *J* = 8.8, 6.5, 5.1 Hz, 1H, ***H***C5), 4.74 (td, *J* = 6.5, 3.0 Hz, 1H, ***H***C9), 4.06 (q, *J* = 7.0 Hz, 1H, ***H***C11), 3.86 (q, *J* = 8.4 Hz, 1H, ***H***C15), 2.62 – 2.55 (m, 1H, ***H***C8), 2.53 – 2.45 (m, 2H, ***H**A*C10+***H***C12), 2.38 – 2.33 (m, 2H, ***H2***C2), 2.30 – 2.22 (m, 1H, ***H**A*C4), 2.13 – 2.07 (m, 1H, ***H**B*C4), 1.87 – 1.79 (m, 2H, ***H**A*C3+***H**B*C10), 1.74 – 1.67 (m, 1H, ***H**B*C3), 1.59 – 1.54 (m, 1H, ***H**A*C16), 1.50 – 1.42 (m, 1H, ***H**B*C16), 1.31 – 1.28 (m, 6H, ***H2***C17+***H2***C18+***H2***C19), 0.89 (t, *J* = 6.8 Hz, 3H, ***H3***C20).

^{19}**F NMR** (470 MHz, $CDCl_3$) (*Z*)-**6**: δ_F = -84.15 (dd, $^2J_{FF}$ = 250.8 Hz, $^3J_{HF}$ = 15.1 Hz), -117.84 (d, $^2J_{FF}$ = 251.0 Hz); (*E*)-**6**: δ_F = -85.09 (dd, $^2J_{FF}$ = 259.2 Hz, $^3J_{HF}$ = 15.4 Hz), -110.62 (d, $^2J_{FF}$ = 259.0 Hz).

^{13}**C NMR** (125 MHz, $CDCl_3$) (*Z*)-**6**: δ_C = 178.1 (**C1**), 149.2 (dd, $^2J_{CF}$ = 30.5 Hz, $^2J_{CF}$ = 25.6 Hz, **C6**), 136.6 (**C14**), 130.7 (**C13**), 124.07 (dd, $^1J_{CF}$ = 254.6 Hz, $^1J_{CF}$ = 241.1 Hz, **C7**), 100.5 (d, $^3J_{CF}$ = 4.6 Hz, **C5**), 81.1 (d, $^3J_{CF}$ = 6.2 Hz, **C9**), 77.3 (d, $^4J_{CF}$ = 1.8 Hz, **C11**), 73.5 (**C15**), 52.0 (dd, $^2J_{CF}$ = 25.6 Hz, $^2J_{CF}$ = 19.3 Hz, **C8**), 50.7 (d, $^3J_{CF}$ = 4.4 Hz, **C12**), 40.4 (**C10**), 37.0 (**C16**), 33.1 (**C2**), 31.8 (**C17**), 25.1 (**C18**), 23.9 (d, $^4J_{CF}$ = 2.6 Hz, **C4**), 23.6 (d, $^5J_{CF}$ = 1.3 Hz, **C3**), 22.7 (**C19**), 14.1 (**C20**).

HRMS (ESI^+) calculated for $C_{20}H_{30}F_2NaO_5$ [$M+Na^+$] 411.1954, found 411.1972.

(3a*R*,4*R*,5*R*,6a*S*)-5-(*tert*-Butyldimethylsilyloxy)-4-((*3*S,4*S*,*E*)-3-(tert-butyldimethylsilyloxy)-4-methyloct-1-en-6-ynyl)-3,3-difluorohexahydro-2*H*-cyclopenta[*b*]furan-2-one (39)

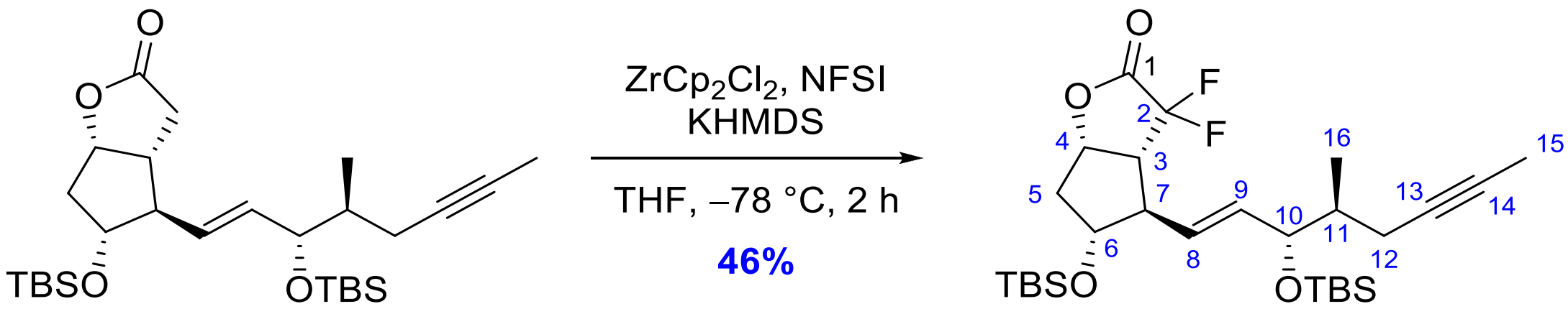

28 **39**

A solution of NFSI (883 mg, 2.80 mmol, 4.0 eq.) and $ZrCp_2Cl_2$ (818 mg, 2.80 mmol, 4.0 eq.) in THF (8.8 mL) was stirred at r.t. for 1 h and then cooled to –78 °C. A solution of **28** (355 mg, 0.70 mmol, 1.0 eq.) in THF (4.7 mL) was added followed by the drop-wise addition of KHMDS (1.0 M in THF, 3.50 mL, 3.50 mmol, 5.0 eq.) over 1 h, the reaction mixture was then stirred at –78 °C for a further hour. The reaction mixture was warmed to r.t., quenched by addition of saturated $NaHCO_3$ solution (15 mL) and the aqueous phase was subsequently extracted with Et_2O (3 × 15 mL). The organic phases were combined, washed with brine, dried over $MgSO_4$, filtered and concentrated *in vacuo*. The crude product was purified by flash column chromatography on silica gel (Hexanes/Et_2O = 100:0 to 90:10) to give pure **39** as a white foam (175 mg, 0.32 mmol, 46%).

Mp 58–61 °C

$\boldsymbol{R_f}$ = 0.25 (Hexanes/Et_2O = 90:10)

IR (thin film) ν_{max}/cm^{-1}: 2955, 2929, 2895, 2857, 1755, 1474, 1360, 1250, 1162, 1031, 968, 838, 775.

^{1}H NMR (400 MHz, $CDCl_3$) δ_H = 5.51 (ddd, J = 15.5, 6.6, 0.8 Hz, 1H, $\boldsymbol{H}C_9$), 5.37 (dd, J = 15.5, 7.7 Hz, 1H, $\boldsymbol{H}C_8$), 5.15 (t, J = 6.0 Hz, 1H, $\boldsymbol{H}C_4$), 4.09 – 4.06 (m, 1H, $\boldsymbol{H}C_6$), 3.98 – 3.95 (m, 1H, $\boldsymbol{H}C_{10}$), 3.04 – 3.03 (m, 1H, $\boldsymbol{H}C_7$), 2.95 (ddd, J = 25.8, 6.9, 3.1 Hz, 1H, $\boldsymbol{H}C_3$), 2.19 – 2.10 (m, 3H, $\boldsymbol{H_A}C_5$+ $\boldsymbol{H_A}C_{12}$+$\boldsymbol{H_B}C_{12}$), 2.09 – 2.04 (m, 1H, $\boldsymbol{H_B}C_5$), 1.77 (t, J = 2.5 Hz, 3H, $\boldsymbol{H_3}C_{15}$), 1.71 – 1.64 (m, 1H, $\boldsymbol{H}C_{11}$), 0.89 – 0.85 (m, 21H, SiC(C$\boldsymbol{H_3}$)$_3$+SiC(C$\boldsymbol{H_3}$)$_3$+$\boldsymbol{H_3}C_{16}$), 0.04 (s, 3H, SiC$\boldsymbol{H_3}$), 0.04 (s, 6H, SiC$\boldsymbol{H_3}$+SiC$\boldsymbol{H_3}$), -0.02 (s, 3H, SiC$\boldsymbol{H_3}$).

^{19}F NMR (377 MHz, $CDCl_3$) δ_F = -91.66 (dd, $^2J_{FF}$ = 279.9 Hz, $^3J_{HF}$ = 25.8 Hz), -113.57 (d, $^2J_{FF}$ = 279.9 Hz).

^{13}C NMR (100 MHz, $CDCl_3$) δ_C = 165.4 (dd, $^2J_{CF}$ = 35.3 Hz, $^2J_{CF}$ = 32.7 Hz, **C$_1$**), 134.5 (**C$_9$**), 129.0 (**C$_8$**), 116.2 (dd, $^1J_{CF}$ = 265.0 Hz, $^1J_{CF}$ = 243.9 Hz, **C$_2$**), 81.8 (dd, $^3J_{CF}$ = 3.4 Hz, $^3J_{CF}$ = 2.1 Hz, **C$_4$**), 78.4 (**C$_{14}$**), 77.6 (**C$_6$**), 76.8 (**C$_{13}$**), 75.9 (**C$_{10}$**), 51.6 (dd, $^3J_{CF}$ = 5.3 Hz, $^3J_{CF}$ = 2.4 Hz, **C$_7$**), 50.0 (dd, $^2J_{CF}$ = 23.4 Hz, $^2J_{CF}$ = 19.0 Hz, **C$_3$**), 40.1 (**C$_5$**), 39.5 (**C$_{11}$**), 26.0 (SiC(***C***H$_3$)$_3$), 25.8 (SiC(***C***H$_3$)$_3$), 22.0 (**C$_{12}$**), 18.3 (Si***C***(CH$_3$)$_3$), 18.2 (Si***C***(CH$_3$)$_3$), 15.5 (**C$_{16}$**), 3.6 (**C$_{15}$**), -4.0 (Si***C***H$_3$), -4.7 (Si***C***H$_3$), -5.0 (Si***C***H$_3$), -5.0 (Si***C***H$_3$).

HRMS (ESI$^+$) calculated for $C_{28}H_{48}F_2NaO_4Si_2$ [M+Na$^+$] 565.2951, found 565.2933.

$[\alpha]_D^{22}$ = –8.3 (c 1.00, $CHCl_3$)

5-((3a*R*,4*R*,5*R*,6a*S*)-5-(*tert*-Butyldimethylsilyloxy)-4-((3*S*,4*S*,*E*)-3-(*tert*-butyldimethylsilyloxy)-4-methyloct-1-en-6-ynyl)-3,3-difluorohexahydro-2*H*-cyclopenta[*b*]furan-2-ylidene)pentanoic acid (40)

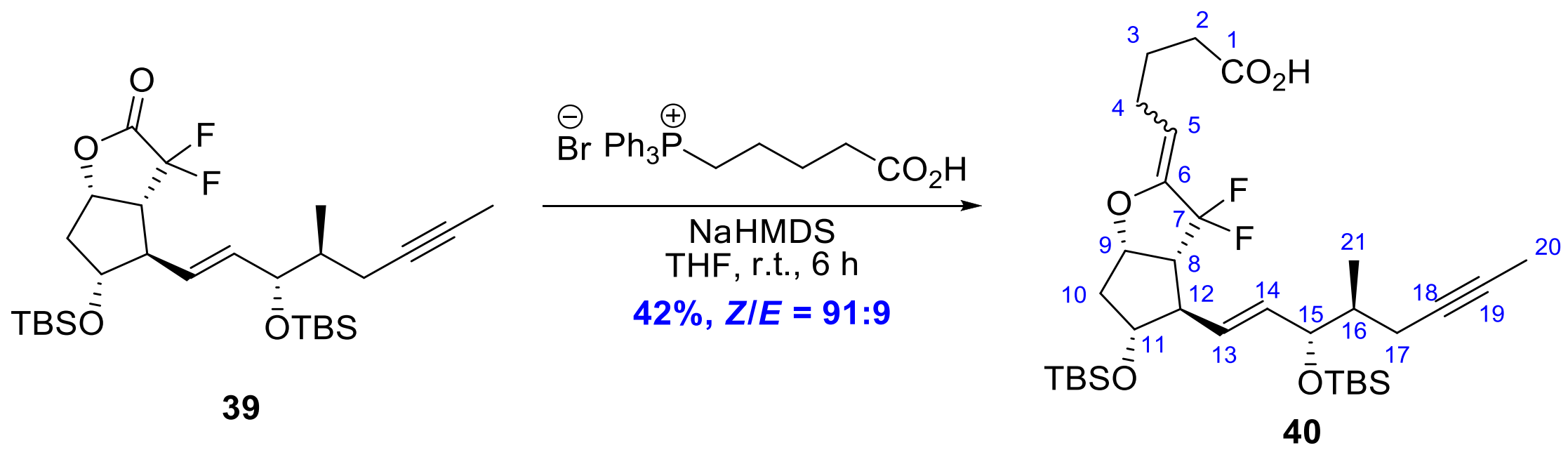

NaHMDS (1.0 M in THF, 0.50 mL, 0.50 mmol, 10.0 eq.) was added to a stirring suspension of (4-carboxybutyl)triphenylphosphonium bromide (111 mg, 0.25 mmol, 5.0 eq.) in THF (0.34 mL). The resulting orange suspension was heated to 70 °C for 1 h before cooling to r.t.. **39** (27.1 mg, 51.0 µmol 1.0 eq.) was added as a solution in THF (50 µL) to the orange suspension and the reaction was stirred at r.t. for 6 h. The reaction mixture was quenched by addition of water (5 mL) and extracted with EtOAc (3 × 5 mL). The combined organic phases were washed with water (10 mL), treated with aqueous HCl (1.0 M, 10 mL), washed with brine, dried over

$MgSO_4$, filtered and concentrated *in vacuo*. The crude product was purified by flash column chromatography on silica gel (Hexanes/EtOAc = 95:5 to 90:10; then Hexanes/EtOAc/AcOH = 90:9:1) to give **40** as a colourless oil (13.2 mg, 21.1 µmol, 42%, 91:9 *Z*/*E*).

$\boldsymbol{R_f}$ = 0.40 (Hexanes/EtOAc/AcOH = 80:19:1)

IR (thin film) $\boldsymbol{\nu_{max}}$/cm^{-1}: 2955, 2929, 2857, 1788, 1474, 1254, 1190, 1084, 1005, 890, 833, 774.

^{1}H NMR (400 MHz, $CDCl_3$) (*Z*)-**40**: δ_H = 5.54 – 5.52 (m, 2H, ***H***C13+***H***C14), 4.81 (td, *J* = 7.5, 4.8 Hz, 1H, ***H***C5), 4.76 (td, *J* = 6.6, 3.3 Hz, 1H, ***H***C9), 4.02 – 3.99 (m, 1H, ***H***C11), 3.90 (q, *J* = 7.4 Hz, 1H, ***H***C15), 2.67 – 2.63 (m, 1H, ***H***C12), 2.62 – 2.55 (m, 1H, ***H***C8), 2.43 – 2.35 (m, 3H, $\boldsymbol{H_2}$C2+$\boldsymbol{H_A}$C10), 2.21 – 2.13 (m, 3H, $\boldsymbol{H_A}$C4+ $\boldsymbol{H_A}$C17+$\boldsymbol{H_B}$C17), 2.11 – 2.03 (m, 1H, $\boldsymbol{H_B}$C4), 1.86 – 1.81 (m, 1H, $\boldsymbol{H_B}$C10), 1.78 (t, *J* = 2.5 Hz, 3H, $\boldsymbol{H_3}$C20), 1.77 – 1.73 (m, 2H, $\boldsymbol{H_A}$C3+***H***C16), 1.70 – 1.65 (m, 1H, $\boldsymbol{H_B}$C3), 0.92 – 0.88 (m, 12H, SiC(C$\boldsymbol{H_3}$)$_3$+ $\boldsymbol{H_3}$C21), 0.87 (s, 9H, SiC(C$\boldsymbol{H_3}$)$_3$), 0.05 (s, 3H, SiC$\boldsymbol{H_3}$), 0.04 (s, 3H, SiC$\boldsymbol{H_3}$), 0.04 (s, 3H, SiC$\boldsymbol{H_3}$), 0.01 (s, 3H, SiC$\boldsymbol{H_3}$).

^{19}F NMR (377 MHz, $CDCl_3$) (*Z*)-**40**: δ_F = -82.53 (dddd, $^2J_{FF}$ = 251.7 Hz, $^3J_{HF}$ = 17.1 Hz, $^4J_{HF}$ = 9.0 Hz, $^4J_{HF}$ = 4.4 Hz), -114.29 (d, $^2J_{FF}$ = 251.4 Hz); (*E*)-**40**: δ_F = -84.16 (ddd, $^2J_{FF}$ = 259.7 Hz, $^3J_{HF}$ = 19.8 Hz, $^4J_{HF}$ = 3.3 Hz), -107.64 (d, $^2J_{FF}$ = 259.3 Hz).

^{13}C NMR (100 MHz, $CDCl_3$) (*Z*)-**40**: δ_C = 179.2 (**C1**), 149.6 (dd, $^2J_{CF}$ = 30.3 Hz, $^2J_{CF}$ = 25.6 Hz, **C6**), 133.8 (**C14**), 129.7 (**C13**), 124.2 (dd, $^1J_{CF}$ = 254.9 Hz, $^1J_{CF}$ = 240.4 Hz, **C7**), 99.9 (d, $^3J_{CF}$ = 4.5 Hz, **C5**), 81.6 (d, $^3J_{CF}$ = 5.4 Hz, **C9**), 78.1 (**C19**), 78.1 (**C11**), 76.6 (**C18**), 75.9 (**C15**), 51.6 (dd, $^2J_{CF}$ = 25.7 Hz, $^2J_{CF}$ = 19.5 Hz, **C8**), 49.9 (d, $^3J_{CF}$ = 4.1 Hz, **C12**), 41.9 (**C10**), 39.9 (**C16**), 33.5 (**C2**), 26.0 (SiC(***C***H$_3$)$_3$), 25.9 (SiC(***C***H$_3$)$_3$), 24.3 (d, $^4J_{CF}$ = 2.9 Hz, **C4**), 24.1 (d, $^5J_{CF}$ = 1.2 Hz, **C3**), 22.2 (**C17**), 18.3 (Si***C***(CH$_3$)$_3$), 18.1 (Si***C***(CH$_3$)$_3$), 15.5 (**C21**), 3.6 (**C20**), -4.0 (Si***C***H$_3$), -4.4 (Si***C***H$_3$), -4.6 (Si***C***H$_3$), -4.8 (Si***C***H$_3$).

HRMS (ESI$^+$) calculated for $C_{33}H_{56}F_2NaO_5Si_2$ [M+Na$^+$] 649.3527, found 649.3539.

5-((3a*R*,4*R*,5*R*,6a*S*)-3,3-Difluoro-5-hydroxy-4-((3*S*,4*S*,*E*)-3-hydroxy-4-methyloct-1-en-6-ynyl)hexahydro-2*H*-cyclopenta[*b*]furan-2-ylidene)pentanoic acid (7)

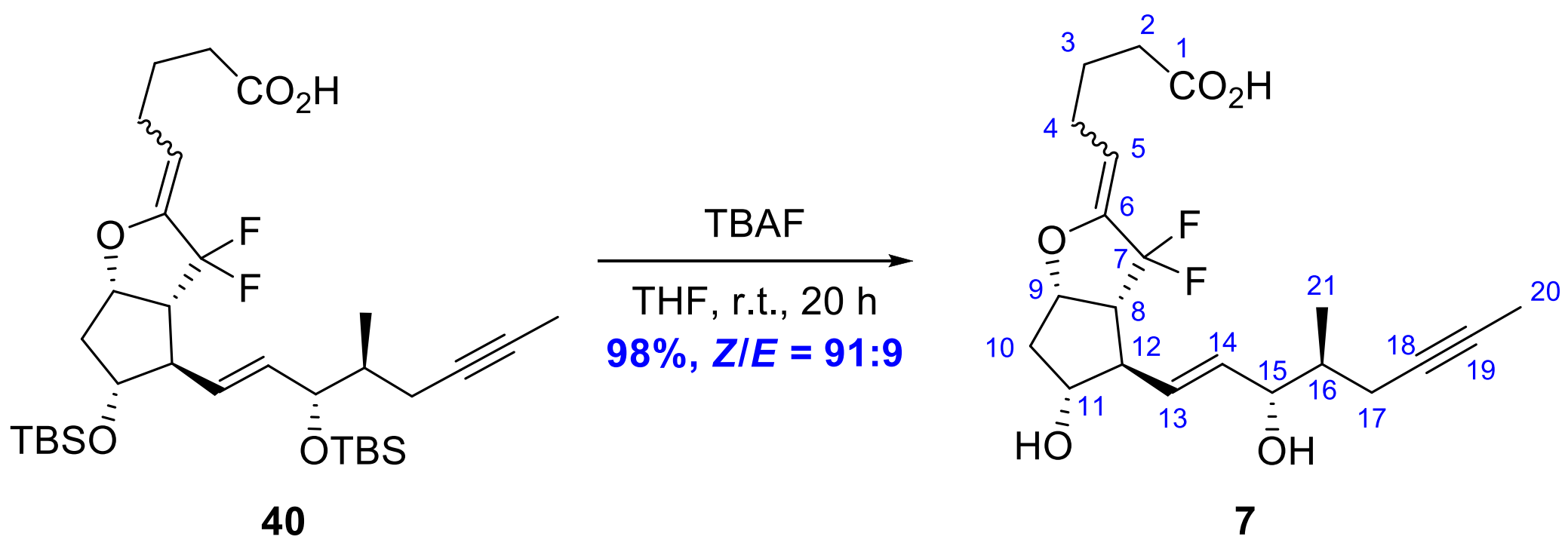

To a stirring solution of **40** (23.7 mg, 37.8 µmol, 1.0 eq.) in THF (0.38 mL) at 0 °C, TBAF (1.0 M in THF, 113 µL, 113 mmol, 3.0 eq.) was added dropwise. The reaction mixture was warmed to r.t. and stirred for a further 20 h. The reaction was quenched by addition of H_2O (1 mL) and the aqueous phase was extracted with Et_2O (3 × 3 mL). The combined organic phases were washed with brine, dried over $MgSO_4$, filtered and concentrated *in vacuo*. The crude product was purified by flash column chromatography on silica gel (CH_2Cl_2/EtOAc/AcOH = 97:2:1 to 91:8:1) to give **7** as a colourless oil (14.8 mg, 37.1 µmol, 98%, 91:9 *Z*/*E*).

$\boldsymbol{R_f}$ = 0.24 (CH_2Cl_2/EtOAc/AcOH = 95:4:1)

IR (thin film) $\boldsymbol{\nu_{max}}$/cm^{-1}: 3375, 2956, 2925, 2857, 1706, 1558, 1458, 1298, 1240, 1140, 1090, 1005, 970, 910, 806.

^{1}H NMR (500 MHz, $CDCl_3$) (*Z*)-**7**: δ_H = 5.62 (dd, J = 15.3, 7.8 Hz, 1H, $\boldsymbol{H}C_{14}$), 5.52 (dd, J = 15.2, 8.1 Hz, 1H, $\boldsymbol{H}C_{13}$), 4.92 – 4.56 (overlapping, br.s+ br.s, 2H, O***H***+O***H***), 4.84 – 4.80 (m, 1H, $\boldsymbol{H}C_5$), 4.74 (td, J = 6.3, 2.4 Hz, 1H, $\boldsymbol{H}C_9$), 3.97 (t, J = 7.6 Hz, 1H, $\boldsymbol{H}C_{11}$), 3.89 (q, J = 8.1 Hz, 1H, $\boldsymbol{H}C_{15}$), 2.64 – 2.47 (m, 3H, $\boldsymbol{H}C_8$+$\boldsymbol{H_A}C_{10}$+$\boldsymbol{H}C_{12}$), 2.37 – 2.35 (t, J = 6.3 Hz, 2H, $\boldsymbol{H_2}C_2$), 2.28 – 2.21 (m, 3H, $\boldsymbol{H_A}C_4$+ $\boldsymbol{H_A}C_{17}$+$\boldsymbol{H_B}C_{17}$), 2.13 – 2.07 (m, 1H, $\boldsymbol{H_B}C_4$), 1.87 – 1.81 (m, 2H, $\boldsymbol{H_B}C_{10}$+$\boldsymbol{H}C_{16}$), 1.79 (t, J = 2.5 Hz, 3H, $\boldsymbol{H_3}C_{20}$), 1.76 – 1.66 (m, 2H, $\boldsymbol{H_2}C_3$), 0.93 (d, J = 6.8 Hz, 3H, $\boldsymbol{H_3}C_{21}$).

^{19}F NMR (470 MHz, $CDCl_3$) (*Z*)-**7**: δ_F = -84.16 (dd, $^2J_{FF}$ = 251.2 Hz, $^3J_{FF}$ = 15.5 Hz), -117.93 (d, $^2J_{FF}$ = 251.0 Hz); (*E*)-**7**: δ_F = -85.12 (dd, $^2J_{FF}$ = 260.1 Hz, $^3J_{FF}$ = 16.5 Hz), -110.90 (d, $^2J_{FF}$ = 258.9 Hz).

^{13}C NMR (125 MHz, $CDCl_3$) (*Z*)-**7**: δ_C = 178.3 (**C$_1$**), 149.1 (dd, $^2J_{CF}$ = 30.4 Hz, $^2J_{CF}$ = 25.2 Hz, **C$_6$**), 134.7 (**C$_{14}$**), 131.8 (**C$_{13}$**), 24.1 (dd, $^1J_{CF}$ = 254.5 Hz, $^1J_{CF}$ = 241.0 Hz, **C$_7$**), 100.6 (d, $^3J_{CF}$ = 4.4 Hz, **C$_5$**), 81.1 (d, $^3J_{CF}$ = 6.2 Hz, **C$_9$**), 77.4 (**C$_{19}$**), 77.3 (**C$_{11}$**), 77.3 (**C$_{18}$**), 76.5 (**C$_{15}$**), 52.0 (dd, $^2J_{CF}$ = 25.6 Hz, $^2J_{CF}$ = 19.0 Hz, **C$_8$**), 50.8 (d, $^3J_{CF}$ = 4.3 Hz, **C$_{12}$**), 40.4 (**C$_{10}$**), 38.3 (**C$_{16}$**), 33.3 (**C$_2$**), 24.0 (d, $^4J_{CF}$ = 1.6 Hz, **C$_4$**), 23.7 (d, $^5J_{CF}$ = 0.6 Hz, **C$_3$**), 22.4 (**C$_{17}$**), 15.7 (**C$_{21}$**), 3.7 (**C$_{20}$**).

HRMS (ESI$^+$) calculated for $C_{21}H_{28}F_2NaO_5$ [M+Na$^+$] 421.1797, found 421.1777.

Synthesis of monofluorinated targets 2 and 3.

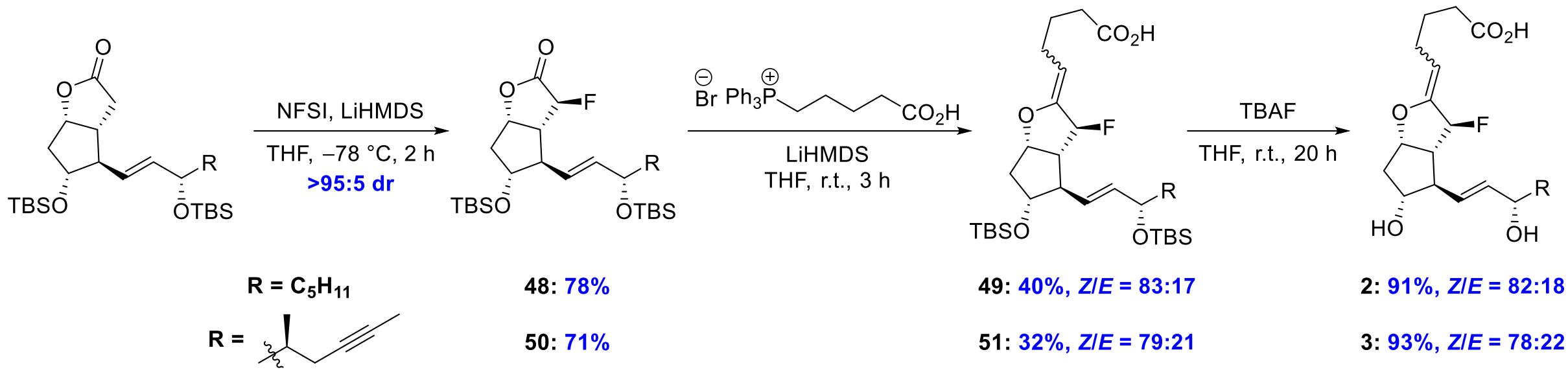

(3*S*,3a*R*,4*R*,5*R*,6a*S*)-5-(*tert*-Butyldimethylsilyloxy)-4-((*S*,*E*)-3-(*tert*-butyldimethylsilyloxy)oct-1-enyl)-3-fluorohexahydro-2*H*-cyclopenta[*b*]furan-2-one (48)

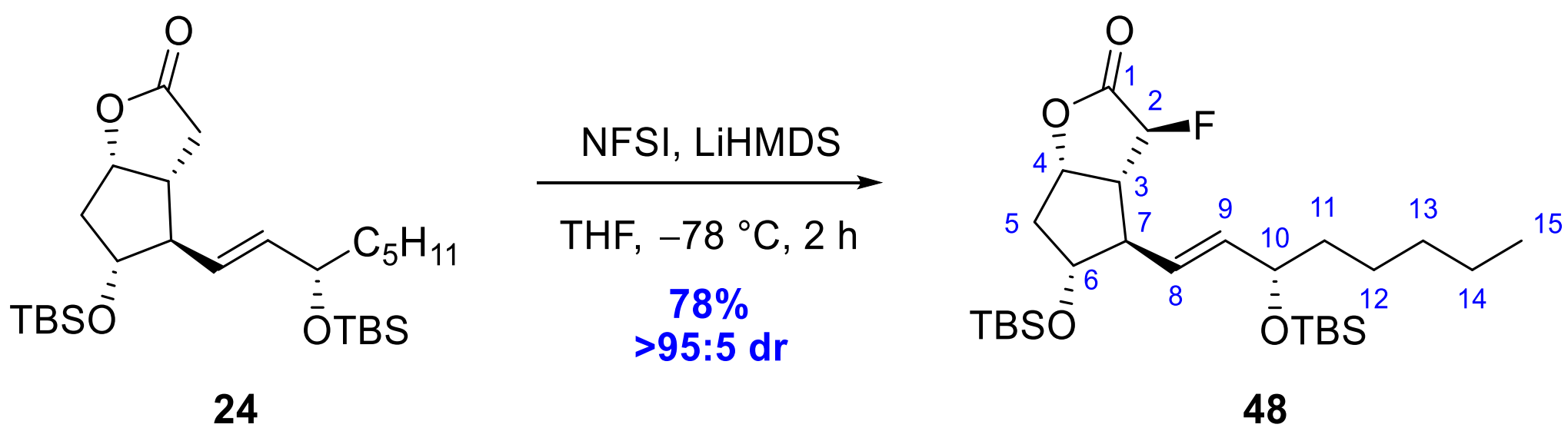

To a solution of **24** (745 mg, 1.50 mmol, 1.0 eq.) and NFSI (591 mg, 1.88 mmol, 1.25 eq.) in THF (7.5 mL) at –78 °C, LiHMDS (1.0 M in THF, 1.73 mL, 1.73 mmol, 1.15 eq.) was added dropwise. The reaction mixture was stirred at –78 °C for 2 h before it was warmed to r.t., quenched by addition of saturated $NaHCO_3$ solution (15 mL) and the aqueous phase was extracted with Et_2O (3 × 15 mL). The organic phases were combined, washed with brine, dried over $MgSO_4$, filtered and concentrated *in vacuo*. The crude product was purified by flash column chromatography on silica gel (Hexanes/Et_2O = 100:0 to 95:5) to give pure **48** as a colourless oil (602 mg, 1.17 mmol, 78%, >95:5 dr).

$\boldsymbol{R_f}$ = 0.38 (Hexanes/Et_2O = 95:5)

IR (thin film) ν_{max}/cm^{-1}: 2956, 2928, 2858, 1788, 1462, 1406, 1393, 1251, 1192, 1077, 1066, 968, 890, 834, 775.

^{1}H NMR (400 MHz, $CDCl_3$) δ_H = 5.51 (ddd, J = 15.5, 5.8, 1.0 Hz, ***H***C9), 5.36 (ddd, J = 15.5, 7.3, 1.2 Hz, 1H, ***H***C8), 5.18 – 5.14 (m, 1H, ***H***C4), 5.08 (dd, J = 51.9, 2.4 Hz, 1H, ***H***C2), 4.07 – 4.06 (m, 1H, ***H***C6), 4.05 – 4.02 (m, 1H, ***H***C10), 2.93 (ddt, J = 32.6, 7.4, 2.5 Hz, 1H, ***H***C3), 2.81 (d, J = 7.0 Hz, 1H, ***H***C7), 2.10 – 1.99 (m, 2H, ***H*2**C5), 1.49 – 1.36 (m, ***H*2**C11), 1.32 – 1.21 (m,

6H, H_2C_{12}+H_2C_{13}+H_2C_{14}), 0.90 – 0.85 (m, 21H, SiC(CH_3)$_3$+SiC(CH_3)$_3$+H_3C_{15}), 0.06 (s, 3H, SiCH_3), 0.05 (s, 3H, SiCH_3), 0.03 (s, 3H, SiCH_3), -0.00 (s, 3H, SiCH_3).

^{19}F NMR (377 MHz, $CDCl_3$) δ_F = -176.91 (dd, $^2J_{HF}$ = 51.8 Hz, $^3J_{HF}$ = 32.5 Hz).

^{13}C NMR (100 MHz, $CDCl_3$) δ_C = 172.0 (d, $^2J_{CF}$ = 21.2 Hz, **C1**) 136.2 (**C9**), 127.4 (**C8**), 92.1 (d, $^1J_{CF}$ = 183.4 Hz, **C2**), 83.2 (d, $^3J_{CF}$ = 2.7 Hz, **C4**), 78.6 (**C6**), 73.0 (**C10**), 55.0 (d, $^3J_{CF}$ = 4.9 Hz, **C7**), 50.2 (d, $^2J_{CF}$ = 20.2 Hz, **C3**), 39.7 (d, $^4J_{CF}$ = 1.5 Hz, **C5**), 38.4 (**C11**), 31.9 (**C12**), 26.0 (SiC(CH_3)$_3$), 25.8 (SiC(CH_3)$_3$), 25.1 (**C13**), 22.7 (**C14**), 18.4 (Si*C*(CH_3)$_3$), 18.2 (Si*C*(CH_3)$_3$), 14.2 (**C15**), -4.2 (Si*C*H_3), -4.5 (Si*C*H_3), -4.8 (Si*C*H_3), -4.9 (Si*C*H_3).

HRMS (ESI$^+$) calculated for $C_{27}H_{51}FNaO_4Si_2$ [M+Na$^+$] 537.3202, found 537.3197.

[*α*]$_D^{22}$ = –29.7 (*c* 0.50, $CHCl_3$)

5-((3*S*,3a*R*,4*R*,5*R*,6a*S*)-5-(*tert*-Butyldimethylsilyloxy)-4-((*S*,*E*)-3-(*tert*-butyldimethylsilyloxy)oct-1-enyl)-3-fluorohexahydro-2*H*-cyclopenta[*b*]furan-2-ylidene)pentanoic acid (49)

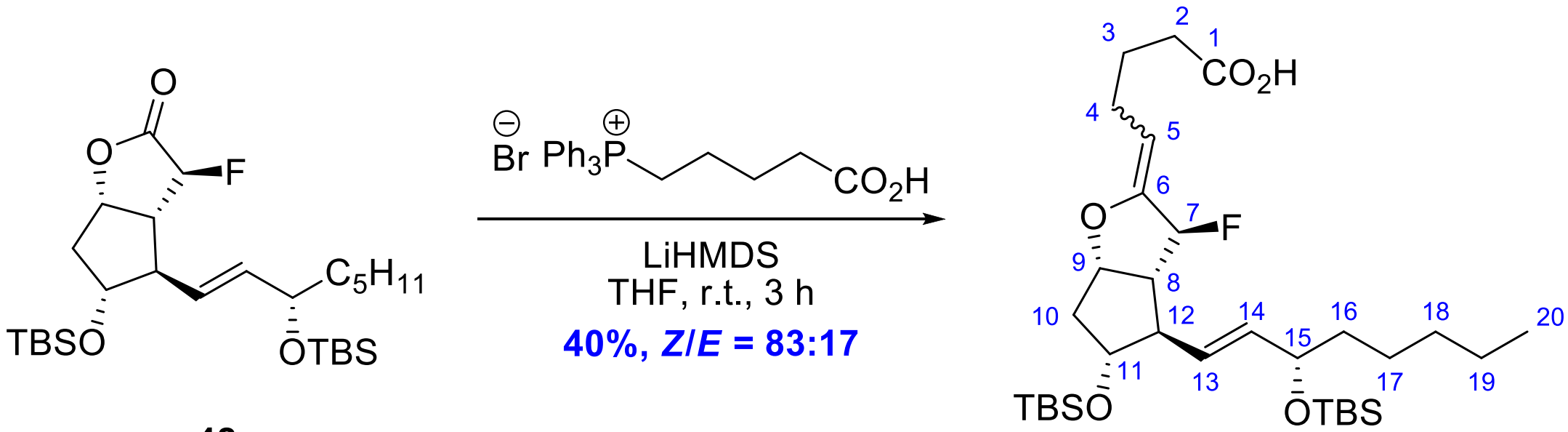

48 **49**

LiHMDS (1.0 M in THF, 0.26 mL, 0.26 mmol, 3.2 eq.) was added to a stirring suspension of (4-carboxybutyl)triphenylphosphonium bromide (53.2 mg, 0.12 mmol, 1.5 eq.) in THF (0.4 mL). The resulting orange suspension was stirred at r.t. for 1 h. **48** (41.2 mg, 80.0 µmol, 1.0 eq.) was added as a solution in THF (0.40 mL) to the orange suspension and the reaction was stirred at r.t. for 3 h. The reaction mixture was quenched by addition of water (8 mL) and extracted with EtOAc (3 × 8 mL). The combined organic phases were washed with water (8 mL), treated with aqueous HCl (0.1 M, 8 mL), washed with brine, dried over $MgSO_4$, filtered and concentrated *in vacuo*. The crude product was purified by flash column chromatography on silica gel (Hexanes/EtOAc = 95:5; then Hexanes/EtOAc/AcOH = 95:4:1) to give **49** as a colourless oil (19.2 mg, 32.1 µmol, 40%, 83:17 *Z*/*E*).

***R*$_f$** = 0.25 (Hexanes/EtOAc/AcOH = 80:19:1)

IR (thin film) ν_{max}/cm^{-1}: 2954, 2929, 2857, 1708, 1462, 1252, 1219, 1125, 1102, 970, 938, 907, 835, 775.

^{1}H NMR (500 MHz, $CDCl_3$) (*Z*)-**49**: δ_H = 5.58 (dd, *J* = 15.4, 5.2 Hz, 1H, ***H***C_{14}), 5.48 (ddd, *J* = 15.4, 7.7, 0.9 Hz, 1H, ***H***C_{13}), 4.91 (d, *J* = 56.0 Hz, 1H, ***H***C_7), 4.80 (td, *J* = 6.7, 2.9 Hz, 1H, ***H***C_5), 4.67 (q, *J* = 7.3 Hz, 1H, ***H***C_9), 4.10 – 4.06 (m, 1H, ***H***C_{11}), 3.86 (q, *J* = 8.0 Hz, 1H, ***H***C_{15}), 2.47 – 2.36 (m, 4H, $\boldsymbol{H_2}C_2$+***H***C_8+$\boldsymbol{H_A}C_{10}$), 2.20 – 2.14 (m, 2H, $\boldsymbol{H_2}C_4$), 2.09 – 2.04 (m, 1H, ***H***C_{12}), 1.77 – 1.71 (m, 3H, $\boldsymbol{H_2}C_3$+ $\boldsymbol{H_B}C_{10}$), 1.48 – 1.40 (m, 2H, $\boldsymbol{H_2}C_{16}$), 1.35 – 1.25 (m, 6H, $\boldsymbol{H_2}C_{17}$+$\boldsymbol{H_2}C_{18}$+$\boldsymbol{H_2}C_{19}$), 0.90 – 0.85 (m, 21H, $SiC(C\boldsymbol{H_3})_3$+$SiC(C\boldsymbol{H_3})_3$+$\boldsymbol{H_3}C_{20}$), 0.04 (s, 3H, $SiC\boldsymbol{H_3}$), 0.03 (s, 3H, $SiC\boldsymbol{H_3}$), 0.02 (s, 3H, $SiC\boldsymbol{H_3}$), 0.02 (s, 3H, $SiC\boldsymbol{H_3}$).

^{19}F NMR (377 MHz, $CDCl_3$) (*Z*)-**49**: δ_F = -158.64 (ddd, $^2J_{HF}$ = 55.4 Hz, $^3J_{HF}$ = 20.7 Hz, $^4J_{HF}$ = 7.3 Hz); (*E*)-**49**: δ_F = -164.31 (ddd, $^2J_{HF}$ = 54.1 Hz, $^3J_{HF}$ = 21.4 Hz, $^4J_{HF}$ = 4.7 Hz).

^{13}C NMR (125 MHz, $CDCl_3$) (*Z*)-**49**: δ_C = 179.4 (**C_1**), 153.9 (d, $^2J_{CF}$ = 14.7 Hz, **C_6**), 136.7 (**C_{14}**), 128.1 (**C_{13}**), 103.8 (d, $^3J_{CF}$ = 10.6 Hz, **C_5**), 95.0 (d, $^1J_{CF}$ = 176.0 Hz, **C_7**), 82.9 (**C_9**), 78.0 (d, $^4J_{CF}$ = 2.7 Hz, **C_{11}**), 72.8 (**C_{15}**), 51.9 (d, $^2J_{CF}$ = 21.4 Hz, **C_8**), 51.0 (d, $^3J_{CF}$ = 6.5 Hz, **C_{12}**), 41.6 (**C_{10}**), 38.6 (**C_{16}**), 33.6 (**C_2**), 32.0 (**C_{17}**), 26.1 ($SiC(\boldsymbol{C}H_3)_3$), 25.9 ($SiC(\boldsymbol{C}H_3)_3$), 25.2 (**C_{18}**), 24.9 (d, $^5J_{CF}$ = 3.8 Hz, **C_3**), 24.5 (d, $^4J_{CF}$ = 5.2 Hz, **C_4**), 22.8 (**C_{19}**), 18.2 ($Si\boldsymbol{C}(CH_3)_3$), 18.2 ($Si\boldsymbol{C}(CH_3)_3$), 14.2 (**C_{20}**), -4.1 ($Si\boldsymbol{C}H_3$), -4.4 ($Si\boldsymbol{C}H_3$), -4.5 ($Si\boldsymbol{C}H_3$), -4.6 ($Si\boldsymbol{C}H_3$).

HRMS (ESI$^+$) calculated for $C_{32}H_{59}FNaO_5Si_2$ [M+Na$^+$] 621.3777, found 621.3779.

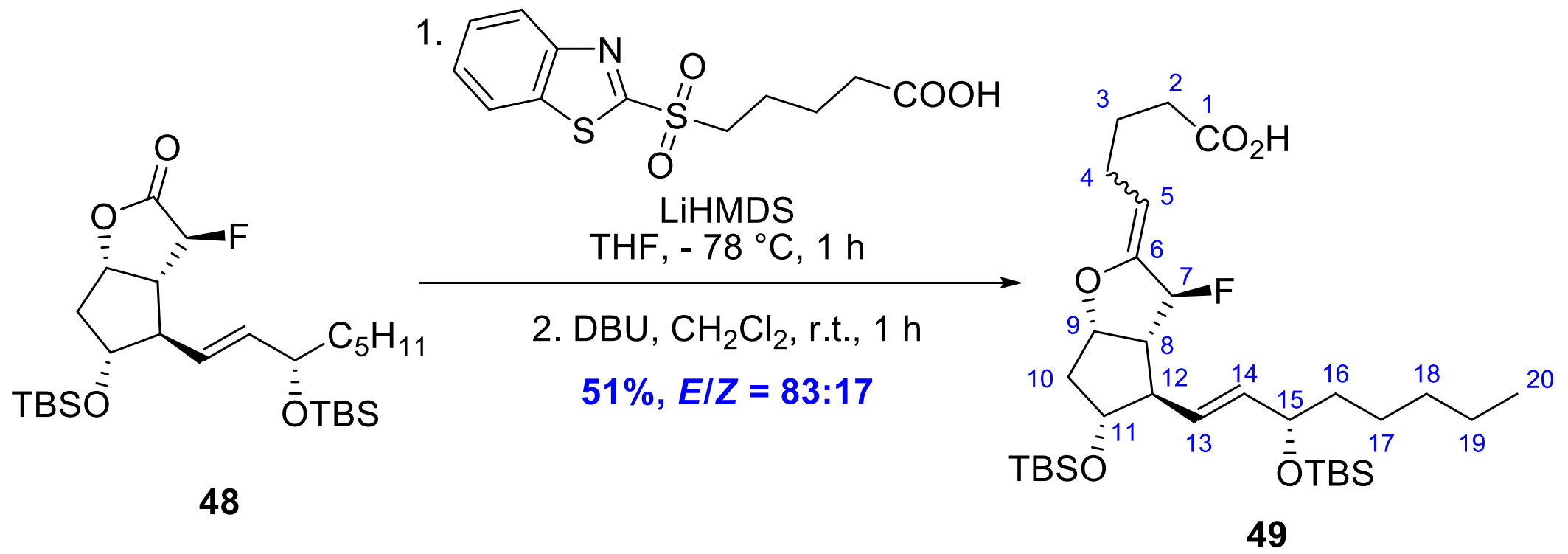

To a solution of **48** (25.7 mg, 50.0 μmol, 1.0 eq.) and (5-(benzo[*d*]thiazol-2-ylsulfonyl)pentanoic acid (20.0 mg, 60.0 μmol, 1.20 eq.) in THF (0.2 mL) at –78 °C, LiHMDS (1.0 M in THF, 0.18 mL, 0.18 mmol, 3.60 eq.) was added dropwise. The resulting bright yellow solution was stirred at –78 °C for 1 h, quenched by addition of AcOH (32.0 μL, 33.0 mg, 0.55 mmol, 11.0 eq.) and warmed to r.t. The reaction mixture was poured over H_2O (5 mL) and the aqueous phase was extracted with EtOAc (3 × 10 mL). The combined organic phases were washed with brine, dried over $MgSO_4$, filtered and concentrated *in vacuo*. The obtained crude

material was dissolved in CH_2Cl_2 (1.0 mL) and DBU (15 µL, 15 mg, 0.1 mmol, 2.0 eq.) was added drop-wise. The bright orange reaction mixture was stirred at r.t. for 1 h before H_2O (5 mL) was added and the aqueous phase was extracted with EtOAc (3 × 5 mL). The combined organic phases were washed with brine, dried over $MgSO_4$, filtered and concentrated *in vacuo*. The crude product was purified by flash column chromatography on silica gel (Hexanes/EtOAc = 95:5; then Hexanes/EtOAc/AcOH = 95:4:1) to give **49** as a colourless oil (15.3 mg, 25.5 µmol, 51%, 83:17 *E*/*Z*).

^{1}H NMR (500 MHz, CD_2Cl_2) (*E*)-**49**: δ_H = 5.59 (dd, *J* = 15.5, 4.9 Hz, 1H, ***H***C14), 5.51 (dd, *J* = 15.5, 7.6 Hz, 1H, ***H***C13), 5.25 (d, *J* = 55.3 Hz, 1H, ***H***C7), 4.97 (q, *J* = 7.4 Hz, 1H, ***H***C9), 4.73 (td, *J* = 6.5, 2.6 Hz, 1H, ***H***C5), 4.14 – 4.10 (m, 1H, ***H***C11), 3.93 – 3.88 (m, 1H, ***H***C15), 2.52 – 2.44 (m, 1H, ***H***C8), 2.40 – 2.35 (m, 3H, ***H₂***C2+***H_A***C10), 2.16 – 2.12 (m, 2H, ***H₂***C4), 2.10 – 2.04 (m, 1H, ***H***C12), 1.73 – 1.67 (m, 3H, ***H₂***C3+ ***H_B***C10), 150 – 1.42 (m, 2H, ***H₂***C16), 1.33 – 1.26 (m, 6H, ***H₂***C17+***H₂***C18+***H₂***C19), 0.91 – 0.85 (m, 21H, SiC(C***H₃***)3+SiC(C***H₃***)3+***H₃***C20), 0.06 (s, 3H, SiC***H₃***), 0.04 (s, 3H, SiC***H₃***), 0.03 (s, 3H, SiC***H₃***), 0.03 (s, 3H, SiC***H₃***).

^{19}F NMR (470 MHz, CD_2Cl_2) (*Z*)-**49**: δ_F = -159.17 (ddd, $^2J_{HF}$ = 56.1 Hz, $^3J_{HF}$ = 20.7 Hz, $^4J_{HF}$ = 7.0 Hz); (*E*)-**49**: δ_F = -164.83 (ddd, $^2J_{HF}$ = 55.3 Hz, $^3J_{HF}$ = 21.7 Hz, $^4J_{HF}$ = 5.3 Hz).

^{13}C NMR (125 MHz, CD_2Cl_2) (*E*)-**49**: δ_C = 178.8 (**C1**), 154.9 (d, $^2J_{CF}$ = 14.6 Hz, **C6**), 137.0 (**C14**), 128.5 (**C13**), 103.6 (d, $^3J_{CF}$ = 10.0 Hz, **C5**), 91.8 (d, $^1J_{CF}$ = 175.5 Hz, **C7**), 82.5 (**C9**), 78.2 (**C11**), 73.1 (**C15**), 52.5 (d, $^2J_{CF}$ = 20.5 Hz, **C8**), 51.7 (d, $^3J_{CF}$ = 6.4 Hz, **C12**), 41.6 (**C10**), 38.8 (**C16**), 33.3 (**C2**), 32.3 (**C17**), 26.9 (d, $^5J_{CF}$ = 2.4 Hz, **C3**), 26.1 (SiC(*C*H3)3), 25.8 (SiC(*C*H3)3), 26.0 (d, $^4J_{CF}$ = 3.7 Hz, **C4**), 25.5 (C**18**), 23.0 (**C19**), 18.5 (Si*C*(CH3)3), 18.3 (Si*C*(CH3)3), 14.2 (**C20**), -4.1 (Si*C*H3), -4.5 (Si*C*H3), -4.6 (Si*C*H3), -4.6 (Si*C*H3).

HRMS (ESI$^+$) calculated for $C_{32}H_{59}FNaO_5Si_2$ [M+Na$^+$] 621.3777, found 621.3775.

5-((3*S*,3a*R*,4*R*,5*R*,6a*S*)-3-Fluoro-5-hydroxy-4-((*S*,*E*)-3-hydroxyoct-1-enyl)hexahydro-2*H*-cyclopenta[*b*]furan-2-ylidene)pentanoic acid (2)

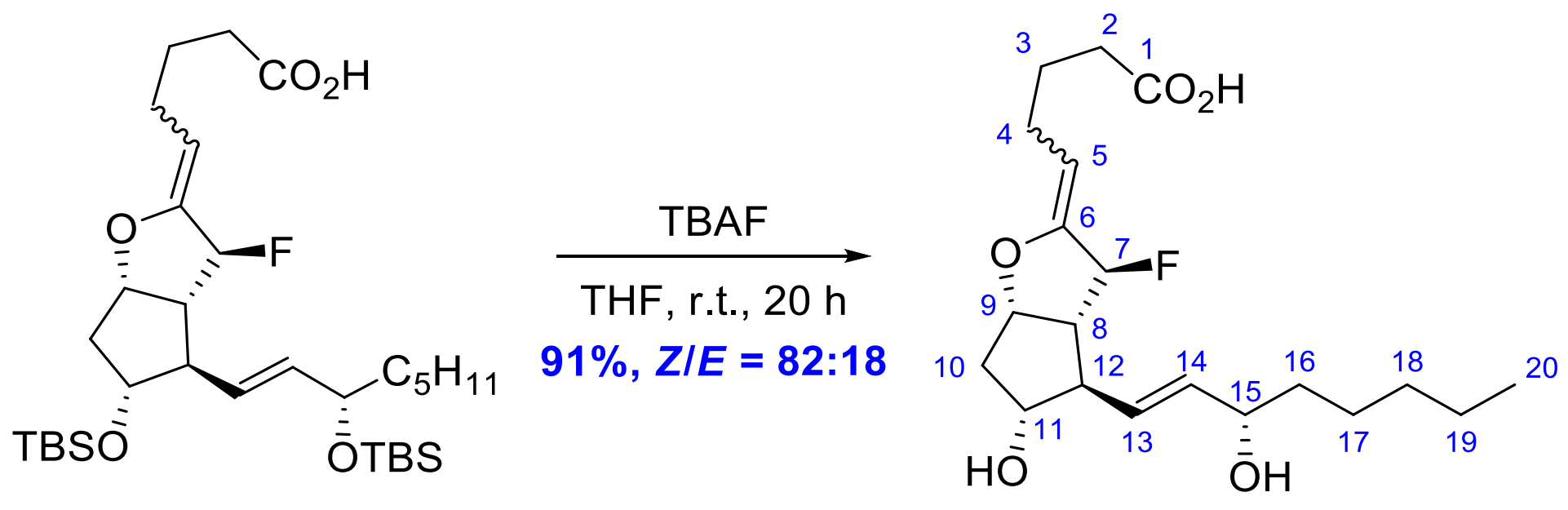

49 **2a**

To a stirring solution of **49** (12.0 mg, 20.0 μmol, 1.0 eq., 83:17 *Z*/*E*) in THF (0.2 mL) at 0 °C, TBAF (1.0 M in THF, 60.0 μL, 60.0 mmol, 3.0 eq.) was added dropwise. The reaction mixture was warmed to r.t. and stirred for a further 20 h. The reaction was quenched by addition of H_2O (1 mL) and the aqueous phase was extracted with Et_2O (3 × 3 mL). The combined organic phases were washed with brine, dried over $MgSO_4$, filtered and concentrated *in vacuo*. The crude product was purified by flash column chromatography on silica gel (CH_2Cl_2/EtOAc/AcOH = 60:39:1 to 40:59:1) to give **2a** as a colourless oil (6.80 mg, 18.4 μmol, 91%, 82:18 *Z*/*E*).

$\boldsymbol{R}_\mathbf{f}$ = 0.17 (CH_2Cl_2/EtOAc/AcOH = 40:59:1)

IR (thin film) $\boldsymbol{\nu}_{\mathbf{max}}$/cm^{-1}: 3384, 2955, 2925, 2863, 1703, 1454, 1431, 1392, 1343, 1251, 1125, 1173, 1097, 969, 774.

^{1}H NMR (500 MHz, CD_2Cl_2+CD_3OD) (*Z*)-**2**: δ_H = 5.59 – 5.53 (m, 1H, ***H***C$_{14}$), 5.53 – 5.47 (m, 1H, ***H***C$_{13}$), 4.89 (d, *J* = 55.5 Hz, 1H, ***H***C$_7$), 4.79 (t, *J* = 6.0 Hz, 1H, ***H***C$_5$), 4.72 (dt, *J* = 8.2, 4.1 Hz, 1H, ***H***C$_9$), 4.01 (q, *J* = 6.5 Hz, 1H, ***H***C$_{11}$), 3.86 – 3.80 (m, 1H, ***H***C$_{15}$), 2.56 – 2.41 (m, 2H, ***H***C$_8$+$\boldsymbol{H_A}$C$_{10}$), 2.30 – 2.23 (m, 2H, $\boldsymbol{H_2}$C$_2$), 2.19 – 2.01 (m, 2H, $\boldsymbol{H_2}$C$_4$), 1.95 – 1.89 (m, 1H, ***H***C$_{12}$), 1.76 – 1.64 (m, 3H, $\boldsymbol{H_2}$C$_3$+$\boldsymbol{H_B}$C$_{10}$), 1.55 – 1.50 (m, 1H, $\boldsymbol{H_A}$C$_{16}$), 1.47 – 1.41 (m, 1H, $\boldsymbol{H_B}$C$_{16}$), 1.29 – 1.24 (m, 6H, $\boldsymbol{H_2}$C$_{17}$+$\boldsymbol{H_2}$C$_{18}$+$\boldsymbol{H_2}$C$_{19}$), 0.89 (d, *J* = 6.7 Hz, 3H, $\boldsymbol{H_3}$C$_{20}$).

^{19}F NMR (470 MHz, CD_2Cl_2); (*Z*)-**2**: δ_F = 160.19 (dd, $^2J_{HF}$ = 54.9 Hz, $^3J_{HF}$ = 10.9 Hz); (*Z*)-**2**: δ_F = -165.51 (dd, $^2J_{HF}$ = 52.0 Hz, $^3J_{HF}$ = 13.2 Hz).

^{13}C NMR (125 MHz, CD_2Cl_2) (*Z*)-**2**: δ_C = 178.2 (**C$_1$**), 154.0 (d, $^2J_{CF}$ = 13.8 Hz, **C$_6$**), 137.4 (**C$_{14}$**), 130.9 (**C$_{13}$**), 104.9 (d, $^3J_{CF}$ = 9.8 Hz, **C$_5$**), 94.9 (d, $^1J_{CF}$ = 175.9 Hz, **C$_7$**), 83.3 (**C$_9$**), 77.5 (**C$_{11}$**), 73.3 (**C$_{15}$**), 52.6 (d, $^2J_{CF}$ = 21.6 Hz, **C$_8$**), 51.8 (d, $^3J_{CF}$ = 3.9 Hz, **C$_{12}$**), 40.6 (**C$_{10}$**), 37.4 (**C$_{16}$**), 32.1 (**C$_2$**), 30.1 (**C$_{17}$**), 25.6 (**C$_3$**), 24.7 (d, $^4J_{CF}$ = 3.2 Hz, **C$_4$**), 24.5 (**C$_{18}$**), 23.0 (**C$_{19}$**), 14.2 (**C$_{20}$**).

HRMS (ESI$^+$) calculated for $C_{20}H_{31}FNaO_5$ [M+Na$^+$] 393.2048, found 393.2051.

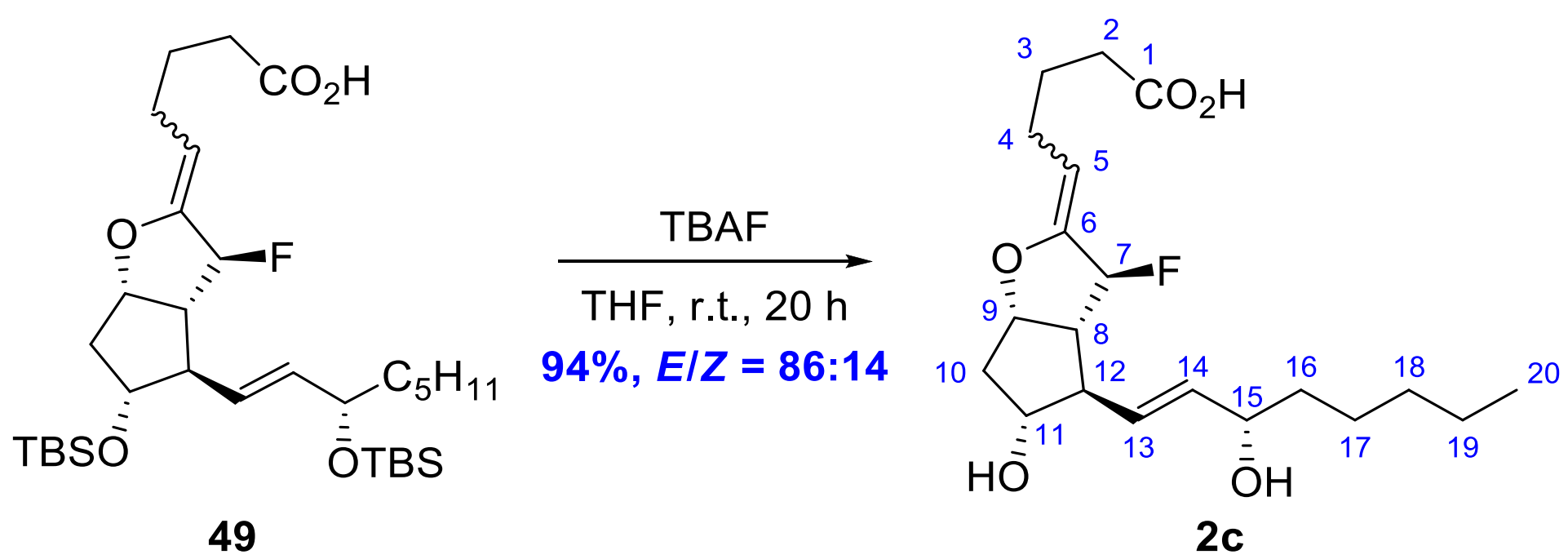

To a stirring solution of **49** (12.0 mg, 20.0 μmol, 1.0 eq., 83:17 *E*/*Z*) in THF (0.2 mL) at 0 °C, TBAF (1.0 M in THF, 60.0 μL, 60.0 mmol, 3.0 eq.) was added dropwise. The reaction mixture was warmed to r.t. and stirred for a further 20 h. The reaction was quenched by addition of H_2O (1 mL) and the aqueous phase was extracted with Et_2O (3 × 3 mL). The combined organic phases were washed with brine, dried over $MgSO_4$, filtered and concentrated *in vacuo*. The crude product was purified by flash column chromatography on silica gel (CH_2Cl_2/EtOAc/AcOH = 60:39:1 to 40:59:1) to give **2c** as a colourless oil (7.00 mg, 18.9 μmol, 94%, 86:14 *E*/*Z*).

^{1}H NMR (500 MHz, CD_2Cl_2+CD_3OD) (*E*)-**2**: δ_H = 5.62 – 5.51 (m, 1H, ***H***C14), 5.53 (dd, *J* = 15.0, 7.8 Hz, 1H, ***H***C13), 5.23 (d, *J* = 55.0 Hz, 1H, ***H***C7), 5.01 – 4 .97 (m, 1H, ***H***C9), 4.73 (td, *J* = 6.4, 2.5 Hz, 1H, ***H***C5), 4.04 – 3.98 (m, 1H, ***H***C11), 3.84 (q, *J* = 8.4 Hz, 1H, ***H***C15), 2.53 – 2.46 (m, 2H, ***H***C8+ $\boldsymbol{H_A}$C10), 2.34 – 2.21 (m, 2H, $\boldsymbol{H_2}$C2), 2.19 – 2.03 (m, 2H, $\boldsymbol{H_2}$C4), 2.03 – 1.94 (m, 1H, ***H***C12), 1.75 – 1.63 (m, 3H, $\boldsymbol{H_2}$C3+ $\boldsymbol{H_B}$C10), 1.59 – 1.53 (m, 1H, $\boldsymbol{H_A}$C16), 1.48 – 1.41 (m, 1H, $\boldsymbol{H_B}$C16), 1.33 – 1.25 (m, 6H, $\boldsymbol{H_2}$C17+$\boldsymbol{H_2}$C18+$\boldsymbol{H_2}$C19), 0.89 (t, *J* = 6.7 Hz, 3H, $\boldsymbol{H_3}$C20).

^{19}F NMR (470 MHz, CD_2Cl_2); (*E*)-**2**: δ_F = -165.61 (dd, $^2J_{HF}$ = 54.9 Hz, $^3J_{HF}$ = 18.3 Hz); (*Z*)-**2**: δ_F = -160.03 (ddd, $^2J_{HF}$ = 54.9 Hz, $^3J_{HF}$ = 18.3 Hz, $^4J_{HF}$ = 8.4 Hz).

^{13}C NMR (125 MHz, CD_2Cl_2) (*E*)-**2**: δ_C = 178.3 (**C1**), 154.4 (d, $^2J_{CF}$ = 13.9 Hz, **C6**), 137.2 (**C14**), 130.5 (**C13**), 104.6 (d, $^3J_{CF}$ = 9.3 Hz, **C5**), 91.3 (d, $^1J_{CF}$ = 175.5 Hz, **C7**), 82.5 (**C9**), 77.5 (**C11**), 73.3 (**C15**), 52.8 (d, $^2J_{CF}$ = 20.9 Hz, **C8**), 51.9 (d, $^3J_{CF}$ = 4.1 Hz, **C12**), 40.6 (**C10**), 37.4 (**C16**), 33.7 (**C2**), 32.1 (**C17**), 30.1 (**C3**), 26.9 (**C4**), 25.6 (**C18**), 23.0 (**C19**), 14.2 (**C20**).

HRMS (ESI$^+$) calculated for $C_{20}H_{31}FNaO_5$ [M+Na$^+$] 393.2048, found 393.2050.

(3*S*,3a*R*,4*R*,5*R*,6a*S*)-5-(*tert*-Butyldimethylsilyloxy)-4-((3*S*,4*S*,*E*)-3-(*tert*-butyldimethylsilyloxy)-4-methyloct-1-en-6-ynyl)-3-fluorohexahydro-2*H*-cyclopenta[*b*]furan-2-one (50)

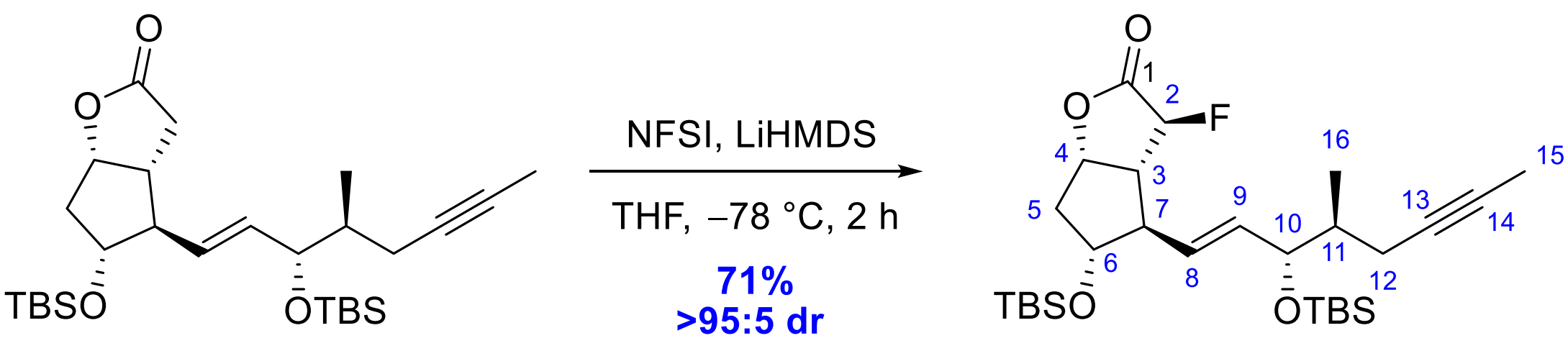

28 **50**

To a solution of **28** (208 mg, 0.42 mmol, 1.0 eq..) and NFSI (165 mg, 0.52 mmol, 1.25 eq.) in THF (2.1 mL) at –78 °C, LiHMDS (1.0 M in THF, 0.48 mL, 0.48 mmol, 1.15 eq.) was added

dropwise. The reaction mixture was stirred at –78 °C for 2 h before it was warmed to r.t., quenched by addition of saturated $NaHCO_3$ solution (4 mL) and the aqueous phase was extracted with Et_2O (3 × 4 mL). The organic phases were combined, washed with brine, dried over $MgSO_4$, filtered and concentrated *in vacuo*. The crude product was purified by flash column chromatography on silica gel (Hexanes/Et_2O = 100:0 to 90:10) to give pure **50** as a white foam (157 mg, 0.30 mmol, 71%, >95:5 dr).

Mp 68–71 °C

$\boldsymbol{R_f}$ = 0.38 (Hexanes/Et_2O = 90:10)

IR (thin film) $\boldsymbol{\nu_{max}}$/cm^{-1}: 2954, 2928, 2857, 1788, 1471, 1253, 1191, 1084, 1062, 1004, 938, 888, 833, 814, 775.

^{1}H NMR (400 MHz, $CDCl_3$) δ_H = 5.47 (dd, J = 15.8, 6.4 Hz, 1H, $\boldsymbol{H}C_9$), 5.37 (dd, J = 15.6, 7.1 Hz, 1H, $\boldsymbol{H}C_8$), 5.19 – 5.16 (m, 1H, $\boldsymbol{H}C_4$), 5.09 (dd, J = 52.0, 2.5 Hz, 1H, $\boldsymbol{H}C_2$), 4.08 (s, 1H, $\boldsymbol{H}C_6$), 3.96 (t, J = 6.3 Hz, 1H, $\boldsymbol{H}C_{10}$), 2.94 (ddt, J = 32.8, 7.5, 2.4 Hz, 1H, $\boldsymbol{H}C_3$), 2.85 (d, J = 7.0 Hz, 1H, $\boldsymbol{H}C_7$), 2.18 – 2.08 (m, 2H, $\boldsymbol{H_A}C_5$+ $\boldsymbol{H_A}C_{12}$), 2.07 – 2.00 (m, 2H, $\boldsymbol{H_B}C_5$+$\boldsymbol{H_B}C_{12}$), 1.77 (t, J = 2.5 Hz, 3H, $\boldsymbol{H_3}C_{15}$), 1.67 (dt, J = 12.8, 6.6 Hz, 1H, $\boldsymbol{H}C_{11}$), 0.89 – 0.87 (m, 12H, SiC(C$\boldsymbol{H_3}$)$_3$+$\boldsymbol{H_3}C_{16}$), 0.85 (s, 9H, SiC(C$\boldsymbol{H_3}$)$_3$), 0.06 (s, 3H, SiC$\boldsymbol{H_3}$), 0.05 (s, 3H, SiC$\boldsymbol{H_3}$), 0.04 (s, 3H, SiC$\boldsymbol{H_3}$), -0.02 (s, 3H, SiC$\boldsymbol{H_3}$).

^{19}F NMR (377 MHz, $CDCl_3$) δ_F = -176.57 (dd, $^2J_{HF}$ = 51.8 Hz, $^3J_{HF}$ = 32.8 Hz).

^{13}C NMR (100 MHz, $CDCl_3$) δ_C = 172.0 (d, $^2J_{CF}$ = 21.3 Hz, **C$_1$**), 134.1 (**C$_9$**), 129.4 (**C$_8$**), 92.1 (d, $^1J_{CF}$ = 183.5 Hz, **C$_2$**), 83.2 (d, $^3J_{CF}$ = 1.5 Hz, 1H, **C$_4$**), 78.6 (**C$_{14}$**), 77.6 (**C$_6$**), 76.8 (**C$_{13}$**), 75.9 (**C$_{10}$**), 55.3 (d, $^3J_{CF}$ = 4.9 Hz, **C$_7$**), 50.2 (d, $^2J_{CF}$ = 20.3 Hz, **C$_3$**), 39.8 (**C$_5$**), 39.5 (**C$_{11}$**), 26.0 (SiC($\boldsymbol{C}H_3$)$_3$), 25.8 (SiC($\boldsymbol{C}H_3$)$_3$), 22.1 (**C$_{12}$**), 18.3 (Si$\boldsymbol{C}$(CH_3)$_3$), 18.2 (Si$\boldsymbol{C}$(CH_3)$_3$), 15.6 (**C$_{16}$**), 3.6 (**C$_{15}$**), -4.0 (Si$\boldsymbol{C}H_3$), -4.7 (Si$\boldsymbol{C}H_3$), -4.8 (Si$\boldsymbol{C}H_3$), -5.0 (Si$\boldsymbol{C}H_3$).

HRMS (ESI$^+$) calculated for $C_{28}H_{49}FNaO_4Si_2$ [M+Na$^+$] 547.3046, found 547.3049.

$[\alpha]_D^{20}$ = –20.0 (c 1.00, $CHCl_3$)

5-((3*S*,3a*R*,4*R*,5*R*,6a*S*)-5-(*tert*-Butyldimethylsilyloxy)-4-((3*S*,4*S*,*E*)-3-(*tert*-butyldimethylsilyloxy)-4-methyloct-1-en-6-ynyl)-3-fluorohexahydro-2*H*-cyclopenta[*b*]furan-2-ylidene)pentanoic acid (51)

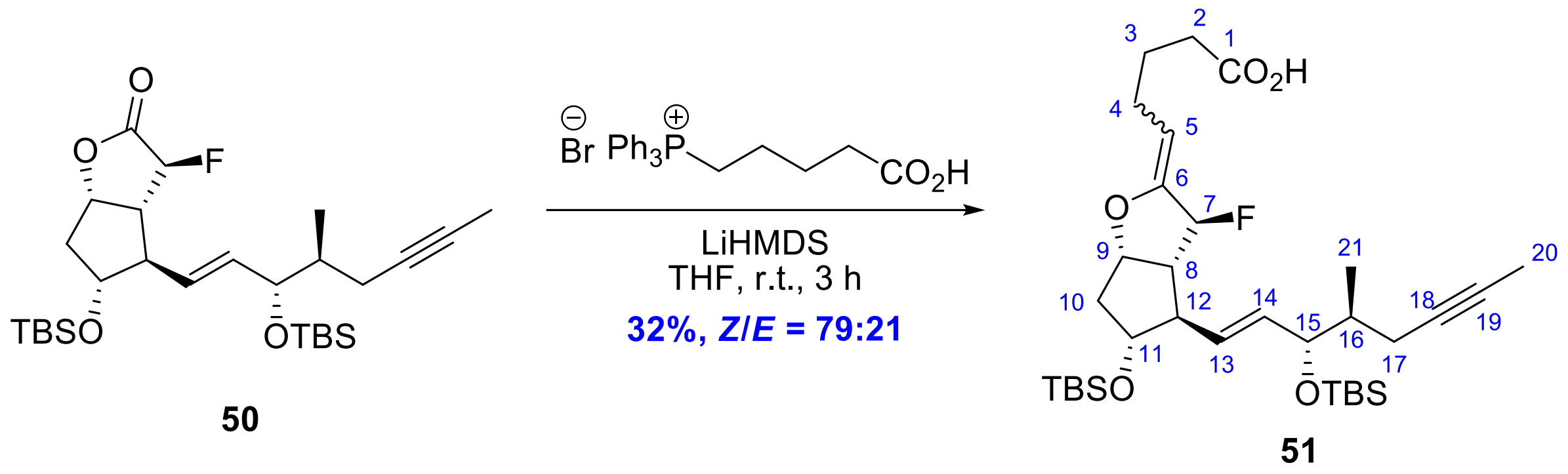

LiHMDS (1.0 M in THF, 0.26 mL, 0.26 mmol, 3.2 eq.) was added to a stirring suspension of (4-carboxybutyl)triphenylphosphonium bromide (53.2 mg, 0.12 mmol, 1.5 eq.) in THF (0.4 mL). The resulting orange suspension was stirred at r.t. for 1 h. **50** (42.0 mg, 80.0 μmol, 1.0 eq.) was added as a solution in THF (0.4 mL) to the orange suspension and the reaction was stirred at r.t. for 3 h. The reaction mixture was quenched by addition of water (8 mL) and extracted with EtOAc (3 × 8 mL). The combined organic phases were washed with water (8 mL), treated with aqueous HCl (0.1 M, 8 mL), washed with brine, dried over $MgSO_4$, filtered and concentrated *in vacuo*. The crude product was purified by flash column chromatography on silica gel (Hexanes/EtOAc = 95:5; then Hexanes/EtOAc/AcOH = 95:4:1) to give **51** as a colourless oil (15.6 mg, 25.6 μmol, 32%, 79:21 *Z*/*E*).

$\boldsymbol{R_f}$ = 0.38 (Hexanes/EtOAc/AcOH = 80:19:1).

IR (thin film) $\boldsymbol{\nu_{max}}$/cm^{-1}: 2955, 2928, 2856, 1709, 1471, 1452, 1315, 1293, 1251, 1123, 1052, 872, 835, 775.

^{1}H NMR (500 MHz, CD_2Cl_2) (*Z*)-**51**: δ_H = 5.55 – 5.54 (m, 2H, ***H***C13+***H***C14), 4.95 (d, *J* = 56.1 Hz, 1H, ***H***C7), 4.81 (td, *J* = 6.7, 2.8 Hz, 1H, ***H***C5), 4.69 (q, *J* = 7.3 Hz, 1H, ***H***C9), 4.06 – 4.04 (m, 1H, ***H***C15), 3.92 (q, *J* = 7.8 Hz, 1H, ***H***C11), 2.49 – 2.40 (m, 2H, ***H***C8+***H***C12), 2.39 – 2.36 (m, 2H, $\boldsymbol{H_2}$C2), 2.22 – 2.19 (m, 1H, $\boldsymbol{H_A}$C10), 2.18 – 2.15 (m, 2H, $\boldsymbol{H_2}$C17), 2.13 – 2.04 (m, 3H, $\boldsymbol{H_2}$C4+$\boldsymbol{H_B}$C10), 1.76 (t, *J* = 2.5 Hz, 3H, $\boldsymbol{H_3}$C21), 1.74 – 1.69 (m, 3H, $\boldsymbol{H_2}$C3+***H***C16), 0.91 – 0.90 (m, 12H, SiC(C$\boldsymbol{H_3}$)3+ $\boldsymbol{H_3}$C20), 0.87 (s, 9H, SiC(C$\boldsymbol{H_3}$)3), 0.07 (s, 3H, SiC$\boldsymbol{H_3}$), 0.04 (s, 3H, SiC$\boldsymbol{H_3}$), 0.04 (s, 3H, SiC$\boldsymbol{H_3}$), 0.03 (s, 3H, SiC$\boldsymbol{H_3}$).

^{19}F NMR (470 MHz, CD_2Cl_2) (*Z*)-**51**: δ_F = -158.95 (ddd, $^2J_{HF}$ = 56.1, $^3J_{HF}$ = 21.0, $^4J_{HF}$ = 6.9 Hz); (*E*)-**51**: δ_F = -164.52 (ddd, $^2J_{HF}$ = 55.2, $^3J_{HF}$ = 22.0, $^4J_{HF}$ = 5.2 Hz).

^{13}C NMR (125 MHz, CD_2Cl_2) (*Z*)-**51**: δ_C = 178.6 (**C1**), 154.2 (d, $^2J_{CF}$ = 14.7 Hz, **C6**), 134.2 (**C14**), 130.5 (**C13**), 104.0 (d, $^3J_{CF}$ = 10.5 Hz, **C5**), 95.4 (d, $^1J_{CF}$ = 175.6 Hz, **C7**), 83.3(**C19**), 78.2 (d, $^3J_{CF}$ = 2.6 Hz, **C9**), 78.1 (**C11**), 76.8 (**C18**), 76.1 (**C15**), 52.2 (d, $^2J_{CF}$ = 21.5 Hz, **C8**), 51.5 (d, $^3J_{CF}$ = 6.5 Hz, **C12**), 42.0 (**C10**), 40.2 (**C16**), 33.6 (**C2**), 26.1 (SiC(*C*H3)3), 26.0 (SiC(*C*H3)3), 25.0 (d, $^5J_{CF}$ = 3.8 Hz, **C3**), 24.8 (d, $^4J_{CF}$ = 5.2 Hz, **C4**), 22.4 (**C17**), 18.5 (Si*C*(CH3)3), 18.3 (Si*C*(CH3)3), 15.6 (**C21**), 3.5 (**C20**), -3.9 (Si*C*H3), -4.4 (Si*C*H3), -4.6 (Si*C*H3), -4.8 (Si*C*H3).
HRMS (ESI^+) calculated for $C_{33}H_{57}FNaO_5Si_2$ [$M+Na^+$] 631.3621, found 631.3616.

5-((3*S*,3a*R*,4*R*,5*R*,6a*S*)-3-Fluoro-5-hydroxy-4-((3*S*,4*S*,*E*)-3-hydroxy-4-methyloct-1-en-6-ynyl)hexahydro-2*H*-cyclopenta[*b*]furan-2-ylidene)pentanoic acid (3)

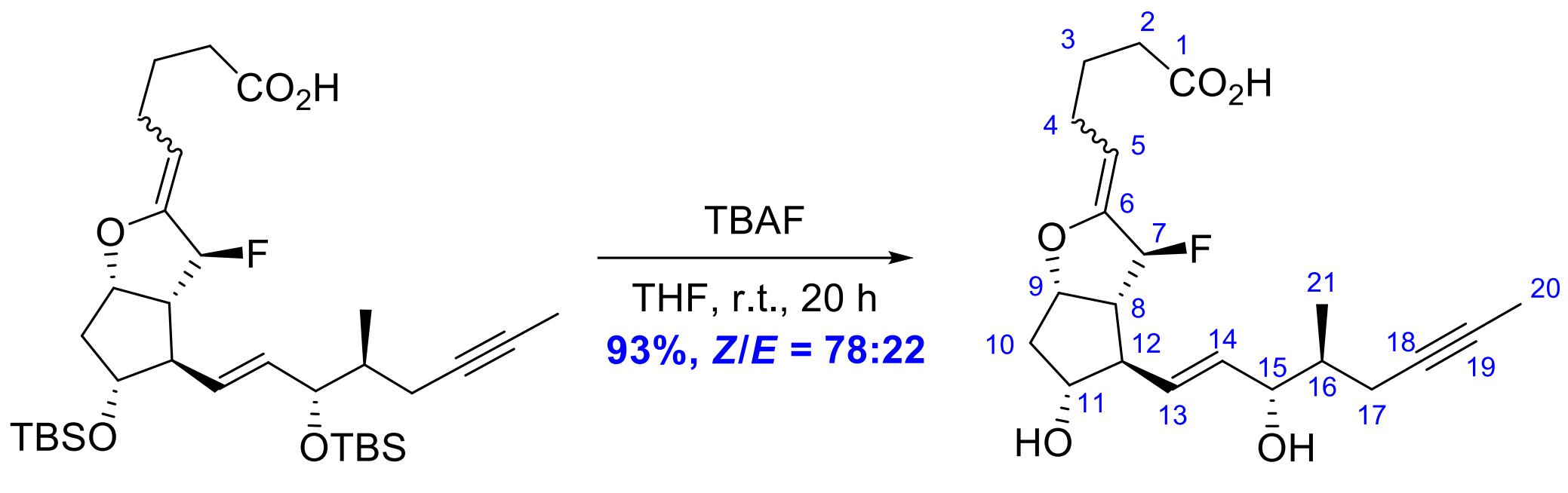

51 **3**

To a stirring solution of **51** (12.0 mg, 19.7 µmol, 1.0 eq.) in THF (0.2 mL) at 0 °C, TBAF (1.0 M in THF, 59.1 µL, 59.1 mmol, 3.0 eq.) was added dropwise. The reaction mixture was warmed to r.t. and stirred for a further 20 h. The reaction was quenched by addition of H2O (1 mL) and the aqueous phase was extracted with Et_2O (3 × 3 mL). The combined organic phases were washed with brine, dried over $MgSO_4$, filtered and concentrated *in vacuo*. The crude product was purified by flash column chromatography on silica gel (CH_2Cl_2/EtOAc/AcOH = 60:39:1 to 40:59:1) to give **3** as a colourless oil (7.00 mg, 18.4 µmol, 93%, 78:22 *Z*/*E*).

R_f = 0.21 (CH_2Cl_2/EtOAc/AcOH = 40:59:1).
IR (thin film) ν_{max}/cm^{-1}: 3373, 2958, 2922, 2856, 1708, 1555, 1456, 1376, 1295, 1236, 1137, 1096, 1004, 969, 915, 800.
^{1}H NMR (500 MHz, CD_2Cl_2+CD_3OD) (*Z*)-**3**: δ_H = 5.58 – 5.54 (m, 2H, ***H***C13+***H***C14), 4.89 (d, *J* = 55.6 Hz, 1H, ***H***C7), 4.79 (td, *J* = 6.4, 1.2 Hz, 1H, ***H***C5), 4.75 – 4.70 (m, 1H, ***H***C9), 3.91 – 3.88 (m, 1H, ***H***C11), 3.87 – 3.80 (m, 1H, ***H***C15), 2.57 – 2.42 (m, 2H, ***H***C8+***H***C12), 2.27 – 2.20 (m, 4H, ***H2***C2+ ***HA***C10+ ***HA***C17), 2.16 – 2.11 (m, 2H, ***HA***C4+***HB***C17), 2.08 – 2.04 (m, 1H, ***HB***C4), 2.00 – 1.92 (m, 1H, ***HB***C10), 1.76 (t, *J* = 2.5 Hz, 3H, ***H3***C20), 1.71 – 1.64 (m, 3H, ***H2***C3+***H***C16), 0.93 (d, *J* = 6.8 Hz, 3H, ***H3***C21).

^{19}F NMR (470 MHz, CD_2Cl_2) (*Z*)-**3**: δ_F = -160.21 (dd, $^2J_{HF}$ = 52.0 Hz, $^4J_{HF}$ = 6.0 Hz); (*E*)-**3**: δ_F = -165.44 (ddd, $^2J_{HF}$ = 54.0 Hz, $^3J_{HF}$ = 16.9 Hz, $^4J_{HF}$ = 9.0 Hz).

^{13}C NMR (125 MHz, CD_2Cl_2) (*Z*)-**3**: δ_C = 178.9 (**C$_1$**), 153.9 (d, $^2J_{CF}$ = 14.4 Hz, **C$_6$**), 135.3 (**C$_{14}$**), 132.3 (**C$_{13}$**), 105.1 (d, $^3J_{CF}$ = 9.9 Hz, **C$_5$**), 94.9 (d, $^1J_{CF}$ = 175.9 Hz, **C$_7$**), 83.3 (**C$_{19}$**), 77.6 (**C$_9$**), 77.4 (**C$_{11}$**), 77.3 (**C$_{18}$**), 76.5 (**C$_{15}$**), 52.6 (d, $^2J_{CF}$ = 21.7 Hz, **C$_8$**), 51.9 (d, $^3J_{CF}$ = 4.3 Hz, **C$_{12}$**), 40.6 (**C$_{10}$**), 38.5 (**C$_{16}$**), 32.3 (**C$_2$**), 30.1 (**C$_3$**), 24.8 (**C$_4$**), 22.5 (**C$_{17}$**), 15.9 (**C$_{21}$**), 3.6 (**C$_{20}$**).

HRMS (ESI$^+$) calculated for $C_{21}H_{28}FO_5$ [M-H$^-$] 379.1926, found 379.1909.

Synthesis for alkyne targets 4, 5, 8 and 9

Synthesis of precursors 32 and 36 for alkyne targets

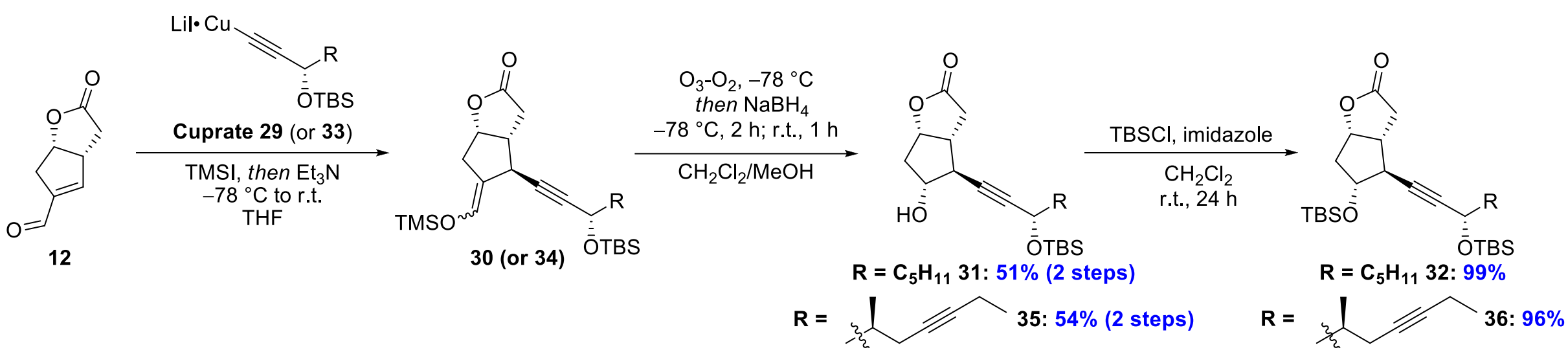

(3a*R*,4*S*,5*R*,6a*S*)-4-((*S*)-3-(*tert*-Butyldimethylsilyloxy)oct-1-ynyl)-5-hydroxyhexahydro-2*H*-cyclopenta[*b*]furan-2-one (31)

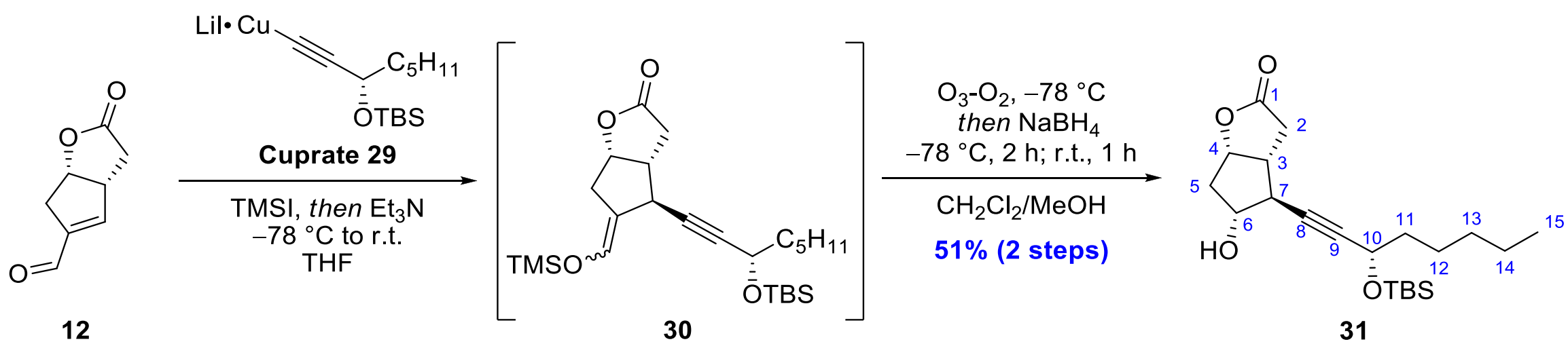

To a solution of alcohol **S8** (2.53 g, 10.5 mmol, 1.5 eq.) in anhydrous THF (27.5 mL) stirring at –10 °C, *n*-BuLi (1.6 M in hexanes, 6.60 mL, 10.5 mmol, 1.5 eq.) was added dropwise and the resulting pale yellow solution was stirred at –10 °C for 30 min whereupon CuI·0.75DMS (2.74 g, 11.6 mmol, 1.65 eq.) was added in one portion and the mixture was stirred for an additional 1 h. The solution was cooled to −78 °C and TMSI (1.50 mL, 2.10 g, 10.5 mmol, 1.5 eq.) was added. After 5 min, enal **12** (1.07 g, 7.00 mmol, 1.0 eq.) in anhydrous THF (10.5 mL) was added dropwise to the solution of cuprate **29**. After the addition was completed, the mixture was stirred for an additional 1 h at –78 °C before Et_3N (4.4 mL, 3.19 g, 31.5 mmol, 4.5 eq.) was added. The reaction mixture was warned to room temperature to stir for 1 h before diluting by addition of Et_2O (30 mL). The reaction mixture was quenched by addition of saturated

aqueous $NaHCO_3$ solution (30 mL). The aqueous phase was extracted with Et_2O (3 × 30 mL), and the combined organic phases were washed further with brine, dried over $MgSO_4$, filtered and concentrated in *vacuo* to give crude **30** as a brown oil, which was used directly in the next step.

In a round-bottom flask, a solution of crude **30** in CH_2Cl_2/MeOH (56.0 mL, 3:1 *v/v*) was cooled to –78 °C and a stream of O_3-O_2(g) was bubbled through the reaction solution. The reaction progress was carefully monitored by TLC (CH_2Cl_2) until only a small amount of **30** remained (~5.0 min). The reaction mixture was then purged of O_3(g) by bubbling N_2(g) through the solution for 30 min followed by the portion-wise addition of freshly ground $NaBH_4$ (794 mg, 21.0 mmol, 3.0 eq.). The reaction was stirred at –78 °C for 2 h before the dry ice/acetone bath was removed and the reaction was allowed to warm to r.t., at which point it was stirred for a further 1 h. The reaction mixture was then poured over saturated brine solution (28 mL) and extracted with EtOAc (3 × 28 mL). The combined organic phases were washed with brine, dried over $MgSO_4$, filtered and concentrated *in vacuo*. The crude product was purified by flash column chromatography on silica gel (CH_2Cl_2/EtOAc = 95:5 to 90:10) to give pure **31** as a pale yellow oil (1.36 g, 3.57 mmol, 51%).

$\boldsymbol{R_f}$ = 0.21 (CH_2Cl_2/EtOAc = 90:10)

IR (thin film) $\boldsymbol{\nu_{max}}$/cm^{-1}: 2953, 2929, 2857, 1732, 1471, 1362, 1329, 1254, 1190, 1088, 1001, 949, 888, 833, 775.

^{1}H NMR (400 MHz, $CDCl_3$) δ_H = 5.06 (t, J = 6.6 Hz, 1H, $\boldsymbol{H}C_4$), 4.31 – 4.24 (m, 2H, $\boldsymbol{H}C_6$+ $\boldsymbol{H}C_{10}$), 3.03 – 2.97 (m, 2H, $\boldsymbol{H_A}C_2$+$\boldsymbol{H}C_7$), 2.83 – 2.75 (m, 2H, $\boldsymbol{H}C_3$+O$\boldsymbol{H}$), 2.56 (dd, J = 18.6, 3.3 Hz, 1H, $\boldsymbol{H_B}C_2$), 2.27 (ddd, J = 15.1, 6.5, 4.5 Hz, 1H, $\boldsymbol{H_A}C_5$), 2.13 (d, J = 15.2 Hz, 1H, $\boldsymbol{H_B}C_5$), 1.61 – 1.49 (m, 2H, $\boldsymbol{H_2}C_{11}$), 1.39 – 1.20 (m, 6H, $\boldsymbol{H_2}C_{12}$+$\boldsymbol{H_2}C_{13}$+$\boldsymbol{H_2}C_{14}$), 0.86 – 0.83 (m, 12H, SiC(C$\boldsymbol{H_3}$)$_3$+$\boldsymbol{H_3}C_{15}$), 0.05 (s, 3H, SiC$\boldsymbol{H_3}$), 0.04 (s, 3H, SiC$\boldsymbol{H_3}$).

^{13}C NMR (101 MHz, $CDCl_3$) δ_C = 177.9 (**C_1**), 85.7(**C_8**), 84.7 (**C_4**), 83.2 (**C_9**), 78.0 (**C_6**), 63.1 (**C_{10}**), 45.9 (**C_3**), 44.7 (**C_7**), 40.1 (**C_5**), 38.7 (**C_{11}**), 35.9 (**C_2**), 31.4 (**C_{12}**), 25.8 (SiC($\boldsymbol{C}H_3$)$_3$), 25.0 (**C_{13}**), 22.6 (**C_{14}**), 18.3 (Si$\boldsymbol{C}$(CH_3)$_3$), 14.0 (**C_{15}**), -4.5 (Si$\boldsymbol{C}H_3$), -4.9 (Si$\boldsymbol{C}H_3$).

HRMS (ESI^+) calculated for $C_{21}H_{36}NaO_4Si$ [M+Na^+] 403.2275, found 403.2266.

$[\alpha]_D^{22}$ = –10.3 (*c* 1.00, $CHCl_3$)

(3a*R*,4*S*,5*R*,6a*S*)-5-(*tert*-Butyldimethylsilyloxy)-4-((*S*)-3-(*tert*-Butyldimethylsilyloxy)oct-1-ynyl)hexahydro-2*H*-cyclopenta[*b*]furan-2-one (32)

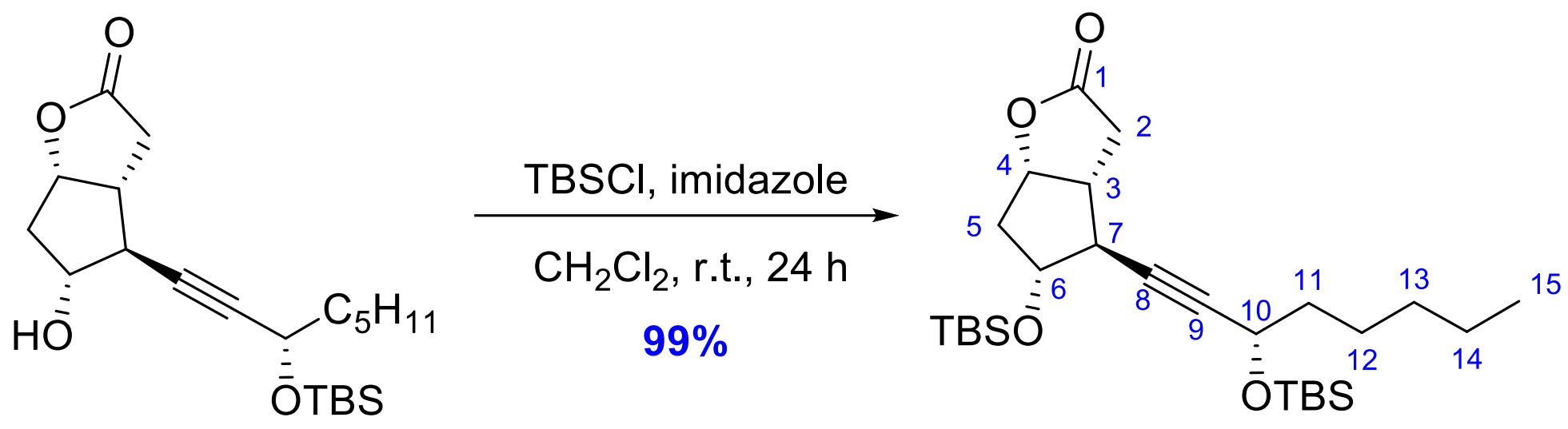

To a solution of **31** (1.26 g, 3.31 mmol, 1.0 eq.) in CH_2Cl_2 (11.0 ml) was added imidazole (1.50 g, 9.93 mmol, 3.0 eq.) and TBSCl (1.08 g, 15.9 mmol, 4.8 eq.) at 0 °C. The reaction mixture was stirred at r.t. for 24 h before the mixture was poured over H_2O (22 mL) and the aqueous phase was extracted with CH_2Cl_2 (3 × 22 mL). The organic phases were combined, washed with brine, dried over $MgSO_4$, filtered and concentrated *in vacuo*. The crude product was purified by flash column chromatography on silica gel (Hexanes/EtOAc = 95:5 to 90:10) to give pure **32** as a white, amorphous solid (1.76 g, 3.27 mmol, 99%).

Mp 35–39 °C

$\boldsymbol{R_f}$ = 0.33 (Hexanes/EtOAc = 90:10)

IR (thin film) $\boldsymbol{\nu_{max}}$/cm^{-1}: 2953, 2929, 2857, 1776, 1471, 1351, 1252, 1171, 1099, 1032, 1005, 897, 833, 775.

^{1}H NMR (400 MHz, $CDCl_3$) δ_H = 5.03 (t, *J* = 6.8 Hz, 1H, ***H***C4), 4.26 (dd, *J* = 6.4, 1.8 Hz, 1H, ***H***C6), 4.24 – 4.22 (m, 1H, ***H***C10), 2.99 (ddt, *J* = 11.1, 7.2, 3.5 Hz, 1H, ***H***C7), 2.77 (dd, *J* = 18.3, 11.3 Hz, 1H, $\boldsymbol{H_A}$C2), 2.72 –2.69 (m, 1H, ***H***C3), 2.50 (dd, *J* = 18.4, 3.7 Hz, 1H, $\boldsymbol{H_B}$C2), 2.27 – 2.20 (m, 1H, $\boldsymbol{H_A}$C5), 2.03 (d, *J* = 14.8 Hz, 1H, $\boldsymbol{H_B}$C5), 1.59 – 1.52 (m, 2H, $\boldsymbol{H_2}$C11), 1.36 – 1.21 (m, 6H, $\boldsymbol{H_2}$C12+$\boldsymbol{H_2}$C13+$\boldsymbol{H_2}$C14), 0.87 – 0.83 (m, 12H, SiC(C$\boldsymbol{H_3}$)$_3$+$\boldsymbol{H_3}$C15), 0.83 (s, 9H, SiC(C$\boldsymbol{H_3}$)$_3$), 0.05 (s, 3H, Si***C***H$_3$), 0.04 (s, 6H, Si***C***H$_3$+Si***C***H$_3$), 0.04 (s, 3H, Si***C***H$_3$).

^{13}C NMR (101 MHz, $CDCl_3$) δ_C = 176.7 (**C1**), 85.7 (**C8**), 84.2 (**C4**), 83.3 (**C9**), 78.8 (**C6**), 63.0 (**C10**), 46.6 (**C3**), 44.7 (**C7**), 40.8 (**C5**), 38.7 (**C11**), 35.8 (**C2**), 31.4 (**C12**), 25.8 (SiC(***C***H$_3$)$_3$), 25.7 (SiC(***C***H$_3$)$_3$), 25.0 (**C13**), 22.6 (**C14**), 18.3 (Si***C***(CH$_3$)$_3$), 18.0 (Si***C***(CH$_3$)$_3$), 14.0 (**C15**), -4.5 (Si***C***H$_3$), -4.9 (Si***C***H$_3$), -4.9 (Si***C***H$_3$), -5.0 (Si***C***H$_3$).

HRMS (ESI$^+$) calculated for $C_{27}H_{50}NaO_4Si_2$ [M+Na$^+$] 517.3140, found 517.3138.

[*α*]$_D^{24}$ = –34.7 (*c* 1.00, $CHCl_3$)

(3a*R*,4*S*,5*R*,6a*S*)-4-((3*S*,4*S*)-3-(*tert*-Butyldimethylsilyloxy)-4-methylnona-1,6-diynyl)-5-hydroxyhexahydro-2*H*-cyclopenta[*b*]furan-2-one (35)

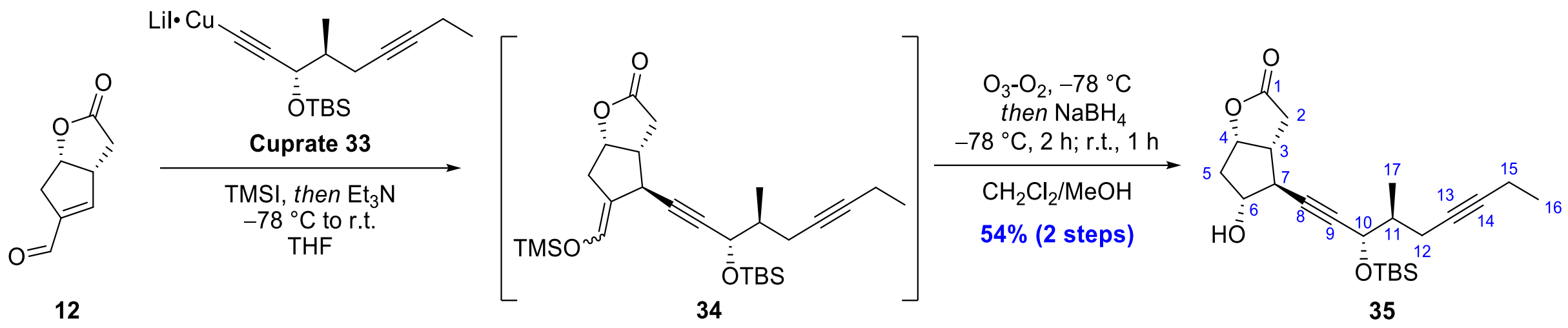

To a solution of alcohol **20** (1.19 g, 4.50 mmol, 1.5 eq.) in anhydrous THF (11.7 mL) stirring at –10 °C, *n*-BuLi (1.6 M in hexanes, 2.81 mL, 4.50 mmol, 1.5 eq.) was added dropwise and the resulting pale yellow solution was stirred at –10 °C for 30 min whereupon CuI·0.75DMS (1.17 g, 4.95 mmol, 1.65 eq.) was added in one portion and the mixture was stirred for an additional 1 h. The solution was cooled to −78 °C and TMSI (0.85 mL, 1.20 g, 6.00 mmol, 2.0 eq.) was added. After 5 min, enal **12** (456 mg, 3.00 mmol, 1.0 eq.) in anhydrous THF (4.5 mL) was added dropwise to the solution of cuprate **33**. After the addition was completed, the mixture was stirred for an additional 1 h at –78 °C before Et_3N (1.88 mL, 1.37 g, 13.5 mmol, 4.5 eq.) was added. The reaction mixture was warned to room temperature to stir for 1 h before diluting by addition of Et_2O (10 mL). The reaction mixture was quenched by addition of saturated aqueous $NaHCO_3$ solution (10 mL). The aqueous phase was extracted with Et_2O (3 × 10 mL), and the combined organic phases were washed further with brine, dried over $MgSO_4$, filtered and concentrated in *vacuo* to give crude **34** as a brown oil, which was used directly in the next step.

In a round-bottom flask, a solution of crude **34** in CH_2Cl_2/MeOH (24.0 mL, 3:1 *v/v*) was cooled to –78 °C and a stream of O_3-O_2(g) was bubbled through the reaction solution. The reaction progress was carefully monitored by TLC (CH_2Cl_2) until only a small amount of **34** remained (~2.0 min). The reaction mixture was then purged of O_3(g) by bubbling N_2(g) through the solution for 20 min followed by the portion-wise addition of freshly ground $NaBH_4$ (341 mg, 9.00 mmol, 3.0 eq.). The reaction was stirred at –78 °C for 2 h before the dry ice/acetone bath was removed and the reaction was allowed to warm to r.t., at which point it was stirred for a further 1 h. The reaction mixture was then poured over saturated brine solution (12 mL) and extracted with EtOAc (3 × 12 mL). The combined organic phases were washed with brine, dried over $MgSO_4$, filtered and concentrated *in vacuo*. The crude product was purified by flash column chromatography on silica gel (CH_2Cl_2/EtOAc = 95:5 to 90:10) to give pure **35** as a colourless oil (656 mg, 1.62 mmol, 54%).

***R*f** = 0.33 (CH_2Cl_2/EtOAc = 90:10)

IR (thin film) ν_{max}/cm^{-1}: 3434, 2956, 2929, 2855, 1757, 1452, 1351, 1320, 1251, 1188, 1085, 1027, 835, 775.

^{1}H NMR (400 MHz, $CDCl_3$) δ_H = 5.08 (dd, *J* = 10.0, 3.8 Hz, 1H, ***H***C4), 4.40 – 4.28 (m, 2H, ***H***C6+ ***H***C10), 3.02 (ddd, *J* = 14.5, 7.3, 3.6 Hz, 1H, ***H***C7), 2.84 (dd, *J* = 16.6, 9.2 Hz, 1H, $\boldsymbol{H_A}$C2), 2.81 – 2.77 (m, 1H, ***H***C3), 2.60 (dd, *J* = 18.4, 3.1 Hz, 1H, $\boldsymbol{H_B}$C2), 2.38 (ddd, *J* = 15.2, 6.6, 4.9 Hz, 1H, ***H***C11), 2.20 – 2.12 (m, 4H, $\boldsymbol{H_A}$C5+***H***C7+$\boldsymbol{H_A}$C12+$\boldsymbol{H_2}$C15), 2.03 (dd, *J* = 8.5, 2.4 Hz, 1H, $\boldsymbol{H_B}$C12), 1.80 (dt, *J* = 12.7, 6.5 Hz, 1H, $\boldsymbol{H_B}$C5), 1.11 (t, *J* = 7.4 Hz, 3H, $\boldsymbol{H_3}$C16), 1.01 (d, *J* = 6.8 Hz, 3H, $\boldsymbol{H_3}$C17), 0.89 (s, 9H, SiC(C$\boldsymbol{H_3}$)3), 0.10 (s, 3H, SiC$\boldsymbol{H_3}$), 0.09 (s, 3H, SiC$\boldsymbol{H_3}$).

^{13}C NMR (101 MHz, $CDCl_3$) δ_C = 177.0 (**C1**), 84.4 (**C8**), 84.1(**C4**), 84.0 (**C14**), 83.4 (**C9**), 78.3 (**C10**), 77.3 (**C13**), 66.3 (**C6**), 46.0 (**C3**), 44.8 (**C7**), 40.5 (**C5**), 40.2 (**C11**), 35.7 (**C2**), 25.9 (SiC(***C***H3)3), 22.1 (**C12**), 18.3 (Si***C***(CH3)3), 15.3 (**C17**), 14.5 (**C16**), 12.6 (**C15**), -4.4 (Si***C***H3), -5.0 (Si***C***H3).

HRMS (ESI$^+$) calculated for $C_{23}H_{36}NaO_4Si$ [M+Na$^+$] 427.2275, found 427.2274.

$[\alpha]_D^{22}$= -2.0 (*c* 1.0, $CHCl_3$)

(3a*R*,4*S*,5*R*,6a*S*)-5-(*tert*-Butyldimethylsilyloxy)-4-((3S,4S)-3-(*tert*-butyldimethylsilyloxy)-4-methylnona-1,6-diynyl)hexahydro-2*H*-cyclopenta[*b*]furan-2-one (36)

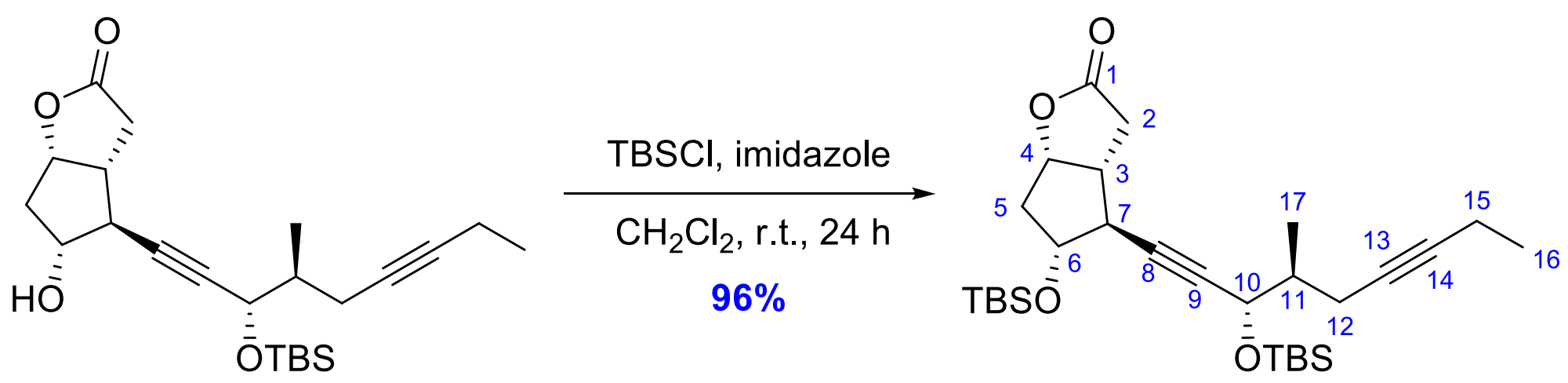

35 **36**

To a solution of **35** (1.26 g, 3.31 mmol, 1.0 eq.) in CH_2Cl_2 (11.0 ml) was added imidazole (1.50 g, 9.93 mmol, 3.0 eq.) and TBSCl (1.08 g, 15.9 mmol, 4.8 eq.) at 0 °C. The reaction mixture was stirred at r.t. for 24 h before the mixture was poured over H_2O (22.0 mL) and the aqueous phase was extracted with CH_2Cl_2 (3 × 20 mL). The organic phases were combined, washed with brine, dried over $MgSO_4$, filtered and concentrated *in vacuo*. The crude product was purified by flash column chromatography on silica gel (Hexanes/EtOAc = 95:5 to 90:10) to give pure **36** as a white, amorphous solid (1.76 g, 3.27 mmol, 96%).

Mp 40–43 °C

***R*f** = 0.20 (Hexanes/EtOAc = 90:10)

IR (thin film) ν_{max}/cm^{-1}: 2955, 2929, 2857, 1780, 1472, 1351, 1252, 1170, 1093, 1031, 1006, 836, 776.

^{1}H NMR (400 MHz, CDCl$_3$) δ_H = 5.07 (t, J = 6.9 Hz, 1H, ***H***C$_4$), 4.38 – 4.25 (m, 2H, ***H***C$_6$+***H***C$_{10}$), 3.06 – 2.99 (m, 1H, ***H***C$_7$), 2.80 (dd, J = 18.4, 11.3 Hz, 1H, ***H$_A$***C$_2$), 2.77 – 2.75 (m, 1H, ***H***C$_3$), 2.54 (dd, J = 18.4, 3.8 Hz, 1H, ***H$_B$***C$_2$), 2.29 – 2.24 (m, 1H, ***H***C$_{11}$), 2.20 – 2.06 (m, 5H, ***H$_A$***C$_5$+***H***C$_7$+***H$_2$***C$_{12}$+***H$_2$***C$_{15}$), 1.79 (dt, J = 12.7, 6.5 Hz, 1H, ***H$_B$***C$_5$), 1.10 (t, J = 7.4 Hz, 3H, ***H$_3$***C$_{16}$), 1.00 (d, J = 6.8 Hz, 3H, ***H$_3$***C$_{17}$), 0.89 (s, 9H, SiC(C***H$_3$***)$_3$), 0.86 (s, 9H, SiC(C***H$_3$***)$_3$), 0.09 (s, 3H, SiC***H$_3$***), 0.08 (s, 3H, SiC***H$_3$***), 0.07 (s, 3H, SiC***H$_3$***), 0.07 (s, 3H, SiC***H$_3$***).

^{13}C NMR (101 MHz, CDCl$_3$) δ_C = 176.8 (**C$_1$**), 84.6 (**C$_8$**), 84.4 (**C$_4$**), 84.2 (**C$_{14}$**), 83.3 (**C$_9$**), 78.9 (**C$_{10}$**), 77.3 (**C$_{13}$**), 66.3 (**C$_6$**), 46.7 (**C$_3$**), 44.8 (**C$_7$**), 40.9 (**C$_5$**), 40.2 (**C$_{11}$**), 36.0 (**C$_2$**), 25.9 (SiC(*C*H$_3$)$_3$), 25.8 (SiC(*C*H$_3$)$_3$), 22.0 (**C$_{12}$**), 18.3 (Si*C*(CH$_3$)$_3$), 18.1 (Si*C*(CH$_3$)$_3$), 15.3 (**C$_{17}$**), 14.5 (**C$_{16}$**), 12.5 (**C$_{15}$**), -4.4 (Si*C*H$_3$), -4.8 (Si*C*H$_3$), -5.0 (Si*C*H$_3$), -5.0 (Si*C*H$_3$).

HRMS (ESI$^+$) calculated for $C_{29}H_{50}NaO_4Si_2$ [M+Na$^+$] 541.3140, found 541.3132.

[α]$_D^{24}$= -22.3 (c 1.0, CHCl$_3$)

Synthesis of Difluorinated Targets 8 and 9.

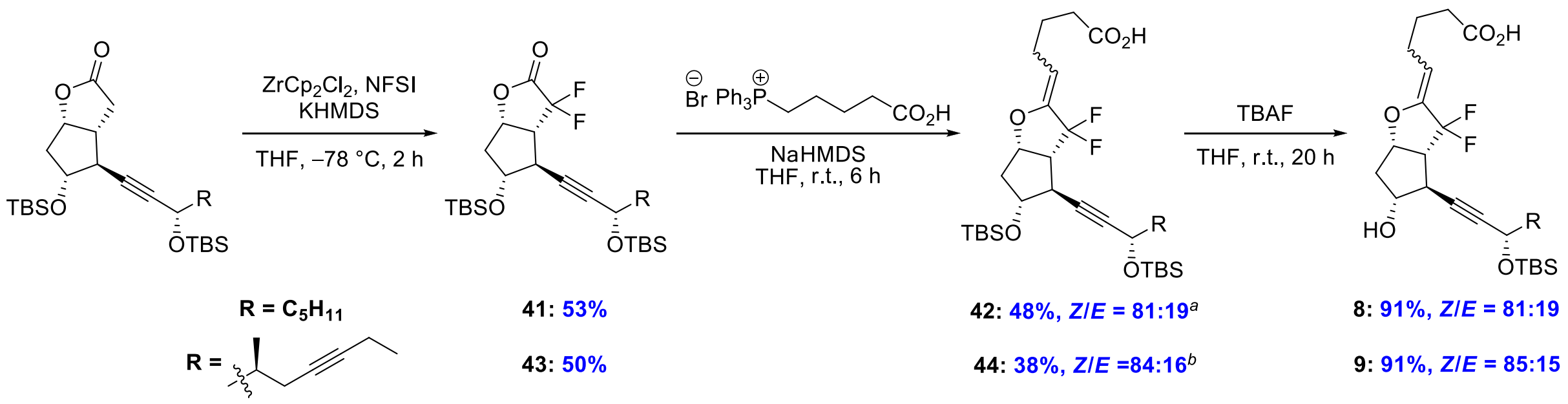

[a] The bisilyl ether (10%) was isolated together with the monosilyl ether (38%), both of which were converted into the final target **8**. [b] Reaction was conducted at r.t. for 3 h.

(3a*R*,4*S*,5*R*,6a*S*)-5-(*tert*-Butyldimethylsilyloxy)-4-((*S*)-3-(*tert*-butyldimethylsilyloxy)oct-1-ynyl)-3,3-difluorohexahydro-2*H*-cyclopenta[*b*]furan-2-one (41)

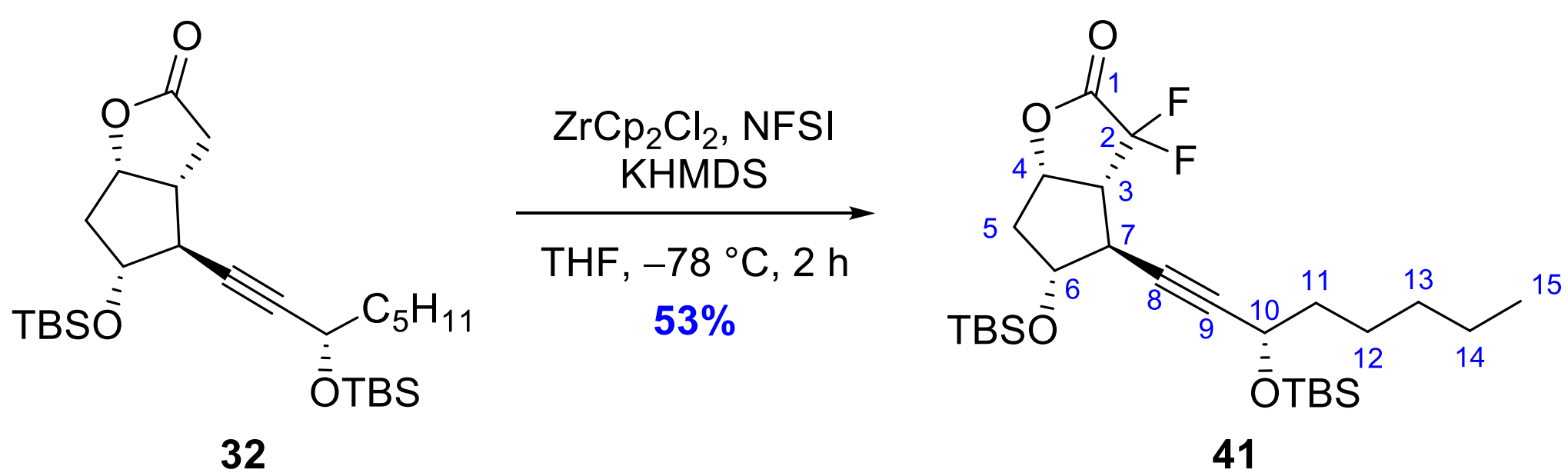

A solution of NFSI (1.89 g, 6.00 mmol, 4.0 eq.) and $ZrCp_2Cl_2$ (1.75 g, 6.00 mmol, 4.0 eq.) in THF (19.0 mL) was stirred at r.t. for 1 h and then cooled to –78 °C. A solution of **32** (742 mg, 1.50 mmol, 1.0 eq.) in THF (10.0 mL) was added followed by the drop-wise addition of KHMDS (1.0 M in THF, 7.50 mL, 7.50 mmol, 5.0 eq.) over 1 h, the reaction mixture was then stirred at –78 °C for a further hour. The reaction mixture was warmed to r.t., quenched by addition of saturated $NaHCO_3$ solution (20 mL) and the aqueous phase was subsequently extracted with Et_2O (3 × 20 mL). The organic phases were combined, washed with brine, dried over $MgSO_4$, filtered and concentrated *in vacuo*. The crude product was purified by flash column chromatography on silica gel (Hexanes/Et_2O = 100:0 to 95:5) to give pure **41** as a yellow oil (422 mg, 0.80 mmol, 53%).

$\boldsymbol{R_f}$ = 0.38 (Hexanes/Et_2O = 95:5)

IR (thin film) $\boldsymbol{\nu_{max}}$/cm^{-1}: 2953, 2929, 2858, 1818, 1471, 1362, 1338, 1240, 1088, 1029, 998, 833, 775.

^{1}H NMR (400 MHz, $CDCl_3$) δ_H = 5.21 (t, J = 6.2 Hz, 1H, $\boldsymbol{H}C_4$), 4.30 – 4.29 (m, 1H, $\boldsymbol{H}C_{10}$), 4.29 – 4.27 (m, 1H, $\boldsymbol{H}C_6$), 3.29 – 3.27 (m, 1H, $\boldsymbol{H}C_7$), 3.23 – 3.21 (m, 1H, $\boldsymbol{H}C_3$), 2.25 (ddd, J = 14.9, 5.7, 3.6 Hz, 1H, $\boldsymbol{H_A}C_5$), 2.17 (d, J = 14.9 Hz, 1H, $\boldsymbol{H_B}C_5$), 1.65 – 1.53 (m, 2H, $\boldsymbol{H_2}C_{11}$), 1.39 – 1.25 (m, 6H, $\boldsymbol{H_2}C_{12}$+$\boldsymbol{H_2}C_{13}$+$\boldsymbol{H_2}C_{14}$), 0.92 – 0.87 (m, 12H, SiC(C$\boldsymbol{H_3}$)$_3$+$\boldsymbol{H_3}C_{15}$), 0.84 (s, 9H, SiC(C$\boldsymbol{H_3}$)$_3$), 0.09 (s, 3H, Si$\boldsymbol{C}H_3$), 0.08 (s, 3H, Si$\boldsymbol{C}H_3$), 0.06 (s, 3H, Si$\boldsymbol{C}H_3$), 0.05 (s, 3H, Si$\boldsymbol{C}H_3$).

^{19}F NMR (377 MHz, $CDCl_3$) δ_F = -91.60 (dd, $^2J_{FF}$ = 281.8 Hz, $^3J_{HF}$ = 26.1 Hz), -112.55 (d, $^2J_{FF}$ = 281.8 Hz).

^{13}C NMR (100 MHz, $CDCl_3$) δ_C = 165.1 (dd, $^2J_{CF}$ =34.5 Hz, $^2J_{CF}$ = 33.2 Hz, **C$_1$**), 115.2 (dd, $^1J_{CF}$ = 265.8 Hz, $^1J_{CF}$ = 244.3 Hz, **C$_2$**), 86.5 (**C$_8$**), 81.9 (dd, $^3J_{CF}$ = 2.7 Hz, $^3J_{CF}$ = 2.6 Hz, **C$_4$**), 81.6 (**C$_9$**), 78.7 (**C$_6$**), 63.1 (**C$_{10}$**), 51.7 (dd, $^2J_{CF}$ = 24.9 Hz, $^2J_{CF}$ = 18.5 Hz, **C$_3$**), 41.3 (dd, $^3J_{CF}$ = 5.8, $^3J_{CF}$ = 4.1 Hz, **C$_7$**), 40.4 (**C$_5$**), 38.7 (**C$_{11}$**), 31.5 (**C$_{12}$**), 25.9 (SiC($\boldsymbol{C}H_3$)$_3$), 25.7 (SiC($\boldsymbol{C}H_3$)$_3$), 25.1 (**C$_{13}$**), 22.7 (**C$_{14}$**), 18.4 (Si$\boldsymbol{C}$(CH$_3$)$_3$), 18.2 (Si$\boldsymbol{C}$(CH$_3$)$_3$), 14.1 (**C$_{15}$**), -4.5 (Si$\boldsymbol{C}H_3$), -4.8 (Si$\boldsymbol{C}H_3$), -5.1 (Si$\boldsymbol{C}H_3$), -5.1 (Si$\boldsymbol{C}H_3$).

HRMS (ESI$^+$) calculated for $C_{27}H_{48}F_2NaO_4Si_2$ [M+Na$^+$] 553.2951, found 553.2945.

$[\alpha]_D^{23}$= –38.0 (*c* 1.00, $CHCl_3$)

5-((3a*R*,4*S*,5*R*,6a*S*)-5-(*tert*-Butyldimethylsilyloxy)-4-((*S*)-3-(*tert*-butyldimethylsilyloxy)oct-1-ynyl)-3,3-difluorohexahydro-2*H*-cyclopenta[*b*]furan-2-ylidene)pentanoic acid (42) and 5-((3a*R*,4*S*,5*R*,6a*S*)-4-((*S*)-3-(*tert*-butyldimethylsilyloxy)oct-1-ynyl)-3,3-difluoro-5-hydroxyhexahydro-2*H*-cyclopenta[*b*]furan-2-ylidene)pentanoic acid (42')

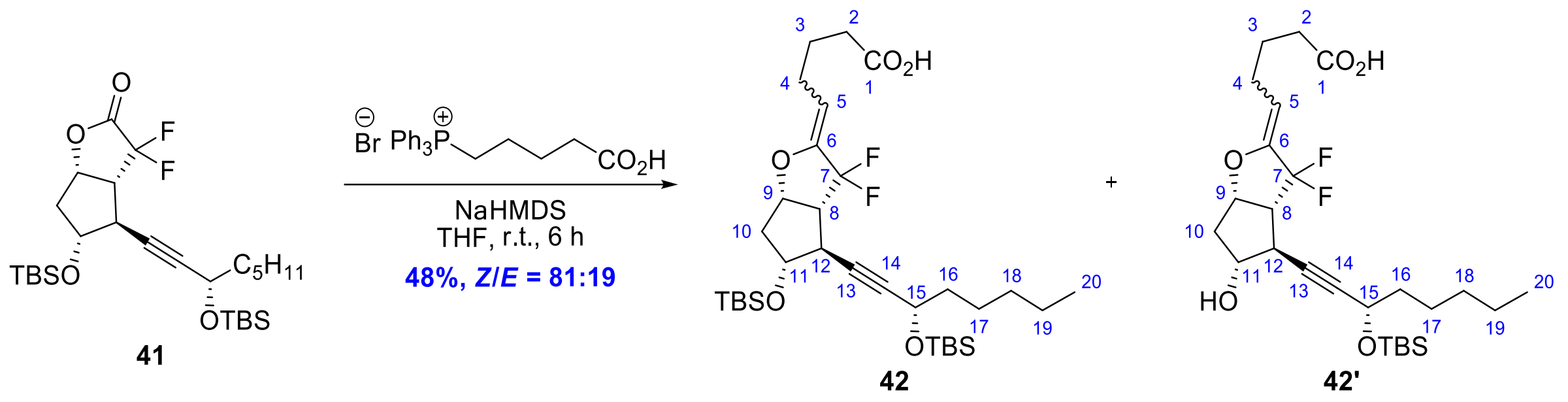

NaHMDS (1.0 M in THF, 1.00 mL, 1.00 mmol, 10.0 eq.) was added to a stirring suspension of (4-carboxybutyl)triphenylphosphonium bromide (222 mg, 0.50 mmol, 5.0 eq.) in THF (0.68 mL). The resulting orange suspension was heated to 70 °C for 1 h before cooling to r.t.. **41** (53.1 mg, 0.10 mmol, 1.0 eq.) was added as a solution in THF (0.1 mL) to the orange suspension and the reaction was stirred at r.t. for 6 h. The reaction mixture was quenched by addition of water (10 mL) and extracted with EtOAc (3 × 10 mL). The combined organic phases were washed with water (5 mL), treated with aqueous HCl (1.0 M, 10 mL), washed with brine, dried over $MgSO_4$, filtered and concentrated *in vacuo*. The crude product was purified by flash column chromatography on silica gel (Hexanes/EtOAc = 95:5 to 90:10; then Hexanes//EtOAc/AcOH = 90:9:1 to 80:19:1) to give **42** as a colourless oil (6.20 mg, 10.1 µmol, 10%, 81:19 *Z*/*E*) and **42'** as a colourless oil (18.8 mg, 37.5 µmol, 38%, 81:19 *Z*/*E*).

5-((3a*R*,4*S*,5*R*,6a*S*)-5-(*tert*-Butyldimethylsilyloxy)-4-((*S*)-3-(*tert*-butyldimethylsilyloxy)oct-1-ynyl)-3,3-difluorohexahydro-2*H*-cyclopenta[*b*]furan-2-ylidene)pentanoic acid (42):

$\boldsymbol{R_f}$ = 0. 48 (Hexanes/EtOAc/AcOH = 75:24:1)

IR (thin film) $\boldsymbol{\nu_{max}}$/cm^{-1}: 2953, 2929, 2857, 1710, 1453, 1294, 1250, 1201, 1084, 1043, 1035, 963, 836, 775.

^{1}H NMR (400 MHz, $CDCl_3$) (*Z*)-**42**: δ_H = 4.96 – 4.73 (m, 2H, ***H***C_5+***H***C_9), 4.35 – 4.31 (m, 1H, ***H***C_{15}), 4.17 (q, *J* = 6.3 Hz, 1H, ***H***C_{11}), 2.97 – 2.82 (m, 2H, ***H***C_8+***H***C_{12}), 2.40 – 2.32 (m, 3H, $\boldsymbol{H_2}C_2$+$\boldsymbol{H_A}C_{10}$), 2.18 – 210 (m, 2H, $\boldsymbol{H_2}C_4$), 1.90 – 1.84 (m, 1H, $\boldsymbol{H_B}C_{10}$), 1.78 – 1.71 (m, 2H, $\boldsymbol{H_2}C_3$), 1.67 – 1.57 (m, 2H, $\boldsymbol{H_2}C_{16}$), 1.30 – 1.26 (m, 6H, $\boldsymbol{H_2}C_{17}$+$\boldsymbol{H_2}C_{18}$+$\boldsymbol{H_2}C_{19}$), 0.91 – 0.88 (m,

12H, SiC(CH_3)$_3$+H_3C$_{20}$), 0.87 (s, 9H, SiC(CH_3)$_3$), 0.11 (s, 3H, SiCH_3), 0.09 (s, 3H, SiCH_3), 0.08 (s, 3H, SiCH_3), 0.07 (s, 3H, SiCH_3).

^{19}F NMR (377 MHz, $CDCl_3$) (*Z*)-**42**: δ_F = -82.91 (dddd, $^2J_{FF}$ = 252.7 Hz, $^3J_{HF}$ = 16.5 Hz, $^3J_{HF}$ = 9.1 Hz, $^4J_{HF}$ = 4.5 Hz), -112.45 (d, $^2J_{FF}$ = 252.7 Hz); (*E*)-**42**: δ_F = -84.27 (ddd, $^2J_{FF}$ = 260.5, $^3J_{HF}$ = 18.4, $^4J_{HF}$ = 3.2 Hz), -106.10 (d, $^2J_{FF}$ = 260.8 Hz).

^{13}C NMR (100 MHz, $CDCl_3$) (*Z*)-**42**: δ_C = 179.2 (**C$_1$**), 149.5 (dd, $^2J_{CF}$ = 30.1 Hz, $^2J_{CF}$ = 25.8 Hz, **C$_6$**), 123.6 (dd, $^1J_{CF}$ = 255.3 Hz, $^1J_{CF}$ = 240.7 Hz, **C$_7$**), 100.1 (d, $^3J_{CF}$ = 4.4 Hz, **C$_5$**), 84.7 (**C$_{13}$**), 83.1 (**C$_{14}$**), 82.1 (dd, $^3J_{CF}$ = 5.1 Hz, $^3J_{CF}$ = 1.0 Hz, **C$_9$**), 78.8 (d, $^4J_{CF}$ = 1.9 Hz, **C$_{11}$**), 63.2 (**C$_{15}$**), 53.9 (dd, $^2J_{CF}$ = 27.2 Hz, $^2J_{CF}$ =19.5 Hz, **C$_8$**), 41.8 (**C$_{10}$**), 39.8 (dd, $^3J_{CF}$ = 4.5 Hz, $^3J_{CF}$ = 2.1 Hz, **C$_{12}$**), 38.9 (**C$_{16}$**), 33.5 (**C$_2$**), 31.6 (**C$_{17}$**), 26.0 (SiC(*C*H$_3$)$_3$), 25.8 (SiC(*C*H$_3$)$_3$), 25.1 (**C$_{18}$**), 24.3 (d, $^4J_{CF}$ = 2.8 Hz, **C$_4$**), 24.1 (d, $^5J_{CF}$ = 1.2 Hz, **C$_3$**), 22.7 (**C$_{19}$**), 18.4 (Si*C*(CH$_3$)$_3$), 18.2 (Si*C*(CH$_3$)$_3$), 14.2 (**C$_{20}$**), -4.4 (Si*C*H$_3$), -4.6 (Si*C*H$_3$), -4.8 (Si*C*H$_3$), -4.9 (Si*C*H$_3$).

HRMS (ESI$^+$) calculated for $C_{32}H_{56}F_2NaO_5Si_2$ [M+Na$^+$] 637.3527, found 637.3529.

5-((3a*R*,4*S*,5*R*,6a*S*)-4-((*S*)-3-(*tert*-butyldimethylsilyloxy)oct-1-ynyl)-3,3-difluoro-5-hydroxyhexahydro-2*H*-cyclopenta[*b*]furan-2-ylidene)pentanoic acid (42'):

***R*$_f$** = 0.24 (Hexanes/EtOAc/AcOH = 75:24:1)

IR (thin film) ν_{max}/cm^{-1}: 3345, 2955, 2929, 2853, 1710, 1453, 1295, 1255, 1203, 1084, 1037, 965, 835, 775.

^{1}H NMR (400 MHz, $CDCl_3$) (*Z*)-**42**: δ_H = 4.90 – 4.80 (m, 2H, *H*C$_5$+*H*C$_9$), 4.32 (td, *J* = 6.8, 1.3 Hz, 1H, *H*C$_{15}$), 4.25 (q, *J* = 5.4 Hz, 1H, *H*C$_{11}$), 2.99 – 2.91 (m, 2H, *H*C$_8$+*H*C$_{12}$), 2.39 – 2.34 (m, 3H, H_AC$_{10}$+H_2C$_2$), 2.19 – 2.10 (m, 2H, H_2C$_4$), 2.04 – 1.99 (m, 1H, H_BC$_{10}$), 1.83 – 1.68 (m, 2H, H_2C$_3$), 1.65 – 1.59 (m, 2H, H_2C$_{16}$), 1.41 – 1.26 (m, 6H, H_2C$_{17}$+H_2C$_{18}$+H_2C$_{19}$), 0.90 – 0.89 (m, 12H, SiC(CH_3)$_3$+H_3C$_{20}$), 0.11 (s, 3H, SiCH_3), 0.09 (s, 3H, SiCH_3).

^{19}F NMR (377 MHz, $CDCl_3$) (*Z*)-**42**: δ_F = -83.47 (ddd, $^2J_{FF}$ = 252.9 Hz, $^3J_{HF}$ = 16.2 Hz, $^4J_{HF}$ = 4.4 Hz), -112.51 (d, $^2J_{FF}$ = 252.9 Hz); (*E*)-**42**: δ_F = -85.00 (ddd, $^2J_{FF}$ = 260.4 Hz, $^3J_{HF}$ = 18.7 Hz, $^4J_{HF}$ = 3.2 Hz), -106.01 (d, $^2J_{FF}$ = 260.3 Hz).

^{13}C NMR (100 MHz, $CDCl_3$) (*Z*)-**42**: δ_C = 178.9 (**C$_1$**), 149.4 (dd, $^2J_{CF}$ = 30.1 Hz, $^2J_{CF}$ = 25.8 Hz, **C$_6$**), 123.5 (dd, $^1J_{CF}$ = 255.2 Hz, $^1J_{CF}$ = 241.2 Hz, **C$_7$**), 100.8 (d, $^3J_{CF}$ = 4.4 Hz, **C$_5$**), 85.3 (**C$_{13}$**), 83.1 (d, $^3J_{CF}$ = 4.3 Hz, **C$_9$**), 82.6 (**C$_{14}$**), 78.9 (d, $^4J_{CF}$ = 1.5 Hz, **C$_{11}$**), 63.2 (**C$_{15}$**), 54.9 (dd, $^2J_{CF}$ = 27.1, $^2J_{CF}$ = 19.6 Hz, **C$_8$**), 40.6 (**C$_{10}$**), 40.2 (dd, $^3J_{CF}$ = 4.9, $^3J_{CF}$ = 2.3 Hz, **C$_{12}$**), 38.8 (**C$_{16}$**), 33. 2 (**C$_2$**), 31.5 (**C$_{17}$**), 25.9 (SiC(*C*H$_3$)$_3$), 25.1 (**C$_{18}$**), 24.2 (d, $^4J_{CF}$ = 2.8 Hz, **C$_4$**), 23.8 (d, $^5J_{CF}$ = 1.5 Hz, **C$_3$**), 22.7 (**C$_{19}$**), 18.4 (Si*C*(CH$_3$)$_3$), 14.1 (**C$_{20}$**), -4.4 (Si*C*H$_3$), -4.8 (Si*C*H$_3$).

HRMS (ESI$^+$) calculated for $C_{26}H_{42}F_2NaO_5Si$ [M+Na$^+$] 523.2662, found 523.2663.

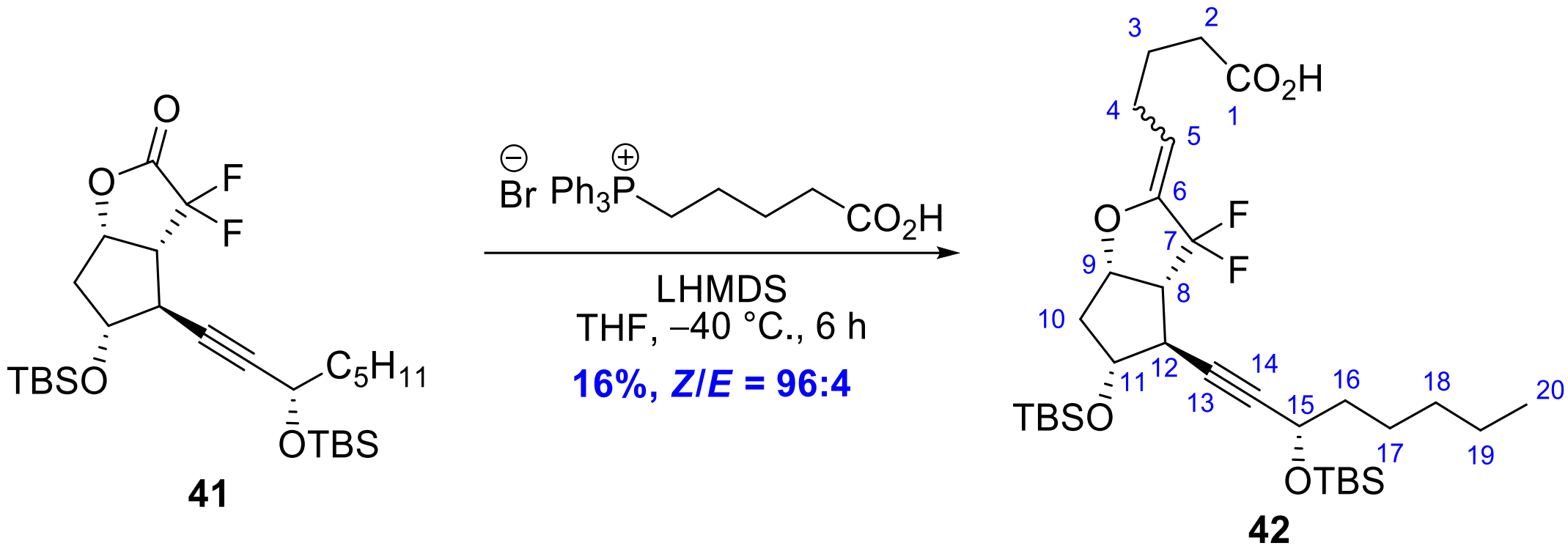

LiHMDS (1.0 M in THF, 0.96 mL, 0.96 mmol, 3.2 eq.) was added to a stirring suspension of (4-carboxybutyl)triphenylphosphonium bromide (200 mg, 0.45 mmol, 1.5 eq.) in THF (1.5 mL). The resulting orange suspension was stirred at r.t. for 1 h. **41** (159 mg, 0.30 mmol, 1.0 eq.) was added as a solution in THF (1.5 mL) to the orange suspension and the reaction was stirred at –40 °C for 6 h. The reaction mixture was quenched by addition of water (30 mL) and extracted with EtOAc (3 × 30 mL). The combined organic phases were washed with water (20 mL), treated with aqueous HCl (1.0 M, 30 mL), washed with brine, dried over $MgSO_4$, filtered and concentrated *in vacuo*. The crude product was purified by flash column chromatography on silica gel (Hexanes/EtOAc = 95:5; then Hexanes/EtOAc/AcOH = 90:9:1) to give **42** as a colourless oil (29.0 mg, 47.2 µmol, 16%, 96:4 *Z*/*E*).

R_f = 0.27 (Hexanes/EtOAc/AcOH = 80:19:1)
^{19}F NMR (377 MHz, $CDCl_3$) (*Z*)-**42**: δ_F = -82.84 (dddd, $^2J_{FF}$ = 252.7 Hz, $^3J_{HF}$ = 16.9 Hz, $^3J_{HF}$ = 9.0 Hz, $^4J_{HF}$ = 4.4 Hz), 112.28 (d, $^2J_{FF}$ = 252.7 Hz); (*E*)-**42**: δ_F = -84.27 (ddd, $^2J_{FF}$ = 261.0 Hz, $^3J_{HF}$ =18.7 Hz, $^4J_{HF}$ = 3.4 Hz), -106.07 (d, $^2J_{FF}$ = 260.3 Hz).
$[\alpha]_D^{24}$= –12.8 (*c* 0.70, $CHCl_3$)

5-((3a*R*,4*S*,5*R*,6a*S*)-3,3-Difluoro-5-hydroxy-4-((*S*)-3-hydroxyoct-1-ynyl)hexahydro-2*H*-cyclopenta[*b*]furan-2-ylidene)pentanoic acid (8)

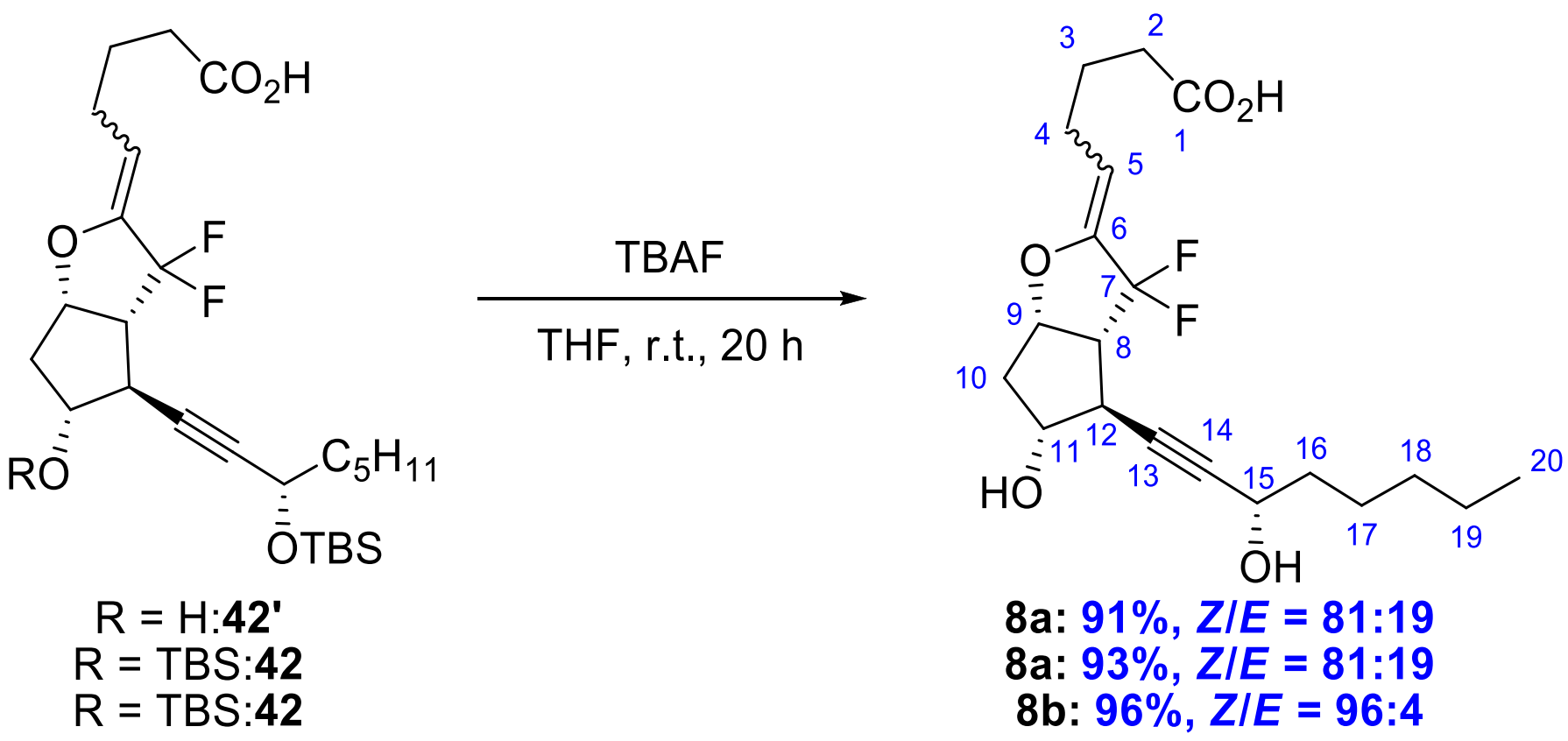

To a stirring solution of **42'** (15.0 mg, 30.0 μmol, 1.0 eq., 81:19 *Z*/*E*) in THF (0.3 mL) at 0 °C, TBAF (1.0 M in THF, 90.0 μL, 90.0 μmol, 3.0 eq.) was added dropwise. The reaction mixture was warmed to r.t. and stirred for a further 20 h. The reaction was quenched by addition of H_2O (1.5 mL) and the aqueous phase was extracted with Et_2O (3 × 3 mL). The combined organic phases were washed with brine, dried over $MgSO_4$, filtered and concentrated *in vacuo*. The crude product was purified by flash column chromatography on silica gel (CH_2Cl_2/EtOH/AcOH = 97:2:1 to 91:8:1) to give **8a** as a colourless oil (10.6 mg, 27.4 μmol, 91%, 81:19 *Z*/*E*).

To a stirring solution of **42** (6.00 mg, 9.80 μmol, 1.0 eq., 81:19 *Z*/*E*) in THF (0.2 mL) at 0 °C, TBAF (1.0 M in THF, 29.3 μL, 29.3 mmol, 3.0 eq.) was added dropwise. The reaction mixture was warmed to r.t. and stirred for a further 20 h. The reaction was quenched by addition of H_2O (0.5 mL) and the aqueous phase was extracted with Et_2O (3 × 1.5 mL). The combined organic phases were washed with brine, dried over $MgSO_4$, filtered and concentrated *in vacuo*. The crude product was purified by flash column chromatography on silica gel (CH_2Cl_2/EtOH/AcOH = 97:2:1 to 91:8:1) to give **8a** as a colourless oil (3.50 mg, 9.10 μmol, 93%, 81:19 *Z*/*E*).

$\boldsymbol{R_f}$ = 0.21 (CH_2Cl_2/EtOH/AcOH = 95:4:1)
IR (thin film) $\boldsymbol{\nu_{max}}$/cm^{-1}: 3342, 2953, 2929, 2859, 1707, 1375, 1298, 1240, 1194, 1072, 1039, 849, 750.
^{1}H NMR (500 MHz, $CDCl_3$) (*Z*)-**8**: δ_H = 4.88 – 4.83 (m, 2H, ***H***C_5+***H***C_9), 4.35 (t, *J* = 6.5 Hz, 1H, ***H***C_{15}), 4.25 – 4.21 (m, 1H, ***H***C_{11}), 4.05 (overlapping, br.s+br.s, 2H, O***H***+O***H***), 2.94 – 2.87 (m, 2H, ***H***C_8+***H***C_{12}), 2.49 – 2.44 (m, 1H, $\boldsymbol{H_A}C_{10}$), 2.38 – 2.32 (m, 2H, $\boldsymbol{H_2}C_2$), 2.23 – 2.19 (m, 1H, $\boldsymbol{H_A}C_4$), 2.14 – 2.06 (m, 1H, $\boldsymbol{H_B}C_4$), 1.97 (dd, *J* = 14.2, 6.1 Hz, 1H, $\boldsymbol{H_B}C_{10}$), 1.85 – 1.78 (m, 1H, $\boldsymbol{H_A}C_3$), 1.72 – 1.64 (m, 3H, $\boldsymbol{H_A}C_3$+$\boldsymbol{H_2}C_{16}$), 1.45 – 1.39 (m, 2H, $\boldsymbol{H_2}C_{19}$), 1.34 – 1.25 (m, 4H, $\boldsymbol{H_2}C_{17}$+$\boldsymbol{H_2}C_{18}$), 0.90 ((t, *J* = 6.9 Hz, 3H, $\boldsymbol{H_3}C_{20}$).
^{19}F NMR (470 MHz, $CDCl_3$) (*Z*)-**8**: δ_F = -84.24 (dd, $^2J_{FF}$ = 252.4 Hz, $^3J_{HF}$ = 13.7 Hz), -114.34 (d, $^2J_{FF}$ = 252.6 Hz); (*E*)-**8**: δ_F = -85.22 (dd, $^2J_{FF}$ = 260.4 Hz, $^3J_{HF}$ = 17.7 Hz), -107.24 (d, $^2J_{FF}$ = 260.4 Hz).
^{13}C NMR (125 MHz, $CDCl_3$) (*Z*)-**8**: δ_C = 178.6 (**C_1**), 149.1 (dd, $^2J_{CF}$ = 30.2 Hz, $^2J_{CF}$ = 25.8 Hz, **C_6**), 123.5 (dd, $^1J_{CF}$ = 254.9 Hz, $^1J_{CF}$ = 241.5 Hz, **C_7**), 101.0 (d, $^3J_{CF}$ = 4.5 Hz, **C_5**), 84.2 (**C_{13}**), 83.8 (**C_{14}**), 82.3 (d, $^3J_{CF}$ = 5.6 Hz, **C_9**), 78.7 (**C_{11}**), 62.7 (**C_{15}**), 54.2 (dd, $^2J_{CF}$ = 26.9 Hz, $^2J_{CF}$ = 19.7 Hz, **C_8**), 40.4 (**C_{10}**), 39.7 (d, $^3J_{CF}$ = 4.2 Hz, **C_{12}**), 38.0 (**C_{16}**), 31.5 (**C_2**), 29.9 (**C_{17}**), 25.0 (**C_{18}**), 24.1 (**C_4**), 23.8 (**C_3**), 22.7 (**C_{19}**), 14.1 (**C_{20}**).

HRMS (ESI^+) calculated for $C_{20}H_{28}F_2NaO_5$ [$M+Na^+$] 409.1797, found 409.1806.

To a stirring solution of **42** (16.6 mg, 27.0 µmol, 1.0 eq., 96:4 *Z*/*E*) in THF (0.27 mL) at 0 °C, TBAF (1.0 M in THF, 81.0 µL, 81.0 mmol, 3.0 eq.) was added dropwise. The reaction mixture was warmed to r.t. and stirred for a further 20 h. The reaction was quenched by addition of H2O (1 mL) and the aqueous phase was extracted with Et_2O (3 × 2 mL). The combined organic phases were washed with brine, dried over $MgSO_4$, filtered and concentrated *in vacuo*. The crude product was purified by flash column chromatography on silica gel (CH_2Cl_2/EtOH/AcOH = 97:2:1 to 91:8:1) to give **8b** as a colourless oil (10.0 mg, 25.9 µmol, 96%, 96:4 *Z*/*E*).

$\boldsymbol{R_f}$ = 0.21 (CH_2Cl_2/EtOH/AcOH = 95:4:1)
^{19}F NMR (470 MHz, $CDCl_3$) (*Z*)-**8**: δ_F = -84.35 (dd, $^2J_{FF}$ =252.4 Hz, $^3J_{HF}$ = 14.4 Hz), -114.69 (d, $^2J_{FF}$ = 252.4 Hz); (*E*)-**8**: δ_F = -85.19 (dd, $^2J_{FF}$ = 260.8 Hz, $^3J_{HF}$ = 17.9 Hz), -107.35 (d, $^2J_{FF}$ = 260.3 Hz).

(3a*R*,4*S*,5*R*,6a*S*)-5-(*tert*-Butyldimethylsilyloxy)-4-((3*S*,4*S*)-3-(*tert*-butyldimethylsilyloxy)-4-methylnona-1,6-diynyl)-3,3-difluorohexahydro-2*H*-cyclopenta[*b*]furan-2-one (43)

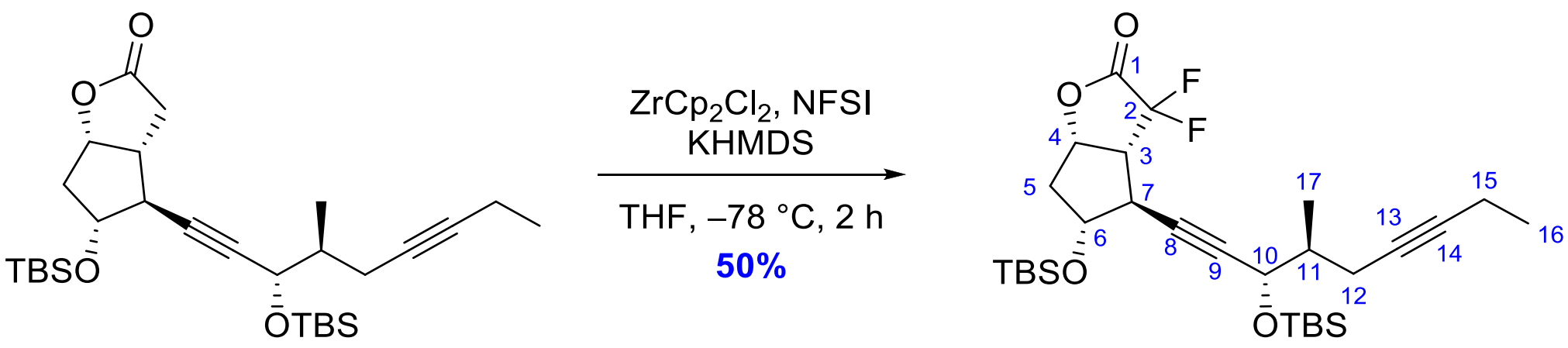

36 **43**

A solution of NFSI (1.00 g, 3.20 mmol, 4.0 eq.) and $ZrCp_2Cl_2$ (935 mg, 3.20 mmol, 4.0 eq.) in THF (10.0 mL) was stirred at r.t. for 1 h and then cooled to –78 °C. A solution of **36** (415 mg, 0.80 mmol, 1.0 eq.) in THF (5.3 mL) was added followed by the drop-wise addition of KHMDS (1.0 M in THF, 4.00 mL, 4.00 mmol, 5.0 eq.) over 1 h, the reaction mixture was then stirred at –78 °C for a further hour. The reaction mixture was warmed to r.t., quenched by addition of saturated $NaHCO_3$ solution (10 mL) and the aqueous phase was subsequently extracted with Et_2O (3 × 10 mL). The organic phases were combined, washed with brine, dried over $MgSO_4$, filtered and concentrated *in vacuo*. The crude product was purified by flash column chromatography on silica gel (Hexanes/Et_2O = 100:0 to 95:5) to give pure **43** as a yellow oil (223 mg, 0.40 mmol, 50%).

$\boldsymbol{R_f}$ = 0.33 (Hexanes/Et_2O = 95:5)

IR (thin film) ν_{max}/cm^{-1}: 2954, 2929, 2857, 1816, 1472, 1362, 1319, 1239, 1204, 1150, 1089, 1074, 998, 834, 775.

^{1}H NMR (400 MHz, CDCl$_3$) δ_H = 5.23 (t, J = 6.0 Hz, 1H, ***H***C$_4$), 4.41 – 4.27 (m, 2H, ***H***C$_6$+***H***C$_{10}$), 3.30 – 3.22 (m, 2H, ***H***C$_3$+***H***C$_7$), 2.29 – 2.23 (m, 1H, ***H***C$_{11}$), 2.21 – 2.13 (m, 5H, ***H$_A$***C$_5$+***H$_2$***C$_{12}$+***H$_2$***C$_{15}$), 1.81 (dt, J = 13.0, 6.4 Hz, 1H, ***H$_B$***C$_5$), 1.12 (t, J = 7.4 Hz, 3H, ***H$_3$***C$_{16}$), 1.01 (d, J = 6.8 Hz, 3H, ***H$_3$***C$_{17}$), 0.90 (s, 9H, SiC(C***H$_3$***)$_3$), 0.85 (s, 9H, SiC(C***H$_3$***)$_3$), 0.10 (s, 3H, SiC***H$_3$***), 0.09 (s, 3H, SiC***H$_3$***), 0.07 (s, 3H, SiC***H$_3$***), 0.06 (s, 3H, SiC***H$_3$***).

^{19}F NMR (377 MHz, CDCl$_3$) δ_F = -91.54 (dd, $^2J_{FF}$ = 281.9 Hz, $^3J_{HF}$ = 26.2 Hz), -112.51 (d, $^2J_{FF}$ = 281.8 Hz).

^{13}C NMR (100 MHz, CDCl$_3$) δ_C = 165.1 (dd, $^2J_{CF}$ = 34.8 Hz, $^2J_{CF}$ =32.8 Hz, **C$_1$**), 115.2 (dd, $^1J_{CF}$ = 265.9 Hz, $^1J_{CF}$ = 244.4 Hz, **C$_2$**), 84.9 (**C$_8$**), 83.5 (**C$_{14}$**), 82.8 (**C$_9$**), 81.9 (d, $^3J_{CF}$ = 2.9 Hz, $^3J_{CF}$ = 2.5 Hz, **C$_4$**), 78.7 (**C$_6$**), 77.1 (**C$_{13}$**), 66.3 (**C$_{10}$**), 51.7 (dd, $^2J_{CF}$ = 25.0 Hz, $^2J_{CF}$ = 18.5 Hz, **C$_3$**), 41.4 (dd, $^3J_{CF}$ = 5.9 Hz, $^3J_{CF}$ = 4.1 Hz, **C$_7$**), 40.5 (**C$_5$**), 40.1 (**C$_{11}$**), 25.9 (SiC(***C***H$_3$)$_3$), 25.7 (SiC(***C***H$_3$)$_3$), 22.1 (**C$_{12}$**), 18.3 (Si***C***(CH$_3$)$_3$), 18.2 (Si***C***(CH$_3$)$_3$), 15.3 (**C$_{17}$**), 14.5 (**C$_{16}$**), 12.6 (**C$_{15}$**), -4.5 (Si***C***H$_3$), -5.0 (Si***C***H$_3$), -5.1 (Si***C***H$_3$), -5.1 (Si***C***H$_3$).

HRMS (ESI$^+$) calculated for $C_{29}H_{48}F_2NaO_4Si_2$ [M+Na$^+$] 577.2951, found 577.2960.

[α]$_D$^{24} = –16.1 (c 0.79, CHCl$_3$)

5-((3a*R*,4*S*,5*R*,6a*S*)-5-(*tert*-Butyldimethylsilyloxy)-4-((3*S*,4*S*)-3-(*tert*-butyldimethylsilyloxy)-4-methylnona-1,6-diynyl)-3,3-difluorohexahydro-2*H*-cyclopenta[*b*]furan-2-ylidene)pentanoic acid (44)

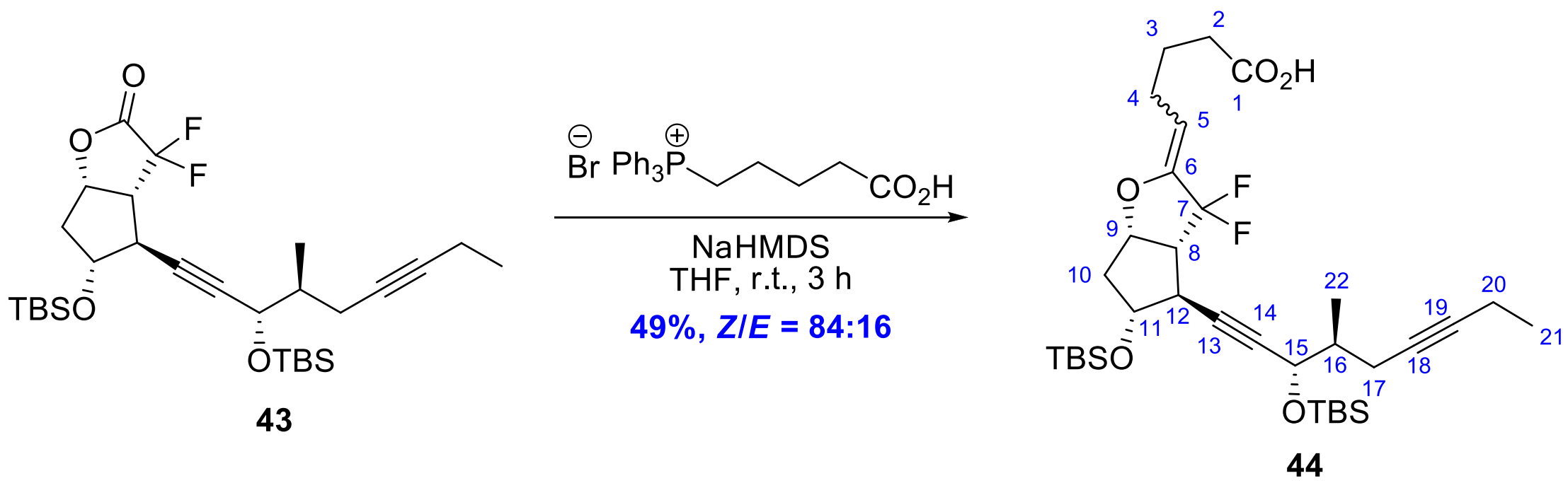

NaHMDS (1.0 M in THF, 1.26 mL, 1.26 mmol, 10.0 eq.) was added to a stirring suspension of (4-carboxybutyl)triphenylphosphonium bromide (279 mg, 0.63 mmol, 5.0 eq.) in THF (0.89 mL). The resulting orange suspension was heated to 70 °C for 1 h before cooling to r.t.. **43** (70.0 mg, 126 µmol, 1.0 eq.) was added as a solution in THF (0.13 mL) to the orange suspension and the reaction was stirred at r.t. for 3 h. The reaction mixture was quenched by addition of water (25.2 mL) and extracted with EtOAc (3 × 12.5 mL). The combined organic

phases were washed with water (12.5 mL), treated with aqueous HCl (0.1 M, 12.5 mL), washed with brine, dried over $MgSO_4$, filtered and concentrated *in vacuo*. The crude product was purified by flash column chromatography on silica gel (Hexanes/EtOAc = 95:5 to 90:10; then Hexanes/EtOAc/AcOH = 90:9:1 to 80:19:1) to give **44** as a colourless oil (39.5 mg, 61.8 μmol, 49%, 84:16 *Z*/*E*).

$\boldsymbol{R}_\mathbf{f}$ = 0.28 (Hexanes/EtOAc/AcOH = 80:19:1)

IR (thin film) $\boldsymbol{\nu}_{\mathbf{max}}$/cm^{-1}: 2954, 2927, 2855, 1710, 1452, 1378, 1322, 1250, 1203, 1142, 1079, 1049, 1003, 836, 777.

^{1}H NMR (500 MHz, $CDCl_3$) (*Z*)-**44**: δ_H = 4.83 – 4.75 (m, 2H, $\boldsymbol{H}C_5$+$\boldsymbol{H}C_9$), 4.30 (dd, *J* = 6.6, 1.1 Hz, 1H, $\boldsymbol{H}C_{15}$), 4.17 (q, *J* = 6.2 Hz, 1H, $\boldsymbol{H}C_{11}$), 2.94 – 2.82 (m, 2H, $\boldsymbol{H}C_8$+$\boldsymbol{H}C_{12}$), 2.39 – 2.34 (m, 3H, $\boldsymbol{H_2}C_2$+$\boldsymbol{H_A}C_{10}$), 2.25 – 2.13 (m, 5H, $\boldsymbol{H_A}C_4$+$\boldsymbol{H_2}C_{17}$+$\boldsymbol{H_2}C_{20}$), 1.90 – 1.86 (m, 1H, $\boldsymbol{H_B}C_4$), 1.84 – 1.79 (m, 1H, $\boldsymbol{H_B}C_{10}$), 1.76 – 1.71 (m, 2H, $\boldsymbol{H_A}C_3$+$\boldsymbol{H}C_{16}$), 1.63 – 1.58 (m, 1H, $\boldsymbol{H_B}C_3$), 1.12 (t, *J* = 7.4 Hz, 3H, $\boldsymbol{H_3}C_{21}$), 1.03 (d, *J* = 6.8 Hz, 3H, $\boldsymbol{H_3}C_{22}$), 0.90 (s, 9H, $SiC(C\boldsymbol{H_3})_3$), 0.87 (s, 9H, $SiC(C\boldsymbol{H_3})_3$), 0.12 (s, 3H, $SiC\boldsymbol{H_3}$), 0.10 (s, 3H, $SiC\boldsymbol{H_3}$), 0.08 (s, 3H, $SiC\boldsymbol{H_3}$), 0.07 (s, 3H, $SiC\boldsymbol{H_3}$).

^{19}F NMR (470 MHz, $CDCl_3$) (*Z*)-**44**: δ_F = -82.70 (ddd, $^2J_{FF}$ = 252.7 Hz, $^3J_{HF}$ =16.9 Hz, $^4J_{HF}$ = 4.5 Hz), -112.26 (d, $^2J_{FF}$ = 252.6 Hz); (*E*)-**44**: δ_F = -84.16 (ddd, $^2J_{FF}$ = 260.1 Hz, $^3J_{HF}$ = 19.2 Hz, $^4J_{HF}$ = 3.2 Hz), -106.01 (d, $^2J_{FF}$ = 260.2 Hz).

^{13}C NMR (125 MHz, $CDCl_3$) (*Z*)-**44**: δ_C = 178.9 (**C$_1$**), 149.5 (dd, $^2J_{CF}$ = 29.9 Hz, $^2J_{CF}$ = 25.9 Hz, **C$_6$**), 123.6 (dd, $^1J_{CF}$ = 255.2 Hz, $^1J_{CF}$ =240.7 Hz, **C$_7$**), 100.2 (d, $^3J_{CF}$ = 4.4 Hz, **C$_5$**), 84.3 (**C$_{13}$**), 83.2 (**C$_{19}$**), 83.1 (**C$_{14}$**), 82.2 (d, $^3J_{CF}$ = 4.3 Hz, **C$_9$**), 78.8 (d, $^4J_{CF}$ = 1.8 Hz, **C$_{11}$**), 77.5 (**C$_{18}$**), 66.4 (**C$_{15}$**), 54.0 (dd, $^2J_{CF}$ = 27.2 Hz, $^2J_{CF}$ = 19.4 Hz, **C$_8$**), 41.8 (**C$_{10}$**), 40.2 (**C$_{16}$**), 39.9 (dd, $^3J_{CF}$ = 4.4 Hz, $^3J_{CF}$ = 2.1 Hz, **C$_{12}$**), 29.9 (**C$_2$**), 25.9 ($SiC(\boldsymbol{C}H_3)_3$), 25.8 ($SiC(\boldsymbol{C}H_3)_3$), 24.3 (d, $^5J_{CF}$ = 2.8 Hz, **C$_3$**), 24.1 (d, $^4J_{CF}$ = 0.7 Hz, **C$_4$**), 22.0 (**C$_{17}$**), 18.3 ($Si\boldsymbol{C}(CH_3)_3$), 18.1 ($Si\boldsymbol{C}(CH_3)_3$), 15.3 (**C$_{22}$**), 14.5 (**C$_{21}$**), 12.6 (**C$_{20}$**), -4.4 ($Si\boldsymbol{C}H_3$), -4.6 ($Si\boldsymbol{C}H_3$), -4.7 ($Si\boldsymbol{C}H_3$), -5.0 ($Si\boldsymbol{C}H_3$).

HRMS (ESI^+) calculated for $C_{34}H_{56}F_2NaO_5Si_2$ [$M+Na^+$] 661.3527, found 661.3542.

5-((3a*R*,4*S*,5*R*,6a*S*)-3,3-Difluoro-5-hydroxy-4-((3*S*,4*S*)-3-hydroxy-4-methylnona-1,6-diynyl)hexahydro-2*H*-cyclopenta[*b*]furan-2-ylidene)pentanoic acid (9)

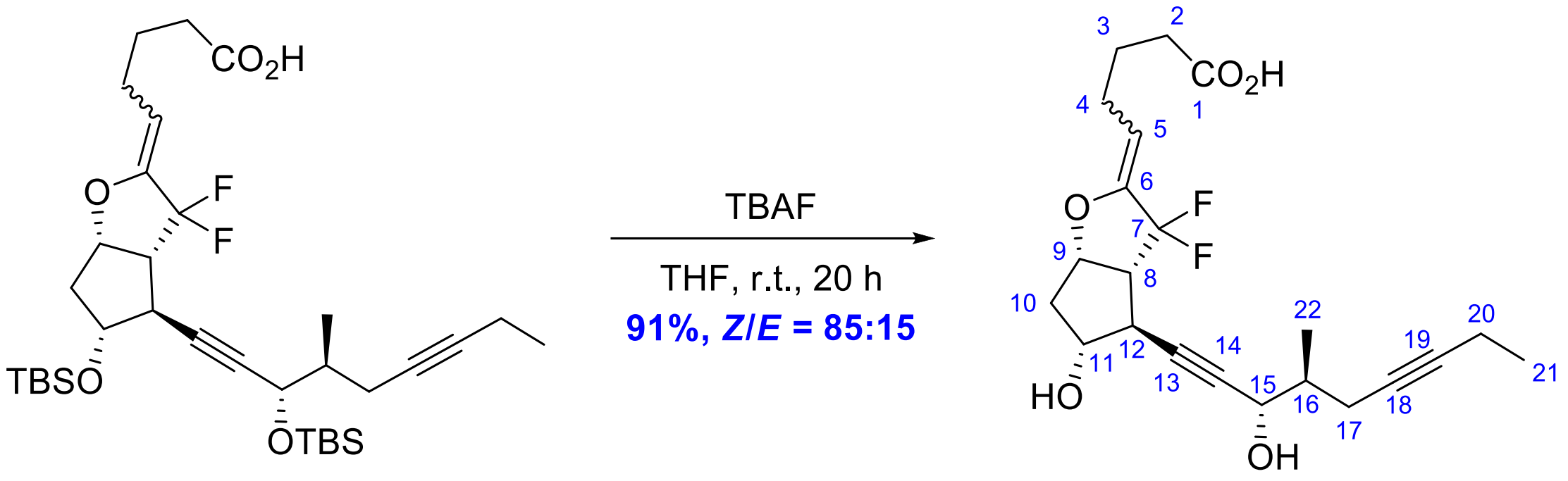

44 **9**

To a stirring solution of **44** (16.0 mg, 25.0 µmol, 1.0 eq.) in THF (0.25 mL) at 0 °C, TBAF (1.0 M in THF, 75.0 µL, 75.0 µmol, 3.0 eq.) was added dropwise. The reaction mixture was warmed to r.t. and stirred for a further 20 h. The reaction was quenched by addition of H_2O (1.2 mL) and the aqueous phase was extracted with Et_2O (3 × 2.5 mL). The combined organic phases were washed with brine, dried over $MgSO_4$, filtered and concentrated *in vacuo*. The crude product was purified by flash column chromatography on silica gel (CH_2Cl_2/EtOAc/AcOH = 60:39:1 to 40:59:1) to give **9** as a colourless oil (9.40 mg, 22.7 µmol, 91%, 85:15 *Z*/*E*).

$\boldsymbol{R_f}$ = 0.31 (CH_2Cl_2/EtOAc/AcOH = 40:59:1)

IR (thin film) $\boldsymbol{\nu_{max}}$/cm^{-1}: 3358, 2964, 2925, 2855, 1708, 1458, 1409, 1375, 1299, 1242, 1197, 1074, 1042, 1018, 985, 890, 775.

^{1}H NMR (500 MHz, $CDCl_3$) (*Z*)-**9**: δ_H = 4.88 – 4.83 (m, 2H, ***H***C$_5$+***H***C$_9$), 4.65 (overlapping, br.s+br.s, 2H, O***H***+O***H***), 4.36 (d, *J* = 6.2 Hz, ***H***C$_{15}$), 4.27 – 4.21 (m, 1H, ***H***C$_{11}$), 2.93 – 2.89 (m, 2H, ***H***C$_8$+***H***C$_{12}$), 2.50 – 2.44 (m, 1H, $\boldsymbol{H_A}$C$_{10}$), 2.38 – 2.32 (m, 2H, $\boldsymbol{H_2}$C$_2$), 2.26 – 2.21 (m, 2H, $\boldsymbol{H_2}$C$_{17}$), 2.19 – 2.07 (m, 4H, $\boldsymbol{H_2}$C$_4$+$\boldsymbol{H_2}$C$_{20}$), 2.01 – 1.95 (m, 1H, $\boldsymbol{H_B}$C$_{10}$), 1.92 – 1.87 (m, 1H, ***H***C$_{16}$), 1.85 – 1.78 (m, 1H, $\boldsymbol{H_A}$C$_3$), 1.74 – 1.66 (m, 1H, $\boldsymbol{H_B}$C$_3$), 1.12 (t, *J* = 7.5 Hz, $\boldsymbol{H_3}$C$_{21}$), 1.07 (d, *J* = 6.8 Hz, 3H, $\boldsymbol{H_3}$C$_{22}$).

^{19}F NMR (470 MHz, $CDCl_3$) (*Z*)-**9**: δ_F = -84.33 (dd, $^2J_{FF}$ = 252.8 Hz, $^3J_{HF}$ =14.4 Hz), -114.75 (d, $^2J_{FF}$ = 252.6 Hz); (*E*)-**9**: δ_F = -85.12 (dd, $^2J_{FF}$ = 260.3 Hz, $^3J_{HF}$ = 17.0 Hz), -107.26 (d, $^2J_{FF}$ = 260.3 Hz).

^{13}C NMR (125 MHz, $CDCl_3$) (*Z*)-**9**: δ_C = 178.9 (**C$_1$**), 149.0 (dd, $^2J_{CF}$ = 30.0 Hz, $^2J_{CF}$ = 25.5 Hz, **C$_6$**), 123.5 (dd, $^1J_{CF}$ = 254.9 Hz, $^1J_{CF}$ = 241.4 Hz, **C$_7$**), 101.1 (d, $^3J_{CF}$ = 4.3 Hz, **C$_5$**), 85.0 (**C$_{13}$**), 83.8 (**C$_{19}$**), 82.4 (**C$_{14}$**), 82.2 (d, $^3J_{CF}$ = 5.3 Hz, **C$_9$**), 78.6 (**C$_{11}$**), 77.1 (**C$_{18}$**), 66.2 (**C$_{15}$**), 54.1 (dd,

$^2J_{CF}$ = 27.2 Hz, $^2J_{CF}$ = 19.4 Hz, **C8**), 40.4 (**C10**), 39.7 (d, $^3J_{CF}$ = 4.0 Hz, **C12**), 39.3 (**C16**), 33.4 (**C2**), 24.1 (**C3**), 23.7 (**C4**), 22.3 (**C17**), 15.3 (**C22**), 14.4 (**C21**), 12.6 (**C20**).
HRMS (ESI^+) calculated for $C_{22}H_{28}F_2NaO_5$ [$M+Na^+$] 433.1797, found 433.1780.

Synthesis of monofluorinated targets 4 and 5

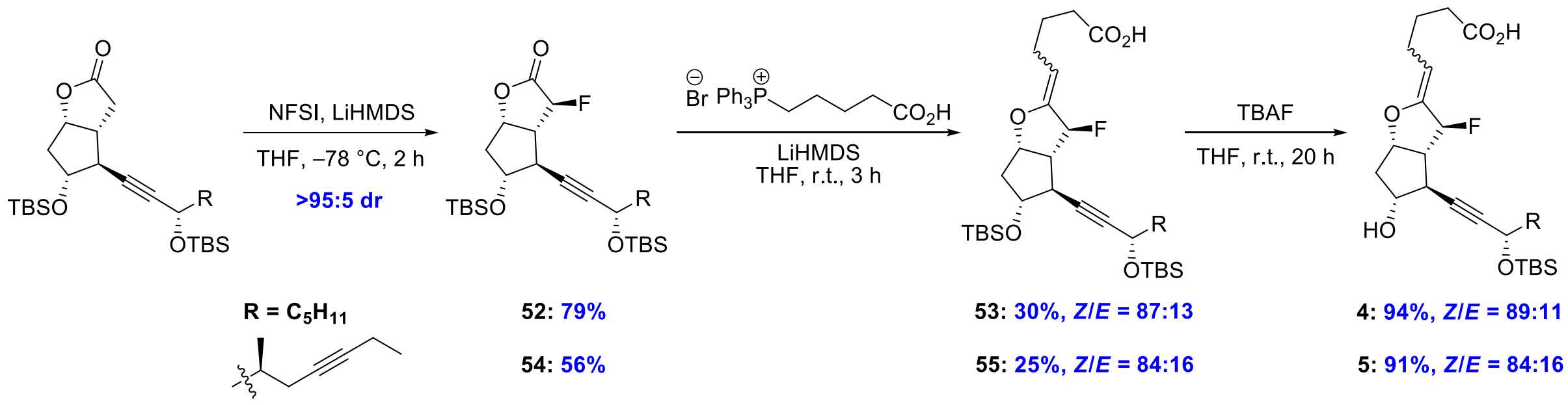

(3*S*,3a*R*,4*S*,5*R*,6a*S*)-5-(*tert*-Butyldimethylsilyloxy)-4-((*S*)-3-(*tert*-butyldimethylsilyloxy)oct-1-ynyl)-3-fluorohexahydro-2*H*-cyclopenta[*b*]furan-2-one (52)

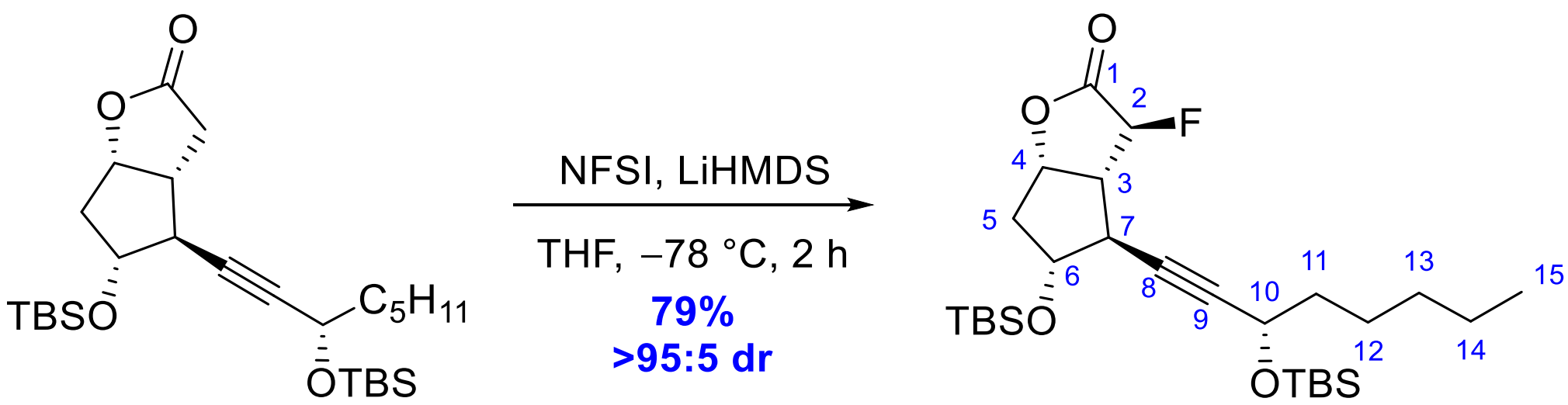

To a solution of **32** (650 mg, 1.31 mmol, 1.0 eq.) and NFSI (517 mg, 1.64 mmol, 1.25 eq.) in THF (6.5 mL) at –78 °C, LiHMDS (1.0 M in THF, 1.51 mL, 1.51 mmol, 1.15 eq.) was added dropwise. The reaction mixture was stirred at –78 °C for 2 h before it was warmed to r.t., quenched by addition of saturated $NaHCO_3$ solution (13 mL) and the aqueous phase was extracted with Et_2O (3 × 13 mL). The organic phases were combined, washed with brine, dried over $MgSO_4$, filtered and concentrated *in vacuo*. The crude product was purified by flash column chromatography on silica gel (Hexanes/Et_2O = 100:0 to 95:5) to give pure **52** as a yellow oil (531 mg, 1.03 mmol, 79%, >95:5 dr).

R_f = 0.33 (Hexanes/Et_2O = 95:5)
IR (thin film) ν_{max}/cm^{-1}: 2954, 2929, 2858, 1810, 1470, 1406, 1367, 1335, 1240, 1080, 1030, 990, 835, 775.
^{1}H NMR (400 MHz, $CDCl_3$) δ_H = 5.22 (t, *J* = 7.2 Hz, 1H, ***H*C4**), 5.05 (dd, *J* = 51.7, 2.7 Hz, 1H, ***H*C2**), 4.28 (d, *J* = 6.1, 1.8 Hz, 1H, ***H*C10**), 4.27 (dd, *J* = 6.8, 1.8 Hz, 1H, ***H*C6**), 3.20 (dd, *J* =

32.2, 7.6 Hz, 1H, ***H***C_3), 3.04 (s, 1H, ***H***C_7), 2.25 (ddd, J = 15.1, 6.8, 3.7 Hz, 1H, ***H_A***C_5), 2.07 (d, J = 15.1 Hz, 1H, ***H_B***C_5), 1.66 – 1.54 (m, 2H, ***H_2***C_{11}), 1.41 – 1.23 (m, 6H, ***H_2***C_{12}+***H_2***C_{13}+***H_2***C_{14}), 0.89 – 0.88 (m, 12H, SiC(C***H_3***)3+***H_3***C_{15}), 0.84 (s, 9H, SiC(C***H_3***)3), 0.08 (s, 3H, SiC***H_3***), 0.07 (s, 6H, SiC***H_3***+SiC***H_3***), 0.05 (s, 3H, SiC***H_3***).

^{19}F NMR (377 MHz, $CDCl_3$) δ_F = -176.50 (dd, $^2J_{HF}$ = 51.7 Hz, $^3J_{HF}$ =32.9 Hz).

^{13}C NMR (100 MHz, $CDCl_3$) δ_C = 171.7 (d, $^2J_{CF}$ = 21.4 Hz, **C1**), 91.4 (d, $^1J_{CF}$ = 184.4 Hz, **C2**), 86.7 (**C8**), 83.1 (d, $^3J_{CF}$ = 3.0 Hz, **C4**), 81.9 (**C9**), 78.6 (**C6**), 63.1 (**C10**), 52.0 (d, $^2J_{CF}$ = 21.2 Hz, **C3**), 44.6 (d, $^3J_{CF}$ = 5.4 Hz, **C7**), 40.1 (d, $^4J_{CF}$ = 1.2 Hz, **C5**), 38.7 (**C11**), 31.5 (**C12**), 25.9 (SiC(***C***H_3)$_3$), 25.7 (SiC(***C***H_3)$_3$), 25.0 (**C13**), 22.7 (**C14**), 18.4 (Si***C***(CH_3)$_3$), 18.1 (Si***C***(CH_3)$_3$), 14.1 (**C15**), -4.5 (Si***C***H_3), -4.8 (Si***C***H_3), -4.9 (Si***C***H_3), -5.1 (Si***C***H_3).

HRMS (ESI$^+$) calculated for $C_{27}H_{49}FNaO_4Si_2$ [M+Na$^+$] 535.3046, found 535.3030.

$[\alpha]_D^{24}$ = –56.3 (c 1.0, $CHCl_3$)

5-((3*S*,3a*R*,4*S*,5*R*,6a*S*)-5-(*tert*-Butyldimethylsilyloxy)-4-((*S*)-3-(*tert*-butyldimethylsilyloxy)oct-1-ynyl)-3-fluorohexahydro-2*H*-cyclopenta[*b*]furan-2-ylidene)pentanoic acid (53)

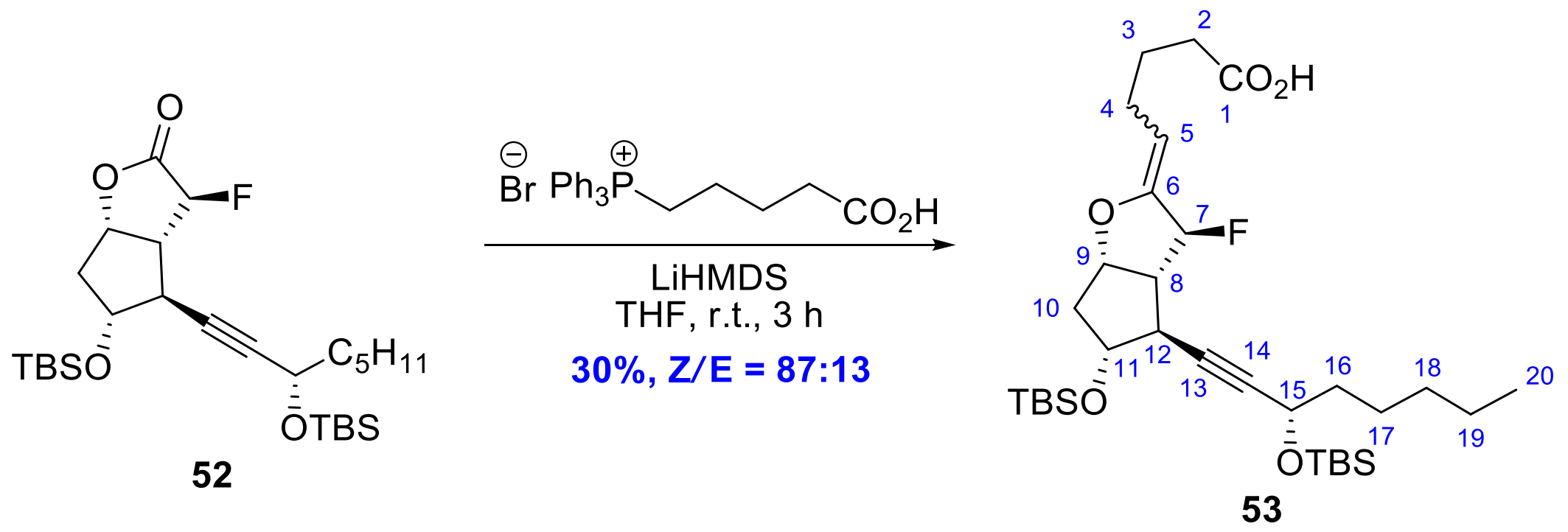

LiHMDS (1.0 M in THF, 0.64 mL, 0.64 mmol, 3.2 eq.) was added to a stirring suspension of (4-carboxybutyl)triphenylphosphonium bromide (133 mg, 0.30 mmol, 1.5 eq.) in THF (1.0 mL). The resulting orange suspension was stirred at r.t. for 1 h. **52** (103 mg, 0.20 mmol, 1.0 eq.) was added as a solution in THF (1.0 mL) to the orange suspension and the reaction was stirred at r.t. for 3 h. The reaction mixture was quenched by addition of water (20 mL) and extracted with EtOAc (3 × 20 mL). The combined organic phases were washed with water (20 mL), treated with aqueous HCl (0.1 M, 20 mL), washed with brine, dried over $MgSO_4$, filtered and concentrated *in vacuo*. The crude product was purified by flash column chromatography on silica gel (Hexanes/EtOAc = 95:5; then Hexanes/EtOAc/AcOH = 95:4:1) to give **53** as a colourless oil (35.8 mg, 60.0 µmol, 30%, 87:13 *Z*/*E*).

R_f = 0.36 (Hexanes/EtOAc/AcOH = 80:19:1).

IR (thin film) ν_{max}/cm^{-1}: 2953, 2928, 2855, 1709, 1453, 1471, 1453, 1441, 1251, 1235, 1130, 1085, 1035, 968, 894, 836, 777.

^{1}H NMR (500 MHz, CDCl$_3$) (*Z*)-**53**: δ_H = 5.11 (d, *J* = 55.8 Hz, 1H, ***H***C$_7$), 4.84 (td, *J* = 6.6, 2.3 Hz, 1H, ***H***C$_5$), 4.68 (q, *J* = 7.3 Hz, 1H, ***H***C$_9$), 4.33 (td, *J* = 6.5, 1.7 Hz, 1H, ***H***C$_{15}$), 4.11 (q, *J* = 7.1 Hz, 1H, ***H***C$_{11}$), 2.73 (ddd, *J* = 20.8, 9.6, 6.2 Hz, 1H, ***H***C$_8$), 2.41 – 2.35 (m, 4H, ***H$_2$***C$_2$+***H$_A$***C$_{10}$+***H***C$_{12}$), 2.17 – 2.14 (m, 2H, ***H$_2$***C$_4$), 1.76 – 1.70 (m, 3H, ***H$_B$***C$_{10}$+***H$_2$***C$_3$), 1.65 – 1.59 (m, 2H, ***H$_2$***C$_{16}$), 1.37 – 1.25 (m, 6H, ***H$_2$***C$_{17}$+***H$_2$***C$_{18}$+***H$_2$***C$_{19}$), 0.91 – 0.90 (m, 12H, SiC(C***H$_3$***)$_3$+***H$_3$***C$_{20}$), 0.87 (s, 9H, SiC(C***H$_3$***)$_3$), 0.11 (s, 3H, SiC***H$_3$***), 0.10 (s, 3H, SiC***H$_3$***), 0.08 (s, 3H, SiC***H$_3$***), 0.07 (s, 3H, SiC***H$_3$***).

^{19}F NMR (470 MHz, CDCl$_3$) (*Z*)-**53**: δ_F = -158.36 (ddd, $^2J_{HF}$ = 55.8 Hz, $^3J_{HF}$ = 21.0 Hz, $^4J_{HF}$ = 6.8 Hz); (*E*)-**53**: δ_F = -163.28 (ddd, $^2J_{HF}$ = 55.0 Hz, $^3J_{HF}$ = 22.3 Hz, $^4J_{HF}$ = 5.4 Hz).

^{13}C NMR (125 MHz, CDCl$_3$) (*Z*)-**53**: δ_C = 179.0 (**C$_1$**), 153.6 (d, $^2J_{CF}$ = 15.0 Hz, **C$_6$**), 103.8 (d, $^3J_{CF}$ = 10.2 Hz, **C$_5$**), 95.0 (d, $^1J_{CF}$ = 177.0 Hz, **C$_7$**), 85.0 (**C$_{13}$**), 83.1 (**C$_9$**), 83.0 (**C$_{14}$**), 78.3 (d, $^4J_{CF}$ = 2.1 Hz, **C$_{11}$**), 63.2 (**C$_{15}$**), 53.9 (d, $^2J_{CF}$ = 22.5 Hz, **C$_8$**), 41.4 (**C$_{10}$**), 40.7 (d, $^3J_{CF}$ = 6.7 Hz, **C$_{12}$**), 38.9 (**C$_{16}$**), 33.5 (**C$_2$**), 31.6 (**C$_{17}$**), 26.0 (SiC(***C***H$_3$)$_3$), 25.9 (SiC(***C***H$_3$)$_3$), 25.2 (**C$_{18}$**), 24.8 (d, $^5J_{CF}$ = 3.7 Hz, **C$_3$**), 24.5 (d, $^4J_{CF}$ = 5.1 Hz, **C$_4$**), 22.7 (**C$_{19}$**), 18.4 (Si***C***(CH$_3$)$_3$), 18.2 (Si***C***(CH$_3$)$_3$), 14.2 (**C$_{20}$**), -4.3 (Si***C***H$_3$), -4.5 (Si***C***H$_3$), -4.7 (Si***C***H$_3$), -4.8 (Si***C***H$_3$).

HRMS (ESI$^+$) calculated for $C_{32}H_{57}FNaO_5Si_2$ [M+Na$^+$] 619.3621, found 619.3613.

5-((3*S*,3a*R*,4*S*,5*R*,6a*S*)-3-fluoro-5-hydroxy-4-((*S*)-3-hydroxyoct-1-ynyl)hexahydro-2*H*-cyclopenta[*b*]furan-2-ylidene)pentanoic acid (4)

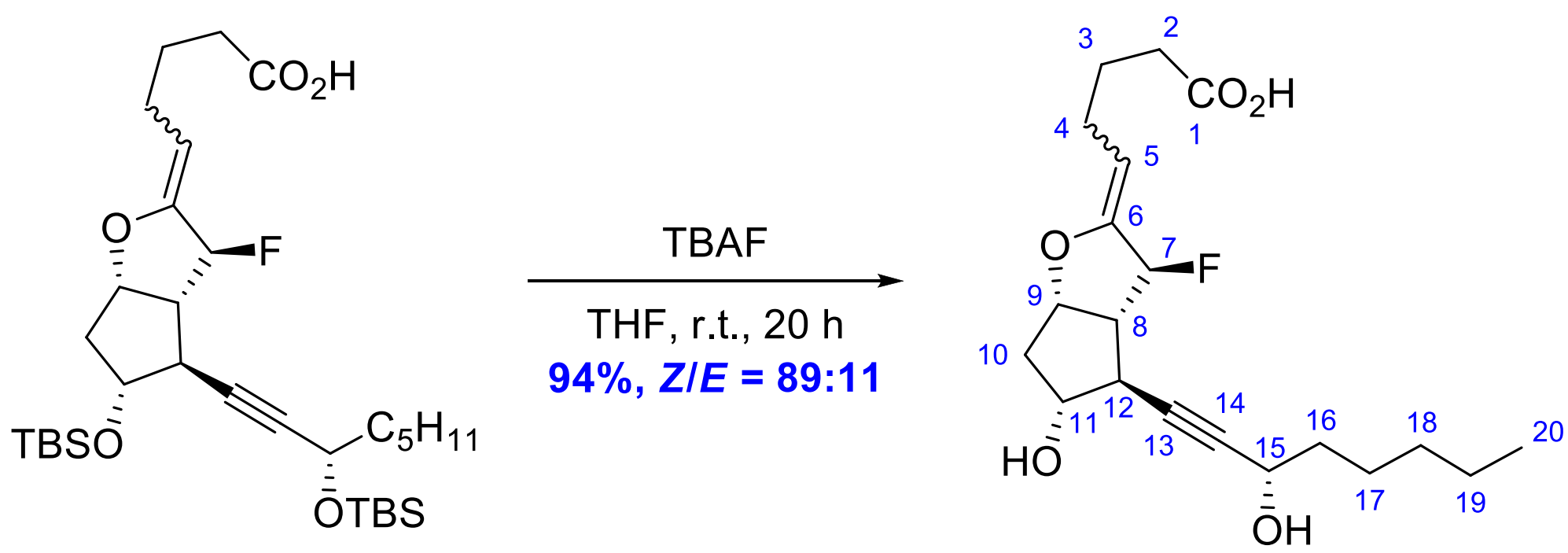

53 **4**

To a stirring solution of **53** (16.8 mg, 28.0 µmol, 1.0 eq.) in THF (0.28 mL) at 0 °C, TBAF (1.0 M in THF, 84.0 µL, 84.0 µmol, 3.0 eq.) was added dropwise. The reaction mixture was warmed to r.t. and stirred for a further 20 h. The reaction was quenched by addition of H2O (1.5 mL) and the aqueous phase was extracted with Et_2O (3 × 3 mL). The combined organic phases were

washed with brine, dried over $MgSO_4$, filtered and concentrated *in vacuo*. The crude product was purified by flash column chromatography on silica gel (CH_2Cl_2/EtOAc/AcOH = 60:39:1 to 40:59:1) to give **4** as a colourless oil (9.70 mg, 26.3 µmol, 94%, 89:11 *Z*/*E*).

$\boldsymbol{R_f}$ = 0.30 (CH_2Cl_2/EtOAc/AcOH = 40:59:1)

IR (thin film) $\boldsymbol{\nu_{max}}$/cm^{-1}: 3341, 2952, 2924, 2855, 1705, 1409, 1375, 1296, 1233, 1135, 1083, 1045, 1025, 958, 913, 780.

^{1}H NMR (500 MHz, $CDCl_3$) (*Z*)-**4**: δ_H = 5.13 (d, J = 55.1 Hz, 1H, ***H***C7), 4.89 (td, J = 6.1, 2.0 Hz, 1H, ***H***C5), 4.76 – 4.71 (m, 1H, ***H***C9), 4.37 (td, J = 6.6, 1.8 Hz, 1H, ***H***C15), 4.17 (q, J = 7.1 Hz, 1H, ***H***C11), 2.76 (ddd, J = 19.1, 9.7, 5.7 Hz, 1H, ***H***C8), 2.50 – 2.44 (m, 1H, ***H***C12), 2.40 – 2.35 (m, 3H, $\boldsymbol{H_2}$C2+$\boldsymbol{H_A}$C10), 2.27 – 2.20 (m, 1H, $\boldsymbol{H_A}$C4), 2.15 – 2.09 (m, 1H, $\boldsymbol{H_B}$C4), 1.88 (ddd, J = 14.9, 6.6, 1.9 Hz, 1H, $\boldsymbol{H_B}$C10), 1.80 (td, J = 14.0, 7.0 Hz, 1H, $\boldsymbol{H_A}$C3), 1.70 – 1.62 (m, 3H, $\boldsymbol{H_B}$C3+$\boldsymbol{H_2}$C16), 1.42 (m, 2H, $\boldsymbol{H_2}$C19), 1.33 – 1.28 (m, 4H, $\boldsymbol{H_2}$C17+$\boldsymbol{H_2}$C18), 0.90 (t, J = 7.0 Hz, 3H, $\boldsymbol{H_3}$C20).

^{19}F NMR (470 MHz, $CDCl_3$) (*Z*)-**4**: δ_F = -158.29 (ddd, $^2J_{HF}$ = 55.1 Hz, $^3J_{HF}$ = 19.0 Hz, $^4J_{HF}$ =6.6 Hz); (*E*)-**4**: δ_F = -163.44 (ddd, $^2J_{HF}$ = 24.9, $^3J_{HF}$ = 20.4 Hz, $^4J_{HF}$ =5.4 Hz).

^{13}C NMR (125 MHz, $CDCl_3$) (*Z*)-**53**: δ_C = 178.6 (**C1**), 153.4 (d, $^2J_{CF}$ = 14.8 Hz, **C6**), 104.8 (d, $^3J_{CF}$ = 10.5 Hz, **C5**), 94.6 (d, $^1J_{CF}$ = 177.2 Hz, **C7**), 84.5 (**C13**), 83.8 (**C9**), 83.6 (**C14**), 78.4 (d, $^4J_{CF}$ = 1.8 Hz, **C11**), 62.7 (**C15**), 54.2 (d, $^2J_{CF}$ = 22.9 Hz, **C8**), 40.6 (d, $^3J_{CF}$ = 7.1 Hz, **C12**), 40.1 (**C10**), 38.0 (**C16**), 33.2 (**C2**), 31.6 (**C17**), 25.1 (**C18**), 24.5 (d, $^5J_{CF}$ = 3.7 Hz, **C3**), 24.2 (d, $^4J_{CF}$ = 5.3 Hz, **C4**), 22.7 (**C19**), 14.1 (**C20**).

HRMS (ESI$^+$) calculated for $C_{20}H_{29}FNaO_5$ [M+Na$^+$] 391.1891, found 391.1883.

(3*S*,3a*R*,4*S*,5*R*,6a*S*)-5-(*tert*-Butyldimethylsilyloxy)-4-((3*S*,4*S*)-3-(*tert*-butyldimethylsilyloxy)-4-methylnona-1,6-diynyl)-3-fluorohexahydro-2*H*-cyclopenta[*b*]furan-2-one (54)

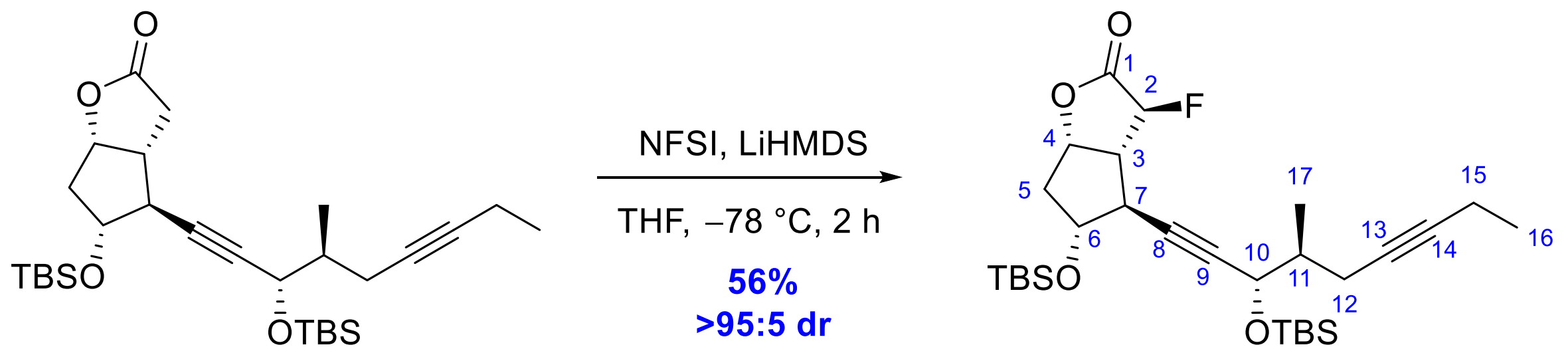

To a solution of **36** (415 mg, 0.80 mmol, 1.0 eq.) and NFSI (315 mg, 1.00 mmol, 1.25 eq.) in THF (4.0 mL) at –78 °C, LiHMDS (1.0 M in THF, 0.92 mL, 0.92 mmol, 1.15 eq.) was added

dropwise. The reaction mixture was stirred at –78 °C for 2 h before it was warmed to r.t., quenched by addition of saturated $NaHCO_3$ solution (8 mL) and the aqueous phase was extracted with Et_2O (3 × 16 mL). The organic phases were combined, washed with brine, dried over $MgSO_4$, filtered and concentrated *in vacuo*. The crude product was purified by flash column chromatography on silica gel (Hexanes/Et_2O = 100:0 to 95:5) to give pure **54** as a colourless oil (240 mg, 0.45 mmol, 56%, >95:5 dr).

$\boldsymbol{R}_\mathbf{f}$ = 0.18 (Hexanes/Et_2O = 95:5)

IR (thin film) $\boldsymbol{\nu}_{\mathbf{max}}$/cm^{-1}: 2955, 2929, 2857, 1792, 1471, 1362, 1328, 1254, 1190, 1091, 1073, 1021, 1001, 887, 834, 775.

^{1}H NMR (400 MHz, $CDCl_3$) δ_H = 5.24 (t, J = 7.2 Hz, 1H, ***H***C4), 5.07 (dd, J = 51.7, 2.7 Hz, 1H, ***H***C2), 4.45 – 4.27 (m, 2H, ***H***C6+***H***C10), 3.21 (dd, J = 31.5, 7.6 Hz, 1H, ***H***C3), 3.07 (s, 1H, ***H***C7), 2.29 – 2.23 (m, 1H, ***H***C11), 2.22 – 2.12 (m, 4H, ***H₂***C12+***H₂***C15), 2.09 (d, J = 15.2 Hz, 1H, $\boldsymbol{H_A}$C5), 1.85 – 1.75 (m, 1H, $\boldsymbol{H_B}$C5), 1.11 (t, J = 7.4 Hz, 3H, $\boldsymbol{H_3}$C16), 1.00 (d, J = 6.8 Hz, 3H, $\boldsymbol{H_3}$C17), 0.90 (s, 9H, SiC(C$\boldsymbol{H_3}$)$_3$), 0.85 (s, 9H, SiC(C$\boldsymbol{H_3}$)$_3$), 0.10 (s, 3H, SiC$\boldsymbol{H_3}$), 0.09 (s, 3H, SiC$\boldsymbol{H_3}$), 0.08 (s, 3H, SiC$\boldsymbol{H_3}$), 0.07 (s, 3H, SiC$\boldsymbol{H_3}$).

^{19}F NMR (377 MHz, $CDCl_3$) δ_F = -176.43 (ddd, $^2J_{HF}$ = 51.6 Hz, $^3J_{HF}$ = 32.9 Hz, $^4J_{HF}$ = 1.0 Hz).

^{13}C NMR (100 MHz, $CDCl_3$) δ_C = 171.7 (d, $^2J_{CF}$ = 21.4 Hz, **C1**), 91.4 (d, $^1J_{CF}$ = 184.4 Hz, **C2**), 85.1 (**C8**), 83.4 (**C14**), 83.1 (d, $^3J_{CF}$ = 3.1 Hz, **C4**), 83.2 (**C9**), 78.6 (**C6**), 77.1 (**C13**), 66.3 (**C10**), 52.1 (d, $^2J_{CF}$ = 21.2 Hz, **C3**), 44.7 (d, $^3J_{CF}$ = 5.4 Hz, **C7**), 40.1 (d, $^4J_{CF}$ = 1.2 Hz, **C5**), 40.1 (**C11**), 25.9 (SiC(***C***H$_3$)$_3$), 25.8 (SiC(***C***H$_3$)$_3$), 22.1 (**C12**), 18.3 (Si***C***(CH$_3$)$_3$), 18.1 (Si***C***(CH$_3$)$_3$), 15.3 (**C17**), 14.5 (**C16**), 12.6 (**C15**), -4.5 (Si***C***H$_3$), -4.9 (Si***C***H$_3$), -5.0 (Si***C***H$_3$), -5.0 (Si***C***H$_3$).

HRMS (ESI$^+$) calculated for $C_{29}H_{49}FNaO_4Si_2$ [M+Na$^+$] 559.3046, found 559.3047.

$[\alpha]_D^{24}$= –30.0 (c 1.0, $CHCl_3$)

5-((3*S*,3a*R*,4*S*,5*R*,6a*S*)-5-(*tert*-Butyldimethylsilyloxy)-4-((3*S*,4*S*)-3-(*tert*-butyldimethylsilyloxy)-4-methylnona-1,6-diynyl)-3-fluorohexahydro-2*H*-cyclopenta[*b*]furan-2-ylidene)pentanoic acid (55)

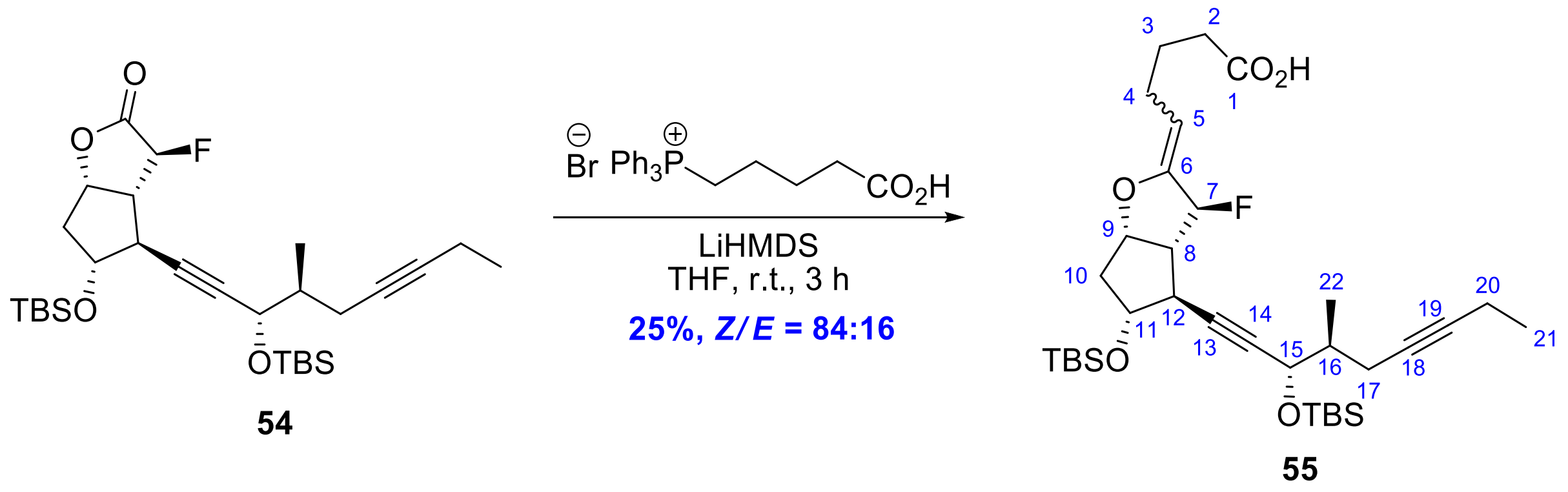

LiHMDS (1.0 M in THF, 0.32 mL, 0.32 mmol, 3.2 eq.) was added to a stirring suspension of (4-carboxybutyl)triphenylphosphonium bromide (66.5 mg, 0.15 mmol, 1.5 eq.) in THF (0.5 mL). The resulting orange suspension was stirred at r.t. for 1 h. **54** (53.7 mg, 0.10 mmol, 1.0 eq.) was added as a solution in THF (0.5 mL) to the orange suspension and the reaction was stirred at r.t. for 3 h. The reaction mixture was quenched by addition of water (10 mL) and extracted with EtOAc (3 × 10 mL). The combined organic phases were washed with water (10 mL), treated with aqueous HCl (0.1 M, 10 mL), washed with brine, dried over $MgSO_4$, filtered and concentrated *in vacuo*. The crude product was purified by flash column chromatography on silica gel (Hexanes/EtOAc = 95:5; then Hexanes/EtOAc/AcOH = 95:4:1) to give **55** as a colourless oil (15.8 mg, 25.4 µmol, 25%, 84:16 *Z*/*E*).

$\boldsymbol{R_f}$ = 0.32 (Hexanes/EtOAc/AcOH = 80:19:1)

IR (thin film) $\boldsymbol{\nu_{max}}$/cm^{-1}: 2955, 2926, 2856, 1710, 1471, 1320, 1251, 1235, 1133, 1075, 1026, 972, 897, 837, 777.

^{1}H NMR (500 MHz, $CDCl_3$) (*Z*)-**55**: δ_H = 5.12 (d, *J* = 55.9 Hz, 1H, ***H***C$_7$), 4.85 (td, *J* = 6.6, 2.3 Hz, 1H, ***H***C$_5$), 4.68 (q, *J* = 7.3 Hz, 1H, ***H***C$_9$), 4.32 (dd, *J* = 6.5, 1.6 Hz, 1H, ***H***C$_{15}$), 4.12 (q, *J* = 7.0 Hz, 1H, ***H***C$_{11}$), 2.73 (ddd, *J* = 20.9, 9.5, 6.2 Hz, 1H, ***H***C$_8$), 2.41 – 2.34 (m, 4H, $\boldsymbol{H_2}$C$_2$+$\boldsymbol{H_A}$C$_{10}$+***H***C$_{12}$), 2.21 – 2.13 (m, 5H, $\boldsymbol{H_A}$C$_4$+ $\boldsymbol{H_2}$C$_{17}$+$\boldsymbol{H_2}$C$_{20}$), 1.85 – 1.75 (m, 3H, $\boldsymbol{H_B}$C$_4$+$\boldsymbol{H_B}$C$_{10}$+***H***C$_{16}$), 1.75 – 1.71 (m, 2H, $\boldsymbol{H_2}$C$_3$), 1.13 (t, *J* = 7.4 Hz, 3H, $\boldsymbol{H_3}$C$_{21}$), 1.03 (d, *J* = 6.8 Hz, 3H, $\boldsymbol{H_3}$C$_{22}$), 0.90 (s, 9H, SiC(C$\boldsymbol{H_3}$)$_3$), 0.87 (s, 9H, SiC(C$\boldsymbol{H_3}$)$_3$), 0.13 (s, 3H, SiC$\boldsymbol{H_3}$), 0.10 (s, 3H, SiC$\boldsymbol{H_3}$), 0.08 (s, 3H, SiC$\boldsymbol{H_3}$), 0.07 (s, 3H, SiC$\boldsymbol{H_3}$).

^{19}F NMR (470 MHz, $CDCl_3$) (*Z*)-**55**: δ_F = -158.25 (ddd, $^2J_{HF}$ = 56.1 Hz, $^3J_{HF}$ = 21.2 Hz, $^4J_{HF}$ = 6.7 Hz, 1H); (*E*)-**55**: δ_F = -163.10 (ddd, $^2J_{HF}$ = 54.8 Hz, $^3J_{HF}$ = 22.2 Hz, $^4J_{HF}$ = 5.3 Hz).

^{13}C NMR (125 MHz, $CDCl_3$) (*Z*)-**55**: δ_C = 178.3 (**C1**), 153.6 (d, $^2J_{CF}$ = 15.0 Hz, **C6**), 103.8 (d, $^3J_{CF}$ = 10.2 Hz, **C5**), 95.0 (d, $^1J_{CF}$ = 177.0 Hz, **C7**), 84.2 (**C13**), 83.3 (**C19**), 83.2 (**C14**), 83.2 (**C9**), 78.3 (d, $^4J_{CF}$ = 2.0 Hz, **C11**), 77.4 (**C18**), 66.4 (**C15**), 54.0 (d, $^2J_{CF}$ = 22.6 Hz, **C8**), 41.5 (**C12**), 40.8 (d, $^4J_{CF}$ = 6.7 Hz, **C10**), 40.2 (**C16**), 33.4 (**C2**), 26.0 (SiC(***C***H3)3), 25.8 (SiC(***C***H3)3), 24.8 (d, $^5J_{CF}$ = 3.7 Hz, **C3**), 24.5 (d, $^4J_{CF}$ = 5.1 Hz, **C4**), 22.1 (**C17**), 18.4 (Si***C***(CH3)3), 18.2 (Si***C***(CH3)3), 15.3 (**C22**), 14.5 (**C21**), 12.6 (**C20**), -4.3 (Si***C***H3), -4.6 (Si***C***H3), -4.6 (Si***C***H3), -5.0 (Si***C***H3).
HRMS (ESI$^+$) calculated for $C_{34}H_{57}FNaO_5Si_2$ [M+Na$^+$] 643.3621, found 643.3617.

5-((3*S*,3a*R*,4*S*,5*R*,6a*S*)-3-Fluoro-5-hydroxy-4-((3*S*,4*S*)-3-hydroxy-4-methylnona-1,6-diynyl)hexahydro-2*H*-cyclopenta[*b*]furan-2-ylidene)pentanoic acid (5)

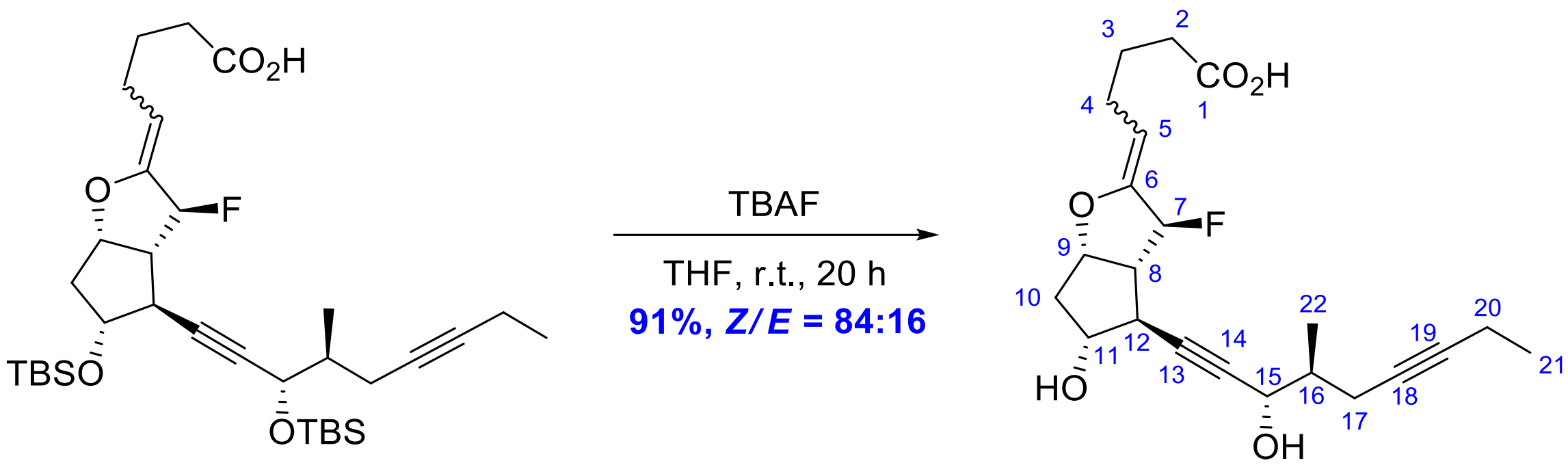

To a stirring solution of **55** (8.00 mg, 12.9 µmol, 1.0 eq.) in THF (0.13 mL) at 0 °C, TBAF (1.0 M in THF, 38.7 µL, 38.7 µmol, 3.0 eq.) was added dropwise. The reaction mixture was warmed to r.t. and stirred for a further 20 h. The reaction was quenched by addition of H2O (1.3 mL) and the aqueous phase was extracted with Et_2O (3 × 2.6 mL). The combined organic phases were washed with brine, dried over $MgSO_4$, filtered and concentrated *in vacuo*. The crude product was purified by flash column chromatography on silica gel (CH_2Cl_2/EtOAc/AcOH = 60:39:1 to 40:59:1) to give **5** as a colourless oil (4.60 mg, 11.7 µmol, 91%, 84:16 *Z*/*E*).

***R*f** = 0.23 (CH_2Cl_2/EtOAc/AcOH = 40:59:1)
IR (thin film) ν_{max}/cm^{-1}: 3356, 2951, 2924, 2853, 1708, 1456, 1375, 1319, 1297, 1234, 1136, 1085, 1022, 978, 818, 799.
^{1}H NMR (500 MHz, $CDCl_3$) (*Z*)-**5**: δ_H = 5.13 (d, *J* = 55.1 Hz, 1H, ***H***C7), 4.90 (td, *J* = 6.1, 1.6 Hz, 1H, ***H***C5), 4.74 (q, *J* = 7.6 Hz, 1H, ***H***C9), 4.39 (dd, *J* = 6.5, 1.7 Hz, 1H, ***H***C15), 4.22 – 4.17 (m, 1H, ***H***C11), 2.82 – 2.75 (m, 1H, ***H***C8), 2.49 – 2.42 (m, 2H, ***HA***C10+***H***C12), 2.38 – 2.35 (m, 2H, ***H2***C2), 2.28 – 2.25 (m, 1H, ***H***C16), 2.19 – 2.09 (m, 4H, ***H2***C17+***H2***C20), 1.93 – 1.87 (m, 2H, ***H2***C4), 1.80 (dd, *J* = 13.9, 6.9 Hz, 1H, ***HB***C10), 1.74 – 1.66 (m, 1H, ***H2***C3), 1.13 (t, *J* = 7.5 Hz, 3H, ***H3***C21), 1.08 (d, *J* = 6.8 Hz, 3H, ***H3***C22).

^{19}F NMR (470 MHz, $CDCl_3$) (*Z*)-**5**: δ_F = -158.05 (ddd, $^2J_{HF}$ = 55.1 Hz, $^3J_{HF}$ =19.0 Hz, $^4J_{HF}$ = 6.4 Hz); (*E*)-**5**: δ_F = -163.25 (ddd, $^2J_{HF}$ = 54.8 Hz, $^3J_{HF}$ = 19.1 Hz, $^4J_{HF}$ = 5.8 Hz).

^{13}C NMR (125 MHz, $CDCl_3$) (*Z*)-**5**: δ_C = 178.2 (**C1**), 153.4 (d, $^2J_{CF}$ = 14.8 Hz, **C6**), 104.8 (d, $^3J_{CF}$ = 10.4 Hz, **C5**), 94.7 (d, $^1J_{CF}$ = 177.3 Hz, **C7**), 85.0 (**C13**), 83.9 (**C19**), 83.8 (**C14**), 82.7 (**C9**), 78.5 (d, $^4J_{CF}$ = 1.5 Hz, **C11**), 77.0 (**C18**), 66.3 (**C15**), 54.4 (d, $^2J_{CF}$ = 22.9 Hz, **C8**), 40.7 (d, $^4J_{CF}$ = 7.1 Hz, **C10**), 40.2 (**C12**), 39.2 (**C16**), 33.1 (**C2**), 24.5 (d, $^5J_{CF}$ = 3.7 Hz, **C3**), 24.2 (d, $^4J_{CF}$ = 5.3 Hz, **C4**), 22.3 (**C17**), 15.4 (**C22**), 14.4 (**C21**), 12.6 (**C20**).

HRMS (ESI^+) calculated for $C_{22}H_{29}FNaO_5$ [$M+Na^+$] 415.1891, found 415.1892.

Synthesis of difluorinated targets *Z*-9 and *E*-9

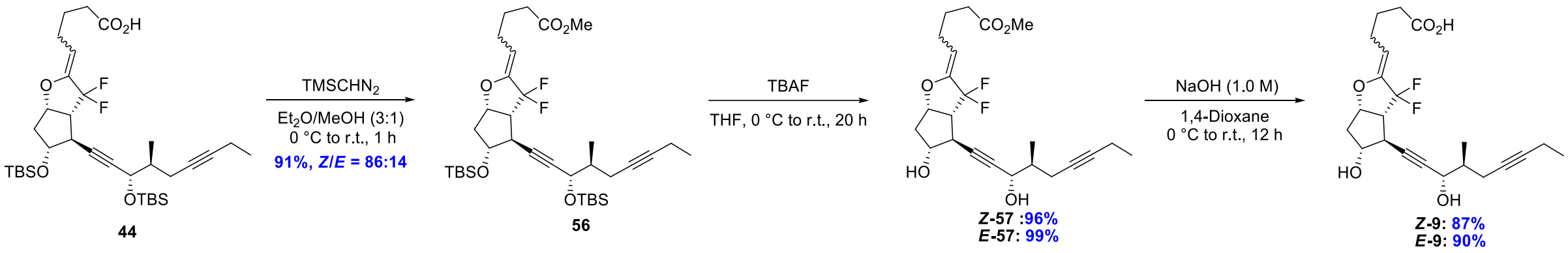

Methyl 5-((3a*R*,4*S*,5*R*,6a*S*)-5-(*tert*-butyldimethylsilyloxy)-4-((3*S*,4*S*)-3-(*tert*-butyldimethylsilyloxy)-4-methylnona-1,6-diynyl)-3,3-difluorohexahydro-2*H*-cyclopenta[*b*]furan-2-ylidene)pentanoate (56)

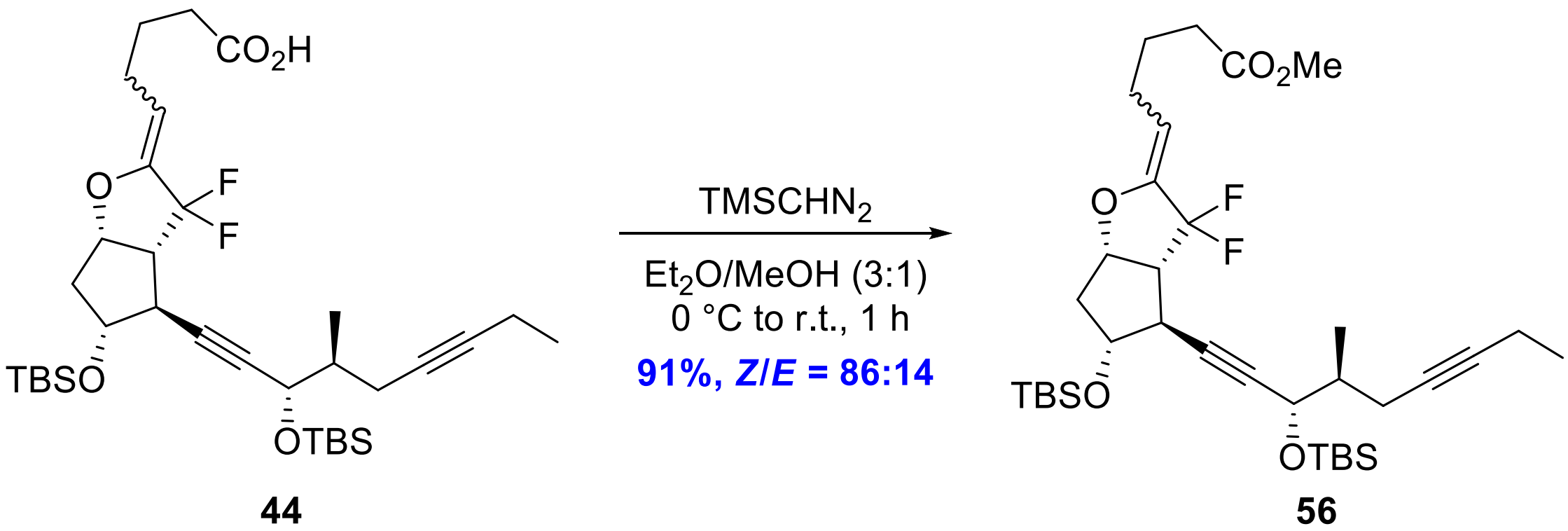

To a stirring solution of **44** (95.5 mg, 149.0 μmol, 1.0 eq.) in Et_2O/MeOH (3.0 mL, 3:1 *v*/*v*) at 0 °C, trimethylsilyldiazomethane (2.0 M in Et_2O, 149 μL, 298 μmol, 2.0 eq.) was added dropwise. The reaction mixture was warmed to r.t. and stirred for 1 h. The reaction was quenched by addition of AcOH until the yellow solution turned into colourless solution, and the solvents were removed in *vacuo*. The crude product was purified by flash column chromatography on silica gel (Hexanes/EtOAc = 100:0 to 90:10) to give **56** as a colourless oil (89.0 mg, 136 μmol, 91%, 86:14 *Z*/*E*). The mixture of *Z*/*E* isomers were purified by chiral HPLC (Chiralpak® OD-3 column; flow rate 1.0 mL/min, Hexanes/*i*-PrOH = 99:1; retention time: $t_{Z\text{-isomer}}$ = 3.2 min, $t_{E\text{-isomer}}$ = 3.6 min) to give pure *Z*-isomer and *E*-isomer.

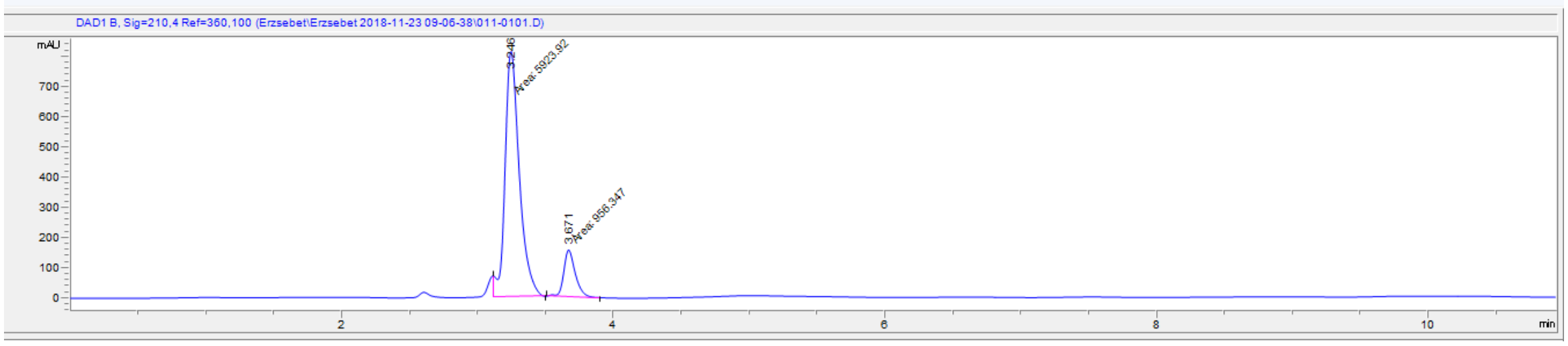

#	Time	Area	Height	Width	Area%	Symmetry
1	3.246	5923.9	814.4	0.1212	86.100	0.693
2	3.671	956.3	156.6	0.1018	13.900	0.687

(*Z*)-Methyl 5-((3a*R*,4*S*,5*R*,6a*S*)-5-(*tert*-butyldimethylsilyloxy)-4-((3*S*,4*S*)-3-(*tert*-butyldimethylsilyloxy)-4-methylnona-1,6-diynyl)-3,3-difluorohexahydro-2*H*-cyclopenta[*b*]furan-2-ylidene)pentanoate (*Z*-56)

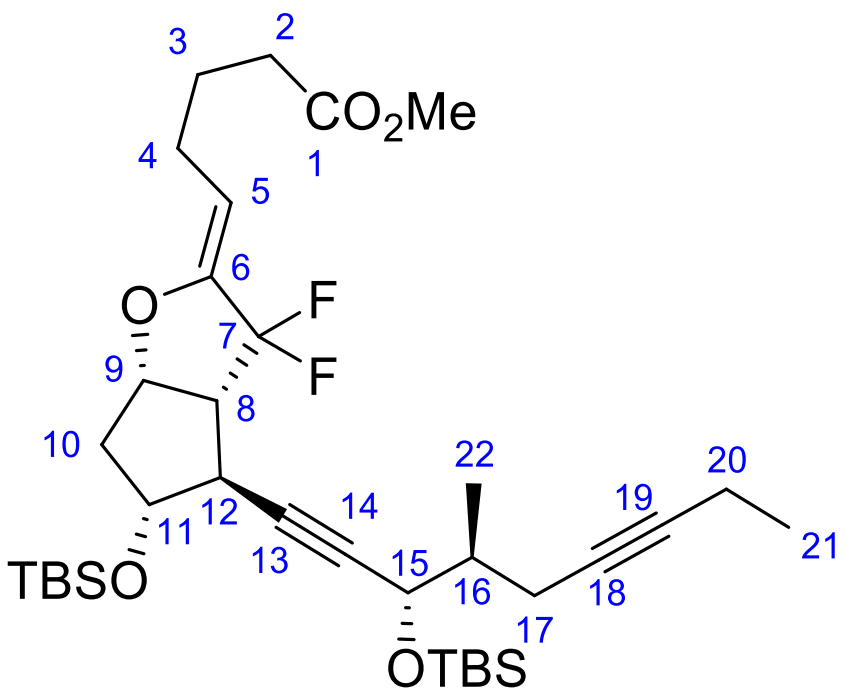

***Z*-56**

***R*f** = 0.48 (Hexanes/EtOAc = 90:10)

IR (thin film) ν_{max}/cm^{-1}: 2956, 2928, 2857, 1741, 1462, 1378, 1294, 1250, 1194, 1075, 1043, 1031, 837, 778.

^{1}H NMR (500 MHz, $CDCl_3$) δ_H = 4.83 – 4.79 (m, 2H, HC_5+HC_9), 4.30 (dd, J = 6.5, 1.3 Hz, 1H, HC_{15}), 4.16 (q, J = 6.3 Hz, 1H, HC_{11}), 3.66 (s, 3H, CO_2CH_3), 2.91 – 2.83 (m, 2H, HC_8+HC_{12}), 2.39 – 2.30 (m, 3H, H_2C_2+H_AC_{10}), 2.27 – 2.05 (m, 6H, H_2C_4+H_2C_{17}+H_2C_{20}), 1.88 – 1.77 (m, 2H, H_BC_{10}+HC_{16}), 1.75 – 1.69 (m, 2H, H_2C_3), 1.11 (t, J = 7.5 Hz, 3H, H_3C_{21}), 1.03 (d, J = 6.8 Hz, 3H, H_3C_{22}), 0.89 (s, 9H, $SiC(CH_3)_3$), 0.87 (s, 9H, $SiC(CH_3)_3$), 0.11 (s, 3H, $SiCH_3$), 0.09 (s, 3H, $SiCH_3$), 0.08 (s, 3H, $SiCH_3$), 0.06 (s, 3H, $SiCH_3$).

^{19}F NMR (377 MHz, $CDCl_3$) δ_F = -82.69 (ddd, $^2J_{FF}$ = 252.5 Hz, $^3J_{HF}$ = 16.8 Hz, $^4J_{HF}$ = 4.6 Hz), -112.30 (d, $^2J_{FF}$ = 252.5 Hz).

^{13}C NMR (125 MHz, $CDCl_3$) δ_C = 174.1 (**C$_1$**), 149.3 (dd, $^2J_{CF}$ = 30.0 Hz, $^2J_{CF}$ = 25.8 Hz, **C$_6$**), 123.6 (dd, $^1J_{CF}$ = 255.4 Hz, $^1J_{CF}$ = 240.6 Hz, **C$_7$**), 100.3 (d, $^3J_{CF}$ = 4.4 Hz, **C$_5$**), 84.2 (**C$_{13}$**), 83.2 (**C$_{19}$**), 83.0 (**C$_{14}$**), 82.0 (dd, $^3J_{CF}$ = 5.0 Hz, $^3J_{CF}$ = 1.0 Hz, **C$_9$**), 78.8 (d, $^4J_{CF}$ = 1.9 Hz, **C$_{11}$**), 77.5

(**C18**), 66.4 (**C15**), 53.9 (dd, $^2J_{CF}$ = 27.2 Hz, $^2J_{CF}$ =19.5 Hz, **C8**), 51.6 (CO_2*C*H_3), 41.8 (**C10**), 40.2 (**C16**), 39.9 (dd, $^3J_{CF}$ = 4.6 Hz, $^3J_{CF}$ = 2.0 Hz, **C12**), 33.6 (**C2**), 25.9 (SiC(*C*H_3)$_3$), 25.8 (SiC(*C*H_3)$_3$), 24.6 (d, $^5J_{CF}$ = 2.9 Hz, **C3**), 24.2 (d, $^4J_{CF}$ = 1.4 Hz, **C4**), 22.0 (**C17**), 18.3 (Si***C***(CH_3)$_3$), 18.1 (Si***C***(CH_3)$_3$), 15.3 (**C22**), 14.5 (**C21**), 12.6 (**C20**), -4.4 (Si*C*H_3), -4.6 (Si*C*H_3), -4.7 (Si*C*H_3), -5.1 (Si*C*H_3).

HRMS ($MALDI^+$) calculated for $C_{35}H_{58}F_2NaO_5Si_2$ [$M+Na^+$] 675.3683, found 675.3692.

[*α*]$_D$^{26} = +28.4 (*c* 0.74, $CHCl_3$)

(*E*)-Methyl 5-((3a*R*,4*S*,5*R*,6a*S*)-5-(*tert*-butyldimethylsilyloxy)-4-((3*S*,4*S*)-3-(*tert*-butyldimethylsilyloxy)-4-methylnona-1,6-diynyl)-3,3-difluorohexahydro-2*H*-cyclopenta[*b*]furan-2-ylidene)pentanoate (*E*-56)

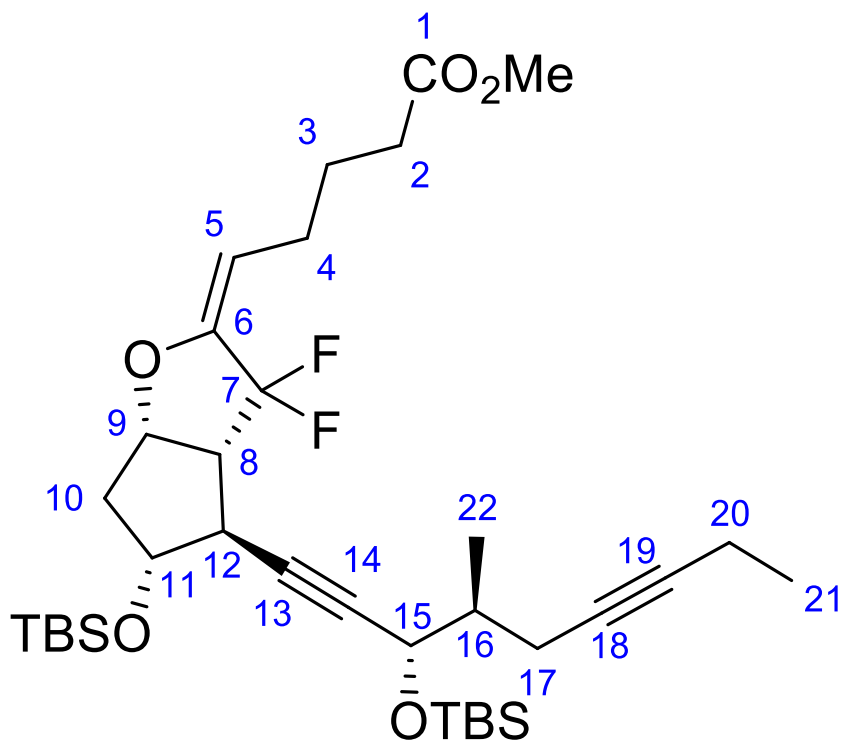

***E*-56**

***R*f** = 0.48 (Hexanes/EtOAc = 90:10)

IR (thin film) ***ν*max**/cm^{-1}: 2954, 2924, 2854, 1743, 1462, 1377, 1251, 1189, 1079, 1047, 837, 771.

^{1}H NMR (500 MHz, $CDCl_3$) δ_H = 4.96 – 4.92 (m, 1H, ***H***C5), 4.75 (td, *J* = 6.4, 2.2 Hz, 1H, ***H***C9), 4.30 (dd, *J* = 6.5, 1.5 Hz, 1H, ***H***C15), 4.18 (q, *J* = 5.9 Hz, 1H, ***H***C11), 3.66 (s, 3H, CO_2C***H3***), 2.99 – 2.89 (m, 2H, ***H***C8+***H***C12), 2.36 – 2.27 (m, 3H, ***H2***C2+***HA***C10), 2.25 – 2.13 (m, 6H, ***H2***C4+***H2***C17+***H2***C20), 1.89 – 1.79 (m, 2H, ***HB***C10+***H***C16), 1.75 – 1.65 (m, 2H, ***H2***C3), 1.11 (t, *J* = 7.5 Hz, 3H, ***H3***C21), 1.03 (d, *J* = 6.8 Hz, 3H, ***H3***C22), 0.90 (s, 9H, SiC(C***H3***)**3**), 0.87 (s, 9H, SiC(C***H3***)**3**), 0.12 (s, 3H, SiC***H3***), 0.10 (s, 3H, SiC***H3***), 0.08 (s, 3H, SiC***H3***), 0.06 (s, 3H, SiC***H3***).

^{19}F NMR (377 MHz, $CDCl_3$) δ_F = -84.25 (ddd, $^2J_{FF}$ = 260.1 Hz, $^3J_{HF}$ = 18.8 Hz, $^4J_{HF}$ = 3.3 Hz), -106.22 (d, $^2J_{FF}$ = 260.1 Hz).

^{13}C NMR (125 MHz, $CDCl_3$) δ_C = 174.2 (**C1**), 149.5 (dd, $^2J_{CF}$ = 28.3 Hz, $^2J_{CF}$ = 26.6 Hz, **C6**), 125.0 (dd, $^1J_{CF}$ =255.7 Hz, $^1J_{CF}$ = 242.2 Hz, **C7**), 103.2 (d, $^3J_{CF}$ = 3.3 Hz, **C5**), 84.4 (**C13**), 83.2

(**C**$_{19}$), 83.1 (**C**$_{14}$), 81.6 (dd, $^3J_{CF}$ = 4.0 Hz, $^3J_{CF}$ = 2.1 Hz, **C**$_9$), 78.8 (d, $^4J_{CF}$ = 1.6 Hz, **C**$_{11}$), 77.5 (**C**$_{18}$), 66.4 (**C**$_{15}$), 55.4 (dd, $^2J_{CF}$ = 26.4 Hz, $^2J_{CF}$ = 20.3 Hz, **C**$_8$), 51.6 ($CO_2\boldsymbol{C}H_3$), 41.5 (**C**$_{10}$), 40.2 (**C**$_{16}$), 39.9 (dd, $^3J_{CF}$ = 4.0 Hz, $^3J_{CF}$ = 4.0 Hz, **C**$_{12}$), 33.5 (**C**$_2$), 25.9 ($SiC(\boldsymbol{C}H_3)_3$), 25.8 ($SiC(\boldsymbol{C}H_3)_3$), 25.5 (d, $^5J_{CF}$ = 1.8 Hz, **C**$_3$), 23.7 (**C**$_4$), 22.0 (**C**$_{17}$), 18.3 ($Si\boldsymbol{C}(CH_3)_3$), 18.1 ($Si\boldsymbol{C}(CH_3)_3$), 15.3 (**C**$_{22}$), 14.5 (**C**$_{21}$), 12.6 (**C**$_{20}$), -4.4 ($Si\boldsymbol{C}H_3$), -4.7 ($Si\boldsymbol{C}H_3$), -4.7 ($Si\boldsymbol{C}H_3$), -5.0 ($Si\boldsymbol{C}H_3$).

HRMS ($MALDI^+$) calculated for $C_{35}H_{58}F_2NaO_5Si_2$ [$M+Na^+$] 675.3683, found 675.3692.

[*α*]$_D^{25}$ = –12.8 (*c* 0.44, $CHCl_3$)

(*Z*)-Methyl 5-((3a*R*,4*S*,5*R*,6a*S*)-3,3-difluoro-5-hydroxy-4-((3*S*,4*S*)-3-hydroxy-4-methylnona-1,6-diynyl)hexahydro-2*H*-cyclopenta[*b*]furan-2-ylidene)pentanoate (*Z*-57)

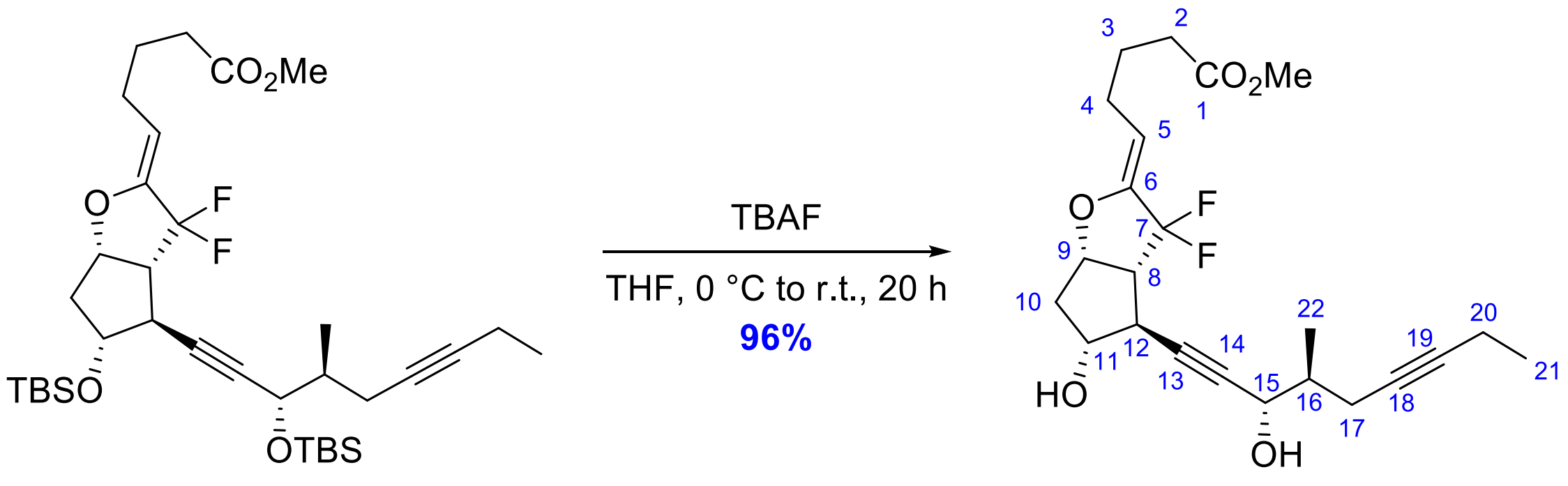

To a stirring solution of ***Z*-56** (29.5 mg, 45.2 μmol, 1.0 eq.) in THF (0.45 mL) at 0 °C, TBAF (1.0 M in THF, 136 μL, 136 μmol, 3.0 eq.) was added dropwise. The reaction mixture was warmed to r.t. and stirred for a further 20 h. The reaction was quenched by addition of H_2O (4.5 mL) and the aqueous phase was extracted with EtOAc (3 × 9.0 mL). The combined organic phases were washed with brine, dried over $MgSO_4$, filtered and concentrated *in vacuo*. The crude product was purified by flash column chromatography on silica gel (Hexanes/EtOAc = 70:30 to 40:60) to give ***Z*-57** as a colourless oil (18.4 mg, 43.4 μmol, 96%).

***R*$_f$** = 0.30 (Hexanes/EtOAc = 50:50)

IR (thin film) $\boldsymbol{\nu}_{max}$/cm^{-1}: 3415, 2953, 2924, 2871, 2854, 1736, 1717, 1455, 1439, 1376, 1300, 1243, 1197, 1168, 1103, 1074, 1042, 1018, 967, 765.

^{1}H NMR (500 MHz, $CDCl_3$) δ_H = 4.86 – 4.82 (m, 2H, ***H***C$_5$+***H***C$_9$), 4.36 (d, *J* = 6.1 Hz, 1H, ***H***C$_{15}$), 4.22 (d, *J* = 4.9 Hz, 1H, ***H***C$_{11}$), 3.66 (s, 3H, $CO_2C\boldsymbol{H_3}$), 3.31 (s, 1H, O***H***), 2.93 – 2.86 (m, 2H, ***H***C$_8$+***H***C$_{12}$), 2.74 (s, 1H, O***H***), 2.49 – 2.43 (m, 1H, ***H$_A$***C$_{10}$), 2.31 (t, *J* = 7.3 Hz, 2H, ***H$_2$***C$_2$), 2.24 (dd, *J* = 5.7, 2.2 Hz, 2H, ***H$_2$***C$_{17}$), 2.17 – 2.06 (m, 4H, ***H$_2$***C$_4$+***H$_2$***C$_{20}$), 1.95 (dd, *J* =

14.9, 5.9 Hz, 1H, H_BC10), 1.92 – 1.85 (m, 1H, ***H***C16), 1.79 – 1.66 (m, 2H, H_2C3), 1.11 (t, J = 7.5 Hz, 3H, H_3C21), 1.06 (d, J = 6.8 Hz, 3H, H_3C22).

^{19}F NMR (377 MHz, CDCl$_3$) δ_F = -84.17 (ddd, $^2J_{FF}$ = 252.6 Hz, $^3J_{HF}$ = 15.4 Hz, $^4J_{HF}$ = 4.2 Hz), -114.13 (d, $^2J_{FF}$ = 252.5 Hz).

^{13}C NMR (125 MHz, CDCl$_3$) δ_C = 174.4 (**C1**), 148.9 (dd, $^2J_{CF}$ = 30.0 Hz, $^2J_{CF}$ =25.6 Hz, **C6**), 123.5 (dd, $^1J_{CF}$ = 254.8 Hz, $^1J_{CF}$ = 241.4 Hz, **C7**), 101.0 (d, $^3J_{CF}$ = 4.5 Hz, **C5**), 85.0 (**C13**), 83.8 (**C19**), 82.3 (**C14**), 82.1 (d, $^3J_{CF}$ = 5.3 Hz, **C9**), 78.6 (d, $^4J_{CF}$ = 1.5 Hz, **C11**), 77.0 (**C18**), 66.2 (**C15**), 54.0 (dd, $^2J_{CF}$ = 27.1 Hz, $^2J_{CF}$ = 19.5 Hz, **C8**), 51.8 (CO$_2$***C***H$_3$), 40.4 (**C10**), 39.7 (dd, $^3J_{CF}$ = 4.9 Hz, $^3J_{CF}$ = 1.3 Hz, **C12**), 39.4 (**C16**), 33.5 (**C2**), 24.4 (d, $^5J_{CF}$ = 3.0 Hz, **C3**), 23.9 (d, $^4J_{CF}$ = 1.7 Hz, **C4**), 22.3 (**C17**), 15.2 (**C22**), 14.4 (**C21**), 12.5 (**C20**).

HRMS (MALDI$^+$) calculated for $C_{23}H_{30}F_2NaO_5$ [M+Na$^+$] 447.1954, found 447.1965.

$[\alpha]_D^{26}$ = +70.6 (c 0.34, CHCl$_3$)

(*E*)-Methyl 5-((3a*R*,4*S*,5*R*,6a*S*)-3,3-difluoro-5-hydroxy-4-((3*S*,4*S*)-3-hydroxy-4-methylnona-1,6-diynyl)hexahydro-2*H*-cyclopenta[*b*]furan-2-ylidene)pentanoate (*E*-57)

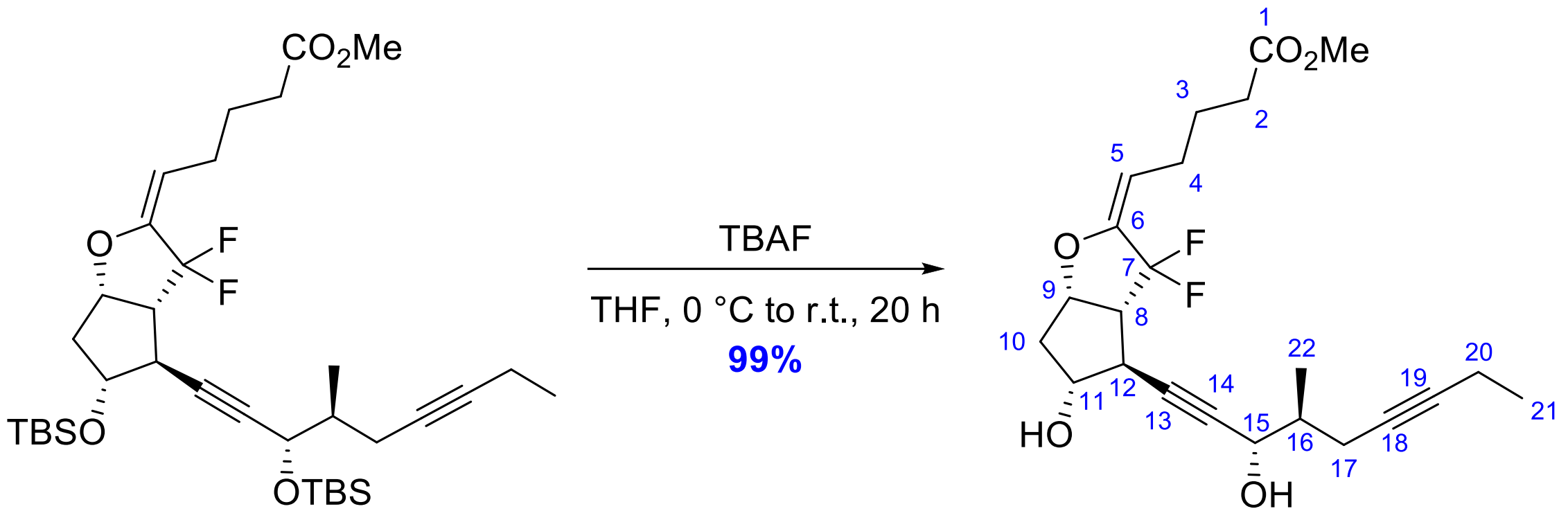

***E*-56** ***E*-57**

To a stirring solution of ***E*-56** (13.5 mg, 20.7 µmol, 1.0 eq.) in THF (0.21 mL) at 0 °C, TBAF (1.0 M in THF, 62.1 µL, 62.1 µmol, 3.0 eq.) was added dropwise. The reaction mixture was warmed to r.t. and stirred for a further 20 h. The reaction was quenched by addition of H_2O (3.0 mL) and the aqueous phase was extracted with EtOAc (3 × 3.0 mL). The combined organic phases were washed with brine, dried over MgSO$_4$, filtered and concentrated *in vacuo*. The crude product was purified by flash column chromatography on silica gel (Hexanes/EtOAc = 70:30 to 40:60) to give ***E*-57** as a colourless oil (8.70 mg, 20.5 µmol, 99%).

***R*f** = 0.30 (Hexanes/EtOAc = 50:50)

IR (thin film) ν_{max}/cm^{-1}: 3417, 2974, 2922, 2875, 2851, 1735, 1715, 1456, 1437, 1374, 1316, 1247, 1227, 1197, 1145, 1112, 1080, 1043, 892, 758.

^{1}H NMR (500 MHz, $CDCl_3$) δ_H = 5.05 – 5.00 (m, 1H, ***H***C5), 4.79 (td, *J* = 6.0, 2.1 Hz, 1H, ***H***C9), 4.38 (d, *J* = 4.5 Hz, 1H, ***H***C15), 4.30 – 4.23 (m, 1H, ***H***C11), 3.67 (s, 3H, CO_2CH_3), 3.05 – 3.02 (m, 1H, ***H***C12), 3.00 – 2.94 (m, 1H, ***H***C8), 2.57 (s, 1H, O***H***), 2.42 – 2.13 (m, 9H, H_2C_2+H_2C_4+H_AC_{10}+H_2C_{17}+H_2C_{20}), 1.99 (dd, *J* = 14.9, 5.3 Hz, 1H, H_BC_{10}), 1.92 – 1.87 (m, 1H, ***H***C16), 1.74 – 1.68 (m, 2H, H_2C_3), 1.12 (t, *J* = 7.5 Hz, 3H, H_3C_{21}), 1.07 (d, *J* = 6.8 Hz, 3H, H_3C_{22}).

^{19}F NMR (377 MHz, $CDCl_3$) δ_F = -85.44 (ddd, $^2J_{FF}$ = 260.7 Hz, $^3J_{HF}$ = 18.1 Hz, $^4J_{HF}$ = 3.3 Hz), -106.87 (d, $^2J_{FF}$ = 260.7 Hz).

^{13}C NMR (125 MHz, $CDCl_3$) δ_C = 174.2 (**C1**), 148.9 (dd, $^2J_{CF}$ = 28.1 Hz, $^2J_{CF}$ = 26.6 Hz, **C6**), 124.9 (dd, $^1J_{CF}$ = 255.0 Hz, $^1J_{CF}$ = 243.3 Hz, **C7**), 104.5 (d, $^3J_{CF}$ = 3.3 Hz, **C5**), 85.1 (**C13**), 83.9 (**C19**), 82.6 (**C14**), 82.0 (dd, $^3J_{CF}$ = 4.4 Hz, $^3J_{CF}$ = 1.7 Hz, **C9**), 78.9 (d, $^4J_{CF}$ = 1.4 Hz, **C11**), 77.0 (**C18**), 66.3 (**C15**), 55.9 (dd, $^2J_{CF}$ = 26.2 Hz, $^2J_{CF}$ = 20.5 Hz, **C8**), 51.7 (CO_2CH_3), 40.2 (**C10**), 40.0 (dd, $^3J_{CF}$ = 4.0 Hz, $^3J_{CF}$ = 4.0 Hz, **C12**), 39.3 (**C16**), 33.3 (**C2**), 25.3 (d, $^5J_{CF}$ = 1.7 Hz, **C3**), 23.6 (d, $^4J_{CF}$ = 1.3 Hz, **C4**), 22.3 (**C17**), 15.3 (**C22**), 14.4 (**C21**), 12.6 (**C20**).

HRMS (ESI^+) calculated for $C_{23}H_{30}F_2NaO_5$ [M+Na^+] 447.1954, found 447.1962.

$[\alpha]_D^{26}$ = +22.5 (*c* 0.33, $CHCl_3$)

(*Z*)-5-((3a*R*,4*S*,5*R*,6a*S*)-3,3-Difluoro-5-hydroxy-4-((3*S*,4*S*)-3-hydroxy-4-methylnona-1,6-diynyl)hexahydro-2*H*-cyclopenta[*b*]furan-2-ylidene)pentanoic acid (*Z*-9)

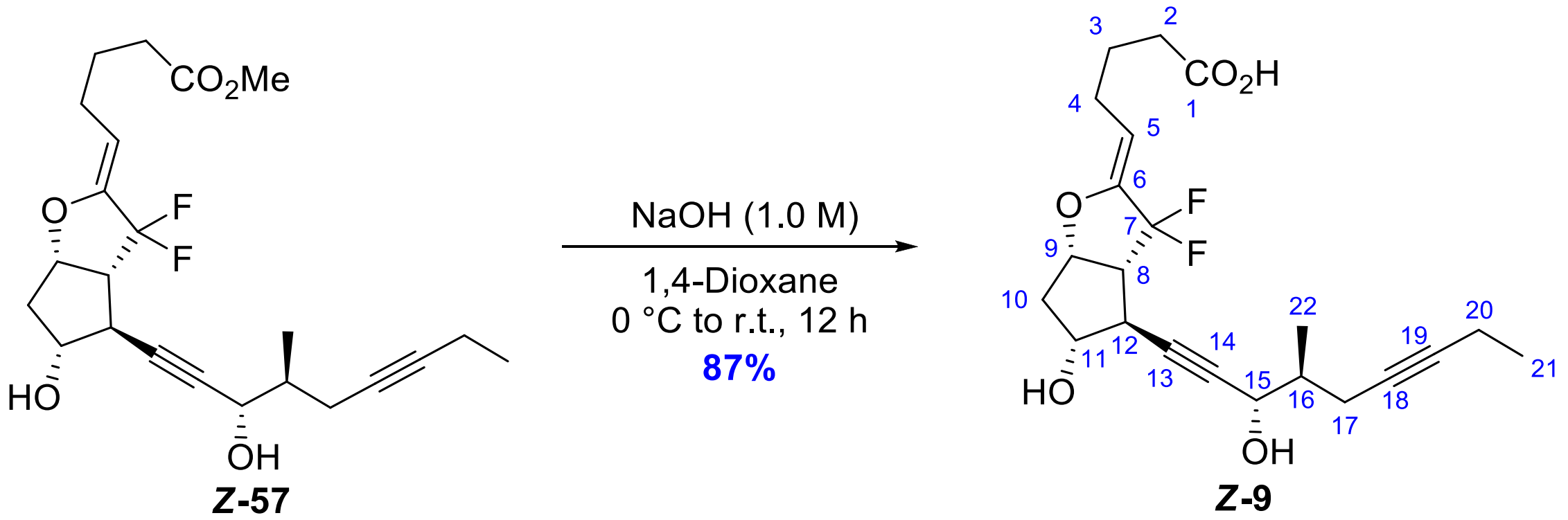

To a stirring solution of ***Z*-57** (26.4 mg, 62.2 µmol, 1.0 eq.) in 1,4-dioxane (6.22 mL) at 0 °C, NaOH (1.0 M aq., 6.22 mL, 6.22 mmol, 100.0 eq.) was added slowly. The reaction mixture was warmed to r.t. and stirred for a further 12 h. The reaction mixture was acidified with 0.1 N HCl to pH = 3 at 0 °C, and the crude mixture was extracted with EtOAc (3 × 30.0 mL). The combined organic phases were washed with brine, dried over $MgSO_4$, filtered and concentrated *in vacuo*. The crude product was purified by flash column chromatography on silica gel

(CH_2Cl_2/EtOAc/AcOH = 60:39:1 to 40:59:1) to give ***Z*-9** as a colourless oil (22.3 mg, 54.3 µmol, 87%).

***R*f** = 0.31 (CH_2Cl_2/EtOAc/AcOH = 40:59:1)

IR (thin film) ν_{max}/cm^{-1}: 3357, 2963, 2924, 2875, 2854, 1708, 1455, 1409, 1377, 1323, 1299, 1241, 1197, 1073, 1042, 1018, 963, 758.

^{1}H NMR (500 MHz, $CDCl_3$) δ_H = 5.46 (overlapping, br.s+br.s, 2H, O***H***+O***H***), 4.87 – 4.81 (m, 2H, ***H***C5+***H***C9), 4.37 (d, *J* = 6.4 Hz, 1H, ***H***C15), 4.22 (d, *J* = 5.1 Hz, 1H, ***H***C11), 2.93 – 2.85 (m, 2H, ***H***C8+***H***C12), 2.51 – 2.45 (m, 1H, ***H$_A$***C10), 2.42 – 2.30 (m, 2H, ***H$_2$***C2), 2.25 –2.21 (m, 2H, ***H$_2$***C17), 2.20 – 2.08 (m, 4H, ***H$_2$***C4+***H$_2$***C20), 1.96 – 1.86 (m, 2H, ***H$_B$***C10+***H***C16), 1.82 – 1.81 – 1.76 (m, 1H, ***H$_A$***C3), 1.74 – 1.65 (m, 1H, ***H$_B$***C3), 1.11 (t, *J* = 7.5 Hz, 3H, ***H$_3$***C21), 1.06 (d, *J* = 6.8 Hz, 3H, ***H$_3$***C22).

^{19}F NMR (377 MHz, $CDCl_3$) δ_F = -84.30 (ddd, $^2J_{FF}$ = 252.7 Hz, $^3J_{HF}$ =15.4, Hz, $^4J_{HF}$ = 3.2 Hz), -114.36 (d, $^2J_{FF}$ = 252.7 Hz).

^{13}C NMR (125 MHz, $CDCl_3$) δ_C = 179.0 (**C1**), 149.0 (dd, $^2J_{CF}$ = 30.0 Hz, $^2J_{CF}$ = 25.5 Hz, **C6**), 123.5 (dd, $^1J_{CF}$ = 254.9 Hz, $^1J_{CF}$ = 241.5 Hz, **C7**), 101.0 (d, $^3J_{CF}$ = 4.4 Hz, **C5**), 85.0 (**C13**), 83.8 (**C19**), 82.3 (**C14**), 82.0 (d, $^3J_{CF}$ = 5.3 Hz, **C9**), 78.5 (**C11**), 77.0 (**C18**), 66.2 (**C15**), 54.0 (dd, $^2J_{CF}$ = 27.2 Hz, $^2J_{CF}$ =19.5 Hz, **C8**), 40.3 (**C10**), 39.6 (d, $^3J_{CF}$ = 4.1 Hz, **C12**), 39.3 (**C16**), 33.2 (**C2**), 24.0 (**C3**), 23.7 (**C4**), 22.3 (**C17**), 15.2 (**C22**), 14.4 (**C21**), 12.6 (**C20**).

HRMS (ESI$^+$) calculated for $C_{22}H_{28}F_2NaO_5$ [M+Na$^+$] 433.1797, found 433.1804.

[*α*]D^{27} = +60.1 (*c* 0.97, $CHCl_3$)

(*E*)-5-((3a*R*,4*S*,5*R*,6a*S*)-3,3-Difluoro-5-hydroxy-4-((3*S*,4*S*)-3-hydroxy-4-methylnona-1,6-diynyl)hexahydro-2*H*-cyclopenta[*b*]furan-2-ylidene)pentanoic acid (*E*-9)

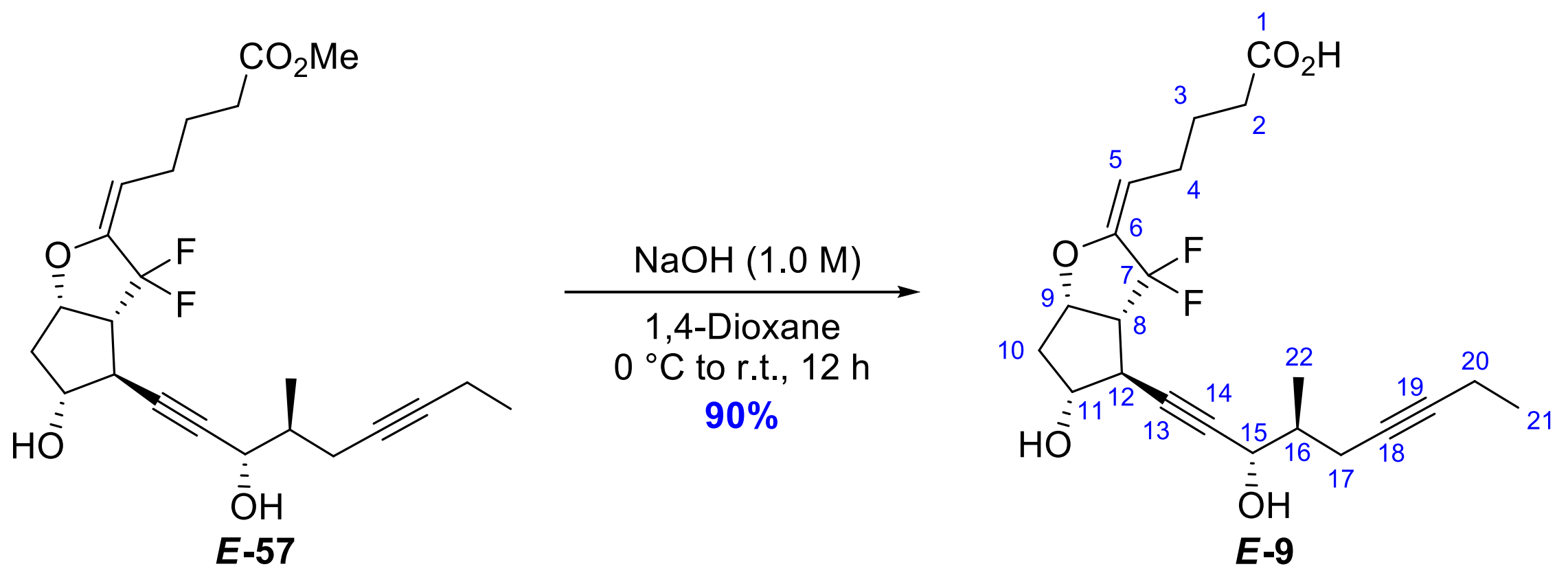

To a stirring solution of ***E*-57** (7.70 mg, 18.1 µmol, 1.0 eq.) in 1,4-dioxane (1.81 mL) at 0 °C, NaOH (1.0 M aq., 1.81 mL, 1.81 mmol, 100.0 eq.) was added slowly. The reaction mixture

was warmed to r.t. and stirred for a further 12 h. The reaction mixture was acidified with 0.1 N HCl to pH = 3 at 0 °C, and the aqueous phase was extracted with EtOAc (3 × 15.0 mL). The combined organic phases were washed with brine, dried over $MgSO_4$, filtered and concentrated *in vacuo*. The crude product was purified by flash column chromatography on silica gel (CH_2Cl_2/EtOAc/AcOH = 60:39:1 to 40:59:1) to give ***E*-9** as a colourless oil (6.70 mg, 16.3 μmol, 90%).

IR (thin film) ν_{max}/cm^{-1}: 3417, 2974, 2922, 2875, 2851, 1735, 1715, 1456, 1437, 1374, 1316, 1247, 1227, 1197, 1145, 1112, 1080, 1043, 892, 758.

^{1}H NMR (400 MHz, $CDCl_3$) δ_H = 5.05 – 5.01 (m, 1H, HC_5), 4.81 – 4.78 (m, 1H, HC_9), 4.39 (dd, J = 6.1, 0.8 Hz, 1H, HC_{15}), 4.27 (dd, J = 11.5, 5.6 Hz, 1H, HC_{11}), 3.07 – 2.94 (m, 2H, HC_8+HC_{12}), 2.43 – 2.35 (m, 3H, H_2C_2+H_AC_{10}), 2.31 – 2.14 (m, 6H, H_2C_4+H_2C_{17}+H_2C_{20}), 2.00 (dd, J = 15.0, 4.5 Hz, 1H, H_BC_{10}), 1.93 – 1. 87 (m, 1H, HC_{16}), 1.79 – 1.68 (m, 2H, H_2C_3), 1.12 (t, J = 7.5 Hz, 3H, H_3C_{21}), 1.07 (d, J = 6.8 Hz, 3H, H_3C_{22}).

^{19}F NMR (377 MHz, $CDCl_3$) δ_F = -85.17 (ddd, $^2J_{FF}$ = 260.6 Hz, $^3J_{HF}$ = 18.3 Hz, $^4J_{HF}$ = 2.9 Hz), -106.98 (d, $^2J_{FF}$ = 260.6 Hz).

^{13}C NMR (125 MHz, $CDCl_3$) δ_C = 178.5 (**C_1**), 148.9 (dd, $^2J_{CF}$ = 27.3 Hz, $^2J_{CF}$ = 27.3 Hz, **C_6**), 124.9 (dd, $^1J_{CF}$ = 255.0 Hz, $^1J_{CF}$ = 243.2 Hz, **C_7**), 104.3 (d, $^3J_{CF}$ = 3.1 Hz, **C_5**), 85.1 (**C_{13}**), 83.9 (**C_{19}**), 82.5 (**C_{14}**), 82.0 (d, $^3J_{CF}$ = 3.1 Hz, **C_9**), 78.8 (**C_{11}**), 77.0 (**C_{18}**), 66.3 (**C_{15}**), 55.8 (dd, $^2J_{CF}$ = 26.2 Hz, $^2J_{CF}$ = 20.5 Hz, **C_8**), 40.2 (**C_{10}**), 39.9 (dd, $^3J_{CF}$ = 3.6 Hz, $^3J_{CF}$ = 3.6 Hz, **C_{12}**), 39.3 (**C_{16}**), 33.0 (**C_2**), 25.0 (**C_3**), 23.5 (**C_4**), 22.3 (**C_{17}**), 15.3 (**C_{22}**), 14.4 (**C_{21}**), 12.6 (**C_{20}**).

HRMS (ESI$^+$) calculated for $C_{22}H_{28}F_2NaO_5$ [M+Na$^+$] 433.1797, found 433.1804.

$[\alpha]_D^{26}$ = +25.2 (*c* 0.28, $CHCl_3$)

Assignment of the Olefin Geometry

The olefin geometry of the carboxylic acid **6** was confirmed by the values of proton-carbon scalar coupling constants.[11] Conformational analysis of two model compounds baring either the *Z* or *E* olefin (**Model-*Z*** and **Model-*E*** respectively, **Table S5**) were performed and the ensemble averaged scalar coupling constants for both model compounds were computed by DFT calculations using the lowest energy conformers with a combined population of 97%.

Table S5 outlines the comparison between the relevant calculated ^1H-^{13}C values for both model compounds and the experimental values measured from the carboxylic acid **6** using IPAP HOBS-HSQMBC NMR spectra.[12] Compound **Model-*Z*** shows a good fit (MAD = 0.9 Hz, SD = 1.2 Hz), while compound **Model-*E*** provides an unreasonable fit (MAD = 3.2Hz, SD = 2.5 Hz). Therefore, the olefin geometry of carboxylic acid **6** was assigned to be *Z* configuration.

Table S5. Experimentally Measured ^1H-^{13}C Scalar Coupling Constants for Carboxylic Acid 6 and the Calculated Values for Model compounds (Model-*Z* and Model-*E*).

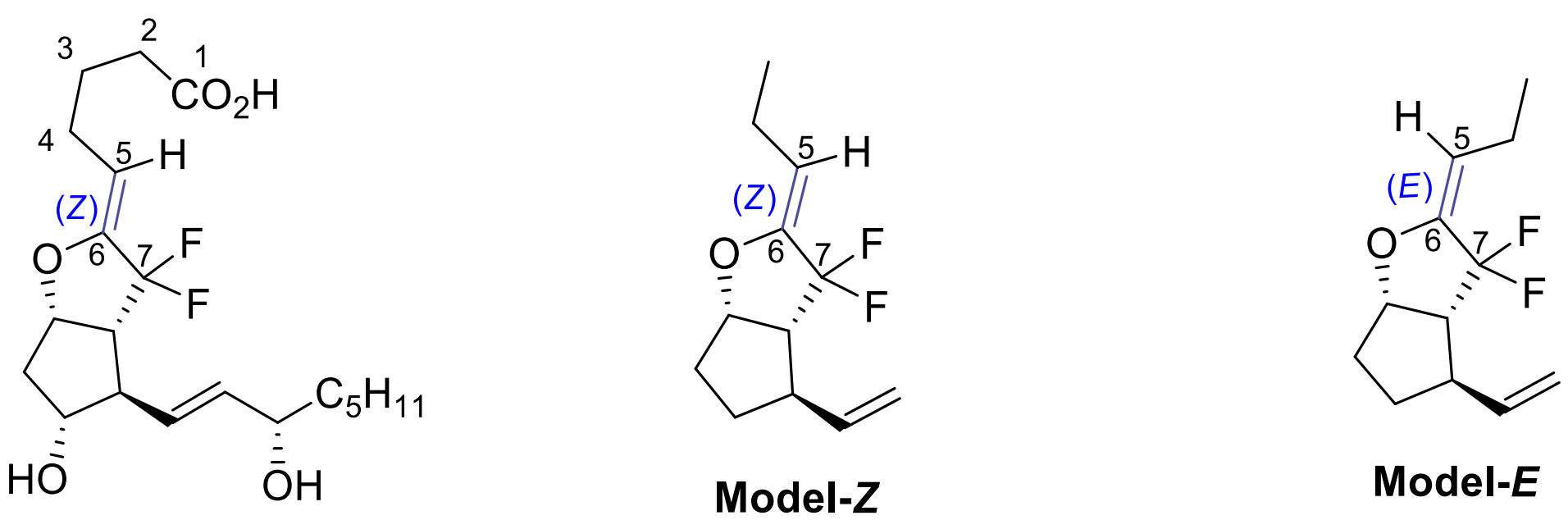

6 **Model-*Z*** **Model-*E***

$^nJ_{HC}$	Exp. $^nJ_{HC}$ /Hz	Calc. $^nJ_{HC}$ /Hz	Deviation /Hz	Calc. $^nJ_{HC}$ /Hz	Deviation/Hz
$^2J_{H5C6}$	5.4	4.5	0.9	6.8	−1.4
$^3J_{H5C7}$	2.6	3.4	−0.8	7.5	−4.9
MAD: Mean Absolute Deviation		**MAD**	0.9	**MAD**	3.2
SD: Standard Deviation		**SD**	1.2	**SD**	2.5

Computation experimental

Molecular mechanics calculation were performed using the MacroModel software package[13] (version 9.9) accessed through the Maestro[14] (version 9.2) program. A Monte Carlo multiple minimum (MCMM)[15] conformational search was used along with Merck Molecular Force Field (MMFFs) in gas phase.[16] Structures were minimised using the truncated Newton Conjugate Gradients (TNCG) method with 1,000 iterations with a gradient convergence criteria of 0.05 and 1,000,000 iterations were used for the search. Conformers within 21.0 kj/mol of

the lowest energy conformer found were stored and subjected to the subsequent Density Functional Theory (DFT) calculations.

All geometry optimisations, frequency calculations and NMR calculations were performed using the Gaussian 09 Software package.[17] Geometry optimisation and frequency calculations were performed using mPW1PW91 6-311 G (dp) level of theory with the Integral Equation Formalism Polarizable Continuum Model (IEF-PCM) continuum dielectric solvation model using chloroform as solvent (ε = 4.71).

The Boltzmann population of conformers (**Table S6**) were calculated based on the computed Gibb's free energies. The lowest energy conformers with a combined population of 97% were subjected to NMR calculations with 'GIAO' (gauge-independent atomic orbitals), 'spinspin' and 'mixed' keywords using mPW1PW91 6-311G (d,p) level of theory with chloroform as solvent (IEF-PCM). The computed scalar coupling constants for both conformers were Boltzmann averaged to deliver the computed values provided in table S6.

Table S6. Relative Gibb's Free Energies (*ΔG*, calculated by DFT mPW1PW91/6-311G (d,p) with $CHCl_3$ as solvent) for Major Conformers of Compounds Model-*Z* and Model-*E*.[a]

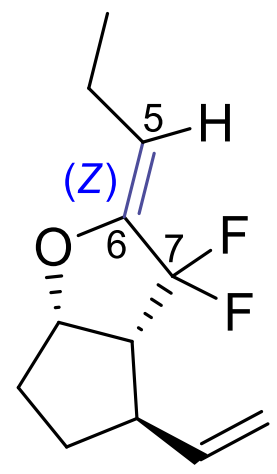

Model-*Z*

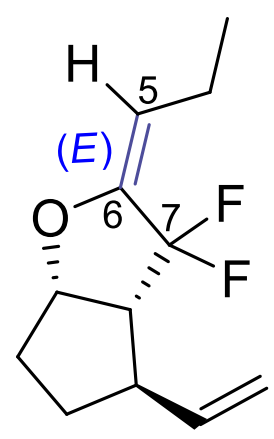

Model-*E*

Conformer	ΔG/ kJ/mol^{-1}	Population	Conformer	ΔG/ kJ/mol^{-1}	Population
1	0.00	19.81	1	0.00	29.61
2	0.47	16.40	2	1.30	17.50
3	0.70	14.94	3	1.96	13.40
4	1.10	12.72	4	2.96	8.97
5	1.64	10.22	5	3.00	8.81
6	2.78	6.44	6	4.11	5.63
7	4.56	3.14	7	4.29	5.23
8	4.70	2.97	8	4.75	4.35
9	5.03	2.60	9	5.05	3.86
10	5.11	2.52			
11	5.59	2.07			
12	5.92	1.81			
13	5.99	1.76			

[a] Cartesian coordinates of the conformers are attached as an Excel file.

Determination of Stereochemistry of Monofluorinated Lactone

^{1}H-^{19}F HOESY experiment and result for monofluorinated lactone 48

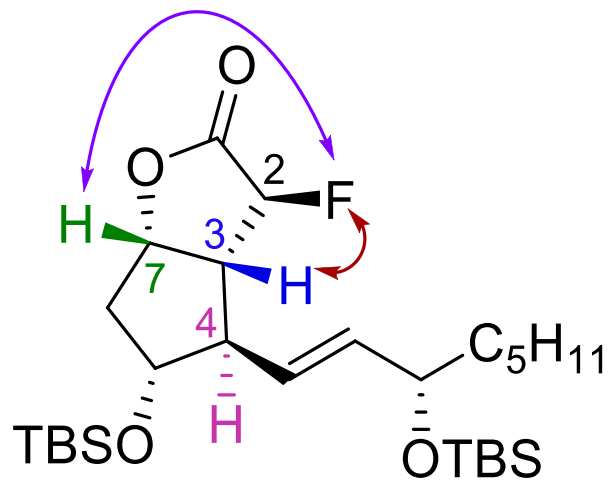

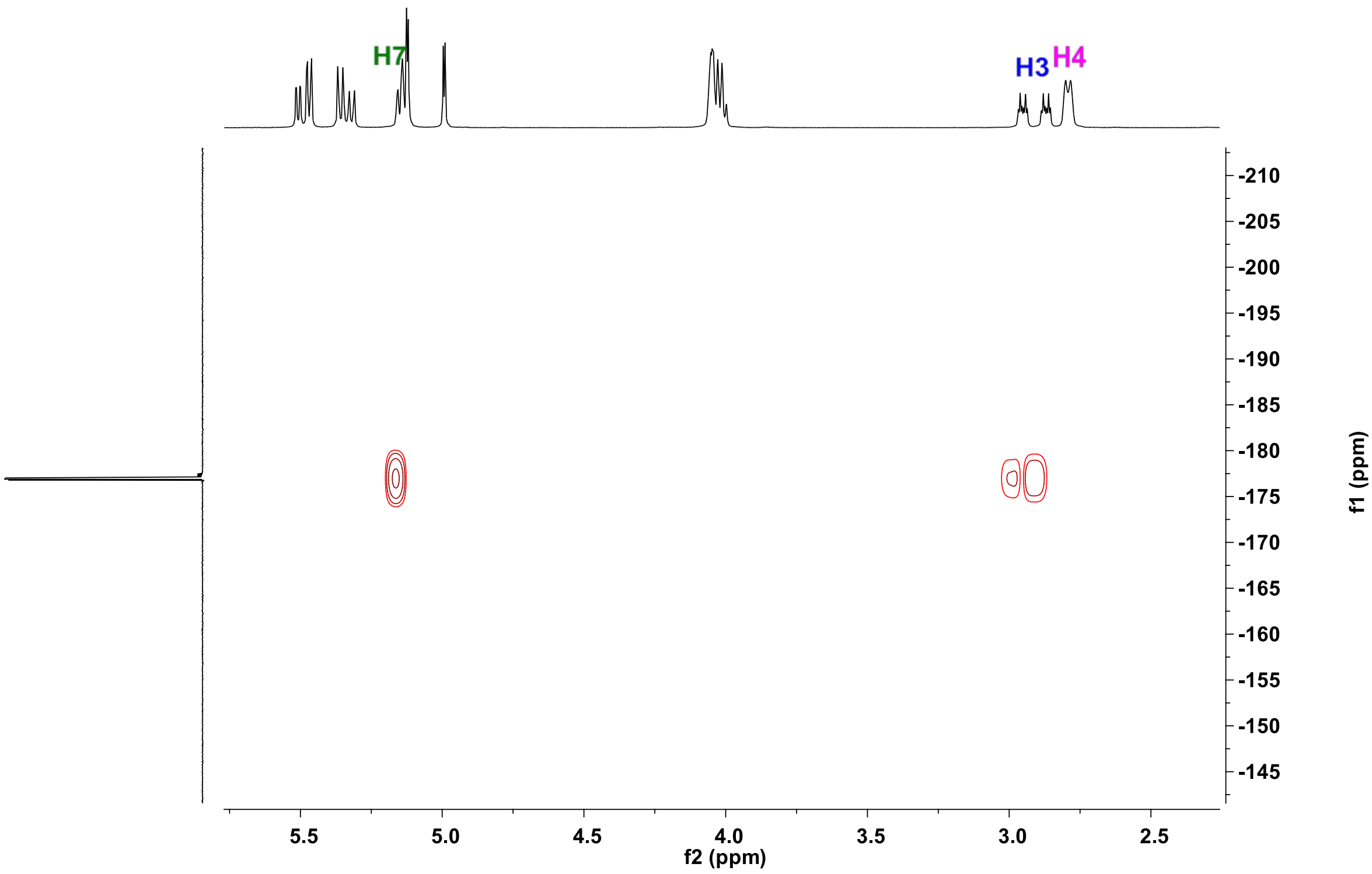

The stereochemistry of lactone **48** was established as (3*S*) for C(*H*)*F* centre by ^{1}H-^{19}F HOESY.

Notes and References

1. Pangborn, A. B.; Giardello, M. A.; Grubbs, R. H.; Rosen, R. K.; Timmers, F. J. Safe and convenient procedure for solvent purification. *Organometallics* **1996**, *15* (5), 1518 – 1520. DOI: 10.1021/om9503712.

2. Burchat, A. F.; Chong, J. M.; Nielsen, N. Titration of alkyllithiums with a simple reagent to a blue endpoint. *J Organomet Chem* **1997**, *542* (2), 281 – 283. DOI: 10.1016/S0022-328X(97)00143-5.

3. Pelss, A.; Gandhamsetty, N.; Smith, J. R.; Mailhol, D.; Silvi, M.; Watson, A. J. A.; Perez-Powell, I.; Prévost, S.; Schützenmeister, N.; Moore, P. R.; Aggarwal, V. K. Re-optimization of the organocatalyzed double aldol domino process to a key enal intermediate and its application to the total synthesis of Δ^{12}-prostaglandin J_3. *Chem Eur J* **2018**, *24* (38), 9542 – 9545. DOI: 10.1002/chem.201802498.

4. Prévost, S.; Thai, K.; Schützenmeister, N.; Coulthard, G.; Erb, W.; Aggarwal, V. K. Synthesis of prostaglandin analogues, Latanoprost and Bimatoprost, using organocatalysis *via* a key bicyclic enal intermediate. *Org Lett* **2015**, *17* (3), 504 – 507. DOI: 10.1021/ol503520f.

5. Yadav, J. S.; Deshpande, P. K.; Sharma, G. V. M. Stereoselective synthesis of (*S*)-13-hydroxy octadeca-(9*Z*,11*E*)-di- and (9*Z*,11*E*,15*Z*)-trienoic acids: Selfdefensive substances against rice blast disease. *Tetrahedron* **1992**, *48* (21), 4465-4471.

6. Noyori, R.; Tomino, I.; Yamada, M.; Nishizawa, M. Synthetic applications of the enantioselective reduction by binaphthol-modified lithium aluminum hydride reagents. *J Am Chem Soc* **1984**, *106* (22), 6717 – 6725. DOI: 10.1021/ja00334a042.

7. Nicolaou, K. C.; Veale, C. A.; Webber, S. E.; Katerinopoulos, H. Stereocontrolled total synthesis of lipoxins A. *J Am Chem Soc* **1985**, *107* (25), 7515 – 7518. DOI: 10.1021/ja00311a048.

8. McGee, P.; Bellavance, G.; Korobkov, I.; Tarasewicz, A.; Barriault, L. Synthesis and isolation of organogold complexes through a controlled 1,2-silyl migration. *Chem Eur J* **2015**, *21* (27), 9662 – 9665. DOI: 10.1002/chem.201501648.

9. Lin, Q. Y.; Meloni, D.; Pan, Y. C.; Xia, M.; Rodgers, J.; Shepard, S.; Li, M.; Galya, L.; Metcalf, B.; Yue, T.-Y.; Liu, P. L; Zhou, J. C. Enantioselective synthesis of Janus kinase inhibitor INCB018424 *via* an organocatalytic aza-Michael reaction. *Org Lett* **2009**, *11* (9), 1999 – 2002. DOI: 10.1021/ol900350k.

10. Al Dulayymi, J. R.; Baird, M. S.; Roberts, E.; Minnikin, D. E. The synthesis of single enantiomers of meromycolic acids from mycobacterial wax esters. *Tetrahedron* **2006**, *62* (51), 11867 – 11880. DOI: 10.1016/j.tet.2006.09.019.

11. Bifulco, G.; Dambruoso, P.; Gomez-Paloma, L.; Riccio, R. Determination of relative configuration in organic compounds by NMR spectroscopy and computational methods. *Chem Rev* **2007**, *107* (9), 3744 – 3779. DOI: 10.1021/cr030733c.

12. Castañar, L.; Saurí, J.; Nolis, P.; Virgili, A.; Parella, T. Implementing homo- and heterodecoupling in region-selective HSQMBC experiments. *J Magn Reson* **2014**, *238*, 63 – 69. DOI: 10.1016/j.jmr.2013.10.022.

13. Mohamadi, F.; Richards, N. G. J.; Guida, W. C.; Liskamp, R.; Lipton, M.; Caufield, C.; Chang, G.; Hendrickson, T.; Still, W. C. Macromodel—an integrated software system for modeling organic and bioorganic molecules using molecular mechanics. *J Comput Chem* **1990**, *11* (4), 440 – 467. DOI: 10.1002.jcc.540110405.

14. Maestro, Version 9.2, Schrödinger, LLC, New York, NY, 2011.

15. Chang, G.; Guida, W. C.; Still, W. C. An internal-coordinate Monte Carlo method for searching conformational space. *J Am Chem Soc* **1989**, *111* (12), 4379 – 4386. DOI: 10.1021/ja00194a035.

16. Halgren, T. A. Merck molecular force field. I. Basis, form, scope, parameterization, and performance of MMFF94. *J Comp Chem* **1996**, *17* (5-6), 490 – 519. DOI: 10.1002/(SICI)1096-987X(199604)17:5/6<490::AID-JCC1>3.0.CO;2-P.

17. Gaussian 09, Revision D.01, Frisch, M. J.; Trucks, G. W.; Schlegel, H. B.; Scuseria, G. E.; M. A. Robb, J. R. C.; G. Scalmani, V. B.; Mennucci, B.; Petersson, G. A.; Nakatsuji, H.; Caricato, M.; X. Li, H. P. H.; Izmaylov, A. F.; J. Bloino, G. Z.; Sonnenberg, J. L.; M. Hada, M. E.; K. Toyota, R. F.; J. Hasegawa, M. I.; T. Nakajima, Y. H.; O. Kitao, H. N.; Vreve, T.; Montgomery, J. A.; Jr., J. E. P.; Ogliaro, F.; Bearpark, M.; Heyd, J. J.; Brothers, E.; Kudin, K. N.; Staroverov, V. N.; Kobayashi, R.; Norman, J.; Raghavachari, K.; Rendell, A.; Burant, J. C.; Iyengar, S. S.; Tomasi, J.; Cossi, M.; Rega, N.; Millam, J. M.; Klene, M.; Knox, J. E.; Cross, J. B.; Bakken, V.; Adamo, C.; Jaramillo, J.; Gomperts, R.; Stratmann, R. E.; Yazyev, O.; Austin, A. J.; Cammi, R.; Pomelli, C.; Ochterski, J. W.; Martin, R. L.; Morokuma, K.; Zakrzewski, V. G.; G. A. Voth, P. S.; Dannenberg, J. J.; Dapprich, S.; Daniels, A. D.; Farkas, Ö.; Foresman, J. B.; Ortiz, J. V.; Cioslowski, J.; Fox, D. J., Gaussian, Inc., Wallingford CT, 2009.

18. Wang, J. J.; Jin, S.; Zhang, H.; Xu, Y.; Hu, W.; Jiang, Y.; Chen, C.; Wang, D. W.; Xu, H. E.; Wu, C. Molecular recognition and activation of the prostacyclin receptor by anti-pulmonary arterial hypertension drugs. *Sci Adv* **2024**, *10* (6), eadk5184. DOI: 10.1126/sciadv.adk5184.

19. McNutt, A. T.; Francouer, P.; Aggarwal, R.; Masuda, T.; Meli, R.; Ragazo, M.; Sunseri, J.; Koes, D. R. GNINA 1.0: molecular docking with deep learning. *J Cheminform* **2021**, *13*, 43. DOI: 10.1186/s13321-021-00522-2.
20. Adasme, M. F.; Linnemann, K. L.; Bolz, S. N.; Kaiser, F.; Salentin, S.; Haupt, V. J.; Schroeder, M. PLIP 2021: expanding the scope of the protein-ligand interaction profiler to DNA and RNA. *Nucleic Acids Res* **2021**, *49* (W1), W450 – W534. DOI: 10.1093/nar/gkab294.
21. Brooks, B. R.; Brooks 3rd, C. L.; Mackerell Jr., A. D.; Nilsson, L.; Petrella, R. J.; Roux, B.; Won, Y.; Archontis, G.; Bartels, C.; Boresch, S.; Caflisch, A.; Caves, L.; Cui, Q.; Dinner, A. R.; Feig, M.; Fischer, S.; Gao, J.; Hodoscek, M.; Im, W.; Kuczera, K.; Lazaridis, T.; Ma, J.; Ovchinnikov, V.; Paci, E.; Pastor, R. W.; Post, C. B.; Pu, J. Z.; Schaefer, M.; Tidor, B.; Venable, R. M.; Woodcock, H. L.; Wu, X.; Yang, W.; York, D. M.; Karplus, M. CHARMM: the biomolecular simulation program. *J Comput Chem* **2009**, *30* (10), 1545 – 1614. DOI: 10.1002/jcc.21287.
22. Best, R. B.; Zhu, X.; Shim, J.; Lopes, P. E. M.; Mittal, J.; Feig, M.; MacKerell Jr., A. D. Optimisation of the additive CHARMM all-atom protein force field targeting improved sampling of the backbone ϕ, ψ and side-chain χ_1 and χ_2 dihedral angles. *J Chem Theory Comput* **2012**, *8* (9), 3257 – 3273. DOI: 10.1021/ct300400x.
23. Huang, J.; Rauscher, S.; Nawrocki, G.; Ran, T.; Feig, M.; Groot, B. L.; Grubmuller, H.; MacKerell Jr., A. D. CHARMM36m: an improved force field for folded and intrinsically disordered proteins. *Nature Methods* **2016**, *14* (1), 71 – 73. DOI: 10.1038/NMETH.4067.
24. Bekker, H.; Berendsen, H. J. C.; Dijkstra, E. J.; Achterop, S.; van Drunen, R.; van der Spoel, D.; Sijbers, A.; Keegstra H. "Gromacs: A parallel computer for molecular dynamics simulations"; pp. 252–256 in Physics computing 92. Edited by R.A. de Groot and J. Nadrchal. World Scientific, Singapore, 1993.
25. Valdes-Tresanco, M. S.; Valdes-Tresanco, M. E.; Valiente, P. A.; Moreno, E. gmx_MMPBSA: A new tool to perform end-state free energy calculations with GROMACS. *J Chem Theory Comput* **2021**, *17* (10), 6281 – 6291. DOI: 10.1021/acs.jctc.1c00645.

NMR Spectra for the Products

(*S*)-1-Bromooct-1-yn-3-ol (S4)

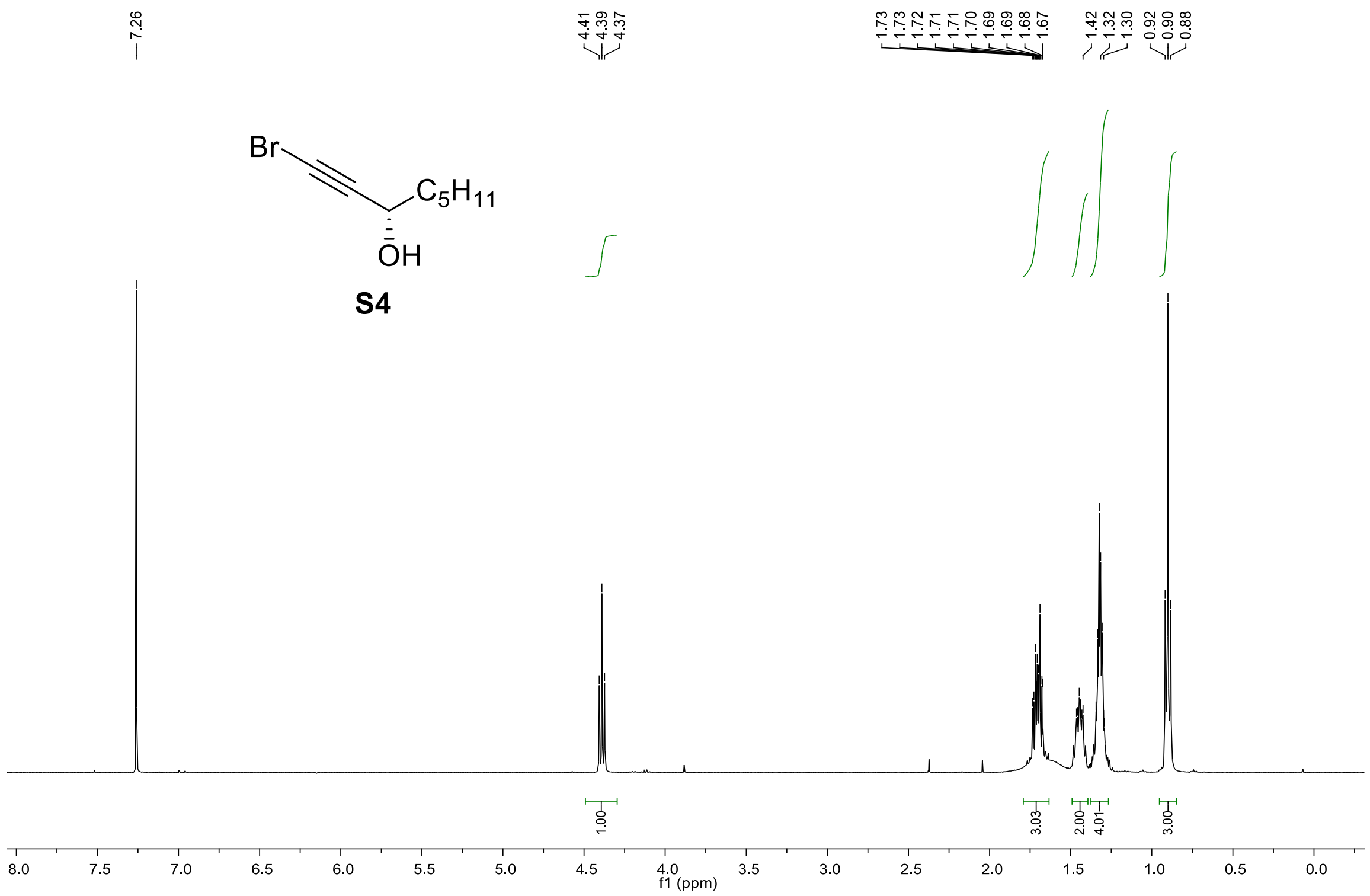

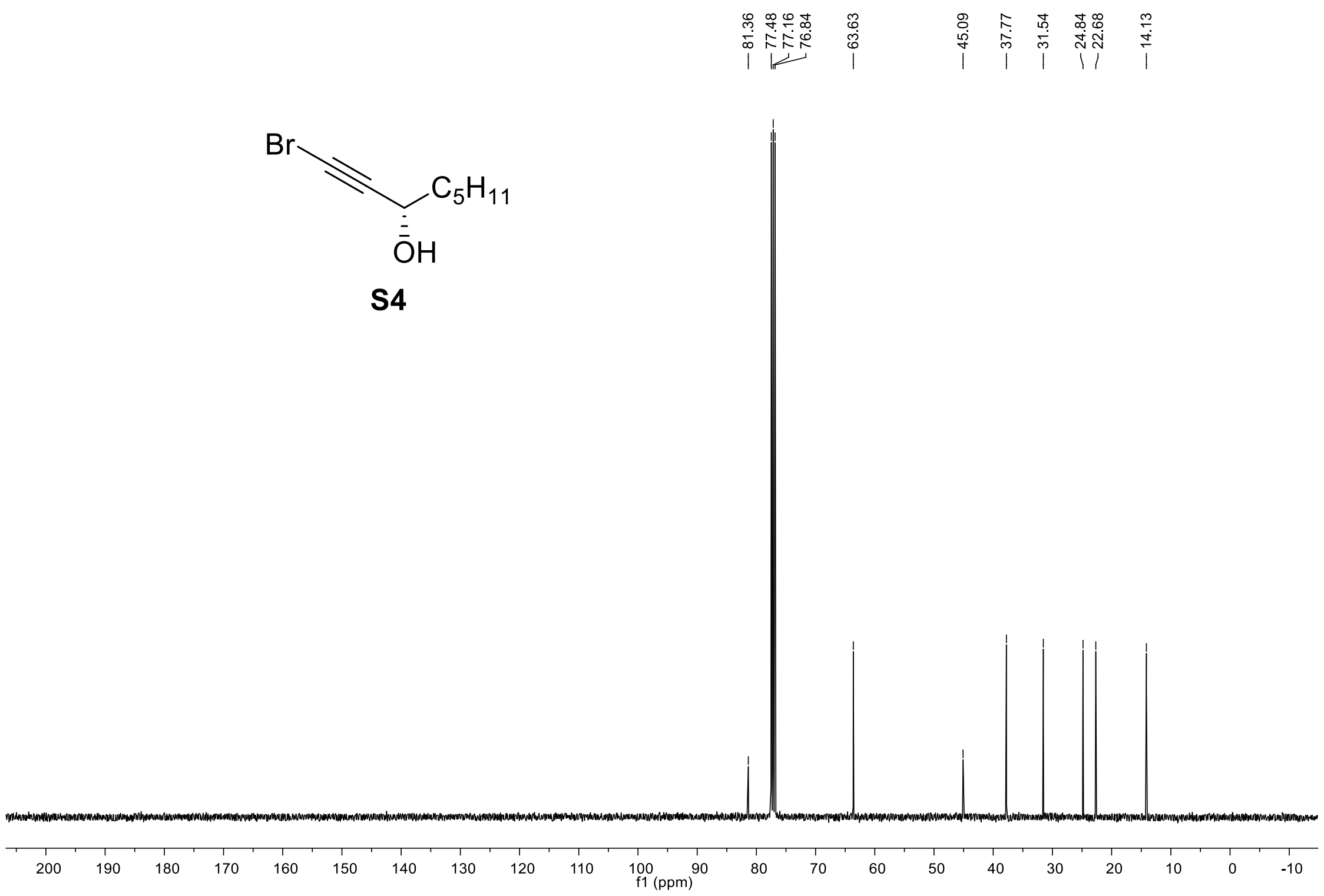

(*S*,*E*)-1-Bromooct-1-en-3-ol (S5)

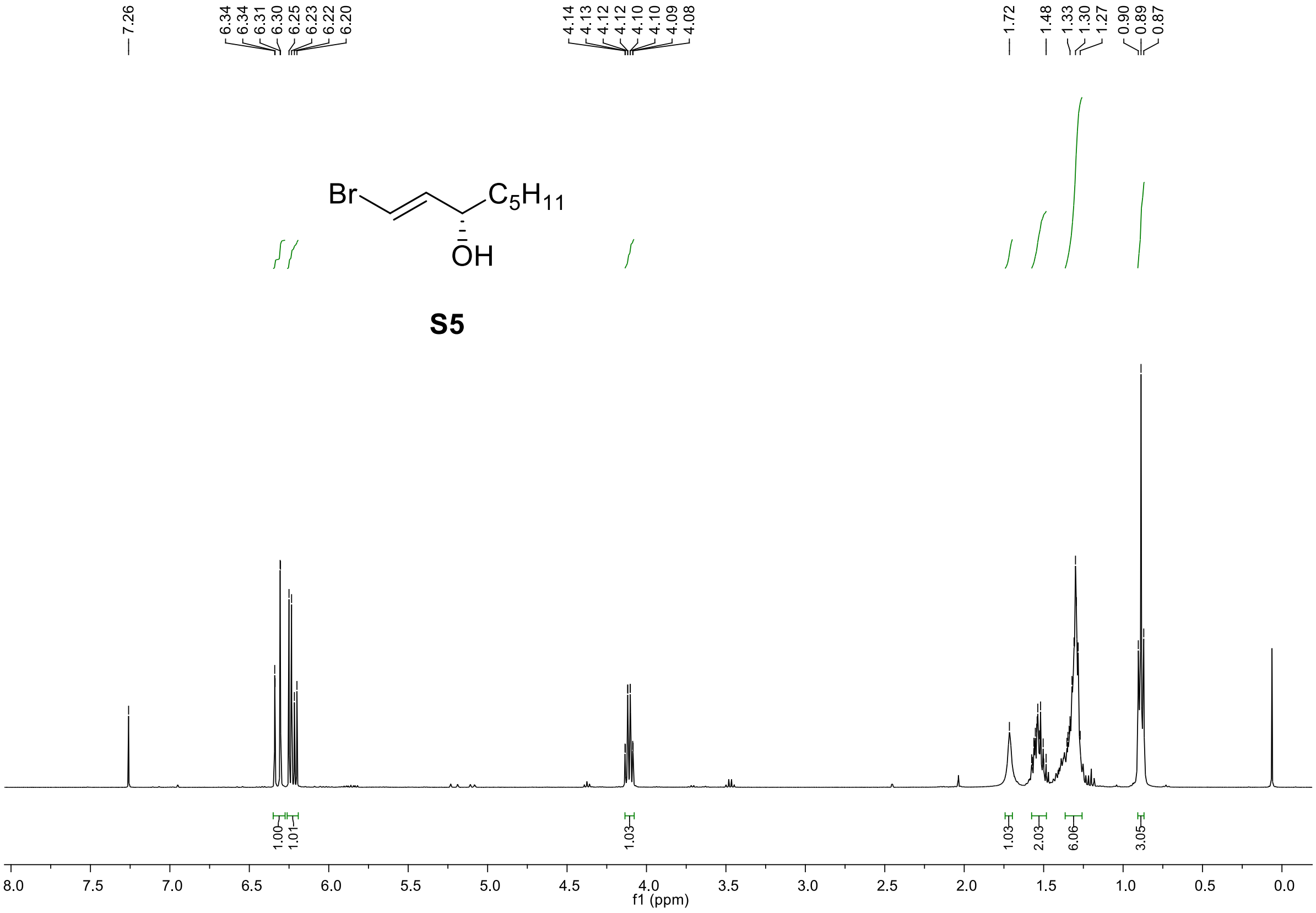

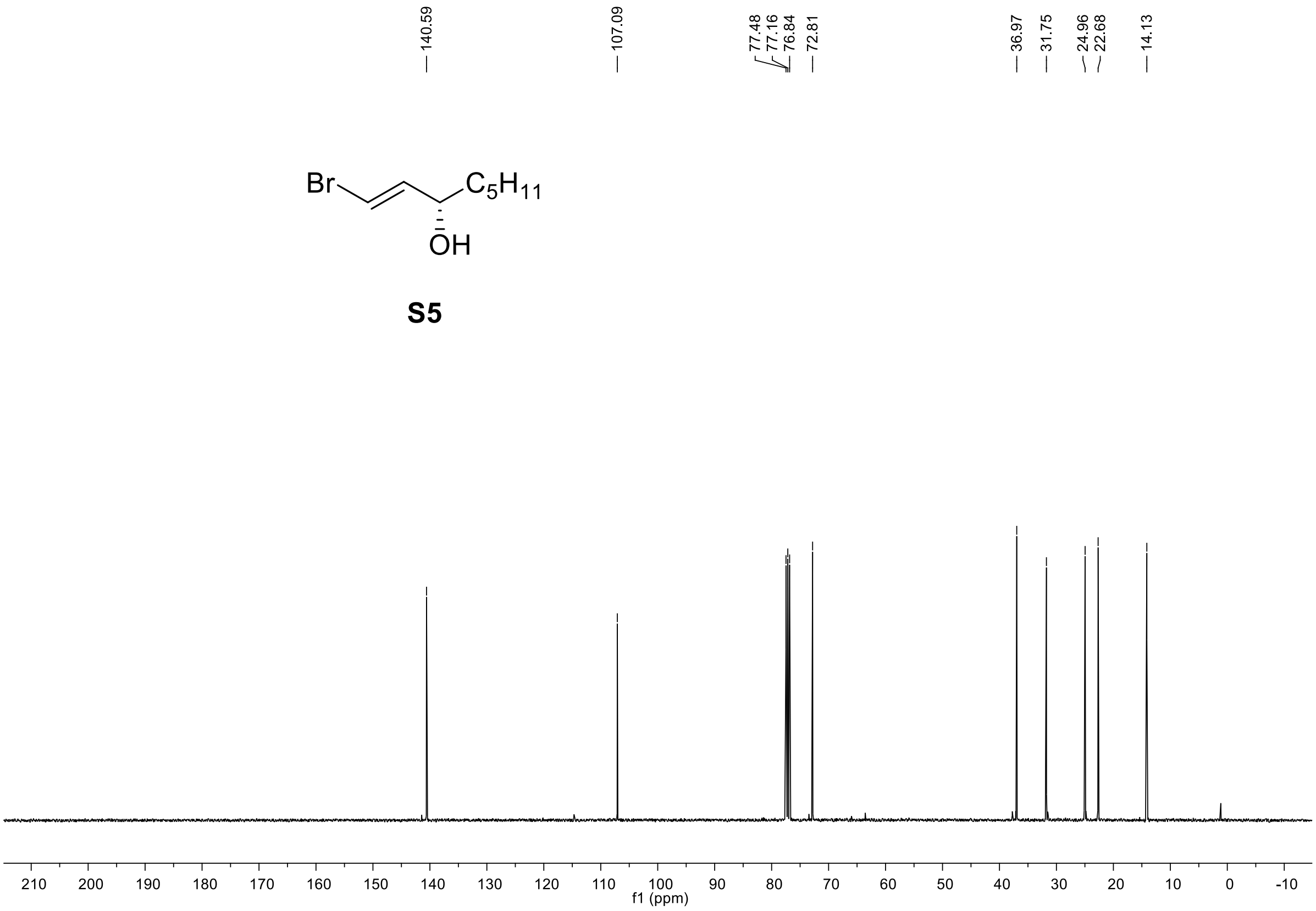

(*S*,*E*)-((1-Bromooct-1-en-3-yl)oxy)(*tert*-butyl)dimethylsilane (S6)

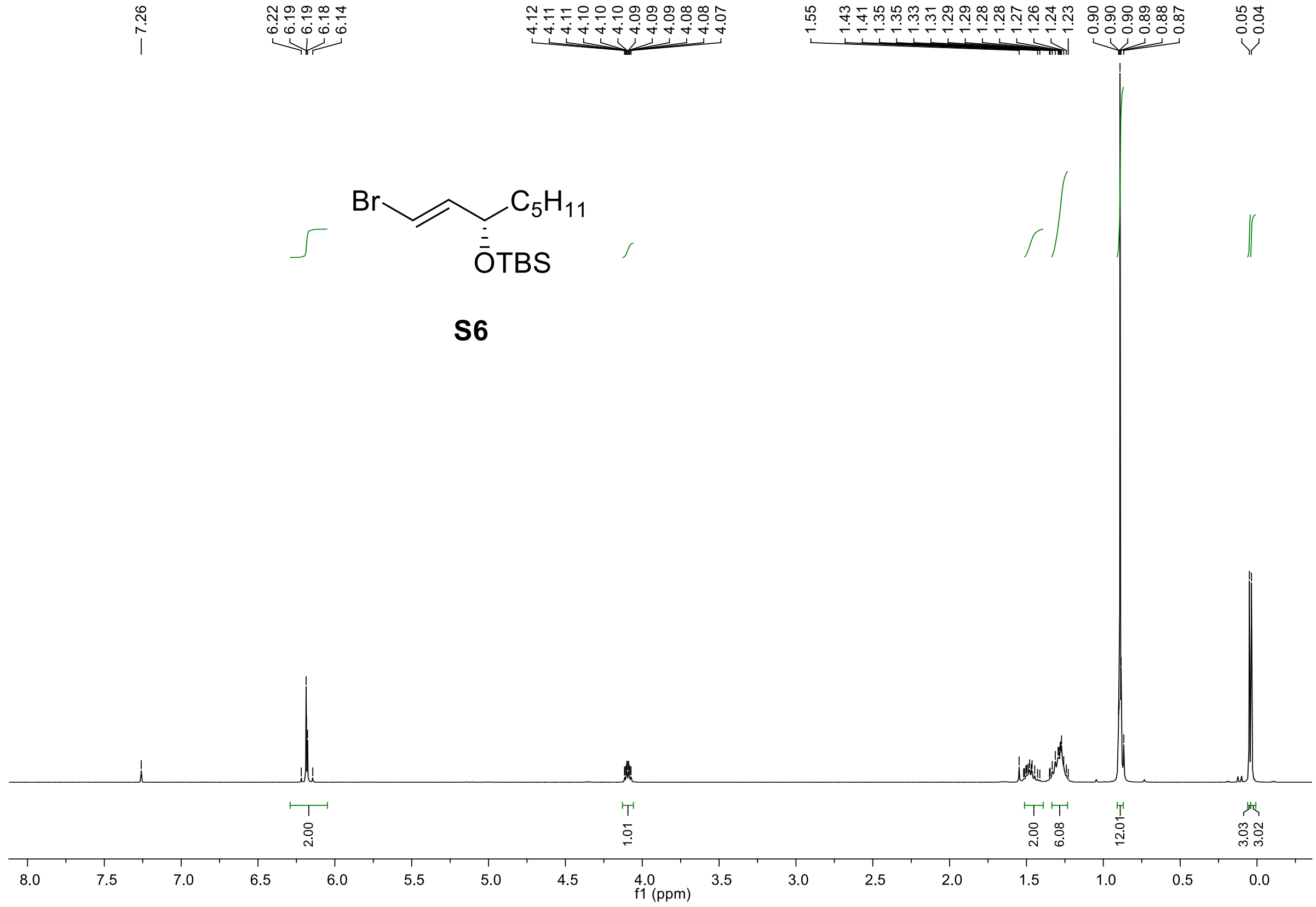

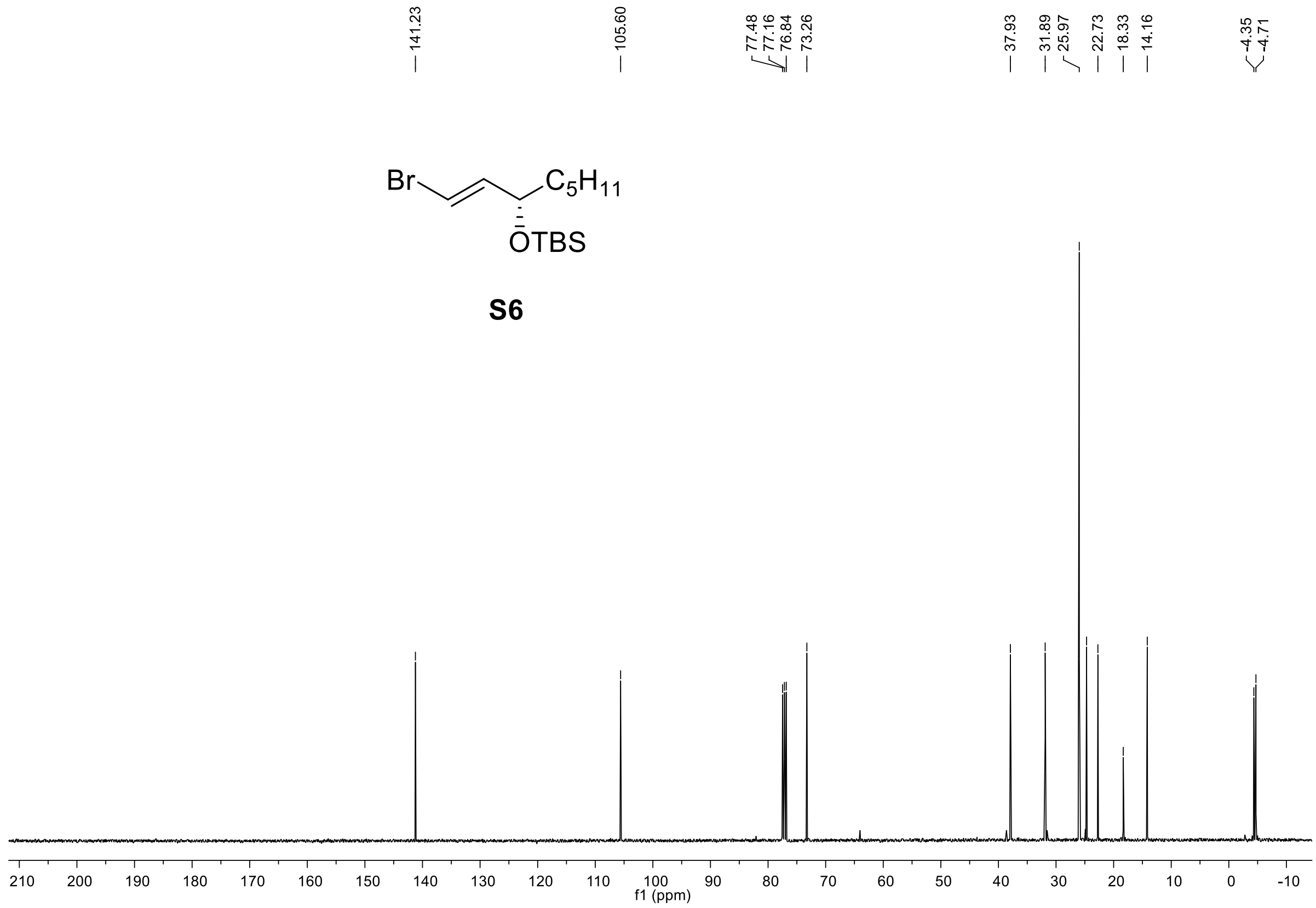

(2*S*,3*S*)-2-Methyl-5-(triethylsilyl)pent-4-yne-1,3-diol (14)

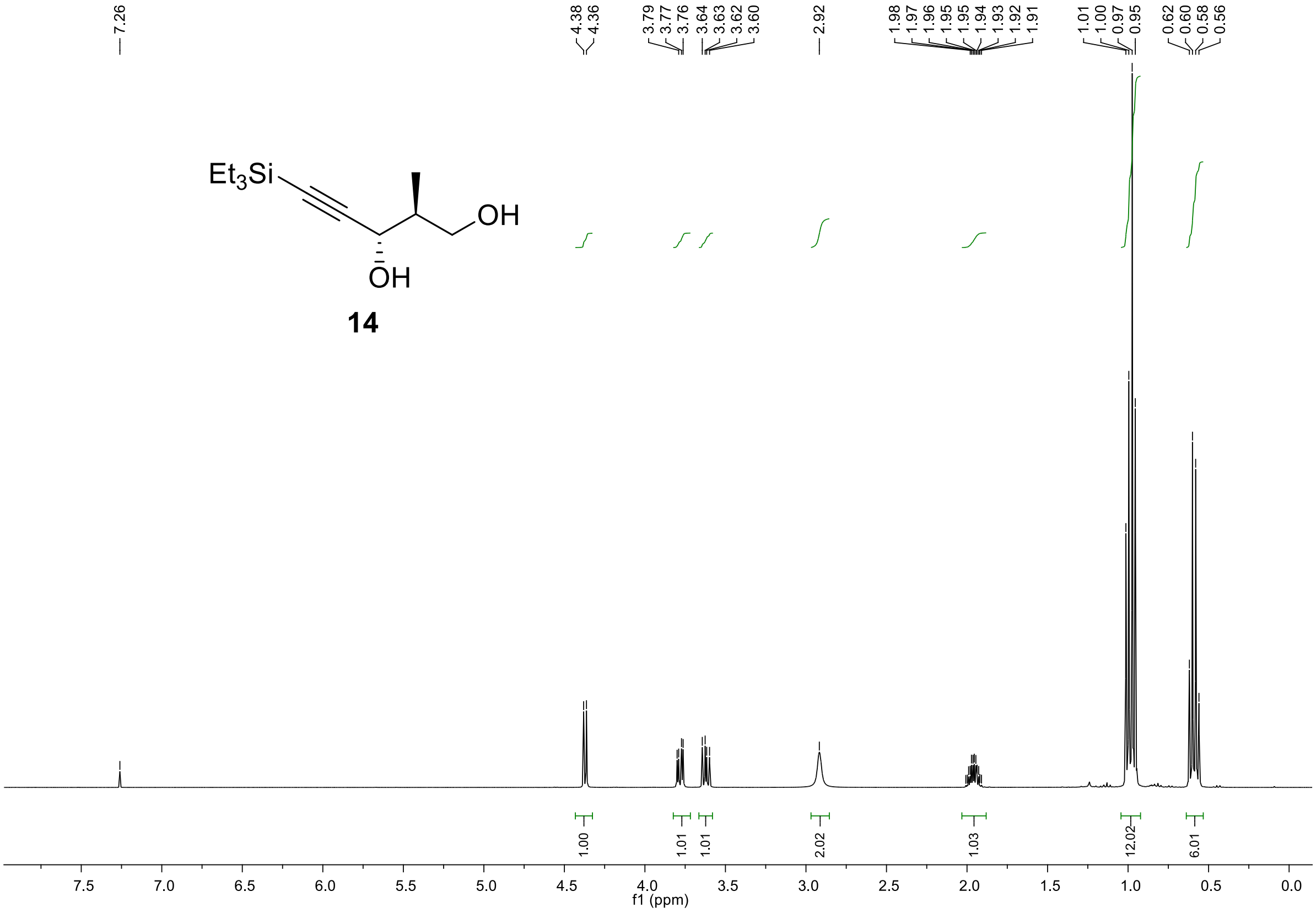

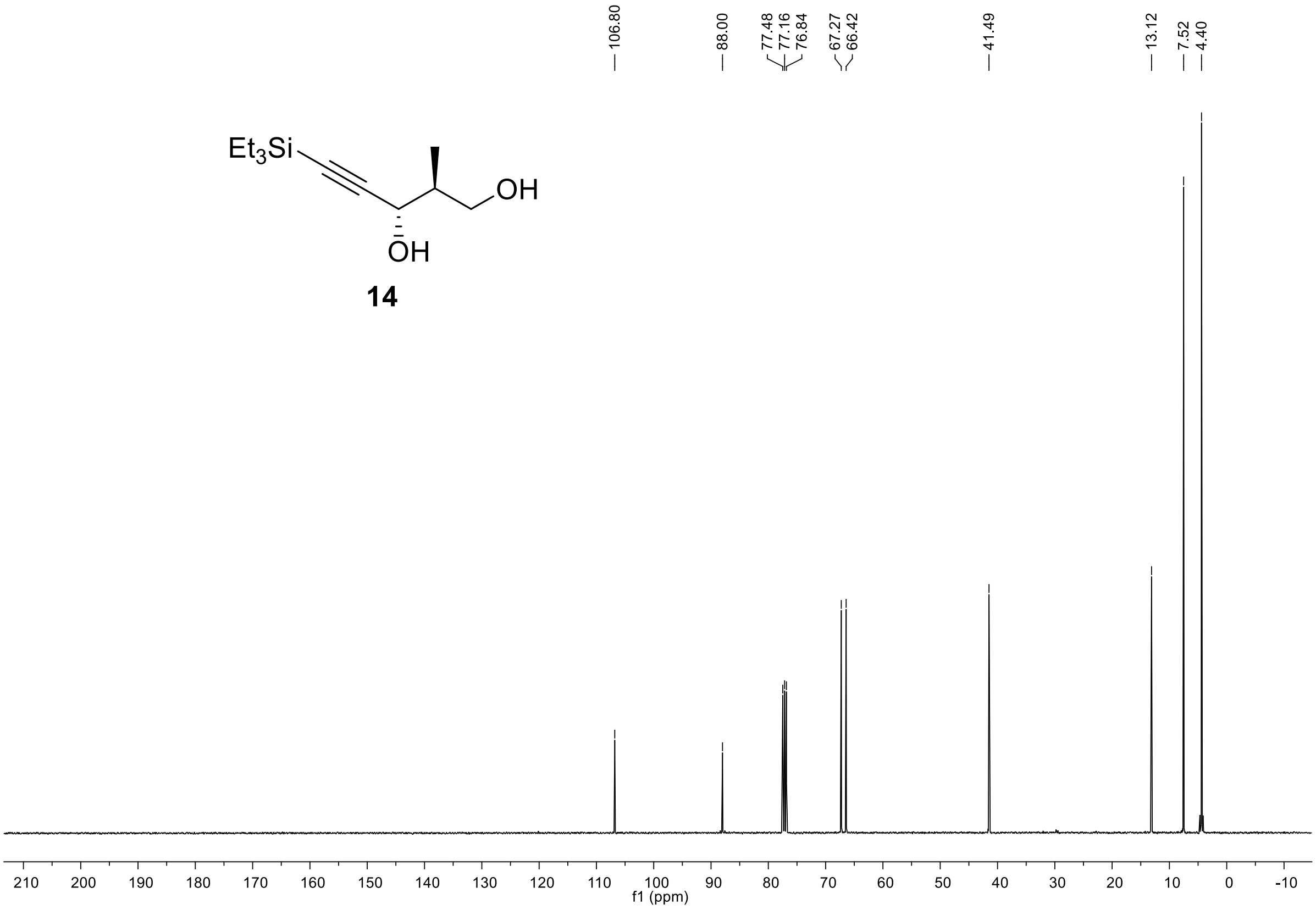

Benzyl (2*S*,3*S*)-3-hydroxy-2-methyl-5-(triethylsilyl)pent-4-ynyl carbonate (S7)

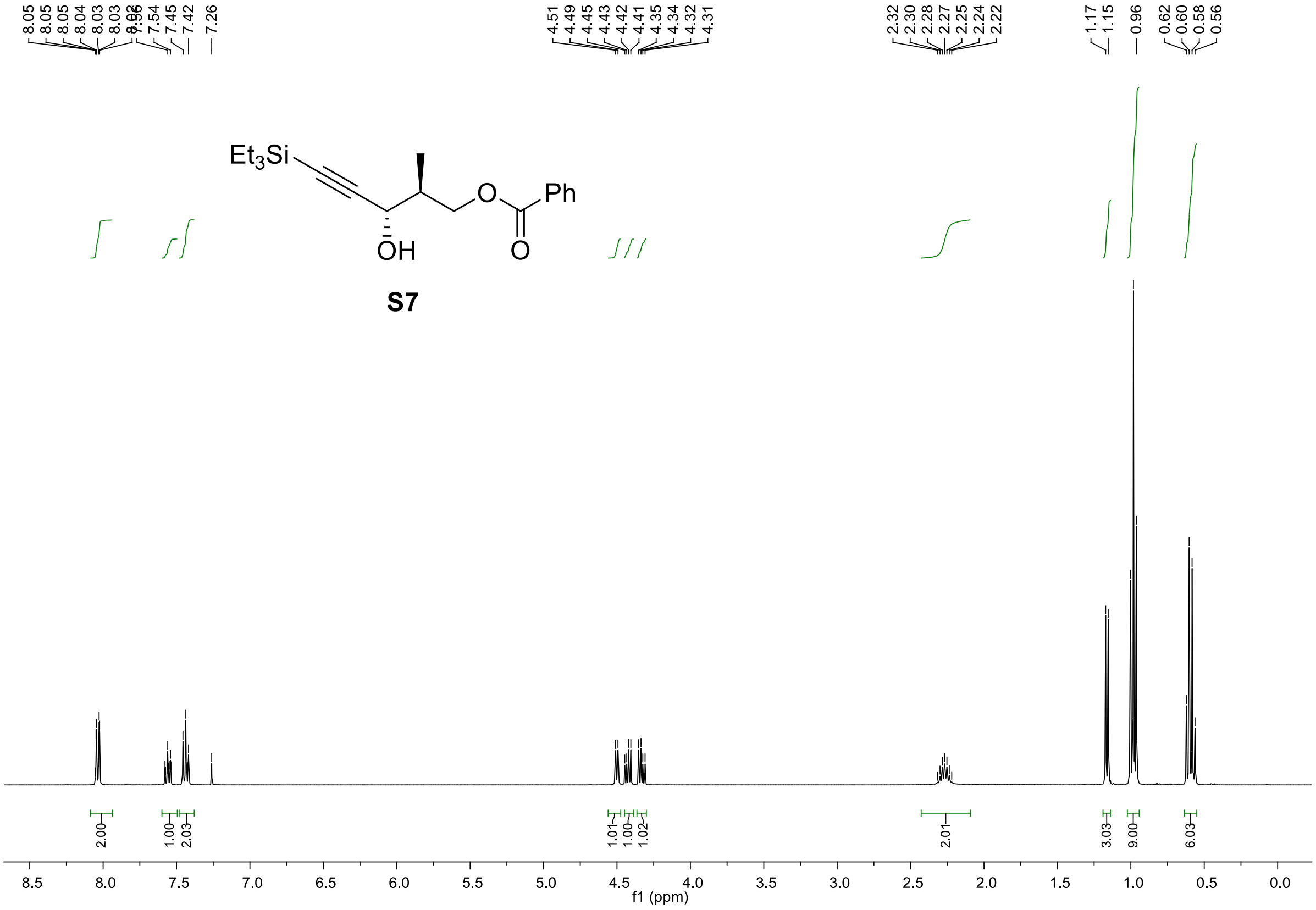

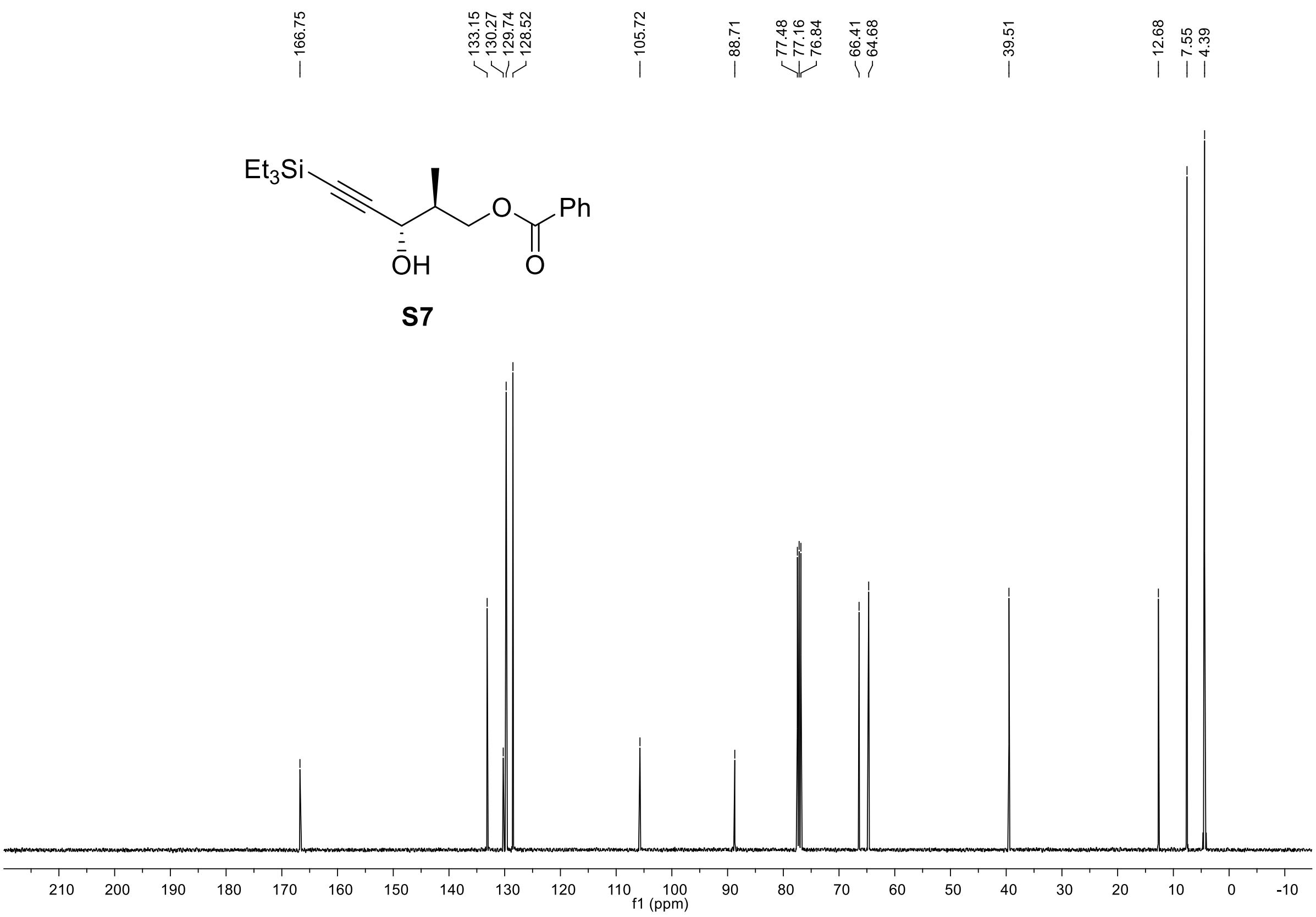

(2*S*,3*S*)-3-(*tert*-Butyldimethylsilyloxy)-2-methyl-5-(triethylsilyl)pent-4-ynyl trifluoromethanesulfonate (15)

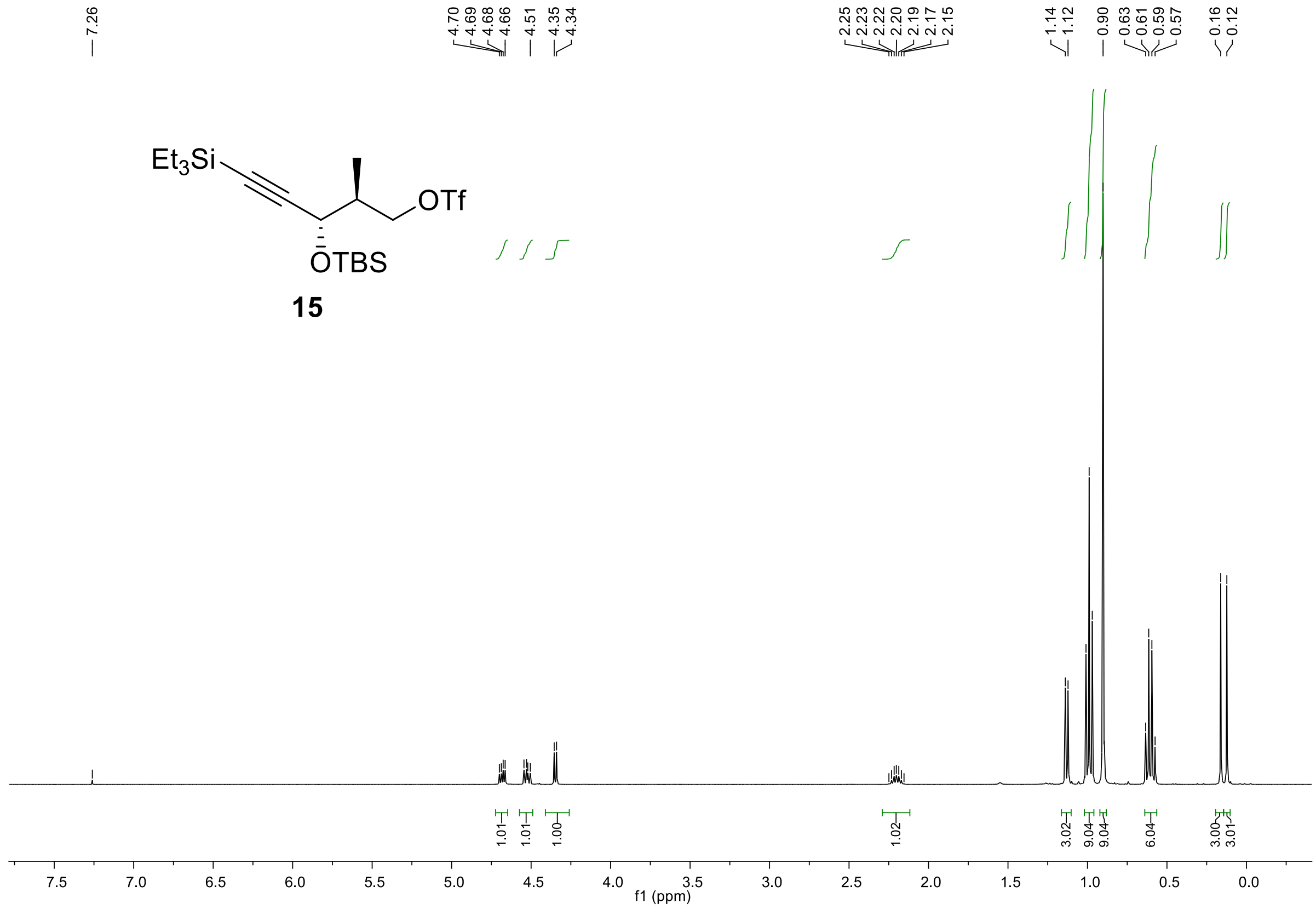

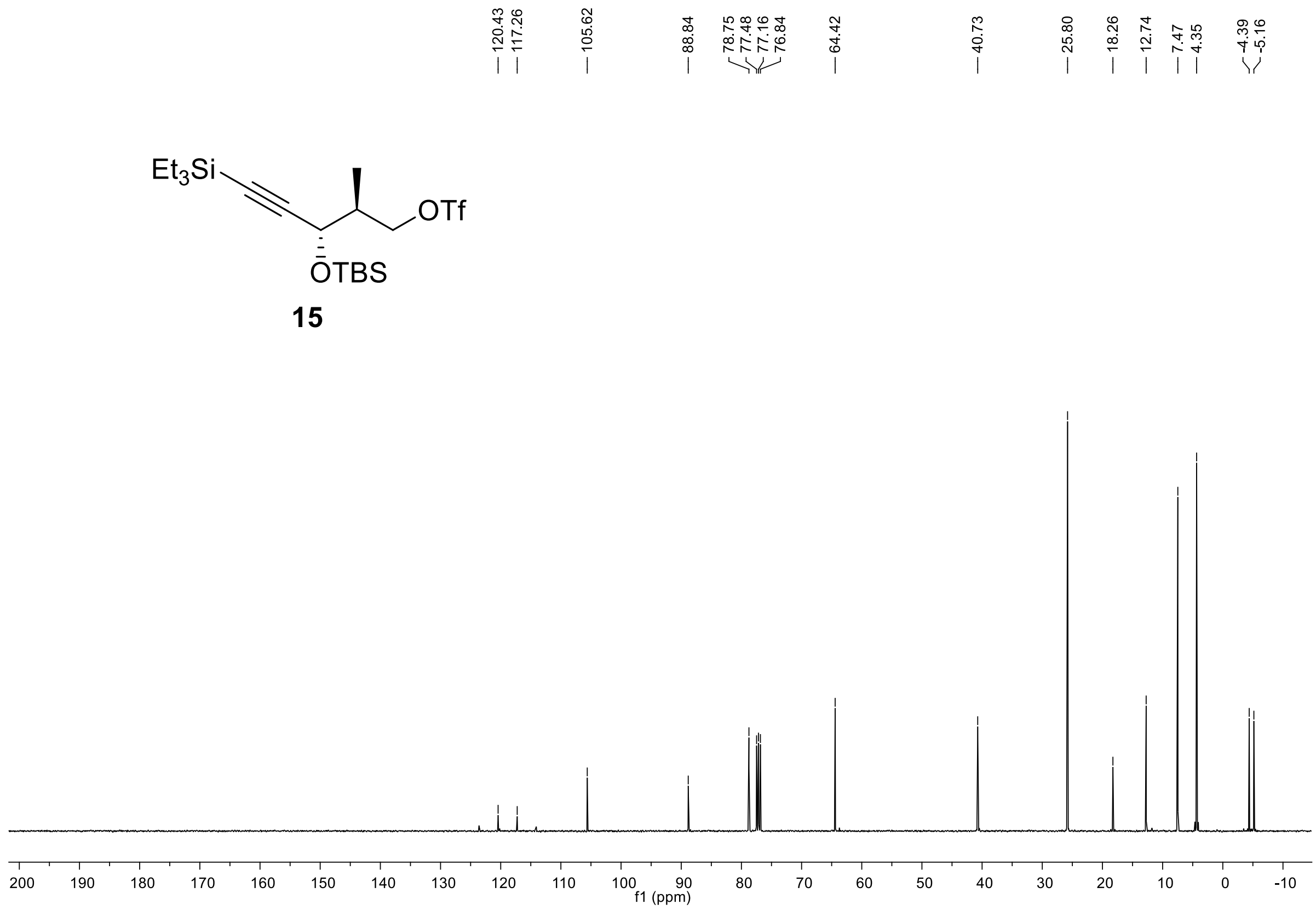

***tert*-Butyldimethyl((3*S*,4*S*)-4-methyl-1-(triethylsilyl)octa-1,6-diyn-3-yloxy)silane (16)**

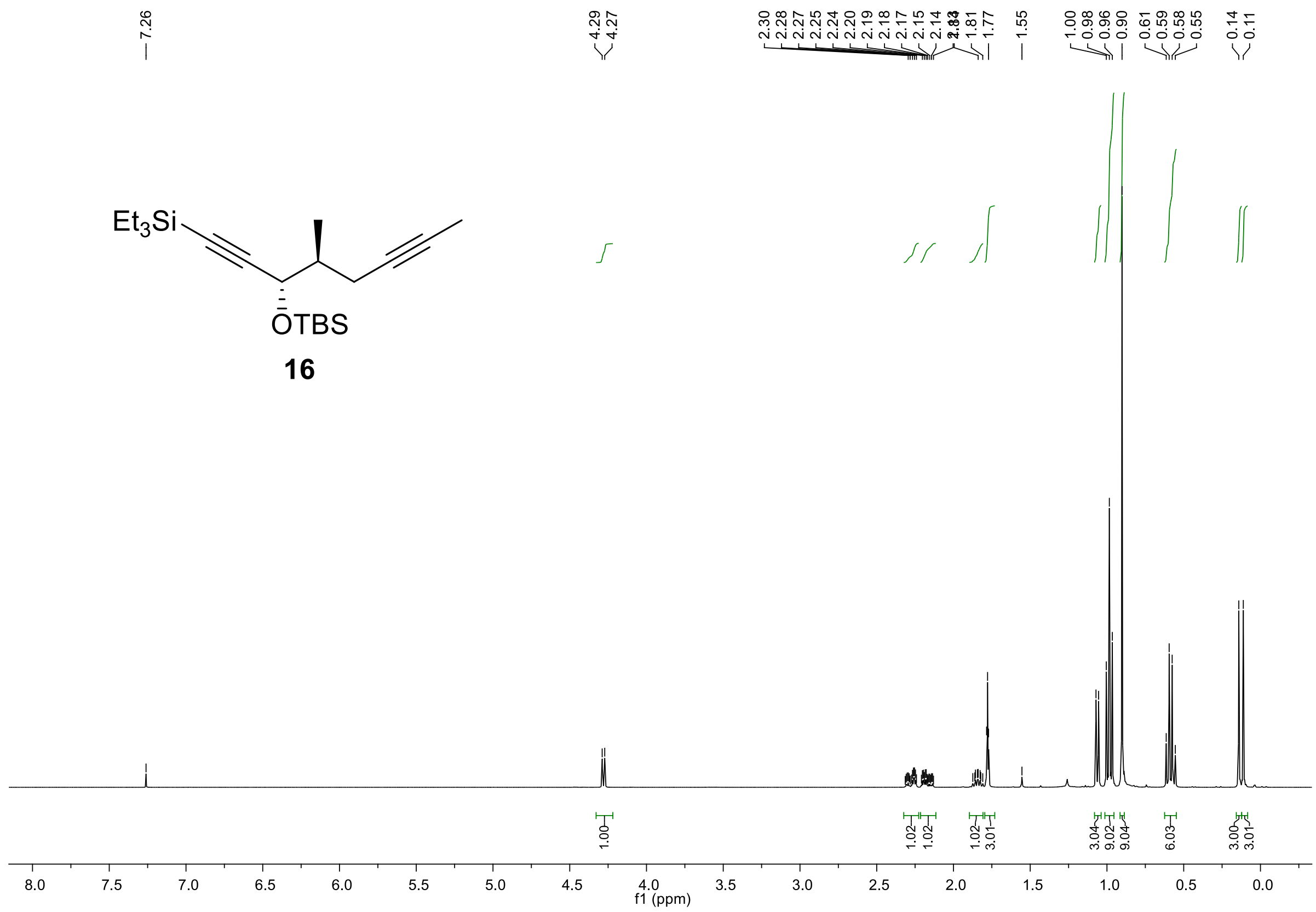

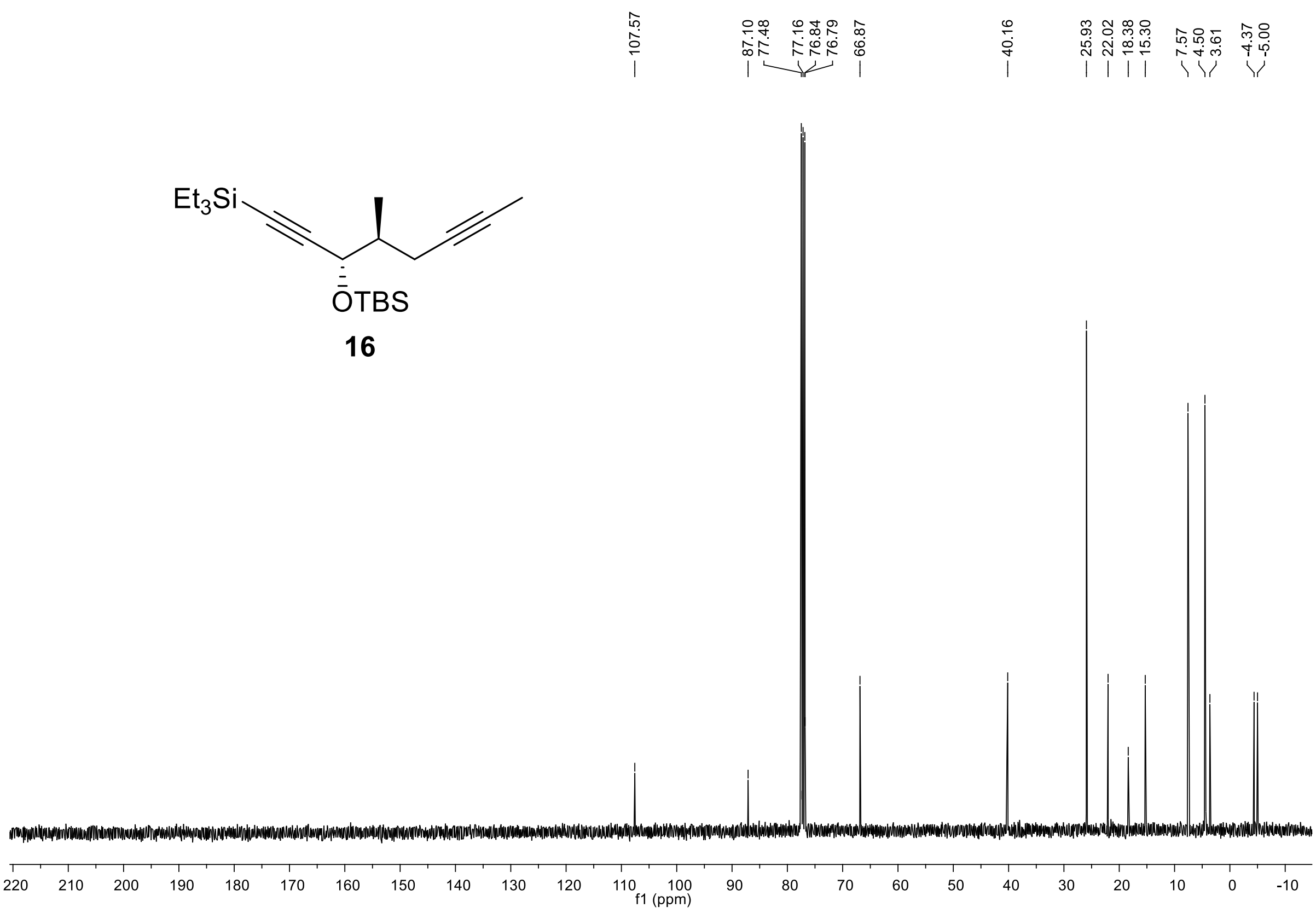

***tert*-Butyldimethyl((3*S*,4*S*)-4-methylocta-1,6-diyn-3-yloxy)silane (17)**

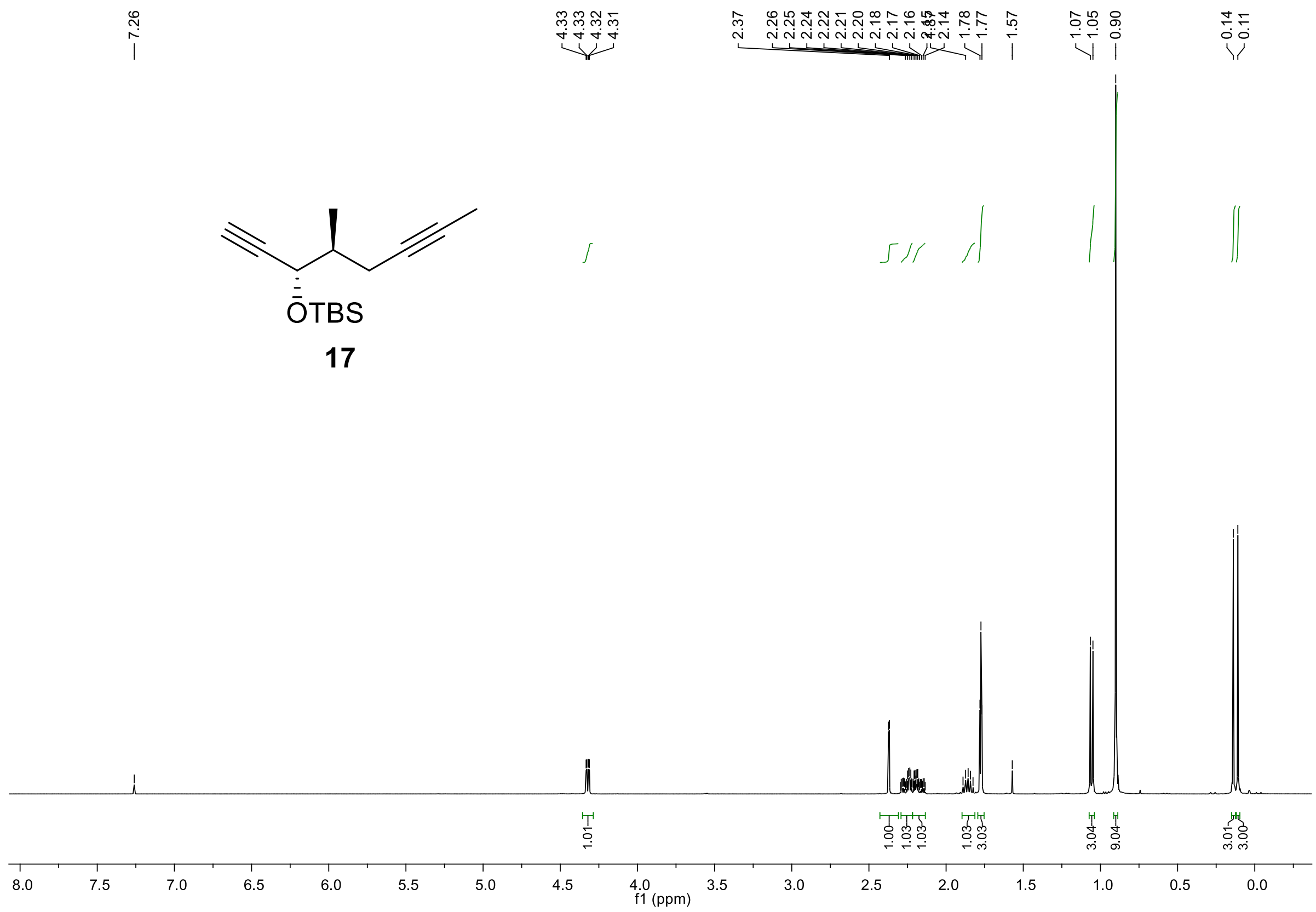

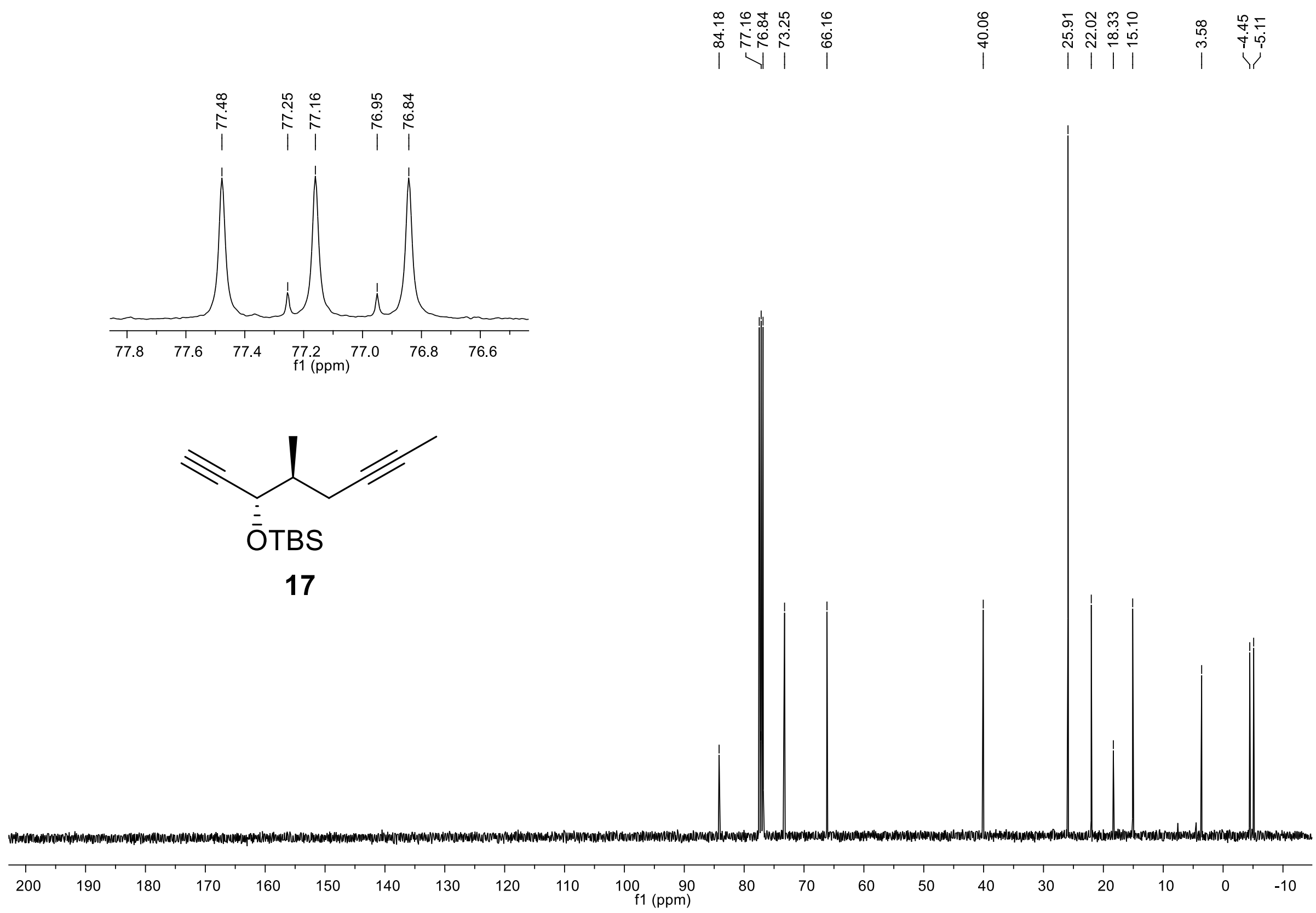

***tert*-Butyl((3*S*,4*S*,*E*)-1-iodo-4-methyloct-1-en-6-yn-3-yloxy)dimethylsilane (18)**

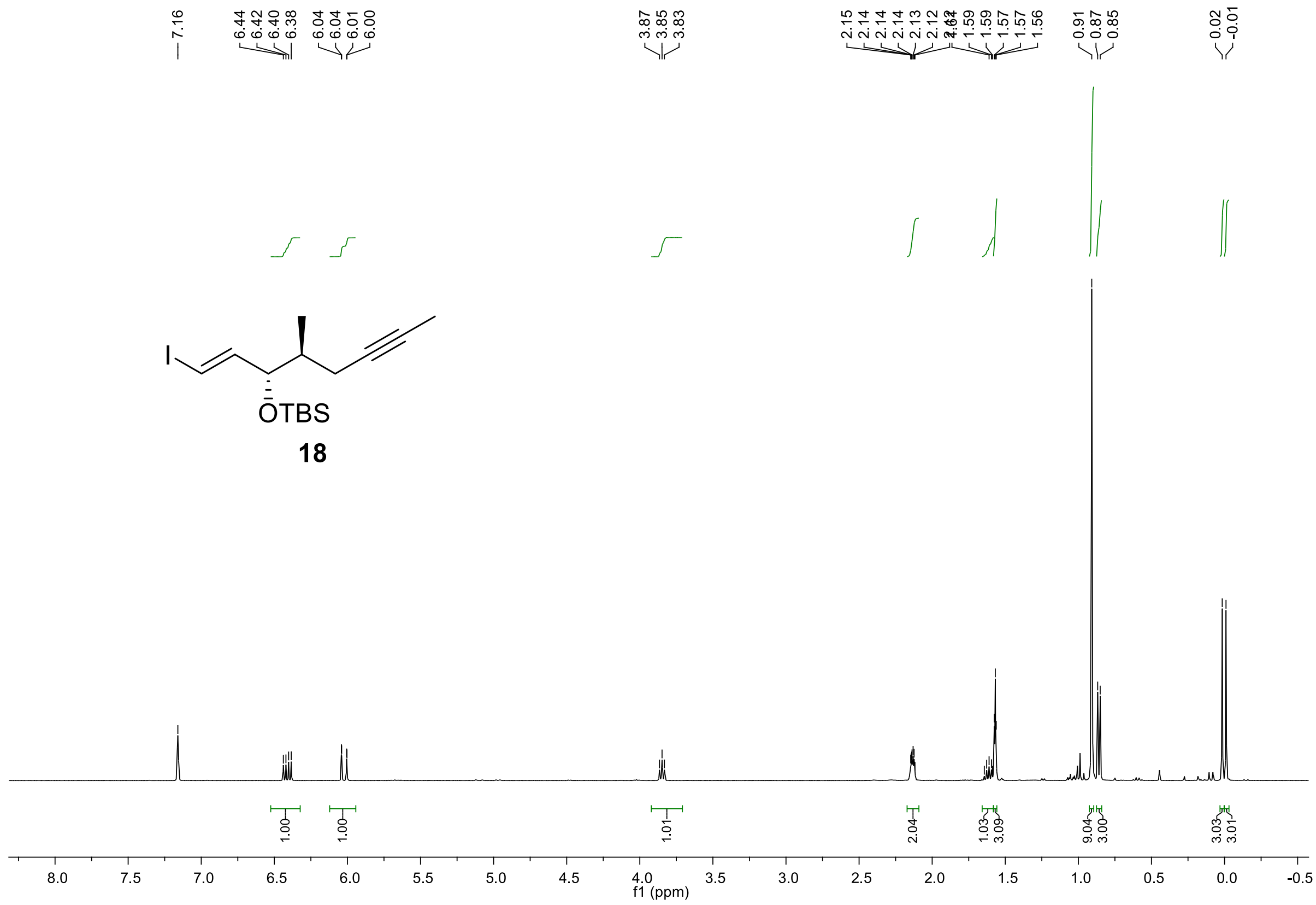

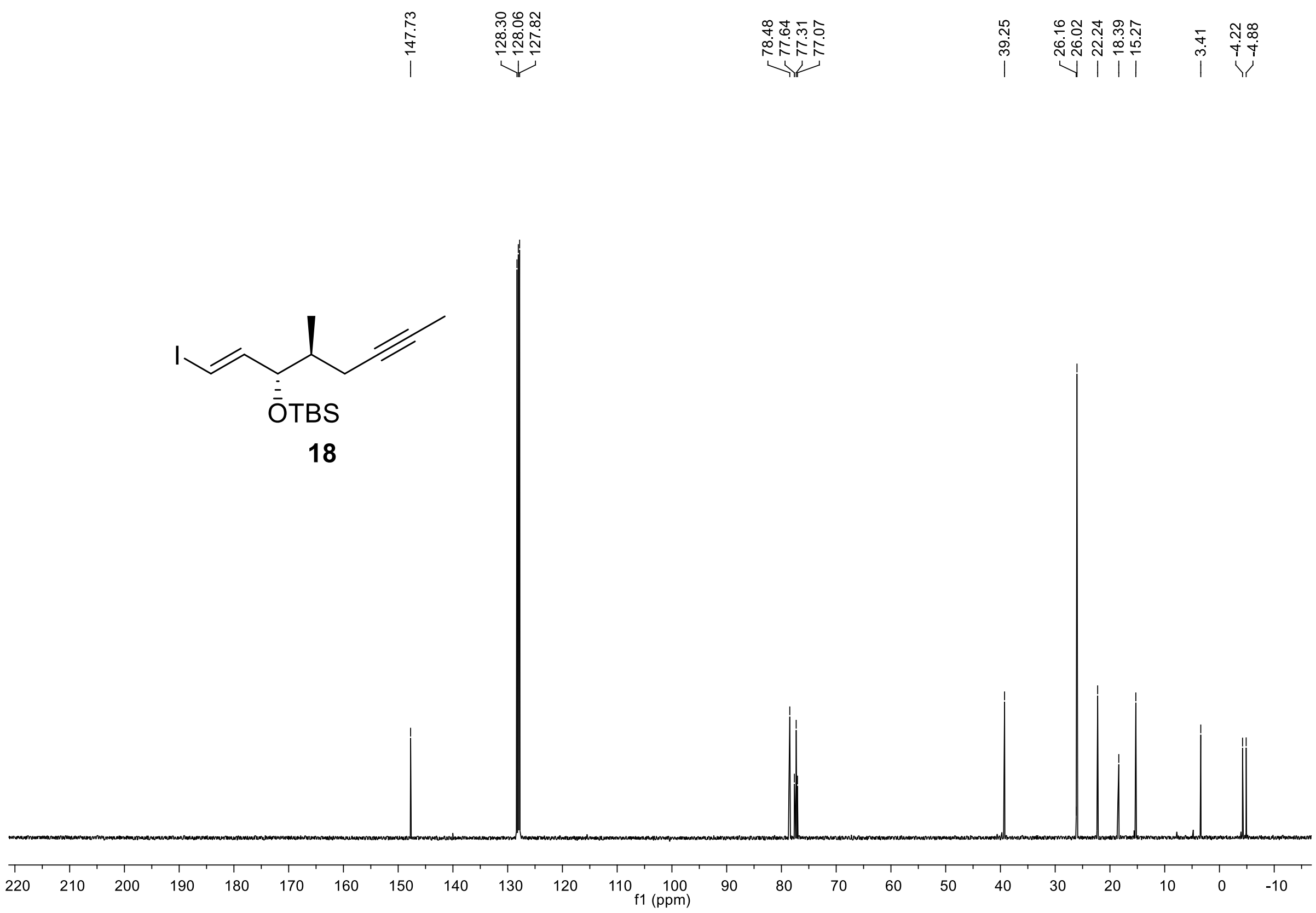

(*R*)-*tert*-Butyldimethyl(oct-1-yn-3-yloxy)silane (S8)

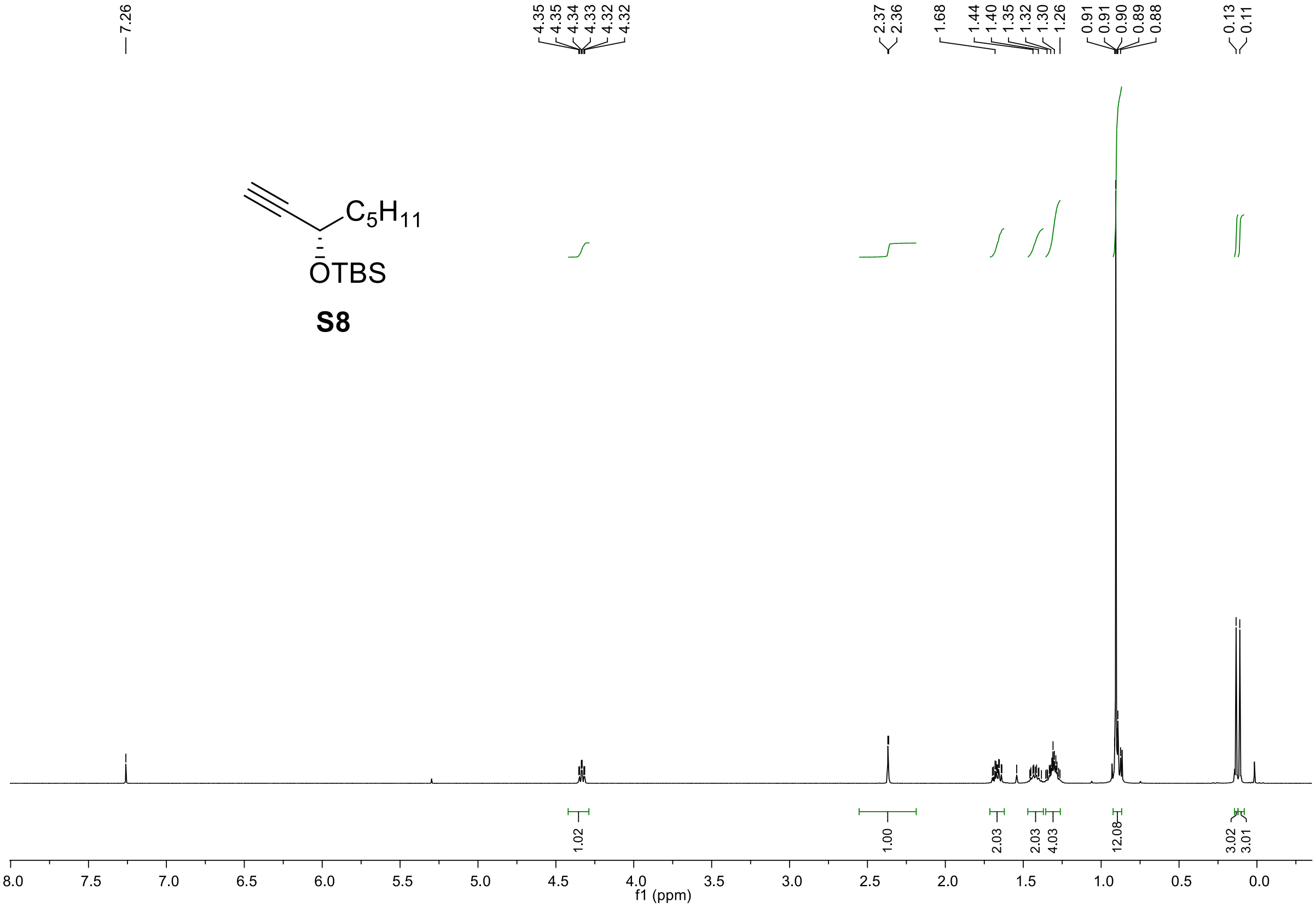

S8

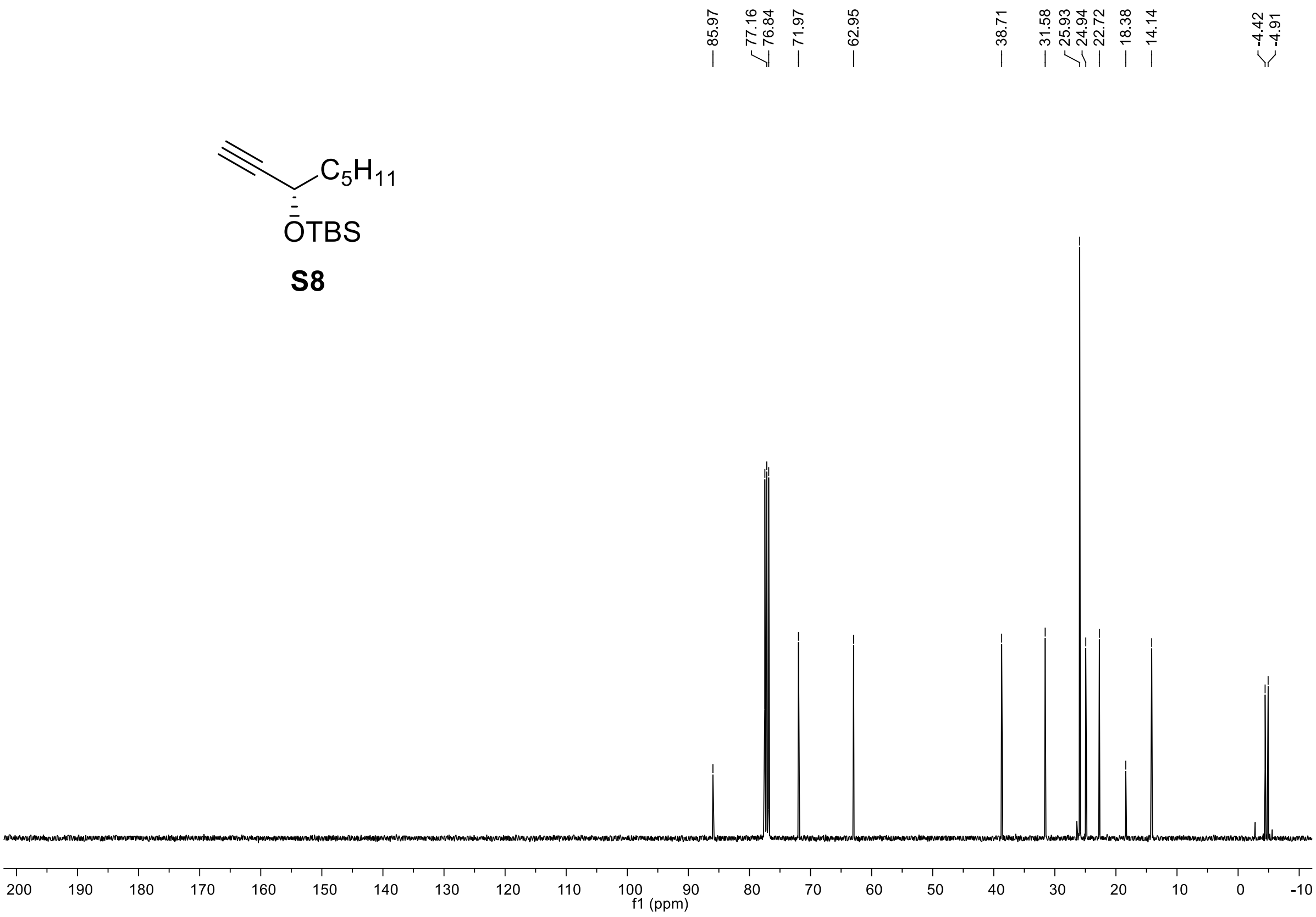

S8

***tert*-Butyldimethyl((3*S*,4*S*)-4-methyl-1-(triethylsilyl)nona-1,6-diyn-3-yloxy)silane (19)**

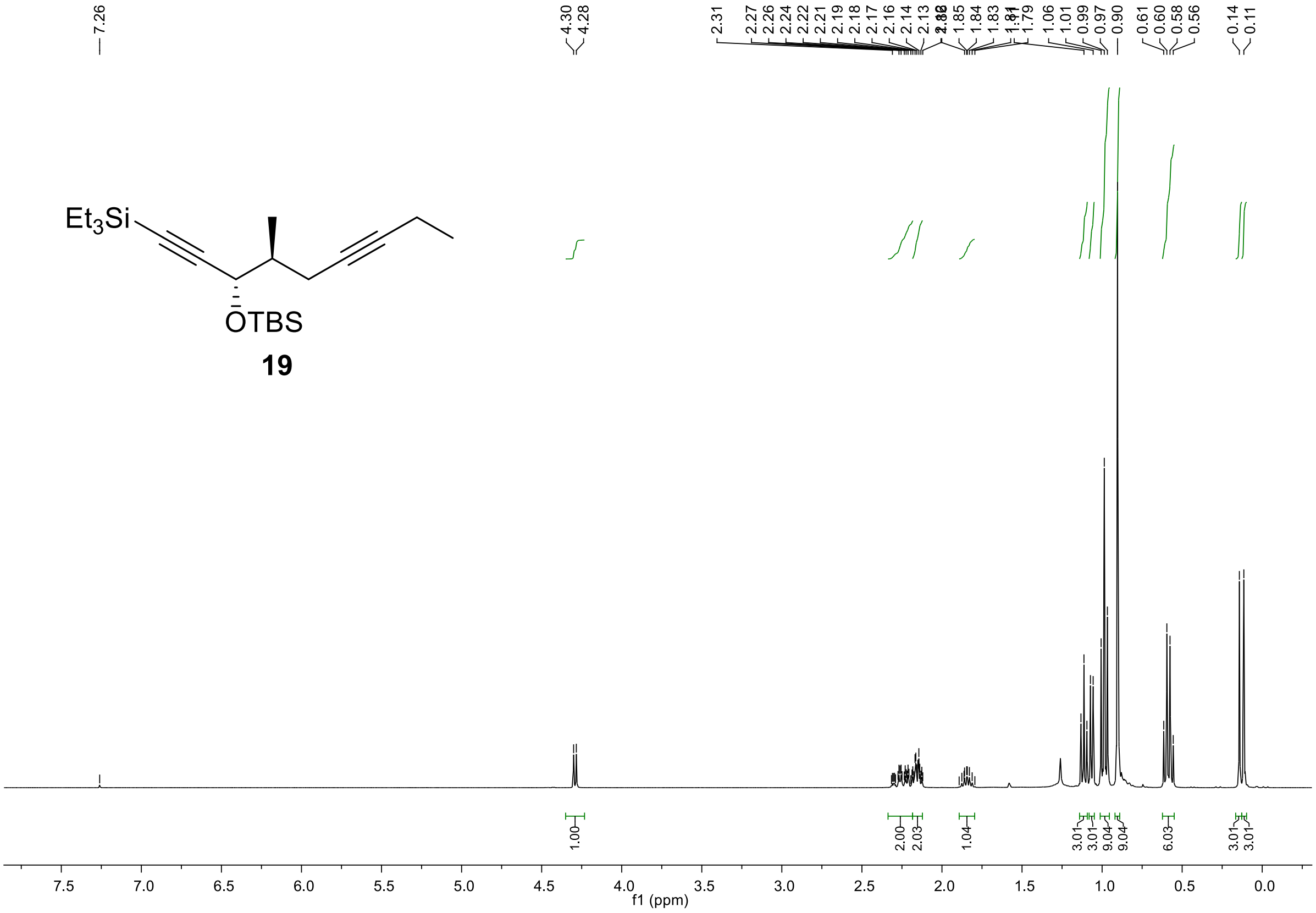

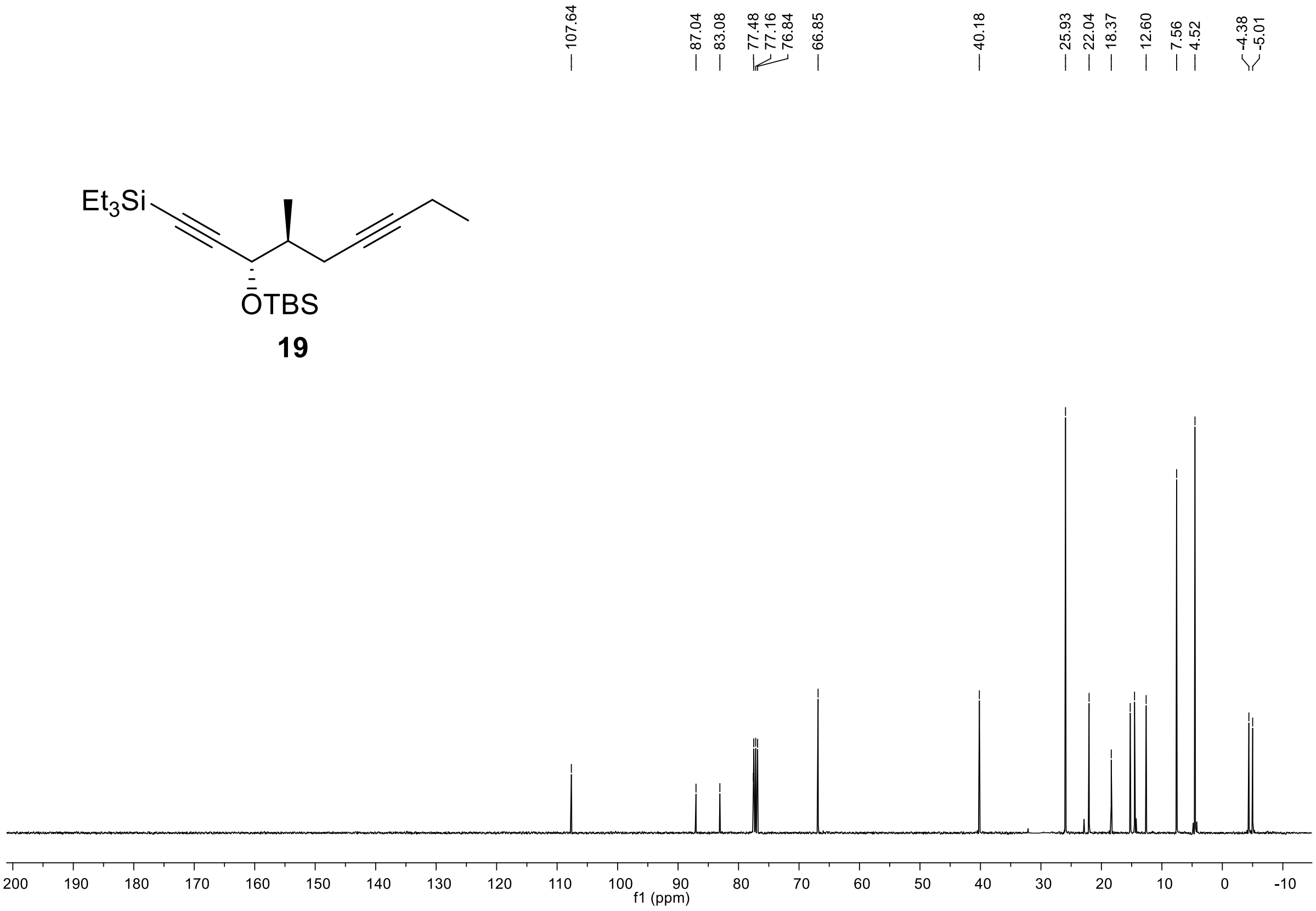

tert-Butyldimethyl((3*S*,4*S*)-4-methylnona-1,6-diyn-3-yloxy)silane (20)

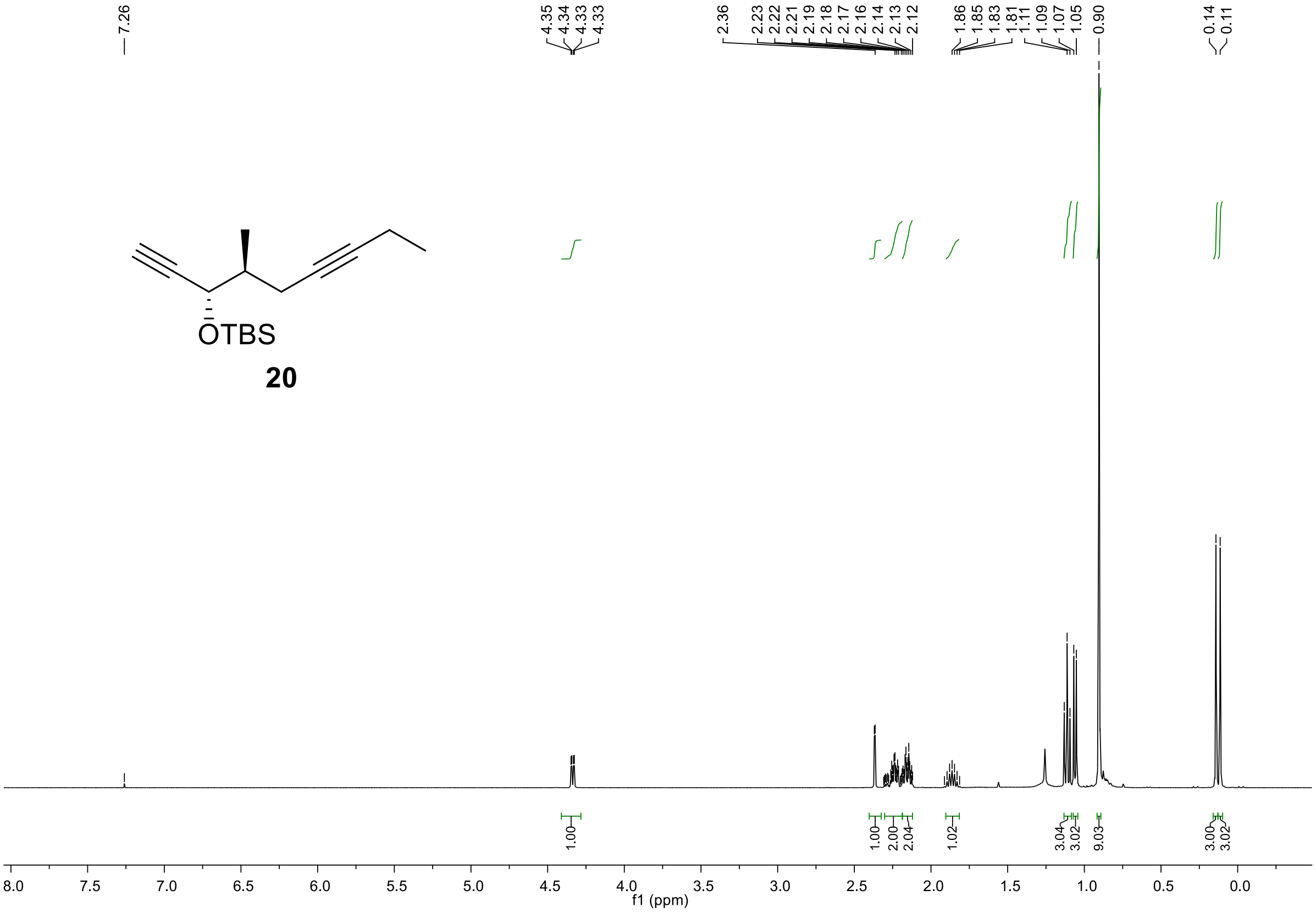

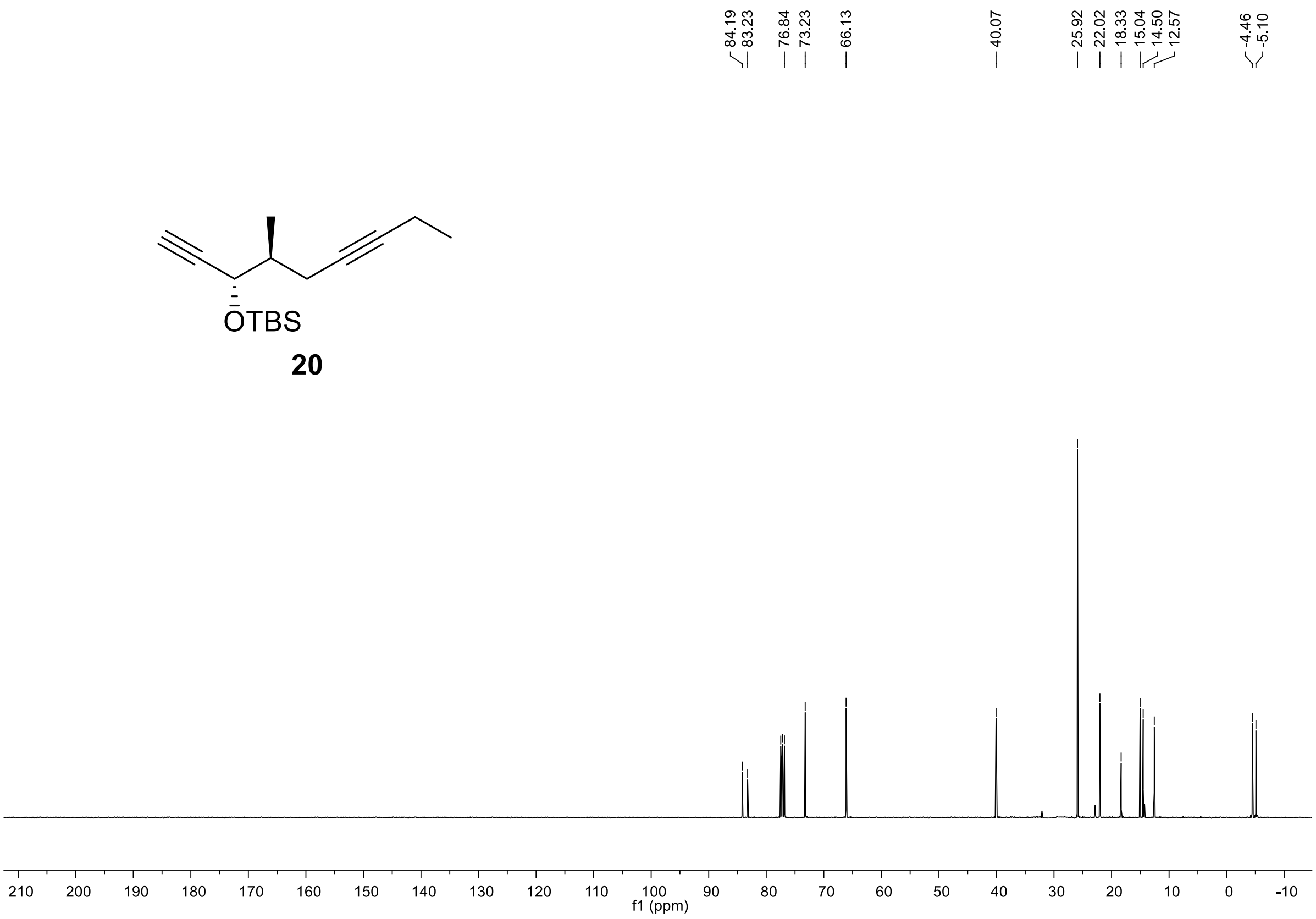

(3a*R*,4*S*,5*R*,6a*S*)-5-(*tert*-Butyldimethylsilyloxy)-4-((*tert*-butyldimethylsilyloxy)methyl)hexahydro-2*H*-cyclopenta[*b*]furan-2-one (S10)

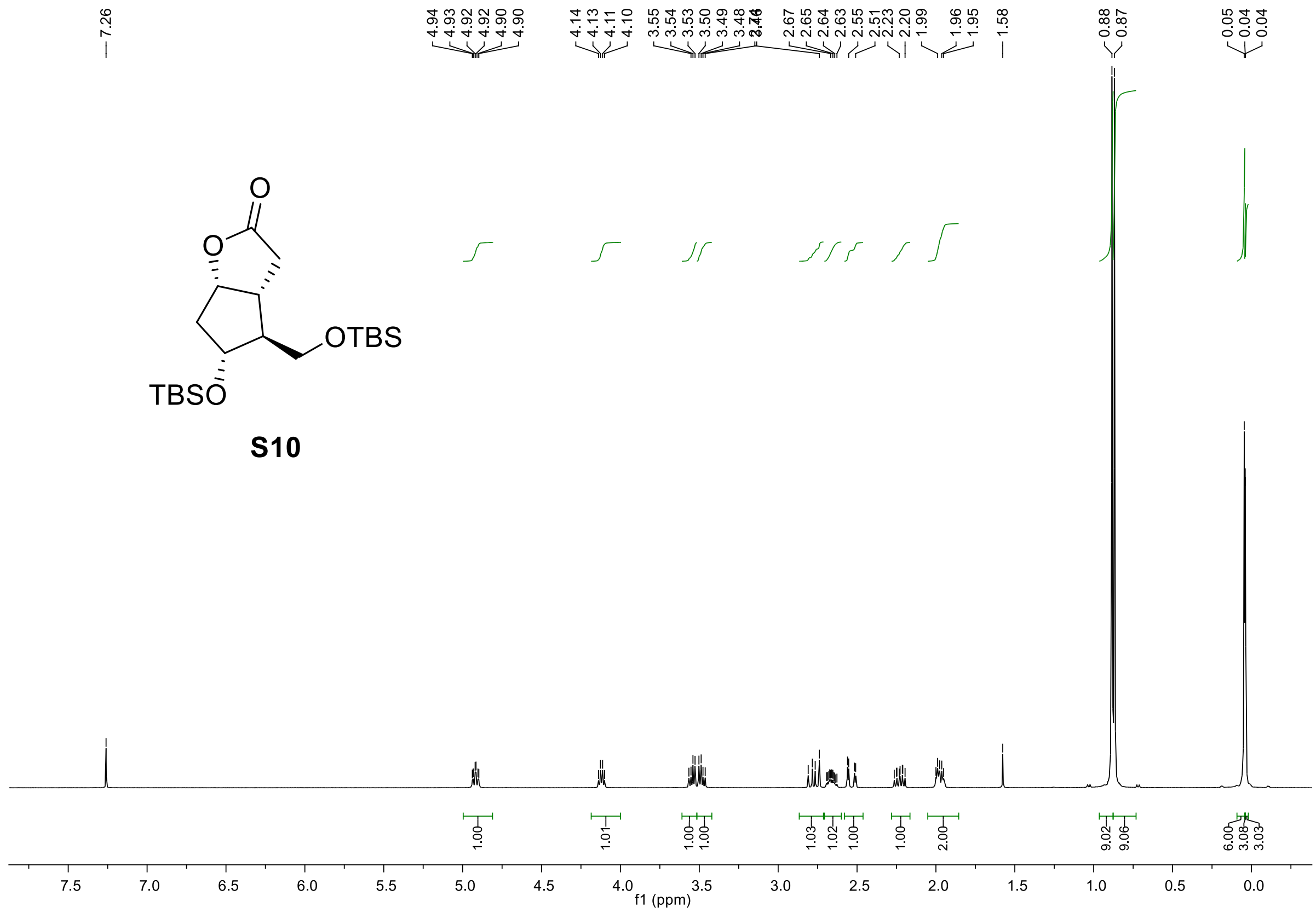

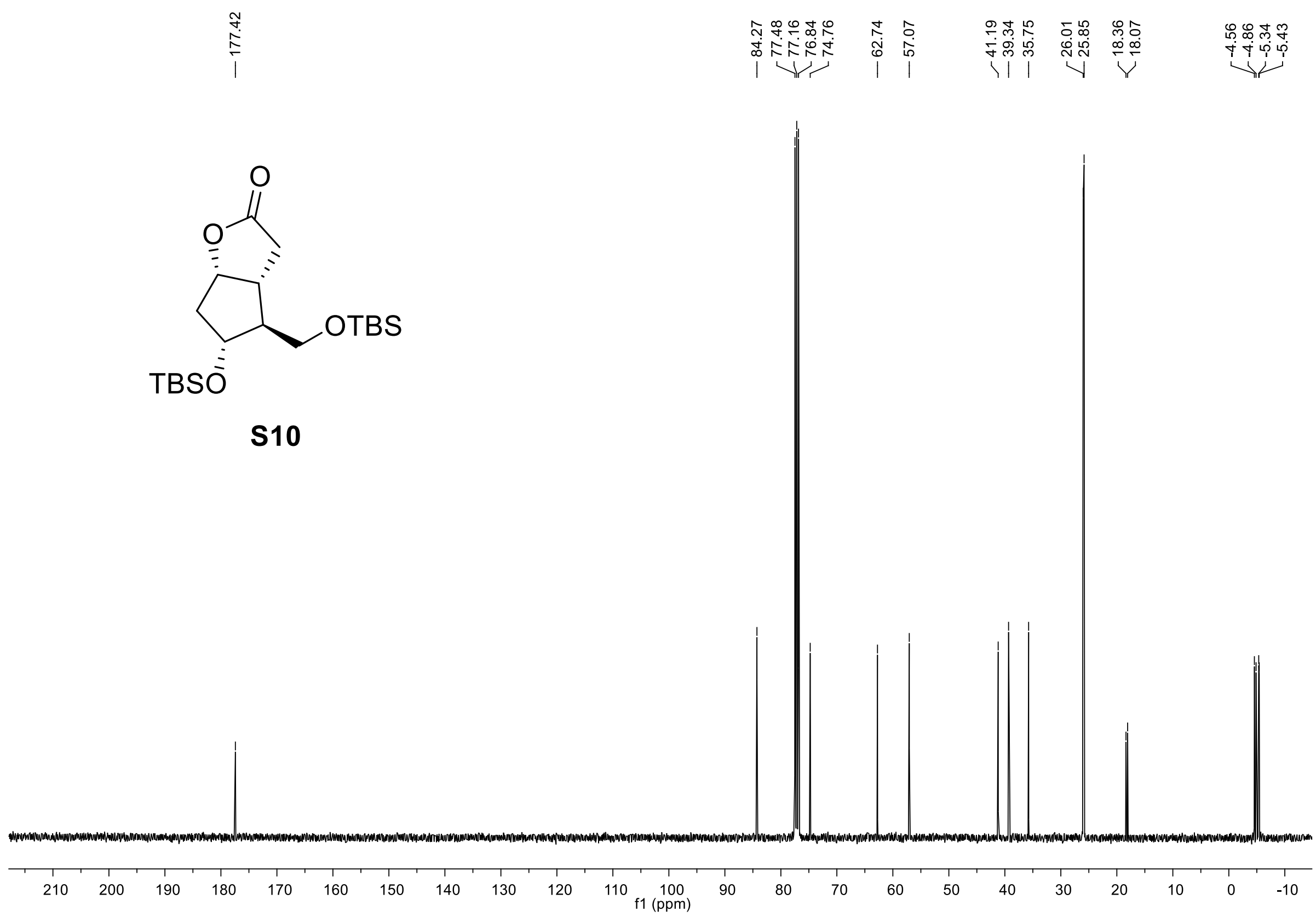

(3*S*,3a*R*,4*S*,5*R*,6a*S*)-5-(*tert*-Butyldimethylsilyloxy)-4-((*tert*-butyldimethylsilyloxy)methyl)-3-fluorohexahydro-2*H*-cyclopenta[*b*]furan-2-one (45)

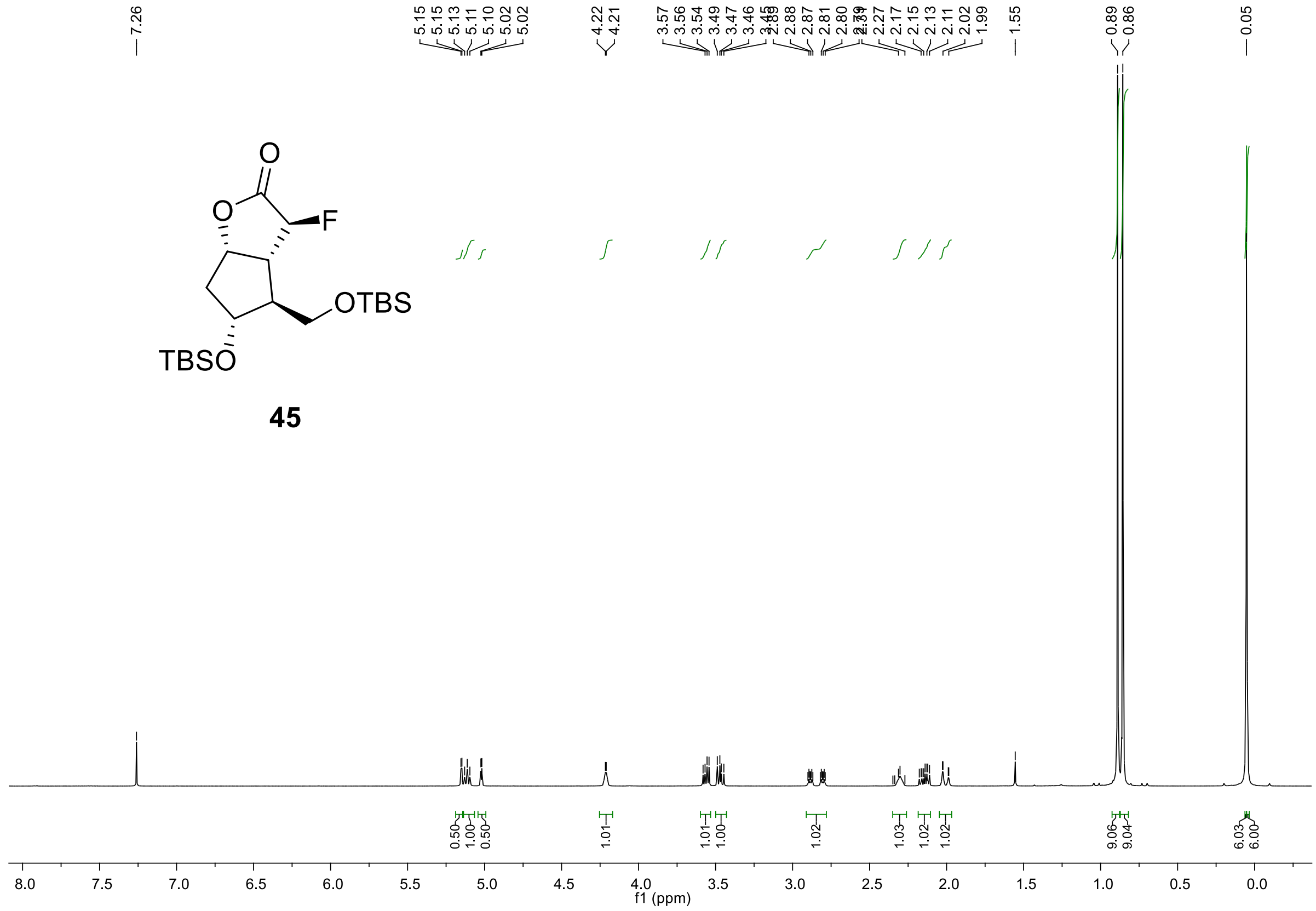

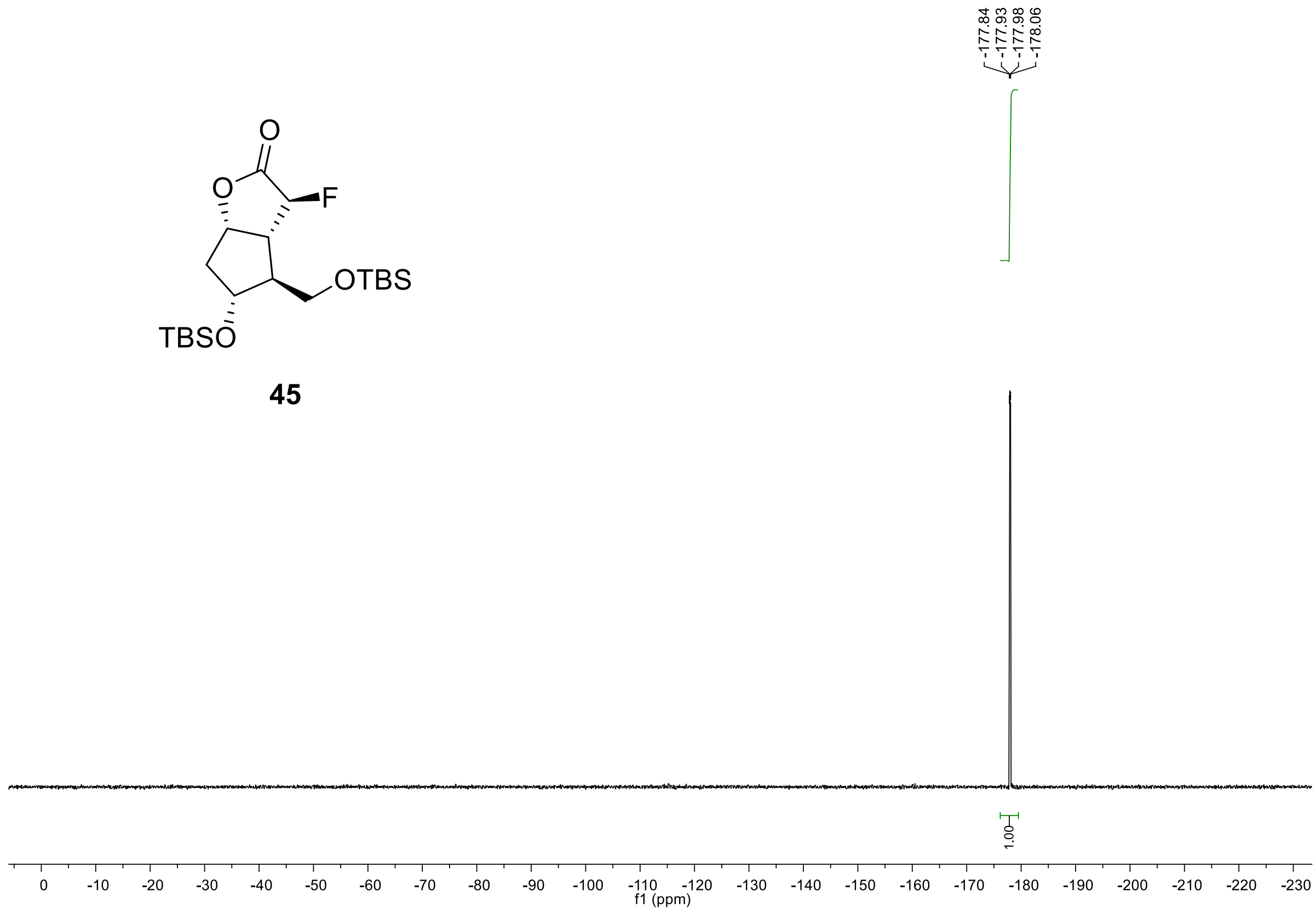

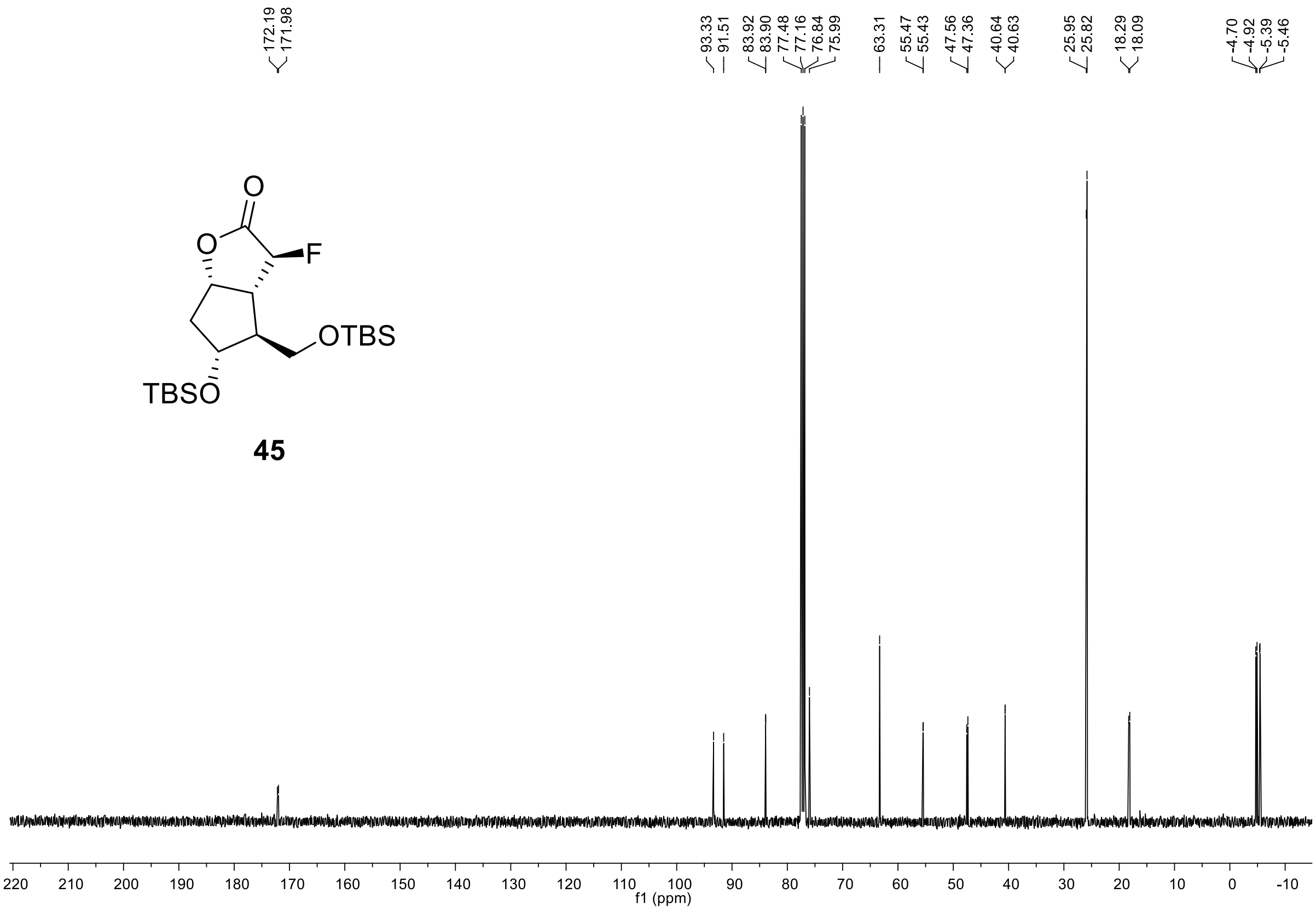
172.19
171.98
93.33
91.51
83.92
83.90
77.48
77.16
76.84
75.99
63.31
55.47
55.43
47.56
47.36
40.64
40.63
25.95
25.82
18.29
18.09
-4.70
-4.92
-5.39
-5.46
220 210 200 190 180 170 160 150 140 130 120 110 100 90 80 70 60 50 40 30 20 10 0 -10
f1 (ppm)

45

5-(Benzo[*d*]thiazol-2-ylsulfonyl)pentanoic acid (S14)

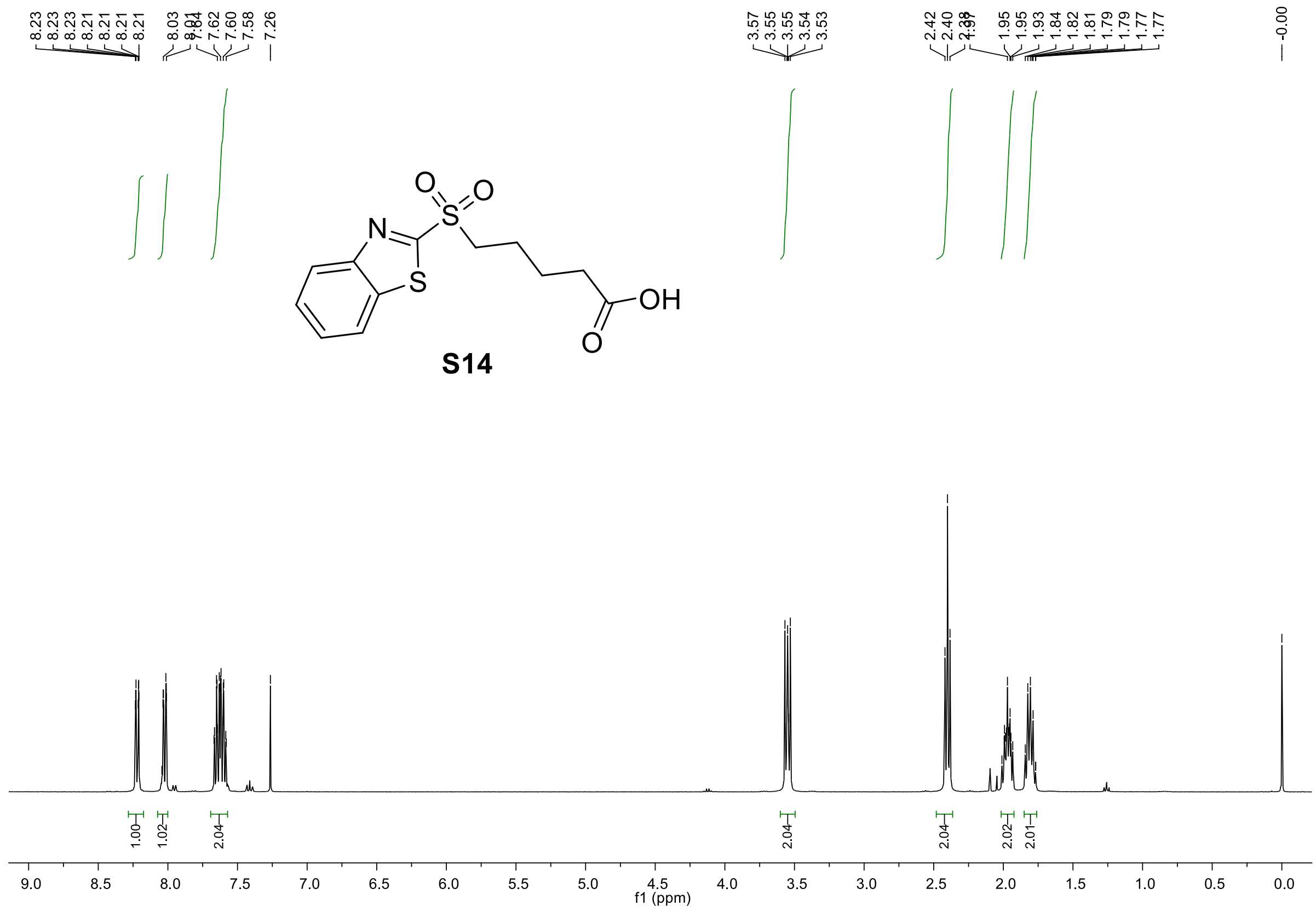

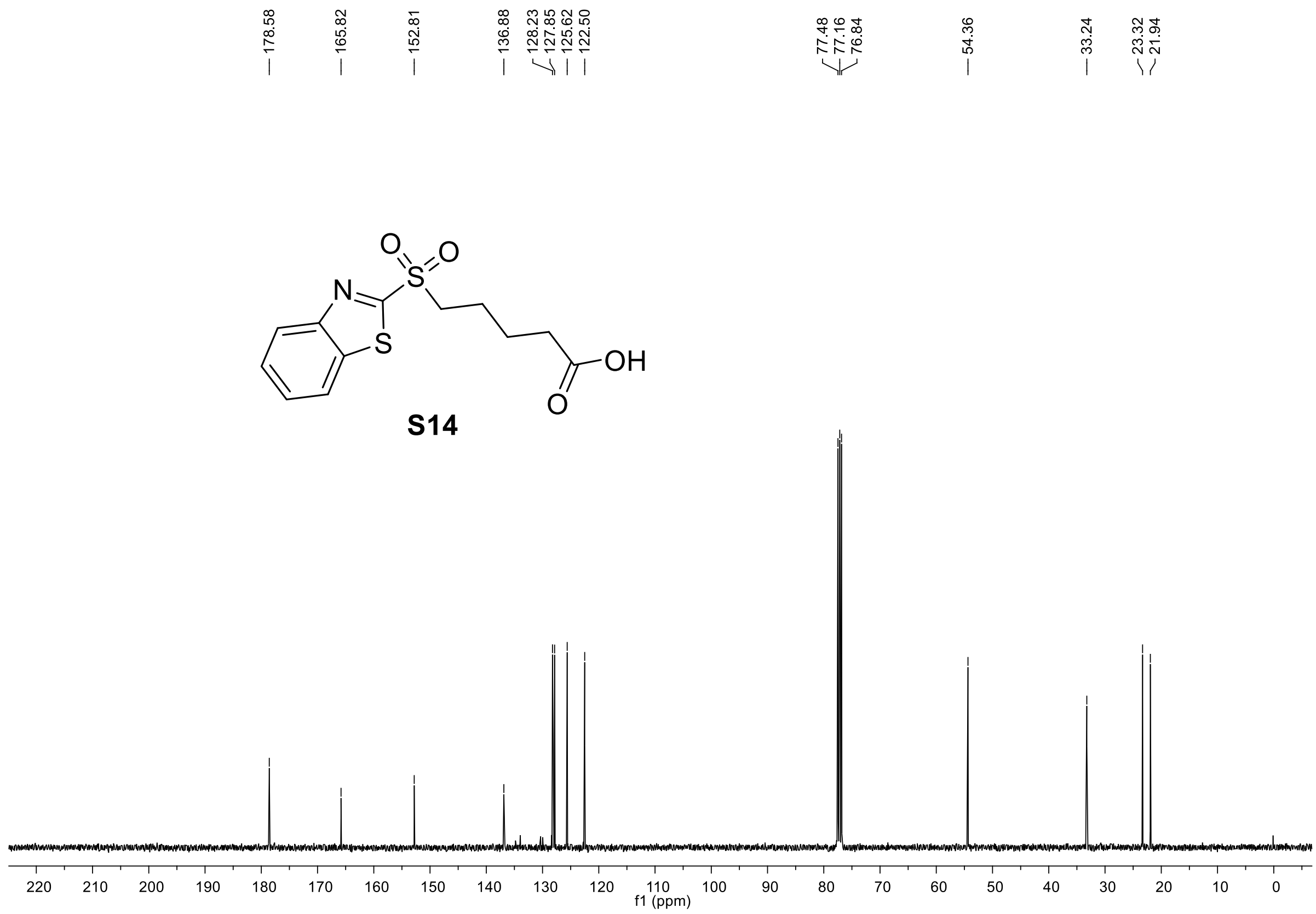

5-((3*S*,3a*R*,4S,5*R*,6a*S*)-5-(*tert*-Butyldimethylsilyloxy)-4-((*tert*-butyldimethylsilyloxy)methyl)-3-fluorohexahydro-2*H*-cyclopenta[*b*]furan-2-ylidene)pentanoic acid (46)

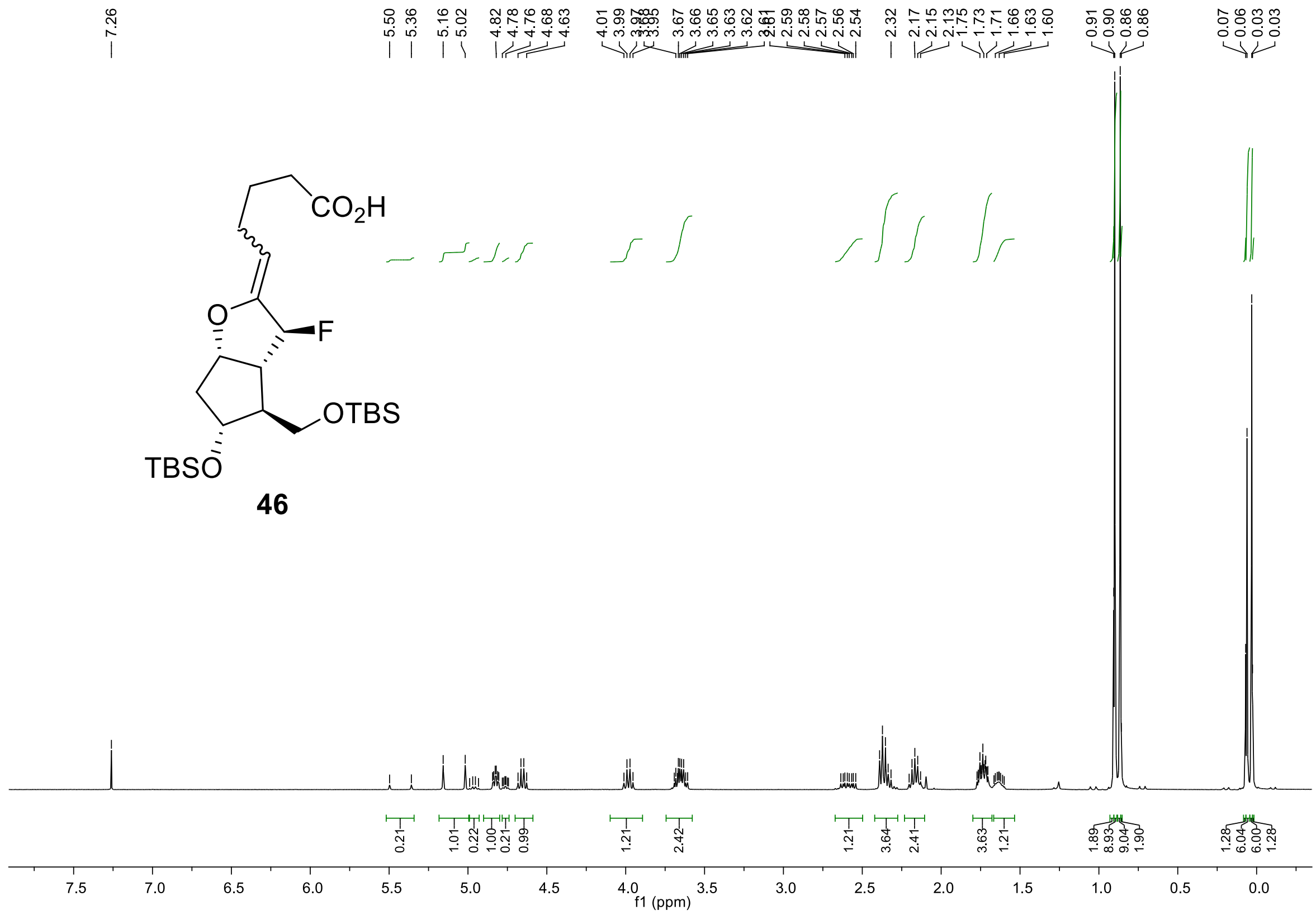

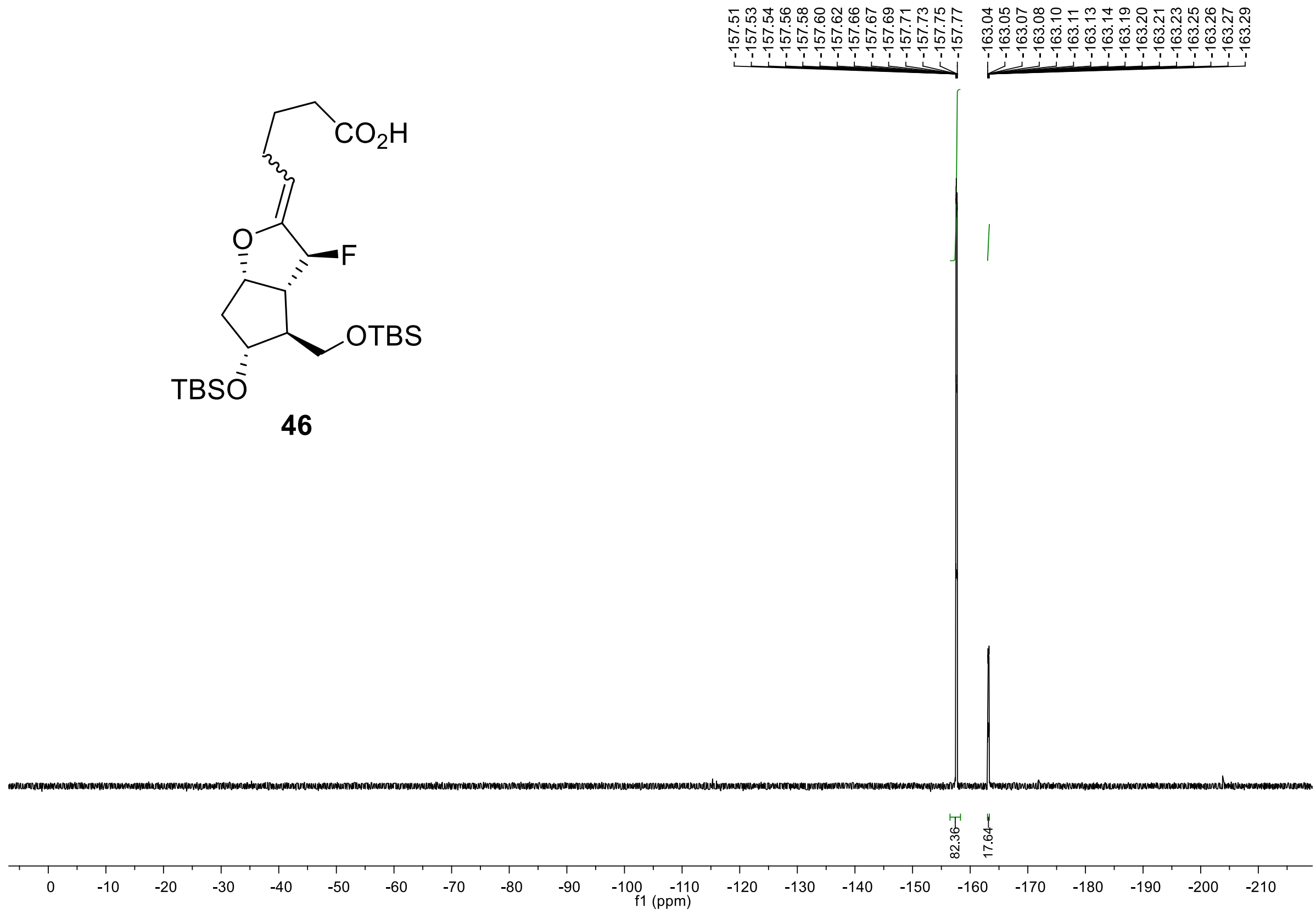

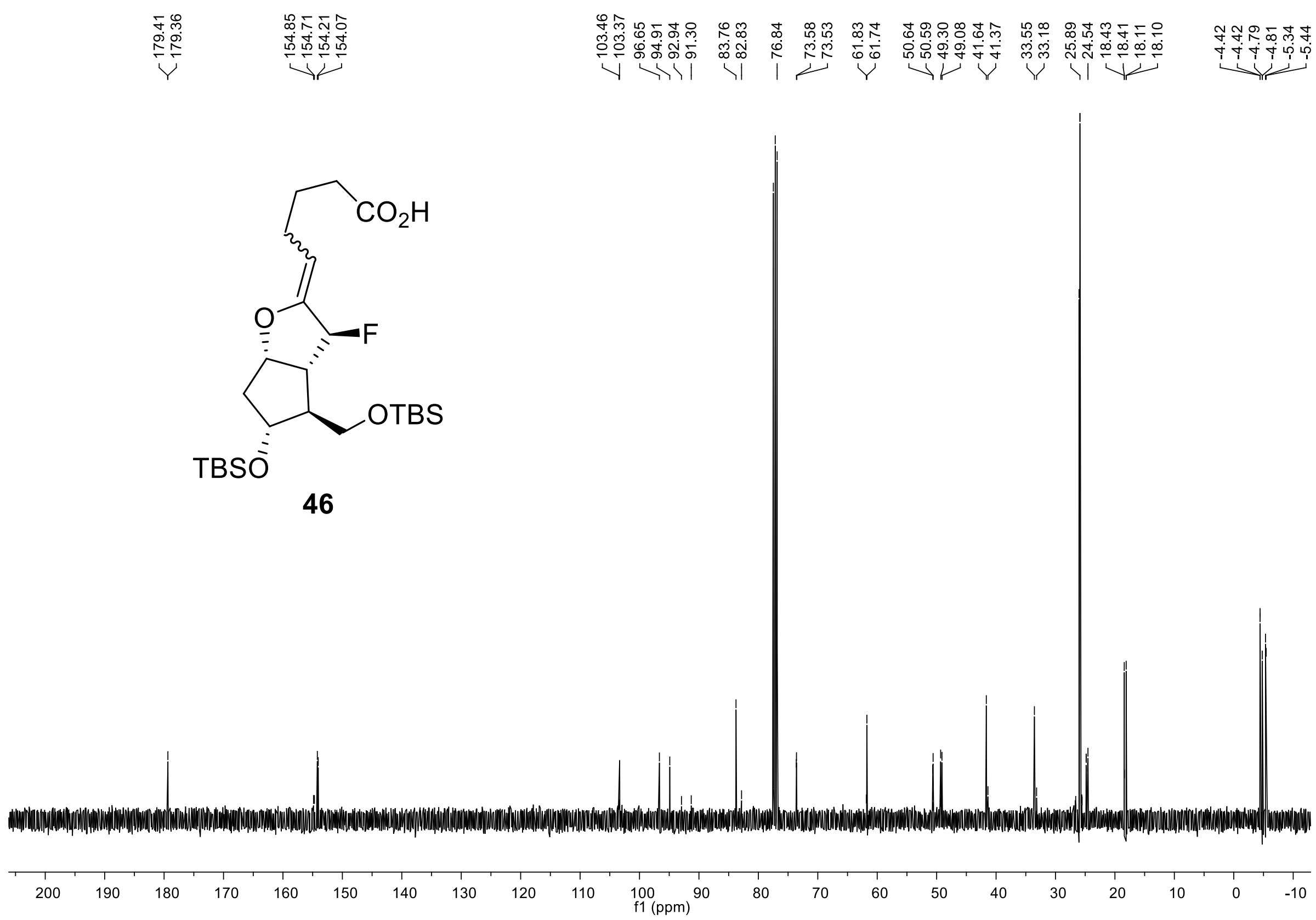
179.41
179.36
154.85
154.71
154.21
154.07
103.46
103.37
96.65
94.91
92.94
91.30
83.76
82.83
76.84
73.58
73.53
61.83
61.74
50.64
50.59
49.30
49.08
41.64
41.37
33.55
33.18
25.89
24.54
18.43
18.41
18.11
18.10
-4.42
-4.42
-4.79
-4.81
-5.34
-5.44
CO2H
O
F
OTBS
TBSO
f1 (ppm)

46

(3a*R*,4*R*,5*R*,6a*S*)-4-((*S*,*E*)-3-((*tert*-Butyldimethylsilyl)oxy)oct-1-enyl)-5-hydroxyhexahydro-2*H*-cyclopenta[*b*]furan-2-one (23)

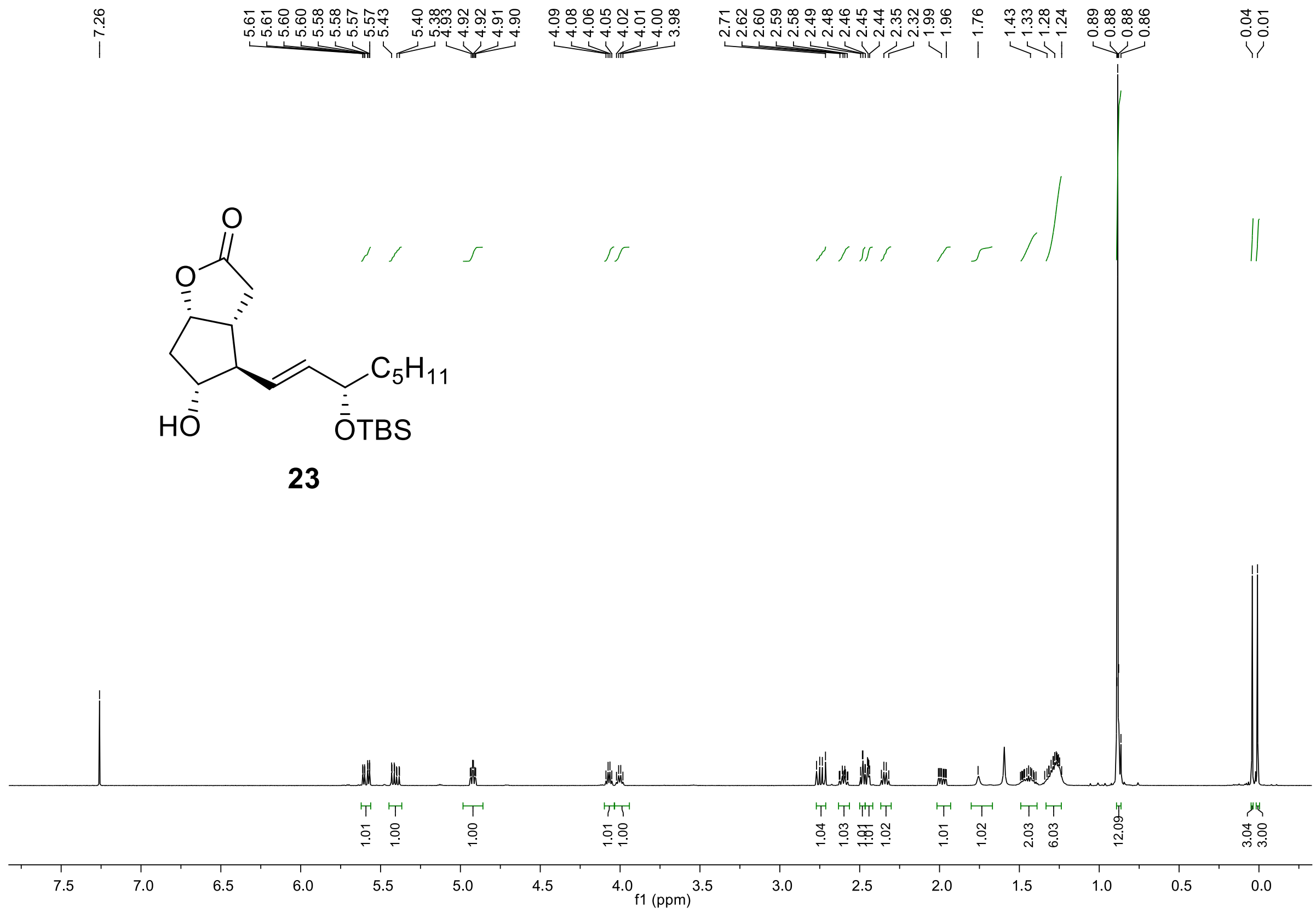

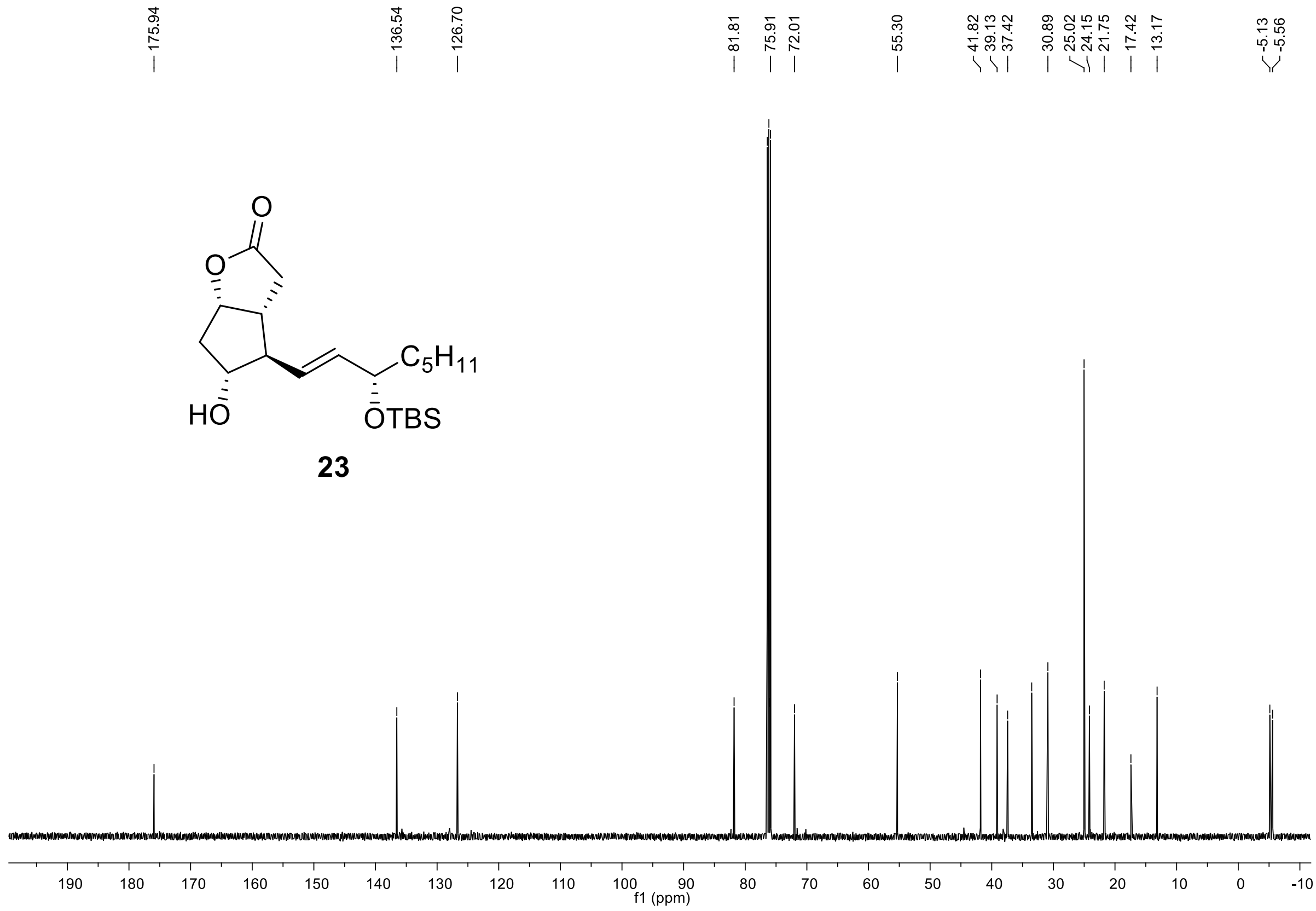

(3a*R*,4*R*,5*R*,6a*S*)-4-((*S*,*E*)-3-(*tert*-Butyldimethylsilyloxy)oct-1-enyl)-5-hydroxyhexahydro-2*H*-cyclopenta[*b*]furan-2-one (24)

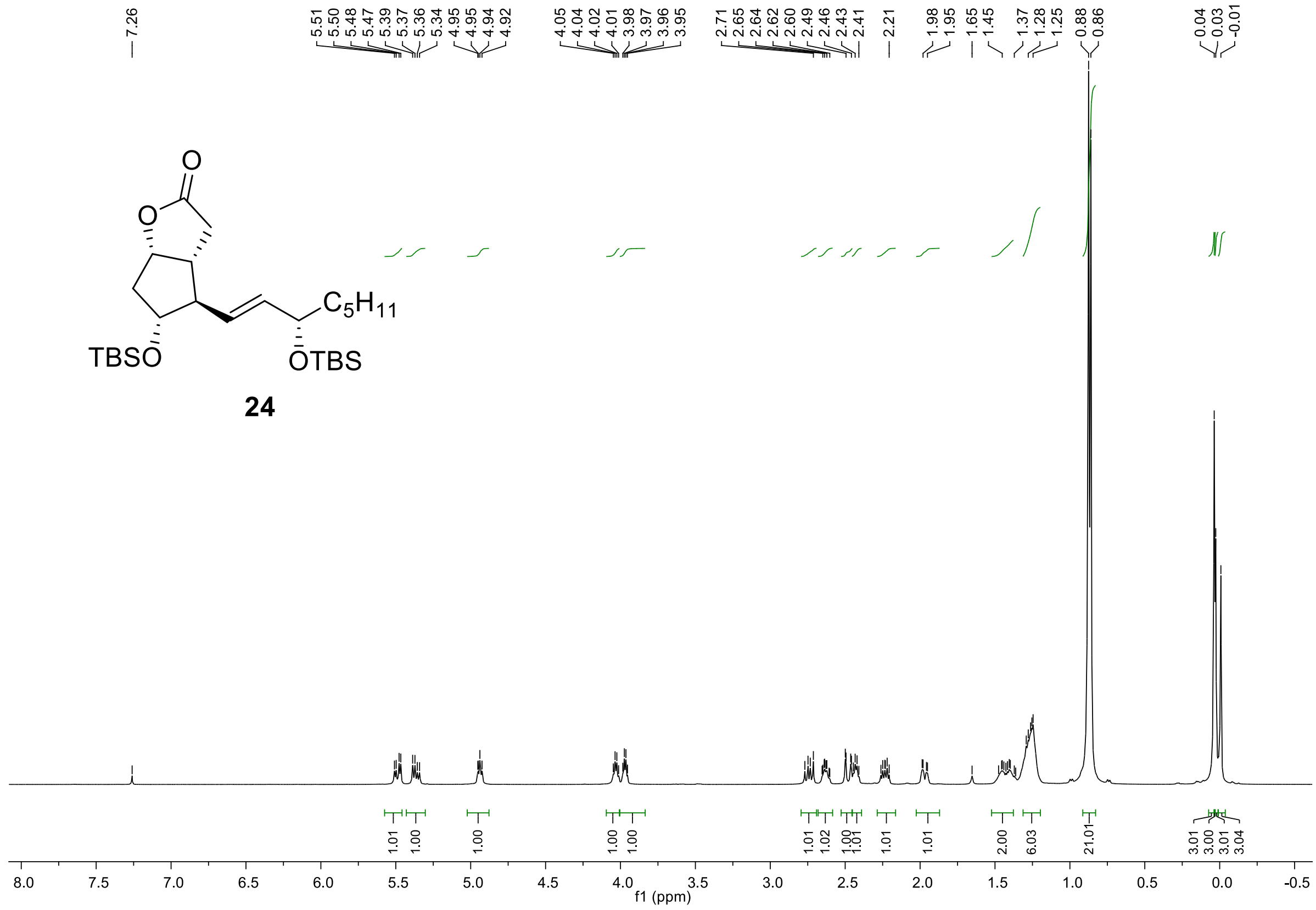

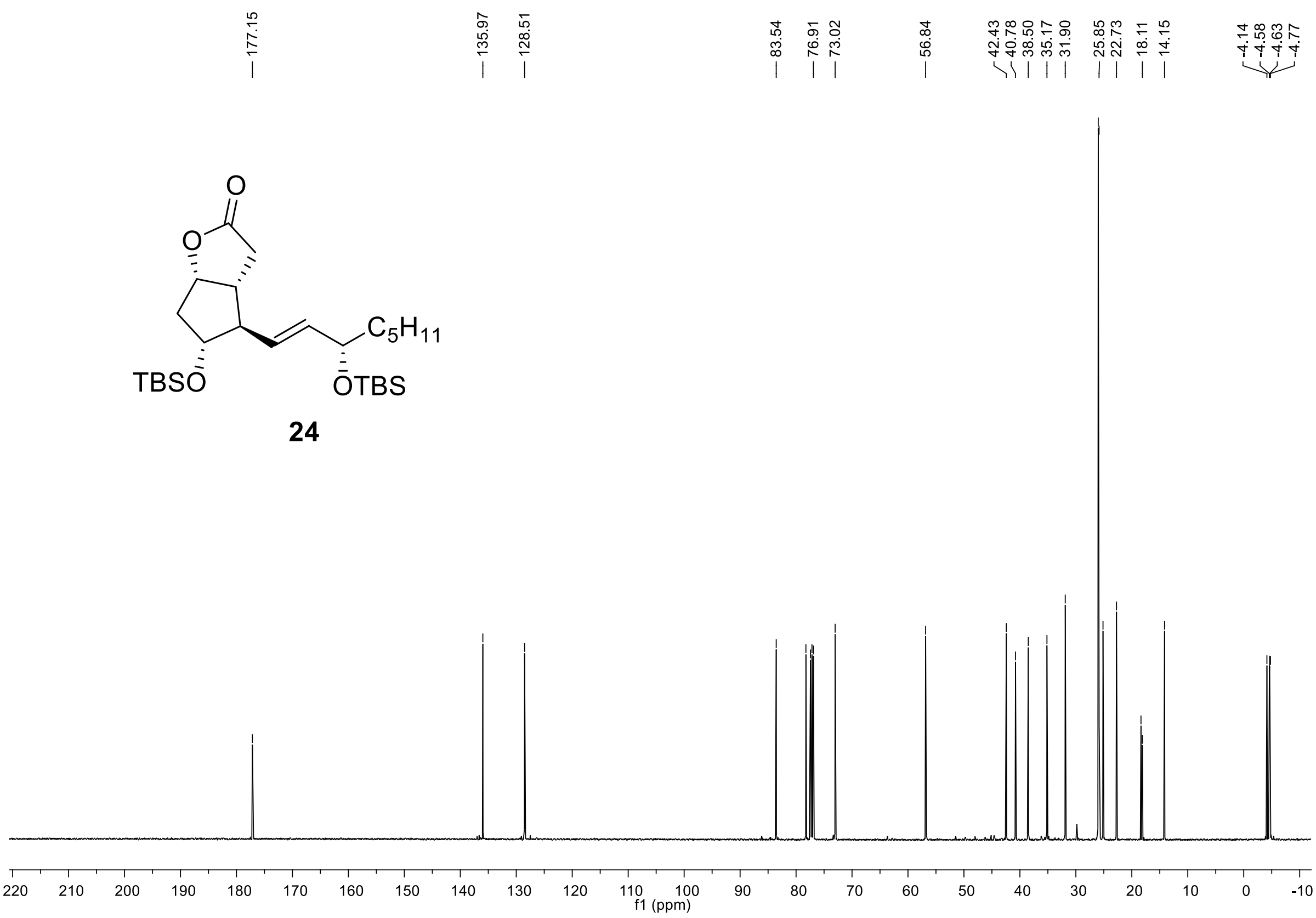

(3a*R*,4*R*,5*R*,6a*S*)-4-((3*S*,4*S*,*E*)-3-(*tert*-Butyldimethylsilyloxy)-4-methyloct-1-en-6-ynyl)-5-hydroxyhexahydro-2*H*-cyclopenta[*b*]furan-2-one (27)

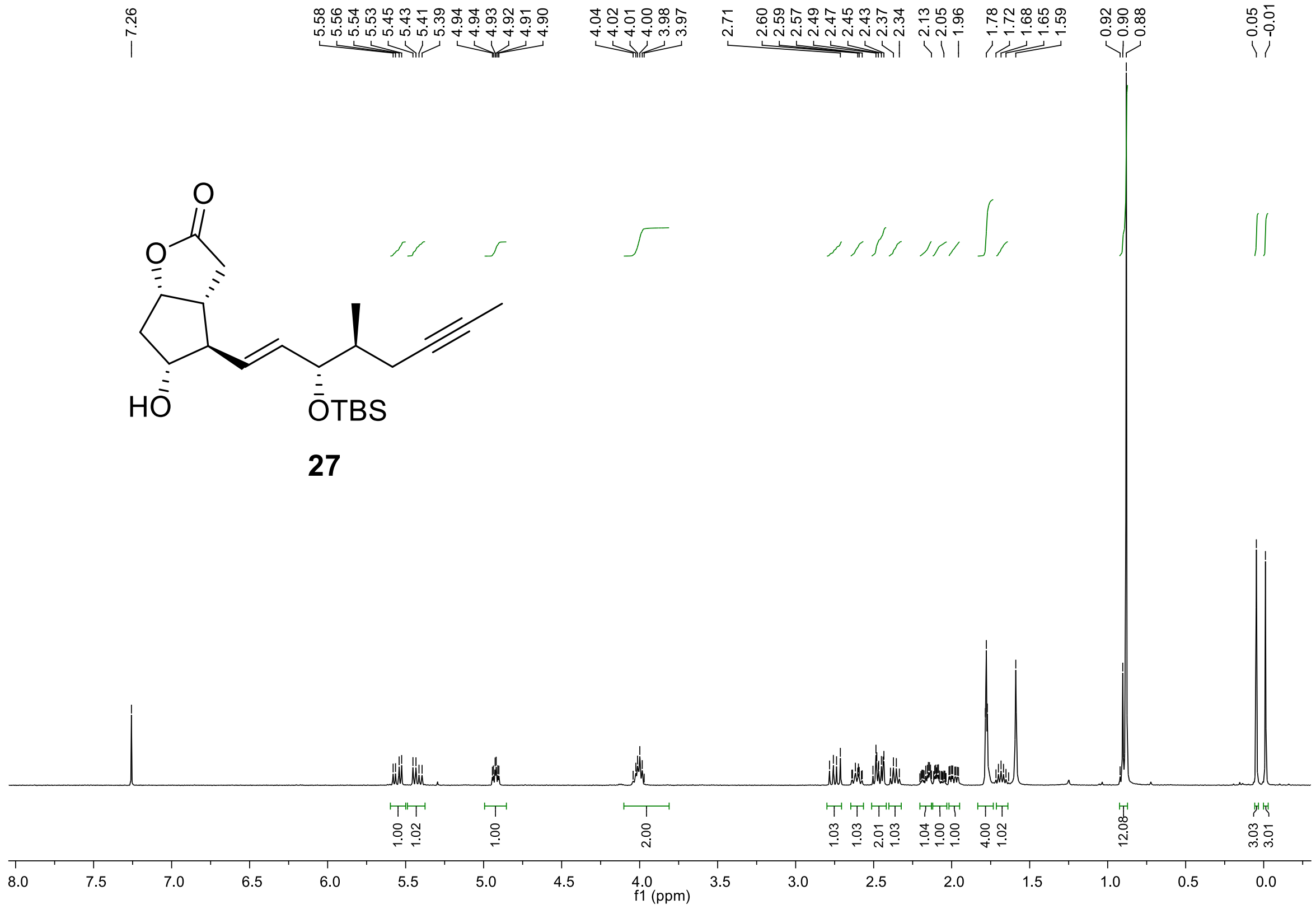

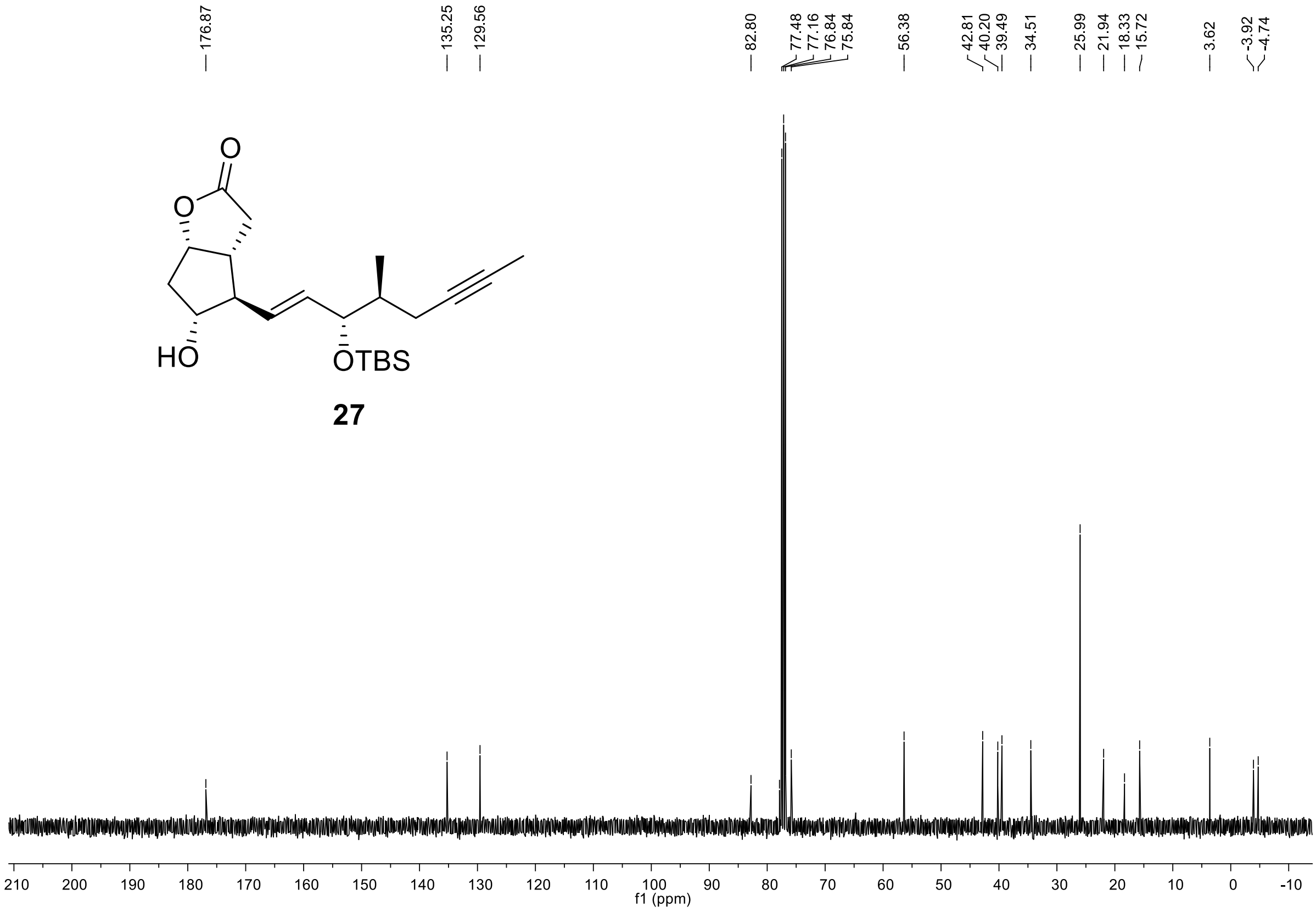

(3a*R*,4*R*,5*R*,6a*S*)-5-(*tert*-Butyldimethylsilyloxy)-4-((3*S*,4*S*,*E*)-3-(*tert*-butyldimethylsilyloxy)-4-methyloct-1-en-6-ynyl)hexahydro-2*H*-cyclopenta[*b*]furan-2-one (28)

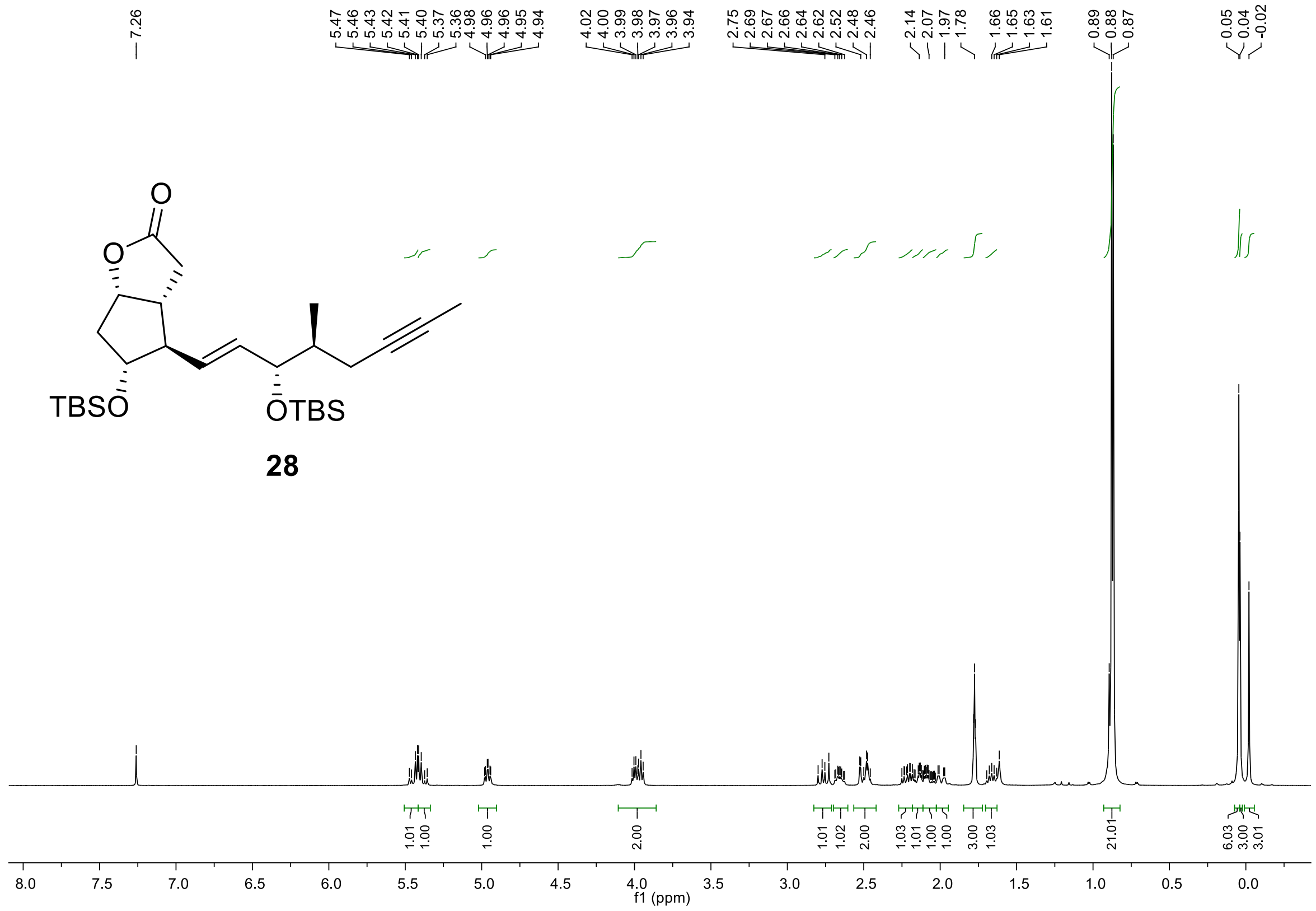

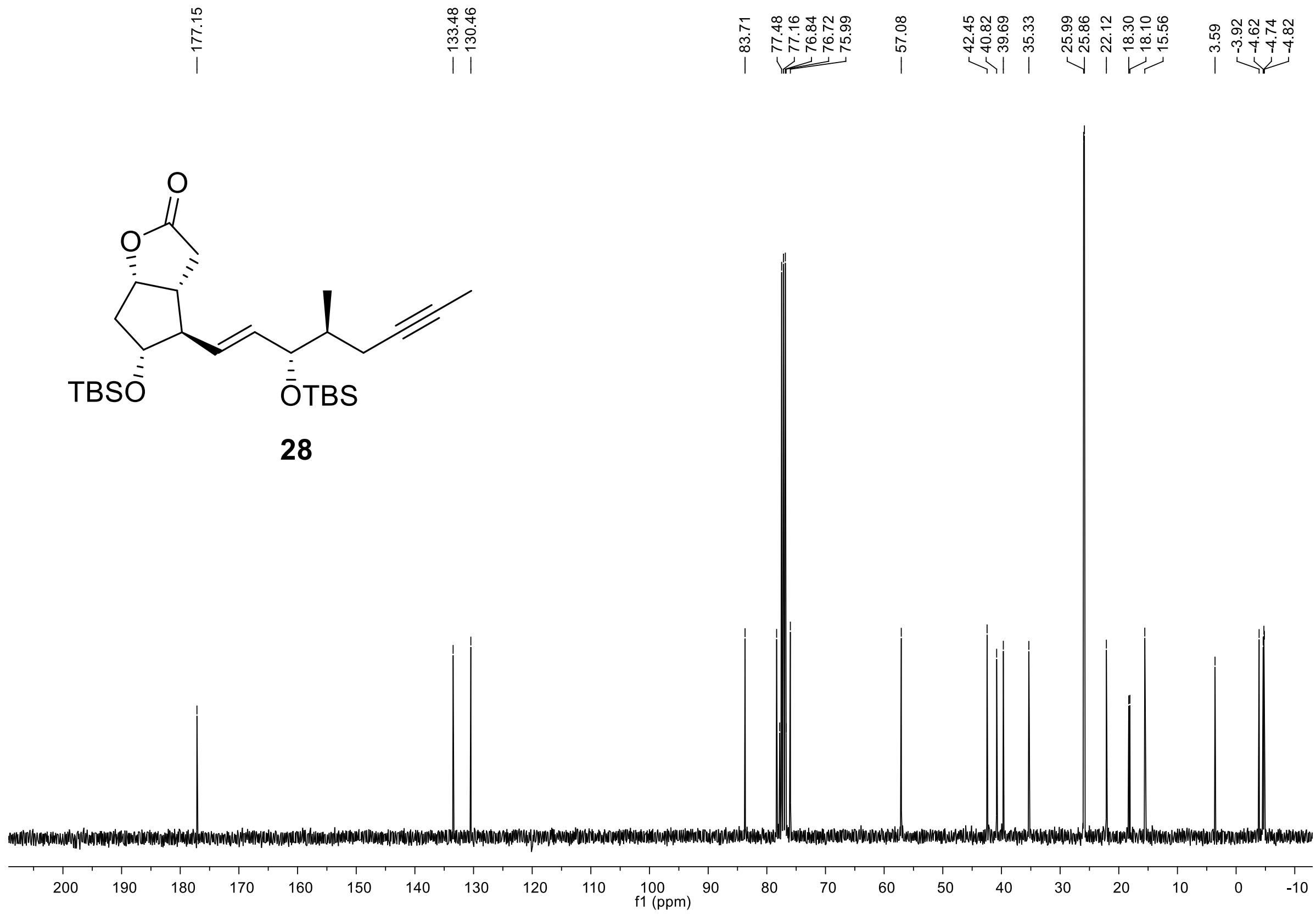

(3a*R*,4*R*,5*R*,6a*S*)-5-(*tert*-Butyldimethylsilyloxy)-4-((*S*,*E*)-3-(*tert*-butyldimethylsilyloxy)oct-1-enyl)-3,3-difluorohexahydro-2*H*-cyclopenta[*b*]furan-2-one (37)

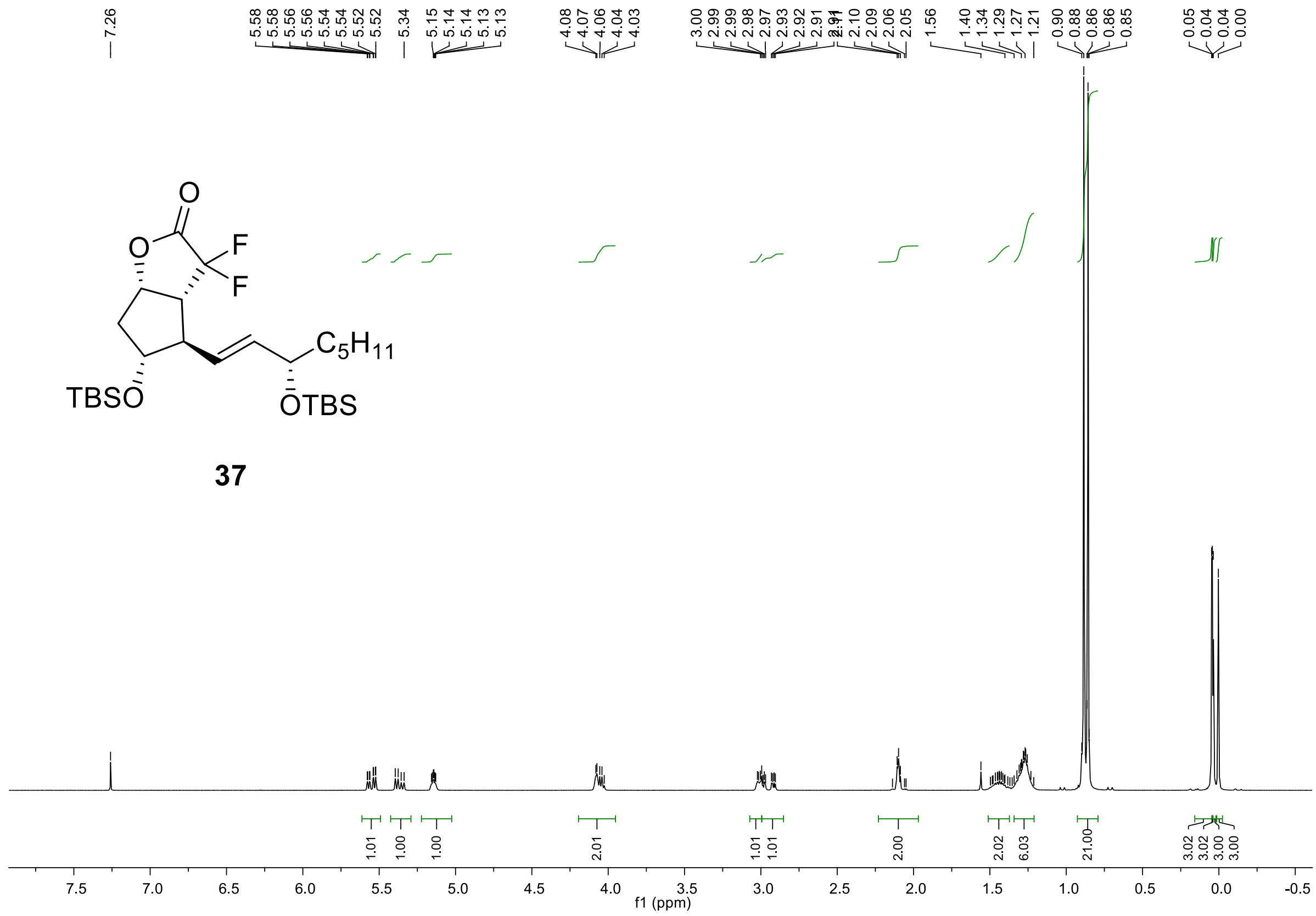

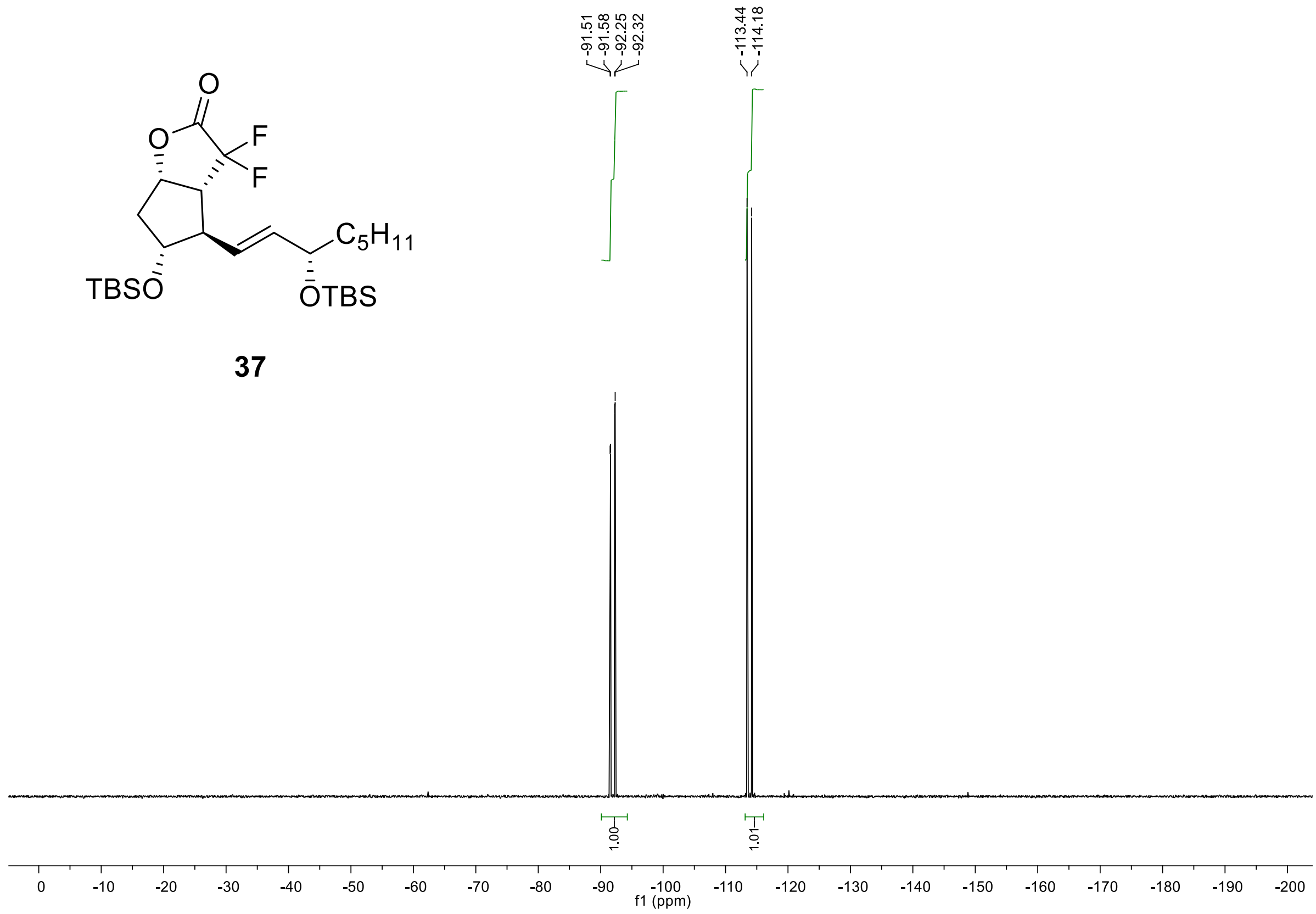

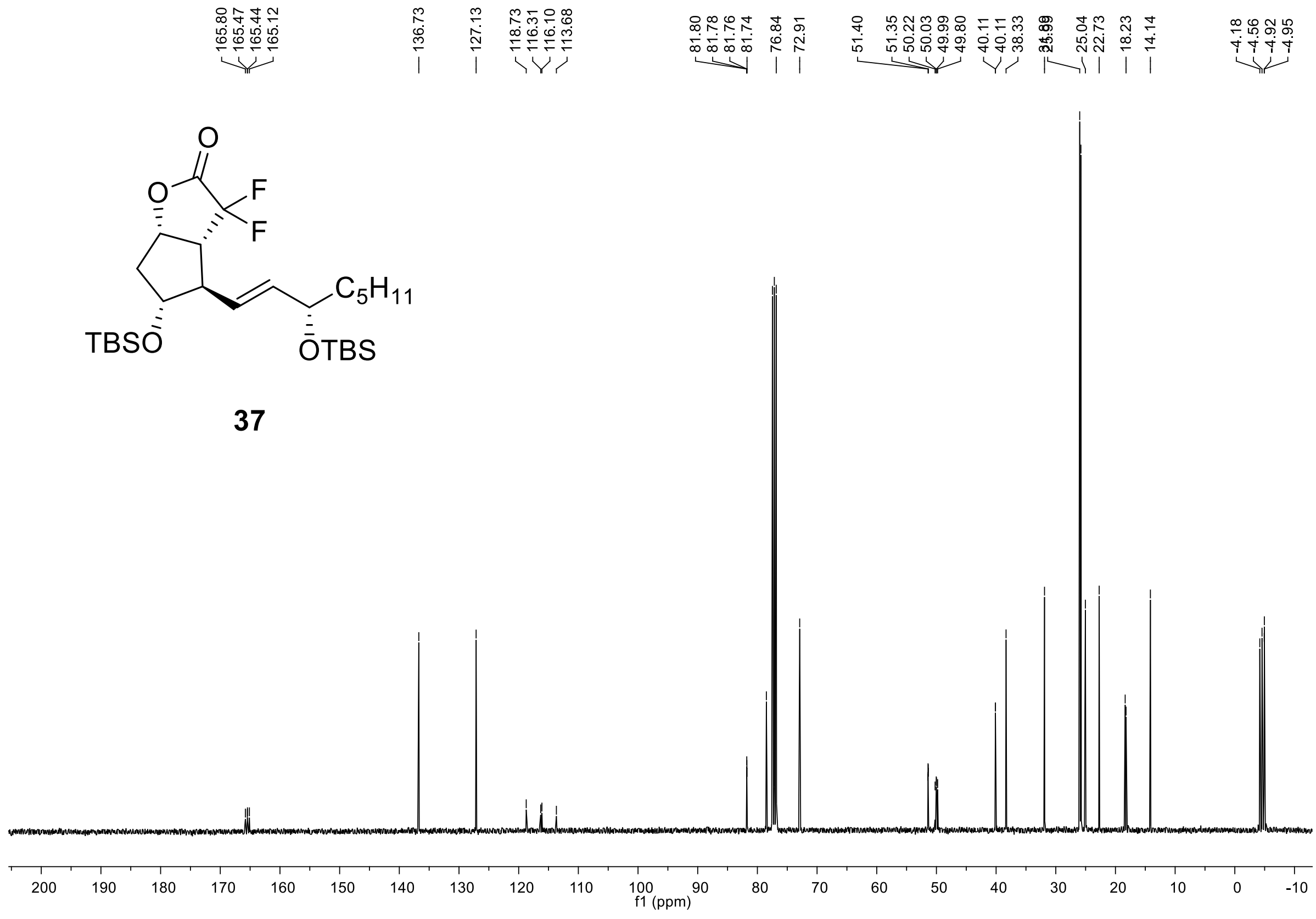

O
F
F
C5H11
TBSO
OTBS
37
165.80
165.47
165.44
165.12
136.73
127.13
118.73
116.31
116.10
113.68
81.80
81.78
81.76
81.74
76.84
72.91
51.40
51.35
50.22
50.03
49.99
49.80
40.11
40.11
38.33
25.04
22.73
18.23
14.14
-4.18
-4.56
-4.92
-4.95
200 190 180 170 160 150 140 130 120 110 100 90 80 70 60 50 40 30 20 10 0 -10
f1 (ppm)

5-((3a*R*,4*R*,5*R*,6a*S*)-5-(*tert*-Butyldimethylsilyloxy)-4-((*S*,*E*)-3-(*tert*-butyldimethylsilyloxy)oct-1-enyl)-3,3-difluorohexahydro-2*H*-cyclopenta[*b*]furan-2-ylidene)pentanoic acid (38)

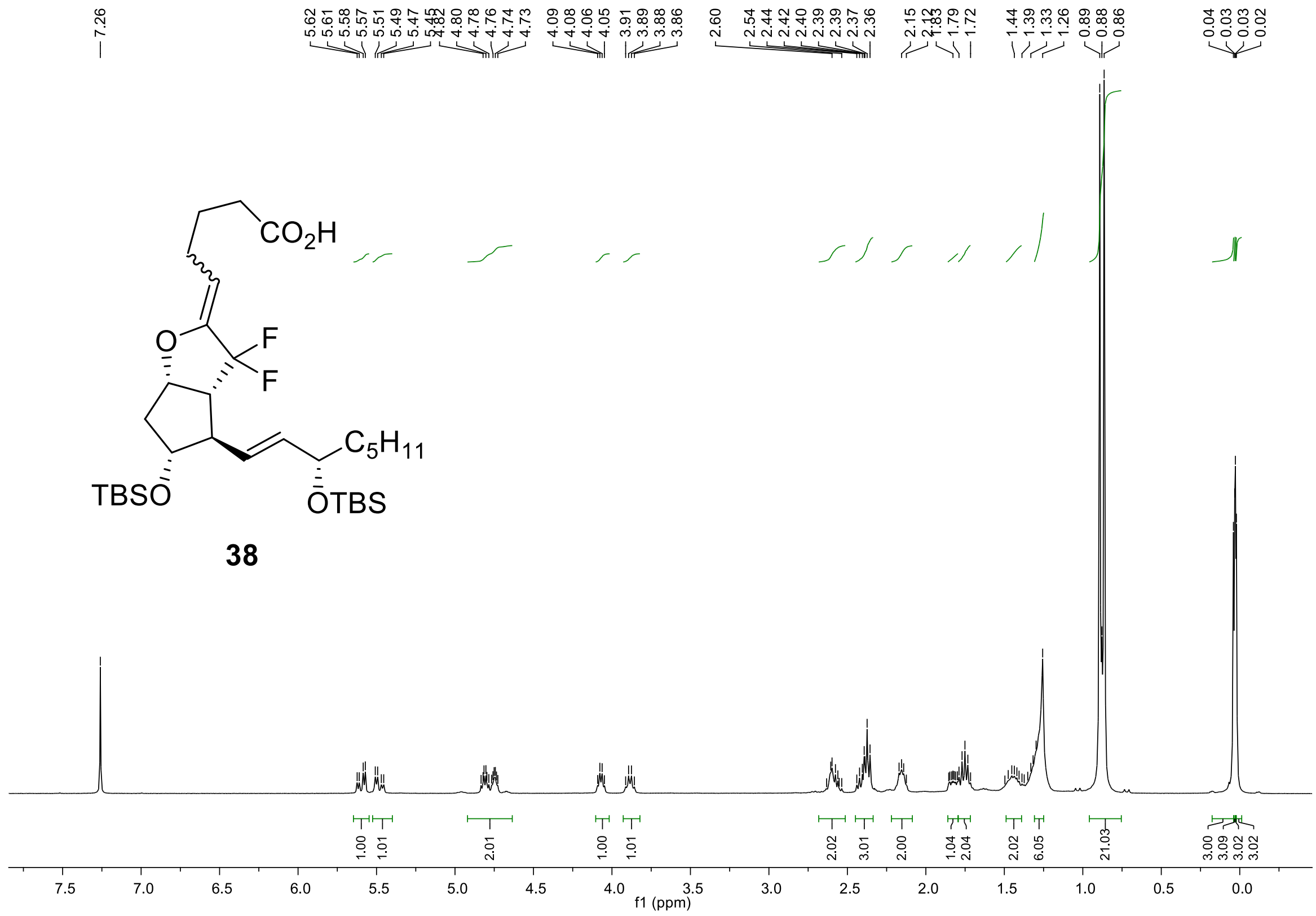

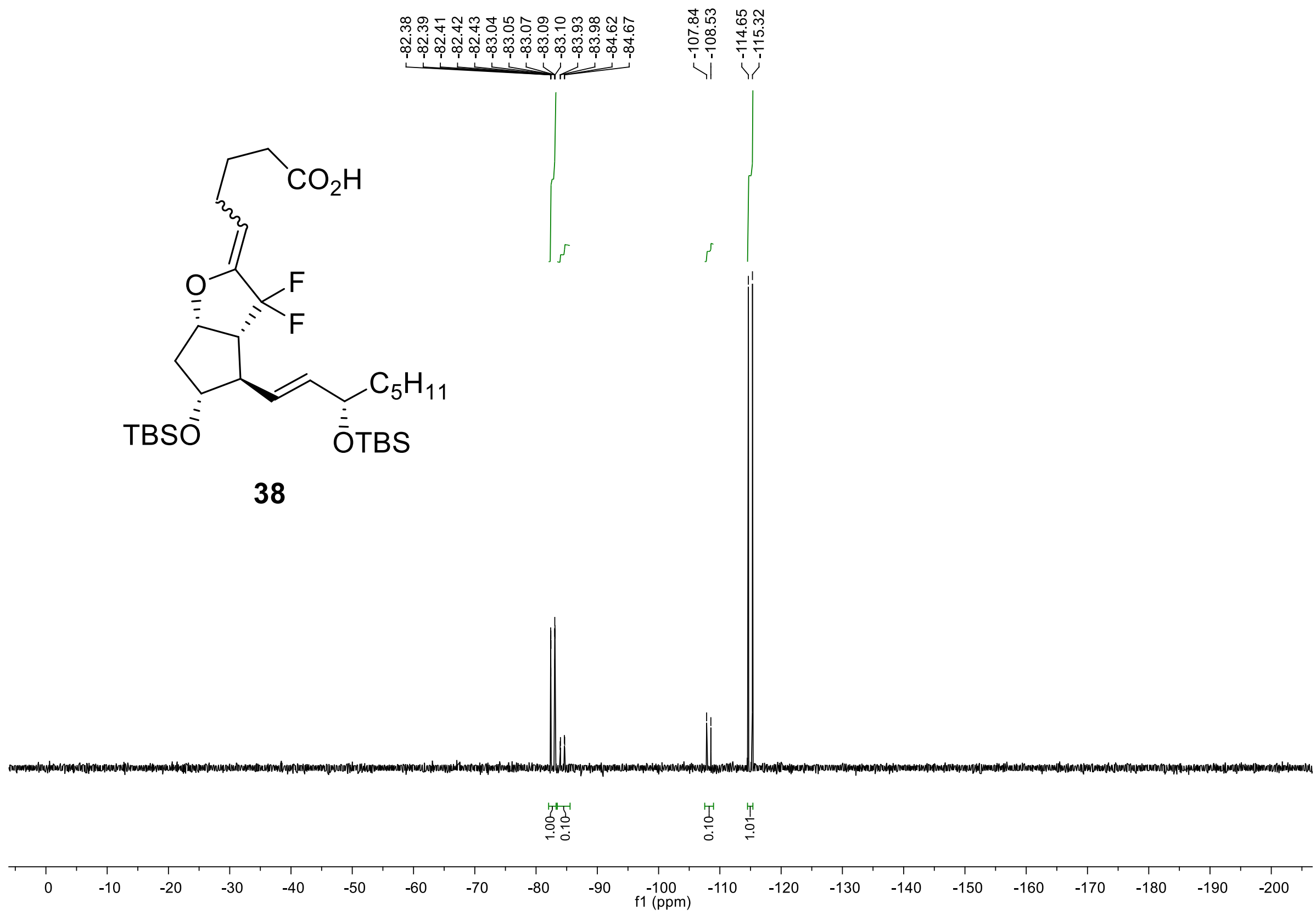

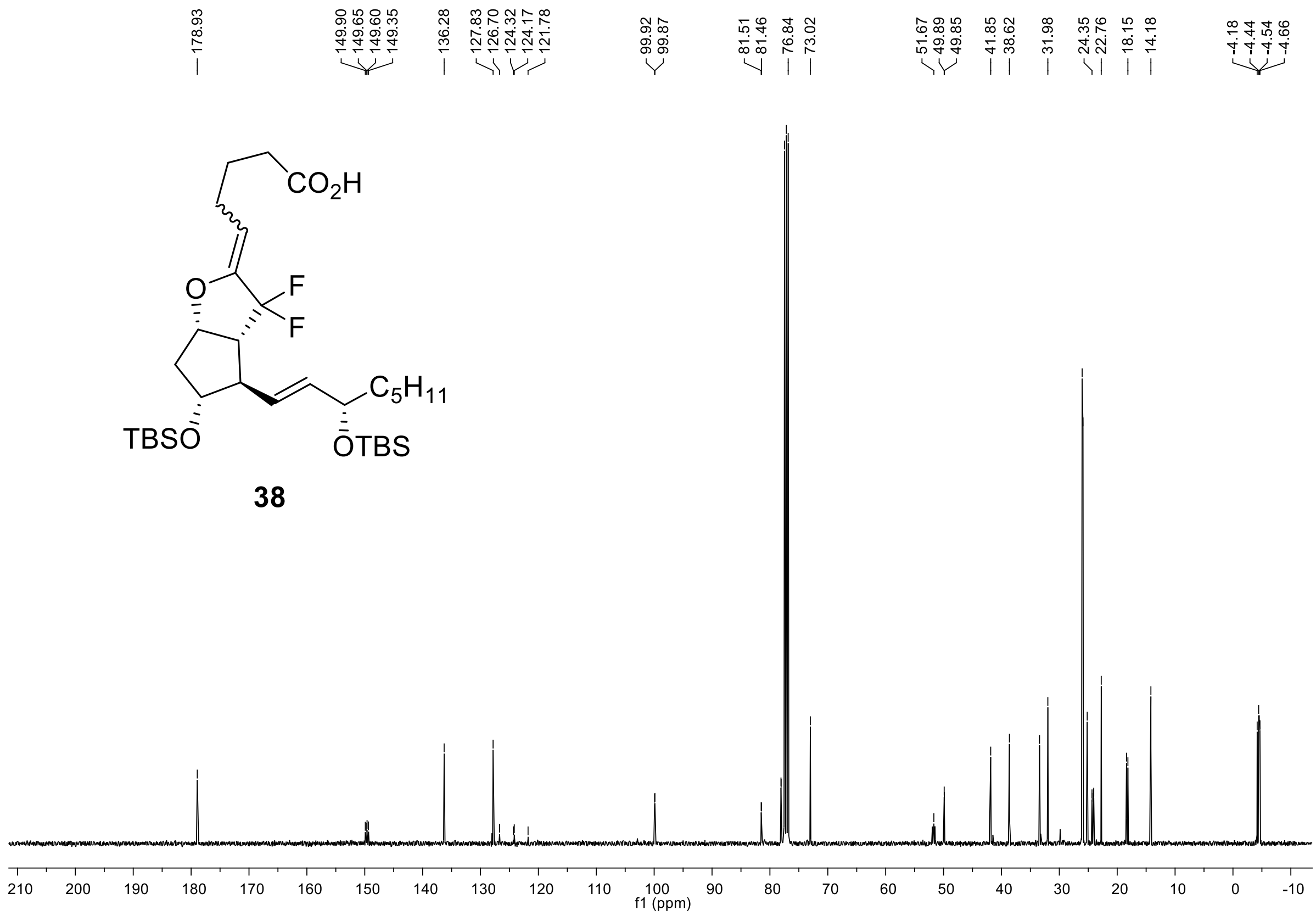

178.93
149.90
149.65
149.60
149.35
136.28
127.83
126.70
124.32
124.17
121.78
99.92
99.87
81.51
81.46
76.84
73.02
51.67
49.89
49.85
41.85
38.62
31.98
24.35
22.76
18.15
14.18
-4.18
-4.44
-4.54
-4.66
CO2H
O
F
F
C5H11
TBSO
OTBS
38
f1 (ppm)

5-((3a*R*,4*R*,5*R*,6a*S*)-3,3-Difluoro-5-hydroxy-4-((*S*,*E*)-3-hydroxyoct-1-enyl)hexahydro-2*H*-cyclopenta[*b*]furan-2-ylidene)pentanoic acid (6)

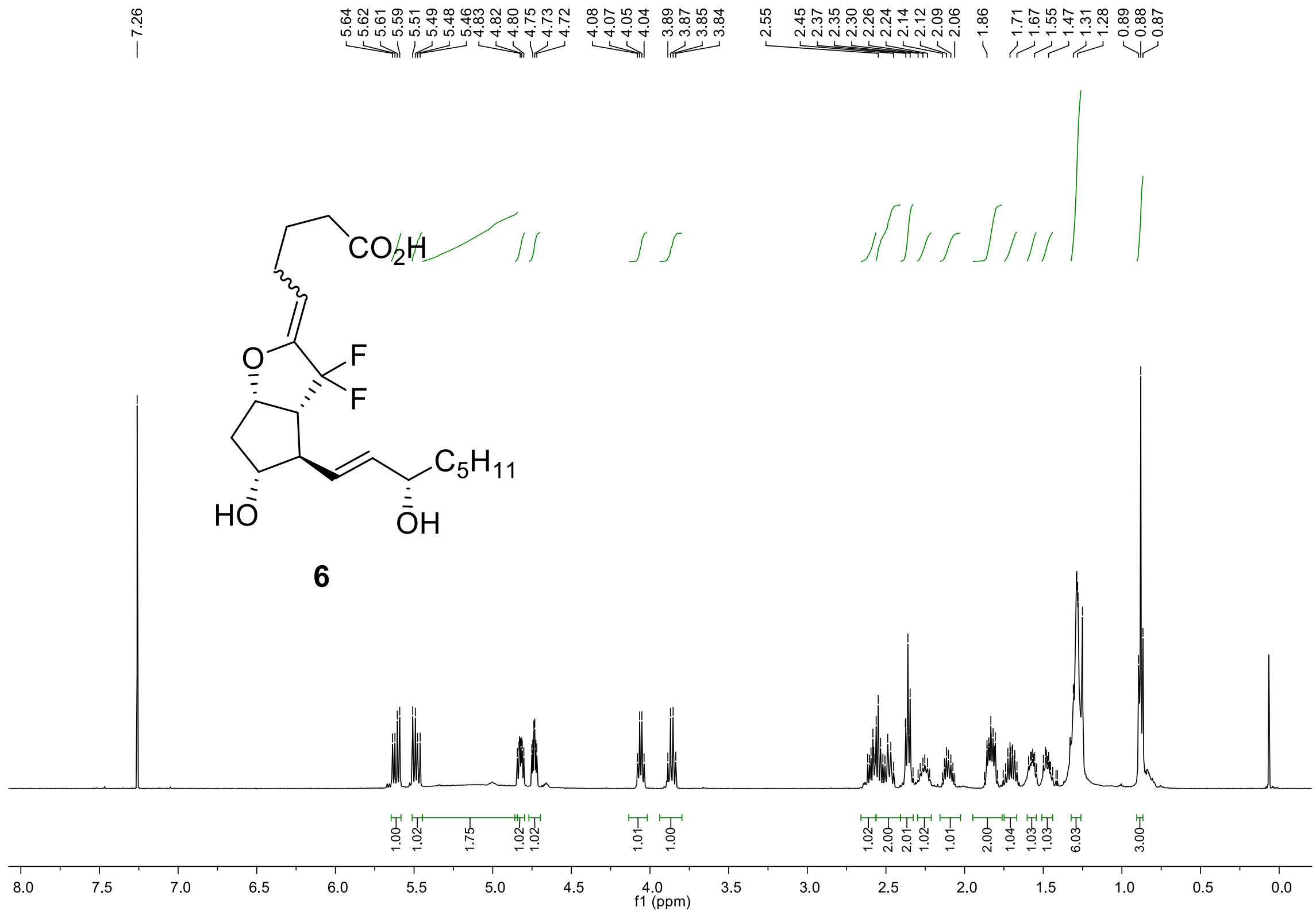

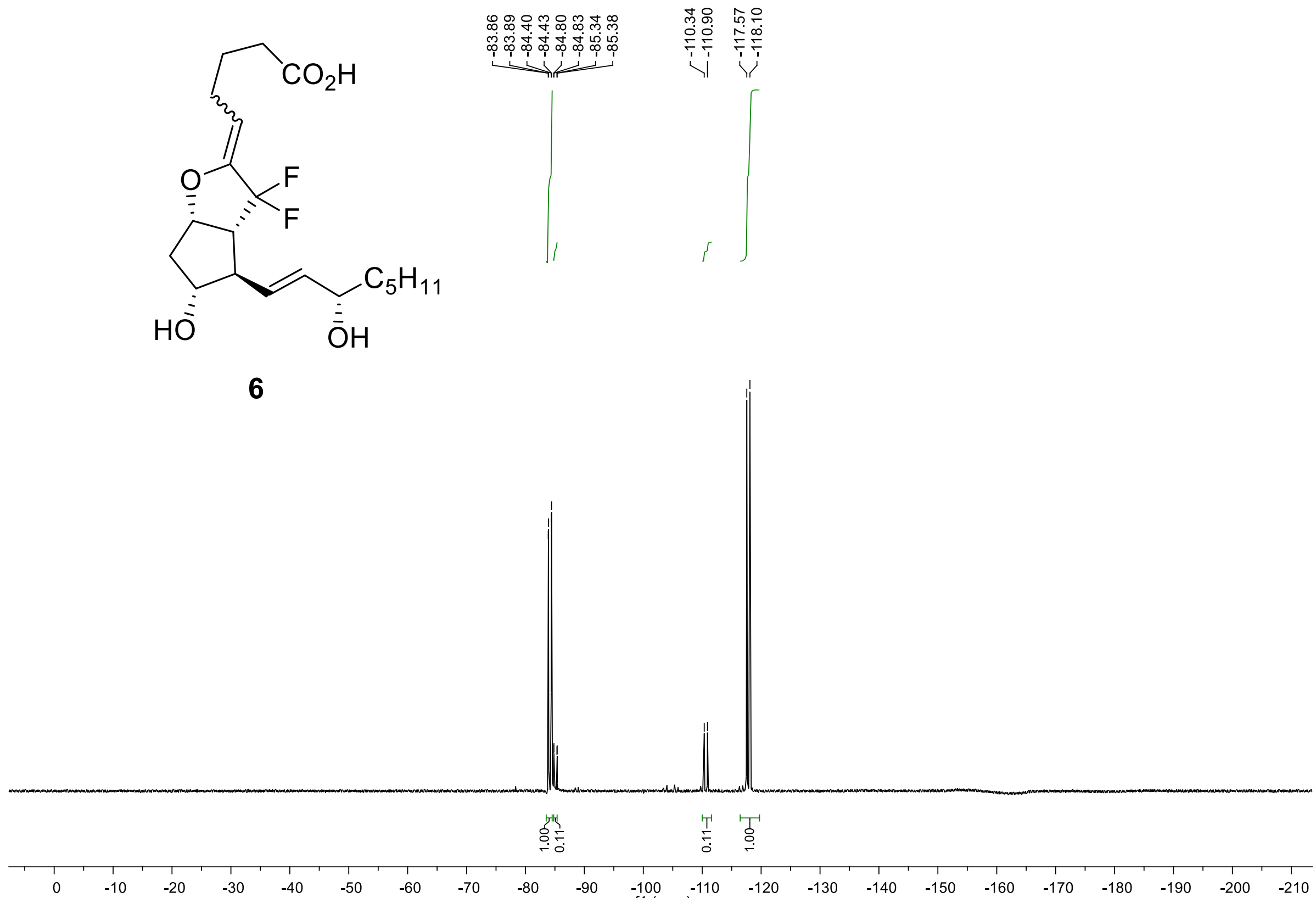

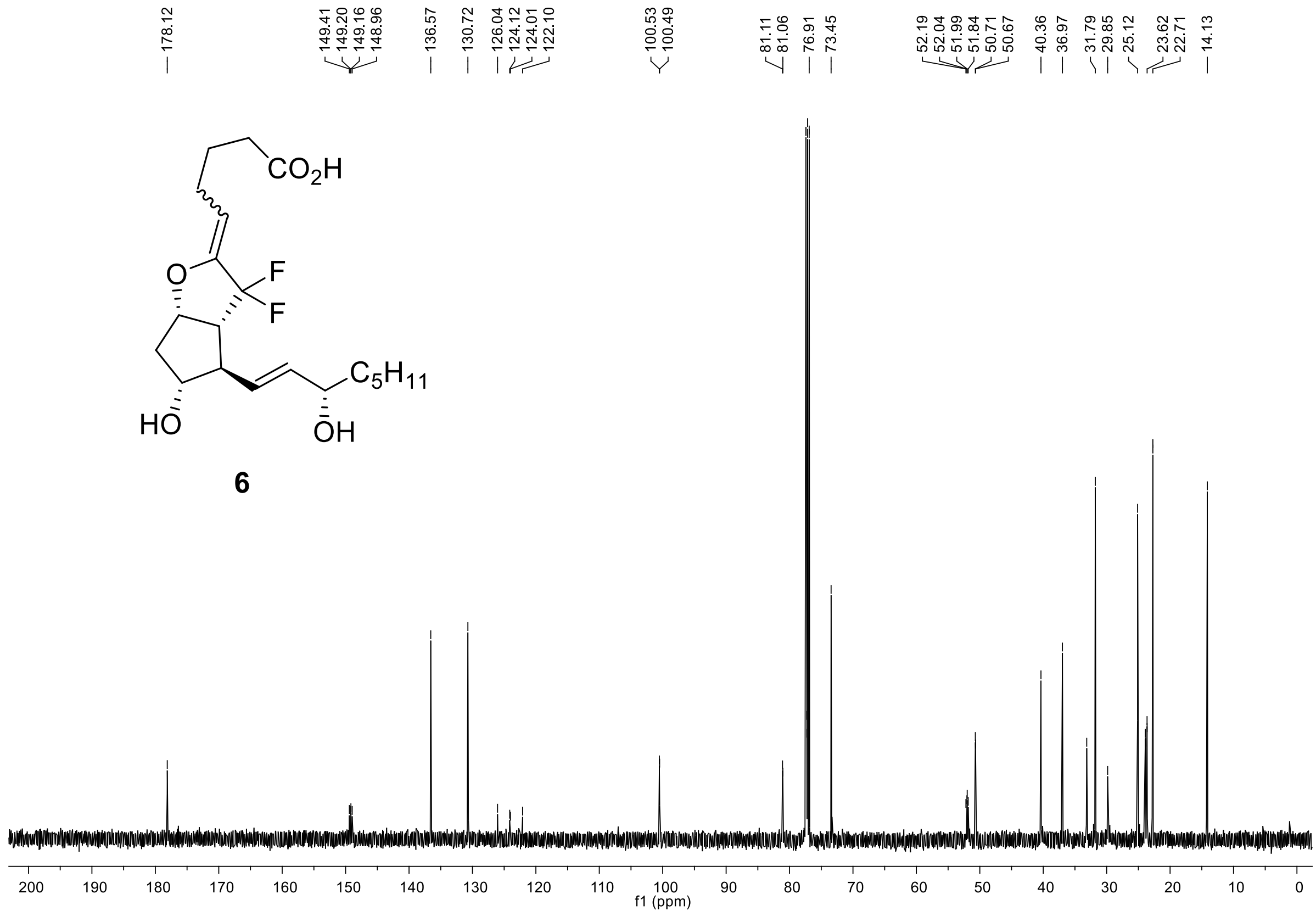
178.12
149.41
149.20
149.16
148.96
136.57
130.72
126.04
124.12
124.01
122.10
100.53
100.49
81.11
81.06
76.91
73.45
52.19
52.04
51.99
51.84
50.71
50.67
40.36
36.97
31.79
29.85
25.12
23.62
22.71
14.13
CO2H
O
F
F
C5H11
HO
OH
6
f1 (ppm)

(3a*R*,4*R*,5*R*,6a*S*)-5-(*tert*-Butyldimethylsilyloxy)-4-((*3*S,4*S*,*E*)-3-(tert-butyldimethylsilyloxy)-4-methyloct-1-en-6-ynyl)-3,3-difluorohexahydro-2*H*-cyclopenta[*b*]furan-2-one (39)

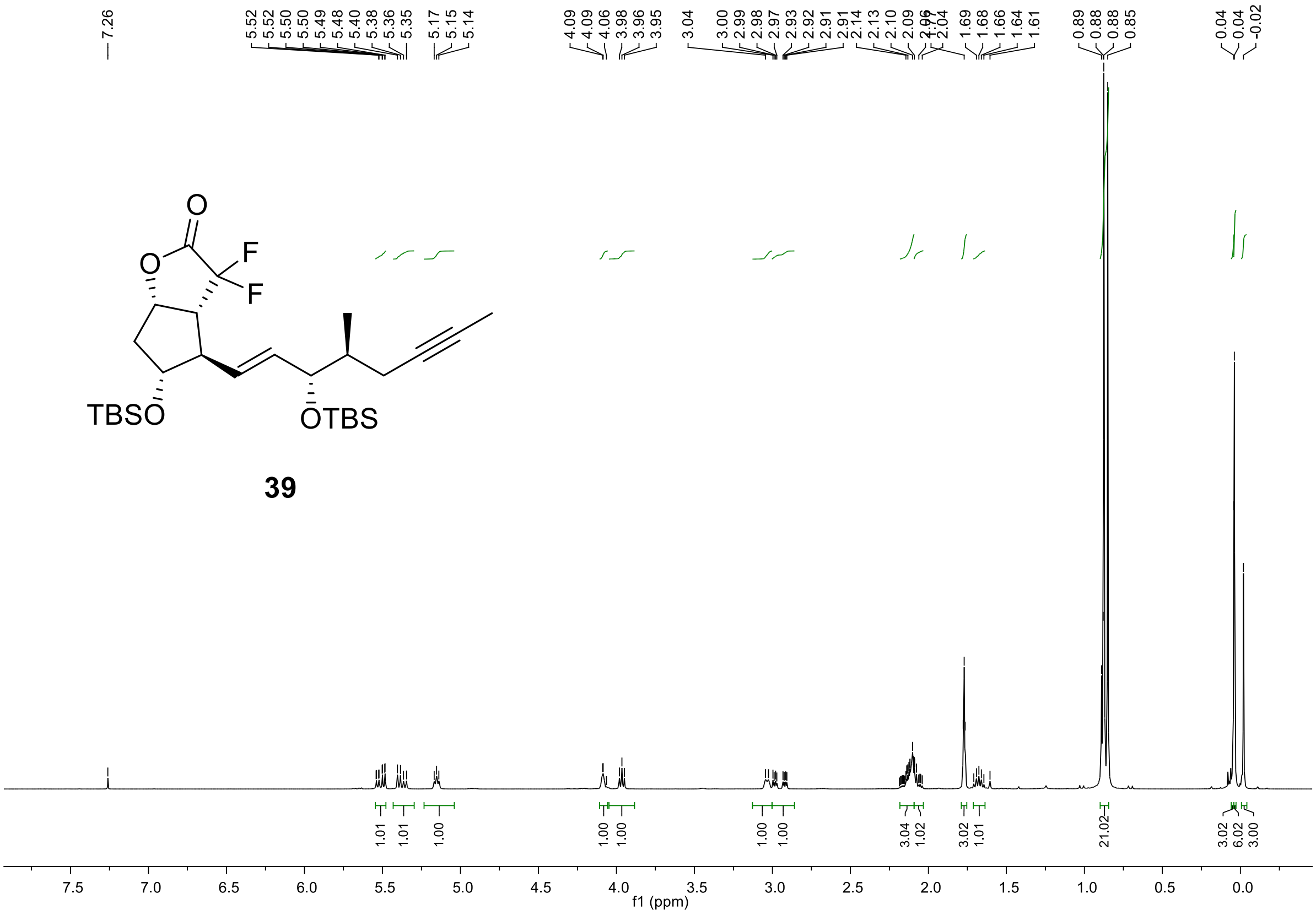

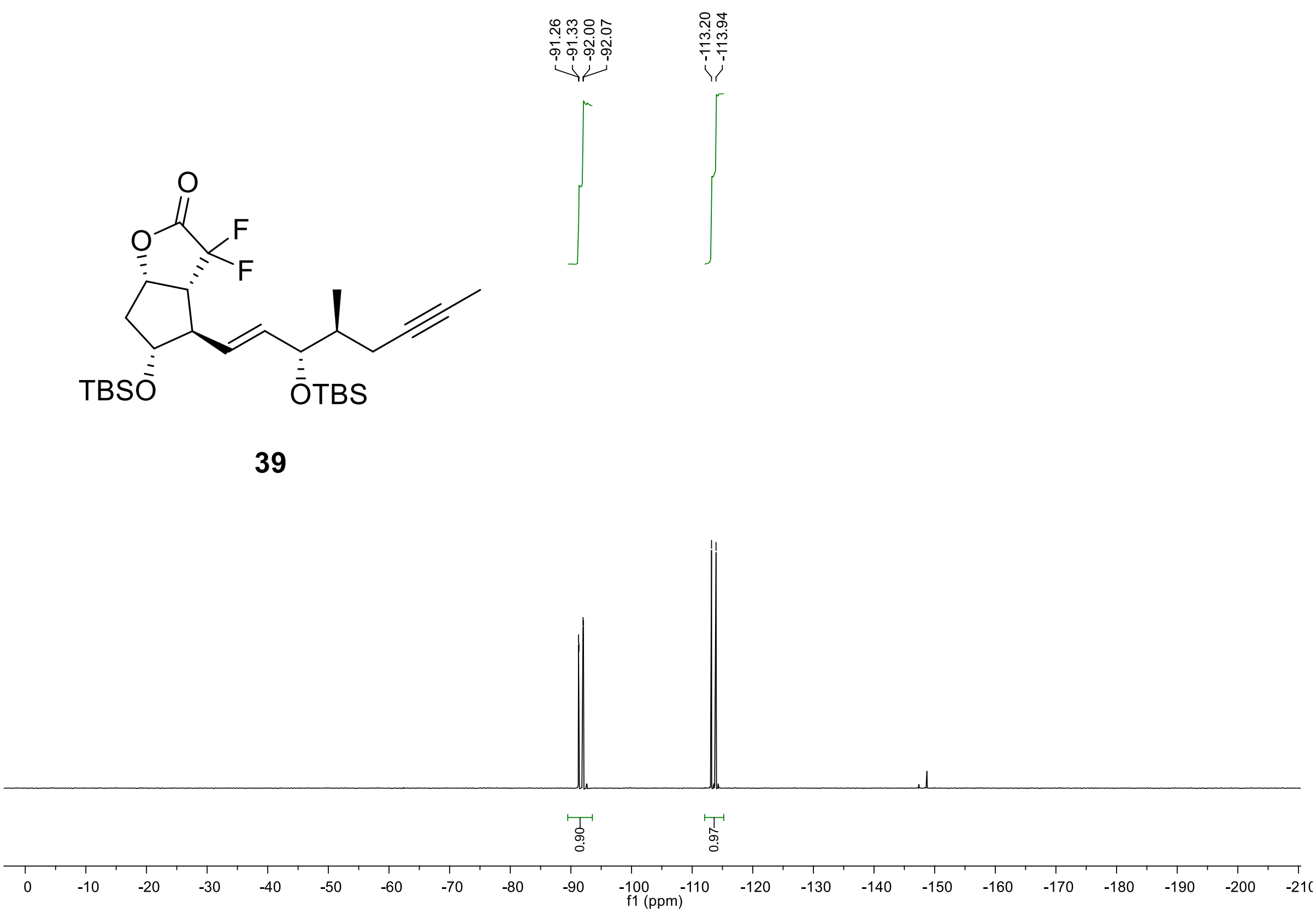

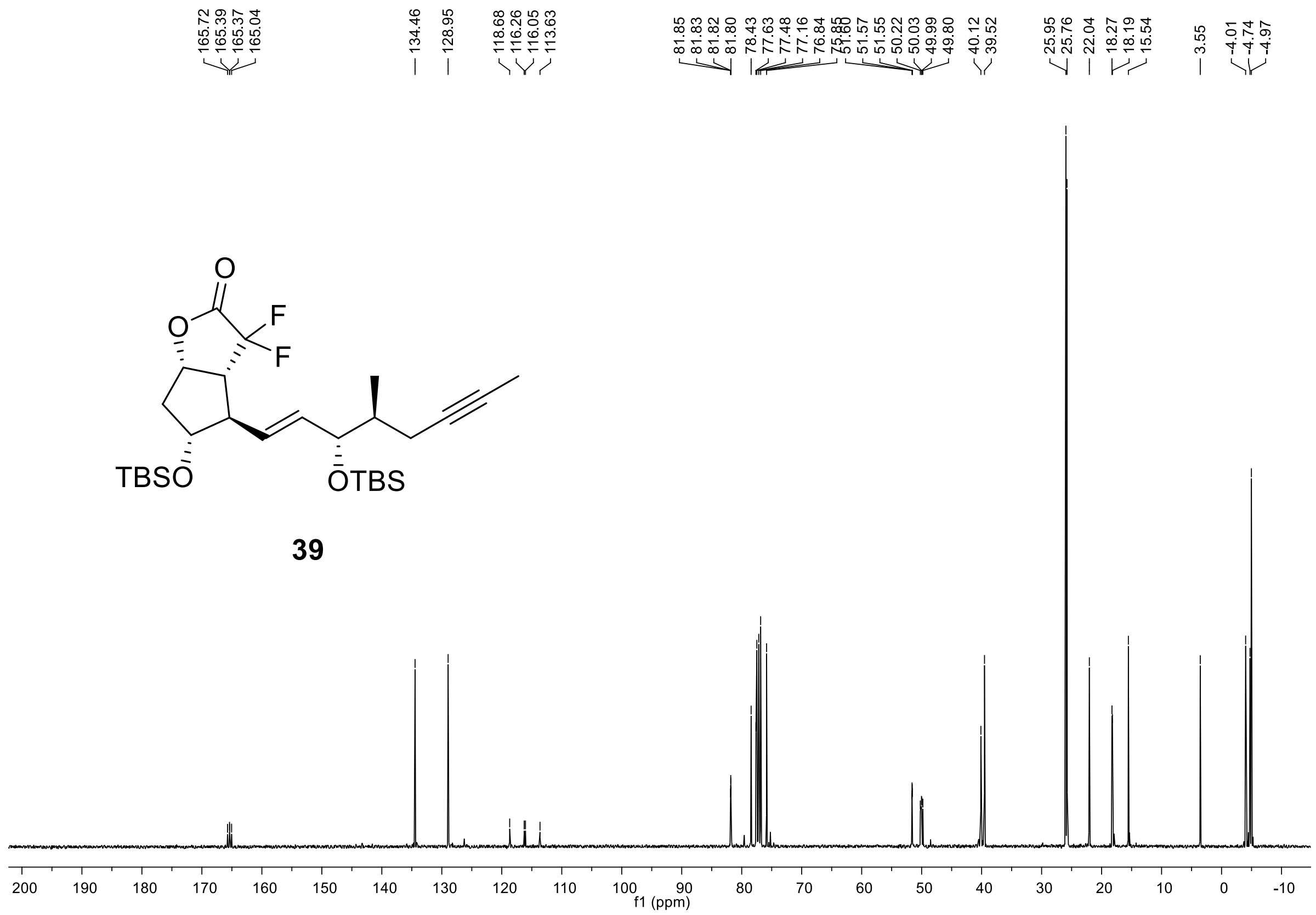
165.72
165.39
165.37
165.04
134.46
128.95
118.68
116.26
116.05
113.63
81.85
81.83
81.82
81.80
78.43
77.63
77.48
77.16
76.84
75.85
51.60
51.57
51.55
50.22
50.03
49.99
49.80
40.12
39.52
25.95
25.76
22.04
18.27
18.19
15.54
3.55
-4.01
-4.74
-4.97
O
O
F
F
TBSO
OTBS
39
200 190 180 170 160 150 140 130 120 110 100 90 80 70 60 50 40 30 20 10 0 -10
f1 (ppm)

5-((3a*R*,4*R*,5*R*,6a*S*)-5-(*tert*-Butyldimethylsilyloxy)-4-((3*S*,4*S*,*E*)-3-(*tert*-butyldimethylsilyloxy)-4-methyloct-1-en-6-ynyl)-3,3-difluorohexahydro-2*H*-cyclopenta[*b*]furan-2-ylidene)pentanoic acid (40)

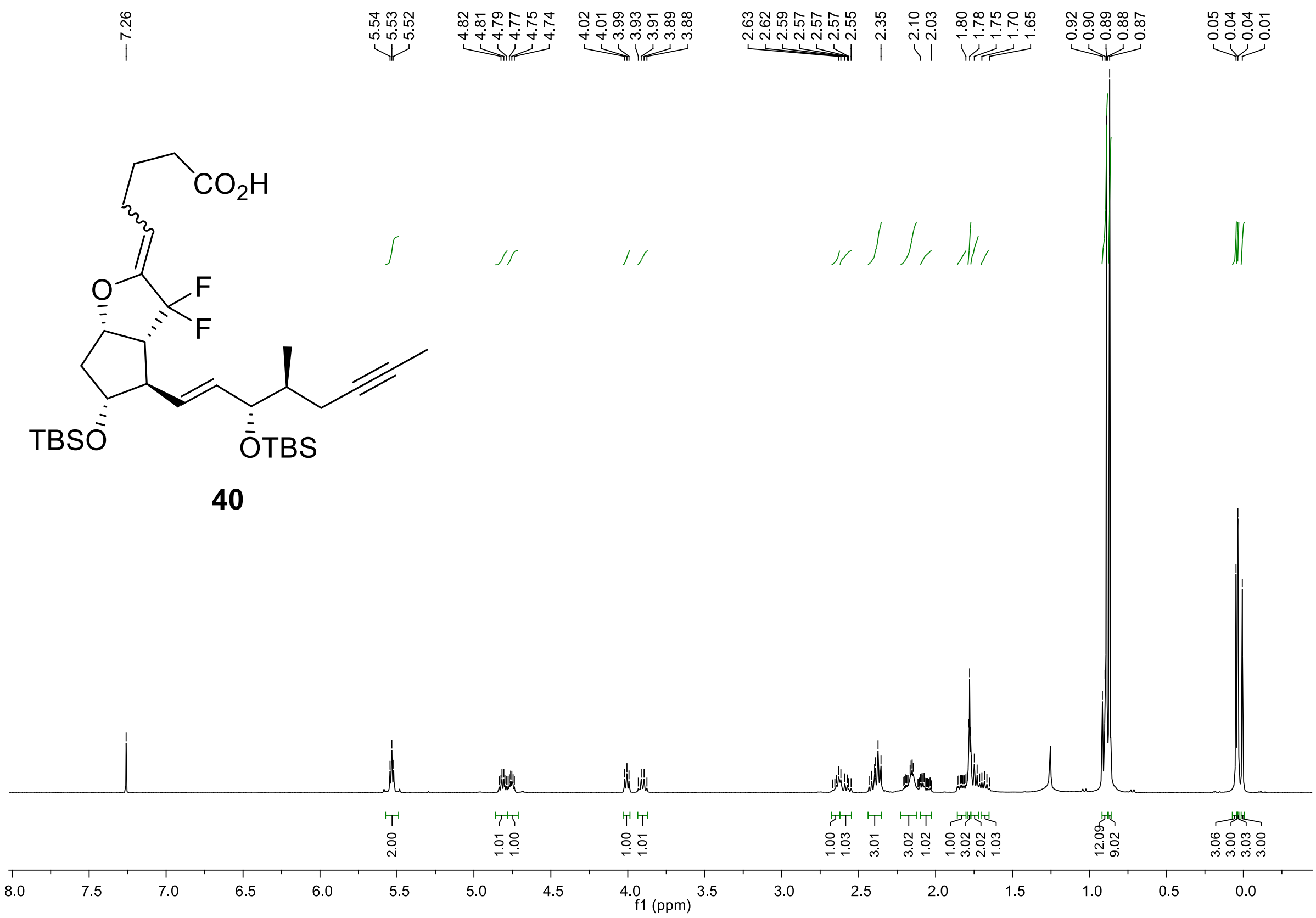

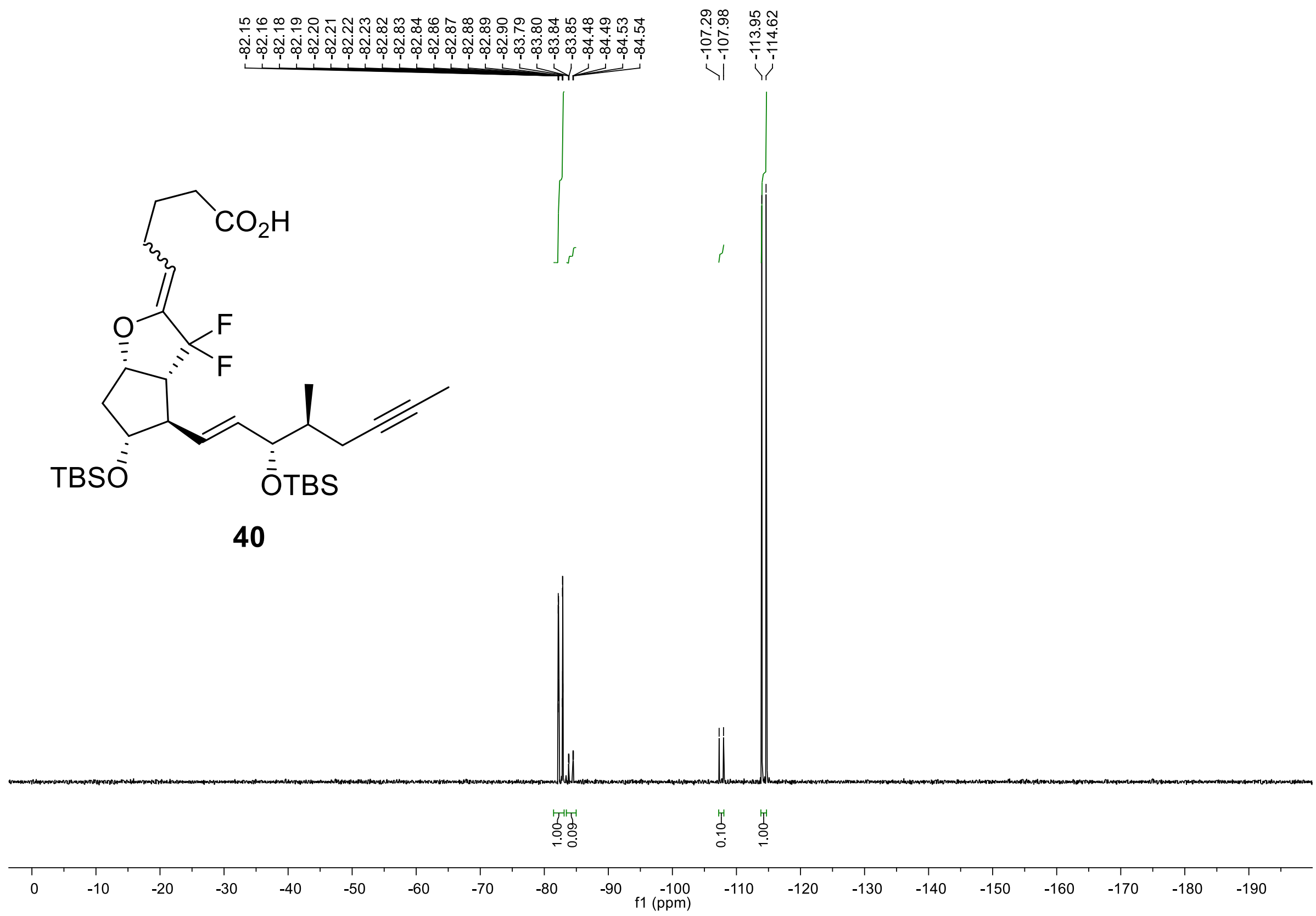

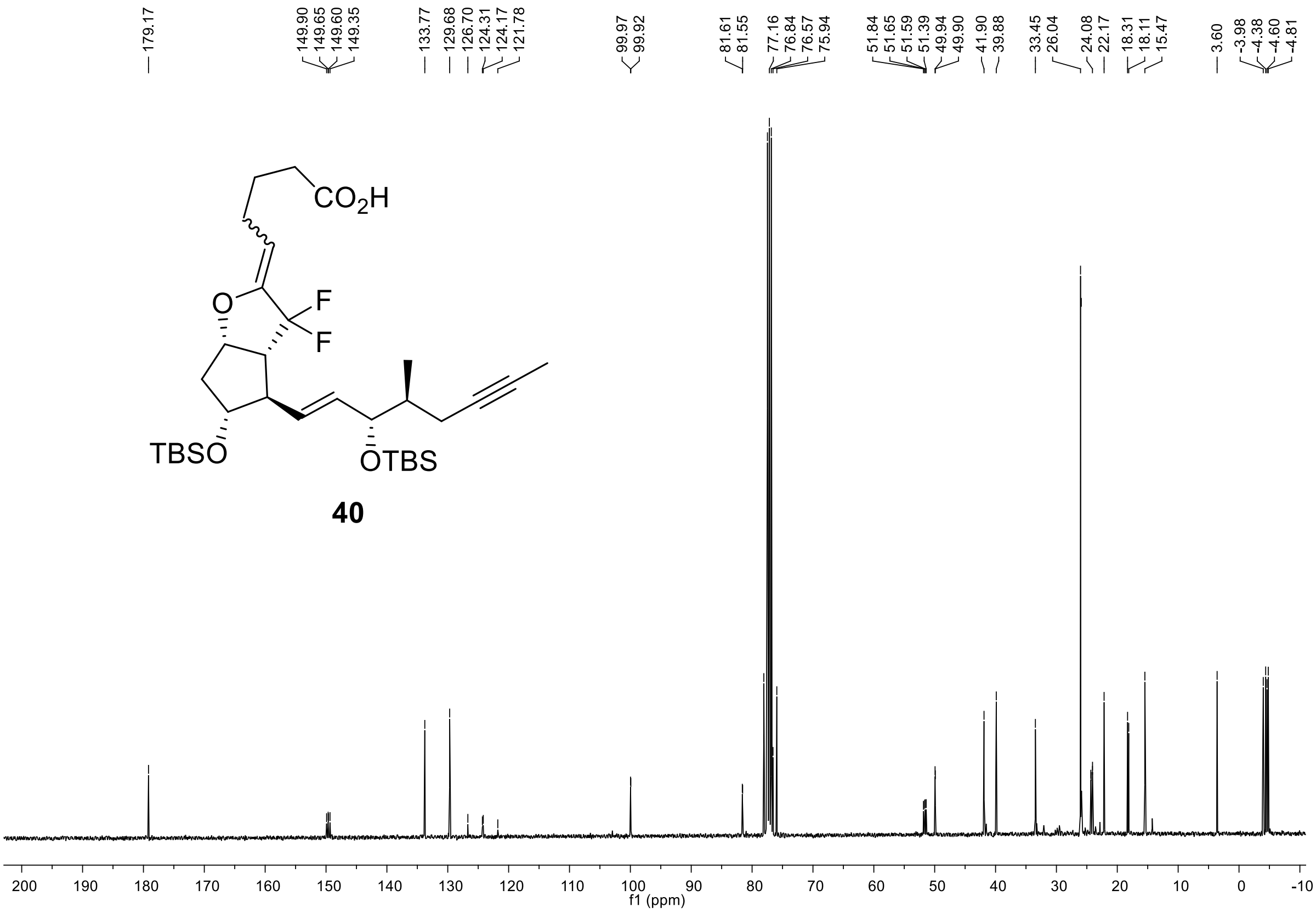
179.17
149.90
149.65
149.60
149.35
133.77
129.68
126.70
124.31
124.17
121.78
99.97
99.92
81.61
81.55
77.16
76.84
76.57
75.94
51.84
51.65
51.59
51.39
49.94
49.90
41.90
39.88
33.45
26.04
24.08
22.17
18.31
18.11
15.47
3.60
-3.98
-4.38
-4.60
-4.81
CO_2H
O
F
F
TBSO
OTBS
40
200
190
180
170
160
150
140
130
120
110
100
90
80
70
60
50
40
30
20
10
0
-10
f1 (ppm)

5-((3a*R*,4*R*,5*R*,6a*S*)-3,3-Difluoro-5-hydroxy-4-((3*S*,4*S*,*E*)-3-hydroxy-4-methyloct-1-en-6-ynyl)hexahydro-2*H*-cyclopenta[*b*]furan-2-ylidene)pentanoic acid (7)

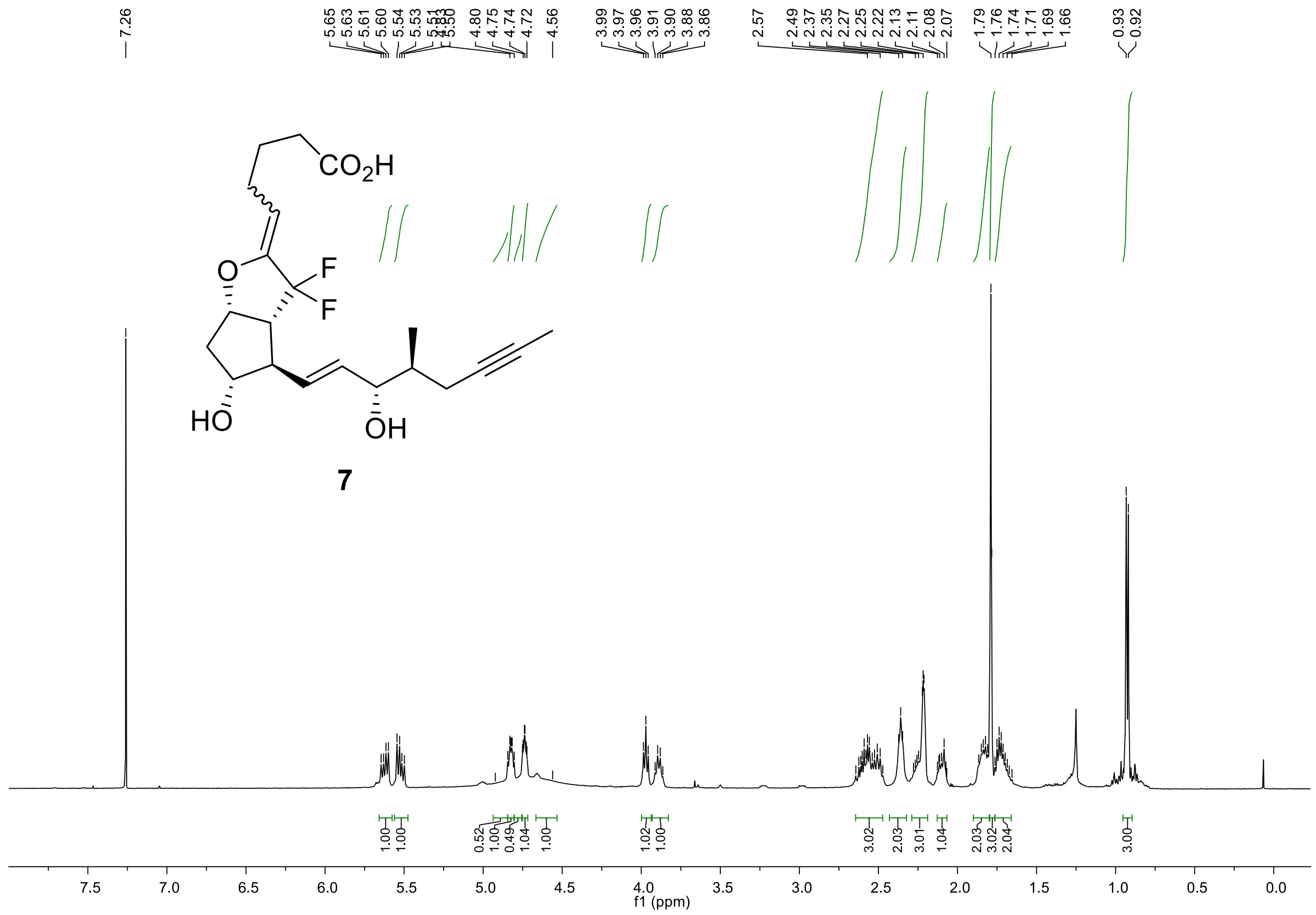

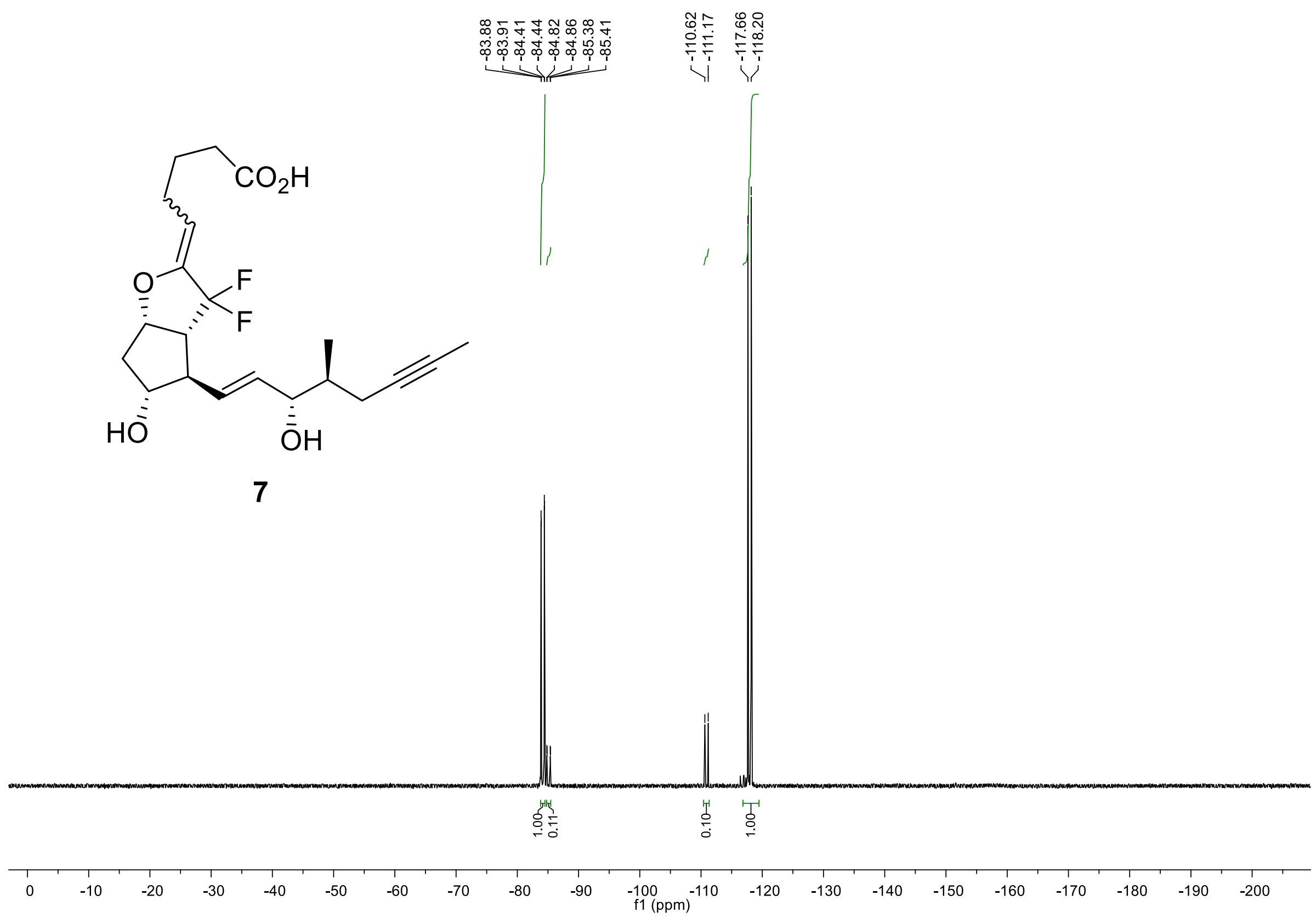

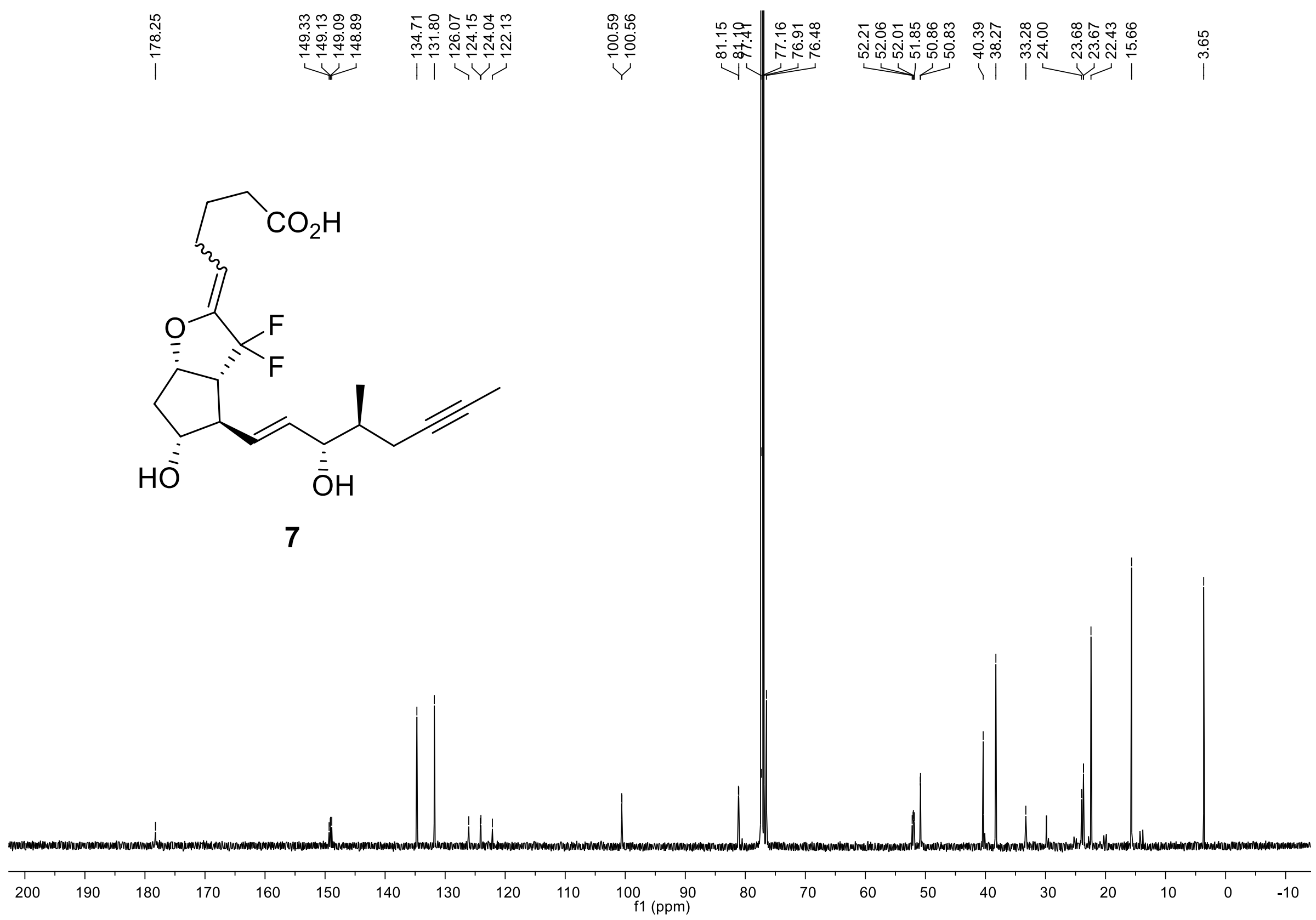

178.25
149.33
149.13
149.09
148.89
134.71
131.80
126.07
124.15
124.04
122.13
100.59
100.56
81.15
81.10
77.41
77.16
76.91
76.48
52.21
52.06
52.01
51.85
50.86
50.83
40.39
38.27
33.28
24.00
23.68
23.67
22.43
15.66
3.65
CO2H
O
F
F
HO
OH
7
f1 (ppm)

(3*S*,3a*R*,4*R*,5*R*,6a*S*)-5-(*tert*-Butyldimethylsilyloxy)-4-((*S*,*E*)-3-(*tert*-butyldimethylsilyloxy)oct-1-enyl)-3-fluorohexahydro-2*H*-cyclopenta[*b*]furan-2-one (48)

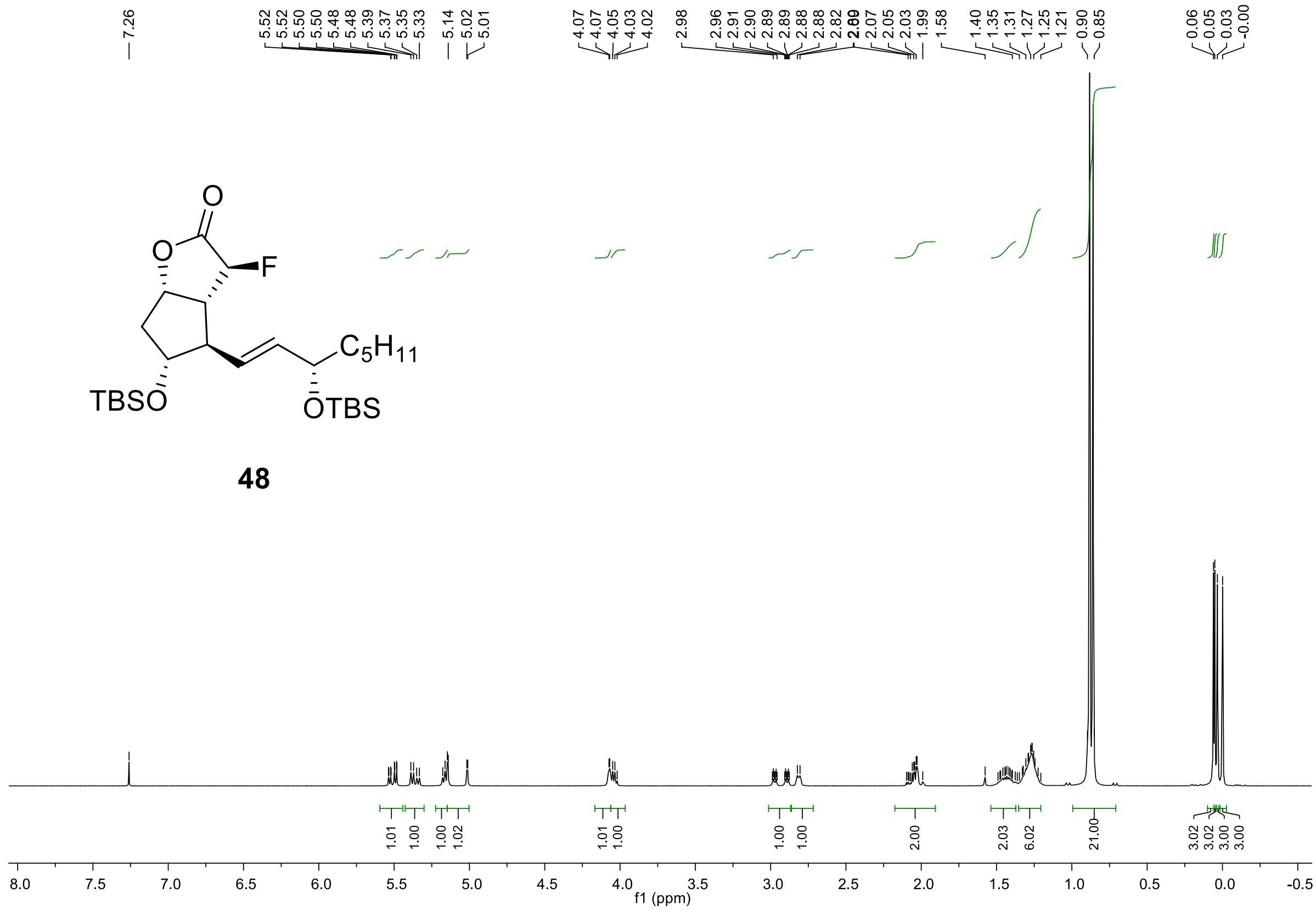

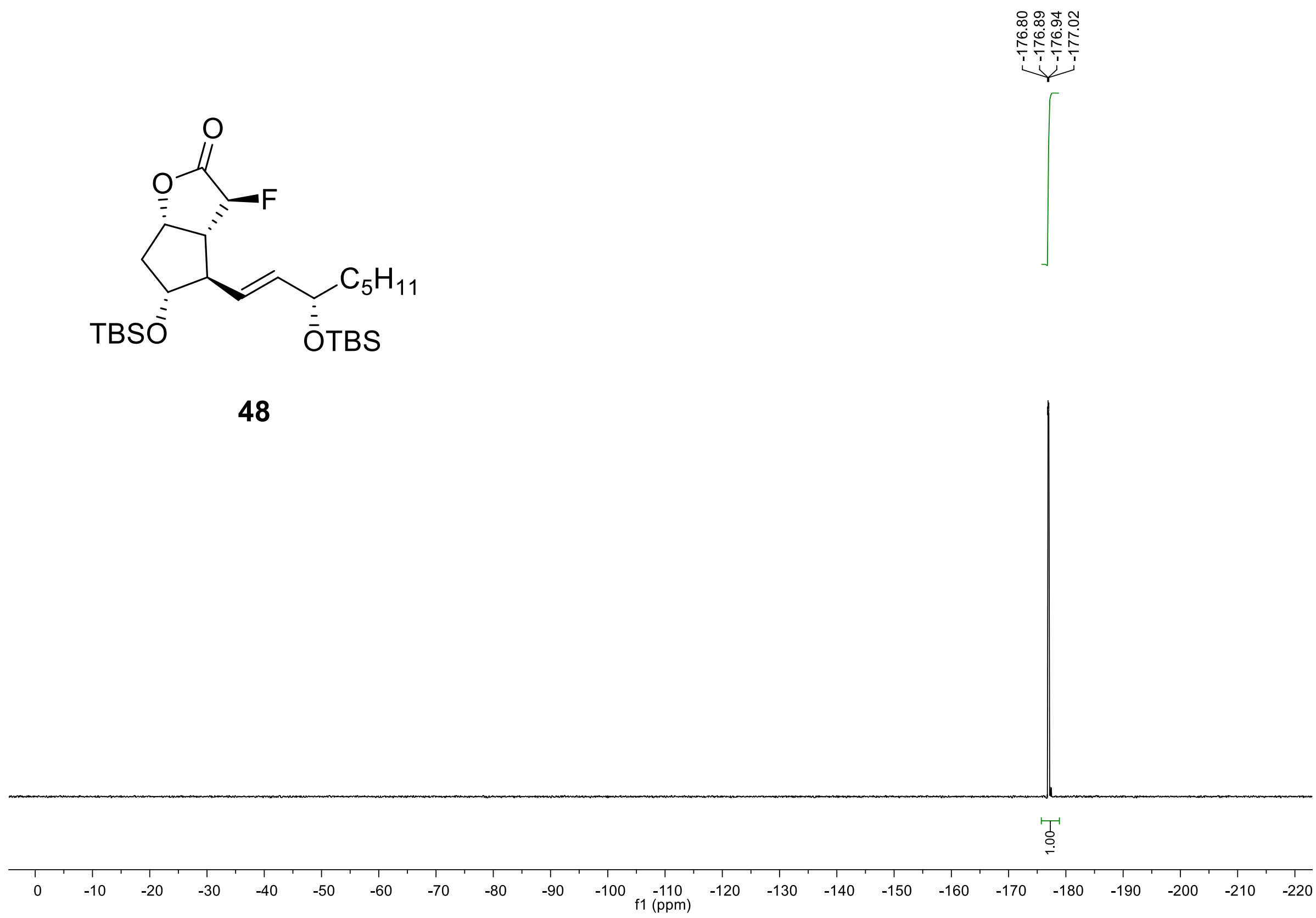

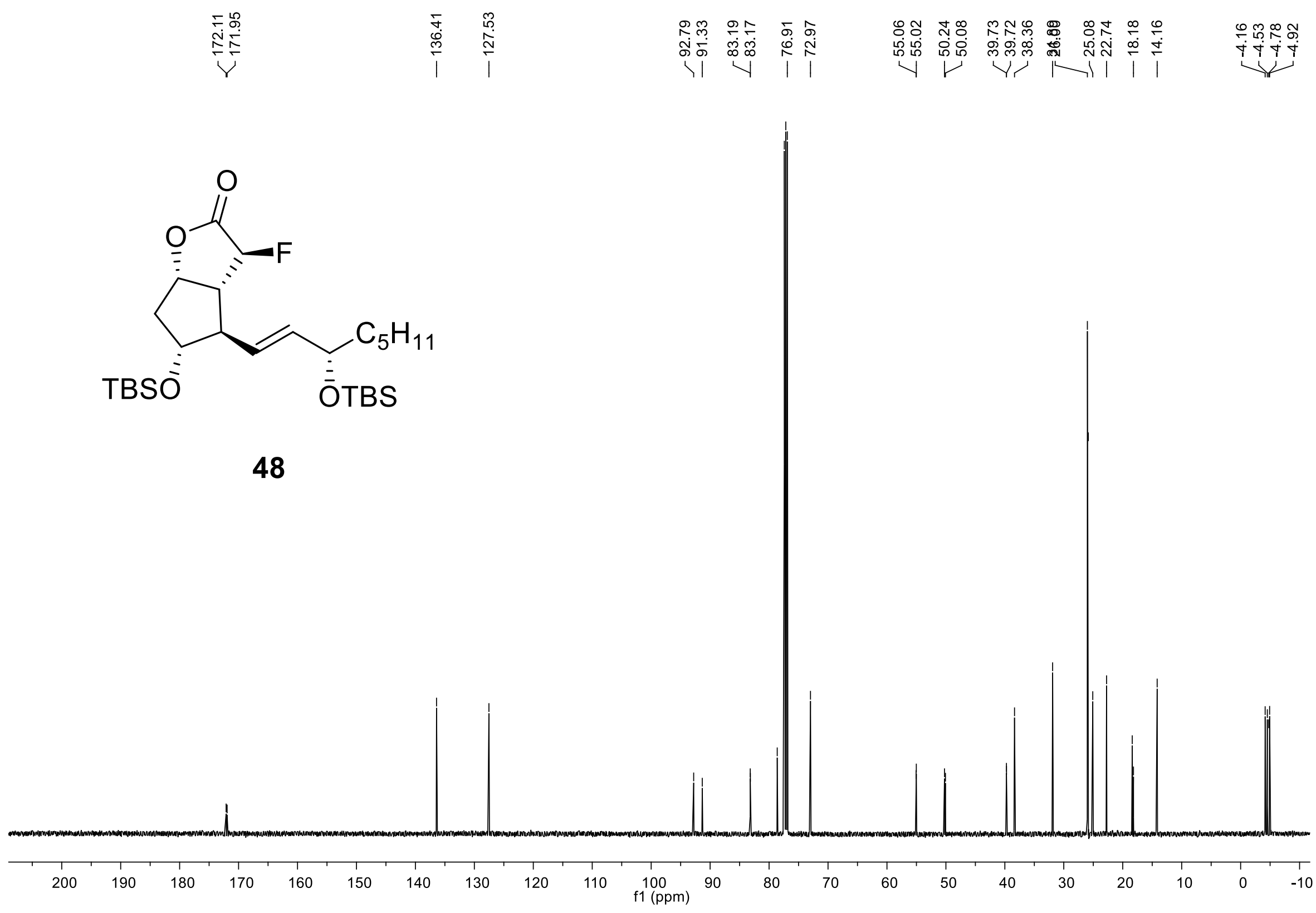

O
O
F
C5H11
TBSO
OTBS
172.11
171.95
136.41
127.53
92.79
91.33
83.19
83.17
76.91
72.97
55.06
55.02
50.24
50.08
39.73
39.72
38.36
25.08
22.74
18.18
14.16
-4.16
-4.53
-4.78
-4.92
200
190
180
170
160
150
140
130
120
110
100
90
80
70
60
50
40
30
20
10
0
-10
f1 (ppm)

48

5-((3*S*,3a*R*,4*R*,5*R*,6a*S*)-5-(*tert*-Butyldimethylsilyloxy)-4-((*S*,*E*)-3-(*tert*-butyldimethylsilyloxy)oct-1-enyl)-3-fluorohexahydro-2*H*-cyclopenta[*b*]furan-2-ylidene)pentanoic acid (49)

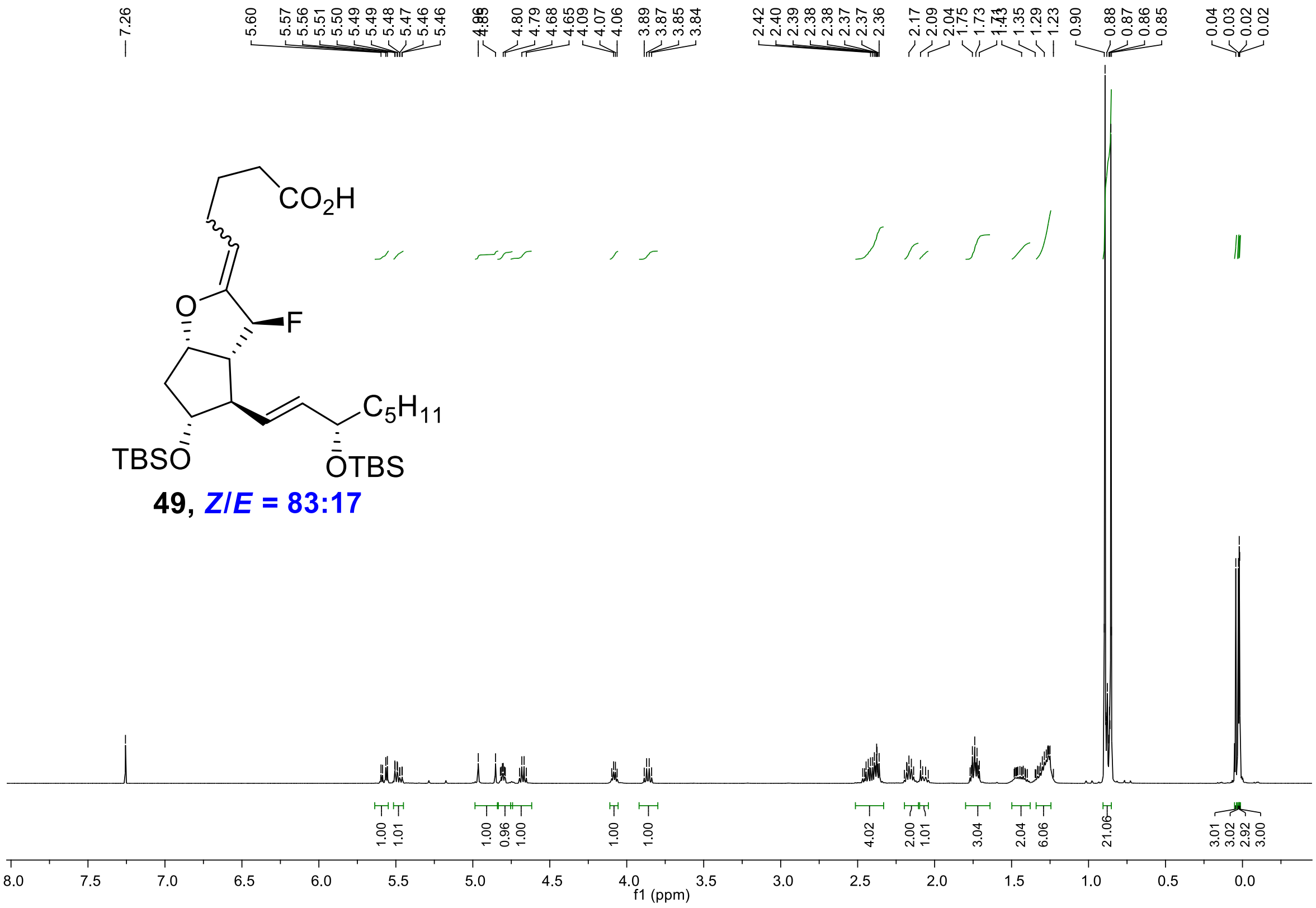

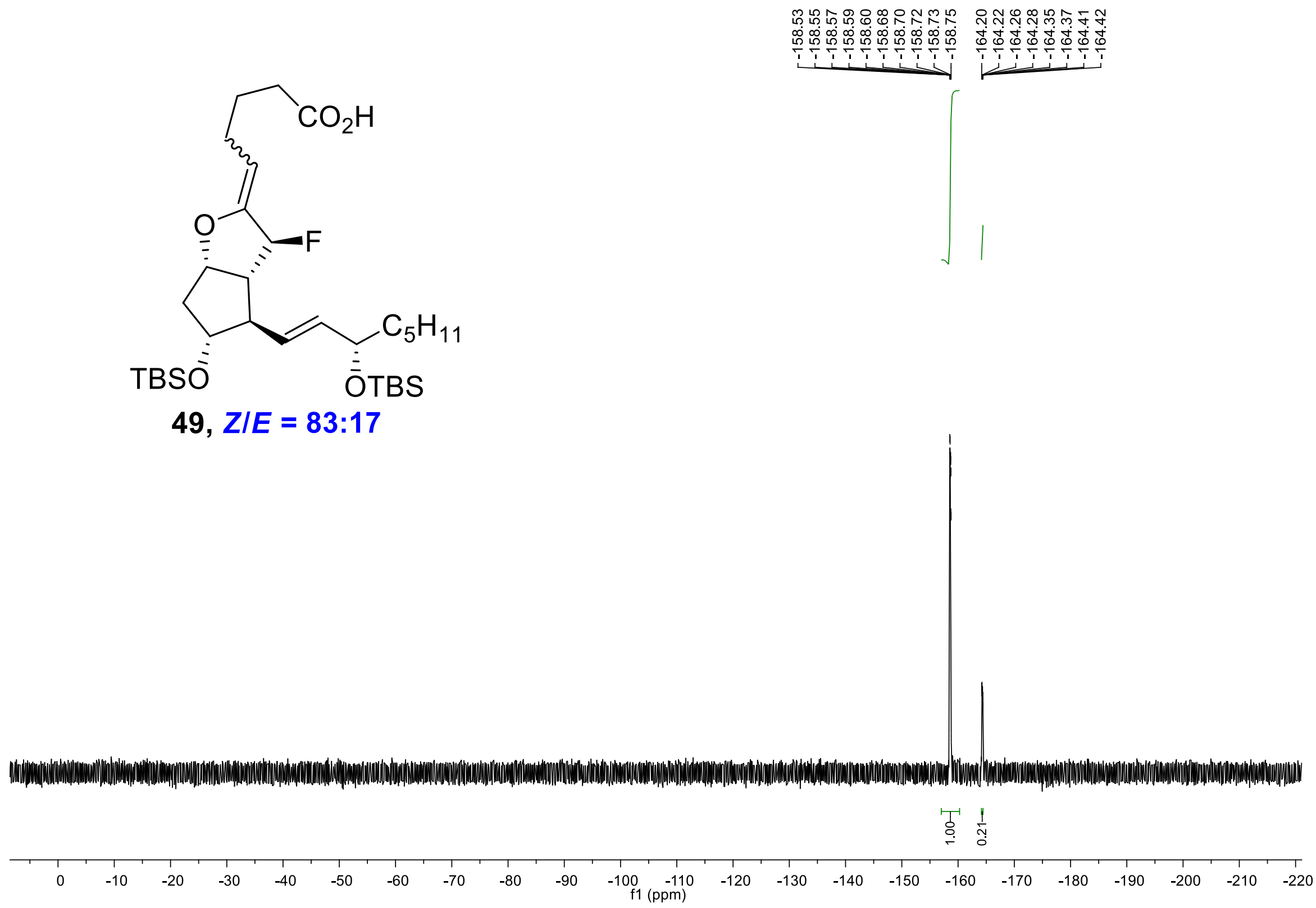

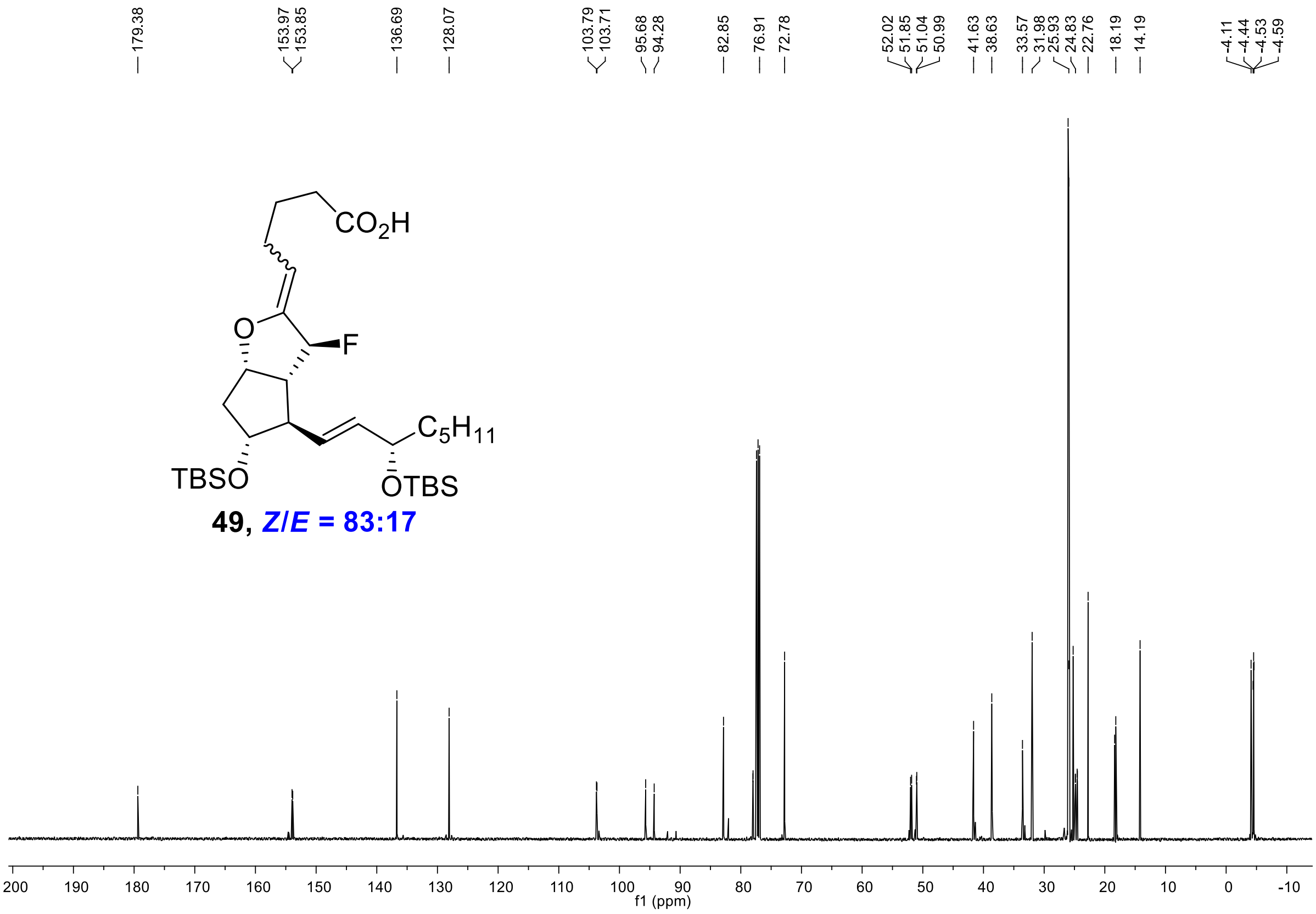

49, ***Z*/*E* = 83:17**

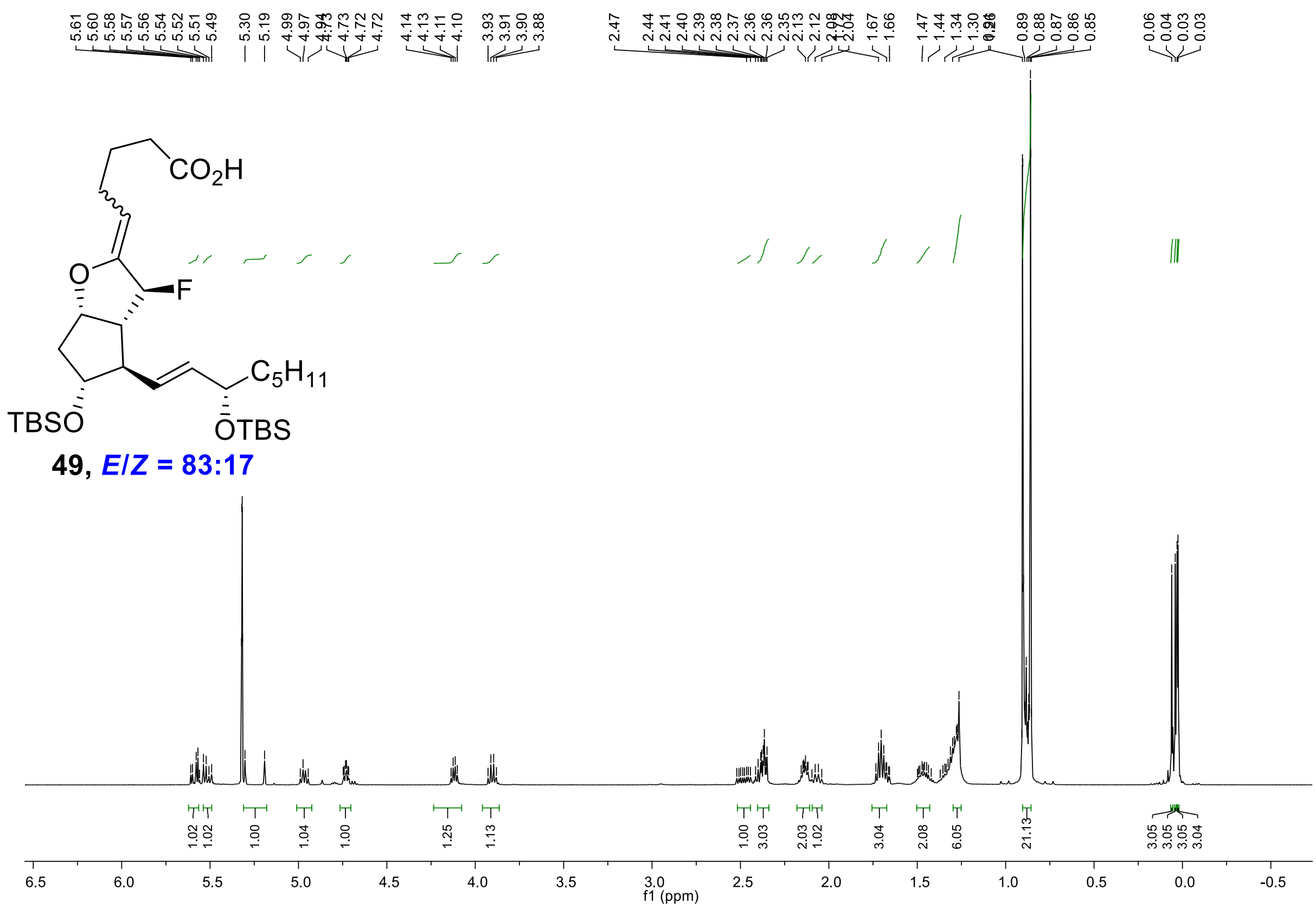

49, ***E*/*Z* = 83:17**

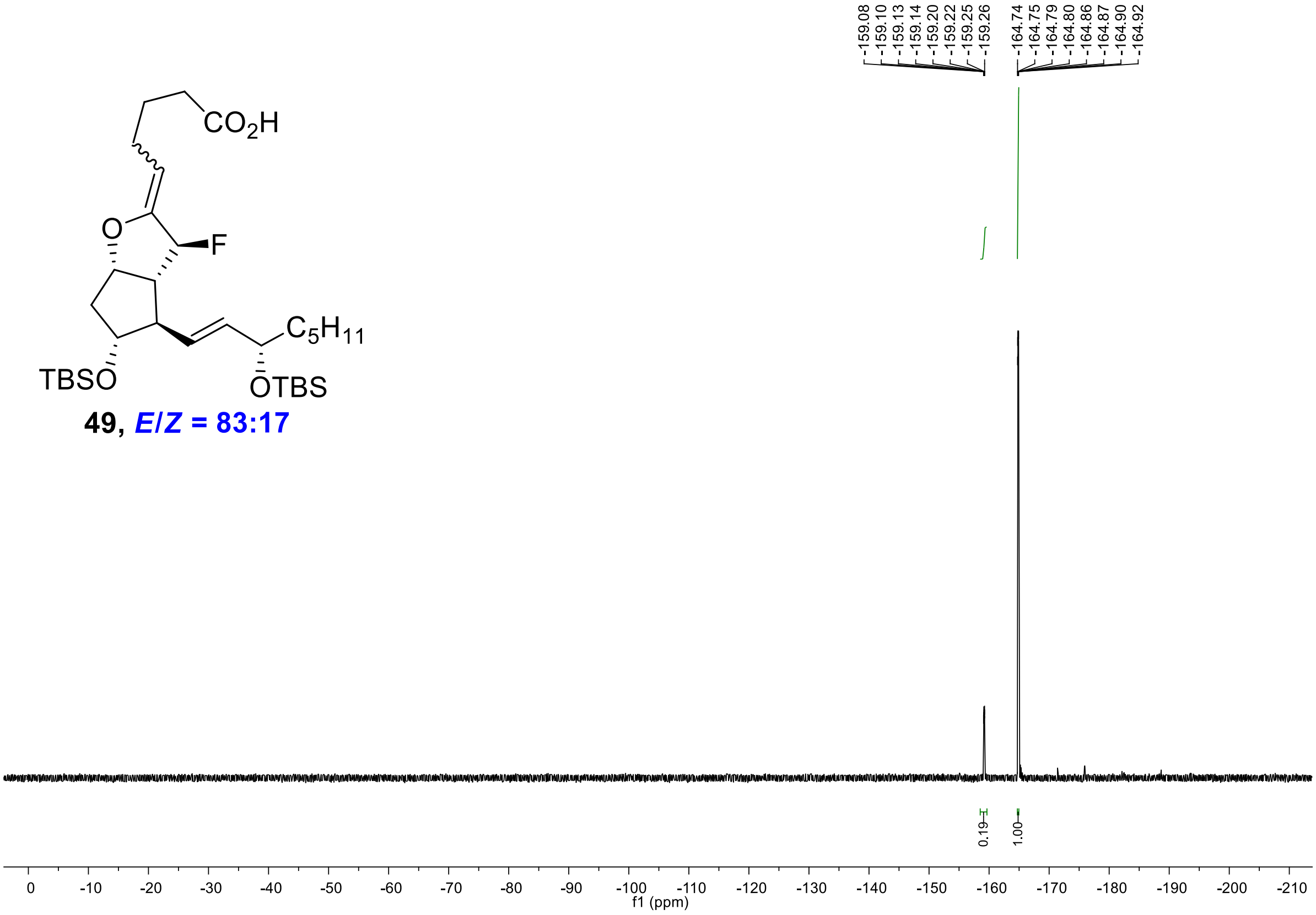
-159.08
-159.10
-159.13
-159.14
-159.20
-159.22
-159.25
-159.26
-164.74
-164.75
-164.79
-164.80
-164.86
-164.87
-164.90
-164.92
CO2H
O
F
C5H11
TBSO
OTBS
49, E/Z = 83:17
0.19
1.00
0 -10 -20 -30 -40 -50 -60 -70 -80 -90 -100 -110 -120 -130 -140 -150 -160 -170 -180 -190 -200 -210
f1 (ppm)

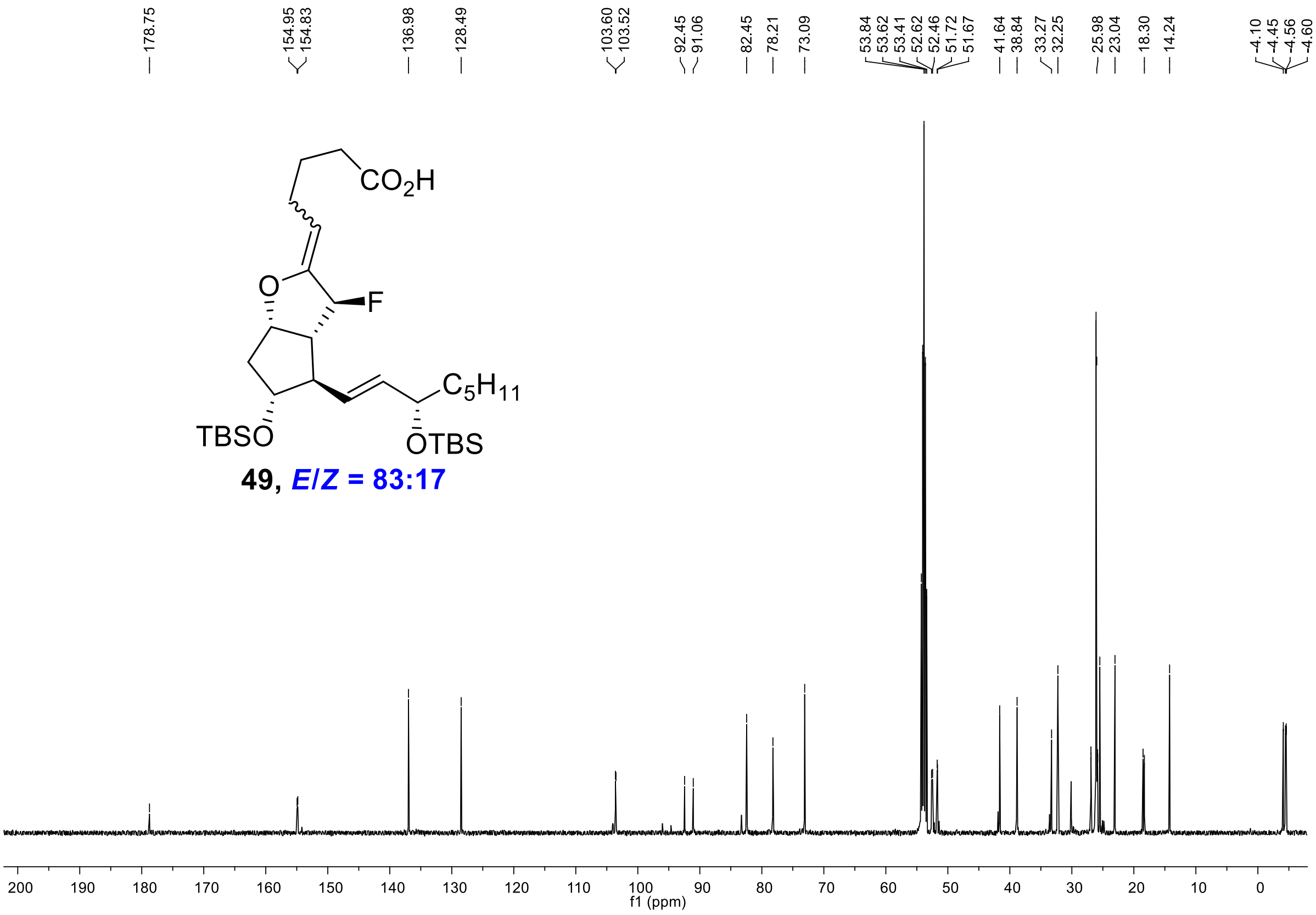
178.75
154.95
154.83
136.98
128.49
103.60
103.52
92.45
91.06
82.45
78.21
73.09
53.84
53.62
53.41
52.62
52.46
51.72
51.67
41.64
38.84
33.27
32.25
25.98
23.04
18.30
14.24
-4.10
-4.45
-4.56
-4.60
CO2H
O
F
C5H11
TBSO
OTBS
49, E/Z = 83:17
200 190 180 170 160 150 140 130 120 110 100 90 80 70 60 50 40 30 20 10 0
f1 (ppm)

5-((3*S*,3a*R*,4*R*,5*R*,6a*S*)-3-Fluoro-5-hydroxy-4-((*S*,*E*)-3-hydroxyoct-1-enyl)hexahydro-2*H*-cyclopenta[*b*]furan-2-ylidene)pentanoic acid (2)

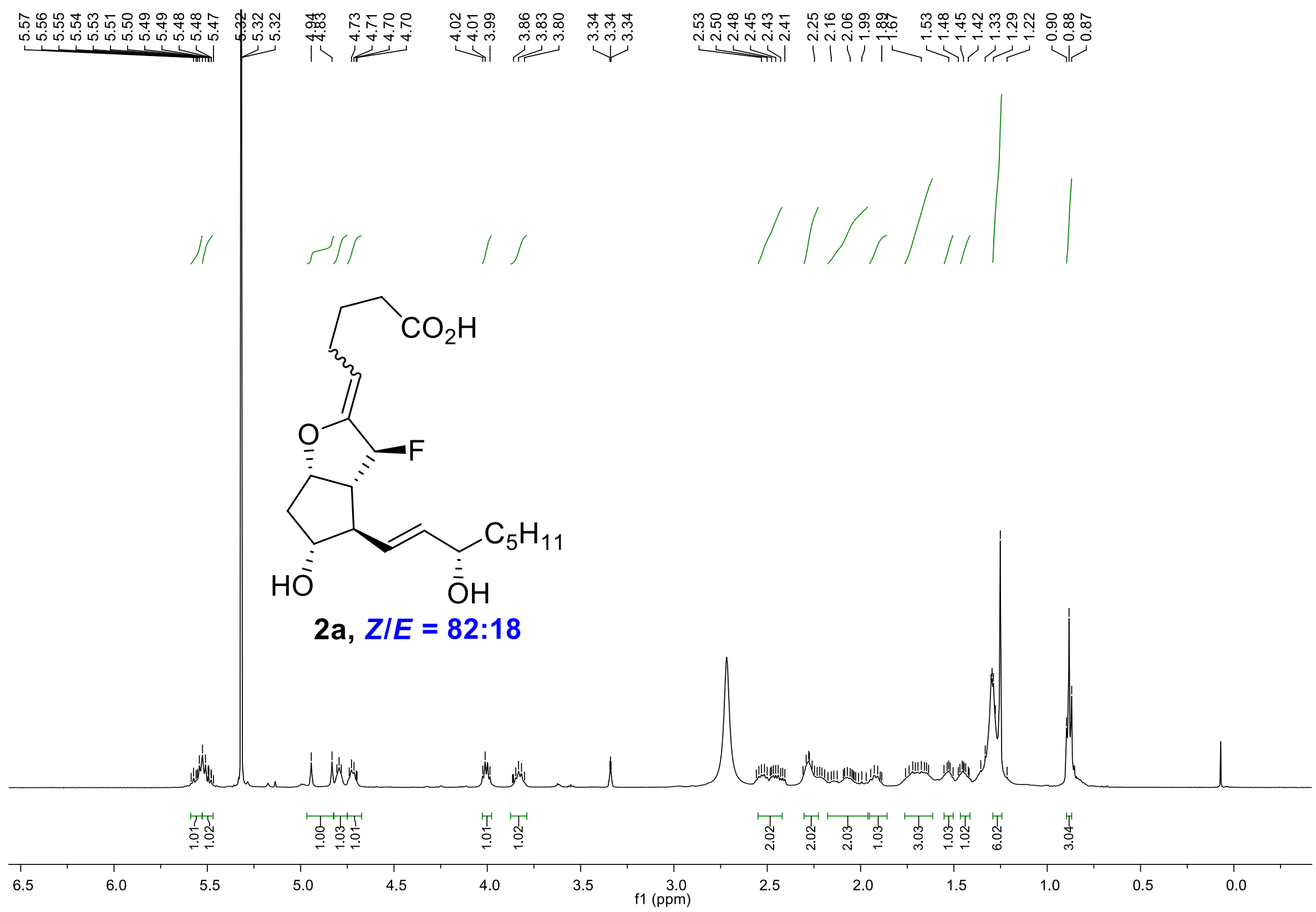

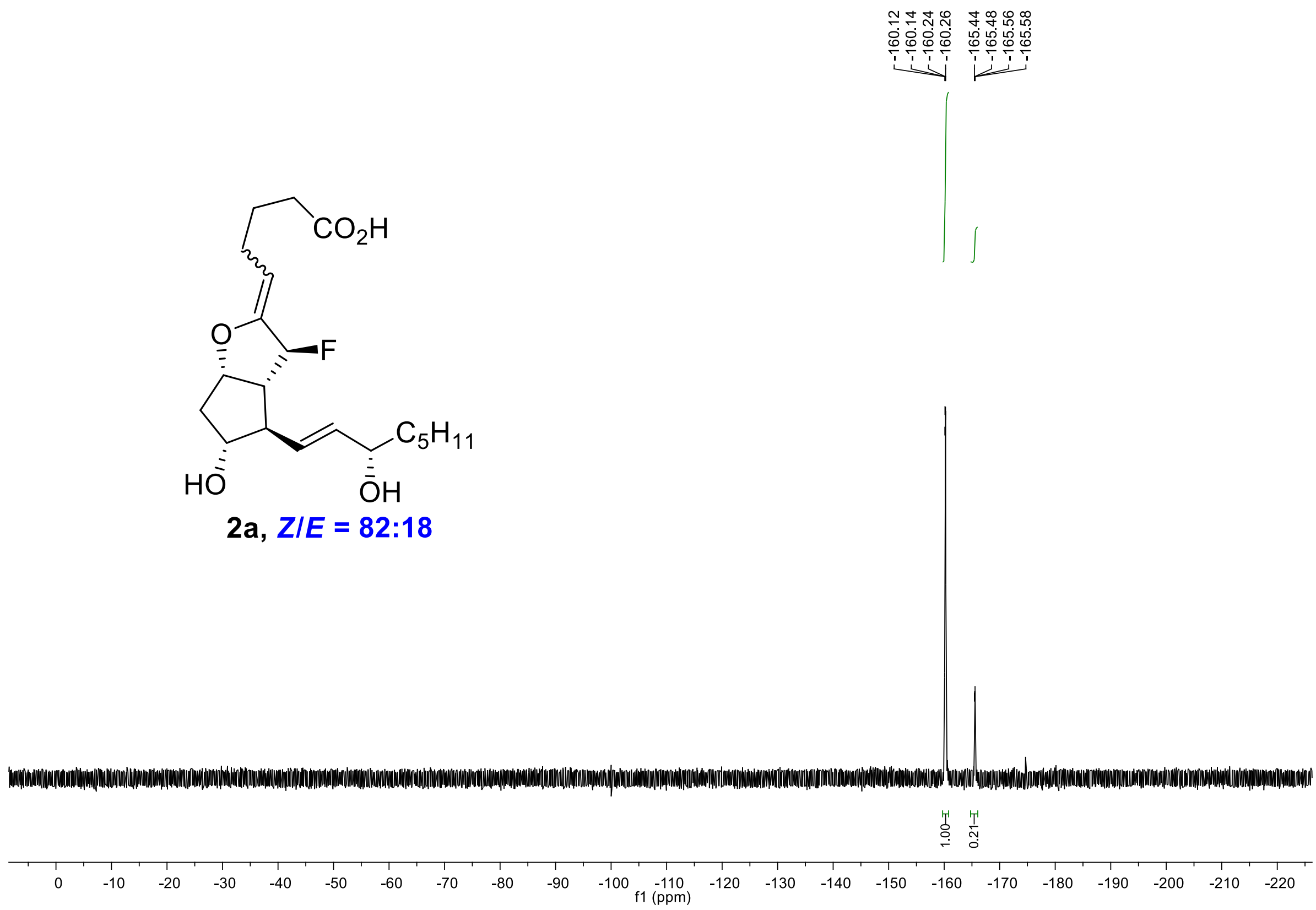

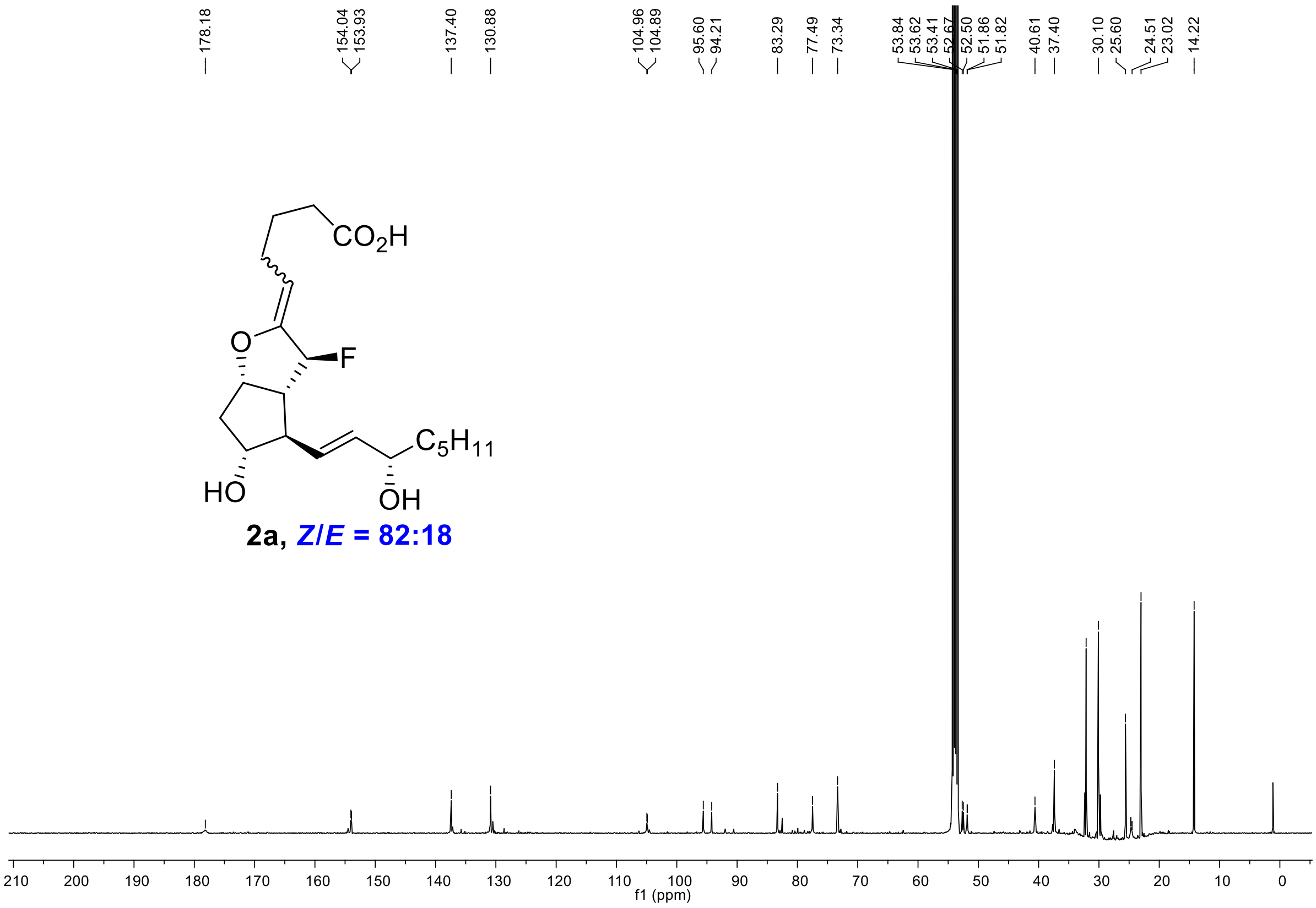

178.18
154.04
153.93
137.40
130.88
104.96
104.89
95.60
94.21
83.29
77.49
73.34
53.84
53.62
53.41
52.50
51.86
51.82
40.61
37.40
30.10
25.60
24.51
23.02
14.22
CO2H
O
F
C5H11
HO
OH
2a, Z/E = 82:18
f1 (ppm)

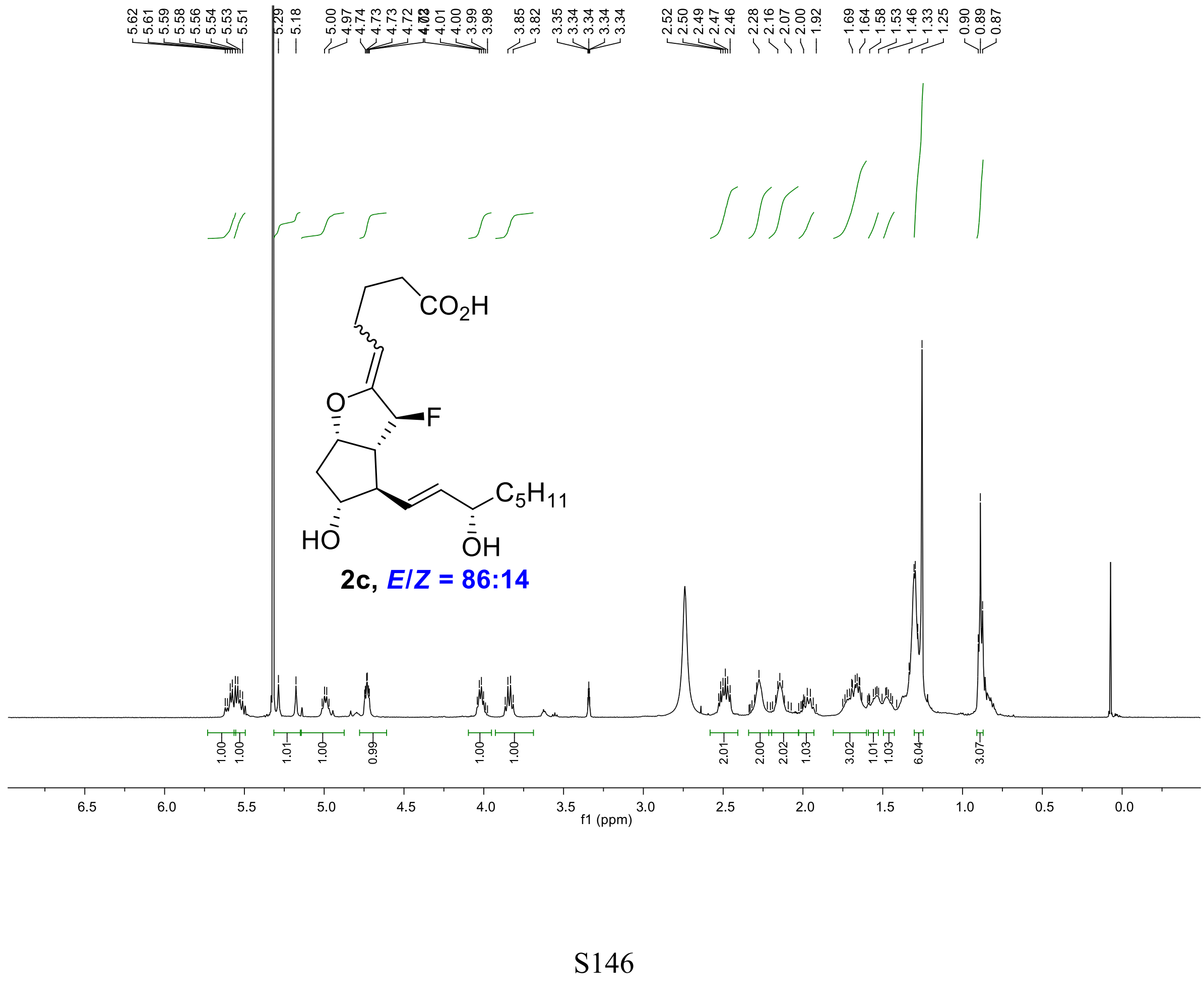

5.62
5.61
5.59
5.58
5.56
5.54
5.53
5.51
5.29
5.18
5.00
4.97
4.74
4.73
4.72
4.01
4.00
3.99
3.98
3.85
3.82
3.35
3.34
3.34
3.34
3.34
2.52
2.50
2.49
2.47
2.46
2.28
2.16
2.07
2.00
1.92
1.69
1.64
1.58
1.53
1.46
1.33
1.25
0.90
0.89
0.87
CO2H
O
F
C5H11
HO
OH
2c, E/Z = 86:14
f1 (ppm)

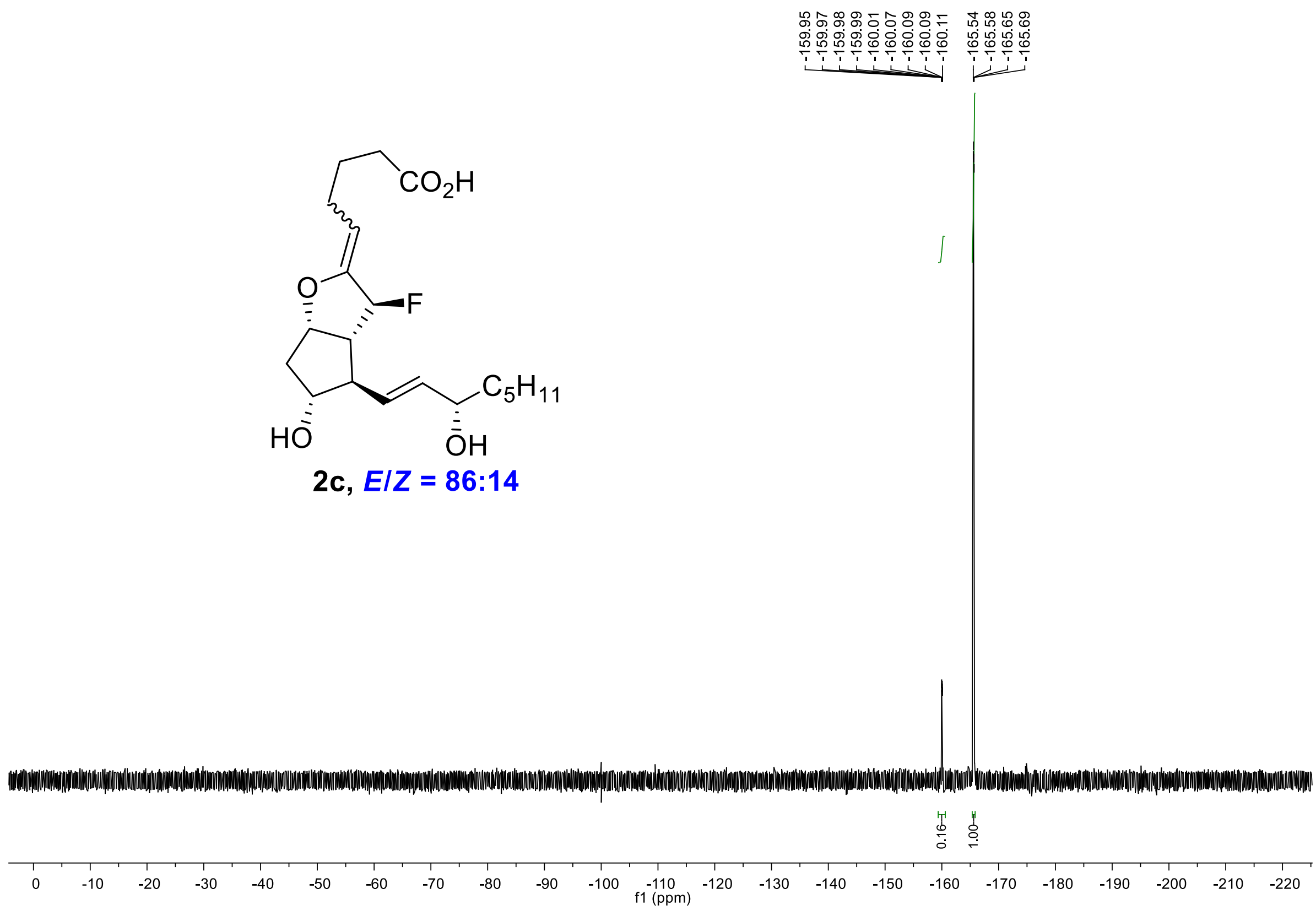

2c, *E*/*Z* = 86:14

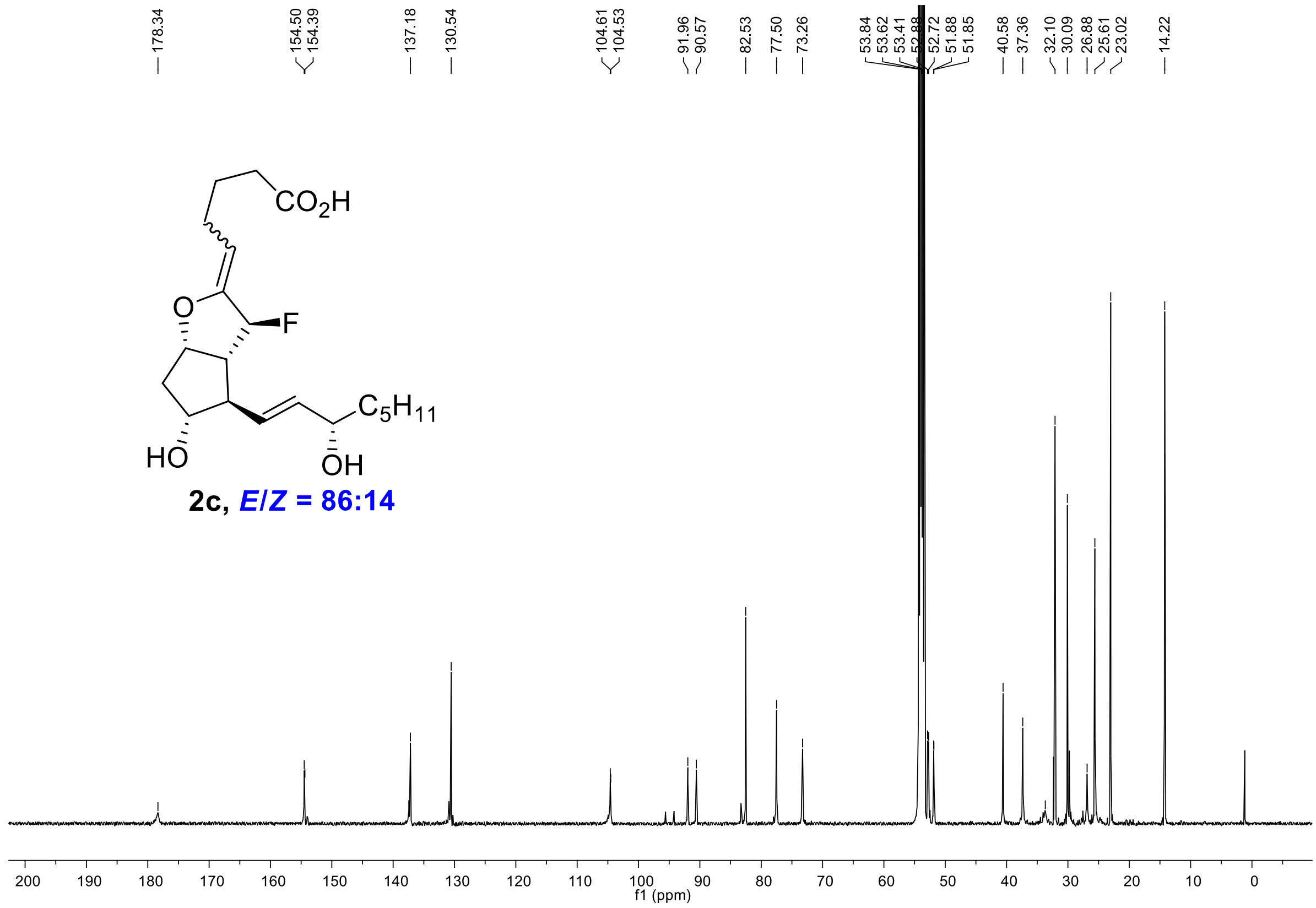

2c, *E*/*Z* = 86:14

(3*S*,3a*R*,4*R*,5*R*,6a*S*)-5-(*tert*-Butyldimethylsilyloxy)-4-((3*S*,4*S*,*E*)-3-(*tert*-butyldimethylsilyloxy)-4-methyloct-1-en-6-ynyl)-3-fluorohexahydro-2*H*-cyclopenta[*b*]furan-2-one (50)

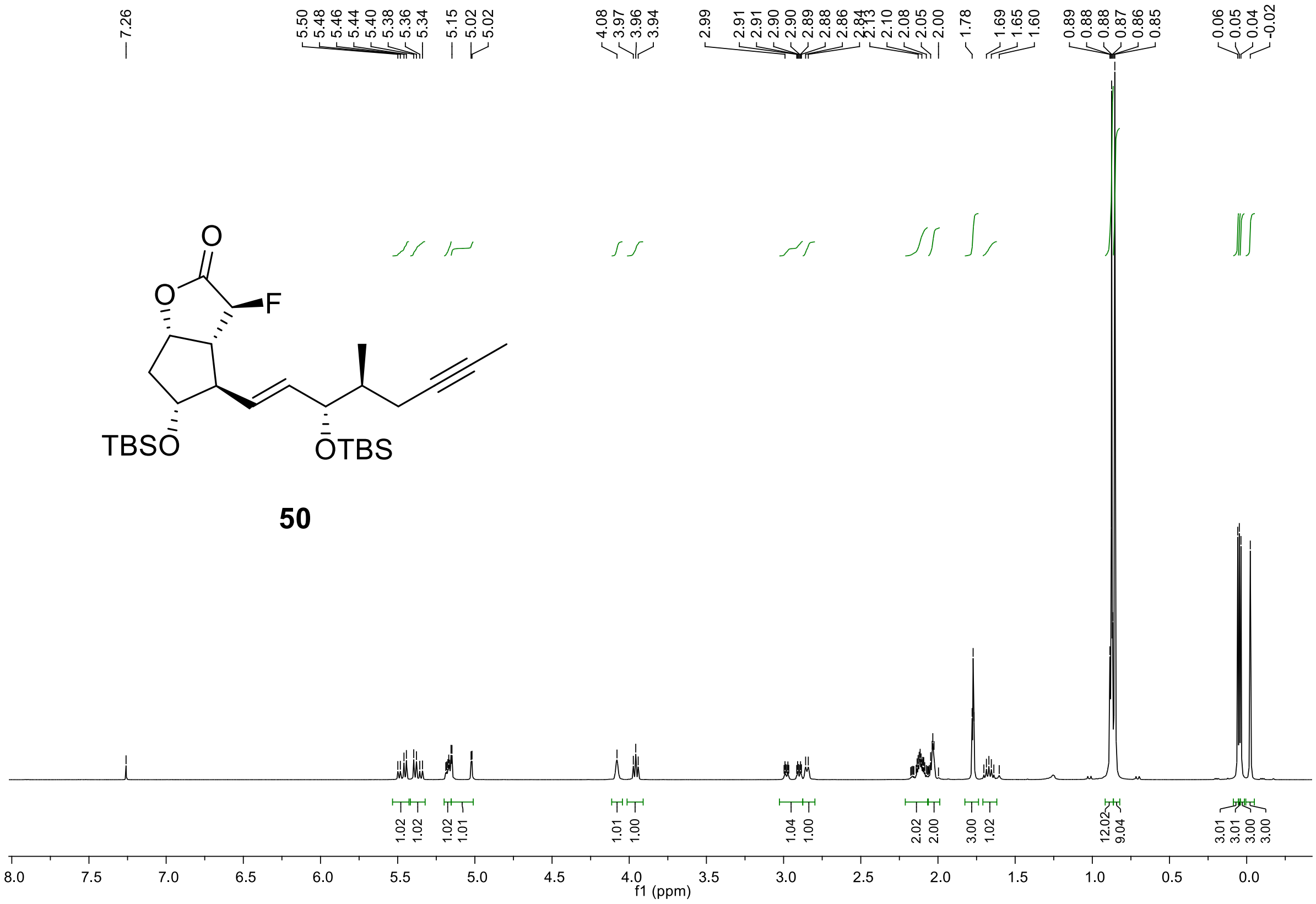

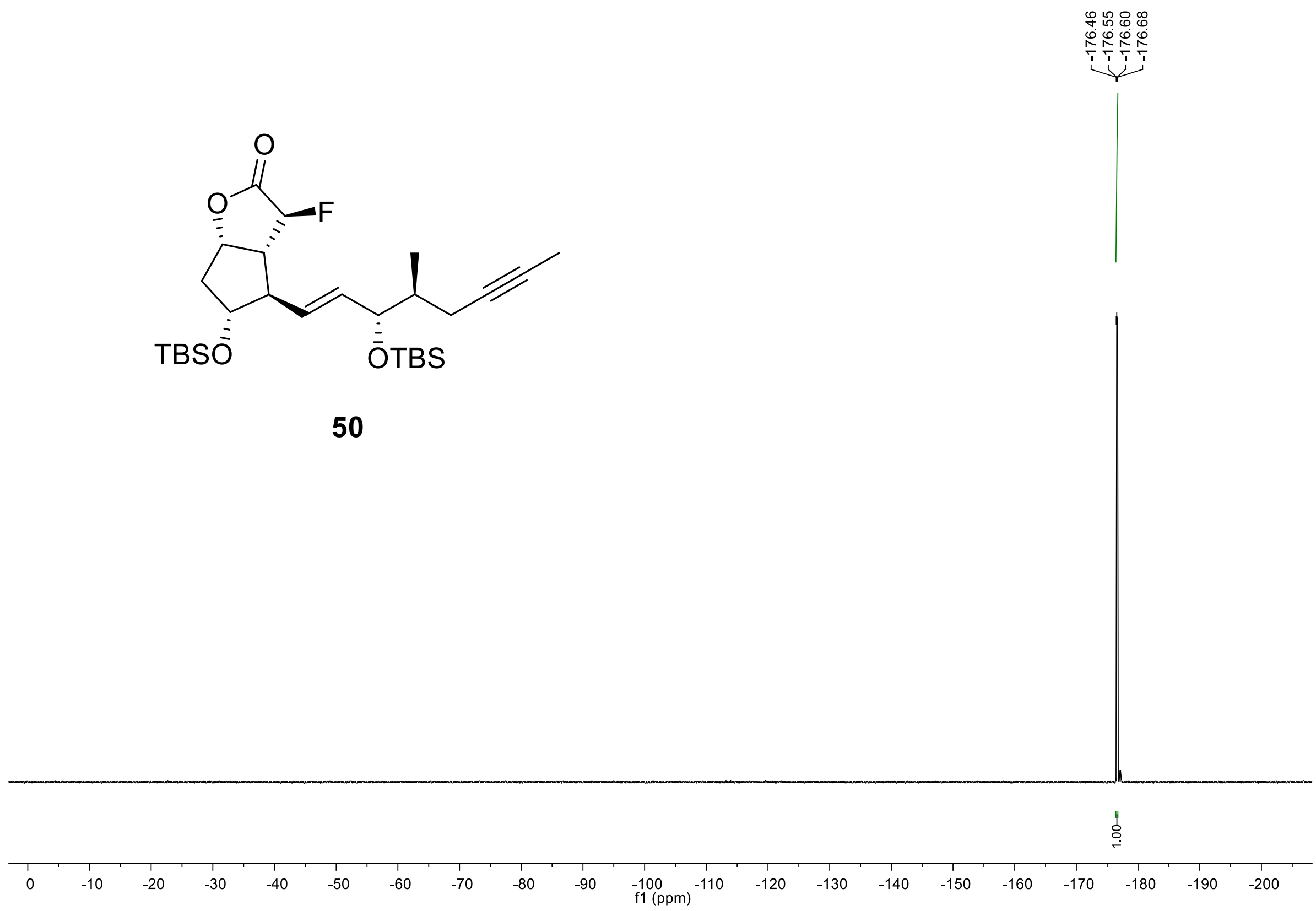

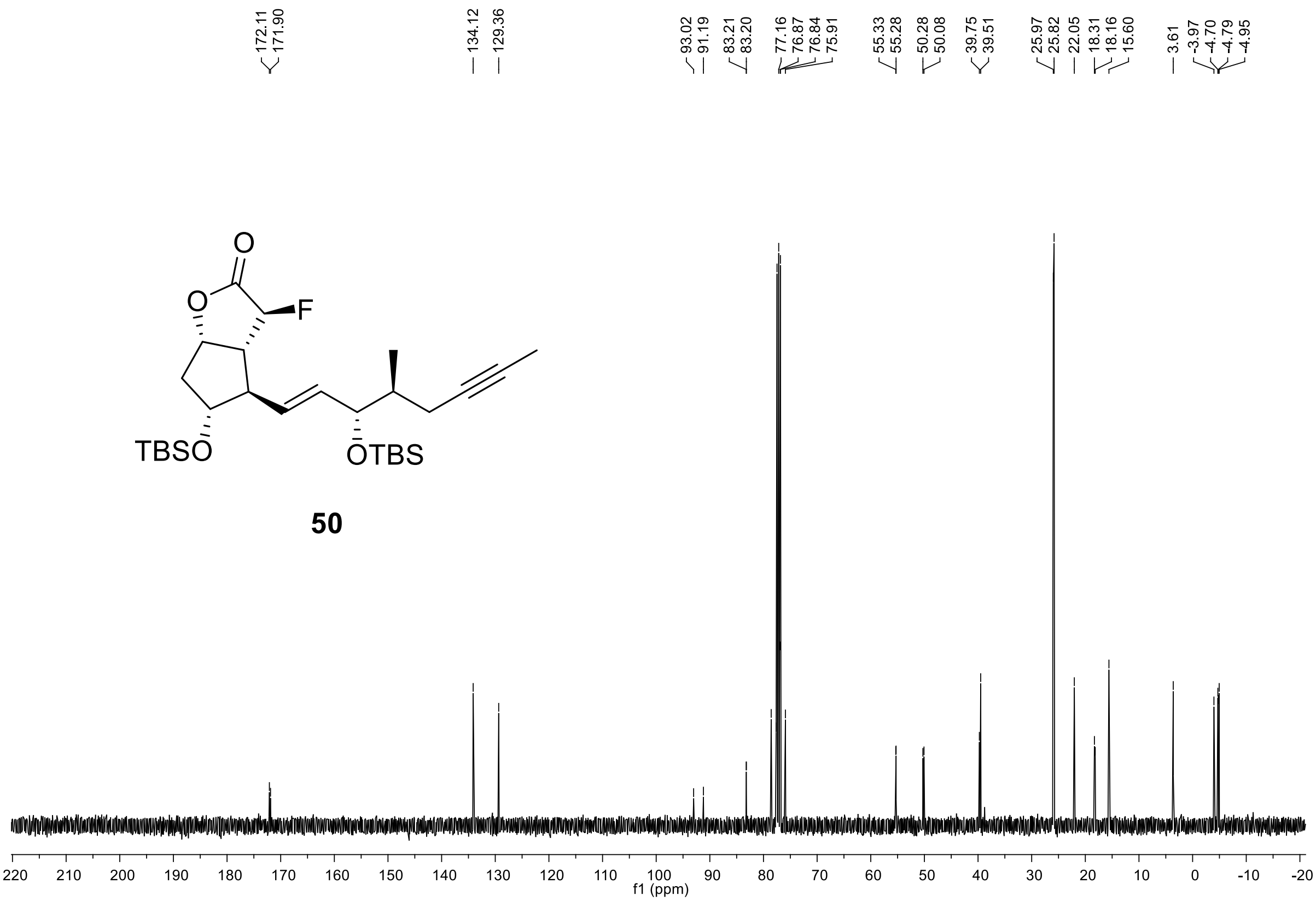
172.11
171.90
134.12
129.36
93.02
91.19
83.21
83.20
77.16
76.87
76.84
75.91
55.33
55.28
50.28
50.08
39.75
39.51
25.97
25.82
22.05
18.31
18.16
15.60
3.61
-3.97
-4.70
-4.79
-4.95
O
O
F
TBSO
OTBS
220 210 200 190 180 170 160 150 140 130 120 110 100 90 80 70 60 50 40 30 20 10 0 -10 -20
f1 (ppm)

50

5-((3*S*,3a*R*,4*R*,5*R*,6a*S*)-5-(*tert*-Butyldimethylsilyloxy)-4-((3*S*,4*S*,*E*)-3-(*tert*-butyldimethylsilyloxy)-4-methyloct-1-en-6-ynyl)-3-fluorohexahydro-2*H*-cyclopenta[*b*]furan-2-ylidene)pentanoic acid (51)

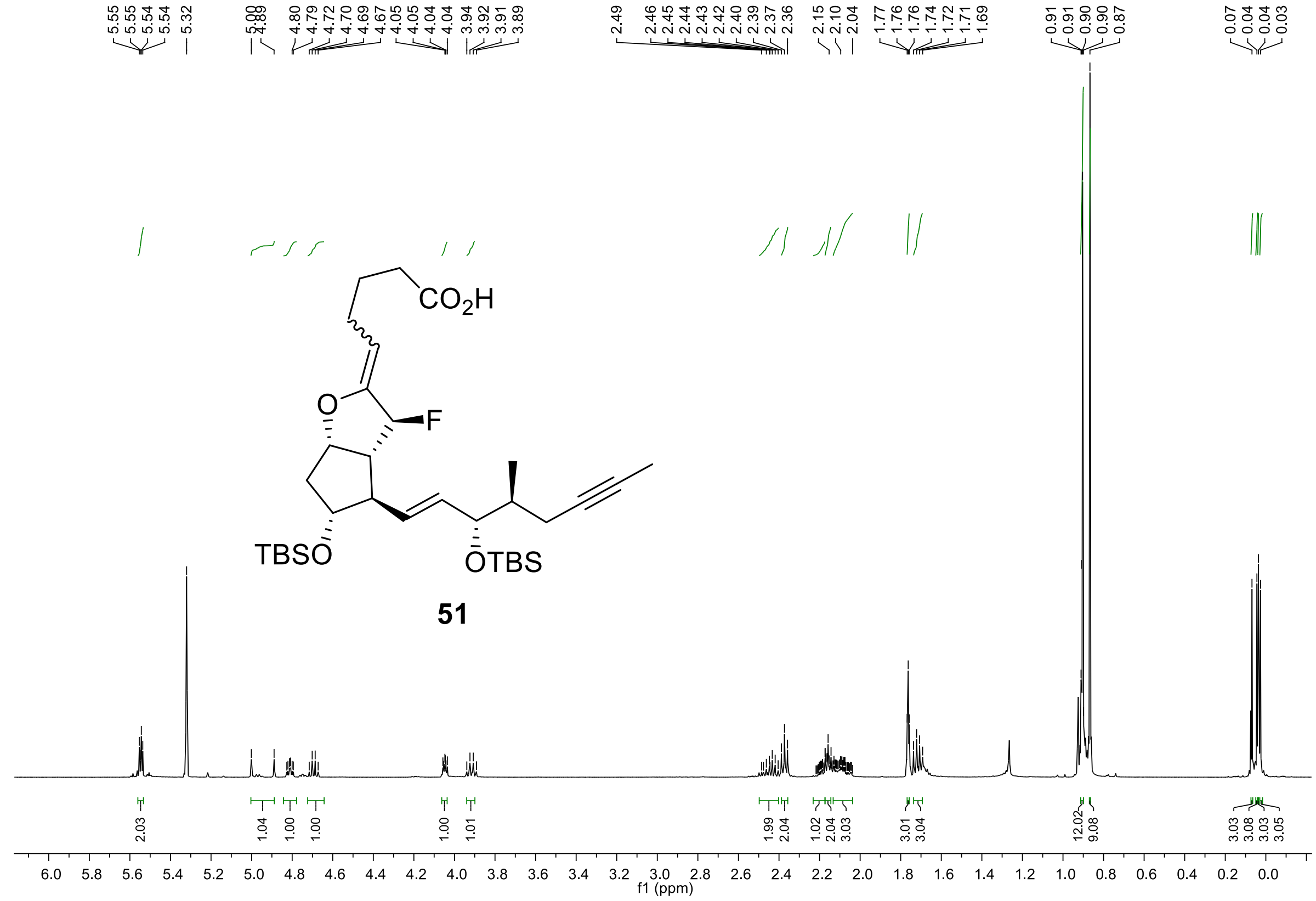

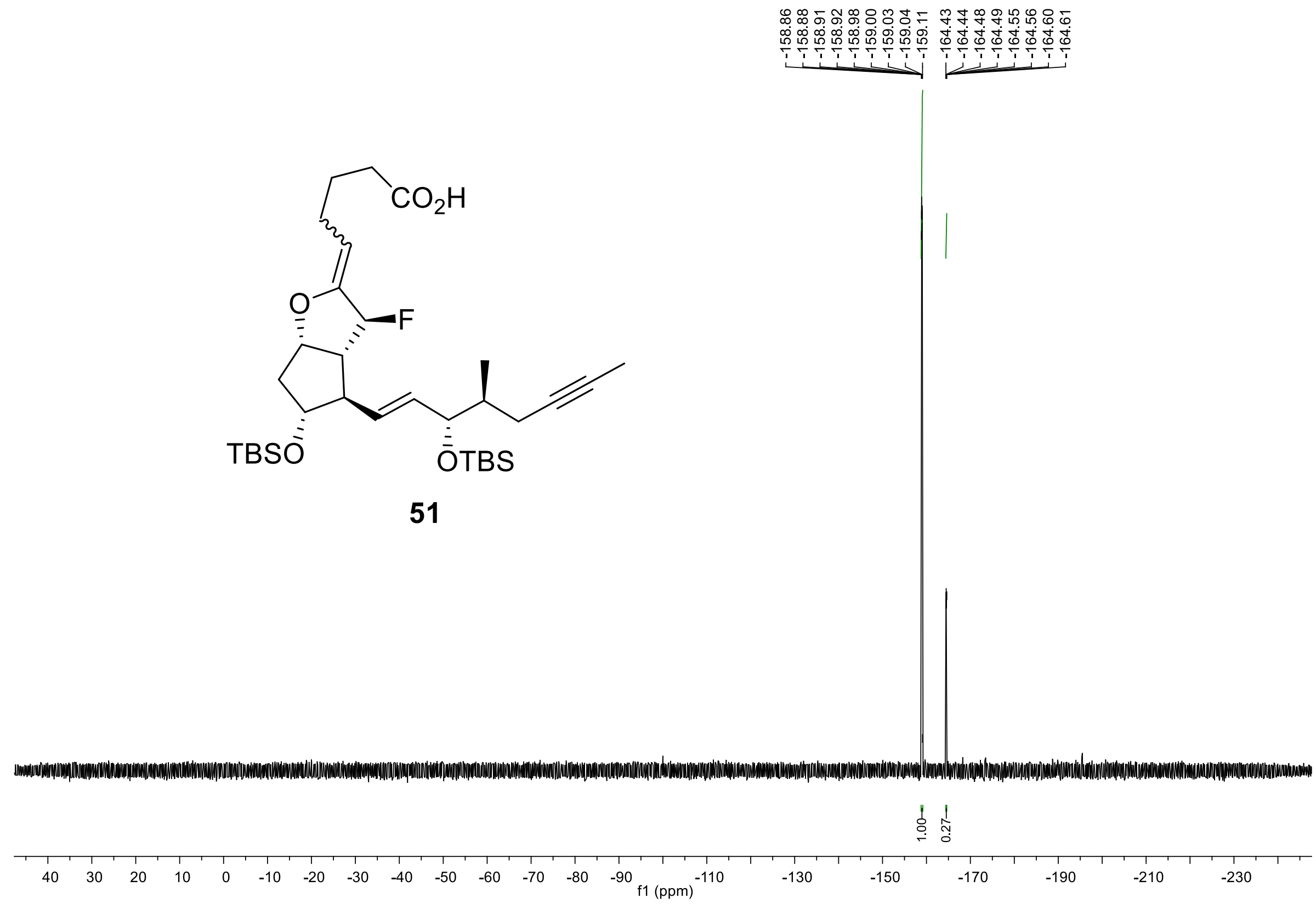

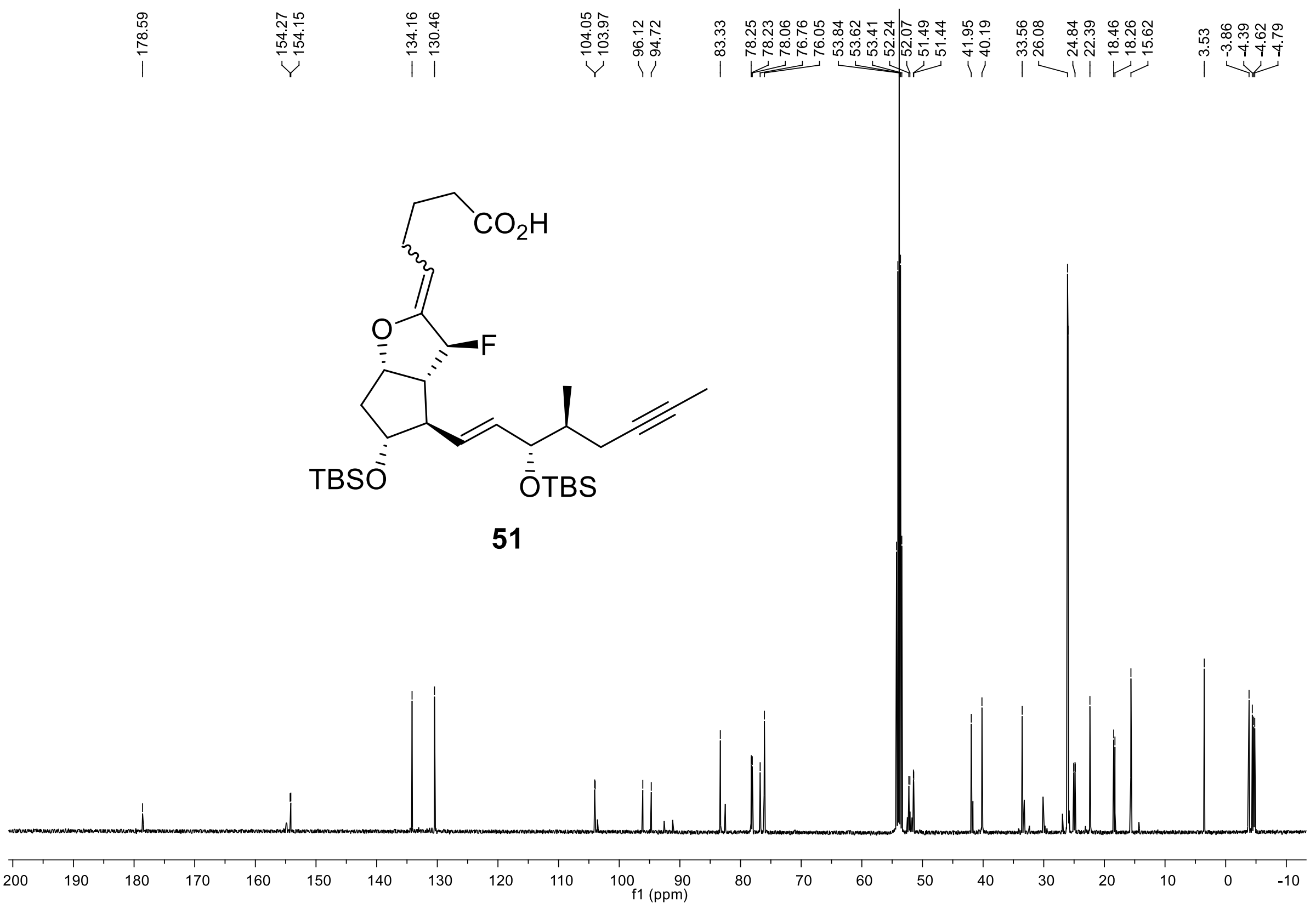
178.59
154.27
154.15
134.16
130.46
104.05
103.97
96.12
94.72
83.33
78.25
78.23
78.06
76.76
76.05
53.84
53.62
53.41
52.24
52.07
51.49
51.44
41.95
40.19
33.56
26.08
24.84
22.39
18.46
18.26
15.62
3.53
-3.86
-4.39
-4.62
-4.79
CO2H
O
F
TBSO
OTBS
51
200
190
180
170
160
150
140
130
120
110
100
90
80
70
60
50
40
30
20
10
0
-10
f1 (ppm)

5-((3*S*,3a*R*,4*R*,5*R*,6a*S*)-3-Fluoro-5-hydroxy-4-((3*S*,4*S*,*E*)-3-hydroxy-4-methyloct-1-en-6-ynyl)hexahydro-2*H*-cyclopenta[*b*]furan-2-ylidene)pentanoic acid (3)

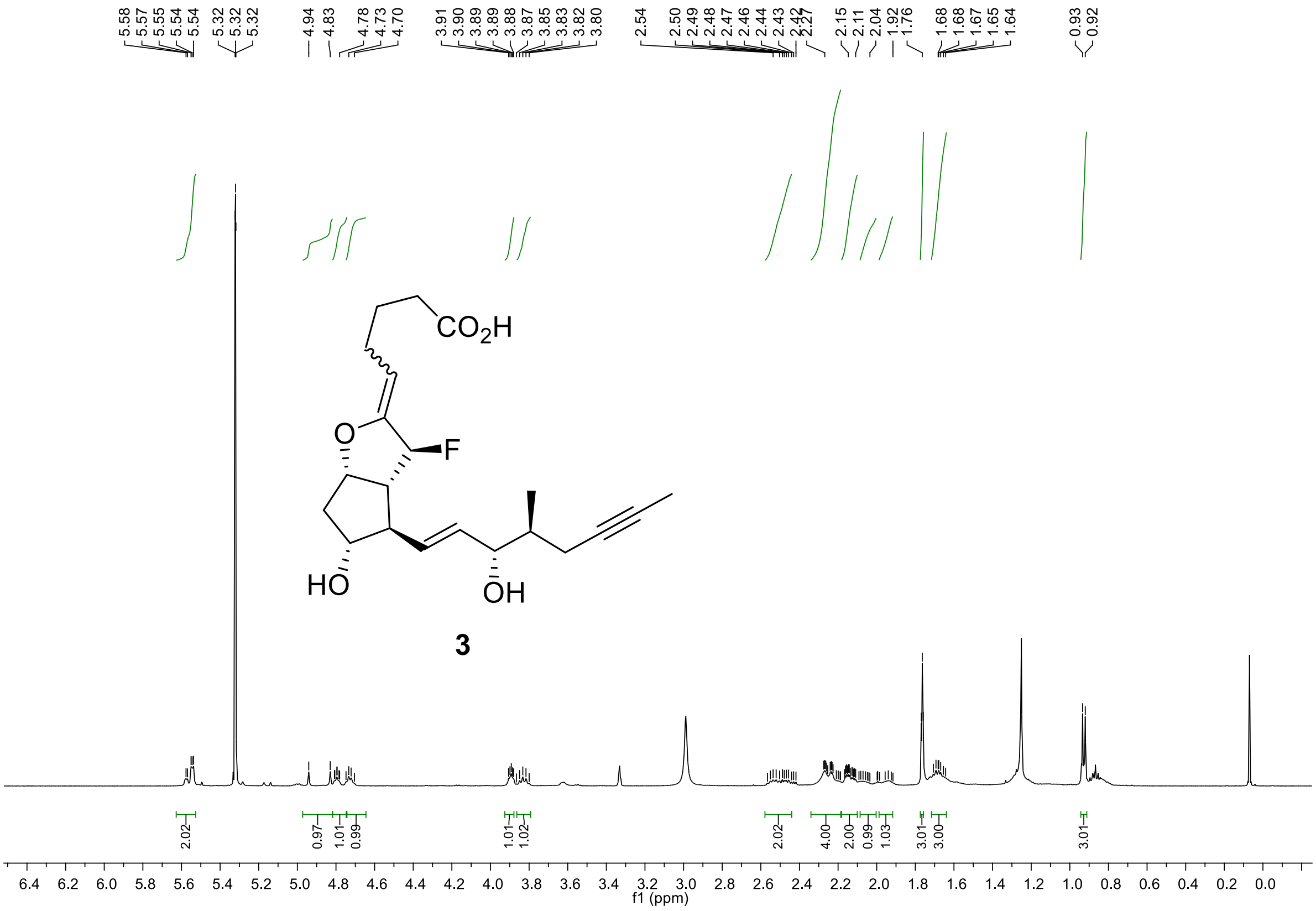

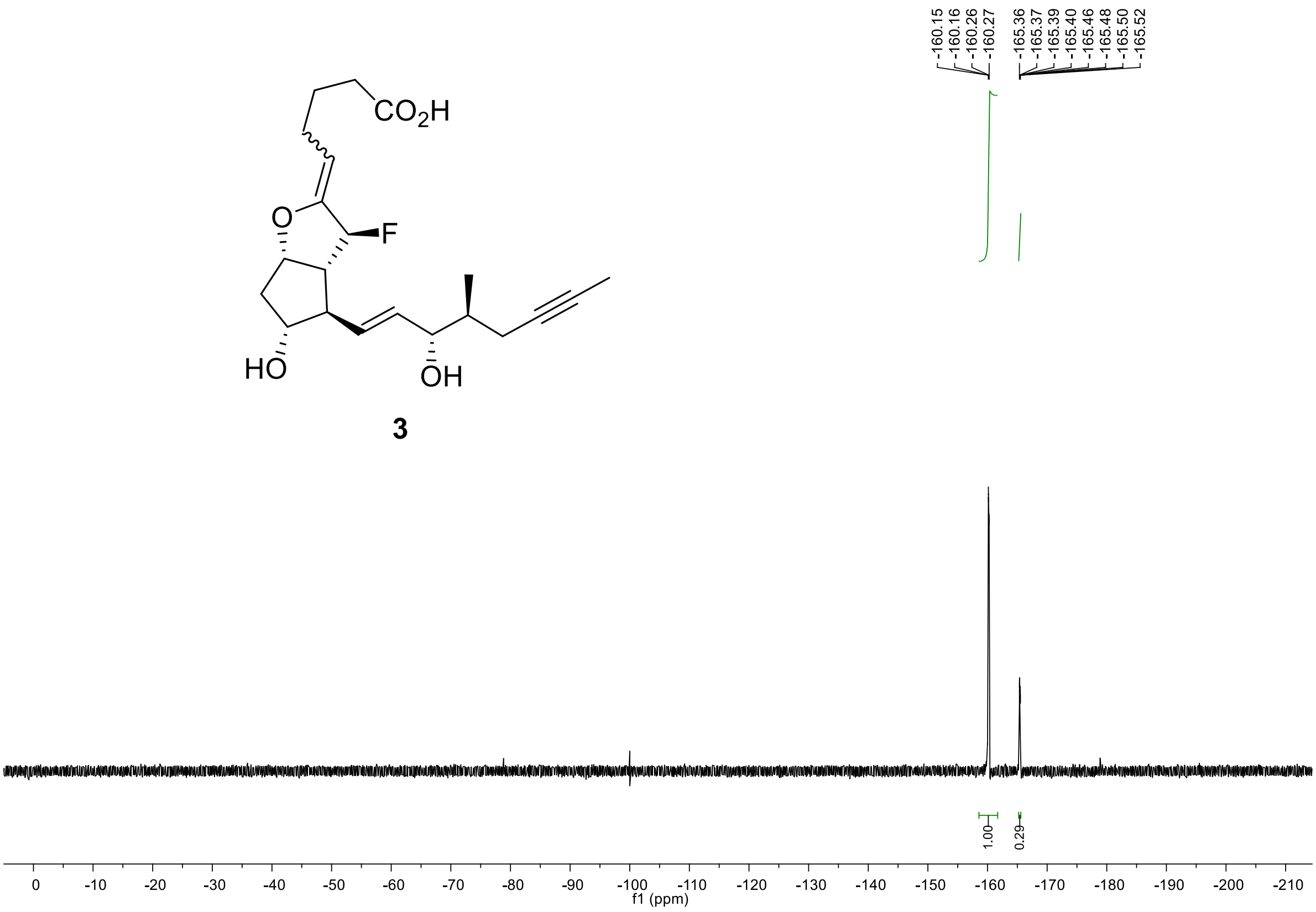

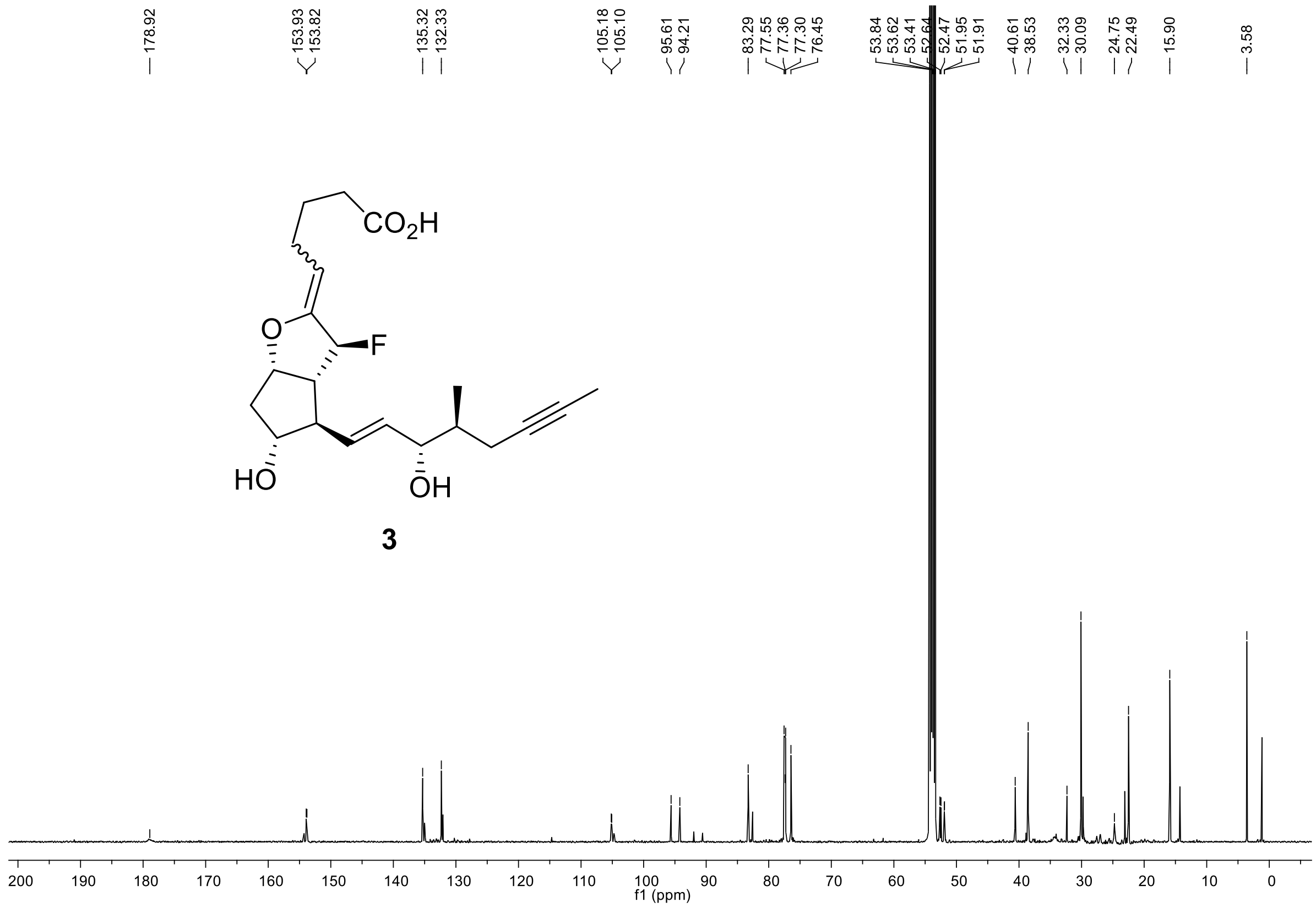
178.92
153.93
153.82
135.32
132.33
105.18
105.10
95.61
94.21
83.29
77.55
77.36
77.30
76.45
53.84
53.62
53.41
52.64
52.47
51.95
51.91
40.61
38.53
32.33
30.09
24.75
22.49
15.90
3.58
CO2H
O
F
HO
OH
3
f1 (ppm)

(3a*R*,4*S*,5*R*,6a*S*)-4-((*S*)-3-(*tert*-Butyldimethylsilyloxy)oct-1-ynyl)-5-hydroxyhexahydro-2*H*-cyclopenta[*b*]furan-2-one (31)

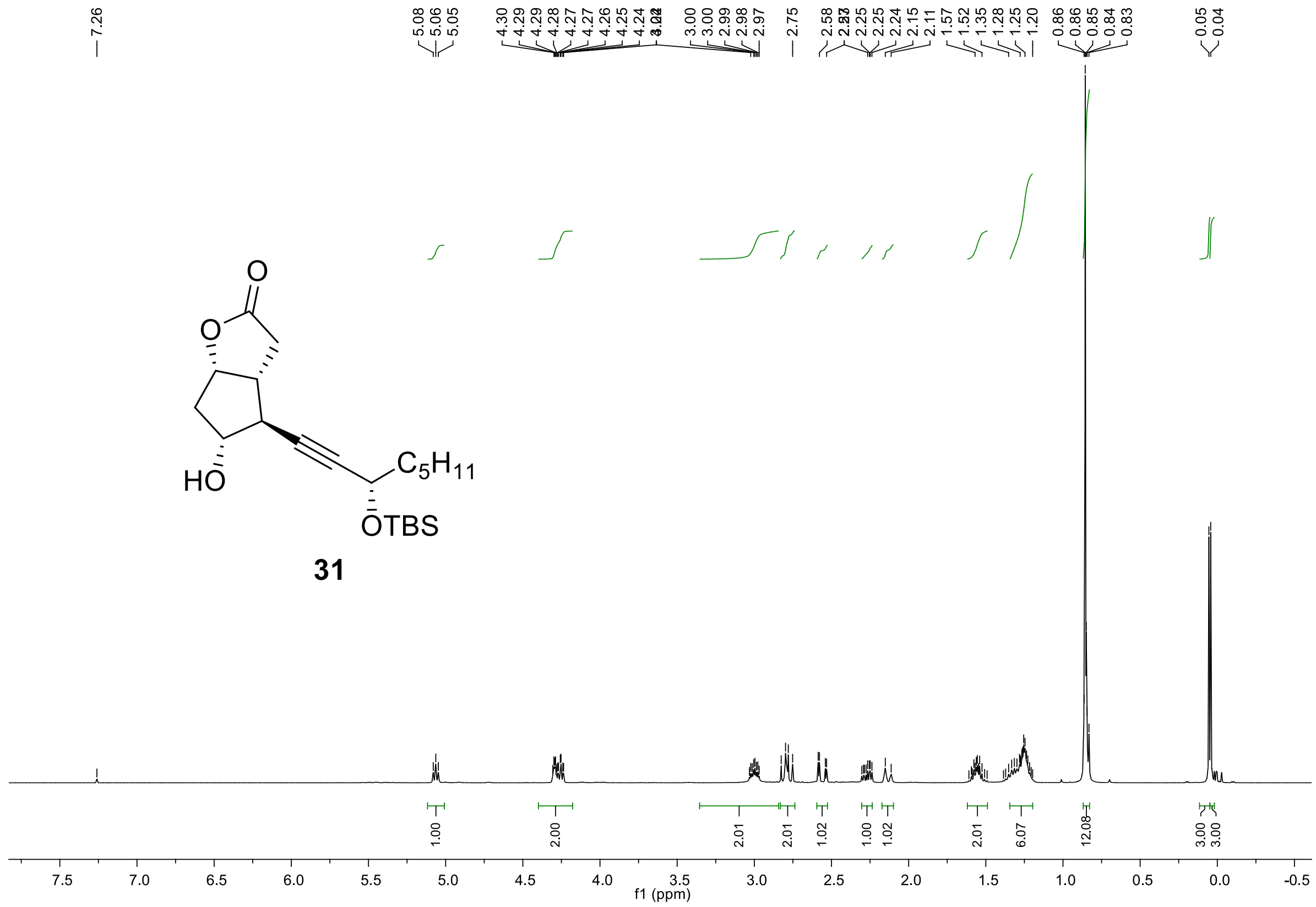

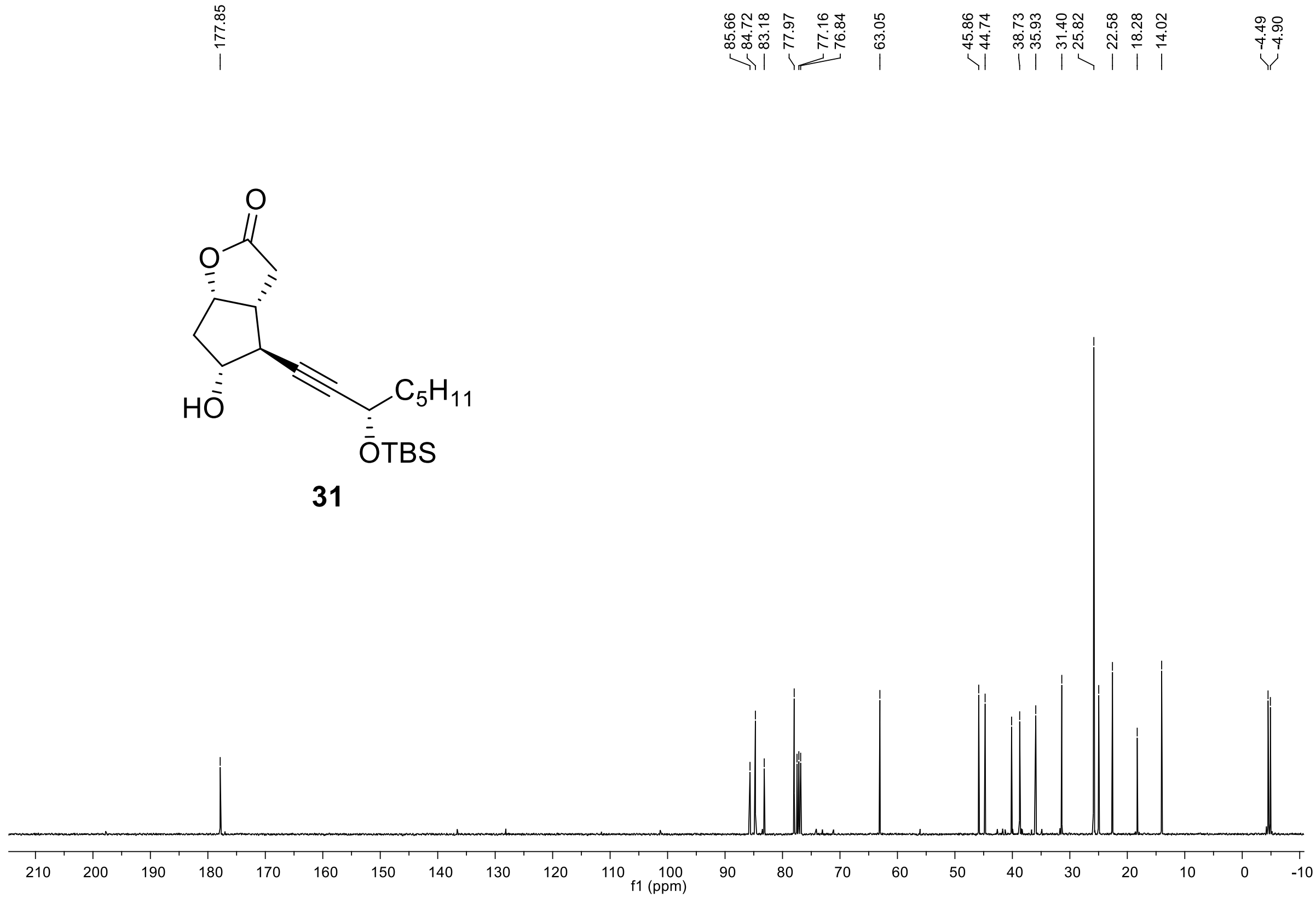

(3a*R*,4*S*,5*R*,6a*S*)-5-(*tert*-Butyldimethylsilyloxy)-4-((*S*)-3-(*tert*-Butyldimethylsilyloxy)oct-1-ynyl)hexahydro-2*H*-cyclopenta[*b*]furan-2-one (32)

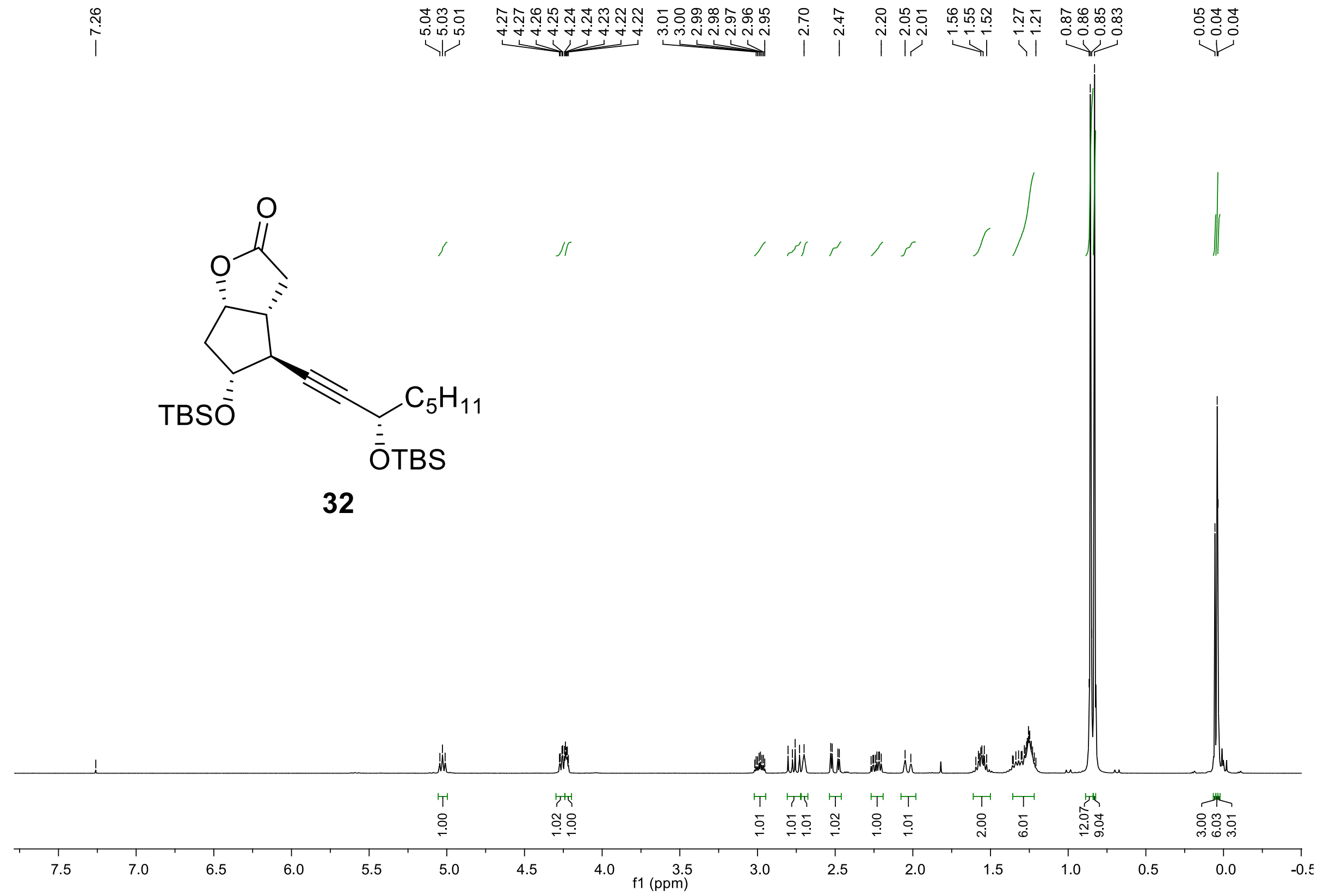

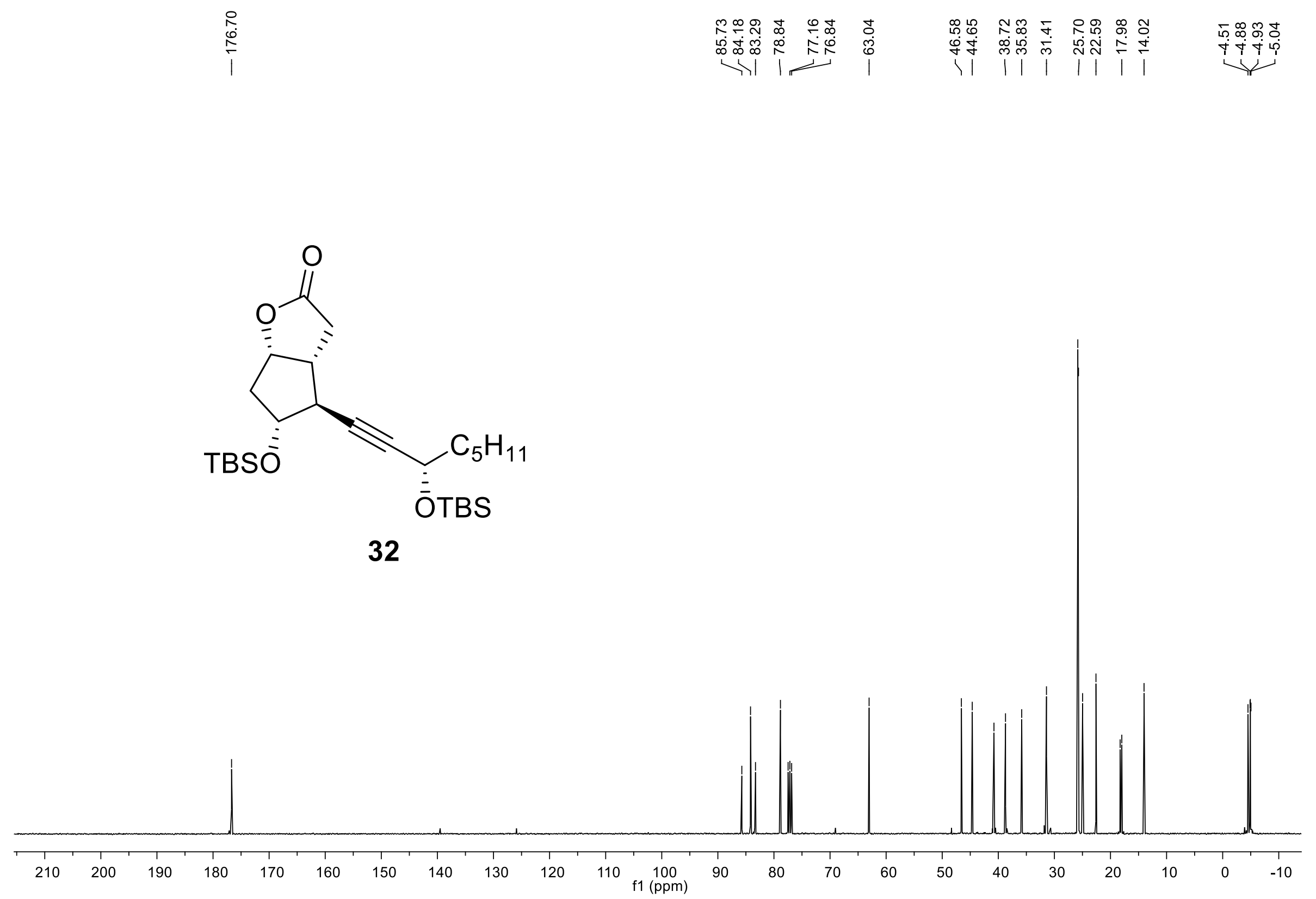

(3a*R*,4*S*,5*R*,6a*S*)-4-((3*S*,4*S*)-3-(*tert*-Butyldimethylsilyloxy)-4-methylnona-1,6-diynyl)-5-hydroxyhexahydro-2*H*-cyclopenta[*b*]furan-2-one (35)

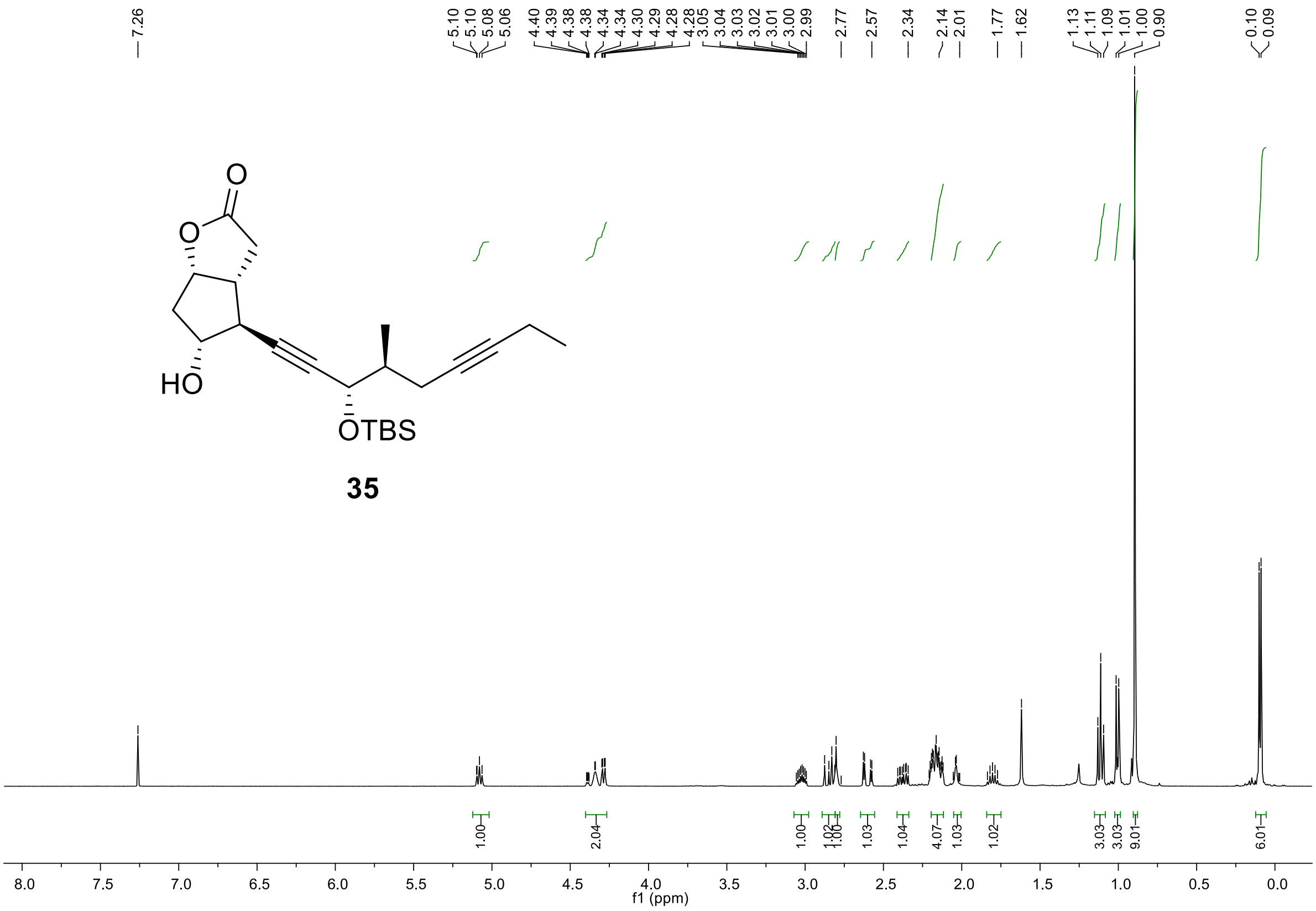

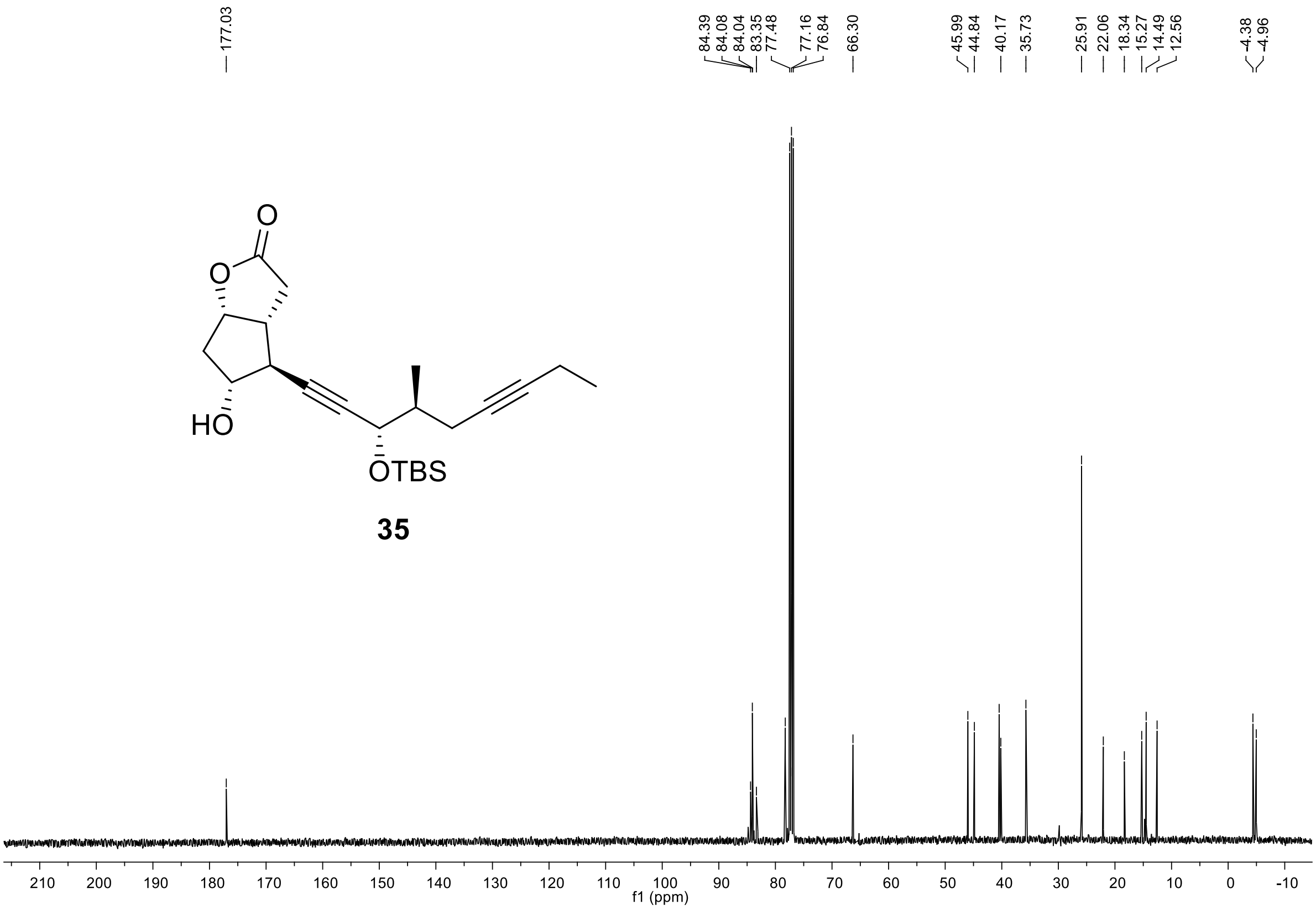

(3a*R*,4*S*,5*R*,6a*S*)-5-(*tert*-Butyldimethylsilyloxy)-4-((3S,4S)-3-(*tert*-butyldimethylsilyloxy)-4-methylnona-1,6-diynyl)hexahydro-2*H*-cyclopenta[*b*]furan-2-one (36)

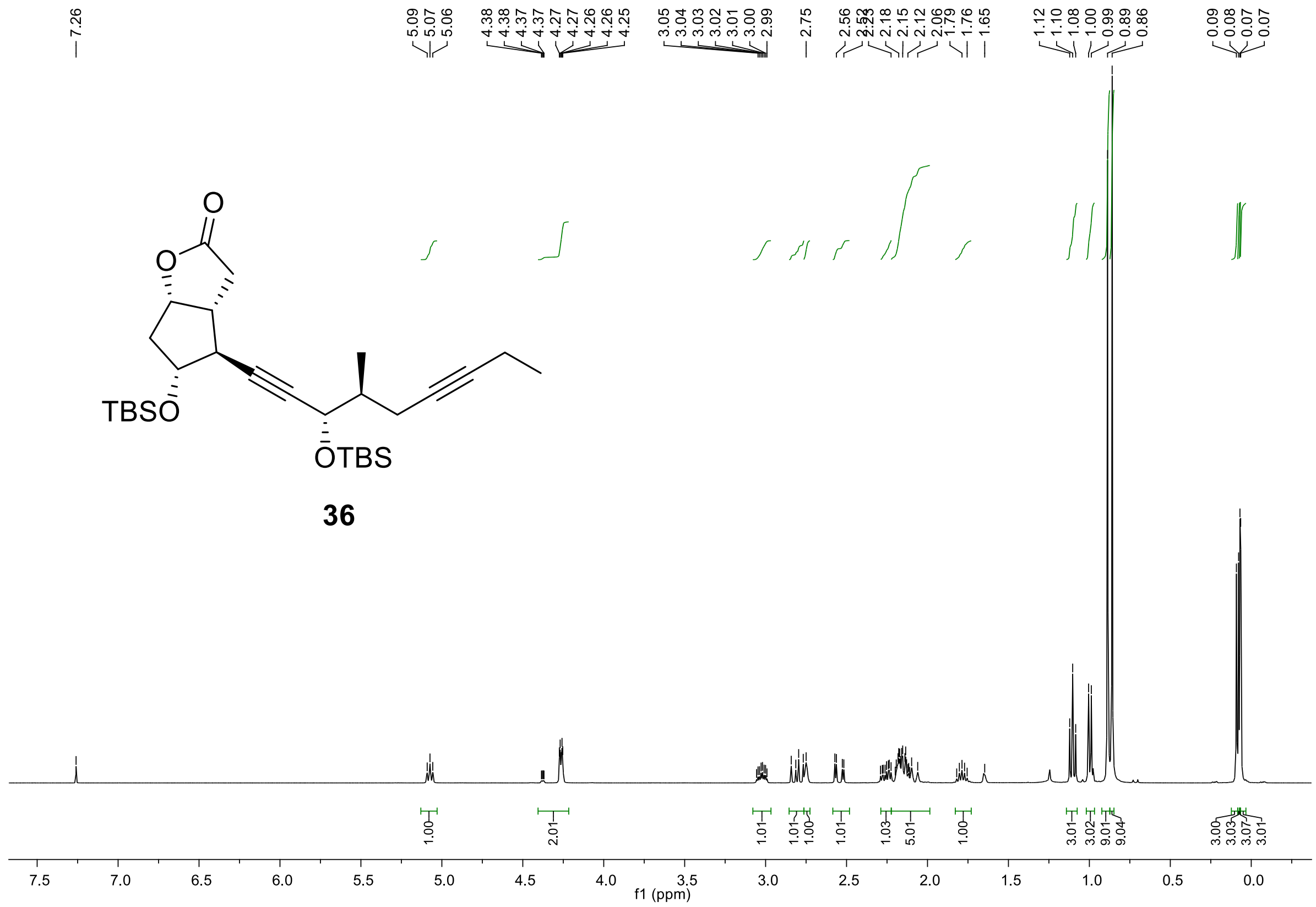

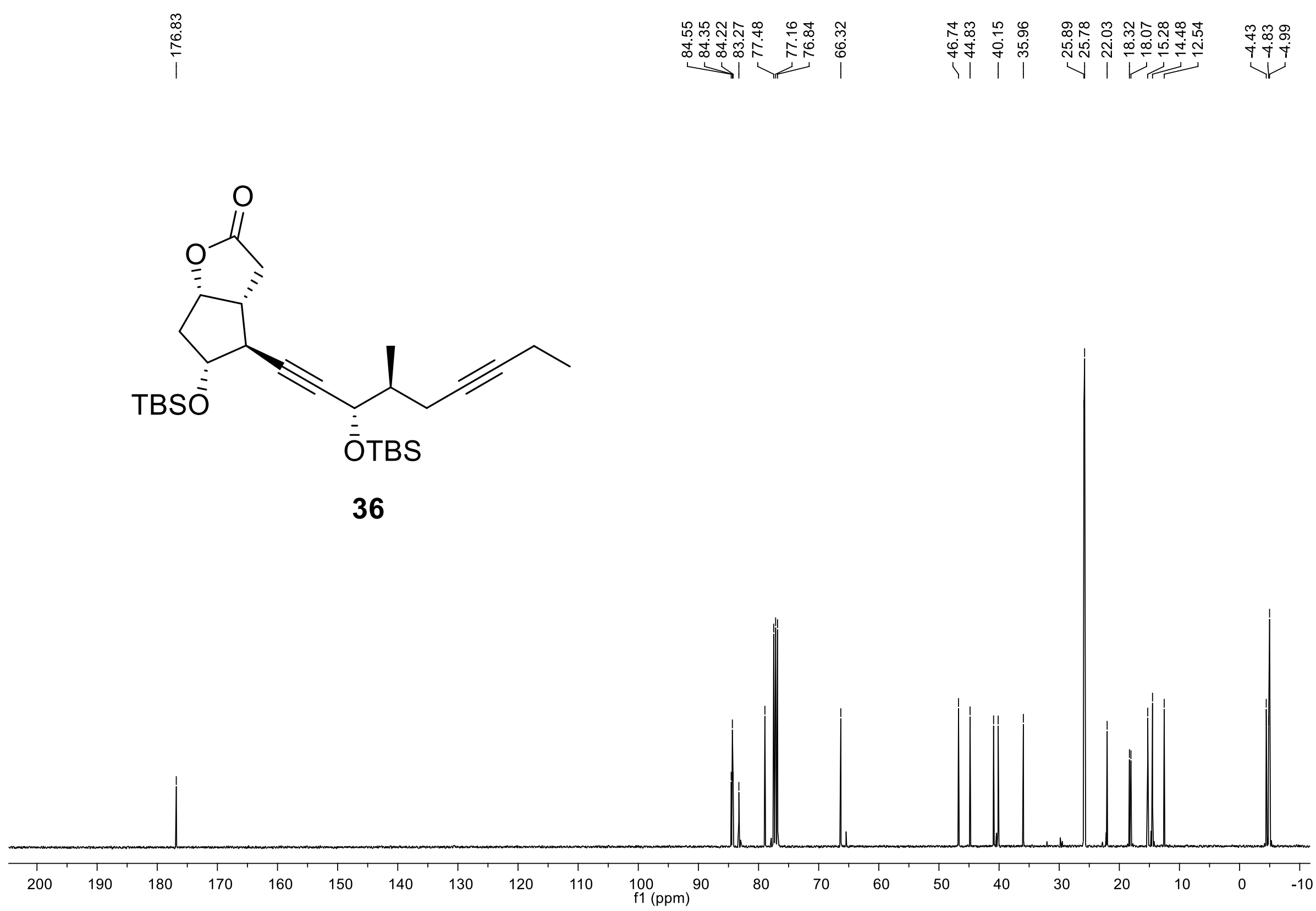

(3a*R*,4*S*,5*R*,6a*S*)-5-(*tert*-Butyldimethylsilyloxy)-4-((*S*)-3-(*tert*-butyldimethylsilyloxy)oct-1-ynyl)-3,3-difluorohexahydro-2*H*-cyclopenta[*b*]furan-2-one (41)

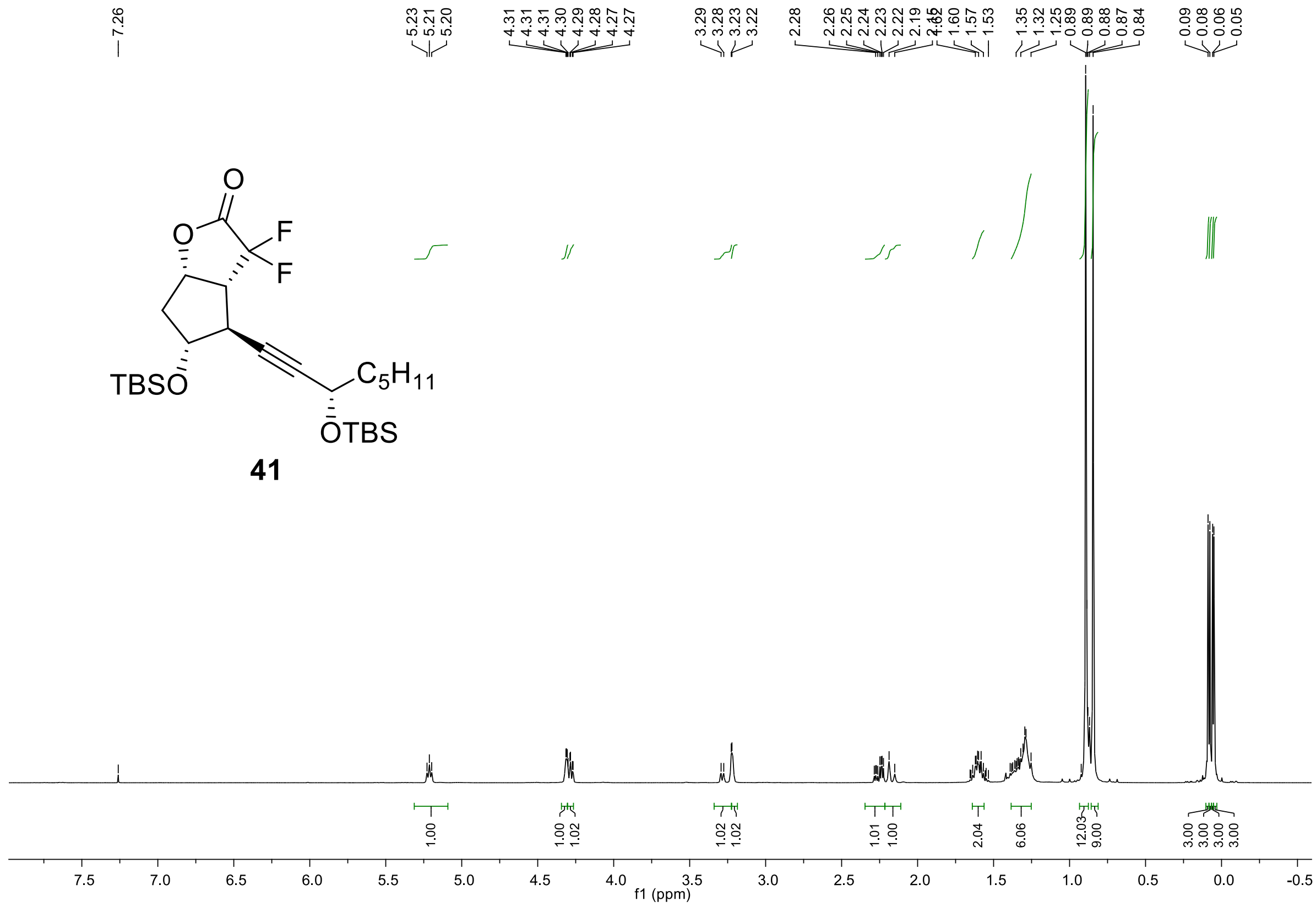

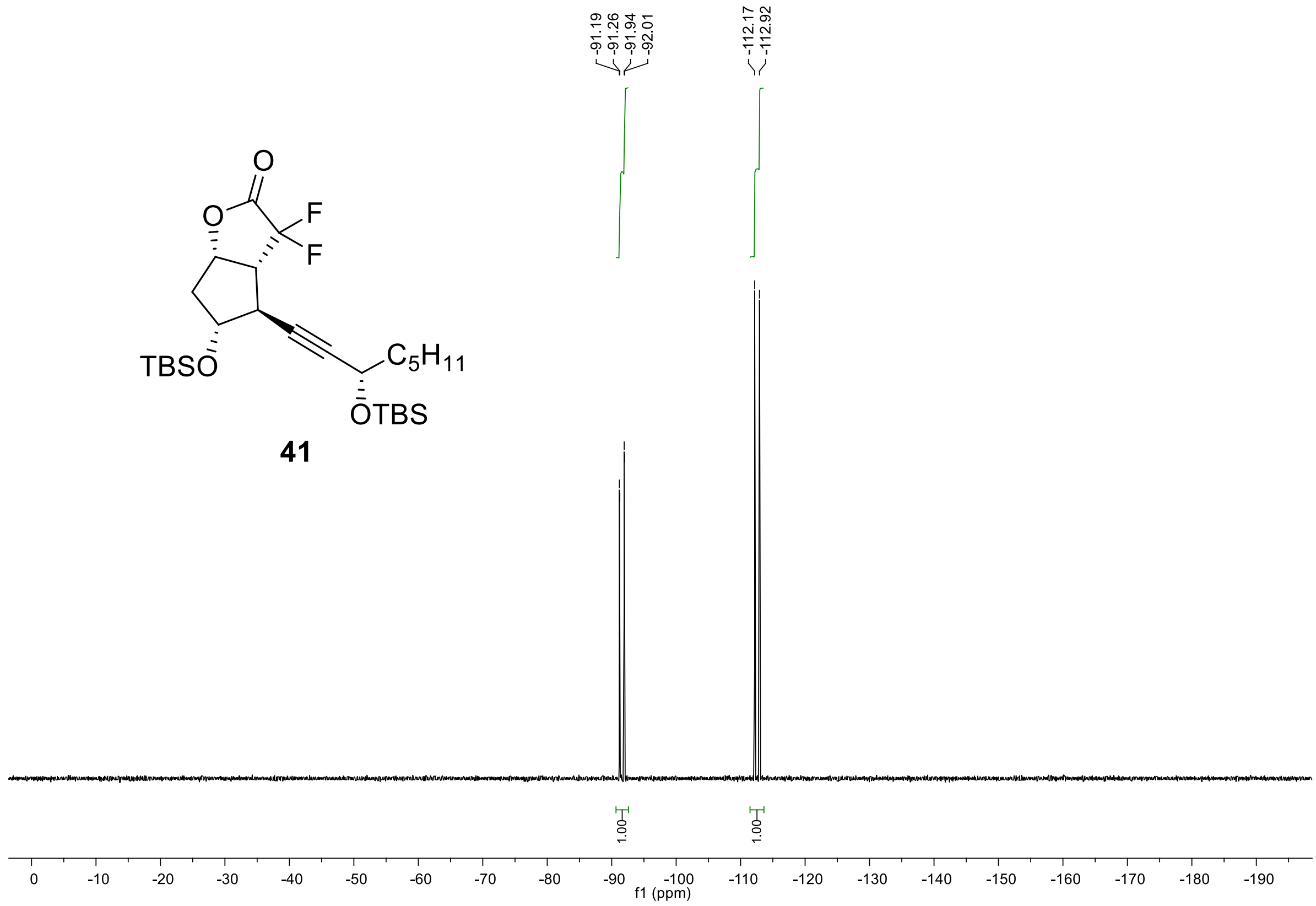

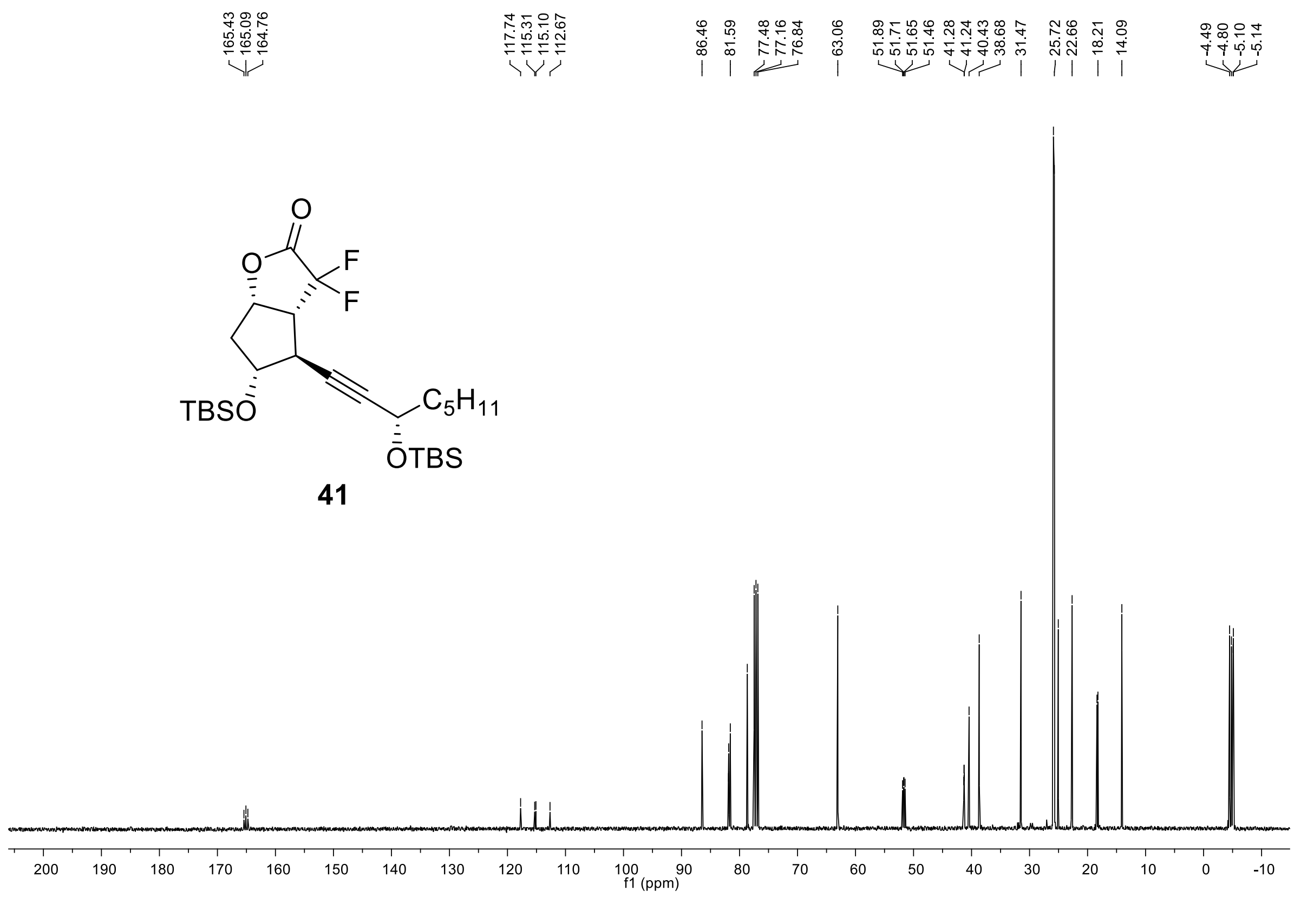
165.43
165.09
164.76
117.74
115.31
115.10
112.67
86.46
81.59
77.48
77.16
76.84
63.06
51.89
51.71
51.65
51.46
41.28
41.24
40.43
38.68
31.47
25.72
22.66
18.21
14.09
-4.49
-4.80
-5.10
-5.14
TBSO
OTBS
C_5H_{11}
41
f1 (ppm)

5-((3a*R*,4*S*,5*R*,6a*S*)-5-(*tert*-Butyldimethylsilyloxy)-4-((*S*)-3-(*tert*-butyldimethylsilyloxy)oct-1-ynyl)-3,3-difluorohexahydro-2*H*-cyclopenta[*b*]furan-2-ylidene)pentanoic acid (42)

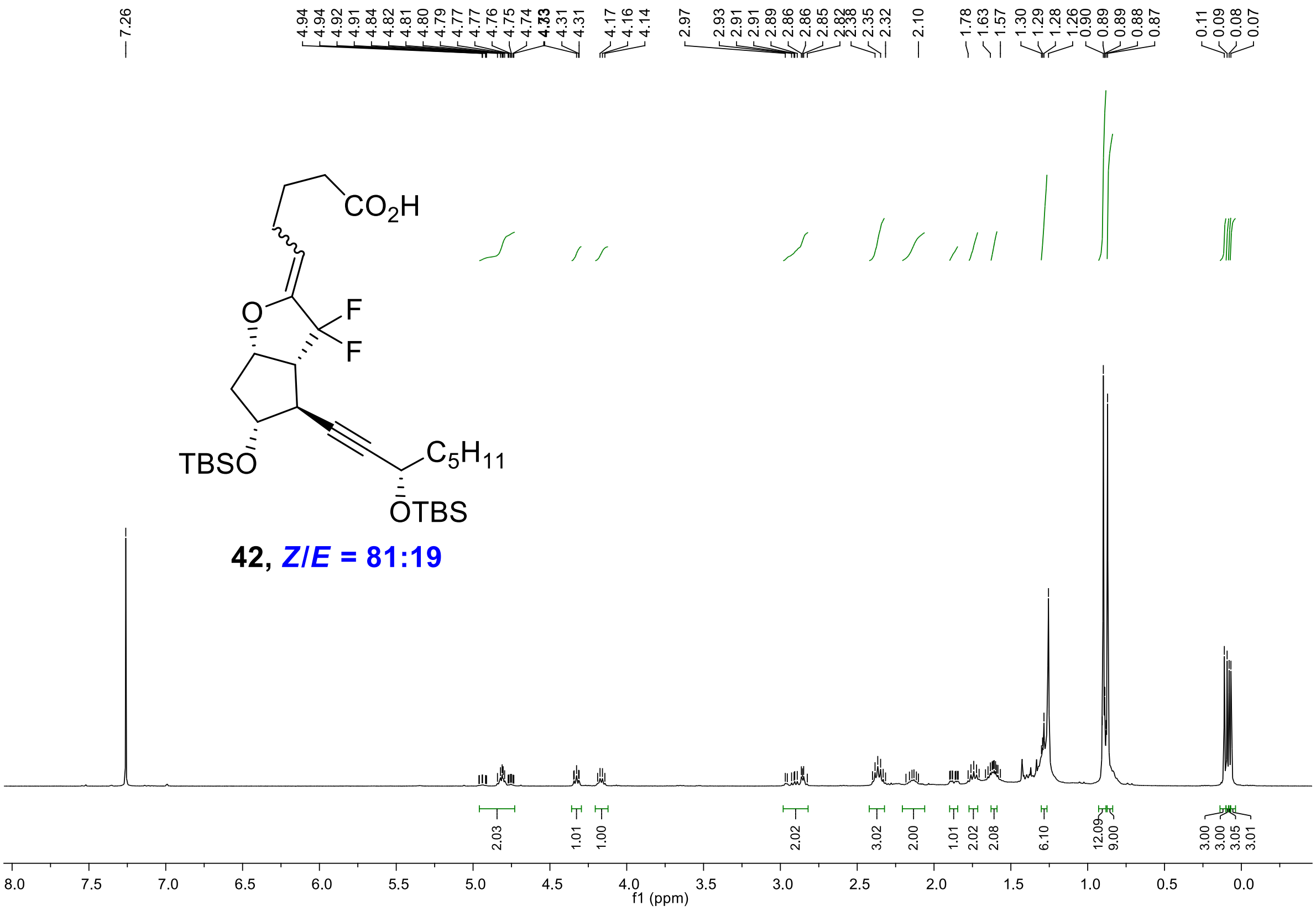

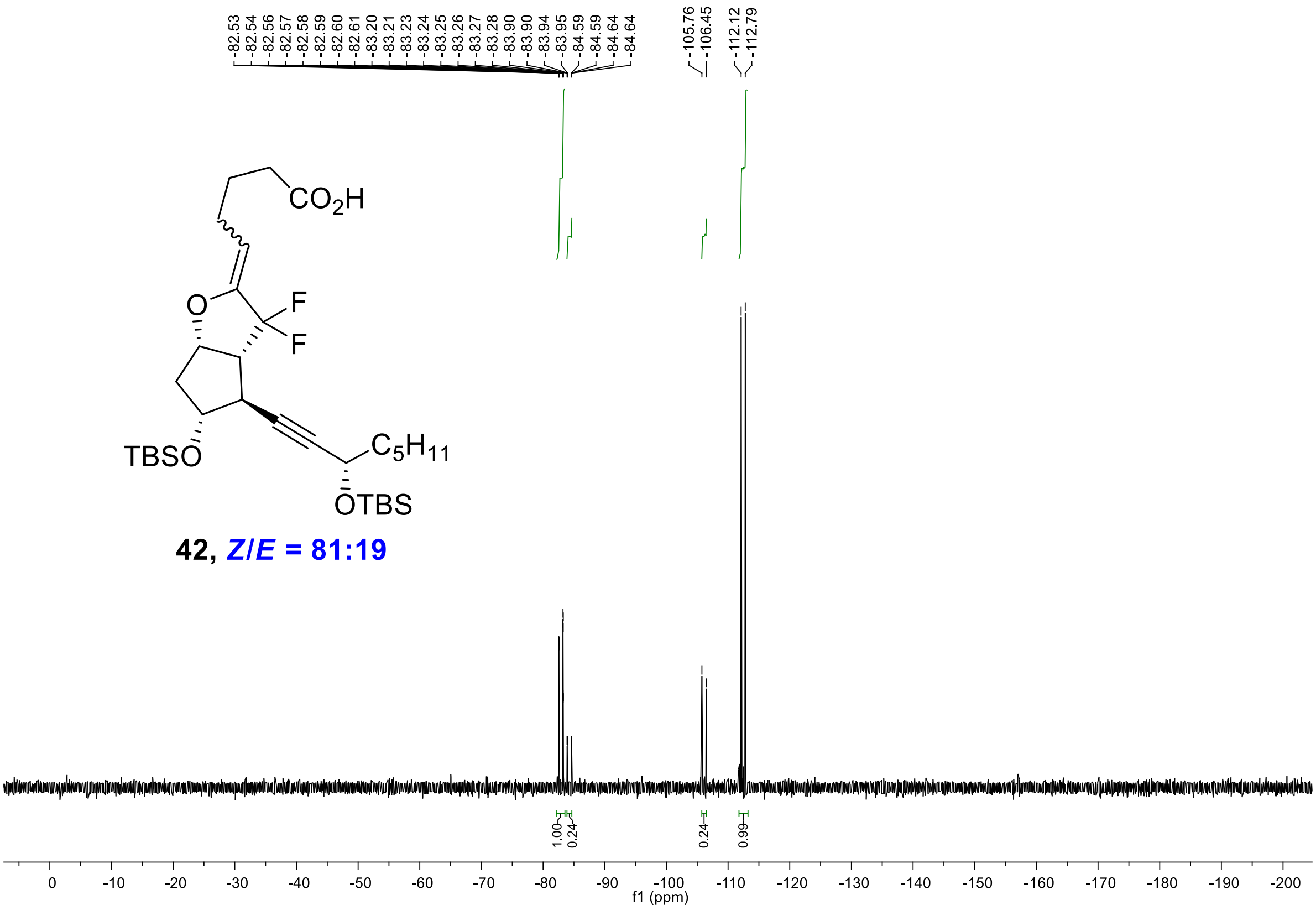

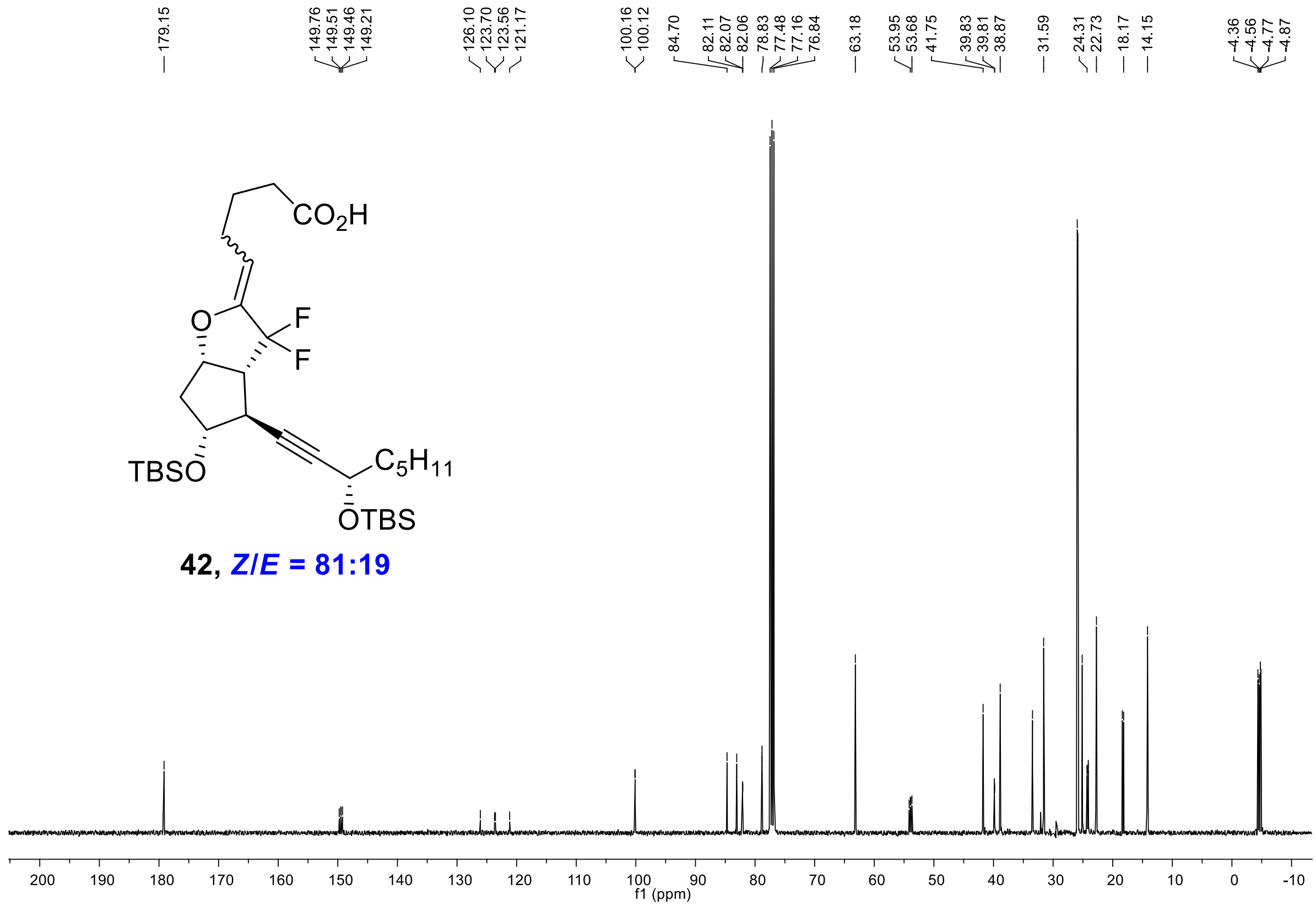

42, ***Z*/*E* = 81:19**

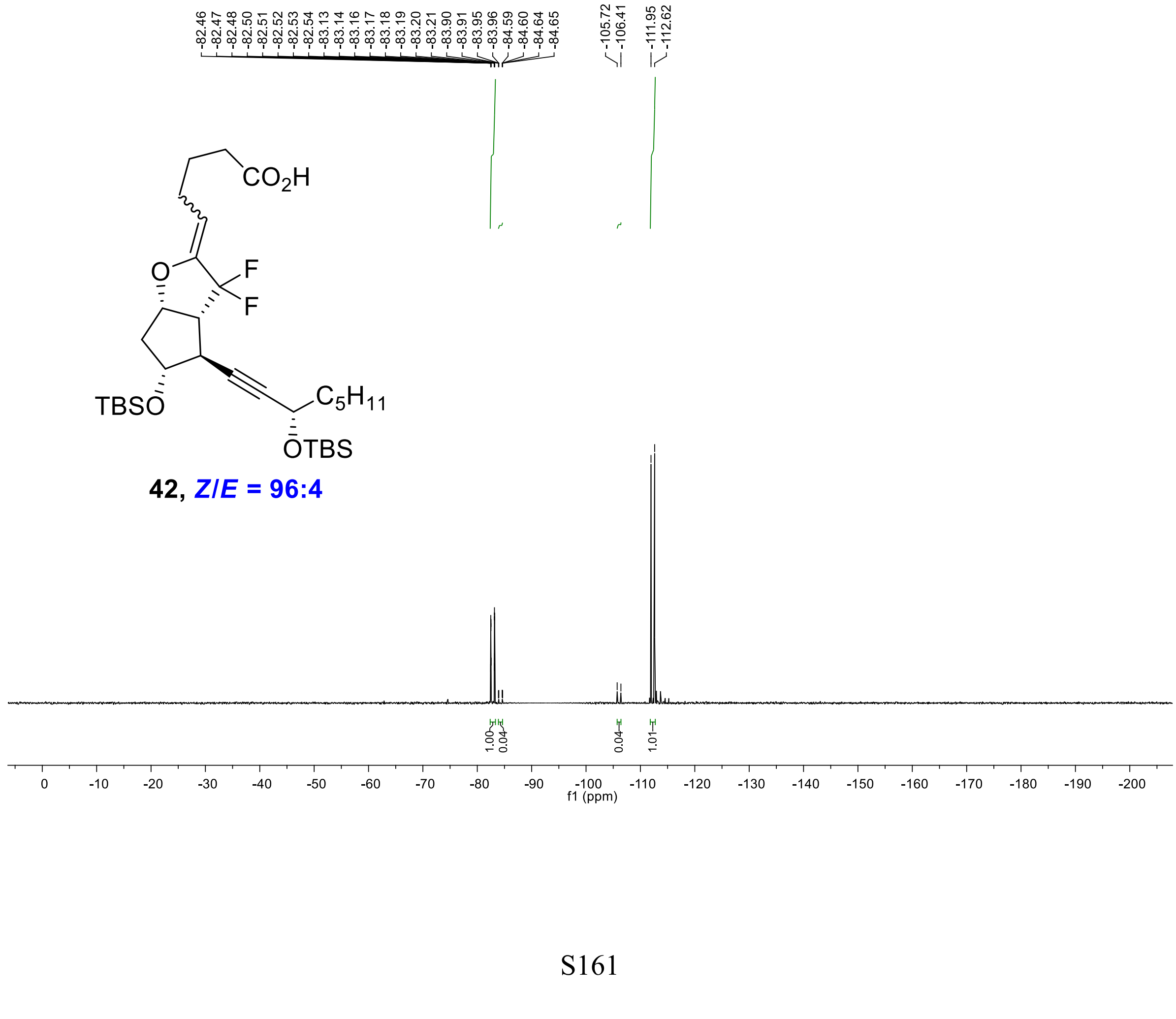

42, ***Z*/*E* = 96:4**

5-((3a*R*,4*S*,5*R*,6a*S*)-4-((*S*)-3-(*tert*-butyldimethylsilyloxy)oct-1-ynyl)-3,3-difluoro-5-hydroxyhexahydro-2*H*-cyclopenta[*b*]furan-2-ylidene)pentanoic acid (42')

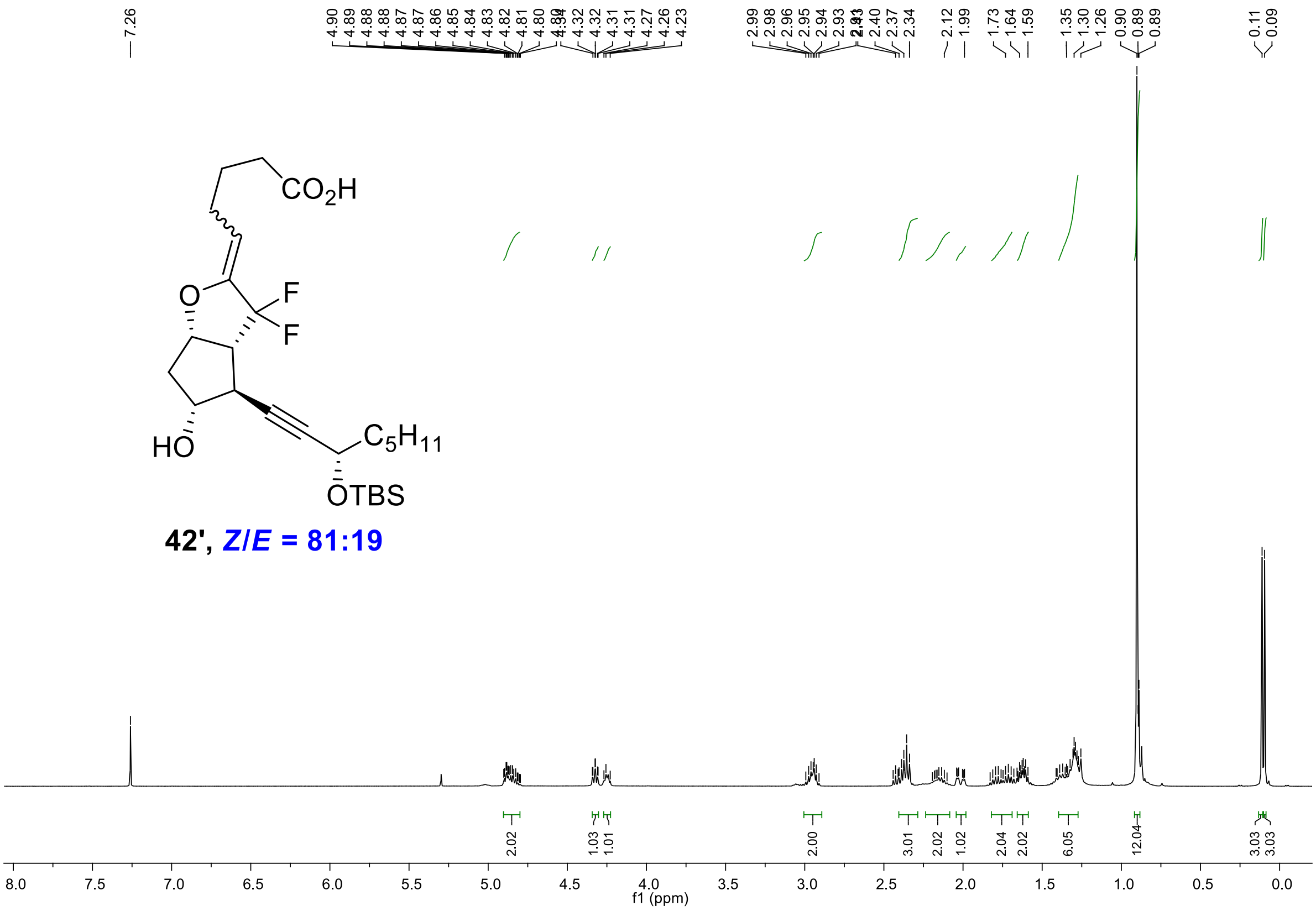

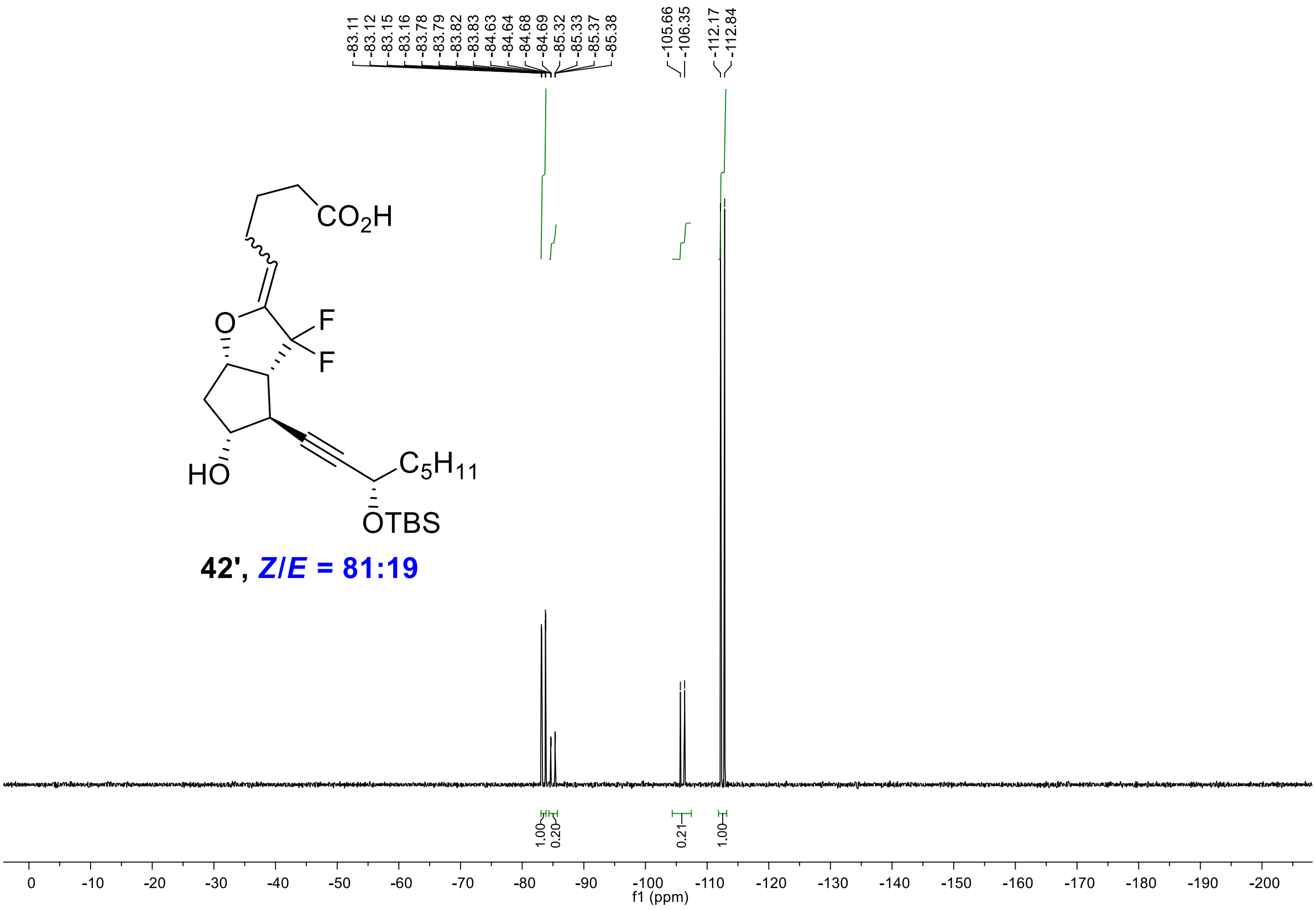

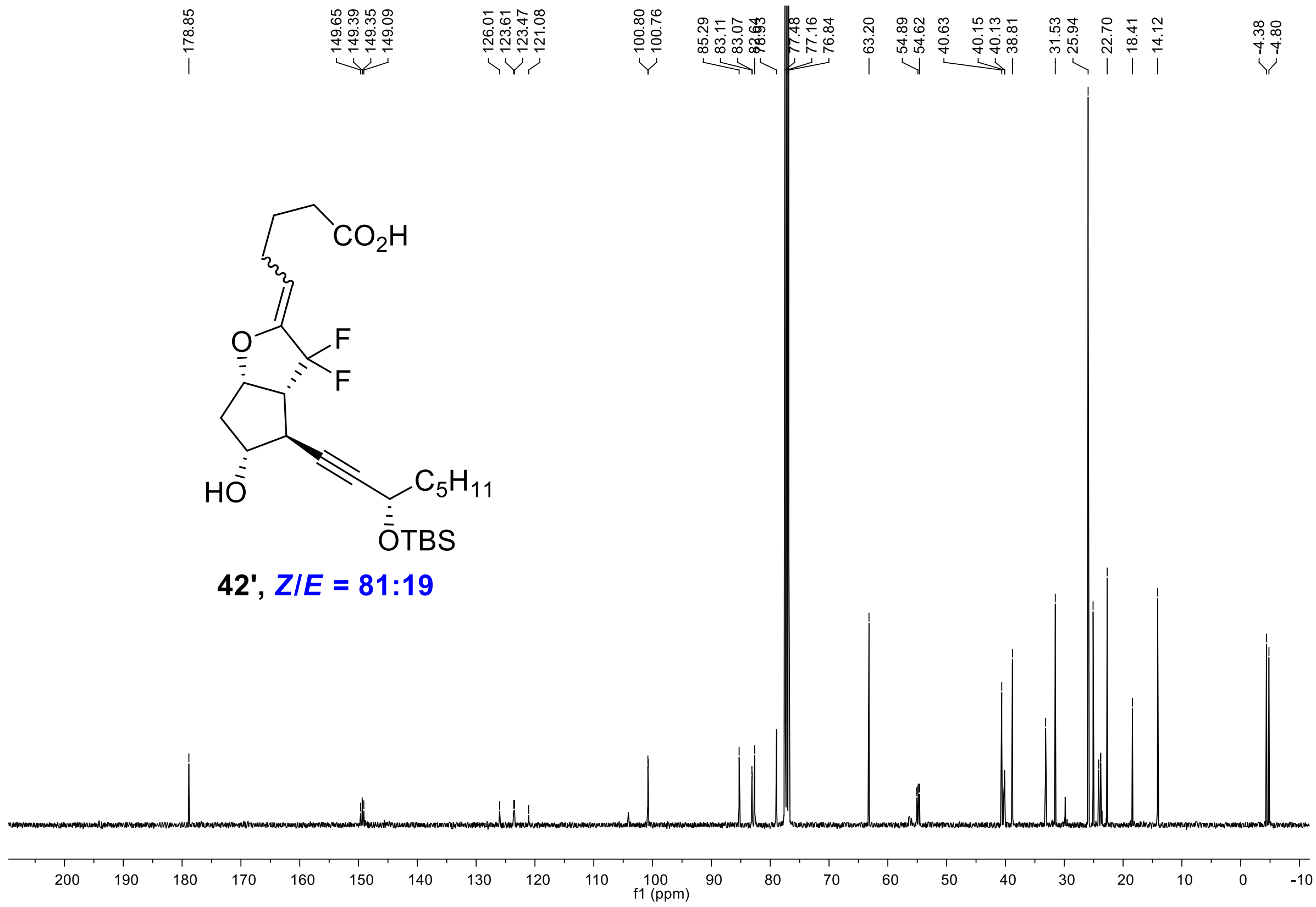

178.85
149.65
149.39
149.35
149.09
126.01
123.61
123.47
121.08
100.80
100.76
85.29
83.11
83.07
77.48
77.16
76.84
63.20
54.89
54.62
40.63
40.15
40.13
38.81
31.53
25.94
22.70
18.41
14.12
-4.38
-4.80
CO_2H
O
F
F
HO
C_5H_{11}
OTBS
42', ***Z*/*E* = 81:19**
200 190 180 170 160 150 140 130 120 110 100 90 80 70 60 50 40 30 20 10 0 -10
f1 (ppm)

5-((3a*R*,4*S*,5*R*,6a*S*)-3,3-Difluoro-5-hydroxy-4-((*S*)-3-hydroxyoct-1-ynyl)hexahydro-2*H*-cyclopenta[*b*]furan-2-ylidene)pentanoic acid (8)

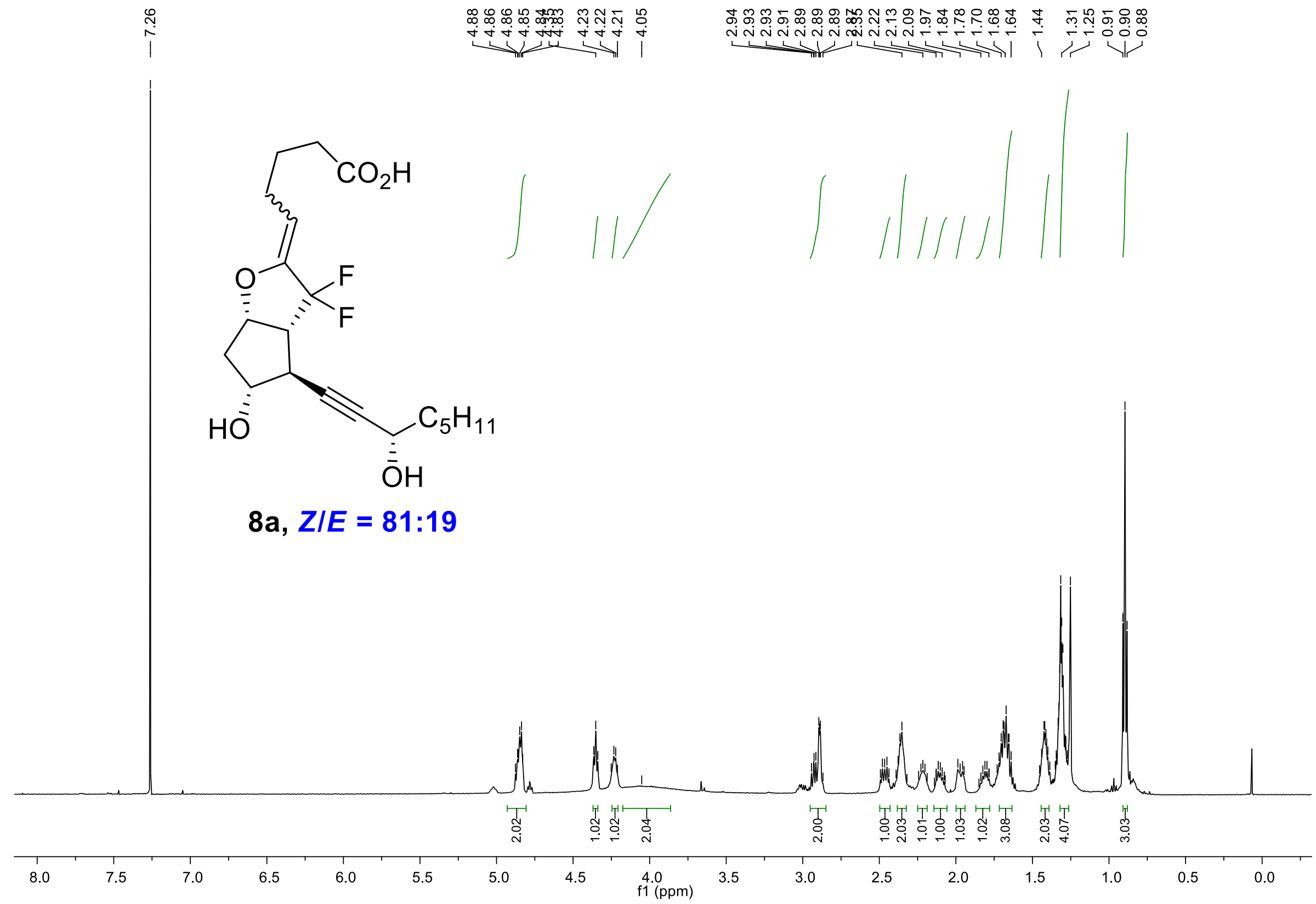

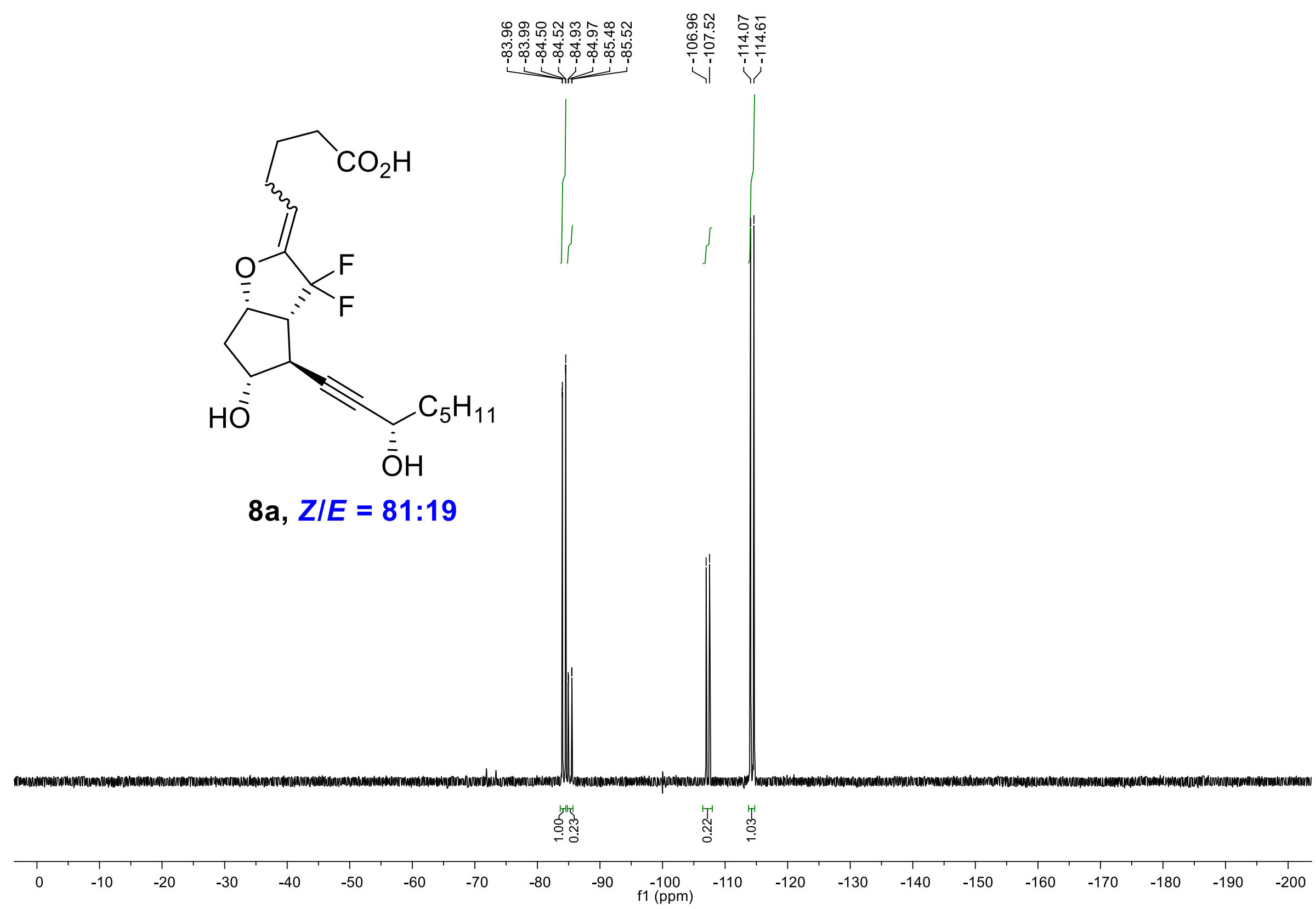

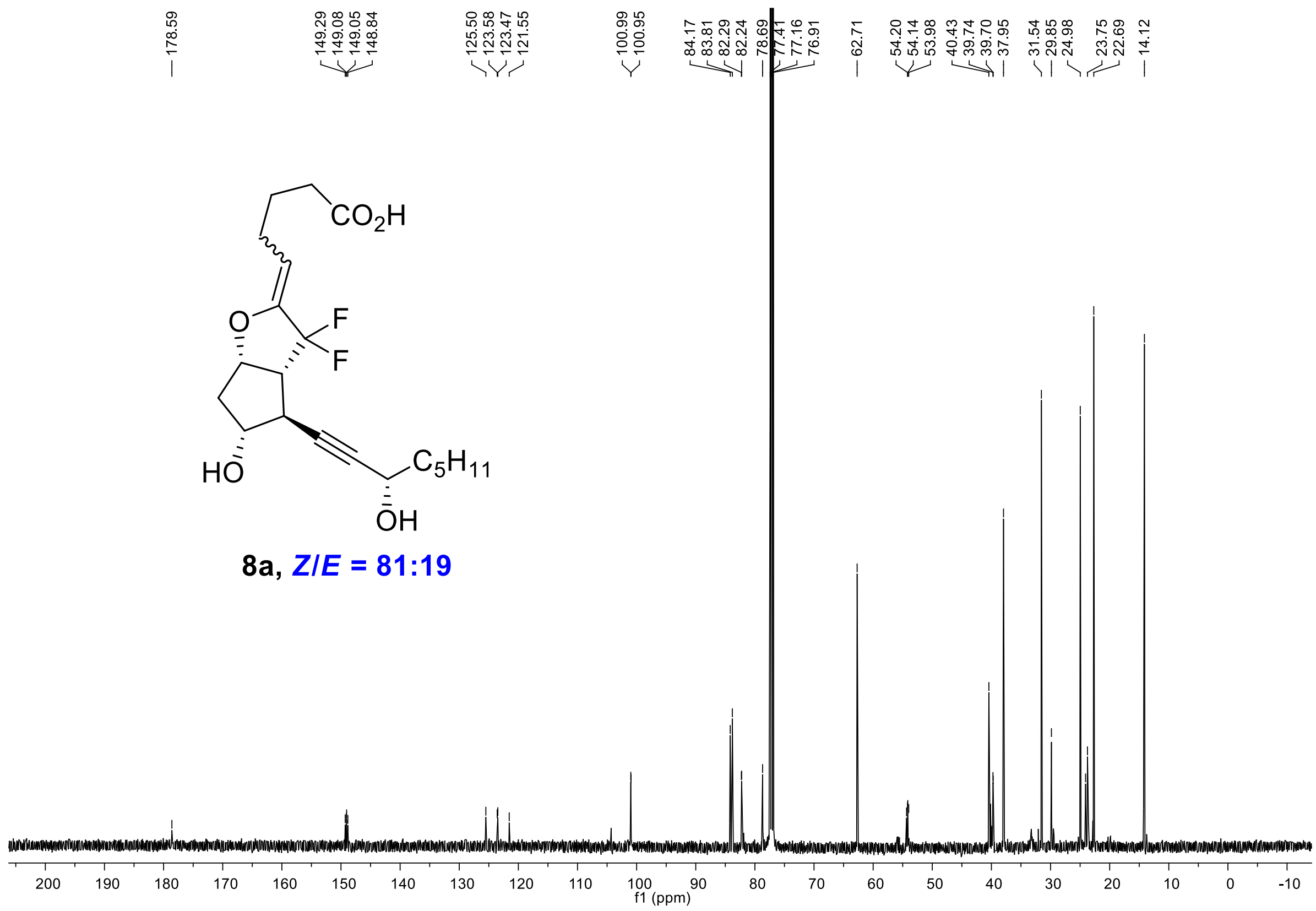
178.59
149.29
149.08
149.05
148.84
125.50
123.58
123.47
121.55
100.99
100.95
84.17
83.81
82.29
82.24
78.69
77.41
77.16
76.91
62.71
54.20
54.14
53.98
40.43
39.74
39.70
37.95
31.54
29.85
24.98
23.75
22.69
14.12
CO2H
O
F
F
HO
C5H11
OH
8a, Z/E = 81:19
f1 (ppm)

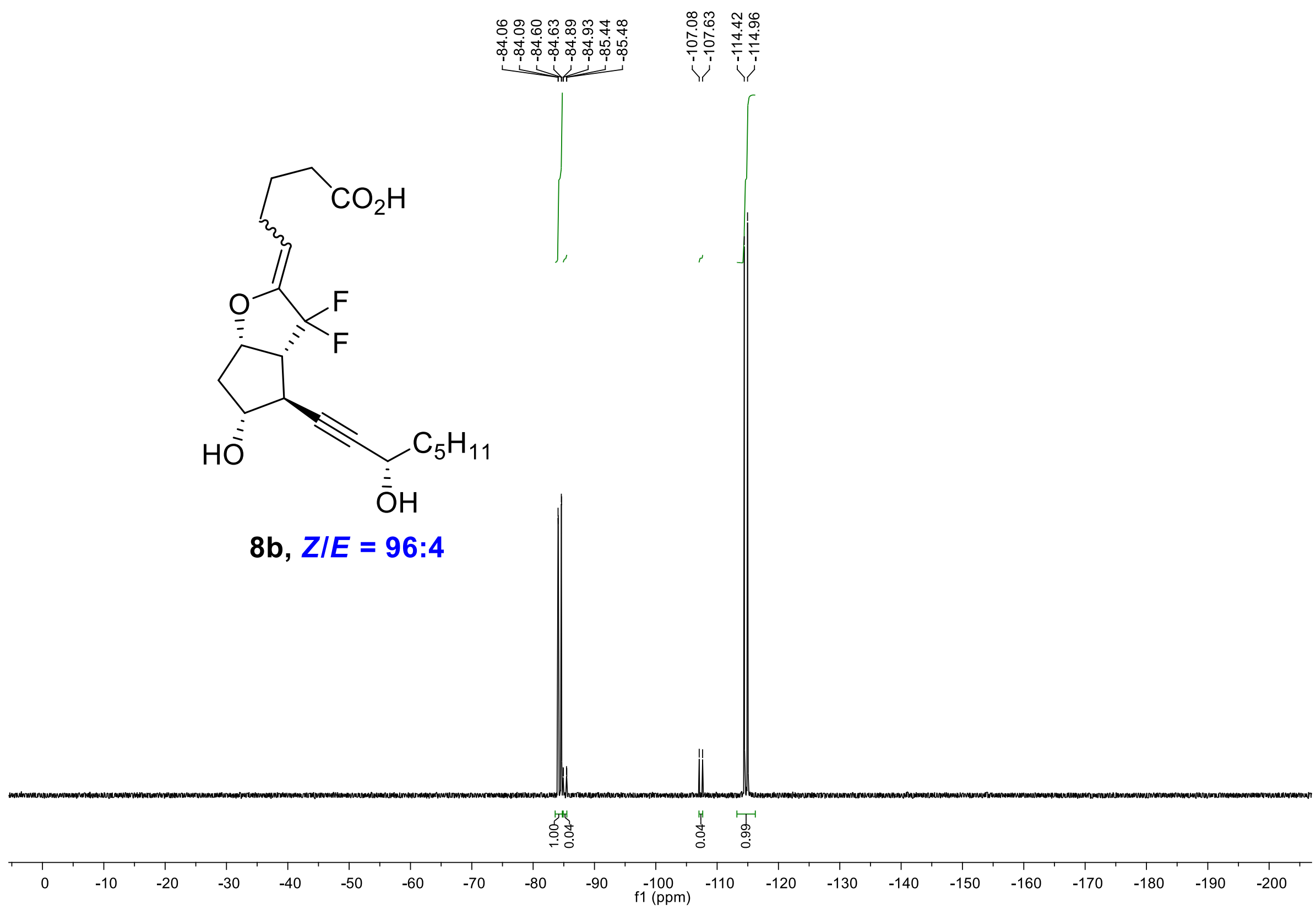
-84.06
-84.09
-84.60
-84.63
-84.89
-84.93
-85.44
-85.48
-107.08
-107.63
-114.42
-114.96
CO2H
O
F
F
HO
C5H11
OH
8b, Z/E = 96:4
1.00
0.04
0.04
0.99
f1 (ppm)

(3a*R*,4*S*,5*R*,6a*S*)-5-(*tert*-Butyldimethylsilyloxy)-4-((3*S*,4*S*)-3-(*tert*-butyldimethylsilyloxy)-4-methylnona-1,6-diynyl)-3,3-difluorohexahydro-2*H*-cyclopenta[*b*]furan-2-one (43)

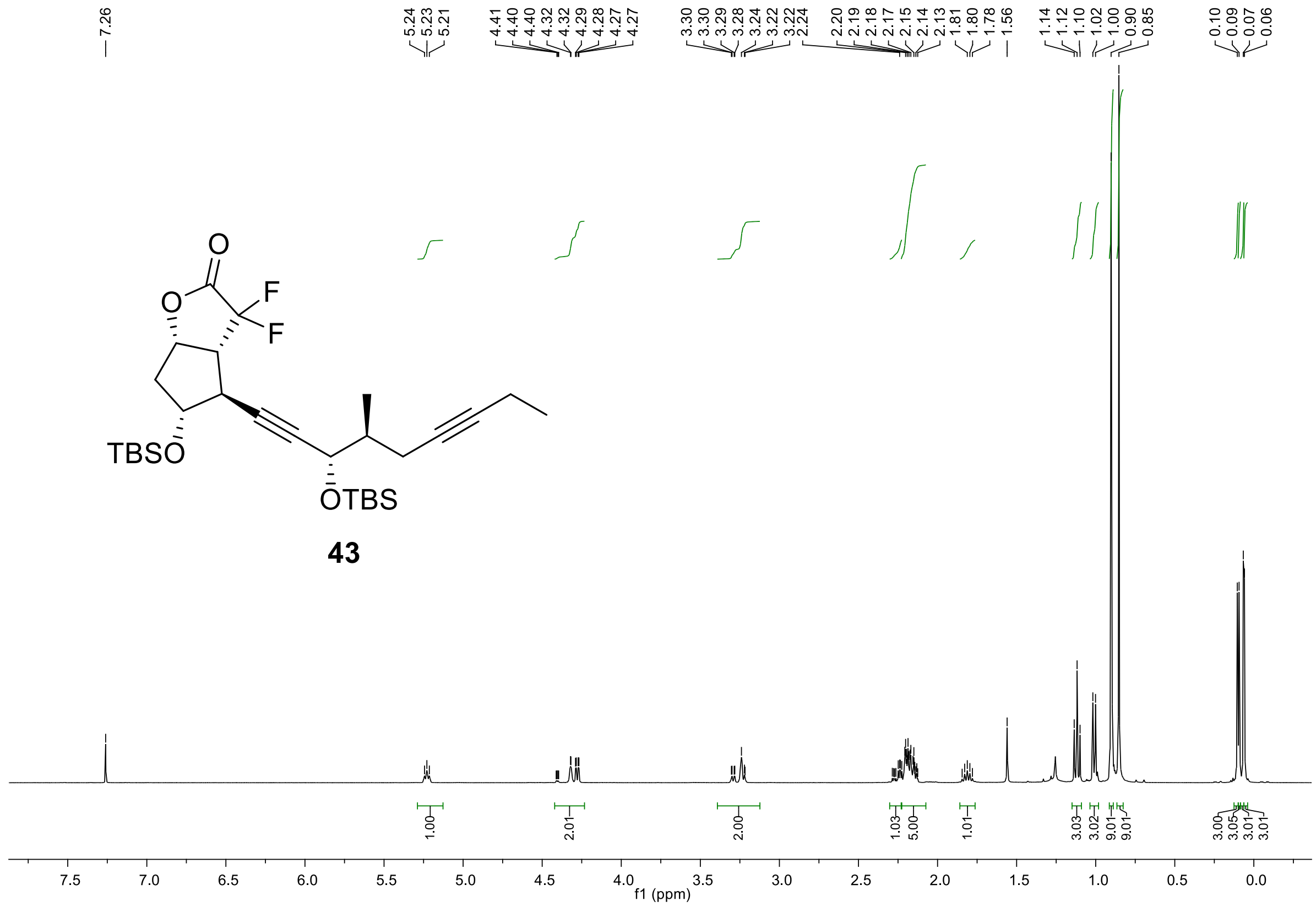

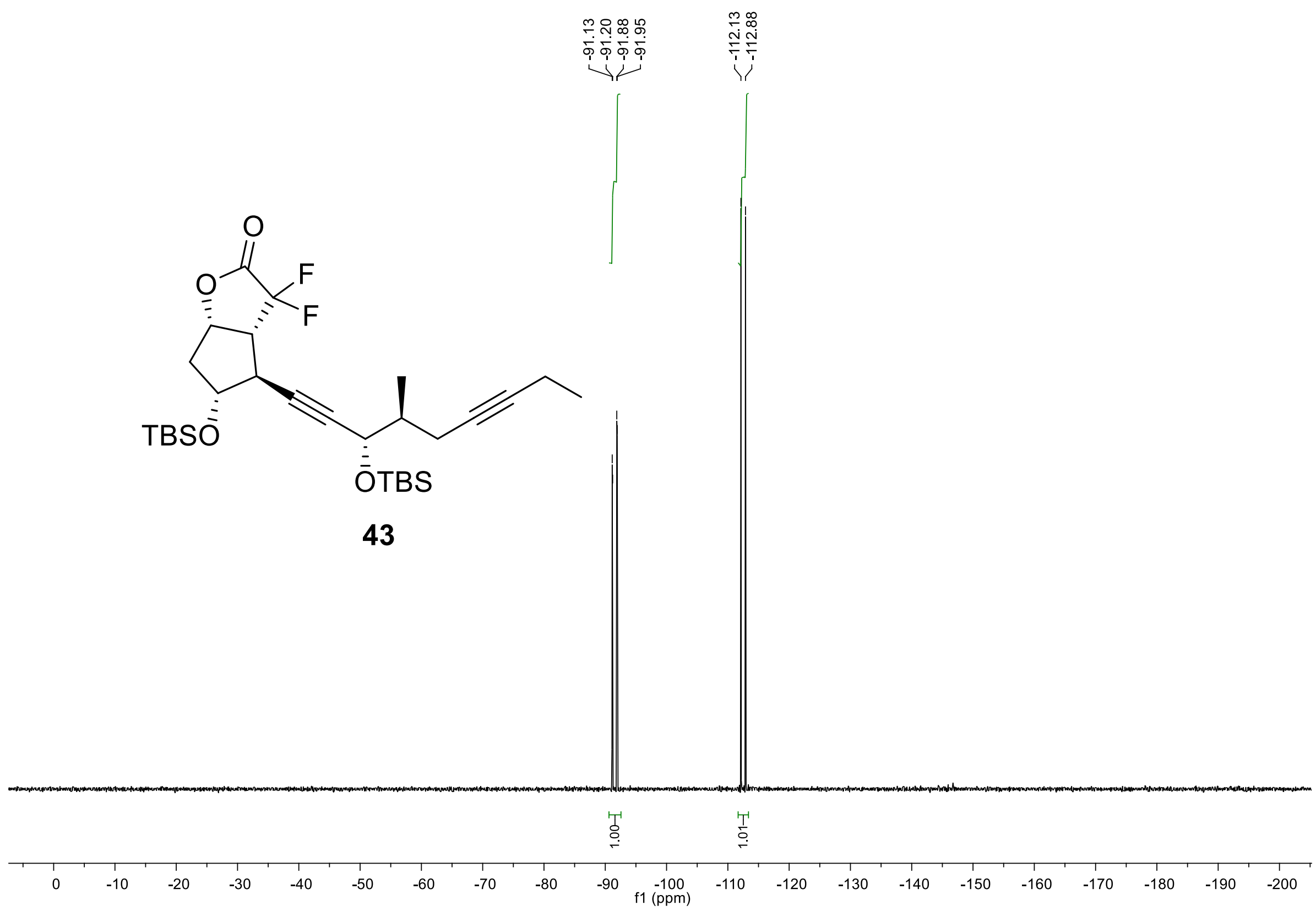

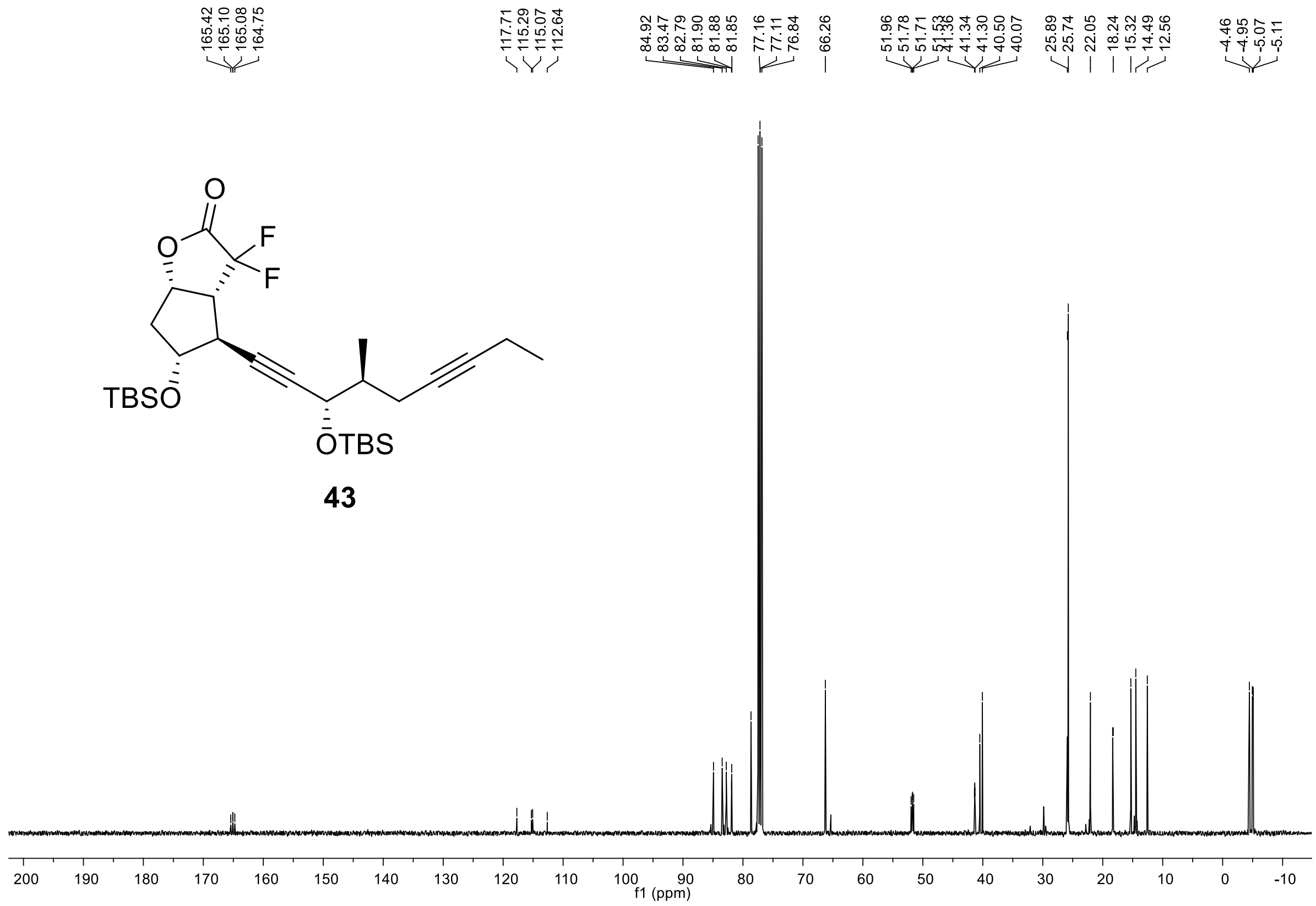
165.42
165.10
165.08
164.75
117.71
115.29
115.07
112.64
84.92
83.47
82.79
81.90
81.88
81.85
77.16
77.11
76.84
66.26
51.96
51.78
51.71
51.53
41.36
41.34
41.30
40.50
40.07
25.89
25.74
22.05
18.24
15.32
14.49
12.56
-4.46
-4.95
-5.07
-5.11
O
O
F
F
TBSO
OTBS
43
200 190 180 170 160 150 140 130 120 110 100 90 80 70 60 50 40 30 20 10 0 -10
f1 (ppm)

5-((3a*R*,4*S*,5*R*,6a*S*)-5-(*tert*-Butyldimethylsilyloxy)-4-((3*S*,4*S*)-3-(*tert*-butyldimethylsilyloxy)-4-methylnona-1,6-diynyl)-3,3-difluorohexahydro-2*H*-cyclopenta[*b*]furan-2-ylidene)pentanoic acid (44)

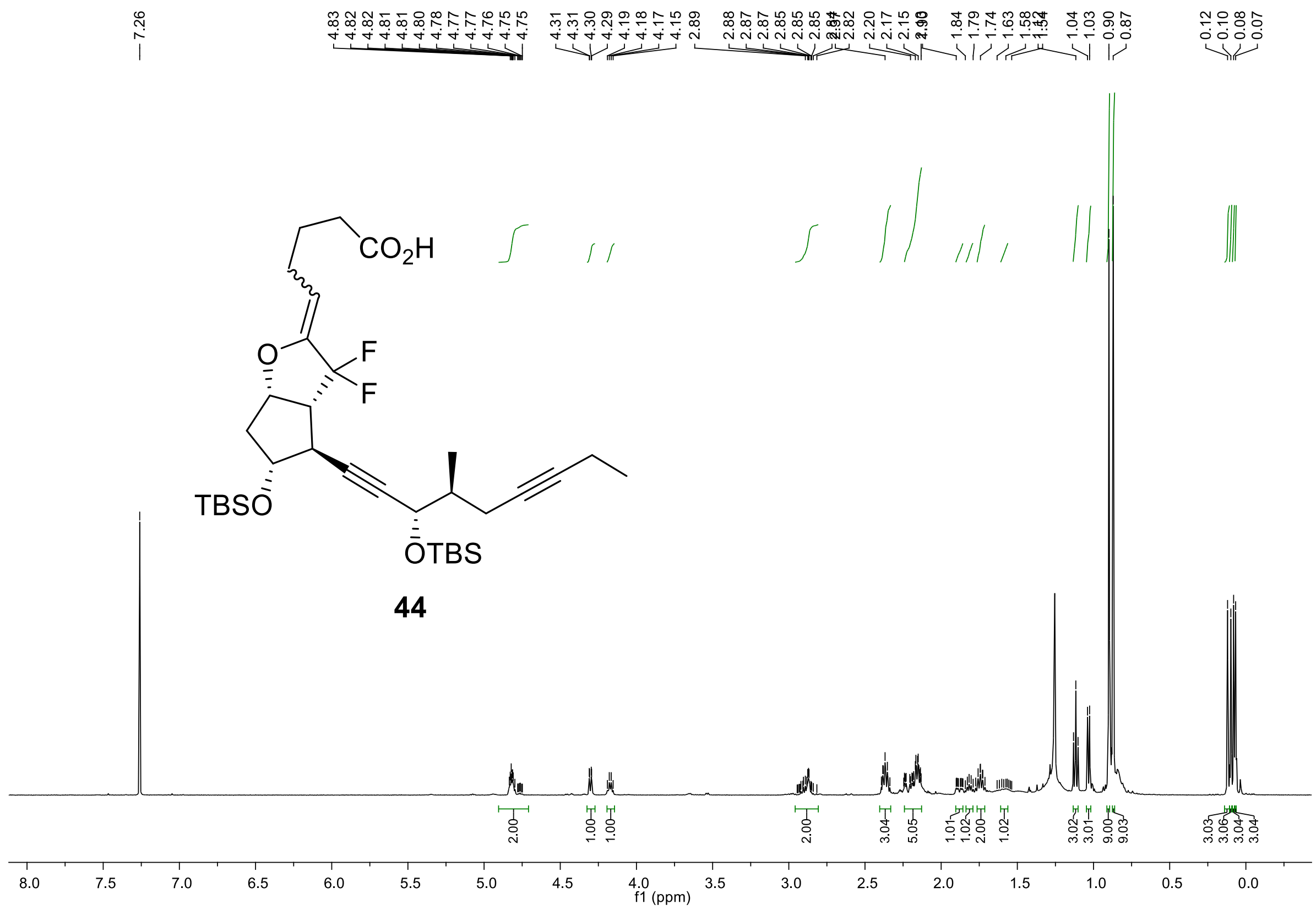

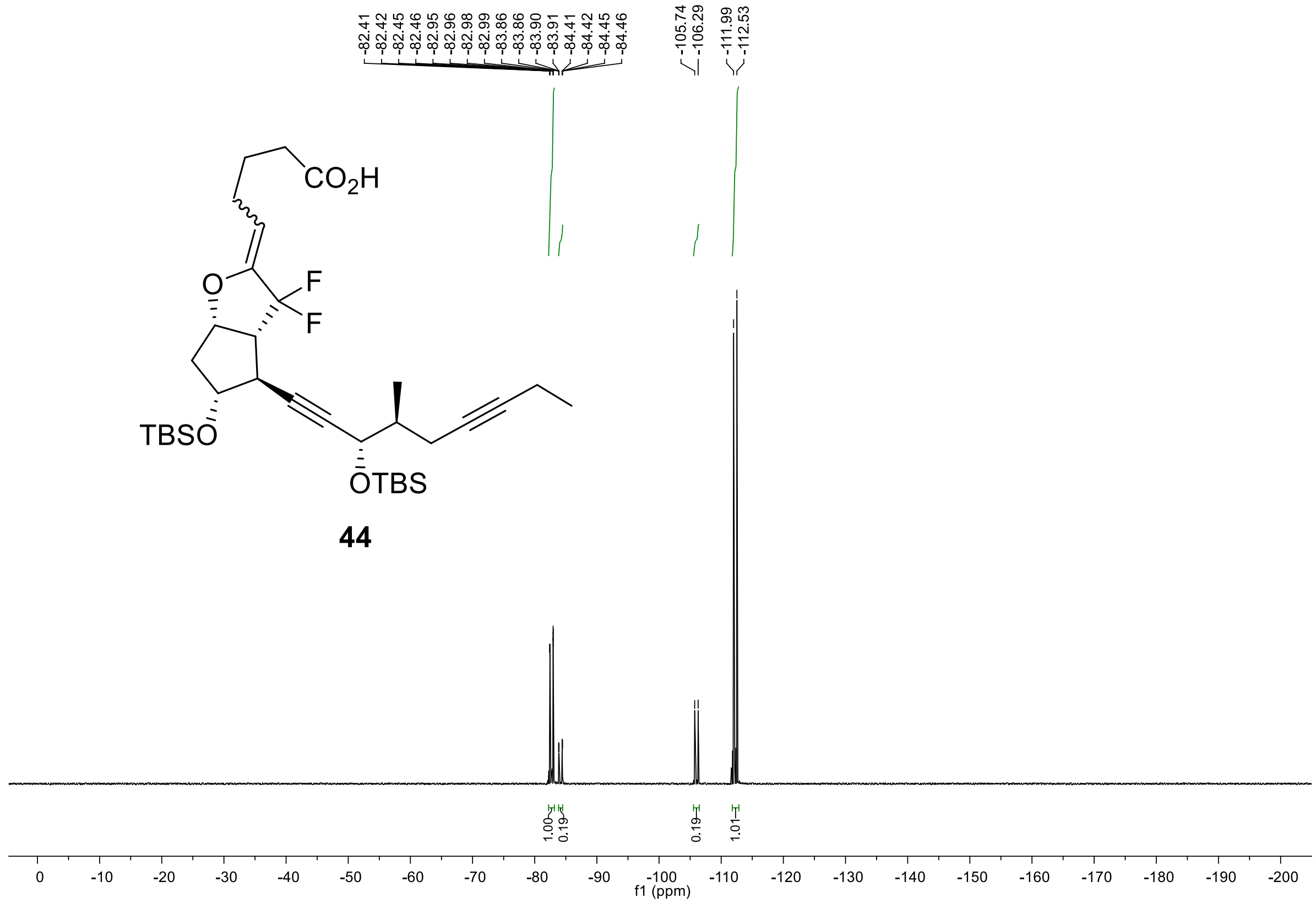

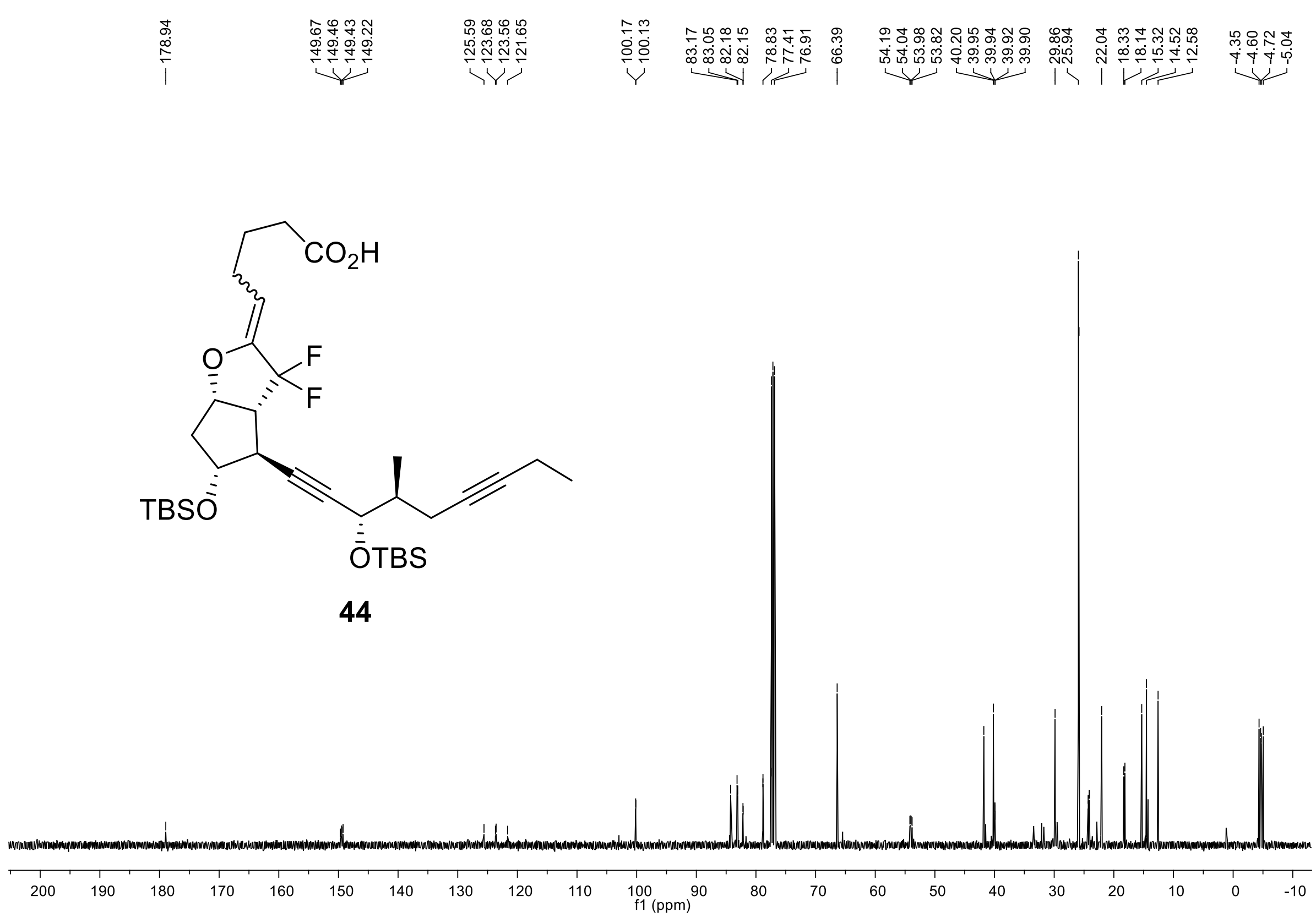
178.94
149.67
149.46
149.43
149.22
125.59
123.68
123.56
121.65
100.17
100.13
83.17
83.05
82.18
82.15
78.83
77.41
76.91
66.39
54.19
54.04
53.98
53.82
40.20
39.95
39.94
39.92
39.90
29.86
25.94
22.04
18.33
18.14
15.32
14.52
12.58
-4.35
-4.60
-4.72
-5.04
CO_2H
O
F
F
TBSO
OTBS
44
f1 (ppm)

5-((3a*R*,4*S*,5*R*,6a*S*)-3,3-Difluoro-5-hydroxy-4-((3*S*,4*S*)-3-hydroxy-4-methylnona-1,6-diynyl)hexahydro-2*H*-cyclopenta[*b*]furan-2-ylidene)pentanoic acid (9)

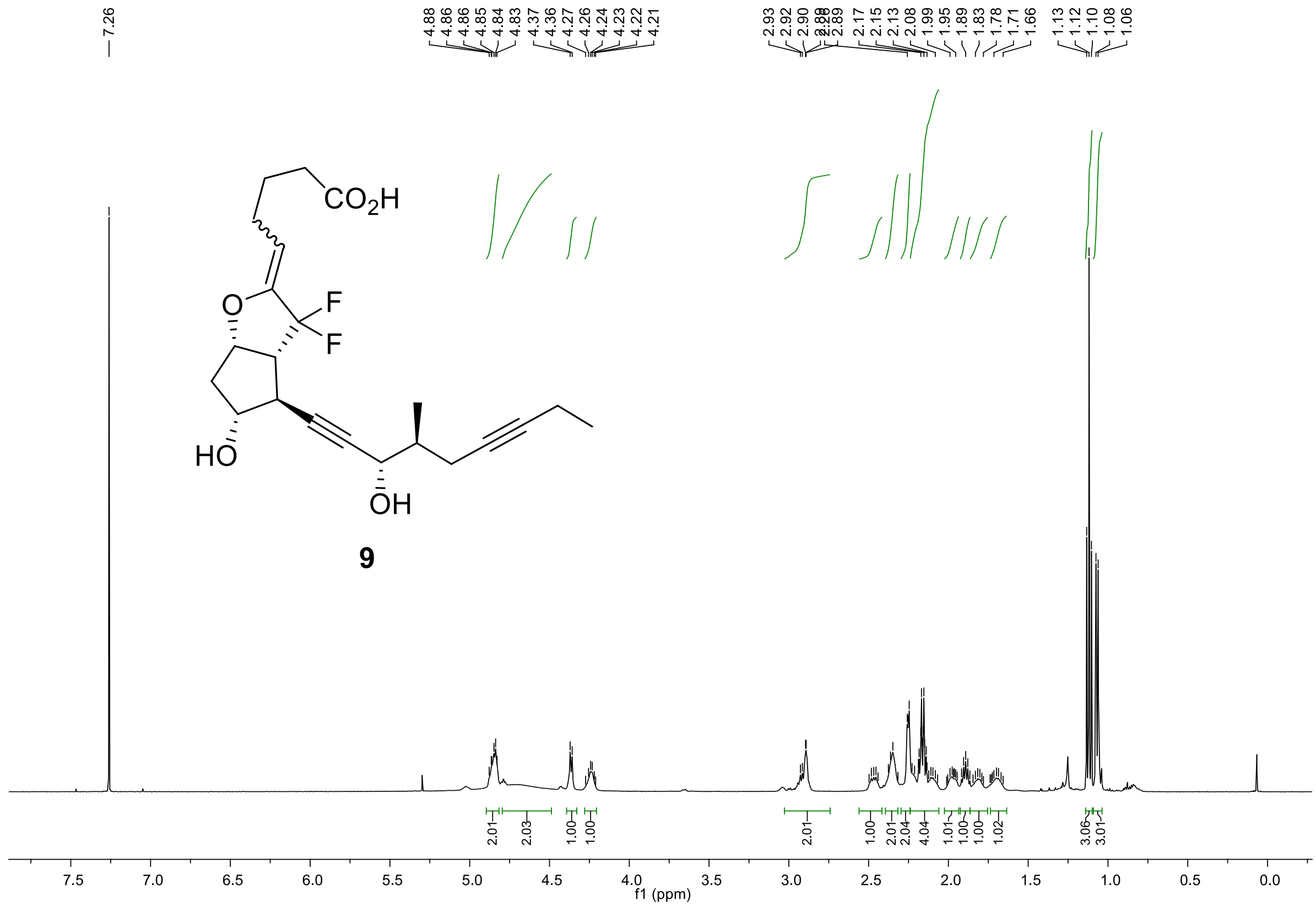

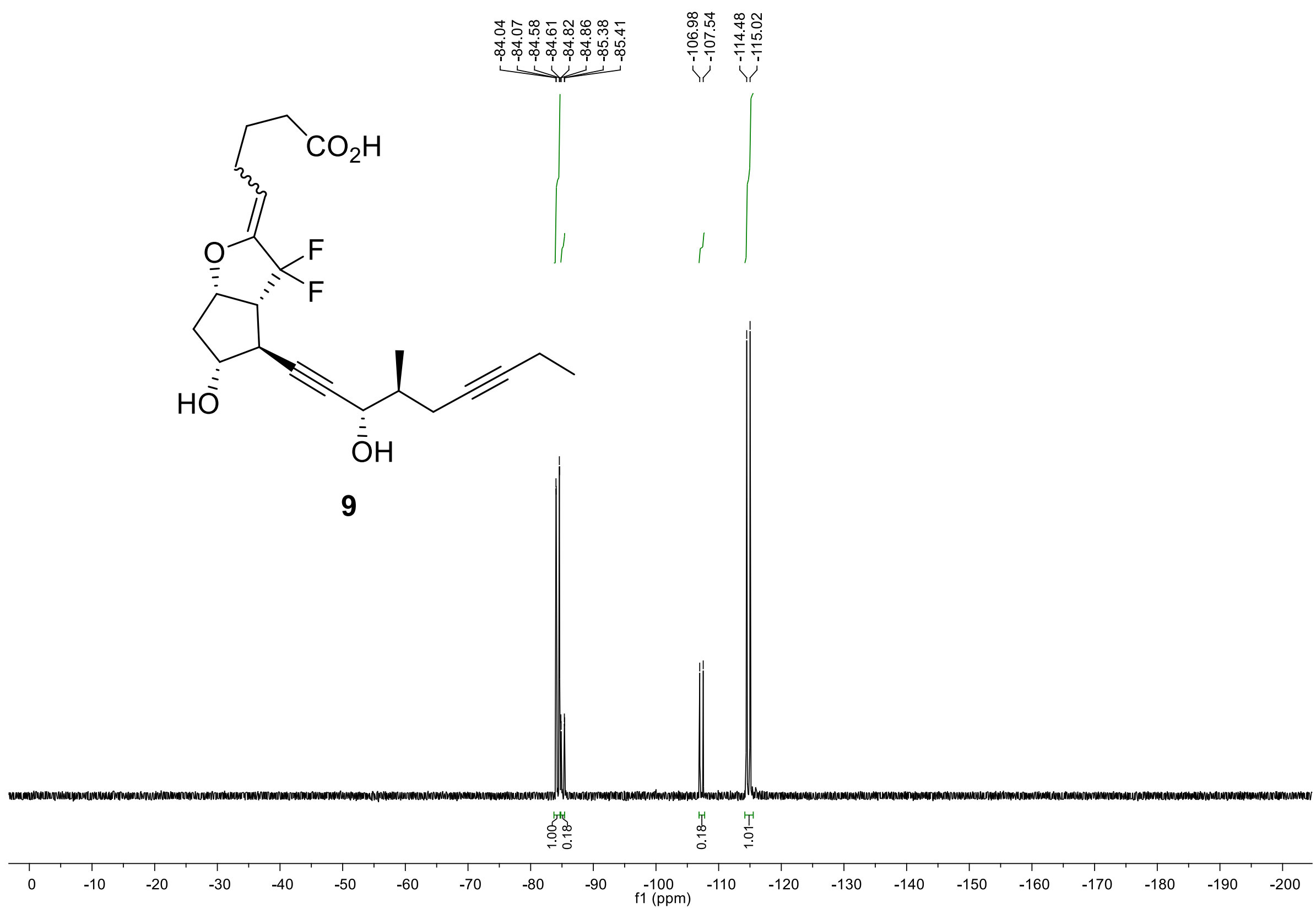

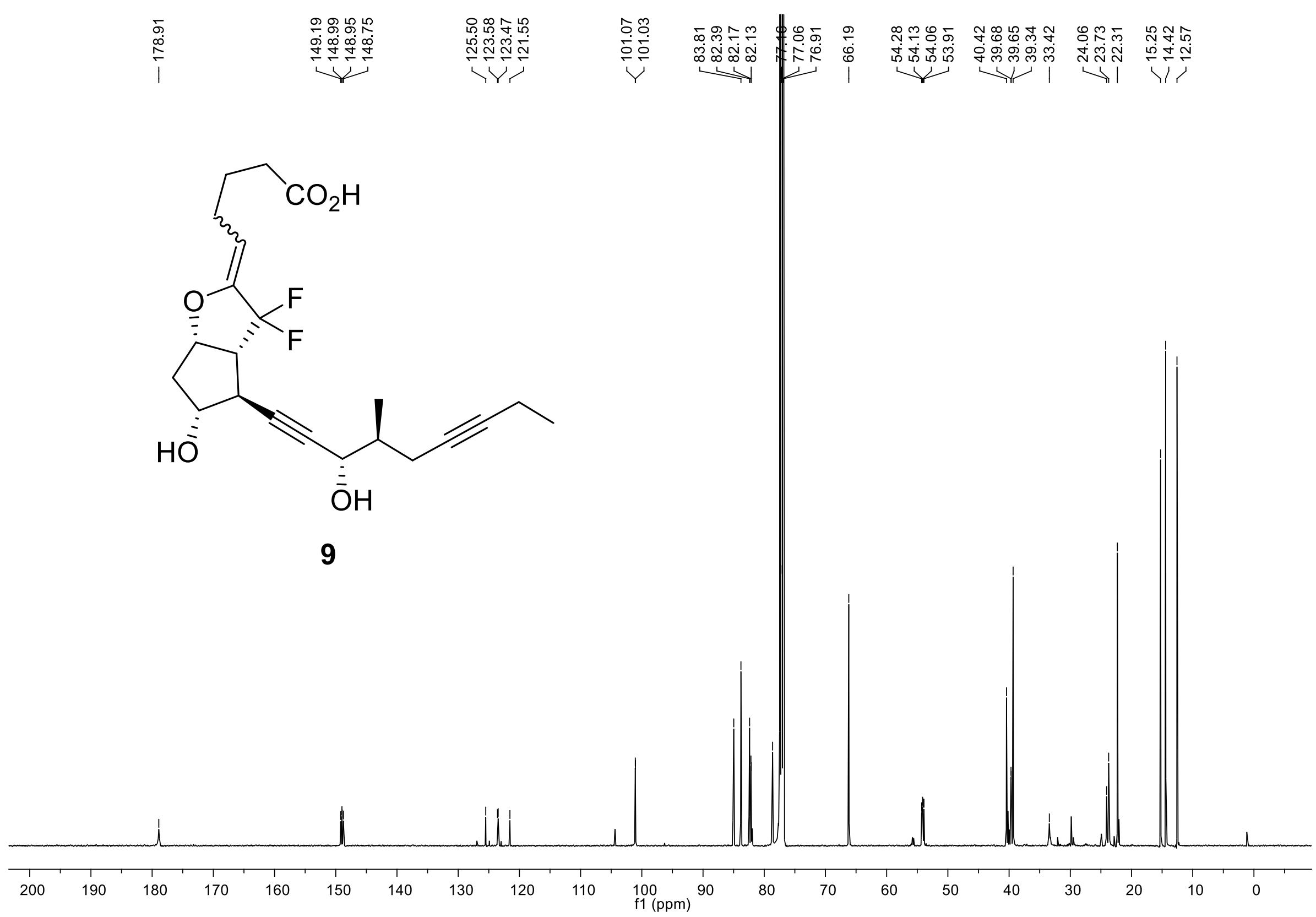
178.91
149.19
148.99
148.95
148.75
125.50
123.58
123.47
121.55
101.07
101.03
83.81
82.39
82.17
82.13
77.06
76.91
66.19
54.28
54.13
54.06
53.91
40.42
39.68
39.65
39.34
33.42
24.06
23.73
22.31
15.25
14.42
12.57
CO2H
O
F
F
HO
OH
9
f1 (ppm)

(3*S*,3a*R*,4*S*,5*R*,6a*S*)-5-(*tert*-Butyldimethylsilyloxy)-4-((*S*)-3-(*tert*-butyldimethylsilyloxy)oct-1-ynyl)-3-fluorohexahydro-2*H*-cyclopenta[*b*]furan-2-one (52)

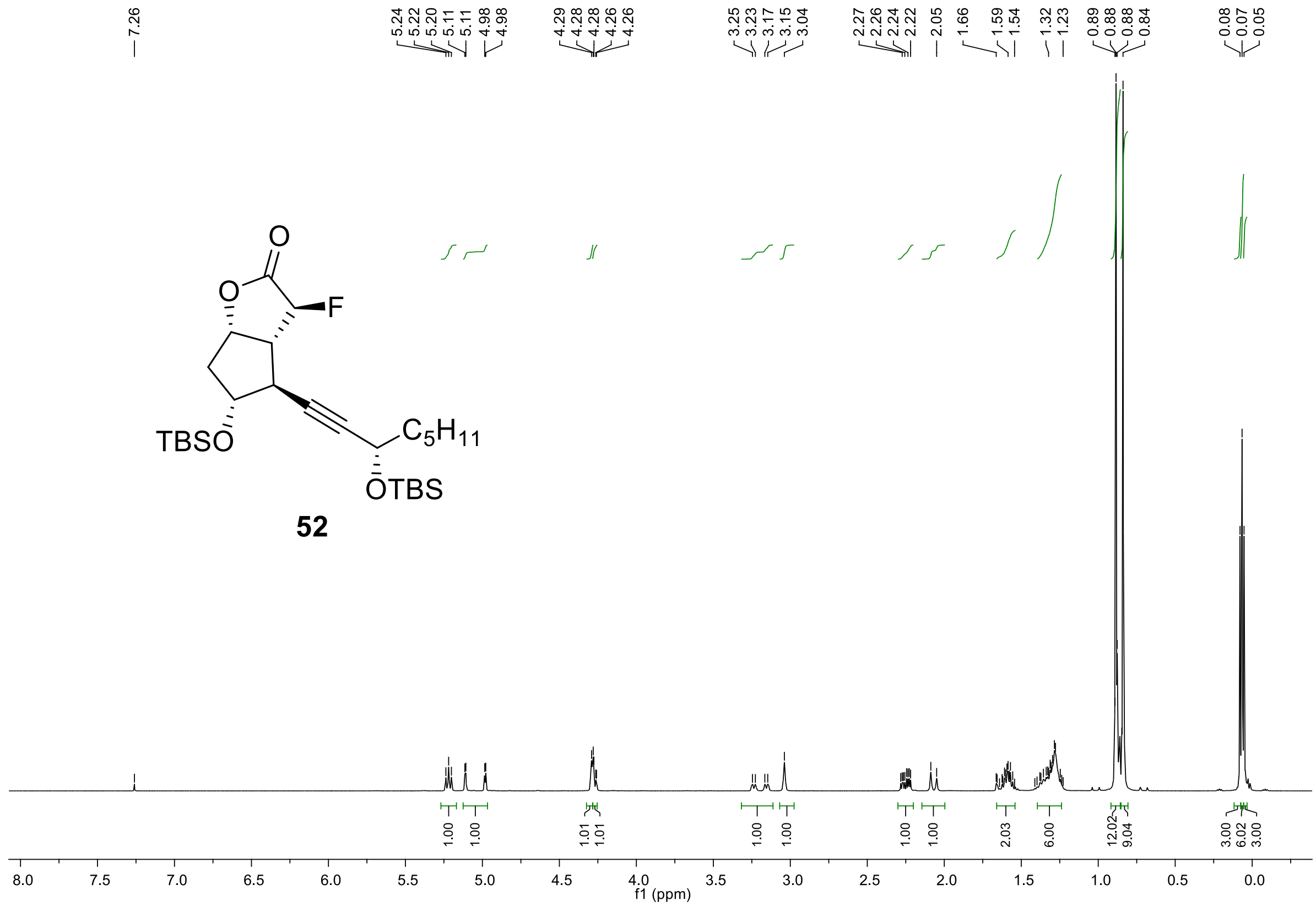

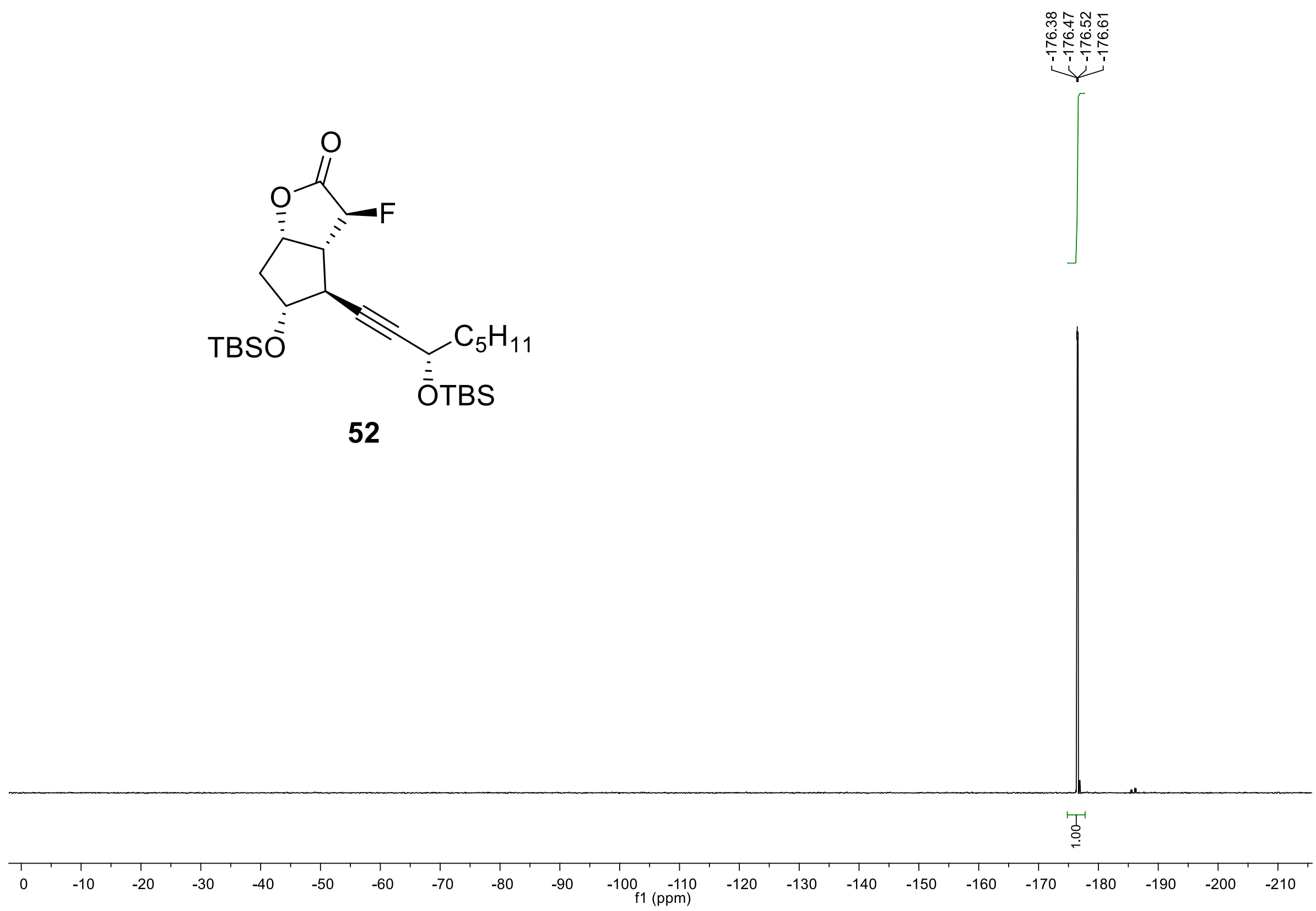

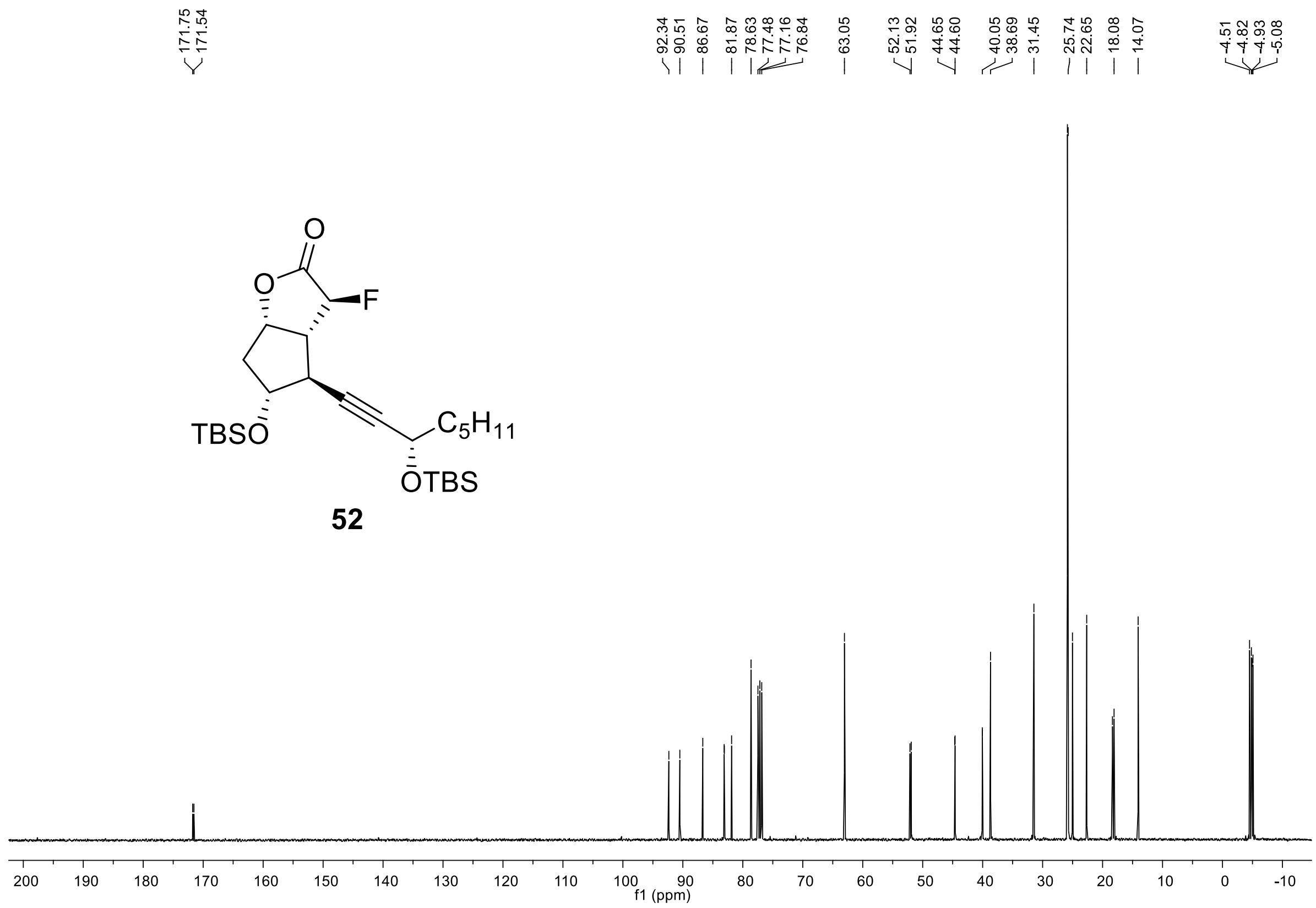

171.75
171.54
92.34
90.51
86.67
81.87
78.63
77.48
77.16
76.84
63.05
52.13
51.92
44.65
44.60
40.05
38.69
31.45
25.74
22.65
18.08
14.07
-4.51
-4.82
-4.93
-5.08
O
O
F
TBSO
C_5H_{11}
OTBS
52
200 190 180 170 160 150 140 130 120 110 100 90 80 70 60 50 40 30 20 10 0 -10
f1 (ppm)

5-((3*S*,3a*R*,4*S*,5*R*,6a*S*)-5-(*tert*-Butyldimethylsilyloxy)-4-((*S*)-3-(*tert*-butyldimethylsilyloxy)oct-1-ynyl)-3-fluorohexahydro-2*H*-cyclopenta[*b*]furan-2-ylidene)pentanoic acid (53)

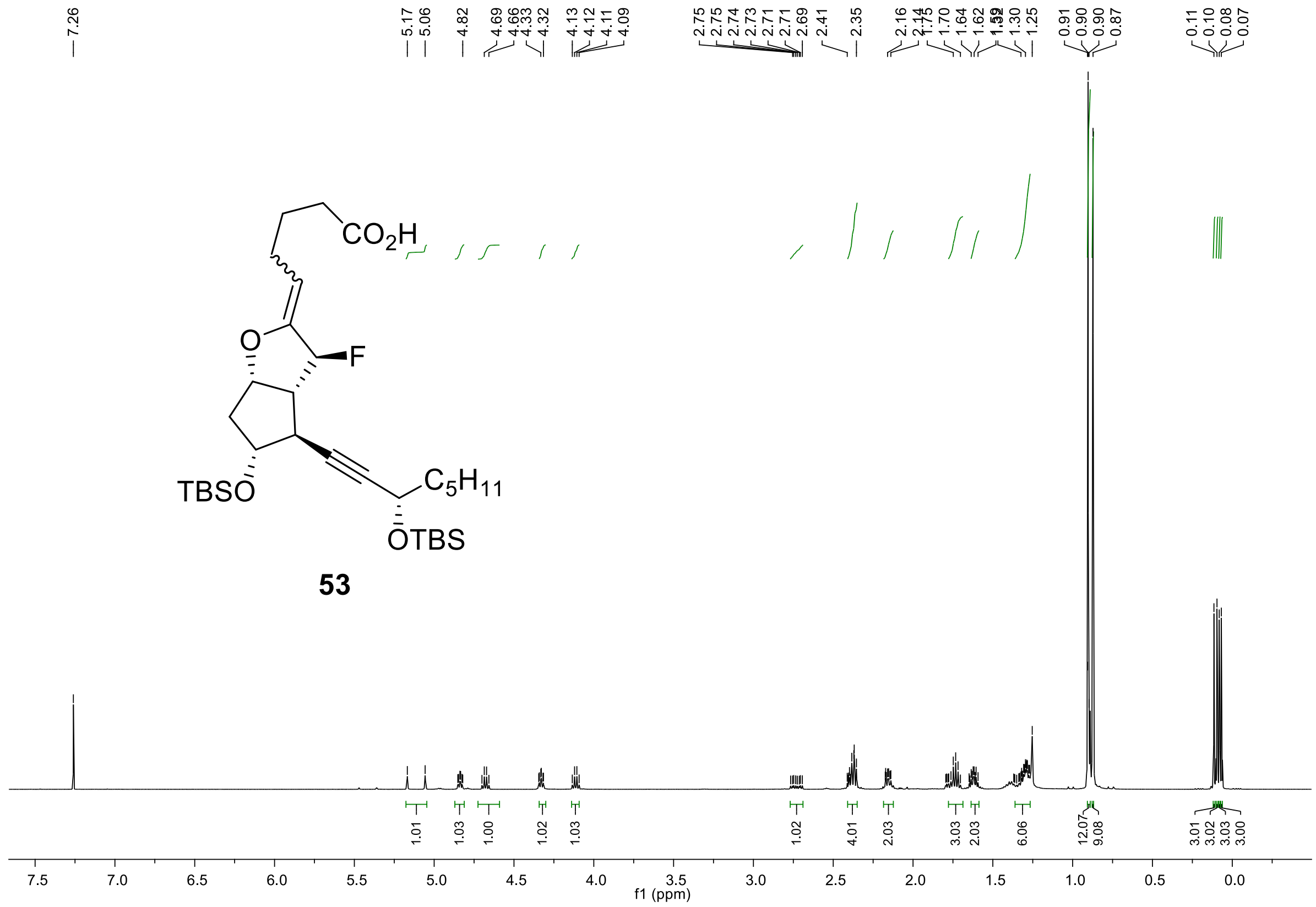

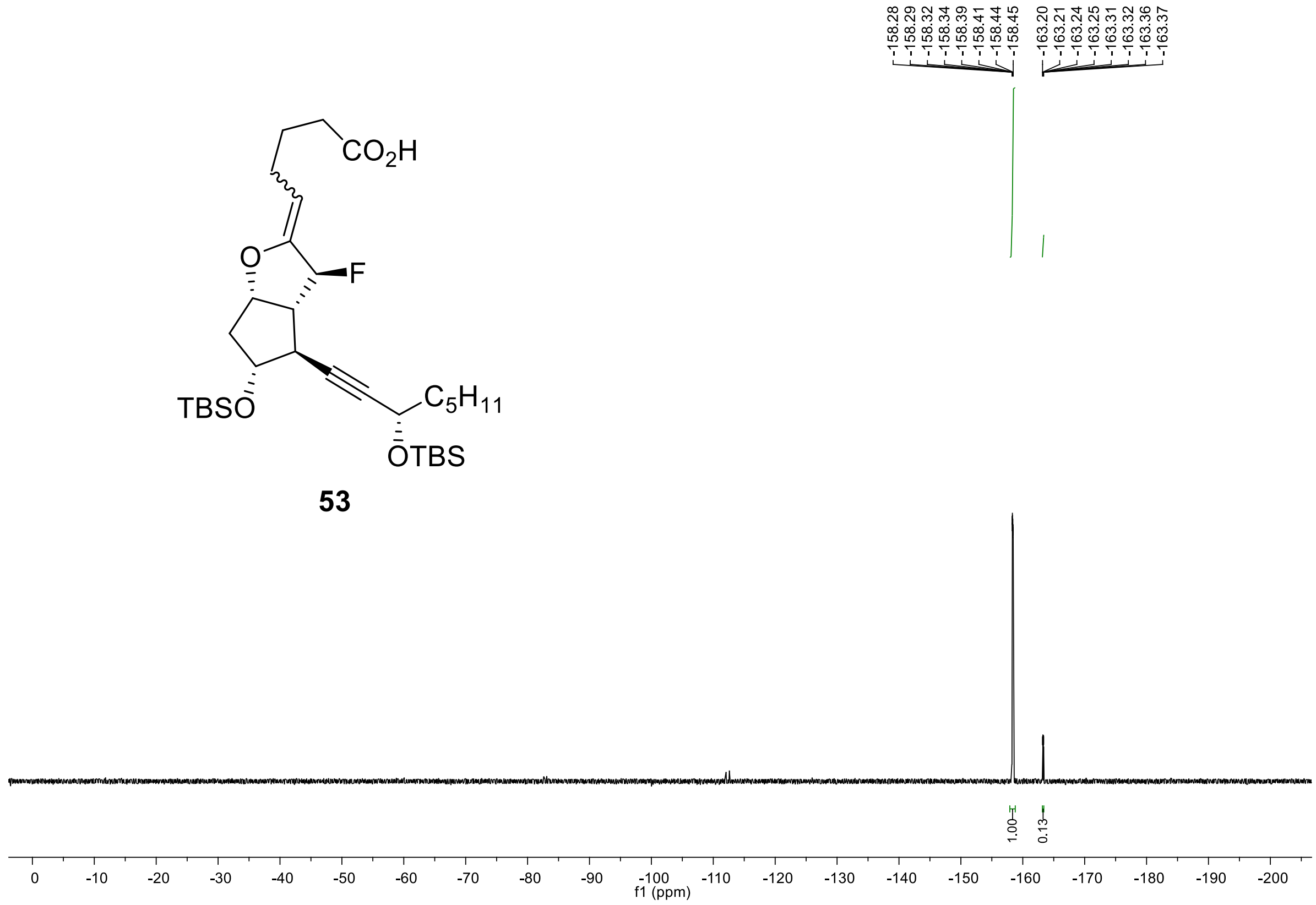

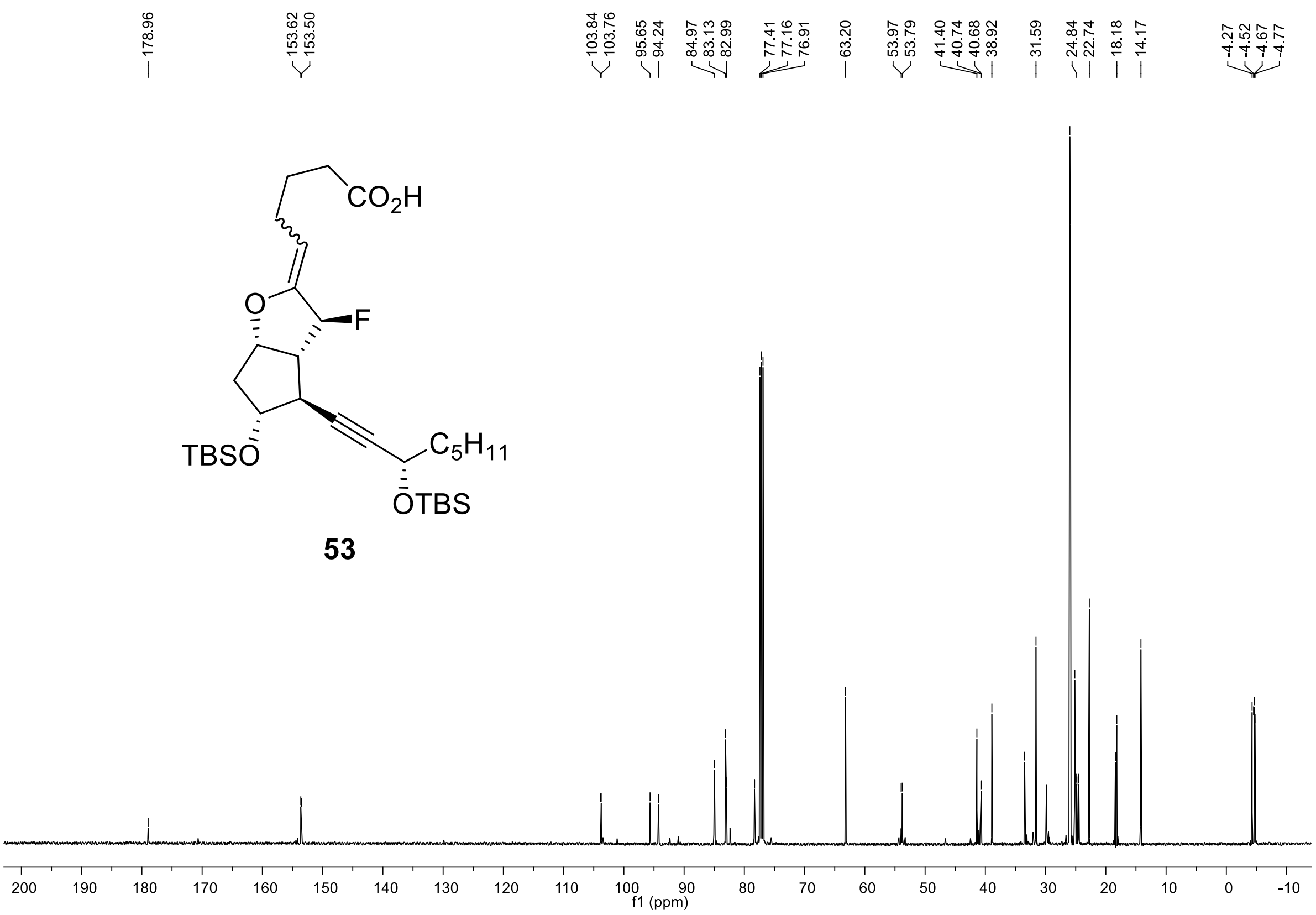
53
178.96
153.62
153.50
103.84
103.76
95.65
94.24
84.97
83.13
82.99
77.41
77.16
76.91
63.20
53.97
53.79
41.40
40.74
40.68
38.92
31.59
24.84
22.74
18.18
14.17
-4.27
-4.52
-4.67
-4.77
200
190
180
170
160
150
140
130
120
110
100
90
80
70
60
50
40
30
20
10
0
-10
f1 (ppm)

5-((3*S*,3a*R*,4*S*,5*R*,6a*S*)-3-fluoro-5-hydroxy-4-((*S*)-3-hydroxyoct-1-ynyl)hexahydro-2*H*-cyclopenta[*b*]furan-2-ylidene)pentanoic acid (4)

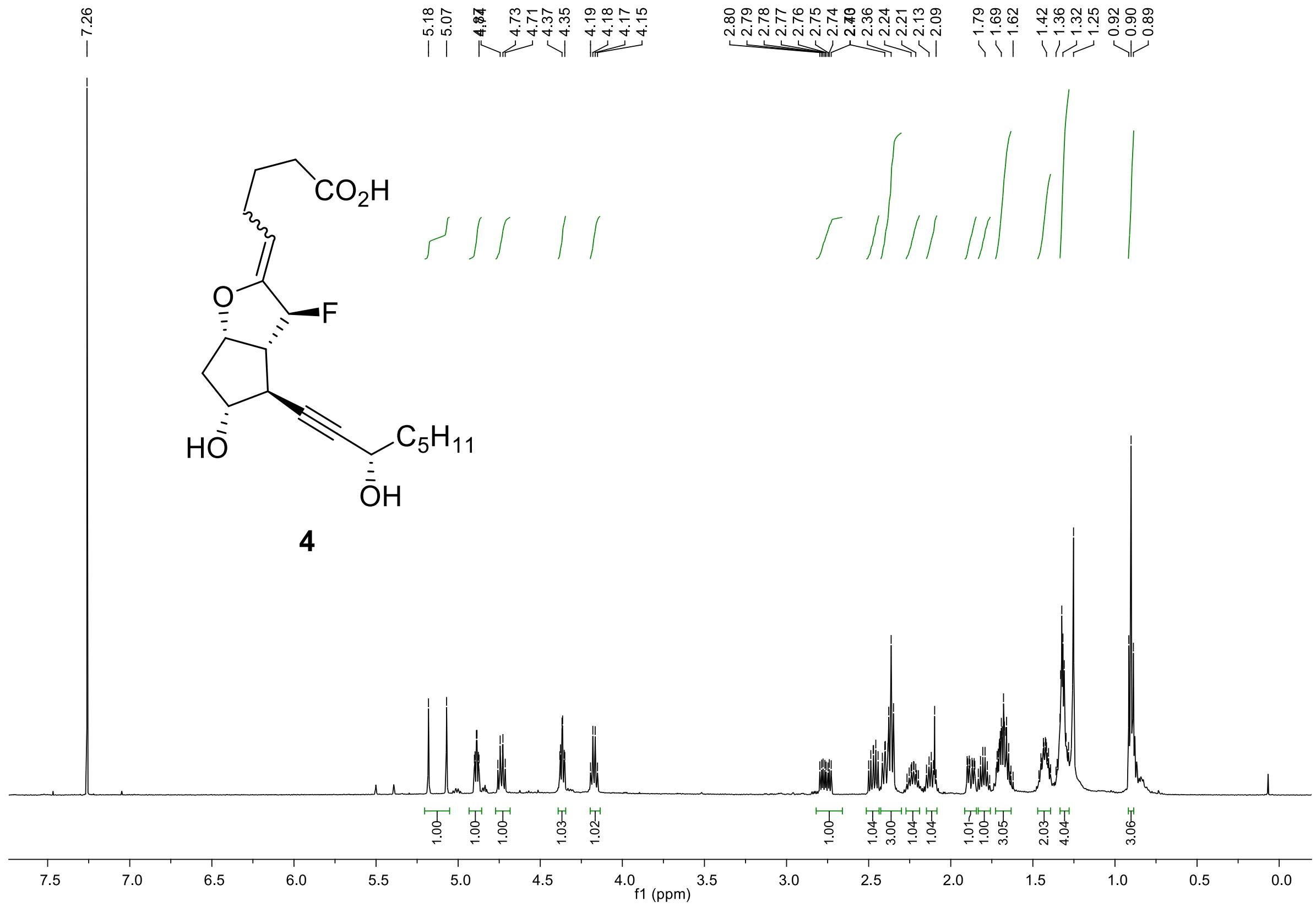

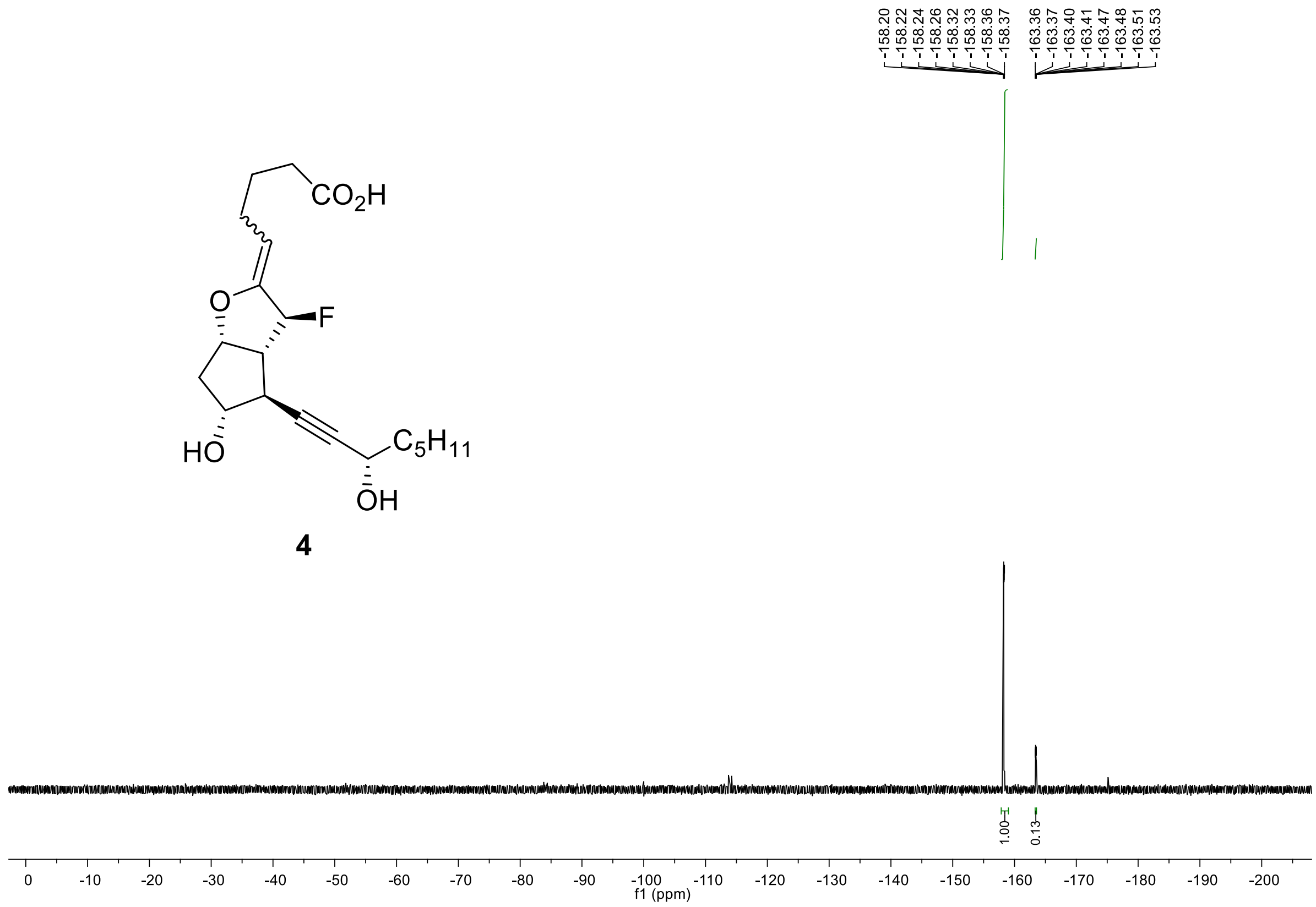

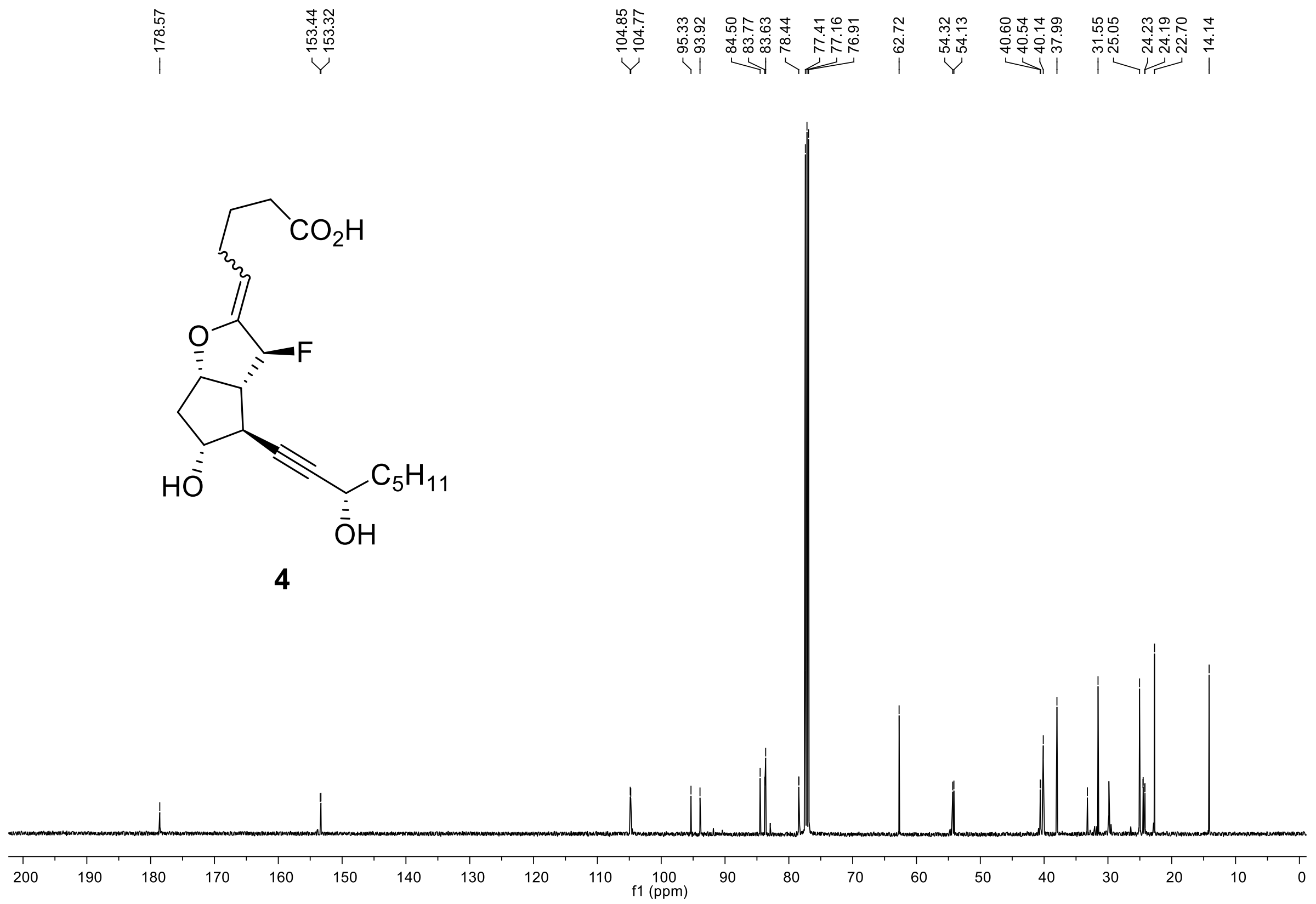
178.57
153.44
153.32
104.85
104.77
95.33
93.92
84.50
83.77
83.63
78.44
77.41
77.16
76.91
62.72
54.32
54.13
40.60
40.54
40.14
37.99
31.55
25.05
24.23
24.19
22.70
14.14
CO2H
O
F
HO
C5H11
OH
4
200 190 180 170 160 150 140 130 120 110 100 90 80 70 60 50 40 30 20 10 0
f1 (ppm)

(3*S*,3a*R*,4*S*,5*R*,6a*S*)-5-(*tert*-Butyldimethylsilyloxy)-4-((3*S*,4*S*)-3-(*tert*-butyldimethylsilyloxy)-4-methylnona-1,6-diynyl)-3-fluorohexahydro-2*H*-cyclopenta[*b*]furan-2-one (54)

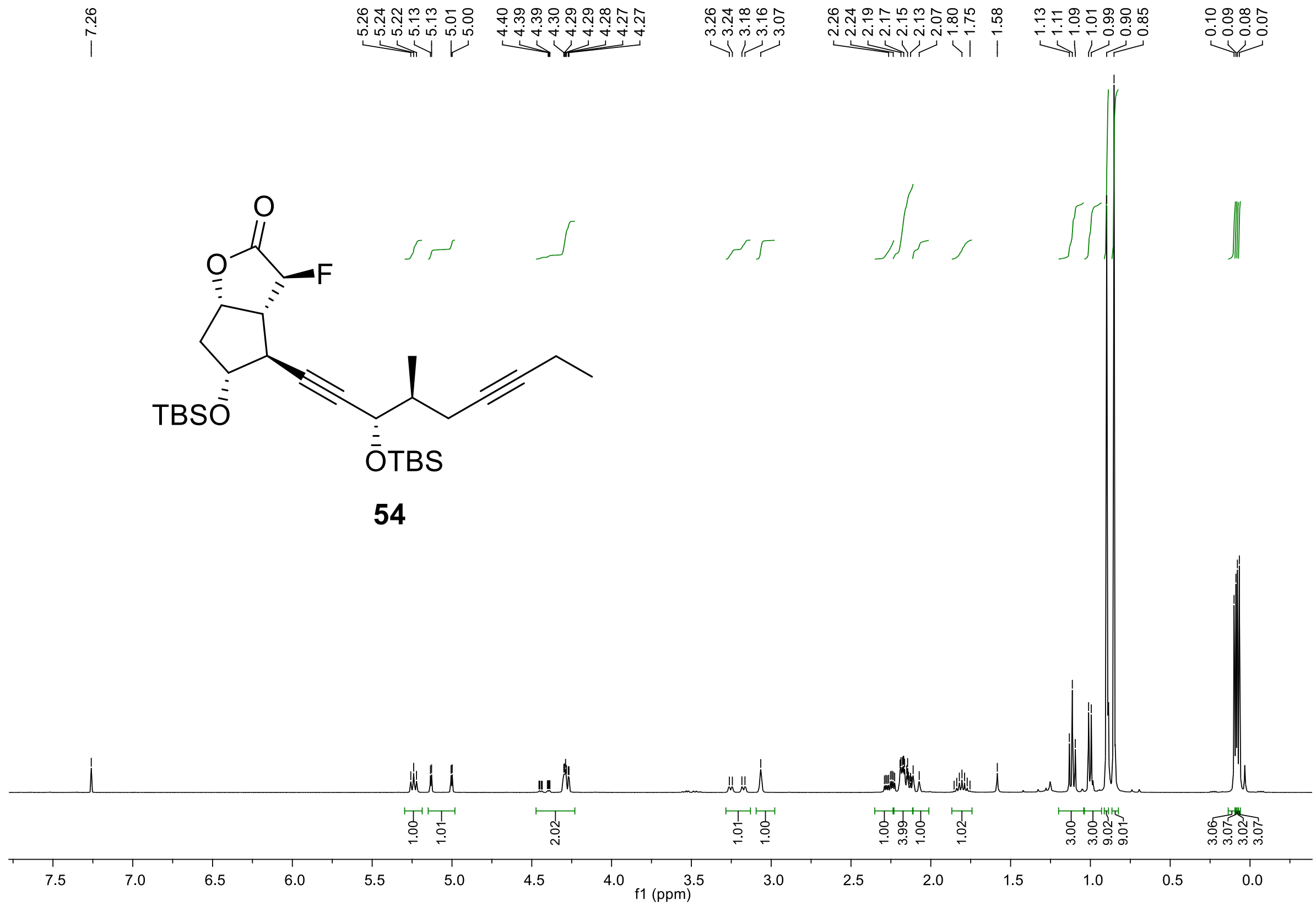

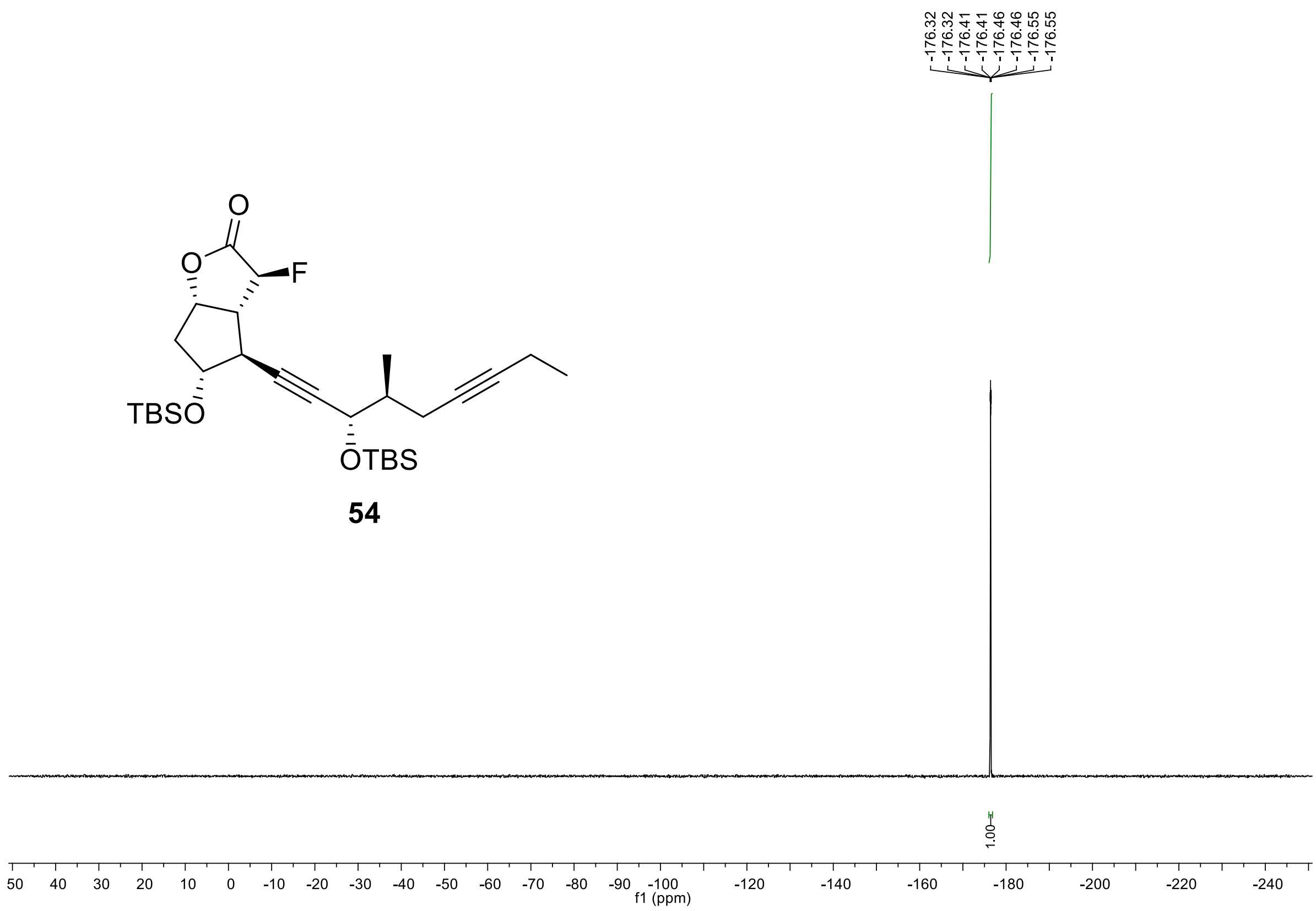

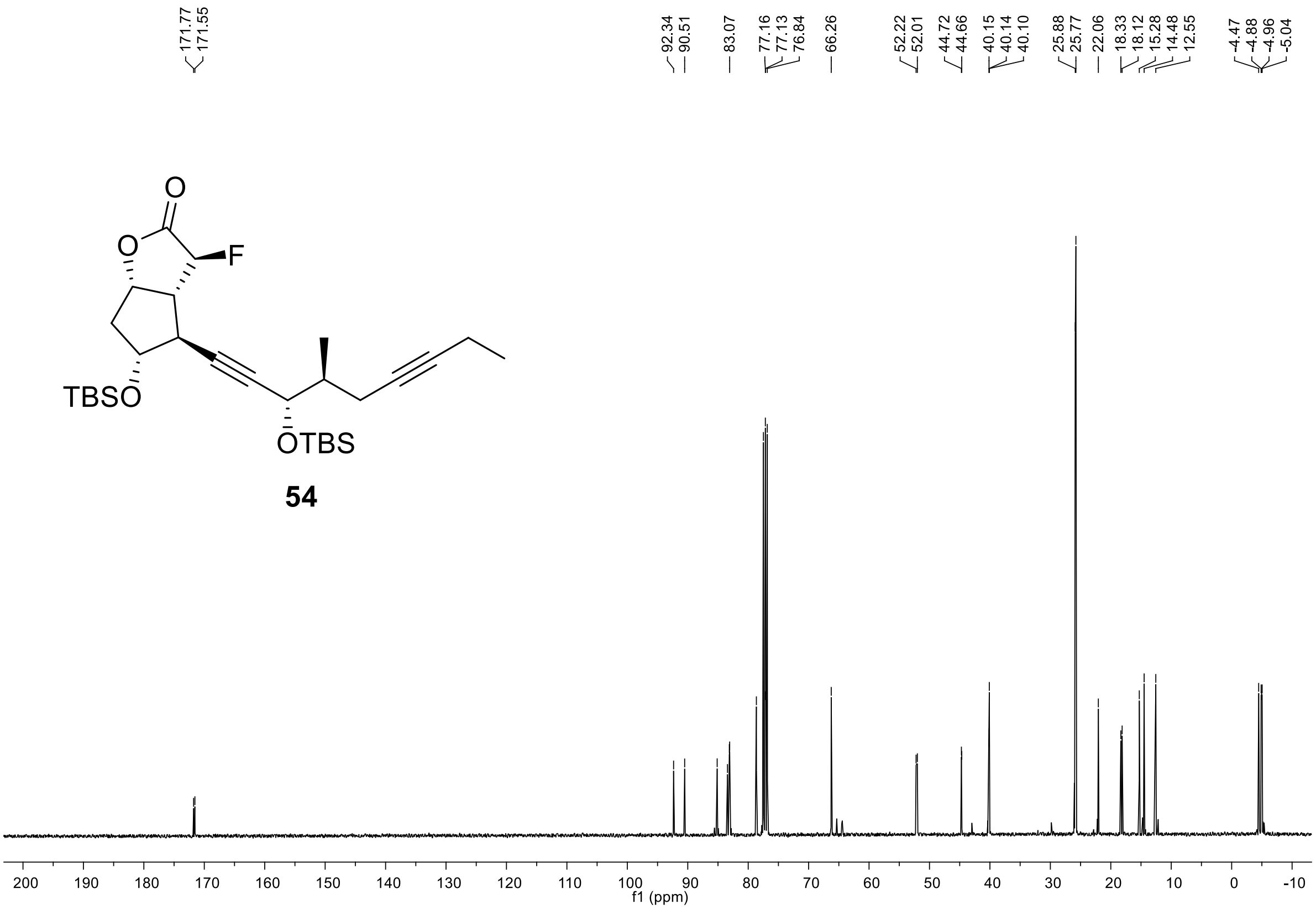
171.77
171.55
92.34
90.51
83.07
77.16
77.13
76.84
66.26
52.22
52.01
44.72
44.66
40.15
40.14
40.10
25.88
25.77
22.06
18.33
18.12
15.28
14.48
12.55
-4.47
-4.88
-4.96
-5.04
O
O
F
TBSO
OTBS
f1 (ppm)

54

5-((3*S*,3a*R*,4*S*,5*R*,6a*S*)-5-(*tert*-Butyldimethylsilyloxy)-4-((3*S*,4*S*)-3-(*tert*-butyldimethylsilyloxy)-4-methylnona-1,6-diynyl)-3-fluorohexahydro-2*H*-cyclopenta[*b*]furan-2-ylidene)pentanoic acid (55)

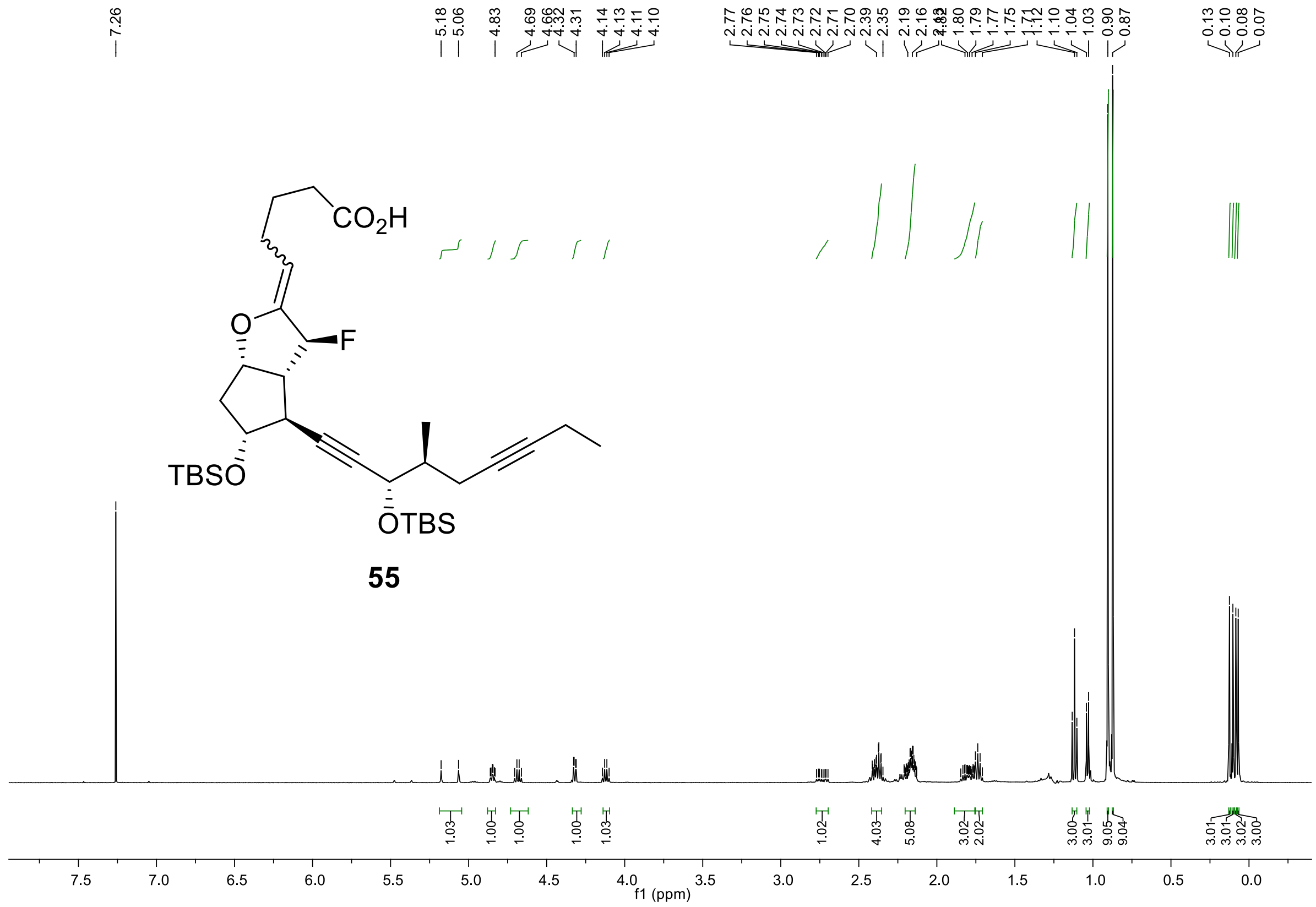

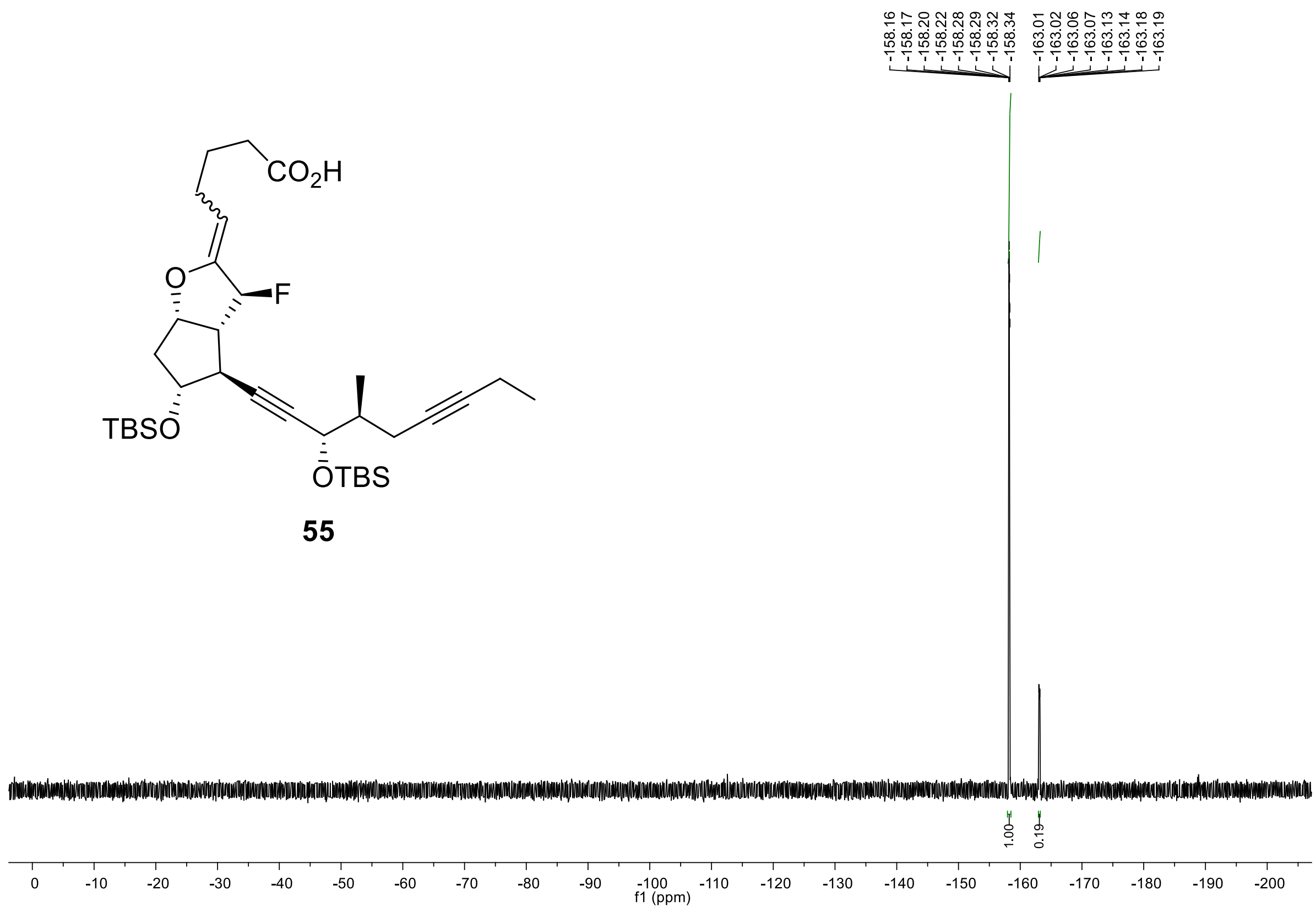

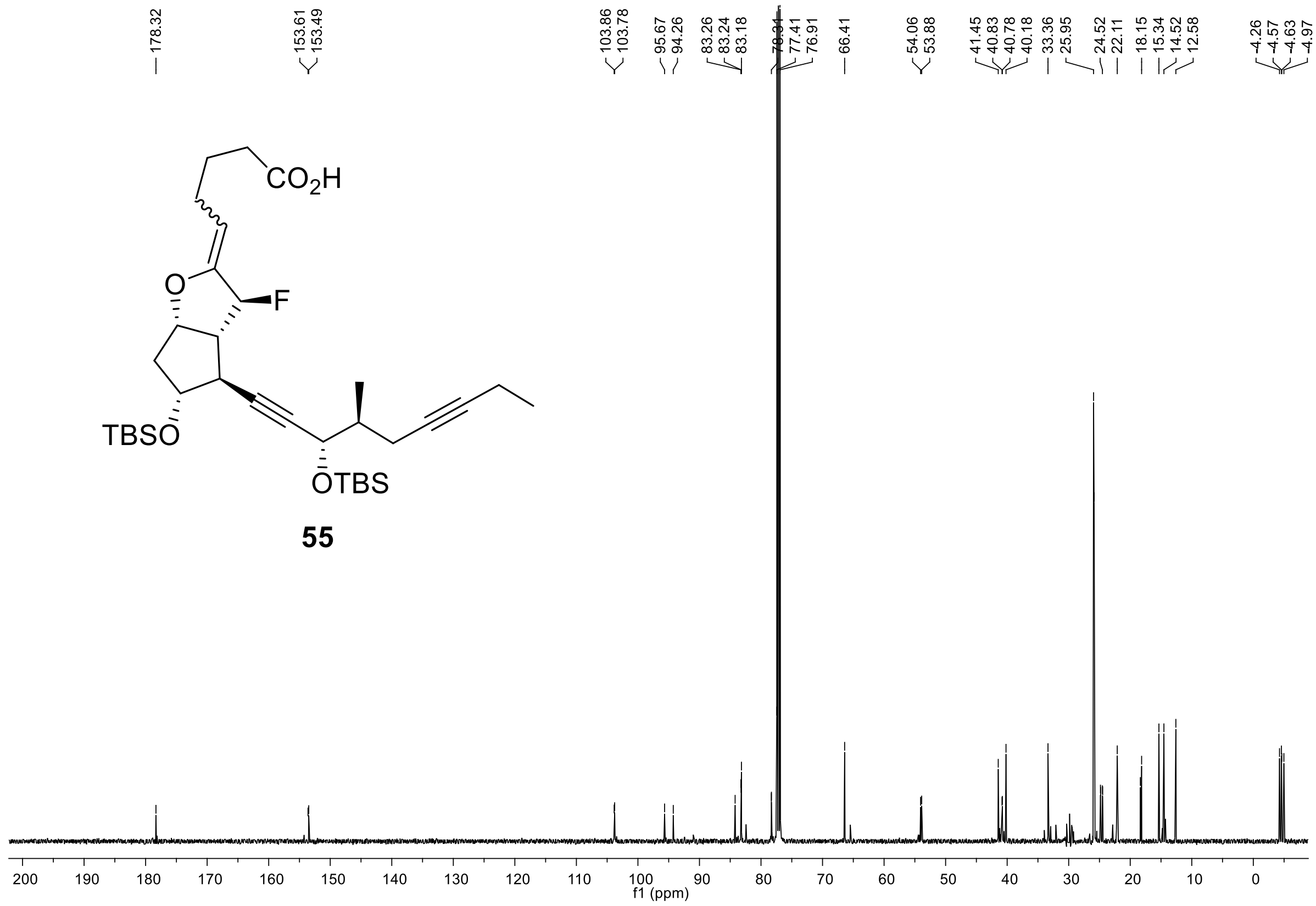
178.32
153.61
153.49
103.86
103.78
95.67
94.26
83.26
83.24
83.18
77.41
76.91
66.41
54.06
53.88
41.45
40.83
40.78
40.18
33.36
25.95
24.52
22.11
18.15
15.34
14.52
12.58
-4.26
-4.57
-4.63
-4.97
CO_2H
O
F
TBSO
OTBS
55
200
190
180
170
160
150
140
130
120
110
100
90
80
70
60
50
40
30
20
10
0
f1 (ppm)

5-((3*S*,3a*R*,4*S*,5*R*,6a*S*)-3-Fluoro-5-hydroxy-4-((3*S*,4*S*)-3-hydroxy-4-methylnona-1,6-diynyl)hexahydro-2*H*-cyclopenta[*b*]furan-2-ylidene)pentanoic acid (5)

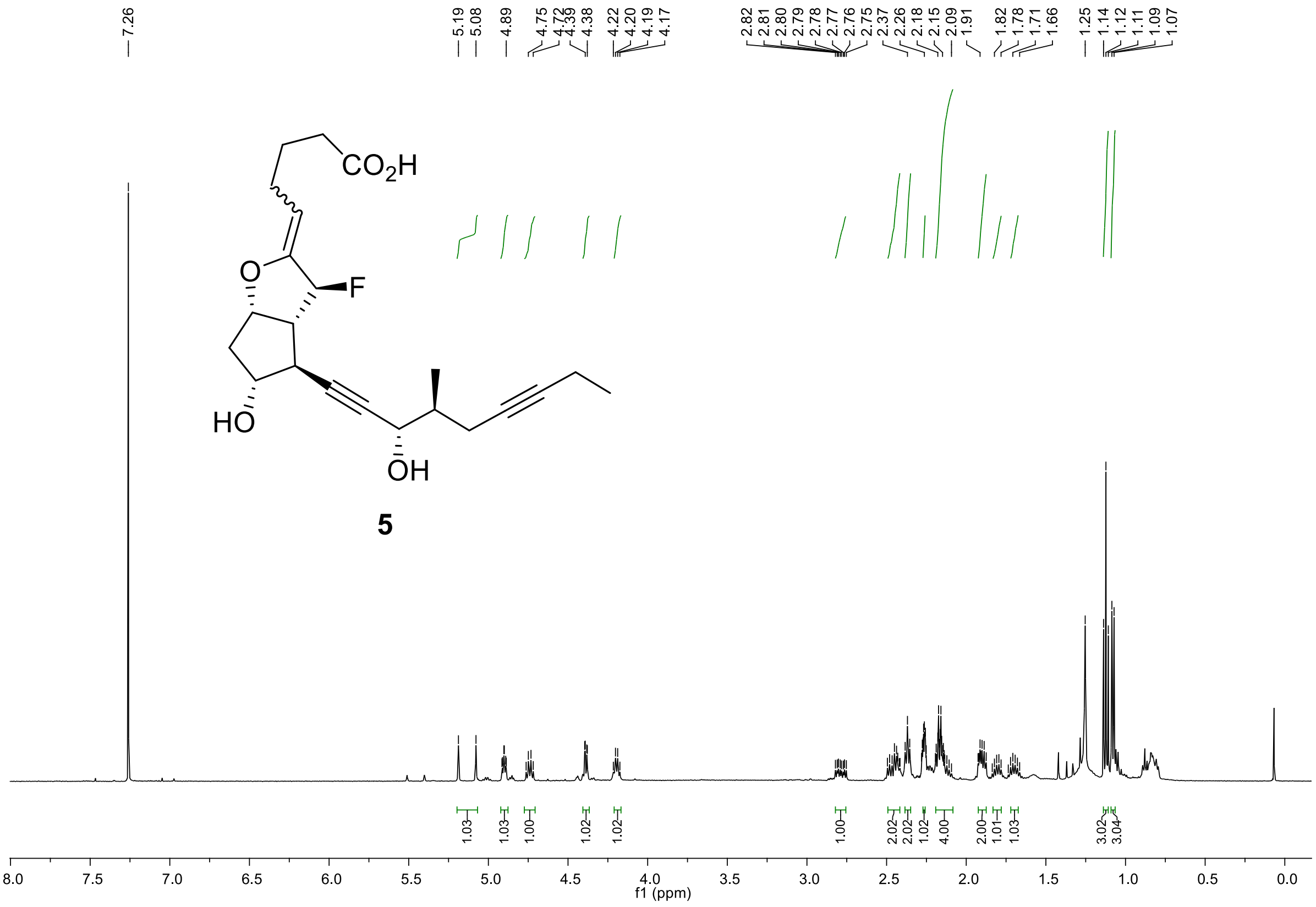

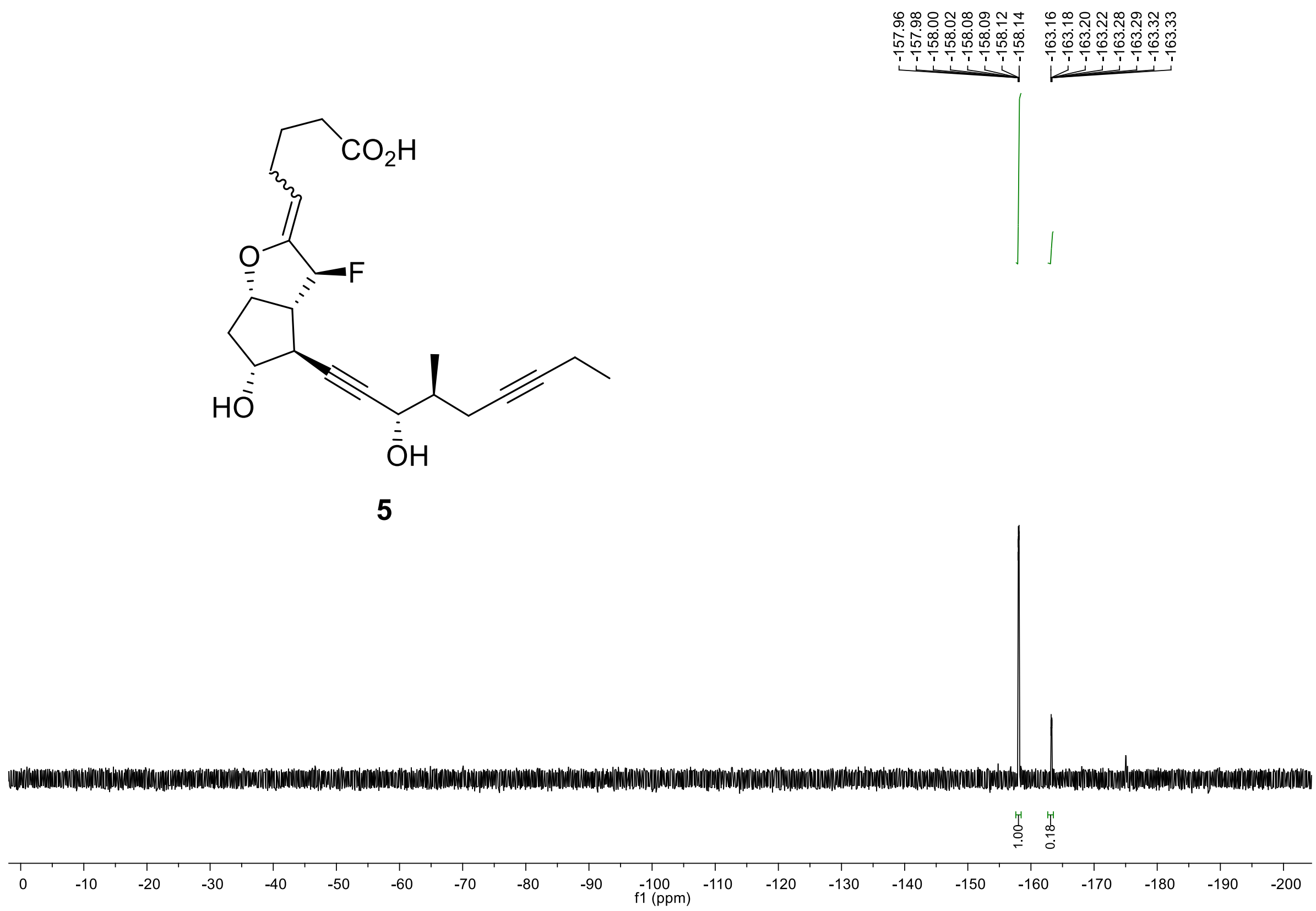

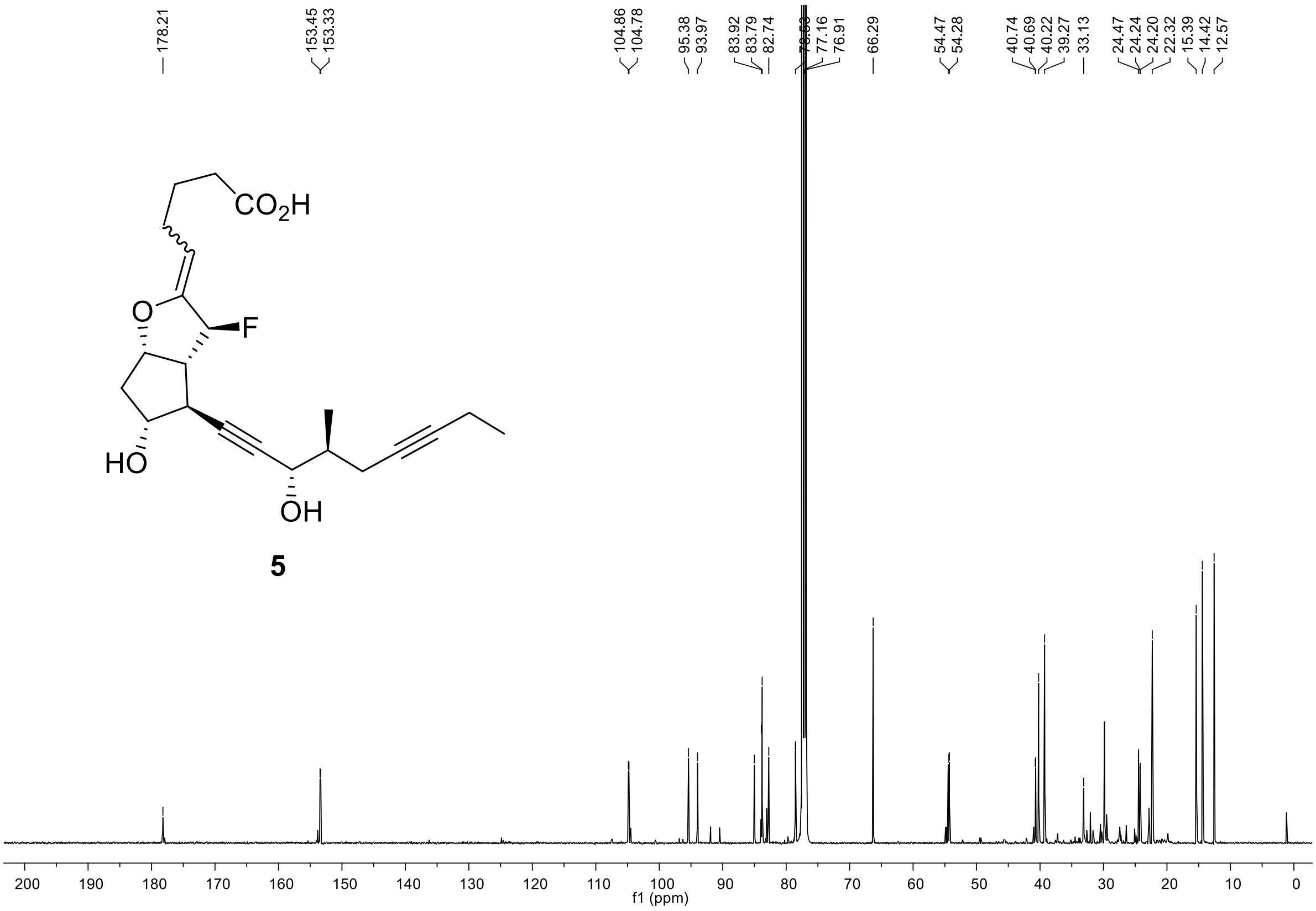
178.21
153.45
153.33
104.86
104.78
95.38
93.97
83.92
83.79
82.74
77.16
76.91
66.29
54.47
54.28
40.74
40.69
40.22
39.27
33.13
24.47
24.24
24.20
22.32
15.39
14.42
12.57
CO2H
O
F
HO
OH
5
200 190 180 170 160 150 140 130 120 110 100 90 80 70 60 50 40 30 20 10 0
f1 (ppm)

(*Z*)-Methyl 5-((3a*R*,4*S*,5*R*,6a*S*)-5-(*tert*-butyldimethylsilyloxy)-4-((3*S*,4*S*)-3-(*tert*-butyldimethylsilyloxy)-4-methylnona-1,6-diynyl)-3,3-difluorohexahydro-2*H*-cyclopenta[*b*]furan-2-ylidene)pentanoate (*Z*-56)

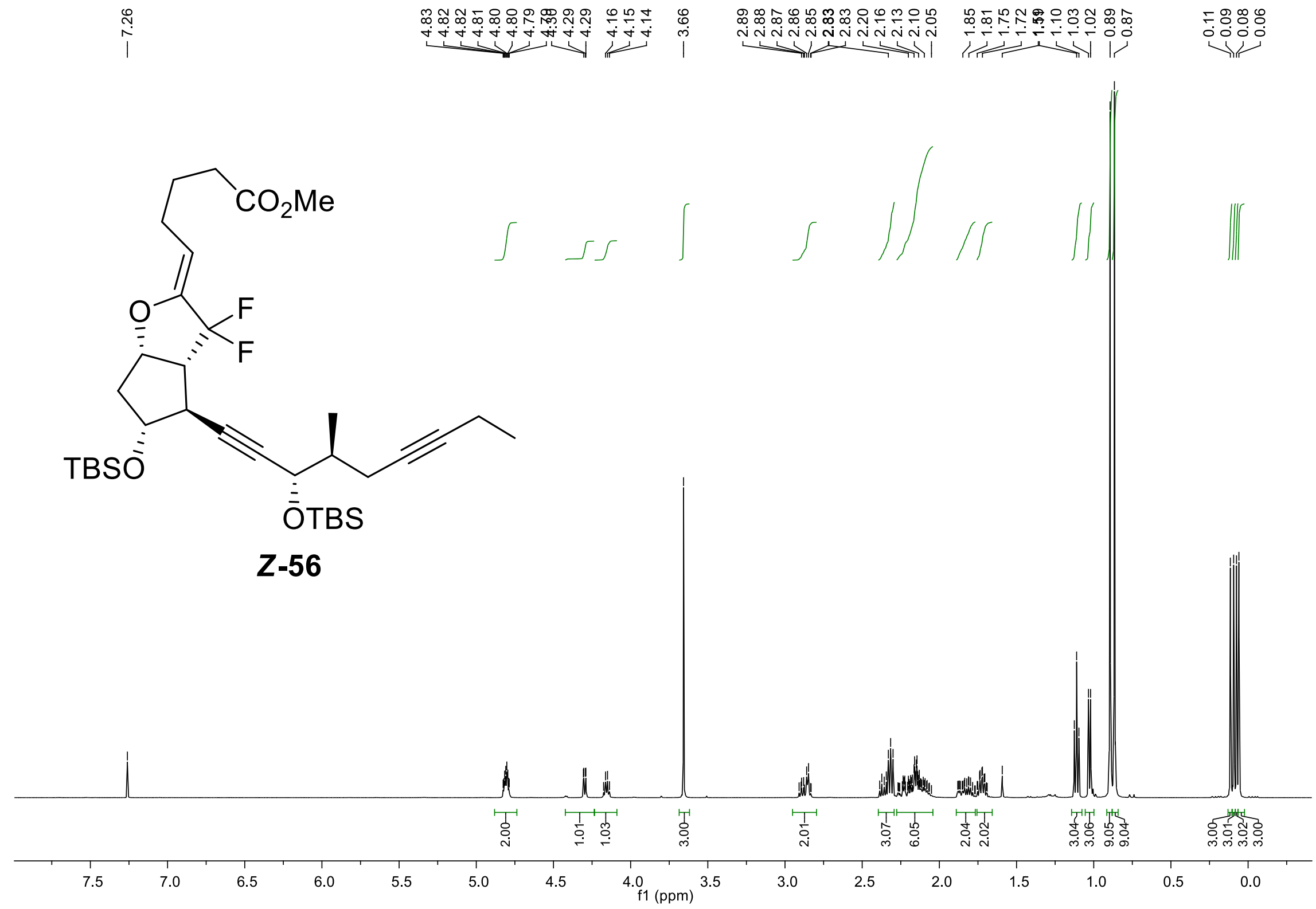

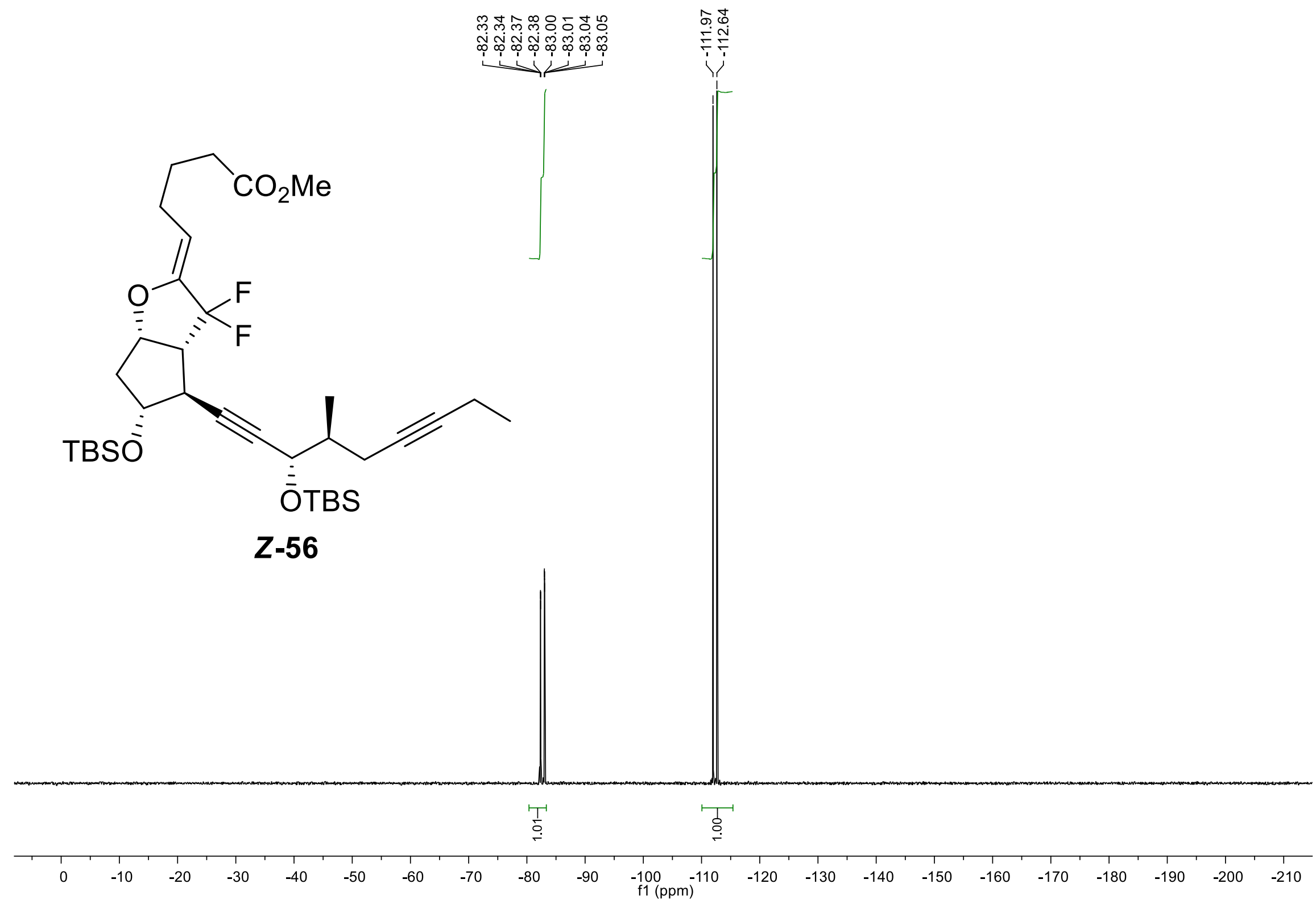

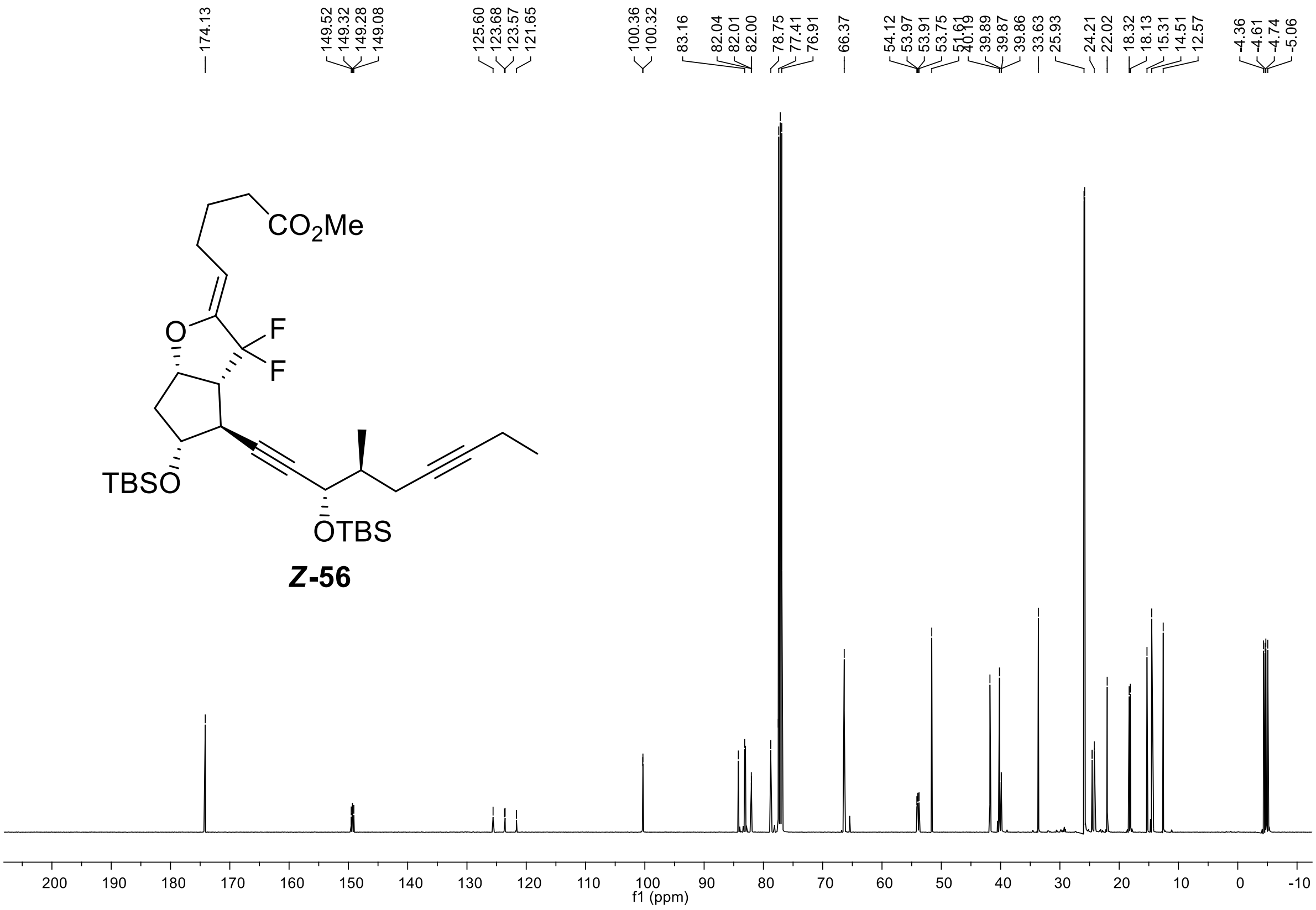
CO2Me
O
F
F
TBSO
OTBS

***Z*-56**

174.13
149.52
149.32
149.28
149.08
125.60
123.68
123.57
121.65
100.36
100.32
83.16
82.04
82.01
82.00
78.75
77.41
76.91
66.37
54.12
53.97
53.91
53.75
51.61
40.19
39.89
39.87
39.86
33.63
25.93
24.21
22.02
18.32
18.13
15.31
14.51
12.57
-4.36
-4.61
-4.74
-5.06
200 190 180 170 160 150 140 130 120 110 100 90 80 70 60 50 40 30 20 10 0 -10
f1 (ppm)

(*E*)-Methyl 5-((3a*R*,4*S*,5*R*,6a*S*)-5-(*tert*-butyldimethylsilyloxy)-4-((3*S*,4*S*)-3-(*tert*-butyldimethylsilyloxy)-4-methylnona-1,6-diynyl)-3,3-difluorohexahydro-2*H*-cyclopenta[*b*]furan-2-ylidene)pentanoate (*E*-56)

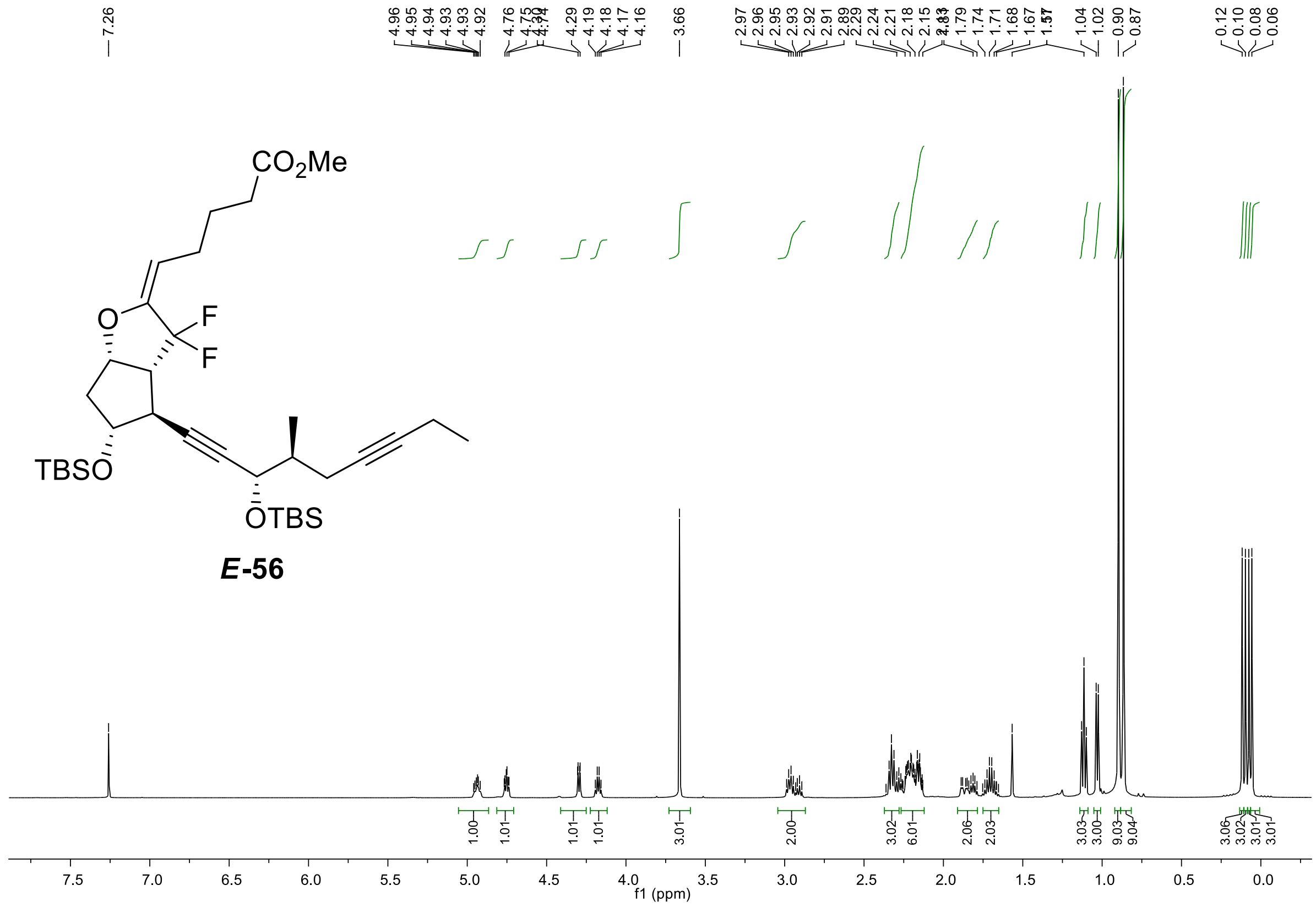

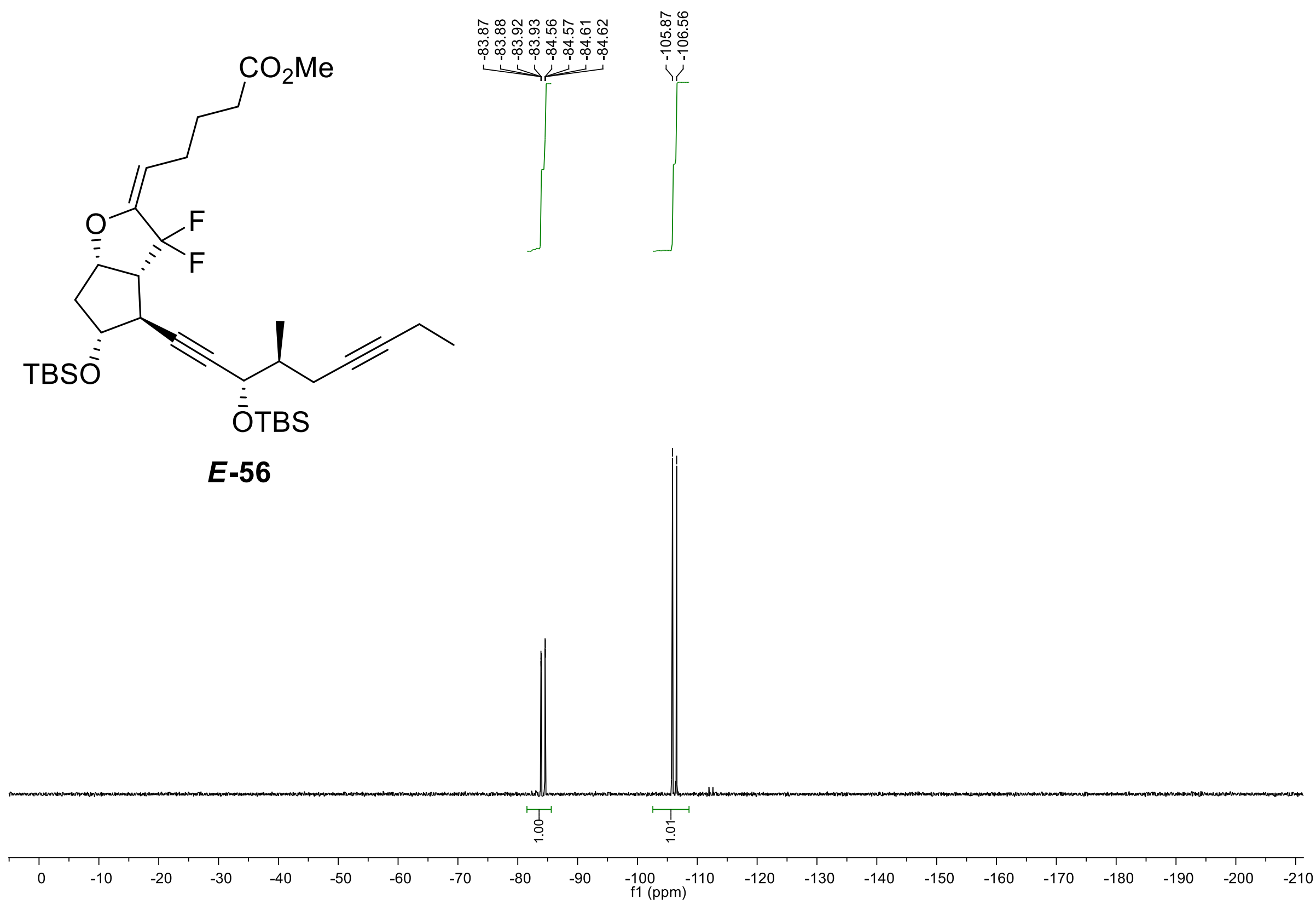

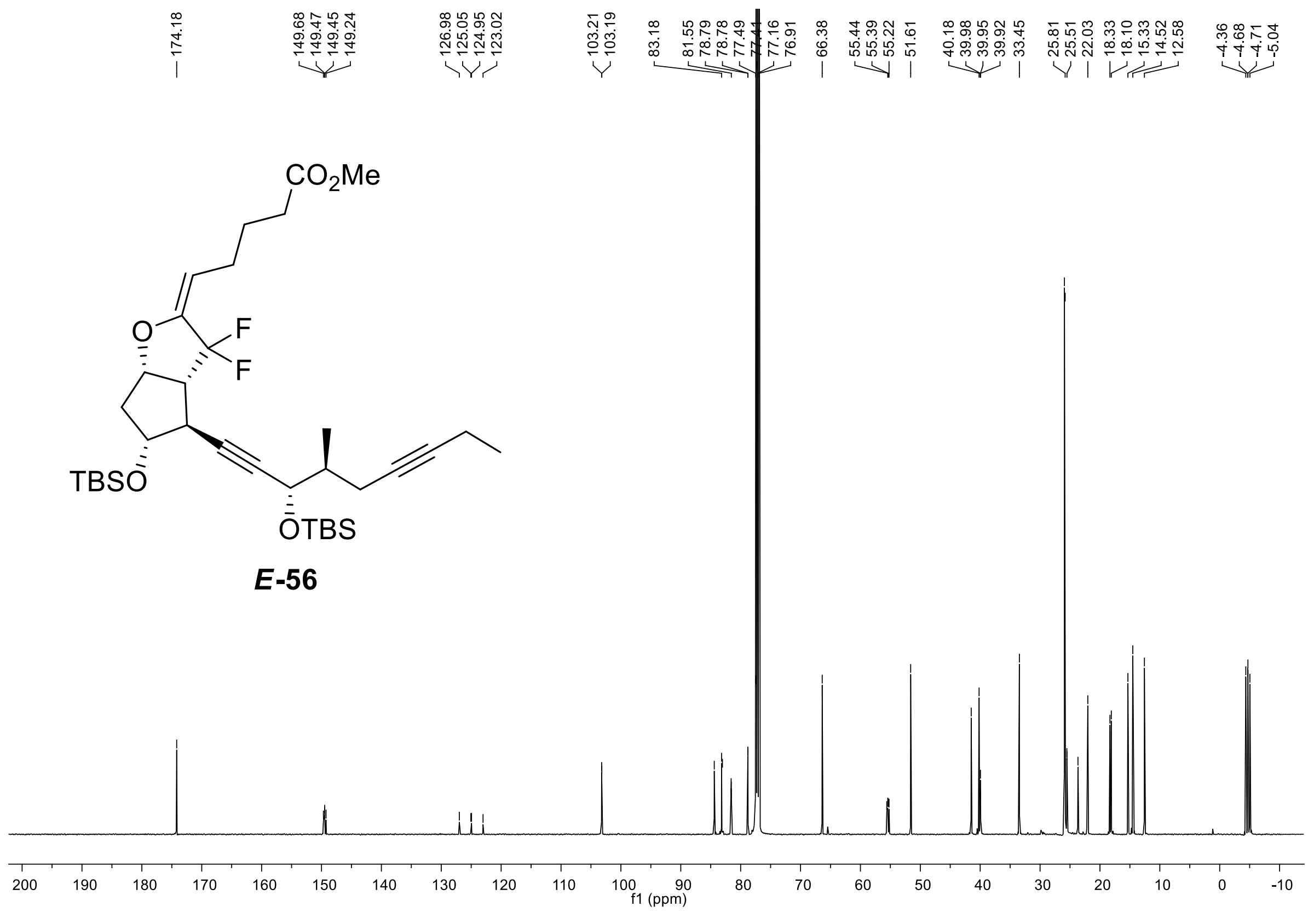
174.18
149.68
149.47
149.45
149.24
126.98
125.05
124.95
123.02
103.21
103.19
83.18
81.55
78.79
78.78
77.49
77.41
77.16
76.91
66.38
55.44
55.39
55.22
51.61
40.18
39.98
39.95
39.92
33.45
25.81
25.51
22.03
18.33
18.10
15.33
14.52
12.58
-4.36
-4.68
-4.71
-5.04
CO2Me
O
F
F
TBSO
OTBS
E-56
200 190 180 170 160 150 140 130 120 110 100 90 80 70 60 50 40 30 20 10 0 -10
f1 (ppm)

(*Z*)-Methyl 5-((3a*R*,4*S*,5*R*,6a*S*)-3,3-difluoro-5-hydroxy-4-((3*S*,4*S*)-3-hydroxy-4-methylnona-1,6-diynyl)hexahydro-2*H*-cyclopenta[*b*]furan-2-ylidene)pentanoate (*Z*-57)

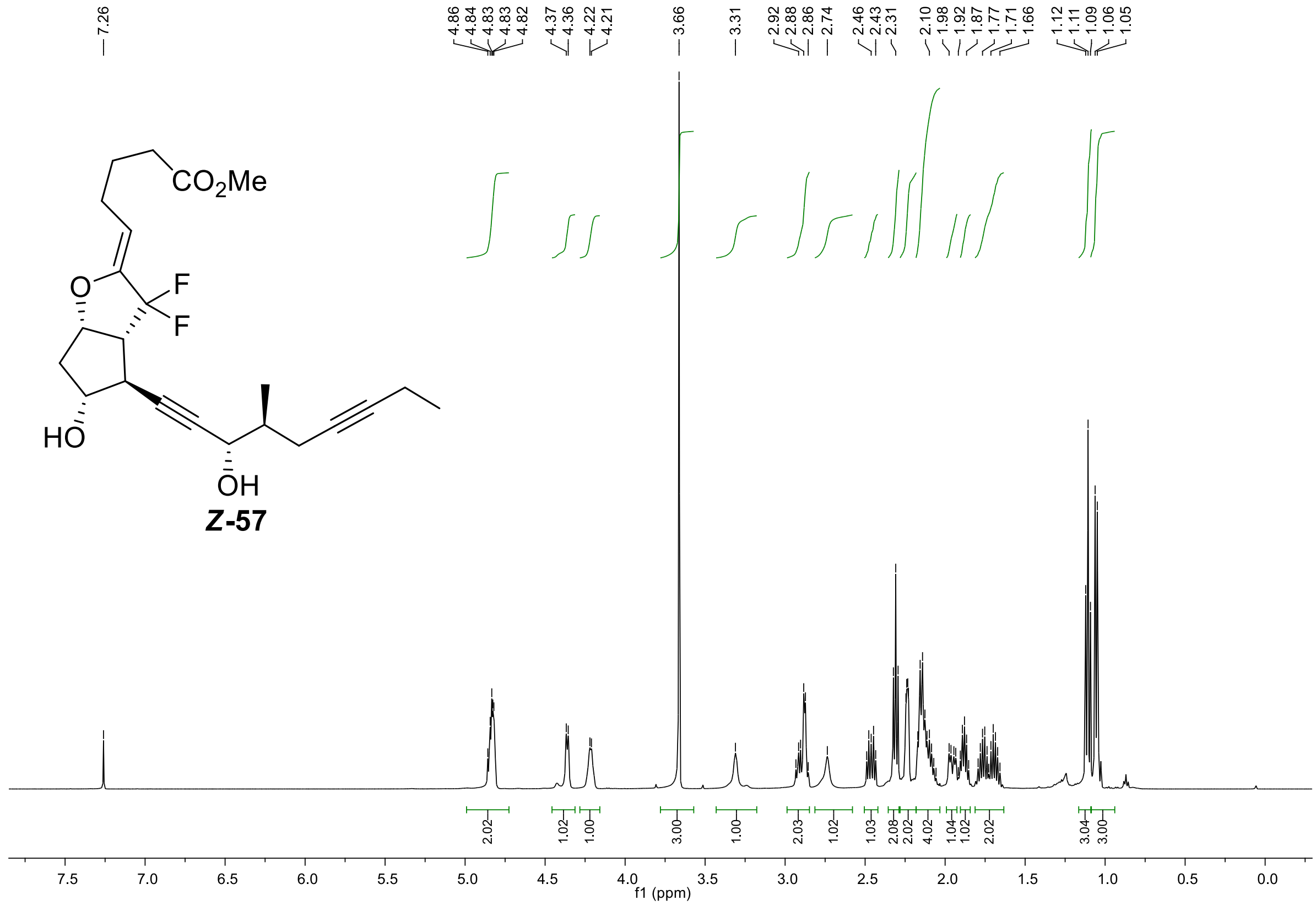

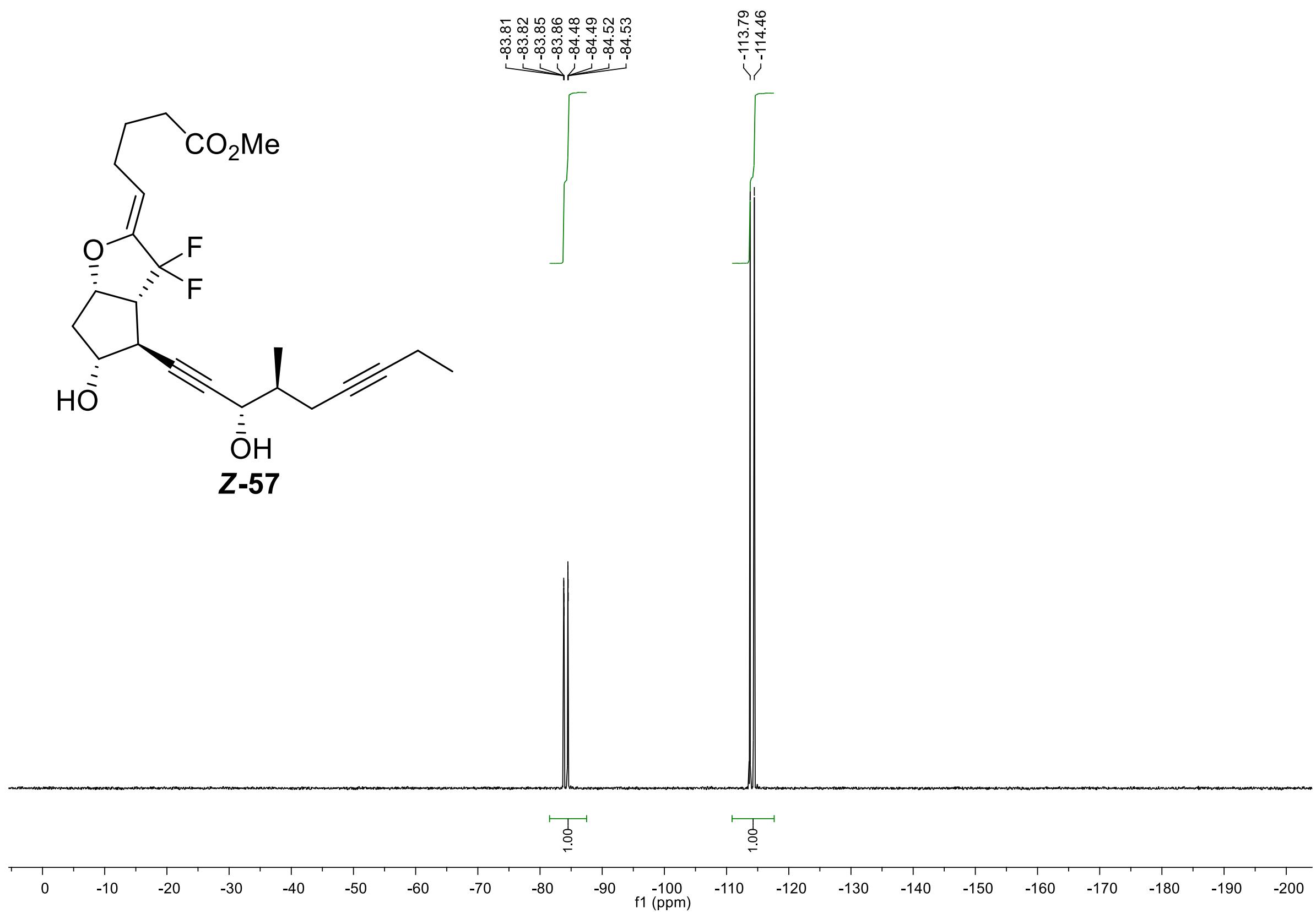

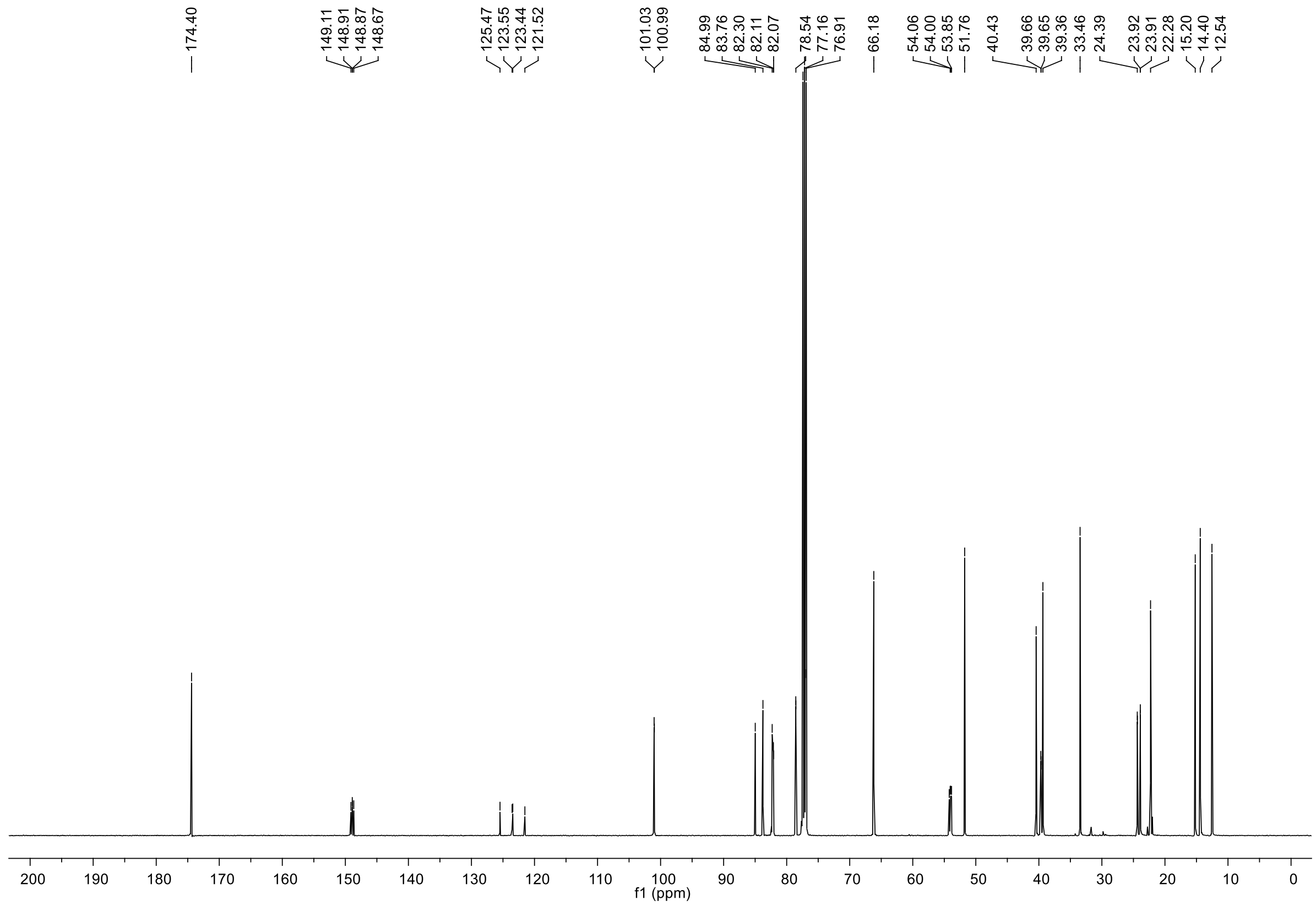
174.40
149.11
148.91
148.87
148.67
125.47
123.55
123.44
121.52
101.03
100.99
84.99
83.76
82.30
82.11
82.07
78.54
77.16
76.91
66.18
54.06
54.00
53.85
51.76
40.43
39.66
39.65
39.36
33.46
24.39
23.92
23.91
22.28
15.20
14.40
12.54
f1 (ppm)

(*E*)-Methyl 5-((3a*R*,4*S*,5*R*,6a*S*)-3,3-difluoro-5-hydroxy-4-((3*S*,4*S*)-3-hydroxy-4-methylnona-1,6-diynyl)hexahydro-2*H*-cyclopenta[*b*]furan-2-ylidene)pentanoate (*E*-57)

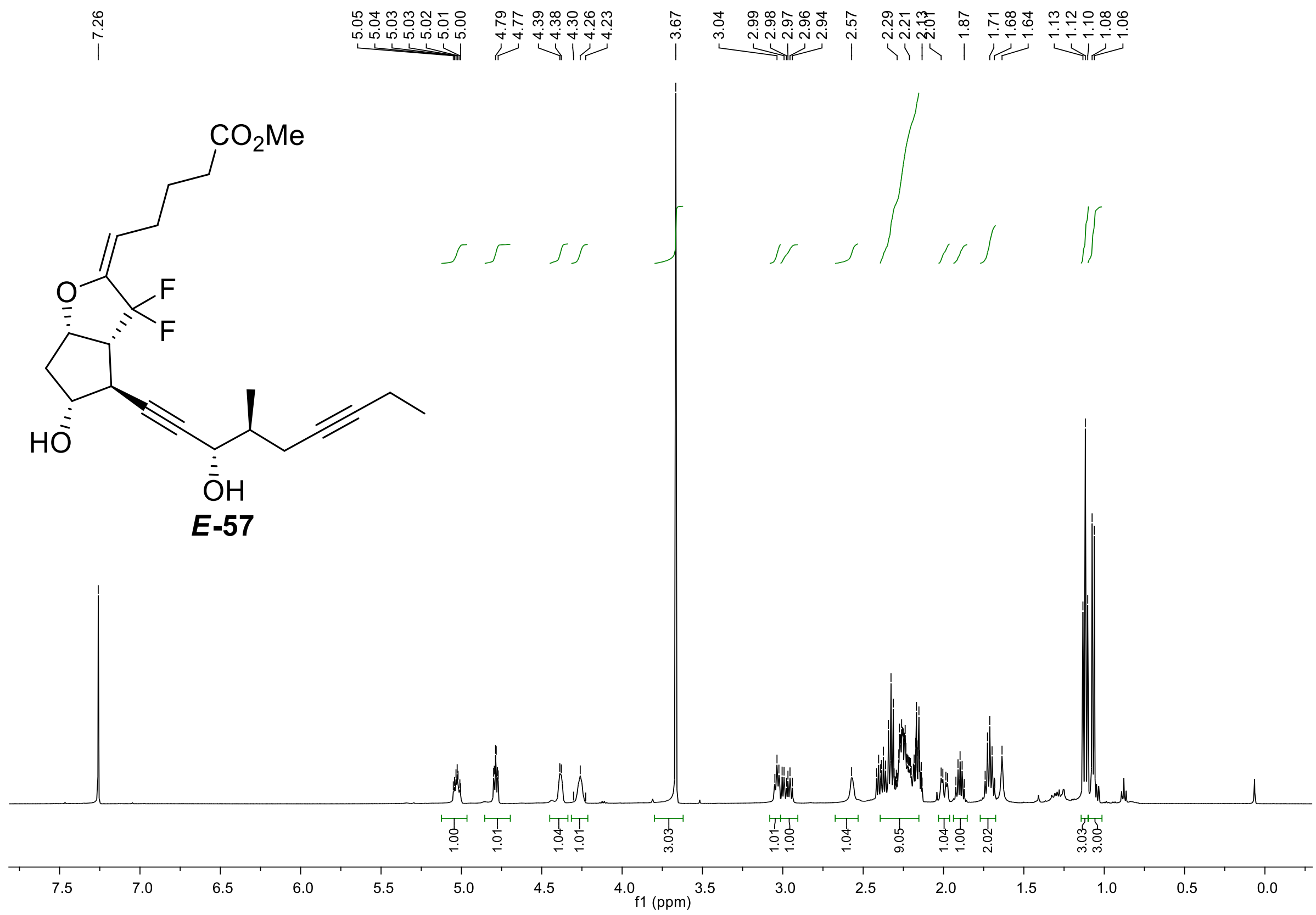

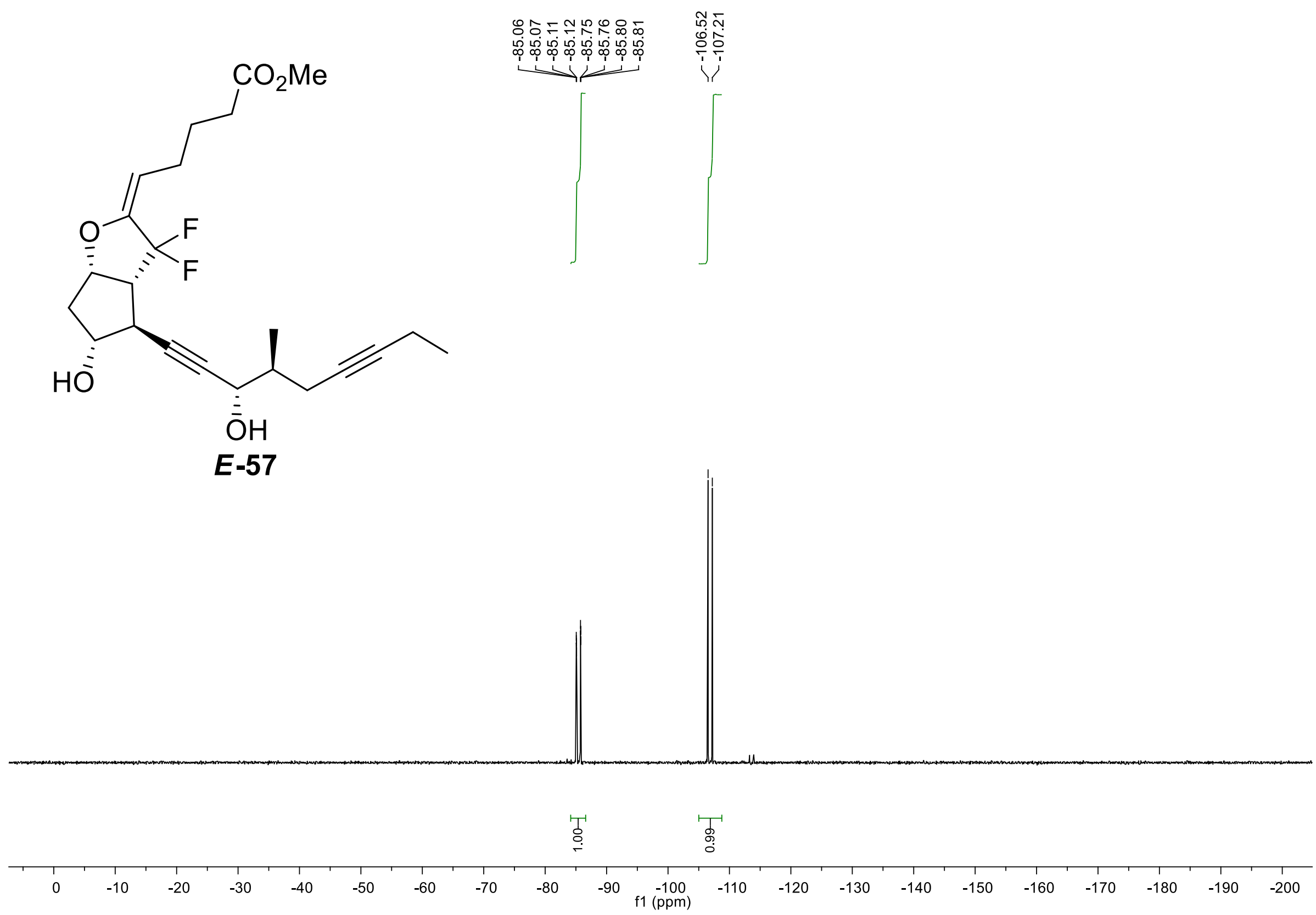

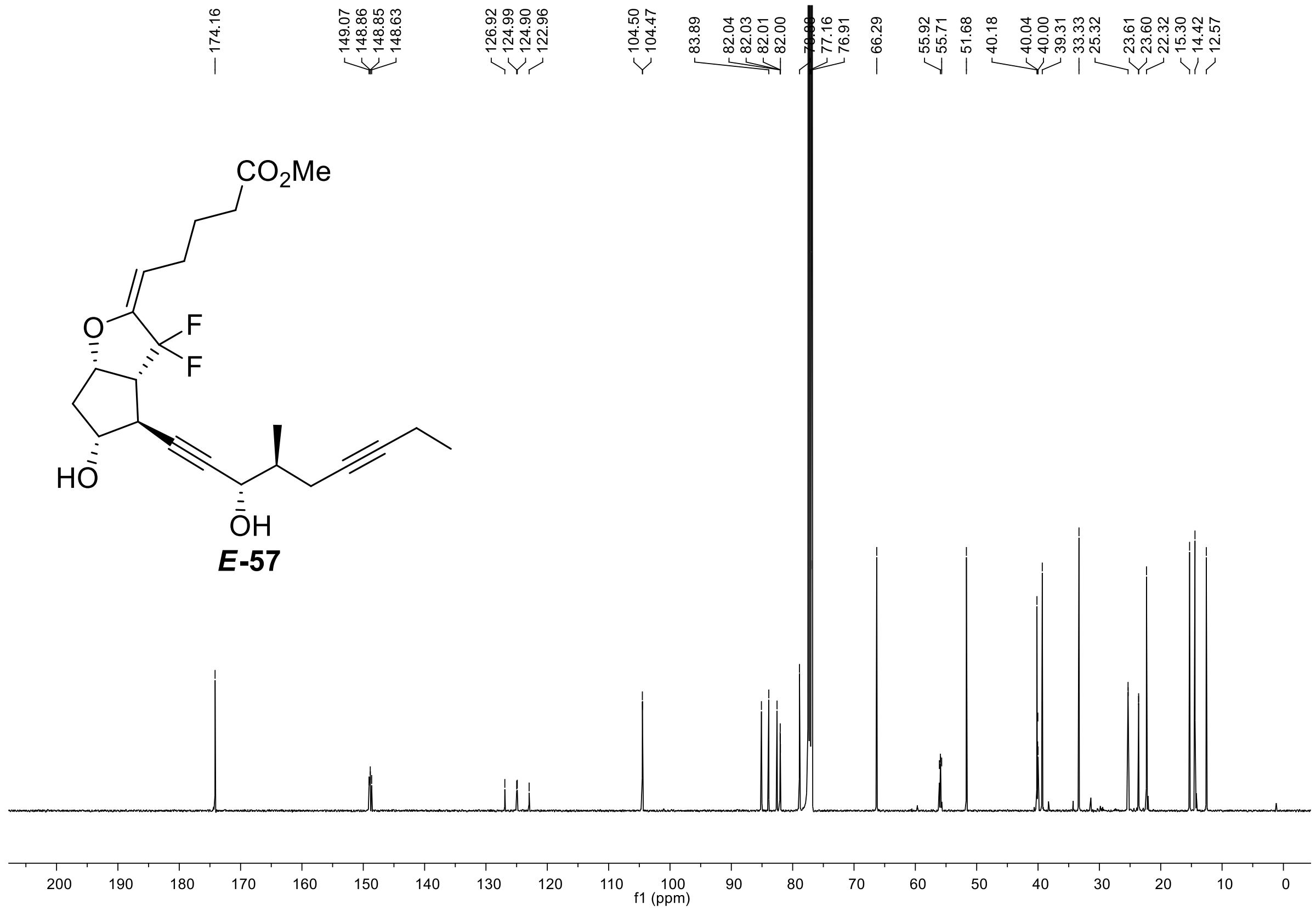

174.16
149.07
148.86
148.85
148.63
126.92
124.99
124.90
122.96
104.50
104.47
83.89
82.04
82.03
82.01
82.00
77.16
76.91
66.29
55.92
55.71
51.68
40.18
40.04
40.00
39.31
33.33
25.32
23.61
23.60
22.32
15.30
14.42
12.57
CO2Me
O
F
F
HO
OH
E-57
200 190 180 170 160 150 140 130 120 110 100 90 80 70 60 50 40 30 20 10 0
f1 (ppm)

(*Z*)-5-((3a*R*,4*S*,5*R*,6a*S*)-3,3-Difluoro-5-hydroxy-4-((3*S*,4*S*)-3-hydroxy-4-methylnona-1,6-diynyl)hexahydro-2*H*-cyclopenta[*b*]furan-2-ylidene)pentanoic acid (*Z*-9)

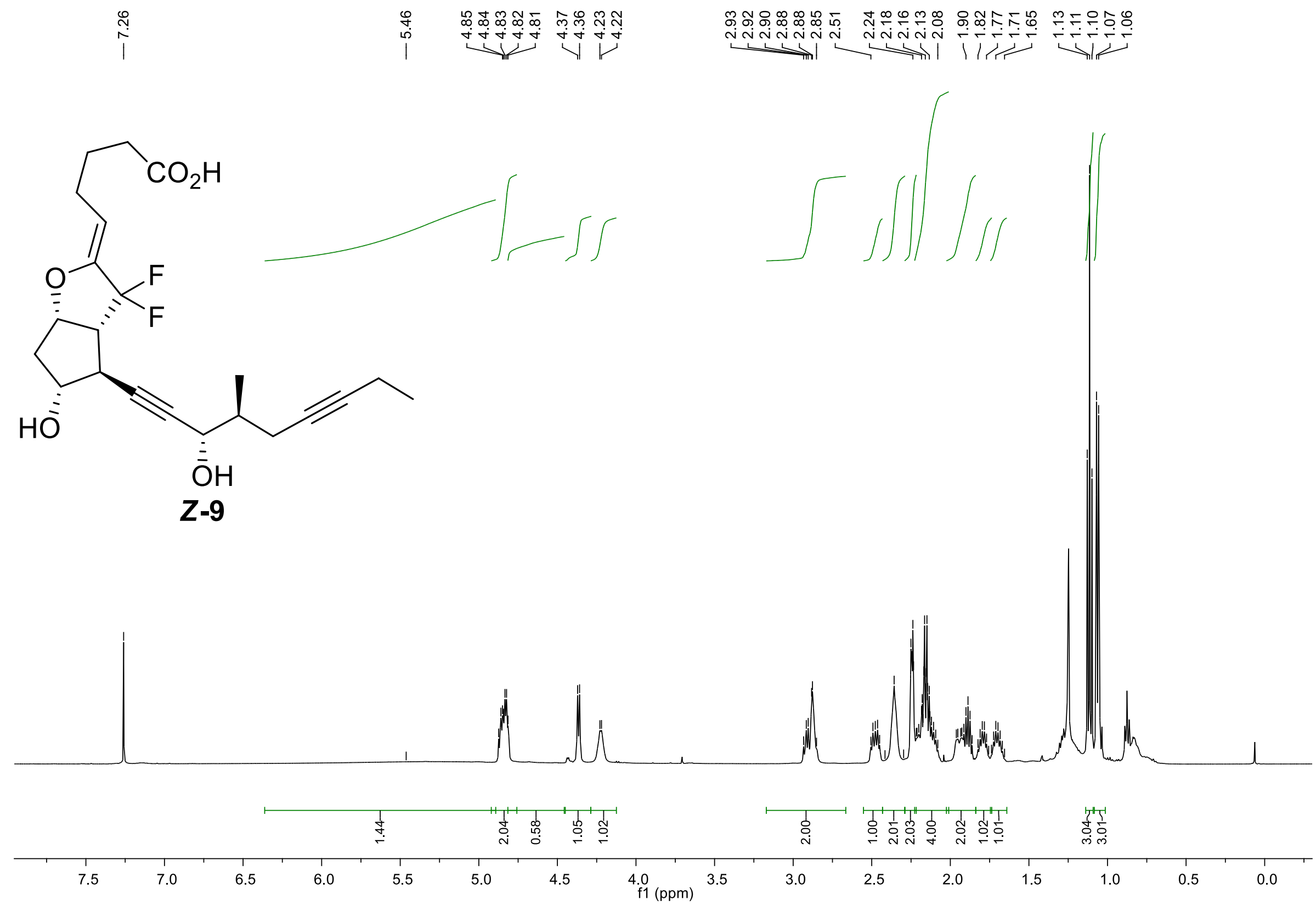

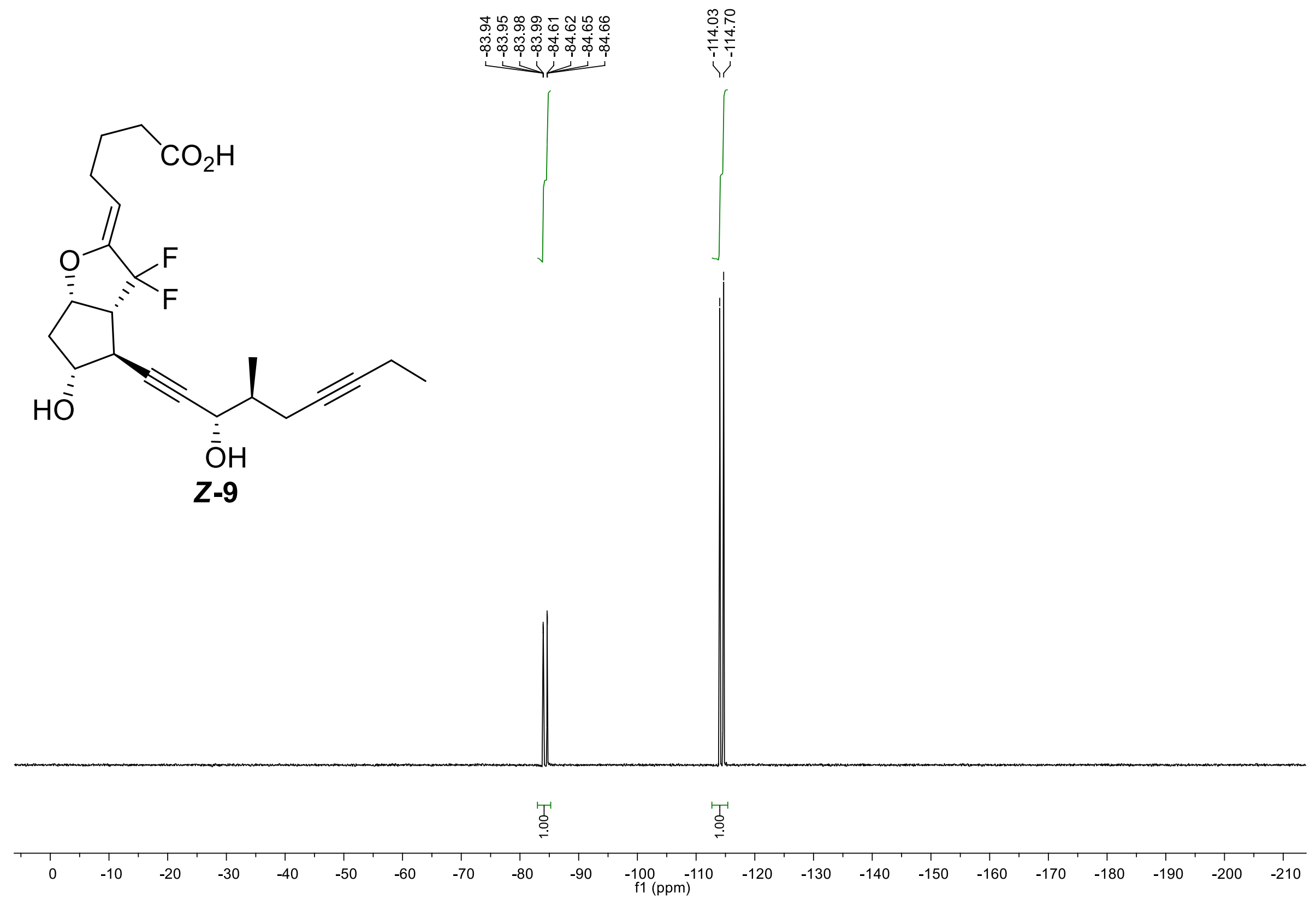

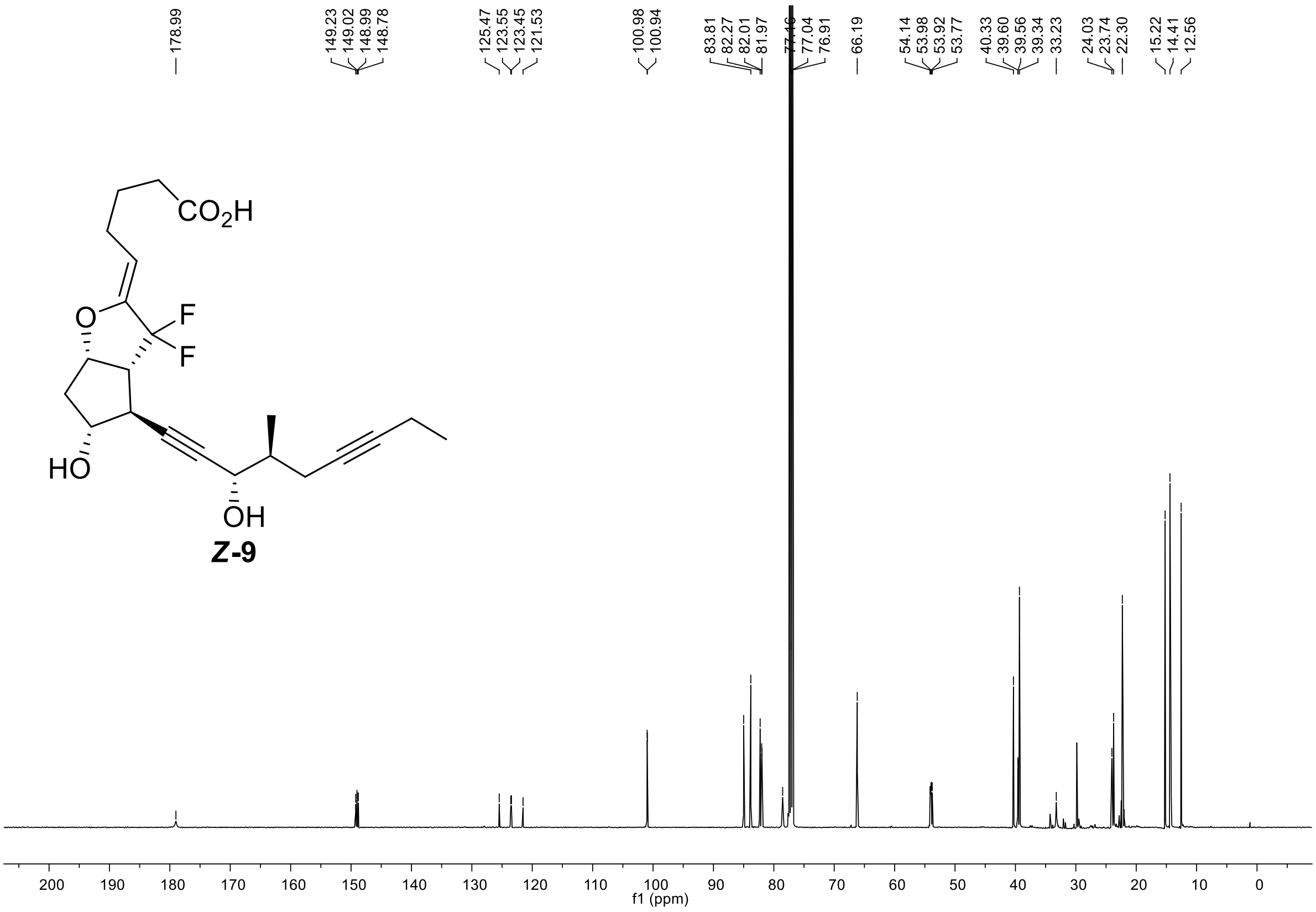
178.99
149.23
149.02
148.99
148.78
125.47
123.55
123.45
121.53
100.98
100.94
83.81
82.27
82.01
81.97
77.16
77.04
76.91
66.19
54.14
53.98
53.92
53.77
40.33
39.60
39.56
39.34
33.23
24.03
23.74
22.30
15.22
14.41
12.56
CO2H
O
F
F
HO
OH
Z-9
200 190 180 170 160 150 140 130 120 110 100 90 80 70 60 50 40 30 20 10 0
f1 (ppm)

(*E*)-5-((3a*R*,4*S*,5*R*,6a*S*)-3,3-Difluoro-5-hydroxy-4-((3*S*,4*S*)-3-hydroxy-4-methylnona-1,6-diynyl)hexahydro-2*H*-cyclopenta[*b*]furan-2-ylidene)pentanoic acid (*E*-9)

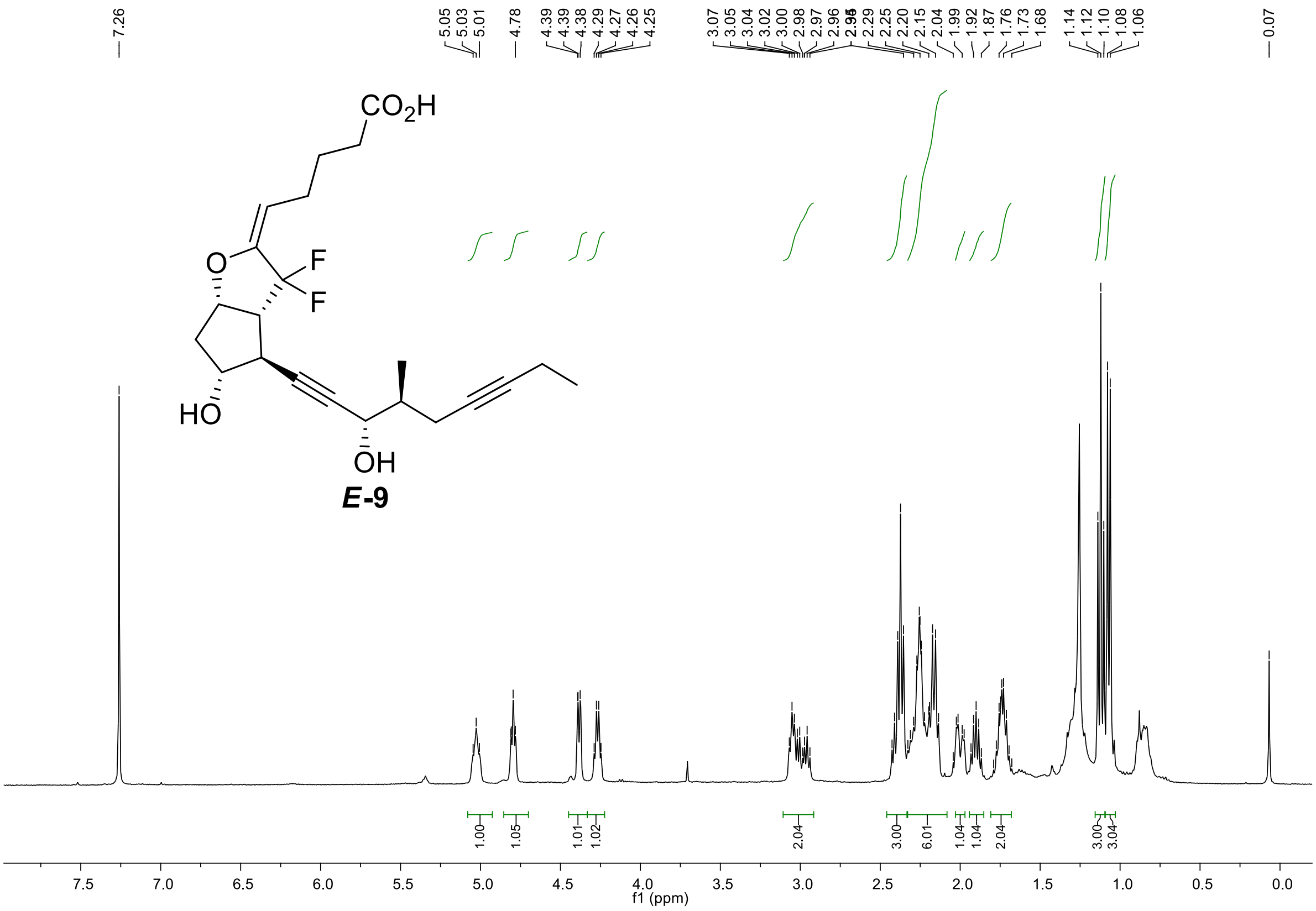

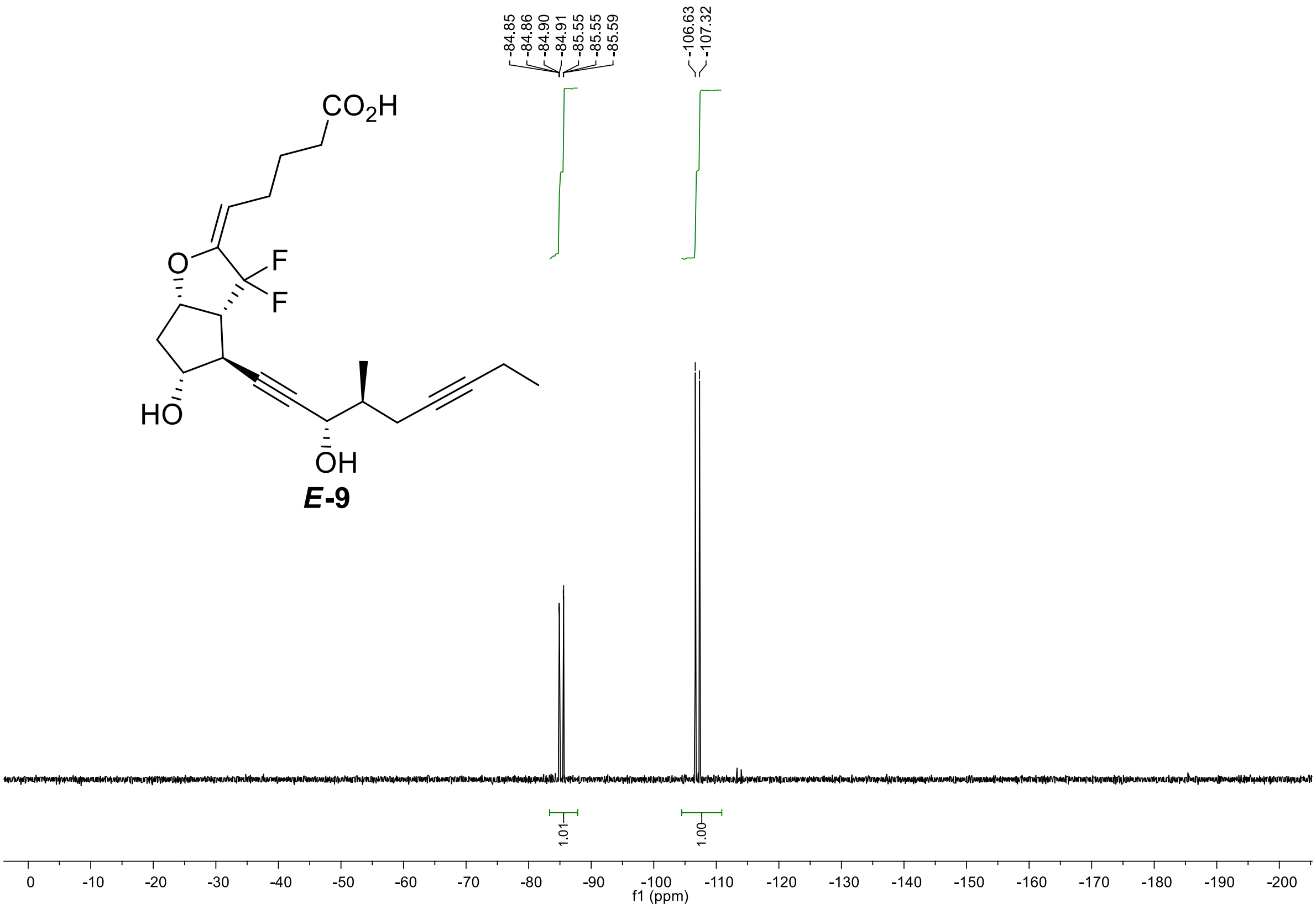

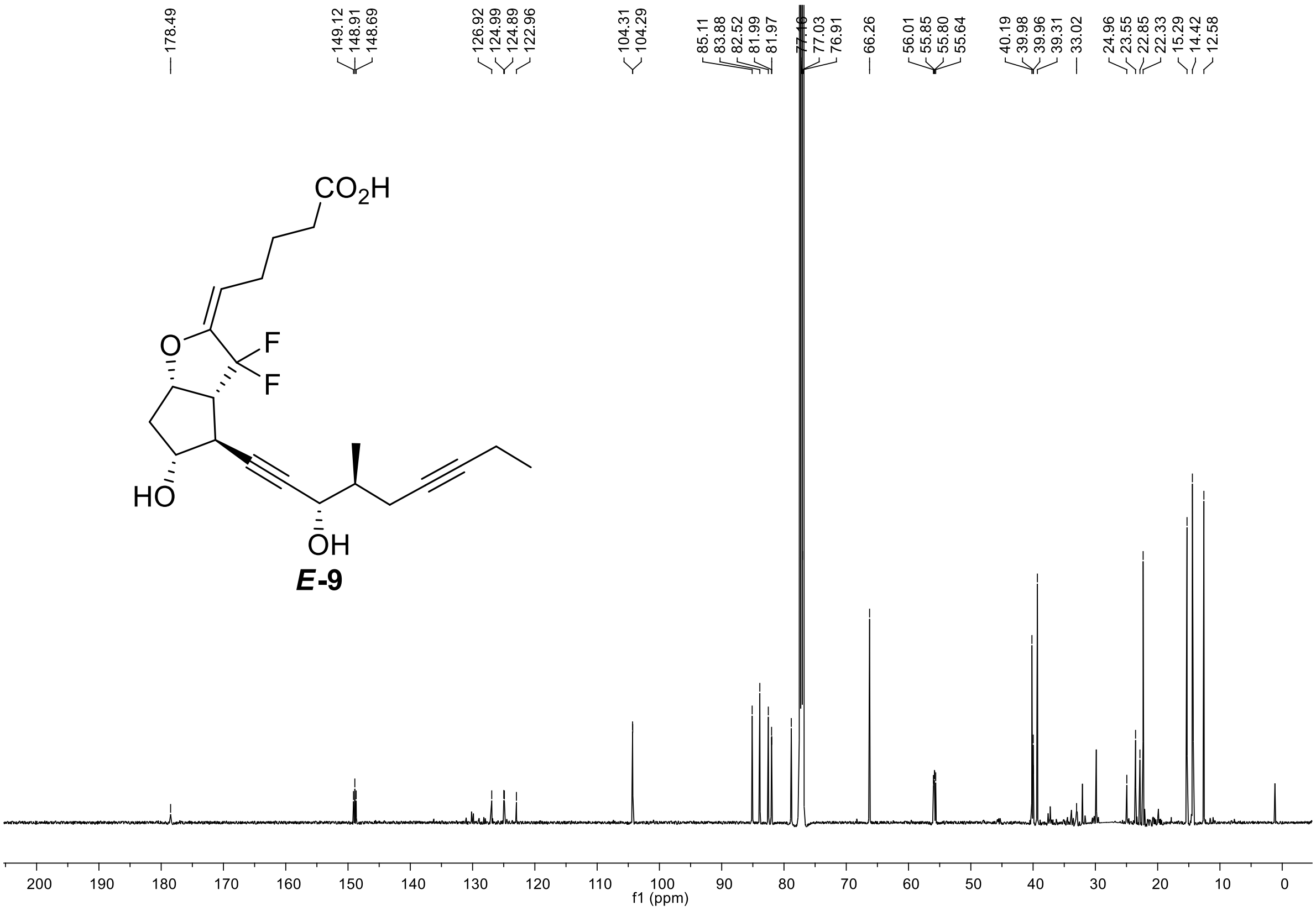

178.49
149.12
148.91
148.69
126.92
124.99
124.89
122.96
104.31
104.29
85.11
83.88
82.52
81.99
81.97
77.03
76.91
66.26
56.01
55.85
55.80
55.64
40.19
39.98
39.96
39.31
33.02
24.96
23.55
22.85
22.33
15.29
14.42
12.58
CO2H
O
F
F
HO
OH
E-9
f1 (ppm)

HPLC Traces for Compounds 5, 7, 8, and 9

Compound **5** Purity: 99.3%

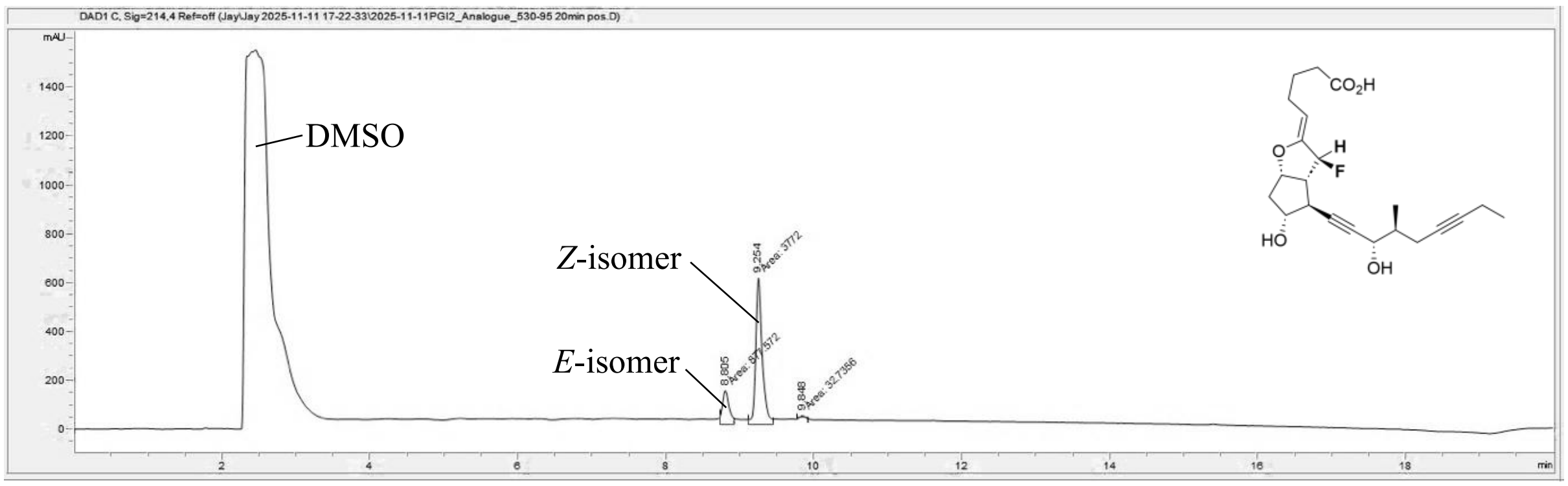

Peak #	RetTime [min]	Type	Width [min]	Area [mAU*s]	Height [mAU]	Area %
1	8.805	MM	0.1041	877.57172	140.47887	18.7423
2	9.254	MM	0.1044	3772.00464	602.43842	80.5586
3	9.848	MM	0.0553	32.73562	9.87163	0.6991
Totals :				4682.31197	752.78891	

Compound **7** Purity: 96.8%

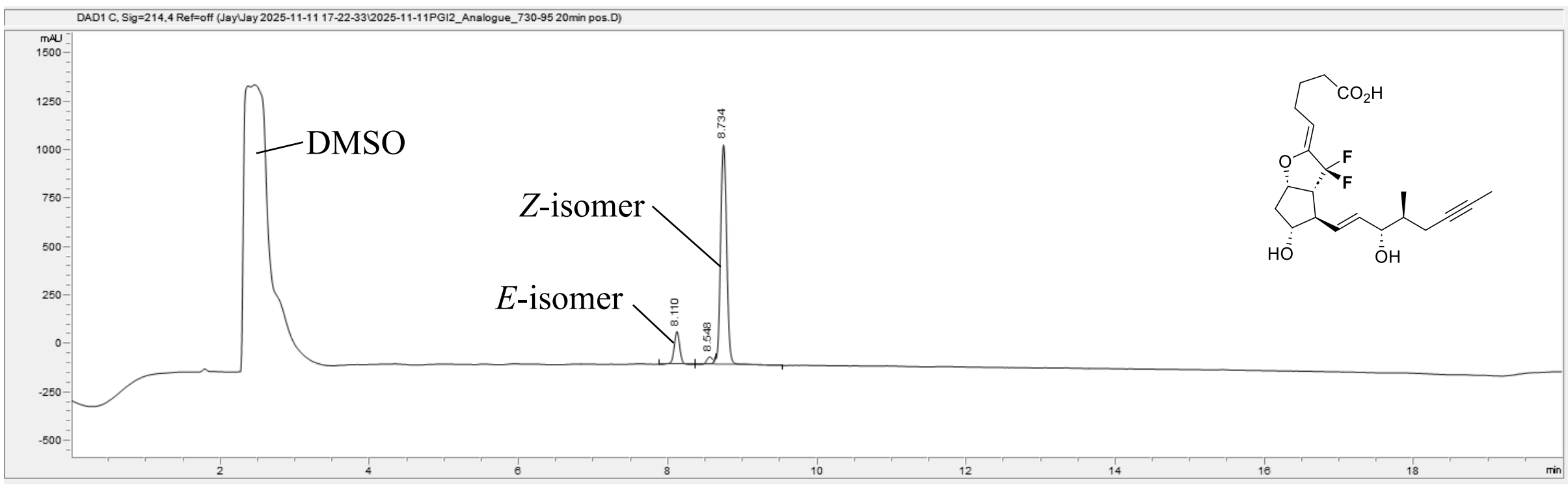

Peak #	RetTime [min]	Type	Width [min]	Area [mAU*s]	Height [mAU]	Area %
1	8.110	BB	0.0822	908.03241	170.24561	11.7565
2	8.548	BV E	0.0921	248.91048	40.28997	3.2227
3	8.734	BB R	0.0910	6566.71387	1138.87061	85.0208
Totals :				7723.65675	1349.40619	

Compound **8b** Purity: 100%

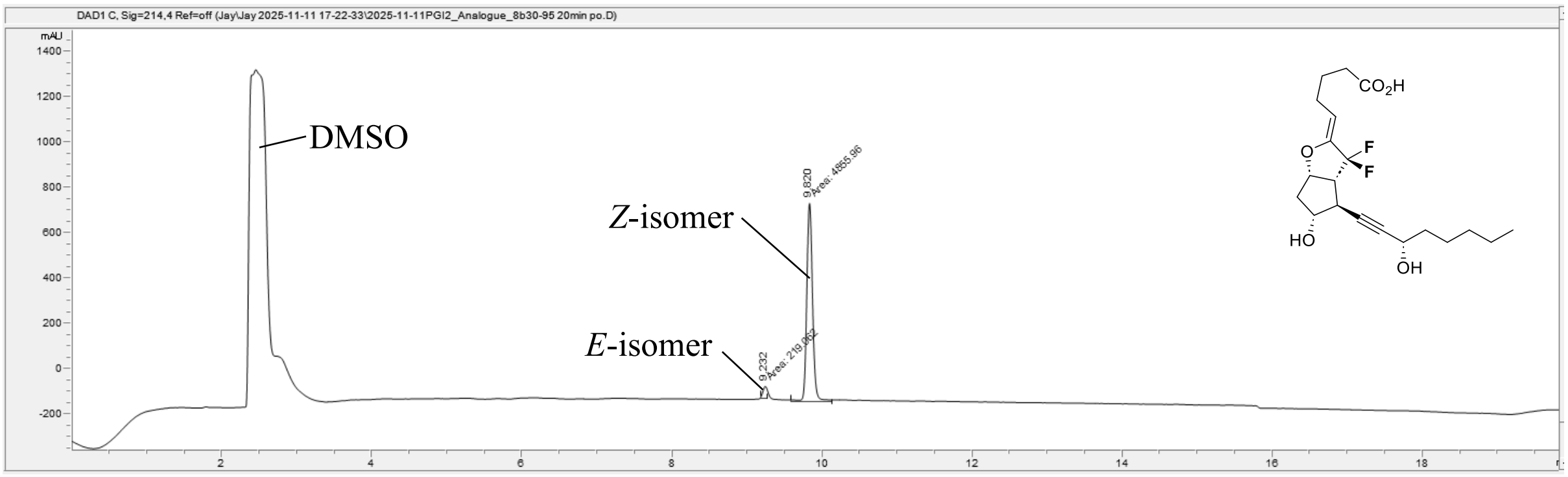

Peak #	RetTime [min]	Type	Width [min]	Area [mAU*s]	Height [mAU]	Area %
1	9.232	MM	0.0677	219.06158	53.90427	4.3165
2	9.820	MM	0.0918	4855.96436	881.30652	95.6835
Totals :				5075.02594	935.21078	

Compound **9** Purity: 100%

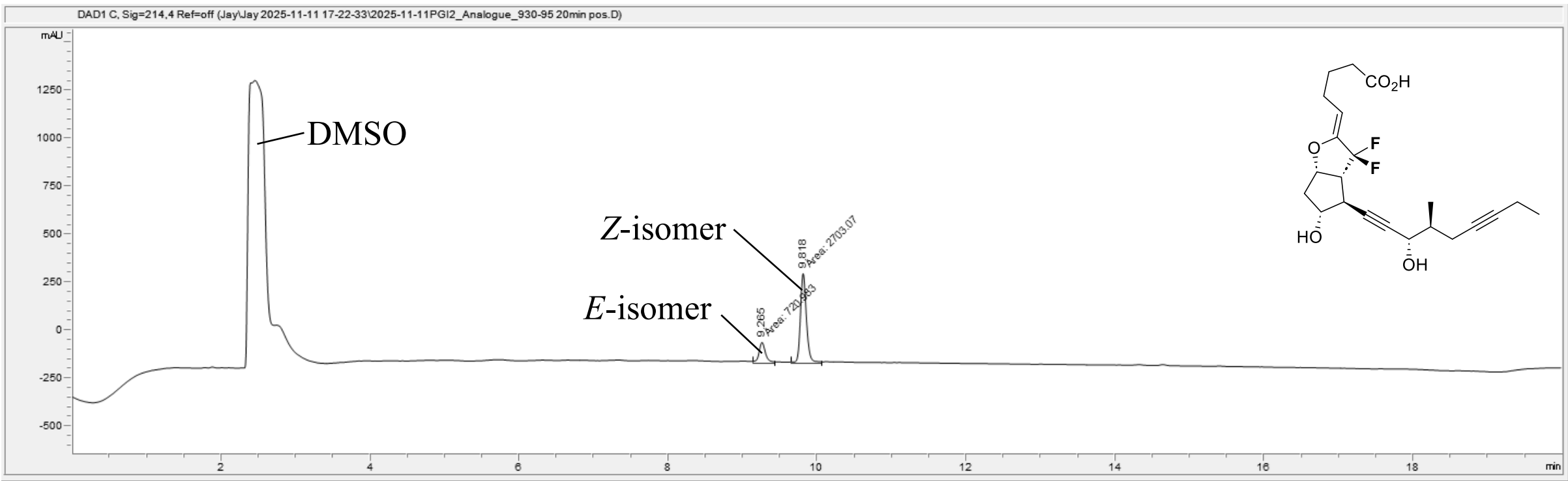

Peak #	RetTime [min]	Type	Width [min]	Area [mAU*s]	Height [mAU]	Area %
1	9.265	MM	0.1090	720.98346	110.26656	21.0564
2	9.818	MM	0.0958	2703.06958	470.25000	78.9436
Totals :				3424.05304	580.51656	

Compound **9*E*** Purity: 97.0%

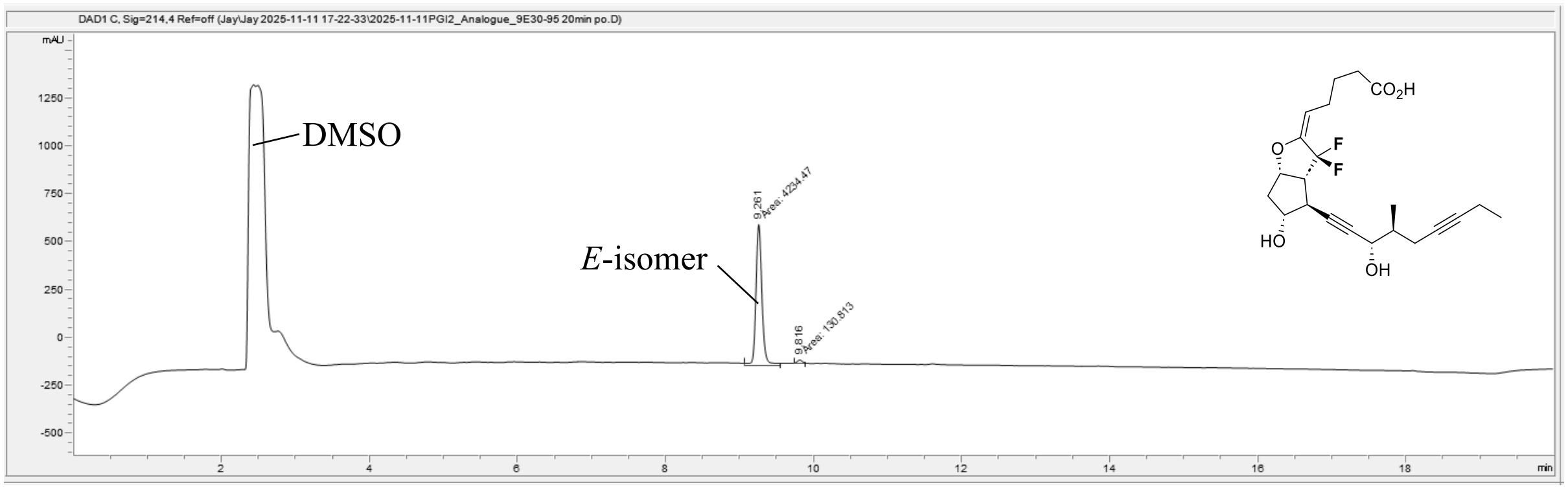

Peak #	RetTime [min]	Type	Width [min]	Area [mAU*s]	Height [mAU]	Area %
1	9.261	MM	0.0956	4234.46582	738.00024	97.0033
2	9.816	MM	0.0954	130.81334	22.86418	2.9967
Totals :				4365.27916	760.86443	

Compound **9*Z*** Purity: 100%

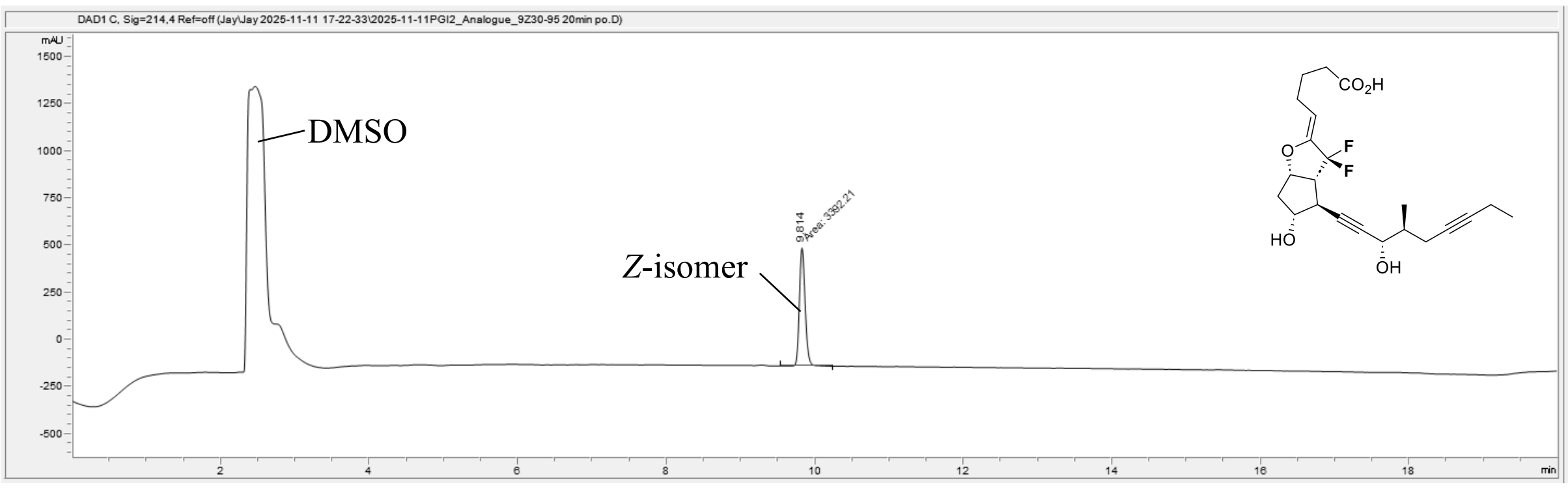

Peak #	RetTime [min]	Type	Width [min]	Area [mAU*s]	Height [mAU]	Area %
1	9.814	MM	0.0898	3392.21069	629.55048	100.0000
Totals :				3392.21069	629.55048	
